# Evolutionary trajectory and co-infection dynamics of human influenza A(H1N1) virus (2000–2025): an integrated framework informed by expert-informed bibliometrics

**DOI:** 10.3389/fmicb.2026.1793244

**Published:** 2026-03-26

**Authors:** Xinrui Cao, Jiahui Tang, Yang Liu, Xiaoyu Wang, Qi Li, Yingxue Xu, Xinran Li, Feipeng Zhao

**Affiliations:** 1 School of Basic Medical Sciences, Mudanjiang Medical University, Mudanjiang, China; 2 The First Clinical Medical College, Mudanjiang Medical University, Mudanjiang, China

**Keywords:** bibliometrics, co-detection, evidence mapping, hemagglutinin, influenza A (H1N1), multiplex diagnostics, surveillance

## Abstract

**Introduction:**

Influenza A (H1N1) remains an important seasonal respiratory pathogen, but evidence on its evolutionary dynamics, reported co-detections, and surveillance priorities remains fragmented.

**Methods:**

We conducted an evidence-mapping synthesis (2000–2025) integrating bibliometric analysis, expert-guided curation, and sequence/structure-informed interpretation. A total of 15,028 records were retrieved from PubMed, Web of Science, and Scopus, and 11,848 unique publications were retained after deduplication. GenBank-derived hemagglutinin (HA) sequences and Swiss-Model homology models were used to characterize mutational patterns and structural features. Literature-derived co-detection records were extracted from eligible publications and interpreted using a method-aware framework.

**Results:**

A post-2010 shift in the HA mutational landscape was observed, with recurrent substitutions at sites including S13, S146, S160, and S202. Structure-informed comparison of representative HA models identified a conformationally flexible segment spanning residues aa190–aa226, suggesting potential relevance to the receptor-binding microenvironment. Mapping of literature-derived co-detection records showed that RSV and SARS-CoV-2 were among the most frequently reported co-pathogens; however, these proportions reflected reporting composition across heterogeneous studies rather than population-level co-infection prevalence. In a China-focused module, G219A in Eurasian avian-like (EA) H1N1 strains was prioritized through protocol-constrained expert annotation requiring isolate-level evidence and was interpreted as a hypothesis-generating site of interest within the receptor-binding region rather than an algorithm-derived global bibliometric signal.

**Discussion:**

This study provides an integrated overview of H1N1 research evolution, HA mutational change, and reported co-detection patterns over the past 25 years. The findings support a tiered, method-aware multi-pathogen surveillance framework for preparedness, while underscoring that heterogeneous literature-derived co-detection data require standardized definitions, assay-aware interpretation, and local calibration before translation into clinical or public health decision-making.

## 1 Introduction

Influenza A (H1N1) remains a major seasonal respiratory pathogen and continues to impose recurrent clinical and public health burdens worldwide (Shahzaib, 2021; Michaelis et al., 2009). The 2009 pandemic provided a vivid reminder of how rapidly a novel reassortant lineage can disseminate: after its emergence in the United States and Mexico (Dawood et al., 2009; Petrosillo et al., 2009), early reports soon spanned multiple continents (Sedyaningsih and Setiawaty, 2009; Petrosillo et al., 2009). By May 2009, infections had been documented in 33 countries (Kou et al., 2009), and the World Health Organization subsequently declared a Phase 6 pandemic (Petrosillo et al., 2009). Although the pandemic wave subsided, H1N1 did not disappear; instead, it established itself as an entrenched seasonal virus with substantial heterogeneity in transmission and evolutionary turnover across regions. For example, genomic surveillance in Kenya (2009–2018) revealed frequent introductions and multiple co-circulating lineages (Qiu et al., 2019), while India reported a considerable burden during 2010–2017, including notable mortality in some settings (Gohil et al., 2017; Chatterjee et al., 2019). Even in temperate regions, continued lineage turnover has been observed, such as the emergence of a distinct 2009-lineage-derived variant in northern Germany in 2014 (Zell et al., 2020). In China, the first cases reported in June 2009 were followed by rapid domestic spread facilitated by extensive transportation networks (Cui et al., 2011), and influenza remains among the most prevalent notifiable infectious diseases with sustained socioeconomic impact (Qu et al., 2025). In the post-COVID-19 era, the concepts of an ‘immune gap’ or ‘immunity debt’ have been discussed to explain transient changes in population susceptibility after periods of reduced circulation of common respiratory pathogens (Cohen et al., 2021). Here, “immune gap/immunity debt” refers to the hypothesized temporary increase in susceptibility to common respiratory pathogens following periods of reduced exposure and transmission, noting that the concept is debated and cannot be causally assessed within our bibliometric design. However, the magnitude and causal relevance of this mechanism remain debated, and observed post-2022 increases in reported respiratory-virus activity may also reflect relaxation of non-pharmaceutical interventions (NPIs), shifts in healthcare-seeking behavior, changes in testing intensity, and altered viral interference patterns. Together, ongoing antigenic drift and changing population immunity make it timely to reassess H1N1 using an approach that connects viral evolution, multi-pathogen contexts and surveillance priorities.

At the mechanistic level, influenza A(H1N1) is a segmented, negative-sense RNA virus (Orthomyxoviridae) encoding polymerase subunits (PB2, PB1, PA), nucleoprotein (NP), matrix proteins (M1, M2) and nonstructural proteins (NS1, NS2) that coordinate replication and host interactions (Arias et al., 2009). Viral fitness and host adaptation are strongly shaped by the surface glycoproteins hemagglutinin (HA) and neuraminidase (NA) (Thanyada et al., 2011; Echeverry, 2011). HA is a metastable homotrimer that mediates attachment and entry (Wang et al., 2021; Echeverry and Rodas, 2012). The HA1 subunit contains the receptor-binding site (RBS), whereas HA2 drives membrane fusion after endocytosis (Gambaryan et al., 2018; Raj et al., 2023). Binding preference for specific sialic-acid linkages (e.g., human-type α2,6) is a key determinant of tissue tropism and cross-species transmission (Yang et al., 2009; Al Khatib et al., 2018; Furuse et al., 2010a). Following endocytosis, low pH triggers conformational rearrangements that expose the fusion peptide, enabling membrane fusion and uncoating (Raj et al., 2023; Shen et al., 2009; Furuse et al., 2010b). Because HA is a dominant target of neutralizing antibodies (Raj et al., 2023), immune selection pressures continually shape HA diversity and promote antigenic drift (Strengell et al., 2011). Notably, even single amino-acid substitutions can have measurable phenotypic consequences (Al Khatib et al., 2018); for example, E47K in HA2 has been associated with altered fusion pH sensitivity, potentially affecting entry efficiency (Gambaryan et al., 2018). These properties motivate high-resolution mapping of HA mutational landscapes to support risk assessment and surveillance prioritization.

Evolutionary plasticity is exemplified by the pandemic 2009 H1N1 lineage (pdm09), which emerged through reassortment involving swine, human and avian influenza lineages (Zhang et al., 2023). Beyond pdm09, recent surveillance has highlighted concern about Eurasian avian-like (EA) H1N1 lineages (e.g., G4 genotype) due to evidence consistent with increased binding to human-type receptors and possible lowering of the species barrier (Wang et al., 2021). Specific substitutions have been discussed in this context; Q223R has been linked to altered receptor binding and replication-related phenotypes in experimental systems and G219A has been reported to increase replication efficiency and pathogenicity in mammalian models, which we discuss later as a China-contextualized, expert-prioritized monitoring marker rather than a global text-mining–derived top signal (Wang et al., 2021; Wang et al., 2013). At the population level, antigenic remodeling can reduce the effectiveness of vaccine-induced immunity, complicating immunization strategies (Wang et al., 2021).

Clinically, H1N1 infection also occurs within a broader ecosystem of co-circulating respiratory pathogens. Co-detections among influenza viruses have been documented during co-circulation, including overlap with H3N2 (Eşki et al., 2021; Baala et al., 2020) and influenza B in some seasons (Santus et al., 2024). Co-detections with other respiratory viruses—such as RSV and human metapneumovirus—have been associated with severe manifestations, particularly in pediatric populations (Zhang et al., 2023; Basu et al., 2017), while adenoviruses and rhinoviruses may complicate clinical courses through interactions with host immune responses (Zhang et al., 2023; Lin et al., 2015). Since the COVID-19 pandemic, SARS-CoV-2 and seasonal coronaviruses have further contributed to a complex respiratory pathogen landscape (Zhang et al., 2023). Secondary bacterial infections remain an important driver of adverse outcomes, with *Streptococcus pneumoniae* and *Staphylococcus aureus* frequently implicated (Van Dongen et al., 2019; Mueller Brown et al., 2022). These multi-pathogen contexts have implications for method-aware diagnostics and preparedness planning, especially when evidence is drawn from heterogeneous study designs and testing strategies.

Despite extensive work on H1N1, an integrated, high-resolution mapping that links (i) global research dynamics, (ii) sequence- and structure-informed HA evolution and (iii) reported multi-pathogen contexts remains limited. Bibliometric indicators can summarize shifts in scientific attention across time and regions, but they should not be interpreted as direct proxies for disease incidence. To address this gap, we conducted a comprehensive scoping review and evidence-mapping synthesis following scoping review reporting principles (Tricco et al., 2018). Specifically, we aimed to: (i) characterize global and regional research priorities over time; (ii) connect temporal shifts in research focus with sequence/structure-informed signals in HA evolution; and (iii) map reported co-detection patterns in the literature to inform a tiered, method-aware surveillance and diagnostic framework.

## 2 Materials and methods

### 2.1 Study design and reporting framework

We performed an evidence-mapping scoping review to synthesize heterogeneous evidence on human influenza A(H1N1) from 2000 to 2025, combining bibliometrics, expert curation, and sequence/structure-informed interpretation. Reporting followed PRISMA-ScR principles (Tricco et al., 2018). The corpus assembly and analytic subset extraction workflow are summarized in Supplementary Figure S1 (corpus-flow diagram), and prespecified eligibility criteria are provided in Supplementary Table S2.

In this manuscript, we primarily use the term “co-detection record” to denote literature-derived, co-reported detections extracted from included publications. We reserve the term “co-infection” only for instances where an included study explicitly confirmed concurrent infection in the same clinical specimen with a clearly reported denominator and testing strategy.

### 2.2 Literature search and database assembly

PubMed, Scopus, and Web of Science (ISI) were searched using “(human) AND ‘influenza virus H1N1’”. Searches were run programmatically in Python. Metadata and abstracts were downloaded as bib and.txt files, respectively. We imported these files into R (version 4.4.3) and used bibliometrix to extract and harmonize publication metadata, including journal, year, title, abstract, affiliations, and author fields, and to standardize outputs to a PubMed-like structure (Aria and Cuccurullo, 2017).

A total of 15,028 records were retrieved before deduplication. Database comparability is described in Supplementary File S3. Deduplication was performed in the harmonized dataset using identifiers where available (DOI/PMID) and title-based matching otherwise, leaving 11,848 unique publications for downstream analyses.

### 2.3 Study selection, eligibility criteria, and screening

Because this study is an evidence-mapping synthesis built on a bibliometric corpus (used for publication-level analyses) rather than a conventional clinical meta-analysis, we did not apply full-text exclusions to reduce the corpus itself; instead, we applied prespecified screening when deriving analytic subsets (e.g., co-detection and sequence/structure subsets). Two reviewers independently screened titles/abstracts of records according to prespecified eligibility criteria (Supplementary Table S2). Full texts were consulted when abstracts were insufficient to confirm extractability (e.g., whether co-detection pathogens were explicitly reported) or to resolve ambiguous study context, particularly during analytic subset derivation. Disagreements were resolved by consensus, with adjudication by a third reviewer when needed. The corpus assembly and subset-extraction workflow are summarized in Supplementary Figure S1.

### 2.4 Country mapping, variable charting, and expert validation

Country assignment was based on matching country-name variants in titles, abstracts, and affiliation fields. Records referencing multiple countries were assigned to all relevant countries. We iteratively scraped, checked, and re-assigned entries to reduce misclassification. We also charted study-level descriptors when reported, including region of circulation, time period of reported outbreaks, and pathogens involved in mixed infections.

Because automated extraction cannot reliably separate “reporting frequency” from biological or clinical relevance, we implemented a prespecified expert curation protocol using a reproducible codebook and decision rules (Supplementary Codebook S4). Domain experts independently reviewed literature-derived co-detection records to (i) harmonize pathogen labels, (ii) remove obvious false co-occurrences, and (iii) assign context tags (e.g., administrative/policy-driven trend vs. biological/epidemiological signal; assay strategy specified vs. unspecified) for bias-aware interpretation. Inter-rater reliability was assessed and documented internally to ensure reproducibility of the expert-coding layer. For transparency, the full coding rubric, decision rules, and adjudication workflow are provided in Supplementary Codebook S4; numeric agreement statistics are available from the authors upon reasonable request. This curation was applied as an annotation and quality-control layer and did not modify the underlying bibliometric counts. Homology modeling (Section 2.7) was used to support structure-informed interpretation of recurrent HA changes; it was not used as experimental validation.

### 2.5 Regional and long-term publication trend modeling

Annual publication counts were analyzed using generalized linear models (GLMs) for count data. We compared Poisson and negative binomial specifications and, given marked overdispersion, adopted a negative binomial model as the primary approach. Model diagnostics and fit statistics are reported in Supplementary File S5 (Supplementary Figures S2-S4) (Muggeo, 2008; Cameron and Trivedi, 2013). We describe the publication series as exhibiting a descriptive, phase-like pattern (e.g., the 2009–2012 surge) rather than statistically confirmed breakpoints, and we do not interpret these trends as measures of disease incidence.

### 2.6 Co-detection evidence mapping and minimal stratified summaries

**Figure 1:**
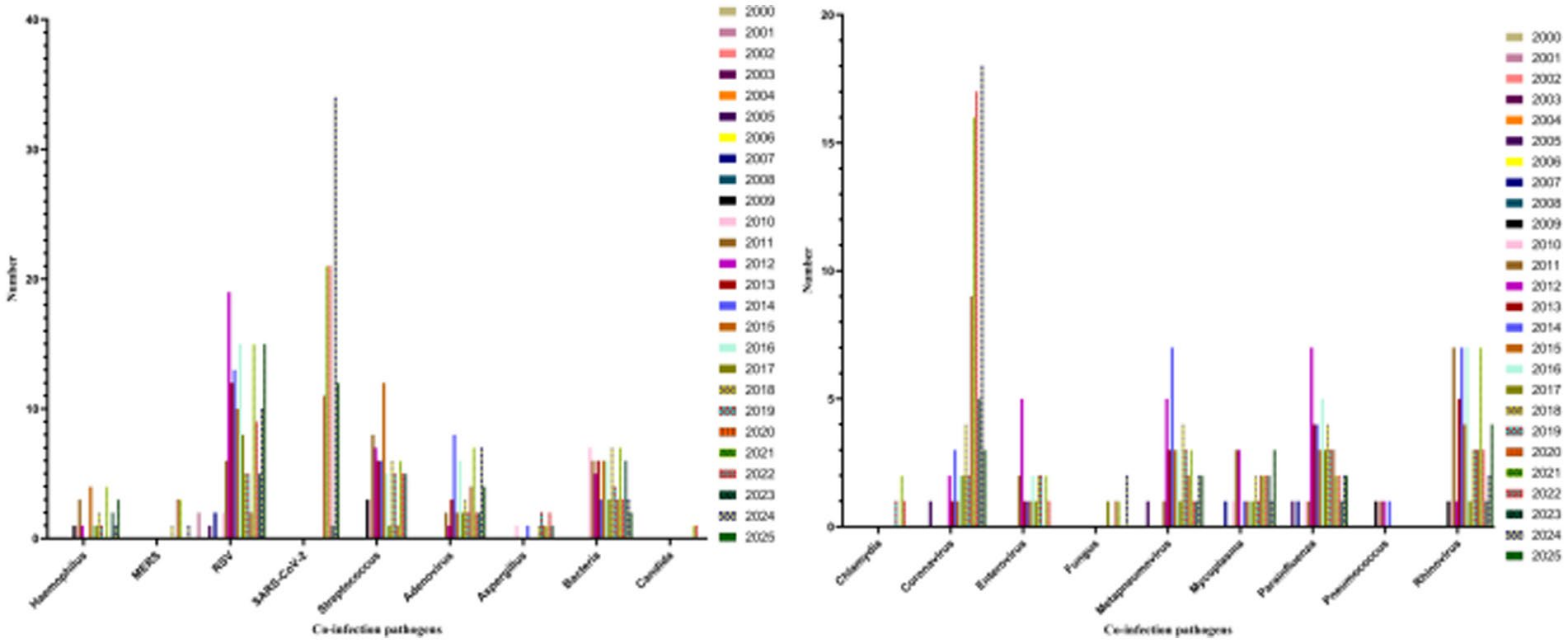
Temporal trends in literature-derived co-detection records involving human influenza A(H1N1) with other pathogens worldwide from 2000 to 2025.

We recorded viral and microbial species reported as co-detected with human influenza A(H1N1) to build an evidence map of mixed infections (Supplementary File S6), and used this information for assay-aware qualitative interpretation of post-2020 trends (Figure 1), acknowledging that changes in testing breadth and intensity can inflate apparent co-detection frequency.

### 2.7 HA sequence dataset, alignment, and mutation annotation

HA reference sequences of circulating H1N1 strains from 2000 to 2025 were retrieved from NCBI GenBank (Release 247.0) (Supplementary File S7). Multiple sequence alignment was performed using Lasergene DNASTAR™ 5.06 (DNASTAR Inc., Madison, WI, United States) and the DNAMAN 6.0 alignment module (Lynnon BioSoft, San Ramon, CA, United States). Amino-acid substitutions were annotated using established nomenclature (Den Dunnen and Antonarakis, 2001). Figures were prepared in GraphPad Prism 8.0 (GraphPad Software, Inc., La Jolla, CA, United States).

Phylogenetic inference was not performed; therefore, we did not make lineage-level claims.

### 2.8 Definition of recurrent substitutions

A substitution was classified as recurrent if it met all of the following criteria: (i) it was observed in at least two non-overlapping time windows (e.g., ≥2 distinct years or ≥2 multi-year bins), and (ii) it exceeded a minimum frequency threshold within at least one window (e.g., ≥1% of sequences in that window). Because public databases are subject to uneven sampling across regions and years, recurrence was interpreted in a bias-aware manner, and we avoided over-interpreting region-specific spikes without broader temporal support.

### 2.9 Sequence filtering and quality control

GenBank-derived HA sequences were filtered to retain: (i) human-host isolates; (ii) complete HA coding sequences; (iii) valid collection year metadata; and (iv) sequences passing basic quality control (e.g., removal of records with excessive ambiguous residues or obvious truncations). To reduce redundancy, identical HA amino-acid sequences within the same year (and, where available, the same country/region) were collapsed to a single representative for mutation-annotation summaries. The filtering workflow and final sequence counts per period are reported in Supplementary Codebook S4, Supplementary File S7.

### 2.10 Homology modeling and structure-informed interpretation

We selected representative HA amino-acid sequences that were consistently detected across 2000–2025 and designated them as 2000_H1N1-strain, 2010_H1N1-strain, and 2020_H1N1-strain (Supplementary File S6). Homology models were generated using Swiss-Model (accessed October 16, 2025) (Biasini et al., 2014). The HA protein of human influenza virus A/Hickox/1940 (PDB ID: Q0HD60) was used as the modeling template. Structural comparisons were used to inform interpretation of whether recurrent substitutions clustered in regions plausibly related to receptor-binding microenvironments or conformational dynamics. No receptor-binding, fusion, or fitness assays were performed.

## 3 Results and discussion

### 3.1 Global research trends and bibliometric landscape (2000–2025)

**Figure 2:**
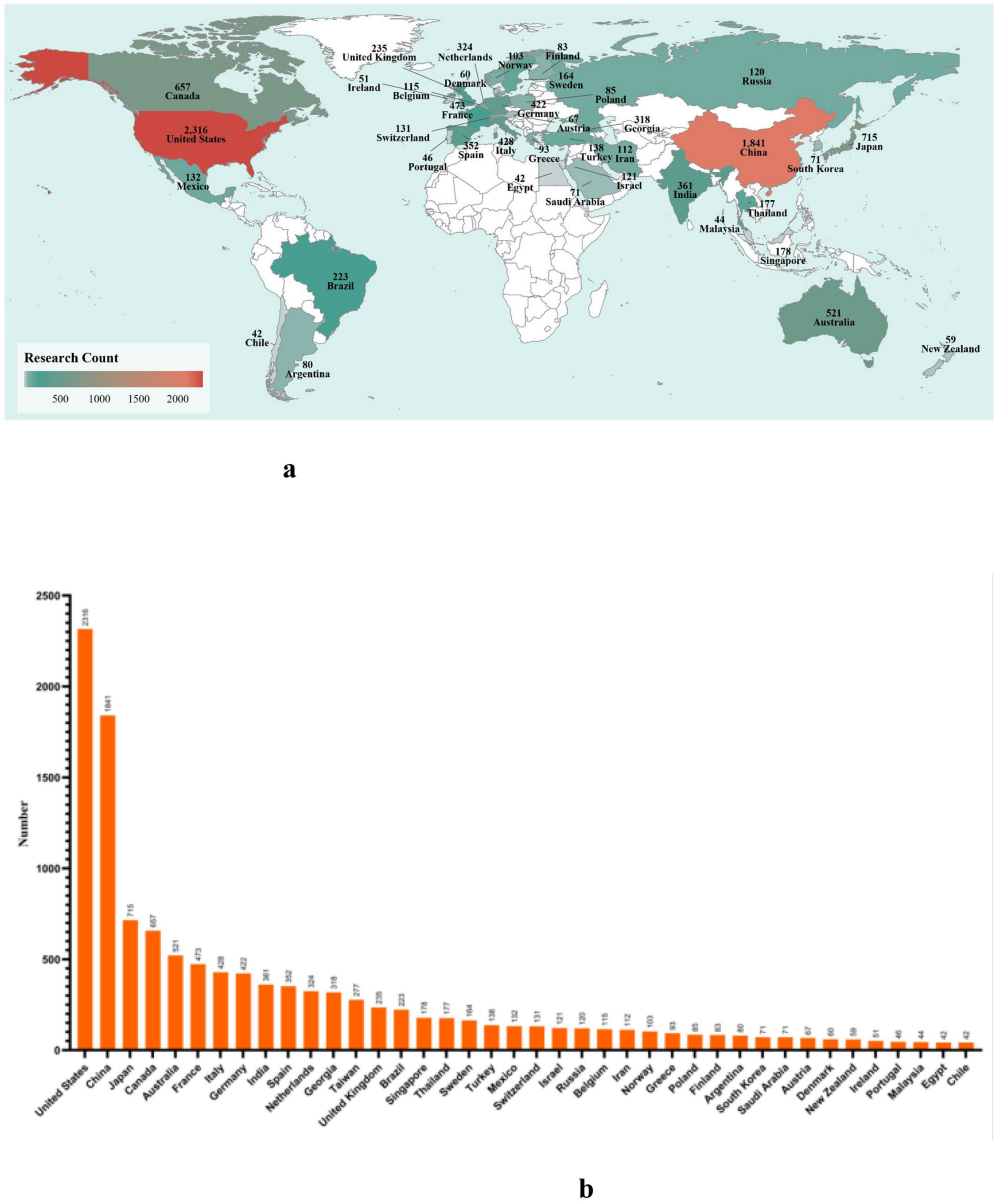
Global publication trends and geographic distribution of human H1N1 influenza virus research from 2000 to 2025. **(a)** Geographic distribution of countries contributing the majority of H1N1 research publications. **(b)** Total number of publications from the top 40 countries/regions ranked by publication count.

In total, this analysis included 11,848 publications spanning from 2000 to September 2025 (Supplementary File S3). To assess regional research priorities over the past 25 years, the top ten countries were identified based on publication counts: United States (*n* = 2,316), China (*n* = 1,841), Japan (*n* = 715), Canada (*n* = 657), Australia (*n* = 521), France (*n* = 473), Italy (*n* = 428), Germany (*n* = 422), India (*n* = 361), and Spain (*n* = 352). Geographically, Europe contributed the largest number of countries (*n* = 4), followed by Asia (*n* = 3), while Oceania was represented by a single country (*n* = 1) (Figures 2a,b).

### 3.2 Temporal evolution of research output (2000–2025)

**Figure 3:**
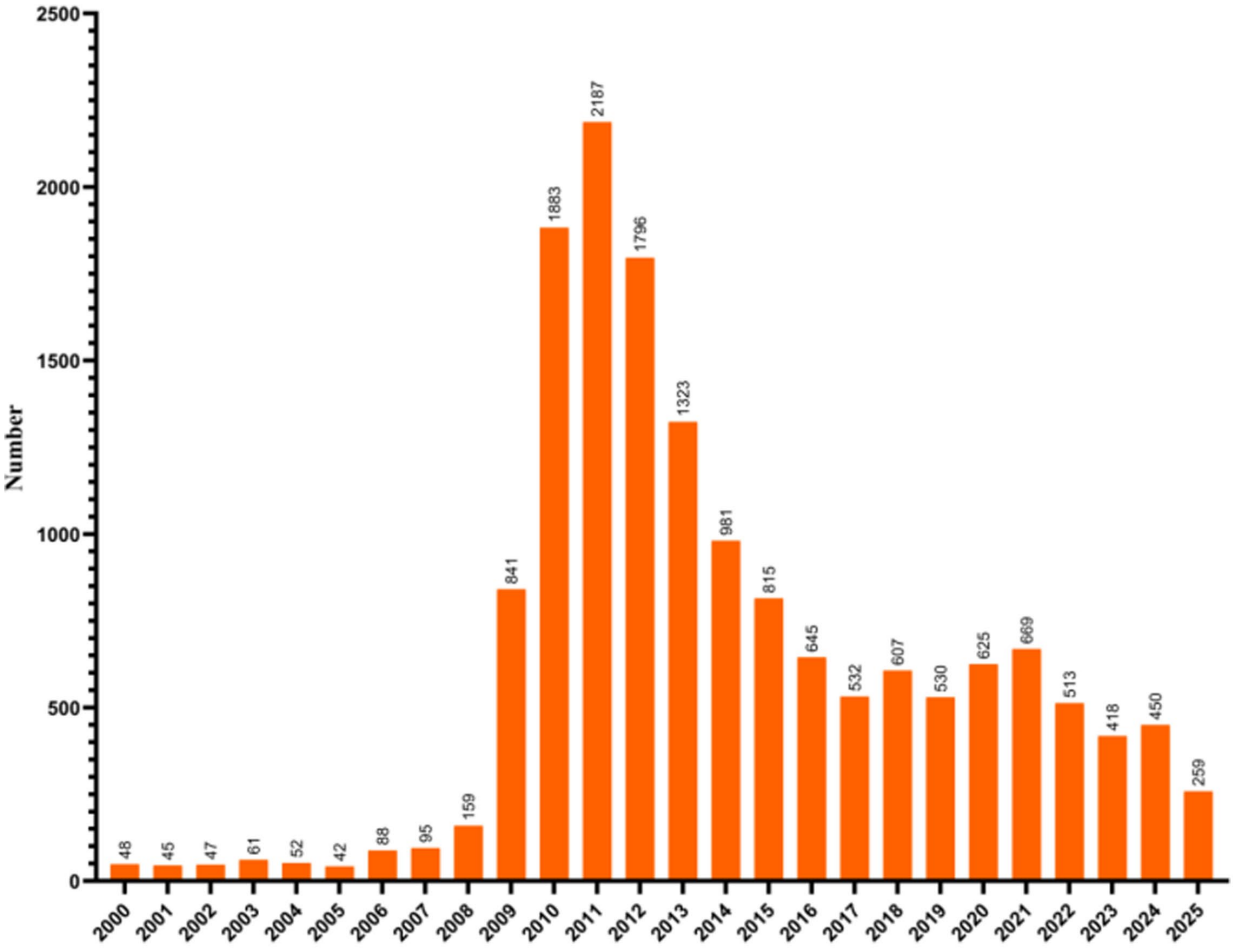
Annual number of publications on human H1N1 influenza virus worldwide between 2000 and 2025.

**Figure 4:**
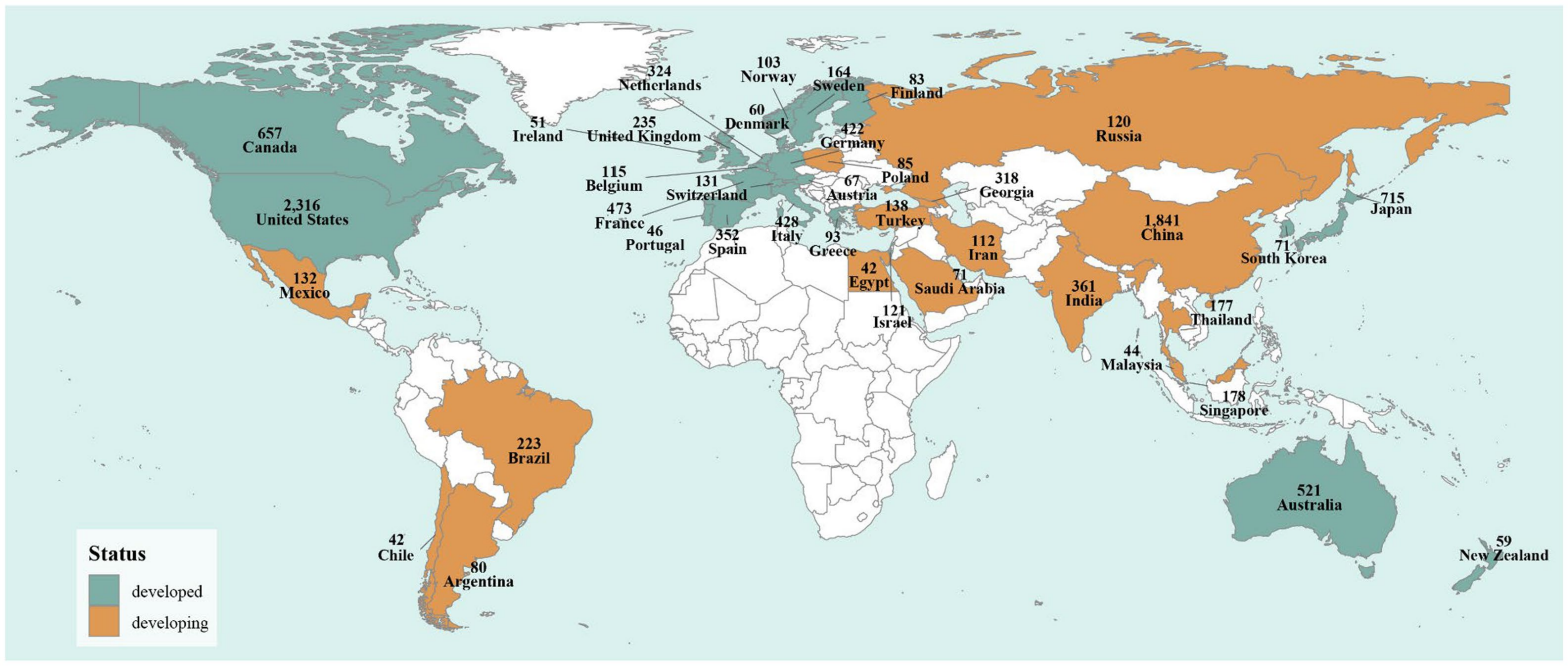
Economic development level of the regions where human influenza virus H1N1 publications were produced during 2000–2025 worldwide.

Annual publication counts exhibited a descriptive, phase-like temporal pattern characterized by an event-driven surge around 2009–2012 followed by a return toward a stable baseline (Figure 3, Supplementary File S3). The annual publication series shows a pronounced surge around the 2009 pandemic period followed by a return toward a stable baseline level, consistent with an event-driven, phase-like pattern in research output (Supplementary File S3; Figures 2–4).

### 3.3 Spatiotemporal dynamics of H1N1 infection

To characterize the global epidemiological footprint and the corresponding scientific response to human H1N1 influenza, this section delineates the distribution of research outputs from 2000 to 2025 across multiple dimensions. By examining global dissemination patterns, the temporal evolution of research priorities, and specific regional anomalies, we provide a comprehensive synthesis of how public health crises, economic factors, and viral evolution have collectively shaped the landscape of H1N1 research over the past quarter-century.

#### 3.3.1 Global dissemination and regional research priorities

The 2009 H1N1 pandemic triggered an unprecedented global health crisis, characterized by its rapid dissemination across more than 200 countries within a remarkably short timeframe. This global expansion was not uniform; rather, it was shaped by a complex interplay of international transportation networks, national economic capacities, and regional industrial priorities, particularly in countries with significant livestock sectors. Following the 1918 “Spanish flu” pandemic, Spain gradually established a modern public health system during the 20th century, which may have influenced the country’s influenza research output. The 2009 H1N1 pandemic led to rapid viral dissemination to over 200 countries and regions within a few months. For example, by May 8, 2009, 2,371 confirmed cases of H1N1 influenza had been reported across 24 countries and regions spanning the Americas, Europe, Oceania, and Asia, with Canada, the United Kingdom, and France among the early affected nations. The widespread circulation of H1N1 prompted intensified research in these affected countries. Studies indicate that the 2009 outbreak strain of human H1N1 influenza arose from genetic reassortment among swine, avian, and human influenza viruses. Countries such as China, the United States, Spain, and France, which are major producers of pigs and poultry, have research outputs likely driven in part by the livestock industry’s focus on associated infectious diseases. At the economic level, publications from developed countries accounted for approximately 60% of the total, while less-developed and developing regions contributed the remaining 40% (Figure 4, Supplementary File S3). This disparity likely reflects uneven research resources and data infrastructure associated with varying levels of economic development.

#### 3.3.2 Temporal evolution of research output (2000–2025)

Annual publication counts showed an early low-output period prior to 2009, followed by a pandemic-associated surge around 2009–2012 and a subsequent return toward baseline with intermittent fluctuations (Figure 3). Because this bibliometric series is influenced by heterogeneous reporting and evolving database coverage, we describe the pattern as descriptive and phase-like rather than a confirmed breakpoint structure. Model-based trend assessment using generalized linear models indicated marked overdispersion and supported a negative binomial specification as the best-fitting model (AIC = 325.92), while the overall yearly coefficient was 0.0327 (*p* = 0.435) (Supplementary Files S5, S7).

Between 2000 and 2008, research on H1N1 was extremely limited, with fewer than 100 publications globally per year, primarily originating from developed countries such as the United States, Europe, and Japan. Following 2009, the number of publications increased annually, reaching a peak in 2011, during which nearly all countries contributed to H1N1 research. From 2011 to 2015, publication counts gradually declined but remained above pre-2008 levels. During this period, research focus shifted toward vaccines, antiviral drugs, and viral mutations. Between 2016 and 2025, publication numbers stabilized at a relatively low level, while research priorities evolved to include seasonal influenza surveillance, cross-species transmission, and viral evolution (Figure 3, Supplementary File S3). The 2009 H1N1 pandemic represented the first global influenza outbreak since the 1968 H3N2 pandemic. The pandemic strain, a reassortant of swine- and avian-origin H1N1 viruses, prompted an immediate response from national research institutions and public health systems, resulting in a sharp surge in publications between 2009 and 2010, peaking in 2010. During this period, countries including the United States, China, Japan, Canada, and the United Kingdom rapidly established national influenza surveillance networks, initiated vaccine development programs, and stockpiled antiviral drugs. For instance, the U. S. Centers for Disease Control and Prevention (CDC) launched the “H1N1 Influenza Emergency Response Plan,” funding extensive basic and clinical research. In China, H1N1 was classified as a Class B infectious disease, leading to the establishment of a national sentinel surveillance system and vaccine production capacity. Similarly, Japan, the European Union, and Australia invested substantial research funding to promote related publications from their institutions. After 2010, H1N1 transitioned to a seasonal virus and perceived public health urgency likely decreased in many settings. Accordingly, research themes shifted from emergency-response topics to long-term surveillance, vaccine effectiveness evaluation, and mutation tracking; however, this interpretation is hypothesis-generating and may also reflect broader funding and agenda shifts. From 2020 onward, the COVID-19 pandemic coincided with broad disruptions to respiratory-virus surveillance, clinical testing, and publication priorities; therefore, we avoid attributing post-2020 changes in H1N1 publication volume to any single explanatory factor (including topic displacement by SARS-CoV-2), which is outside the inferential scope of this evidence-mapping analysis. Nevertheless, during 2020–2022, H1N1 publications showed a modest resurgence that co-occurred with increased cross-referencing of influenza and SARS-CoV-2 topics (e.g., comparative discussions of transmission, vaccine platforms, and antivirals), as well as renewed seasonal-surveillance interest in some settings.

#### 3.3.3 Case studies: counter-cyclical trends in Japan and France

While global research intensity generally declined post-2015, specific regions such as Japan and France exhibited notable “counter-cyclical” surges in research activity driven by localized epidemiological anomalies. These regional spikes were frequently associated with the emergence of highly pathogenic variants, the detection of antiviral resistance, and complex co-circulation events involving multiple respiratory pathogens. In Japan, publication counts showed a modest resurgence in 2015 and during 2017–2020, in contrast to the continuing decline observed in most countries in the Americas and Europe. This “counter-cyclical” trend closely coincided with two prominent seasonal peaks of A(H1N1)pdm09 in Japan during 2015 and 2018–2019, as well as with the government’s timeline for implementing enhanced influenza and novel influenza countermeasures (Supplementary File S3). During this period, viral isolates collected by Japanese researchers in 2015 exhibited higher pathogenicity than the 2009 pandemic strain, raising concerns about “viral resurgence” among local scientists. Subsequent animal experiments, whole-genome sequencing, and functional analyses of viral proteins directly contributed to the increased publication output in 2015 (Mukaka, 2012). In the 2018–2019 influenza season, multiple regions in Japan, including Kanto and Kansai, reported A(H1N1)pdm09 as the predominant strain, along with cases showing reduced sensitivity to oseltamivir. These findings prompted studies on antiviral resistance mechanisms and the development of novel neuraminidase inhibitors (Muawan et al., 2025), resulting in a concentration of related publications around 2020. Moreover, several studies reported that the 2017–2018 RSV-A epidemic in Japan involved a majority of patients co-infected with influenza viruses, which objectively increased the frequency of H1N1 reporting. Similarly, during the same influenza season in France, co-circulation of A/H1N1 and B/Yamagata strains contributed to a notable rise in publications from the region (Goldstein, 2022). The RSV-A infection peak and repeated influenza co-infection events during this period collectively drove the concentration of associated research outputs in 2019–2020.

### 3.4 Mutational landscape and antigenic drift of the HA protein

**Figure 5:**
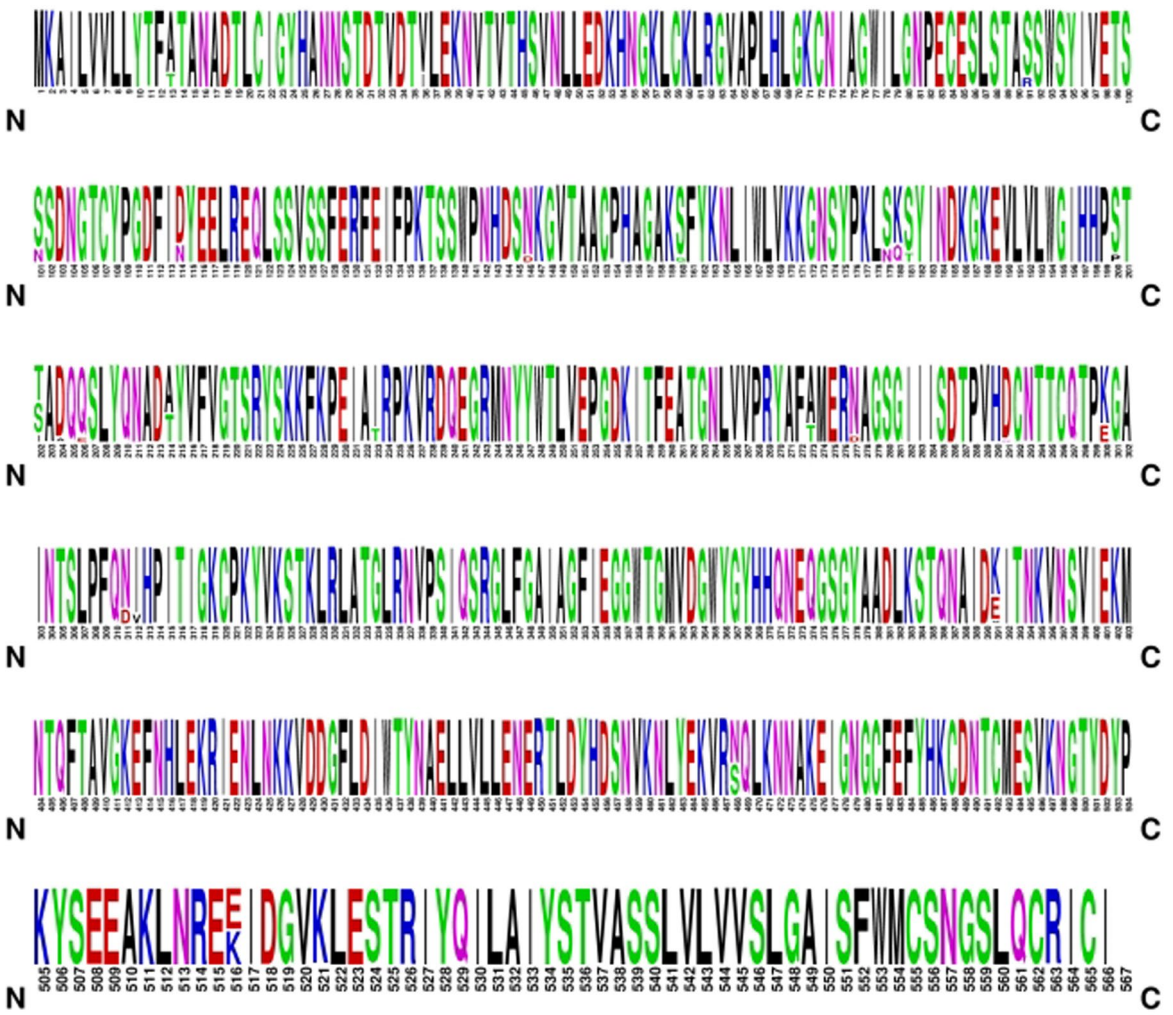
Amino acid mutations in the HA protein of human influenza virus H1N1 from 2000 to 2025.

To characterize the evolutionary patterns of the hemagglutinin (HA) protein of globally circulating H1N1 influenza viruses over the past 25 years, we retrieved all available HA amino acid sequences of H1N1 strains collected between 2000 and 2025 from GenBank. Comprehensive analyses suggest a relatively stable evolutionary trajectory across the study period in the available GenBank-derived HA sequence dataset. Across the 25-year span, HA evolution was primarily characterized by recurrent amino acid substitutions at positions S13, S36, S91, S101, S114, S146, S160, S170, S180, S181, S200, S202, S291, S300, S311, S312, S391, S488, and S516 (Figure 5). The detailed mutation profiles are summarized in Supplementary File S6. In addition, the mutation frequencies at these positions were calculated, and the statistical results are provided in Supplementary File S7 (Figure S5-S8).

To characterize the temporal dynamics of amino acid variations in the H1N1 HA protein, we analyzed the decade-specific mutation patterns from 2000 to 2025. During 2000–2009, only a limited number of amino acid substitutions—V36I, A214T, and E391V—were detected in circulating human H1N1 HA sequences. In contrast, the HA protein exhibited substantial diversification between 2010 and 2019. A total of 18 new mutation sites emerged during this period, including S13, S91, S101, S114, S146, S160, S179, S180, S200, S202, S233, S273, S277, S300, S311, S312, S468, and S516, with position S214 showing continued variability (Supplementary File S6). After 2020, the set of newly observed HA substitution sites appeared to stabilize in this GenBank-derived dataset, with eight additional substitutions identified at S48, S103, S154, S222, S319, S391, S490, and S497. Overall, the mutation landscape of the HA protein displayed a degree of “disorderliness,” characterized by the continual appearance of new mutation sites each decade. This seemingly stochastic mutational pattern, combined with the gradual accumulation of novel substitutions, may reflect the virus’s adaptive strategy under persistent host immune pressure, enabling rapid trial-and-error exploration for improved fitness. The apparent post-2020 stabilization is hypothesis-generating and may reflect altered sampling intensity, reduced circulation in some settings, or an adaptive equilibrium; without bias-adjusted sampling and formal phylodynamic modeling, this should not be interpreted as a confirmed reduction in evolutionary pressure (Gambaryan et al., 2018).

Using 2010 as a critical temporal breakpoint, we identified a cluster of mutations—S13, S91, S101, S114, S146, S160, S179, S180, S200, S202, S233, S273, S277, S300, S311, S312, S468, and S516—that may represent key sites driving the substantial genetic shifts observed in post-2010 H1N1 strains. As time progressed, additional substitutions continued to emerge at other positions, raising the possibility that these mutations could further influence viral virulence, host adaptation, and transmissibility (Koel et al., 2013).

In summary, our analysis suggests that the amino acid evolution of the H1N1 influenza virus HA protein is a dynamic and continuous process, with alterations at key sites that may influence viral antigenicity and, in some contexts, may be associated with changes in pathogenicity or transmissibility. A deeper understanding of these evolutionary patterns—particularly the functional implications of mutations that emerged after 2010—will provide essential scientific insights for improving influenza vaccine design and informing public health strategies (Qiu et al., 2019).

### 3.5 Evaluation of HA molecular structure and conformational dynamics

**Figure 6:**
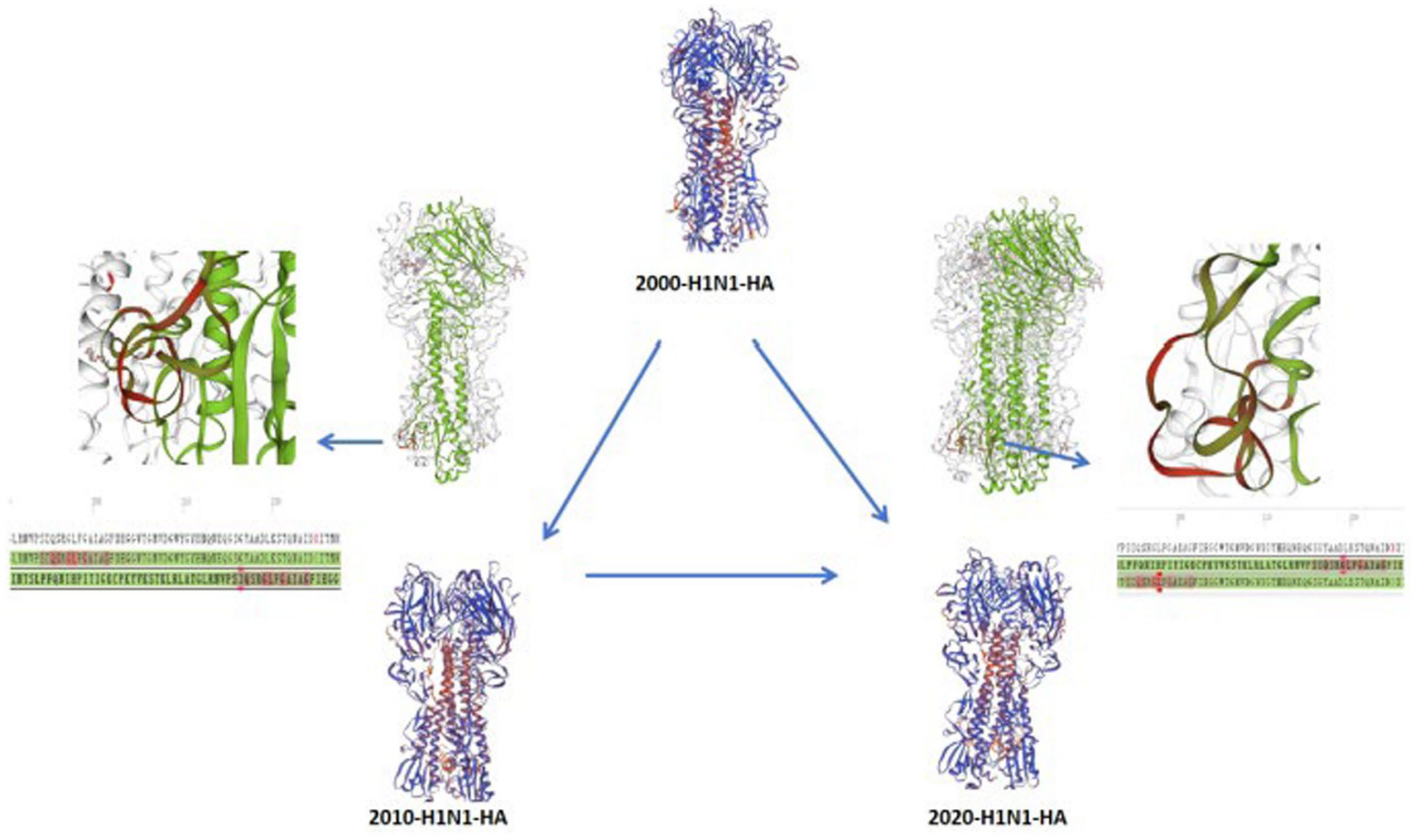
Structural alterations of the H1N1 influenza virus HA protein from 2000 to 2025.

To clarify how cumulative amino acid substitutions drive structural alterations in the H1N1 HA protein, we performed homology modeling of HA sequences spanning the past 25 years. Specifically, a cluster of mutations within residues aa190–aa226 resulted in a conformational shift from a *β*-sheet to a random-coil structure, leading to pronounced alterations in the overall HA architecture (Figure 6). Conformational changes in the HA protein, particularly those within the globular head region, directly influence receptor-binding specificity and viral immunogenicity (Strengell et al., 2011; Igarashi et al., 2010; Thompson et al., 2024).

#### 3.5.1 Conformational transitions and receptor specificity

Amino acid changes occurring in key structural elements—such as the 190-helix, 150-loop, 130-loop, and 220-loop—directly modulate the affinity and specificity of viral binding to host cell receptors, including sialic acid (Takayama et al., 2021). For instance, the D222G/N mutations in the HA protein of the 2009 pandemic strain have been associated with increased disease severity and mortality (Takayama et al., 2021; Matera et al., 2023). Similarly, the D225G mutation has been demonstrated to enhance H1N1 virulence in mice (Thompson et al., 2024). While HA proteins remained relatively stable between 2010 and 2020, comparisons with the 2000 strains reveal potential structural modifications consistent with long-term adaptive evolution (Kistler and Bedford, 2023).

#### 3.5.2 Impact of the G219A substitution in the Chinese context

Expert-provenance and positioning within the evidence map. In this study, G219A is presented as a China-specific, hypothesis-generating marker that was prioritized via protocol-constrained expert annotation within the China module (with isolate-level evidence required), rather than as an automated “top hit” statistically detected from the global bibliometric text-mining output (Wang et al., 2021). Structurally, residue 219 lies within the receptor-binding region (RBS), and a Gly → Ala substitution may plausibly modulate local steric constraints and the microenvironment of the binding pocket. Consistent with this structural plausibility, published experimental work in EA H1N1 backgrounds has reported associations between G219A and increased replication/pathogenicity and altered receptor-binding characteristics (e.g., toward human-type SAα2,6-Gal) in specific models (He et al., 2025). Accordingly, we frame G219A as a site of interest warranting targeted monitoring and further standardized validation in China’s EA H1N1 surveillance contexts, rather than as a definitive evolutionary driver inferred directly from literature frequency. Taken together, these structure-informed interpretations provide a mechanistic context for prioritizing “watch regions” and candidate sites in routine molecular surveillance, as summarized in Figure 6.

To strengthen the conceptual link between evolutionary findings and public health implications, we translate the structure- and sequence-informed signals into surveillance actionability. Recurrent substitutions at antigenic or receptor-binding–adjacent sites motivate routine sequence monitoring for drift and potential vaccine-update relevance, while structure-informed interpretation of flexible regions (e.g., the aa190–aa226 segment) provides a hypothesis-generating “watch region” for changes in the receptor-binding microenvironment. These evolutionary signals complement (rather than replace) epidemiologic surveillance and are integrated with the literature-derived co-detection evidence map to inform the tiered, method-aware preparedness framework presented below.

In conclusion, this review provides an overview of H1N1 influenza virus research and HA protein evolution over the past 25 years, both at regional and global scales. By analyzing publication trends, we documented the shifting research priorities and contextualized these trends within the temporal and geographic variations of H1N1 influenza. Nevertheless, a key limitation of this study is that, while text-mining algorithms enable rapid pattern identification and quantification across large bodies of scientific literature, they cannot replace the expert knowledge of researchers. More detailed examination of publications—such as strain-specific information, identification of emerging pathogenic lineages, or classification by research focus (e.g., epidemiology, control strategies, genetic characteristics, diagnostics, or vaccine development)—would allow for a more comprehensive understanding of H1N1 influenza virus research trends, transmission dynamics, and evolutionary patterns.

It is important to acknowledge that while the text-mining algorithms used in this study enabled the identification of patterns across 11,848 unique publications, they cannot fully replace expert knowledge. To mitigate this limitation, we have refined our findings—such as the co-infection trends and mutation landscapes—by integrating expert-curated data and molecular modeling. For instance, while publication spikes in developed countries (accounting for 60% of the total) reflect research prioritization rather than absolute incidence, we cross-validated selected literature-derived patterns against publicly available viral isolates from GenBank to provide sequence-denominator context where feasible. To strengthen biological interpretability, we implemented a prespecified post-extraction validation layer comprising (i) secondary manual curation by domain experts using the same codebook and decision rules (Supplementary Codebook S4), and (ii) structure-informed interpretation based on representative HA homology models. For example, substitutions frequently discussed in the literature (e.g., at site 202) were examined in representative HA structural contexts to assess whether their locations are consistent with regions plausibly relevant to antigenic or receptor-binding microenvironments, without implying causal confirmation. Importantly, text-mining and bibliometrics primarily capture reporting composition and are not sufficient to infer population-level prevalence or effect size; likewise, structural modeling is used here to contextualize candidate substitutions and flexible regions rather than to establish causal antigenic shifts. Therefore, this hybrid workflow supports method-aware hypothesis generation and preparedness planning, while emphasizing that translation of the Integrated Surveillance and Control Framework into clinical diagnostic protocols requires local calibration against sentinel surveillance, testing strategy, and disease-burden indicators, rather than publication frequency alone.

### 3.6 Co-infection patterns and pathogen interactions

**Figure 7:**
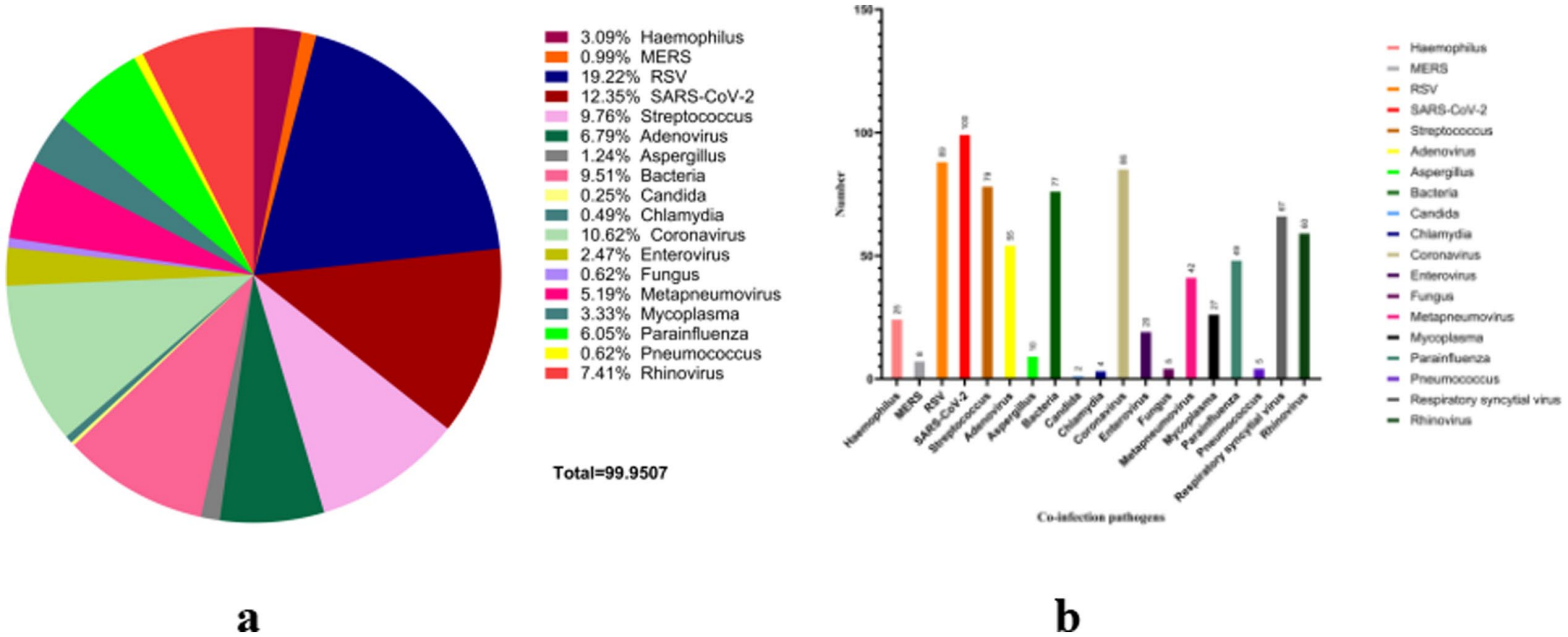
Composition and patterns of reported co-detections with human influenza A(H1N1), 2000–2025. **(a)** Composition of reported co-pathogens in literature-derived co-detection records (*N* = 810); **(b)** Types of co-detection patterns (virus–virus, virus–bacteria, virus–fungi). Percentages in panel **(a)** were calculated as counts/810 and represent the reporting composition of extracted co-detection records rather than population-level co-infection prevalence.

To characterize the multi-pathogen context of human influenza A(H1N1), we collected and compiled published reports of co-detections over the past 25 years (Supplementary File S6). Across 810 literature-derived co-detection records extracted from included publications, respiratory syncytial virus (RSV) was the most frequently reported co-pathogen (156/810, 19.26%), followed by SARS-CoV-2 (100/810, 12.35%) and *Streptococcus* spp. (79/810, 9.75%). The least frequently reported co-pathogens in the extracted records were *Chlamydia* spp. (4/810, 0.49%) and *Candida* spp. (2/810, 0.25%) (Figures 7a,b). These proportions reflect the composition of reported co-detections in the available literature rather than population-level co-infection prevalence. Accordingly, downstream framework elements derived from these records are presented as evidence-availability and surveillance-preparedness signals (research-priority informed), rather than as burden-based clinical risk ranking.

To examine whether reported co-detections varied over time and geography, we further analyzed the country–year evidence map in Supplementary File S6. After excluding influenza itself and non-informative entries (“not specified”), the evidence map yielded 428 literature-derived co-detection records that could be stratified. Record volume was sparse in early years (2000–2008, *N* = 15, 2009–2010, *N* = 10), increased substantially during 2011–2019 (*N* = 155), and remained high in 2020–2022 (*N* = 125) and 2023–2025 (*N* = 123), consistent with expanding diagnostic capacity and intensified respiratory surveillance. Nevertheless, because the included studies are heterogeneous in testing indications and assay breadth, post-2020 trends are likely confounded by the rapid scale-up of multiplex respiratory panels and broader syndromic testing. Accordingly, temporal changes in literature-derived co-detection counts should be treated as evidence-availability and detection-intensity signals, rather than as direct proxies for true epidemiological incidence or co-infection prevalence. Compositionally, SARS-CoV-2 emerged predominantly in 2020–2022 (18.40%) and remained prominent in 2023–2025 (17.07%), while RSV remained frequently reported across eras (2011–2019, 23.23%, 2020–2022, 16.00%; 2023–2025: 22.76%). Reported non-SARS/MERS coronaviruses also increased during 2020–2022 (19.20%) relative to 2011–2019 (5.16%). Country stratification showed that records were concentrated in a limited number of settings (e.g., China contributed 74/428 records), indicating that reported co-detection composition should be interpreted as evidence availability and reporting patterns rather than population-level prevalence.

The extracted records indicate diverse co-detection patterns, including virus–virus (e.g., H1N1 + RSV), virus–bacteria (e.g., H1N1 + *Streptococcus*), and virus–fungi (e.g., H1N1 + *Aspergillus*), consistent with the concept that disruption of the respiratory barrier can facilitate secondary infections with multiple pathogens (Raj et al., 2023). Co-detections involving RSV, SARS-CoV-2, and H1N1 were prominent in the compiled literature. RSV is highly transmissible among infants and older adults, and its co-circulation with H1N1 may contribute to overlapping seasonal epidemics. During the COVID-19 pandemic, SARS-CoV-2 and H1N1 were concurrently discussed and co-detected in multiple reports, reflecting overlapping testing and reporting contexts in the post-2020 respiratory surveillance literature (Tanriover et al., 2025; Pawlus et al., 2021; Matera et al., 2023). Co-detection with *Mycoplasma* spp. is frequently reported in pediatric respiratory illness and may increase diagnostic and treatment complexity. Overall, multi-pathogen co-detections highlight the need for method-aware multi-pathogen surveillance, targeted prevention strategies, and protection of vulnerable populations to mitigate severe outcomes in seasonal epidemics.

From a temporal perspective, the number of published H1N1 co-detection reports was extremely low between 2000 and 2008. Limited detection methods and an underdeveloped influenza surveillance system likely contributed to under-ascertainment and under-reporting of co-detections in the literature, rather than reflecting denominator-based population rates. Following the widespread circulation of H1N1 influenza virus during the 2009–2010 pandemic, virus–bacteria co-detection patterns gained attention. Notably, co-detections involving H1N1 and *Streptococcus* spp. persisted after 2009, consistent with sustained clinical concern regarding secondary bacterial complications following influenza. With the expansion of diagnostic technologies and the establishment of respiratory multi-pathogen surveillance systems, the number of literature-derived H1N1 co-detection records increased from 2011 to 2019 and remained elevated during 2020–2022 and 2023–2025 (Figures 1a–c). Supplementary File S6 summarizes record counts by time period and lists the most frequently mentioned co-detected pathogens, providing context for interpreting temporal changes in reporting composition. Importantly, the apparent post-2020 “increase” should be interpreted cautiously because it is highly susceptible to surveillance and testing-intensity bias, particularly the widespread adoption of syndromic multiplex RT-qPCR panels and expanded respiratory testing practices after COVID-19. Therefore, the post-2020 rise in reported co-detections likely reflects, to a substantial extent, changes in diagnostic coverage, assay breadth, and reporting practices, rather than an inferable increase in population-level co-infection prevalence.

To improve method-awareness, we interpreted Figure 1 in an assay-aware manner by extracting and summarizing the reported diagnostic strategies where available (e.g., multiplex/syndromic panels vs. targeted singleplex PCR vs. culture/other). Among studies reporting diagnostic strategy during 2020–2022, SARS-CoV-2 emerged as a prominent co-detected pathogen, consistent with co-circulation and intensified testing during the pandemic period. However, we refrain from attributing the overall post-2020 increase to biological “rebound” alone, because differences in assay adoption can inflate apparent co-detection frequency. Related hypotheses such as an ‘immune gap/immunity debt’ have been proposed to interpret post-2022 rebounds in other contexts, but they remain debated and cannot be evaluated causally using bibliometric or literature-derived co-detection data; changes in NPIs, healthcare-seeking behavior, and viral interference may also contribute. Importantly, our evidence map captures reporting composition and co-detection mentions from heterogeneous studies, and is not designed to attribute post-2022 changes to any single mechanism. Accordingly, hypotheses such as “immune gap/immunity debt” should be viewed as interpretive context rather than causal inference within this study.

Clinically, co-detections of H1N1 with RSV, *Mycoplasma* spp., SARS-CoV-2, and fungi were frequently reported in high-risk populations, including young children and immunocompromised individuals. Meanwhile, bacterial (e.g., *Streptococcus* spp.) and fungal (e.g., *Aspergillus* spp., *Candida* spp.) detections often co-occurred in the context of severe disease and antibiotic exposure. These findings support the need for method-aware, tiered multi-pathogen surveillance and targeted screening strategies, while emphasizing that literature-derived co-detection counts should not be interpreted as population-level prevalence.

## 4 Integrated surveillance and control framework

Based on cross-validation of large-scale literature signals (research-output patterns and literature-derived co-detection composition) with sequence/structure-informed interpretation of HA evolution, we propose a multi-dimensional framework to support surveillance readiness and research prioritization. Importantly, this framework is evidence-availability informed: bibliometric volume and co-detection reporting composition reflect what has been studied and reported in the literature, rather than population-level incidence or burden. Therefore, the framework should not be interpreted as a direct mandate for national clinical diagnostic protocols. Where clinical diagnostic pathways are considered, the suggested components should be locally calibrated against sentinel surveillance, healthcare utilization, and disease-burden indicators.

### 4.1 Molecular and epidemiological surveillance indicators

Effective surveillance must transition from simple case counting to a qualitative assessment of viral fitness and structural risk:

#### 4.1.1 Genetic variation intensity

Surveillance should prioritize the monitoring of key amino acid substitutions, particularly at sites like S13, S146, S160, and S202, which emerged or showed continued variability post-2010 and drive significant genetic shifts.

#### 4.1.2 Structural transition thresholds

Routine homology modeling is recommended to detect conformational shifts, such as the transition from *β*-sheets to random-coil structures observed in the aa190–aa226 region of the HA protein.

#### 4.1.3 Antiviral sensitivity profiling

Given reports of reduced sensitivity to oseltamivir in circulating strains, such as those observed in Japan during the 2018–2019 season, continuous monitoring of neuraminidase (NA) inhibitor resistance is essential.

While identifying molecular markers is critical for predicting viral fitness, the impact of these genetic shifts is most profoundly observed within specific demographic groups and ecological interfaces.

### 4.2 Priority populations and seasonal windows

The complexity of H1N1 infection and its propensity for multi-pathogen co-detections (and confirmed co-infections where explicitly demonstrated) necessitate targeted protection strategies:

#### 4.2.1 High-risk demographic prioritization

Pediatric and immunocompromised populations require intensive monitoring, as they exhibit the highest susceptibility to co-infections with RSV, *Mycoplasma*, and fungal pathogens.

#### 4.2.2 Occupational exposure monitoring

Due to the reassortant origin of H1N1 from swine, avian, and human precursors, stakeholders in the livestock industry in major producing countries should be prioritized for surveillance.

#### 4.2.3 Post-NPI resurgence windows (hypothesis-generating)

Public health systems should anticipate the possibility of atypical seasonal rebounds following periods of suppressed transmission. Such rebounds are sometimes discussed under the debated ‘immune gap/immunity debt’ hypothesis, but may also arise from relaxation of NPIs, altered healthcare-seeking behavior, expanded multiplex testing, and changes in viral interference. Therefore, preparedness planning should rely on sentinel surveillance and method-aware interpretation rather than assuming a single causal mechanism.

To effectively protect these vulnerable populations, the surveillance strategy must be operationalized through standardized, high-sensitivity diagnostic protocols capable of detecting complex pathogen interactions.

### 4.3 Laboratory diagnostic panel recommendations

To address operational challenges under multi-pathogen co-circulation, we provide evidence-informed, method-aware panel considerations (Table 1) that summarize frequently co-reported pathogens in the literature and common multiplex testing practices. These tiers are intended to support surveillance readiness and research-priority setting; they should be adapted to local epidemiology and calibrated using burden-based surveillance data when used for clinical diagnostic decision-making.

**Table 1:** Evidence-informed, method-aware panel considerations for literature-derived H1N1 co-detection contexts (not burden-ranked).

Screening category	Target pathogens	Target population	Recommended methods	Clinical significance
Core viral panel	H1N1, RSV, SARS-CoV-2	General population, especially children and elderly	Multiplex RT-qPCR	Supporting differential diagnosis and surveillance readiness under multiplex respiratory testing
Expanded respiratory panel	Adenovirus, Rhinovirus, Parainfluenza	Outpatients with influenza-like illness	Syndromic Panels	Assessing community transmission diversity
Secondary bacterial panel	*Streptococcus pneumoniae*, *Staphylococcus aureus*	Hospitalized and severe cases	Sputum/Blood culture, mNGS	Early warning of secondary bacterial pneumonia
Atypical pathogens	*Mycoplasma pneumoniae*, *Legionella* spp.	Children with severe respiratory symptoms	Serology, NAAT	Reducing misdiagnosis in pediatric cases
Fungal risk panel	*Aspergillus* spp., *Candida* spp.	Immunocompromised or ICU patients	G/GM tests, Fungal culture	Prevention of invasive fungal infections following antibiotic usage

Target pathogens listed here are derived from literature-reported co-detection composition and common multiplex respiratory testing practices. They indicate evidence availability and surveillance-readiness considerations rather than population-level co-infection prevalence or disease-burden ranking; local clinical panels should be calibrated to sentinel surveillance and burden metrics. Importantly, these literature-derived co-detection records are not denominator-based and therefore cannot be used to infer population-level co-infection prevalence, incidence, or clinical risk rankings.

### 4.4 Refined surveillance and control framework for China

Based on China’s significant contribution to H1N1 research (*n* = 1,841) and its distinct epidemiological landscape, a specialized framework is essential for mitigating future risks.

#### 4.4.1 Strategic surveillance and regional priorities

##### 4.4.1.1 Migration-driven spatiotemporal tracking

As H1N1 dissemination in China is intrinsically linked to major transportation corridors and mass population movements, surveillance systems must integrate real-time migration data into transmission dynamics models (Cui et al., 2011).

##### 4.4.1.2 Class B regulatory integration

Surveillance should leverage the established national sentinel network under the Class B infectious disease management category, focusing on the long-term monitoring of vaccine effectiveness and shifts in viral virulence (Qu et al., 2025).

##### 4.4.1.3 Zoonotic-human interface surveillance

Given China’s status as a major producer of swine and poultry, prioritizing active monitoring at the zoonotic interface is critical to identify early reassortment events, especially concerning strains with enhanced binding to human-type SA *α* 2,6-Gal receptors (Wang et al., 2021).

##### 4.4.1.4 Targeted screening in high-density hubs

Intensified screening and resource allocation should be focused on urban centers and high-density demographic hubs where the public health burden of influenza remains most pronounced (Qu et al., 2025).

#### 4.4.2 Optimized laboratory diagnostic panel (China settings)

To manage the high incidence of influenza in China, we recommend a tiered diagnostic approach that accounts for regional co-infection patterns.

The implementation of this framework in the Chinese context requires not only structural adjustments but also a rigorous validation of the data driving these regional priorities.

### 4.5 Data validation and addressing text-mining limitations (China focus)

To ensure biological relevance and interpretability in the China-focused framework, we applied the same prespecified expert curation codebook and decision rules described in Supplementary Codebook S4. Extracted records were independently coded for (i) evidence level (clinical denominator/isolate-level evidence present vs. absent), (ii) assay strategy (multiplex/syndromic panels vs. singleplex vs. unspecified), and (iii) explicit administrative/policy signals (e.g., emergency response, guideline dissemination, capacity building, or funding initiative language). Records meeting predefined administrative/policy criteria in the absence of isolate-level or clinical-denominator evidence were tagged as “administrative/policy-driven reporting trends” and treated as interpretive context rather than intrinsic viral fitness signals. This protocol-based tagging prevents conflation of publication surges with evolutionary peaks and increases the reproducibility of expert calibration in heterogeneous reporting environments.

For China-specific variant discussion (e.g., G219A), we explicitly separated term detection from evidence-based prioritization. The automated pipeline could capture mutation strings when explicitly mentioned in text; however, G219A was not treated as a statistically detected global signal from the bibliometric extraction because mutation-level mentions are sparse and heterogeneous across abstracts, and cannot be interpreted as frequency or effect-size without standardized sequence denominators. Instead, G219A entered our China module through expert-led contextual annotation governed by Supplementary Codebook S4, in which “variant-of-interest” tagging requires explicit isolate-level evidence (e.g., isolate identifiers/sequence availability and lineage context) and supporting study-type qualifiers. Therefore, any emphasis on G219A in the China framework reflects a protocol-constrained, hypothesis-generating prioritization for interpretation and targeted monitoring, rather than an algorithm-derived global bibliometric “top mutation” finding.

#### 4.5.1 Statistical verification of clinical co-detection combinations (China focus)

To mitigate “co-occurrence noise”—where pathogens are mentioned together without a biological link—we performed a secondary statistical analysis of high-quality Chinese clinical studies (e.g., Shanghai and Shandong cohorts). This verification suggests that, among the subset of higher-quality Chinese clinical studies included in our evidence map, H1N1 + H3N2 and H1N1 + SARS-CoV-2 were the most frequently reported co-detection combinations. These findings should be interpreted as evidence-availability signals within the included literature rather than as burden-ranked national risk estimates (Table 2).

**Table 2:** China module: preparedness-oriented panel considerations informed by literature-derived co-detection evidence.

Category	Target pathogens	Clinical rationale (expert-led validation)
Core viral panel	H1N1, H3N2, SARS-CoV-2	Based on surveillance data from major Chinese cities, the co-circulation of H1N1 and H3N2 is a primary driver of seasonal respiratory surges.
Zoonotic early-warning	Eurasian Avian-like (EA) H1N1, G4 Genotype	Critical for China’s livestock-heavy regions; specifically targets variants with enhanced binding affinity for Human-type SAα2,6-Gal receptors.
Secondary infection screening	*Streptococcus suis*, *Streptococcus pneumoniae*, *Staphylococcus aureus*	Expert curation highlights the high risk of *S. suis* in regions with intensive swine farming; bacterial co-infection significantly correlates with severe clinical outcomes.
Atypical and fungal risk	*Mycoplasma pneumoniae*, *Aspergillus* spp.	Recommended for pediatric cohorts and immunocompromised patients in anticipation of post-2022 rebound seasons and increased diagnostic complexity. Potential drivers discussed in the literature include a debated ‘immune gap/immunity debt’ hypothesis, relaxation of NPIs, changes in healthcare-seeking behavior, expanded multiplex testing, and altered viral interference; thus, decisions should be guided by local sentinel surveillance and standardized testing definitions.

### 4.6 Limitations

First, bibliometric outputs and literature-derived co-detection records capture reporting composition rather than denominator-based prevalence or burden. In addition, we did not perform counterfactual projection or formal deviation testing to quantify topic-displacement effects during the COVID-19 era (e.g., whether SARS-CoV-2 publication growth reduced H1N1 output relative to a projected baseline); accordingly, any discussion of post-2020 publication ecology is treated as contextual background rather than an evidence-based causal conclusion. Second, substantial heterogeneity in study designs and diagnostic strategies—especially the post-2020 expansion of multiplex respiratory testing—introduces surveillance and testing-intensity bias when interpreting temporal co-detection trends. Third, automated text-mining may misclassify entities; although expert curation improves precision and transparency (Supplementary Codebook S3), residual subjectivity and incomplete reporting in primary studies may remain. Fourth, public sequence databases are subject to uneven sampling across regions and years, which can influence apparent mutation frequencies. Finally, homology modeling provides structure-informed, hypothesis-generating context but does not replace experimental validation and may not capture glycosylation dynamics or host-specific factors.

## 5 Conclusion and future perspectives

### 5.1 Conclusion

This study provides an evidence-mapping synthesis of global research dynamics and hemagglutinin (HA) evolution of human influenza A(H1N1) from 2000 to 2025. Over the past quarter-century, H1N1 has transitioned from a pandemic threat in 2009 to an entrenched seasonal pathogen, with continued genetic drift and a complex multi-pathogen context. Our bibliometric analyses, supported by expert curation and structure-informed interpretation, indicate a marked post-2010 shift in the mutational landscape, including recurrent substitutions at HA sites S13, S146, S160, and S202. We further highlight a conformationally flexible segment spanning aa190–aa226 with potential relevance to receptor-binding microenvironments and host specificity.

Our evidence mapping also highlights the clinical significance of co-detections alongside H1N1. Across 810 literature-derived co-detection records, RSV (19.26%) and SARS-CoV-2 (12.35%) were among the most frequently reported co-pathogens, emphasizing the importance of tiered, method-aware respiratory pathogen surveillance. Using the country–year evidence map (Supplementary File S6; *N* = 428 stratifiable records), SARS-CoV-2–related co-detections were concentrated in 2020–2022 and remained notable in 2023–2025, whereas RSV was consistently reported across eras, supporting a tiered, season- and context-aware respiratory pathogen panel. Finally, by integrating large-scale literature signals with sequence/structure-informed interpretation, this work supports an Integrated Surveillance and Control Framework intended to guide preparedness and mitigation strategies, particularly in high-density settings such as China. These percentages describe the reporting composition of heterogeneous publications and should not be interpreted as pooled prevalence estimates or population-level co-infection burdens. Translation of these signals into clinical protocols requires calibration against denominator-based sentinel surveillance and standardized testing strategies.

### 5.2 Future perspectives

Looking toward 2025 and beyond, the evolution of H1N1 will be increasingly shaped by the interplay between viral mutation and residual population immunity:

### 5.3 Precision structural surveillance

Future monitoring should prioritize real-time modeling of the 190-helix and 220-loop domains to predict antigenic drift before it compromises vaccine efficacy.

### 5.4 China-specific targeted monitoring

Given the unique zoonotic-human interface in China, specialized surveillance for the G219A substitution in Eurasian avian-like (EA) strains is essential for early warning of variants with enhanced human-type receptor affinity.

### 5.5 Syndromic diagnostic integration

Clinical management must transition toward multiplex diagnostic panels that simultaneously screen for H1N1, H3N2, and secondary bacterial pathogens like *Streptococcus pneumoniae* to address the complexities of multi-pathogen co-circulation.

### 5.6 Post-2022 rebound preparedness (hypothesis-aware)

Public health strategies should prepare for the possibility of atypical seasonal peaks after 2022. Although ‘immune gap/immunity debt’ has been proposed as one explanation, it remains debated and cannot be inferred from bibliometric or literature-derived co-detection records alone. Planning should therefore be anchored in serology- and sentinel surveillance-informed indicators, while also accounting for NPI relaxation, healthcare-seeking changes, testing intensity, and viral interference.

### 5.7 Refinement of AI methodologies

While text-mining offers unprecedented breadth, future studies may integrate structure-based analyses (e.g., docking or molecular dynamics) alongside prespecified expert curation, with clear separation between hypothesis generation and experimental/epidemiological validation.

## Data Availability

The datasets presented in this study can be found in online repositories. The names of the repository/repositories and accession number(s) can be found in the article/Supplementary material.

## Supplementary material

Table 1 = Supplementary Figure S1

Table 2=Supplementary Table S2

Table 3=Supplementary File S3

Table 4=Supplementary Codebook S4

Table 5= Supplementary File S5 (Supplementary Figures S2–S4)

Table 6= Supplementary File S6

Table 7= Supplementary File S7

Table 8= Supplementary File S8

Table 9= Supplementary File S9

Table 10=Supplementary File S10

Table 11= Supplementary File S11 (Supplementary Figures S5–S8)

Table 12=Supplementary File S12
