# Supplementary material for: Evolutionary trajectory and co-infection dynamics of human influenza A(H1N1) virus (2000–2025): an integrated framework informed by expert-informed bibliometrics
Source: Front Microbiol. 2026 Mar 26;17:1793244. doi: 10.3389/fmicb.2026.1793244 (PMC13064542; doi:10.3389/fmicb.2026.1793244)
Supplement: Supplementary file 1 — Table 1 = Supplementary Figure S1 [file Table_1.doc]

**
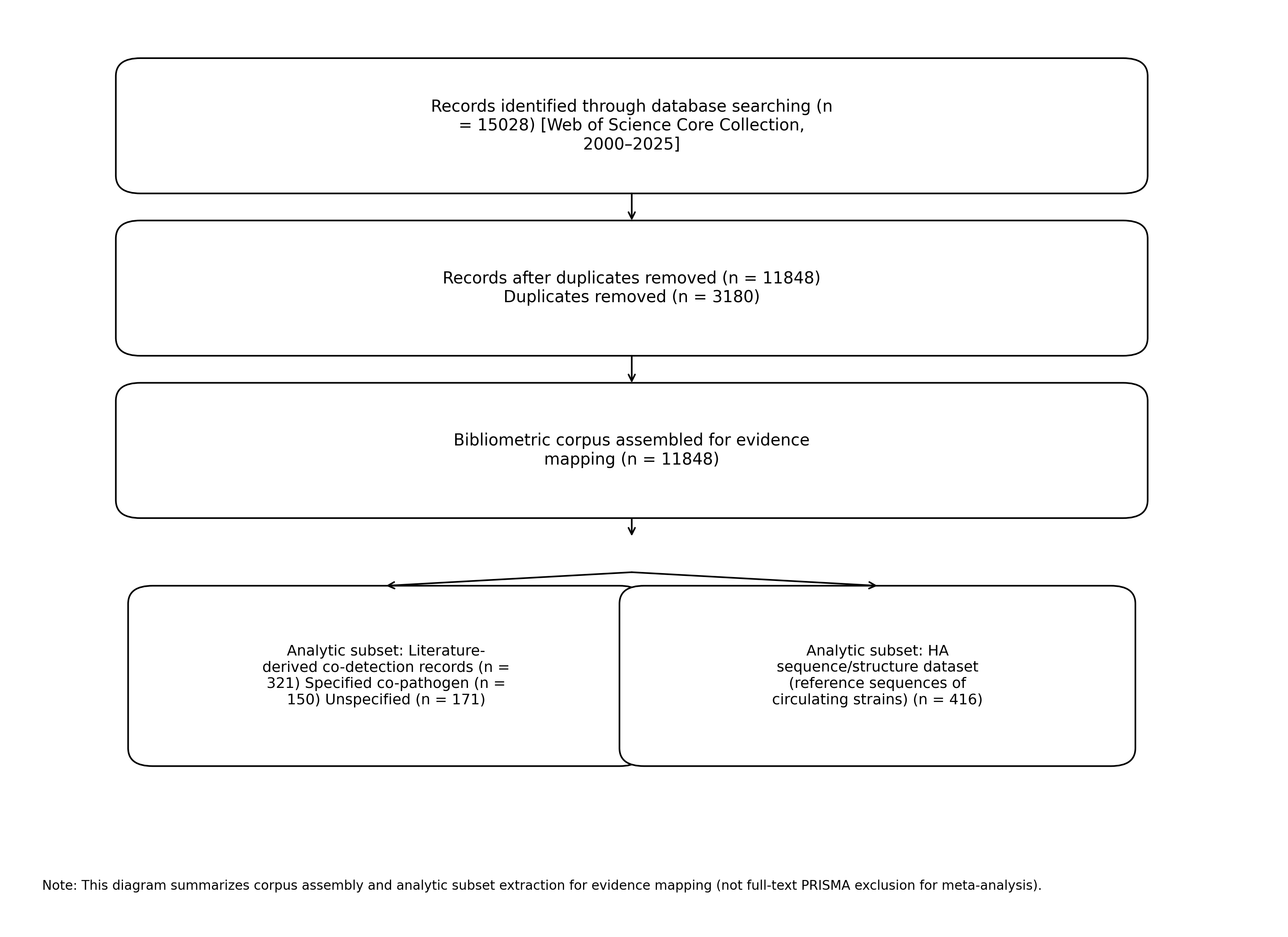
**

**Supplementary Figure S1.** Corpus assembly and analytic subset extraction workflow. Records were identified through database searching in the Web of Science Core Collection (2000–2025; n = 15,028). After deduplication, 11,848 unique records were retained as the bibliometric corpus for evidence mapping. From this corpus, two analytic subsets were derived: (i) literature-derived H1N1 co-detection records (n = 321; specified co-pathogen n = 150; unspecified co-pathogen n = 171) and (ii) the HA sequence/structure dataset of reference sequences representing circulating strains (n = 416). This diagram summarizes corpus construction and subset extraction for evidence mapping and does not represent a full-text PRISMA exclusion workflow for meta-analysis.

This workflow depicts how the bibliometric evidence-mapping corpus was assembled and how analytic subsets were extracted for downstream analyses. All records retrieved from the Web of Science Core Collection were deduplicated to form a unified bibliometric corpus used for trend analyses and mapping of reporting composition. The co-detection subset represents literature-derived co-detection records extracted from eligible publications and is summarized by whether a non-influenza co-pathogen was explicitly specified. The HA sequence/structure subset represents curated reference sequences of circulating strains used for alignment, mutation annotation, and structure-informed interpretation. Because the objective of this study is evidence mapping rather than effect-size synthesis, the workflow is presented as a corpus construction diagram and should not be interpreted as a PRISMA meta-analysis flow with full-text exclusion counts.
