# Supplementary material for: Evolutionary trajectory and co-infection dynamics of human influenza A(H1N1) virus (2000–2025): an integrated framework informed by expert-informed bibliometrics
Source: Front Microbiol. 2026 Mar 26;17:1793244. doi: 10.3389/fmicb.2026.1793244 (PMC13064542; doi:10.3389/fmicb.2026.1793244)
Supplement: Supplementary file 2 — Table 2=Supplementary Table S2 [file Table_2.docx]

**Supplementary Table S2. Eligibility criteria for literature screening and inclusion**

This table documents prespecified inclusion/exclusion criteria applied during title/abstract screening and full-text assessment to improve transparency and reproducibility.

| Domain | Inclusion criteria | Exclusion criteria | Notes / examples |
| --- | --- | --- | --- |
| Scope: topic | Human influenza A(H1N1); studies addressing viral evolution, surveillance/epidemiology, diagnostics, co-detection/co-infection, clinical outcomes, or public health response. | Non-influenza topics; influenza subtypes without extractable H1N1 results; veterinary-only influenza without human relevance. | If multiple subtypes are studied, include only when H1N1-specific data are reported/extractable. |
| Population / host | Human subjects or human-origin isolates/sequences (clinical, surveillance, or database records annotated as human). | Animal-only studies without explicit linkage to human H1N1 isolates; environmental-only sampling without human linkage. | Animal models may be included if evaluating human H1N1 mutations/isolate properties relevant to human strains. |
| Timeframe | Publications from 2000 to 2025 (inclusive). | Outside the prespecified publication window. | Aligned with the 25-year evidence-map objective. |
| Publication type | Peer-reviewed original research, surveillance reports, clinical cohort/case series with extractable data, sequence analyses, modeling studies with explicit H1N1 inputs. | Editorials/commentary/news; conference abstracts without full paper; duplicate records; retracted articles. | Reviews may be used for citation mining but are not counted as primary evidence unless explicitly specified. |
| Language | English-language publications (or languages supported by the extraction workflow, if applicable). | Non-English publications when translation/extraction is not feasible within protocol. | Revise if your protocol included additional languages. |
| Co-detection extractability | Reports mentioning ≥1 additional pathogen detected/reported alongside H1N1 within the same study context, with extractable pathogen identity (and specimen context when available). | Co-circulation mentions without co-detection reporting; non-specific co-infection without pathogen identity; no extractable pathogen information. | Primary term used is 'co-detection record' unless same-specimen co-infection with denominator is explicitly confirmed. |
| HA evolution/structure extractability | Studies reporting HA mutations/substitutions, antigenic/structural interpretation, or identifiers enabling linkage to public sequences for HA analysis. | No HA mutation/sequence information relevant to the study objectives. | Include if residue/substitution is explicitly specified even if full sequence is not reproduced. |
| Metadata sufficiency | Sufficient metadata for classification (year; and at least one of country/region, host, or study type). | Insufficient metadata to categorize and cannot be resolved by full text. | Borderline cases resolved by consensus adjudication. |

Screening workflow note: Title/abstract screening was performed independently by two reviewers; full texts were assessed when eligibility could not be determined from abstracts alone. Disagreements were resolved by consensus, with third-reviewer adjudication when needed.
