# Supplementary material for: Evolutionary trajectory and co-infection dynamics of human influenza A(H1N1) virus (2000–2025): an integrated framework informed by expert-informed bibliometrics
Source: Front Microbiol. 2026 Mar 26;17:1793244. doi: 10.3389/fmicb.2026.1793244 (PMC13064542; doi:10.3389/fmicb.2026.1793244)
Supplement: Supplementary file 4 — Table 4=Supplementary Codebook S4 [file Table_4.docx]

**Supplementary Codebook S4. Expert Curation Codebook and Decision Rules (Reproducible Protocol) Purpose**

**Purpose.** This codebook defines reproducible criteria for expert annotation of literature-derived co-detection records. The protocol aims to distinguish administrative/policy-driven reporting patterns from biological/epidemiological signals and to document assay-related surveillance bias.

**1. Inputs reviewed per record. Reviewers examined:** (i) title and abstract; (ii) extracted pathogen strings; (iii) year and country/setting metadata; and (iv) assay-related keywords when present. Full text was consulted only to resolve ambiguity.

2. Outputs (annotation fields). Each record is tagged with:
(1) Co-detection validity: {Valid co-detection / Likely false co-occurrence / Unclear}
(2) Evidence level: {Clinical denominator present / Isolate-level sequence evidence present / Neither present / Unclear}
(3) Assay strategy (if stated): {Multiplex/syndromic panel / Singleplex PCR / Culture / Serology / mNGS / Unspecified}
(4) Context classification: {Administrative/policy-driven reporting trend / Biological-epidemiological signal / Method-development dominant / Unclear}
(5) Variant-of-interest tag (optional): {Yes/No}, permitted only when isolate-level evidence is explicitly described.

**3. Decision rules.**Rule A — Valid co-detection vs false co-occurrence. “Valid co-detection” requires explicit indication of co-testing/co-detection/coinfection within the same cohort or sampling frame (e.g., “coinfection”, “co-detected”, “multiplex panel detected”). “Likely false co-occurrence” is assigned when pathogens are mentioned only as background or differential diagnosis without evidence of joint testing.

Rule B — Administrative/policy-driven reporting trend. A record is classified as administrative/policy-driven when (i) administrative/policy language is prominent AND (ii) isolate-level evidence and clinical denominators are absent or not described. Policy signals include (non-exhaustive): “emergency response”, “national plan/program”, “guideline”, “policy”, “Class B infectious disease”, “sentinel network establishment”, “capacity building”, “funding initiative”, “preparedness”, “stockpiling”, “government-led”.

Rule C — Biological/epidemiological signal. A record is classified as biological/epidemiological when it contains isolate-level and/or cohort-level evidence supporting transmission/fitness/clinical impact, including at least one of: (i) isolate-based sequencing/phylogenetics or mutation reporting linked to specific strains; (ii) functional evidence (e.g., receptor binding, replication kinetics, pathogenicity, animal model); (iii) explicit clinical denominators with co-detection results in a defined population.

Rule D — Assay strategy tagging. Assay strategy is tagged only when explicitly stated (e.g., “multiplex PCR panel”, “syndromic panel”, “FilmArray”, “Luminex”, “RT-qPCR”, “metagenomic sequencing”). Otherwise, it is tagged as “Unspecified”.

Rule E — Variant-of-interest tagging. A variant may be tagged as “variant-of-interest” only when the record explicitly links it to isolate-level evidence (e.g., isolates from a defined region/lineage, accession-based sequences) and provides functional or epidemiological relevance; narrative mention alone is insufficient.

**4. Adjudication and traceability.** Two reviewers annotate records independently using the above schema. Disagreements are resolved by consensus; if unresolved, a third reviewer adjudicates. All edits are recorded in a structured change log (record ID, original extraction, curated tag(s), and rationale code referencing Rules A–E).

**5. Interpretation constraint.** Context tags are used for bias-aware interpretation (e.g., policy-driven spikes; expansion of multiplex testing) and do not alter raw bibliometric counts or constitute population-level prevalence estimates.
