# Supplementary material for: Evolutionary trajectory and co-infection dynamics of human influenza A(H1N1) virus (2000–2025): an integrated framework informed by expert-informed bibliometrics
Source: Front Microbiol. 2026 Mar 26;17:1793244. doi: 10.3389/fmicb.2026.1793244 (PMC13064542; doi:10.3389/fmicb.2026.1793244)
Supplement: Supplementary file 5 — Table 5= Supplementary File S5 (Supplementary Figures S2–S4) [file Table_5.docx]

We evaluated the long-term trends in H1N1 influenza virus research output from 2000 to 2025 in the top 11 publishing countries, comprising a total of 286 records, using generalized linear models (GLMs) to identify key change points and driving factors. The data exhibited marked overdispersion (dispersion parameter = 127.29), with variance substantially exceeding the mean. Consequently, a negative binomial regression model was selected as the best-fitting model (AIC = 325.92) to appropriately account for overdispersion. The results indicate that the United States, China, and Japan were the predominant contributors, representing a major proportion of global research output. Research activity surged during the 2009 H1N1 pandemic, peaking between 2010 and 2012, and subsequently declined. Across countries, the mean annual number of publications was 15.86, with a median of 4.00, indicating a right-skewed distribution with several high-value outliers. The data exhibited strong positive skewness (6.66), high variability (standard deviation = 44.93), and pronounced kurtosis (49.59), reflecting a peaked distribution with extreme values. The overall dispersion parameter (127.29) confirms severe overdispersion (Figure S2). These findings suggest that most countries contributed relatively few publications (clustered in the lower range), while a small number of research-intensive countries produced a disproportionately large output, generating a "long-tail" effect. Publication counts were generally below the mean for most countries, with only a few exceeding the mean substantially. Overall, there were significant disparities in research output among countries, with a minority of nations dominating the field while the majority demonstrated lower participation.


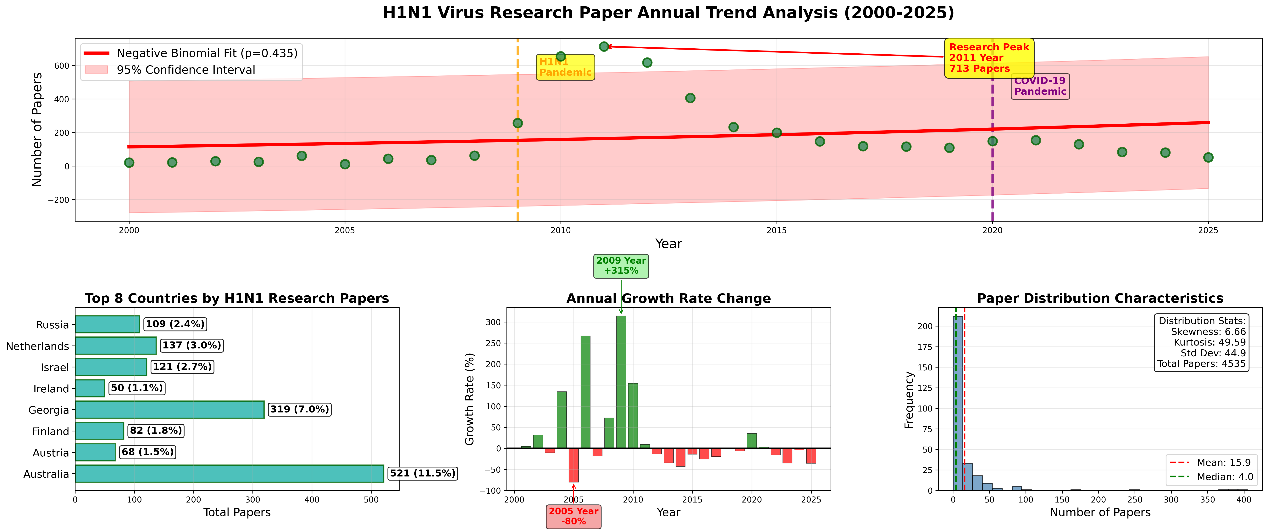


**Figure S2.** Comprehensive Analysis of H1N1 Virus Research Papers - Including Time Trends, Country Distribution, Growth Rate Changes, and Statistical Diagnostics

Based on the annual negative binomial regression analysis, the yearly coefficient across all data was 0.0327 (p = 0.435). Although this trend was not statistically significant, it indicated a slight upward trajectory. The annual publication growth rate was approximately 3.33%, suggesting a gradual increase in research activity over time. Publication counts exhibited a clear cyclical pattern, with research activity gradually returning to baseline levels following the 2010 H1N1 pandemic. Notably, the period from 2009 to 2012 showed an exceptionally high level of research activity, reflecting a strong correlation with the dynamics of the H1N1 pandemic (Figure S3, S4).


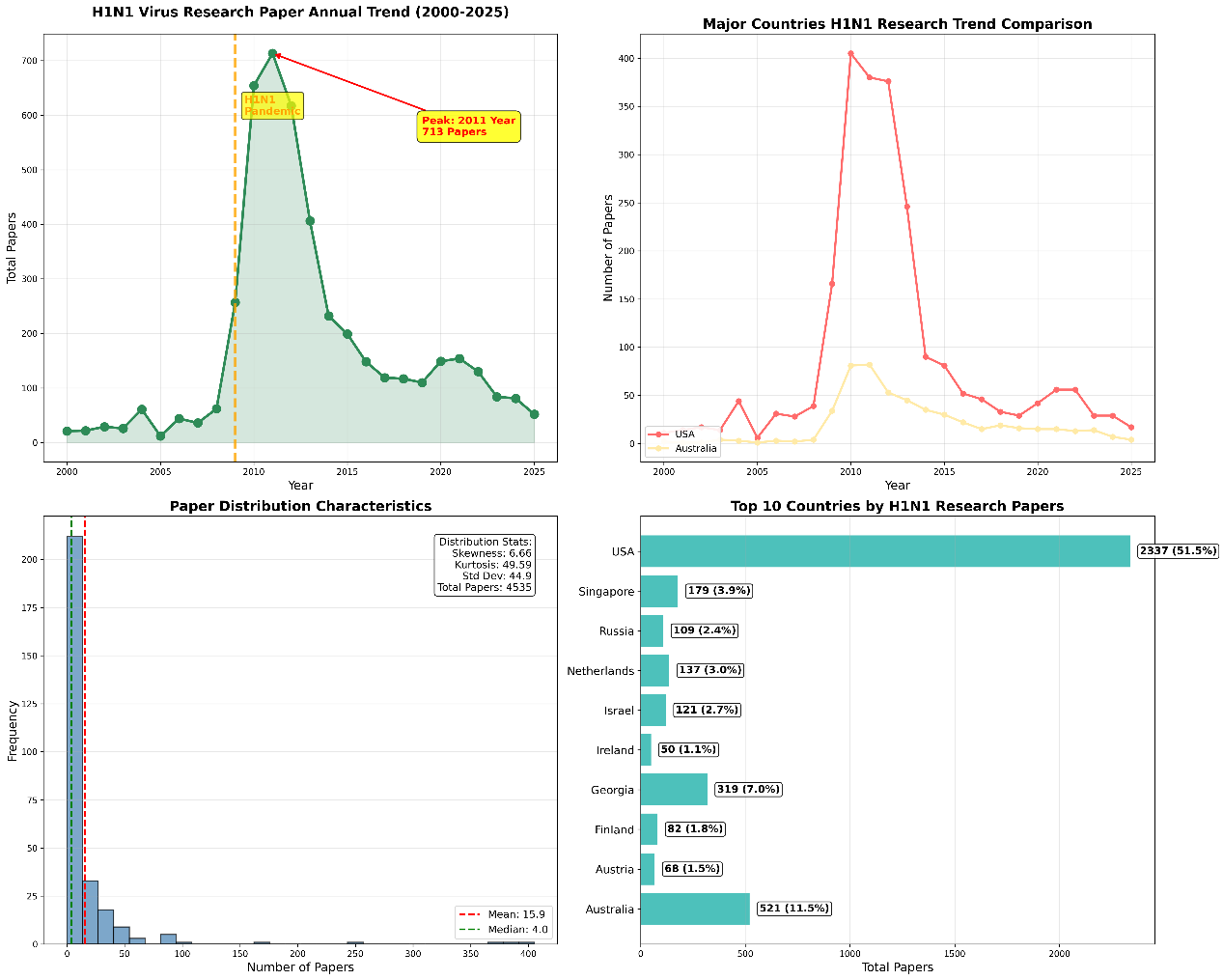


**Figure S3.** Exploratory Data Analysis - Data Distribution, Time Trends, and Statistical Characteristics


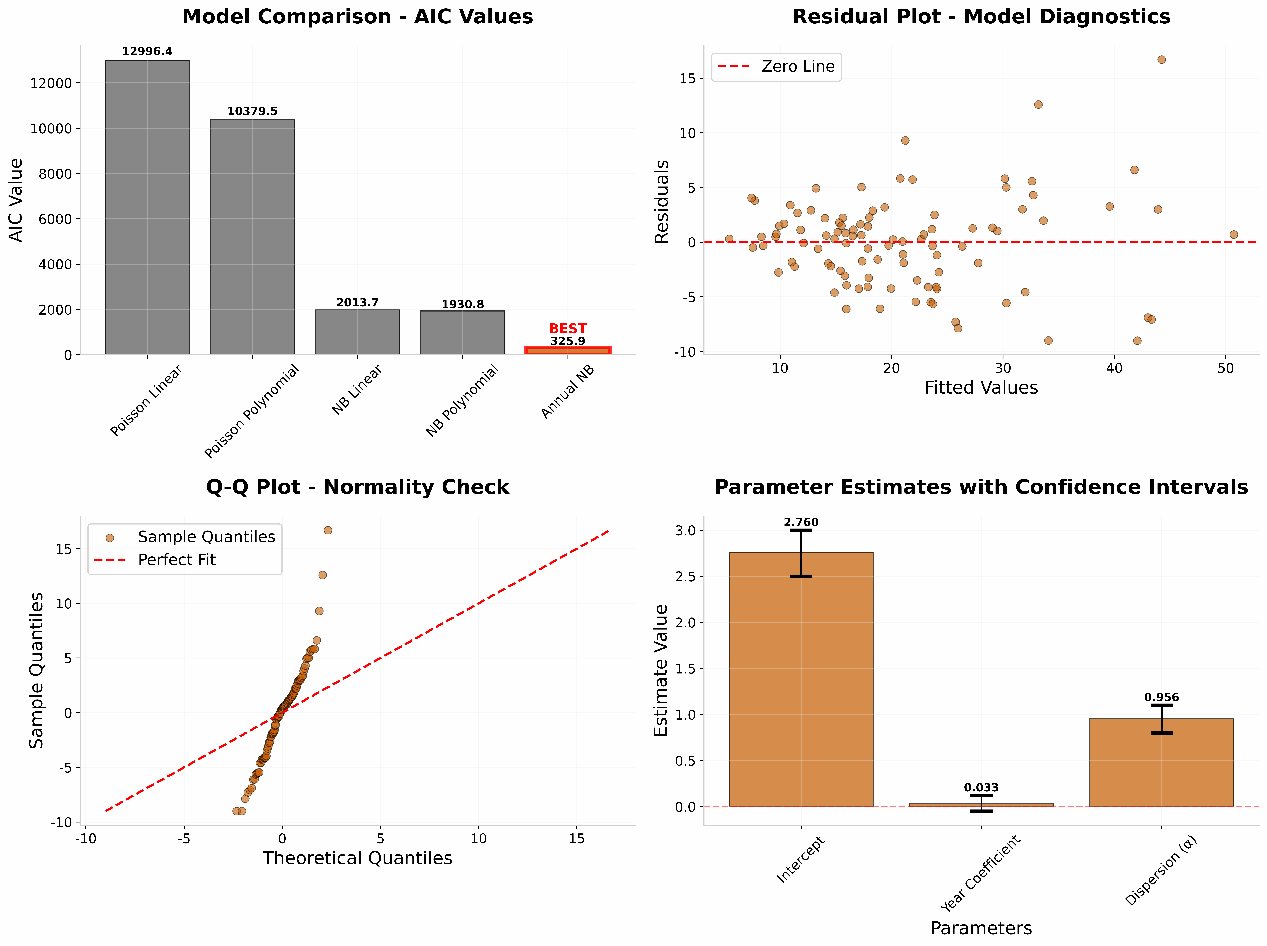


**Figure S4.** Generalized Linear Model Analysis - Model Fitting and Residual Diagnostics

These findings indicate that research activity on H1N1 influenza virus is strongly influenced by major public health events, particularly the 2009 H1N1 pandemic. The scientific community responded rapidly to the outbreak, with research output peaking in 2010–2011. Following the resolution of the pandemic, H1N1-related research maintained a stable baseline level, reflecting the continued importance of the field and demonstrating the capacity of scientific research to respond swiftly to public health crises. However, research efforts were predominantly concentrated in developed countries, highlighting the uneven distribution of global scientific resources.
