# Supplementary material for: Evolutionary trajectory and co-infection dynamics of human influenza A(H1N1) virus (2000–2025): an integrated framework informed by expert-informed bibliometrics
Source: Front Microbiol. 2026 Mar 26;17:1793244. doi: 10.3389/fmicb.2026.1793244 (PMC13064542; doi:10.3389/fmicb.2026.1793244)
Supplement: Supplementary file 7 — Table 7= Supplementary File S7 [file Table_7.doc]

**The reference genome sequences of all local H1N1 strains used in this study from 2000 to 2025.**

>LC660659.1Influenza A virus H1N101701

ATGAAGGCAATACTAGTAGTTCTGCTATATACATTTGCAACCGCAAATGCAGACACATTATGTATAGGTTATCATGCGAACAATTCAACAGACACTGTAGACACAGTACTAGAAAAGAATGTAACAGTAACACACTCTGTTAACATTCTAGAAGACAAGCATAACGGGAAACTATGCAAACTAAGAGGGGTAGCCCCATTGCATTTGGGTAAATGTAACATTGCTGGCTGGATCCTGGGAAATCCAGAGTGTGAATCACTCTCCACAGCAAGCTCATGGTCCTACATTGTGGAAACATCTAGTTCAGACAATGGAACGTGTTACCCAGGAGATTTCATCGATTATGAGGAGCTAAGAGAGCAATTGAGCTCAGTGTCATCATTTGAAAGGTTTGAGATATTCCCCAAGACAAGTTCATGGCCCAATCATGACTCGAACAAAGGTGTAACGGCAGCATGTCCTCATGCTGGAGCAAAAAGCTTCTACAAAAATTTAATATGGCTAGTTAAAAAAGAAAATTCATACCCAAAGCTCAGCAAATCCTACATTAATGATAAAGGGAAAGAAGTCCTCGTGCTATGGGGCATTCACCATCCATCTACTAGTGCTGACCAACAAAGTCTCTATCAGAATGCAGATGCATATGTTTTTGTGGGGACATCAAGATACAGCAAGAAGTTCAAGCCGGAAATAGCAATAAGACCCAAAGTGAGGGATCAAGAAGGGAGAATGAACTATTACTGGACACTAGTAGAGCCGGGAGACAAAATAACATTCGAAGCAACTGGAAATCTAGTGGTACCGAGATATGCATTCGCAATGGAAAGAAATGCTGGATCTGGTATTATCATTTCAGATACACCAGTCCACGATTGCAATACAACTTGTCAGACACCCAAGGGTGCTATAAACACCAGCCTCCCATTTCAGAATATACATCCGATCACAATTGGAAAATGTCCAAAATATGTAAAAAGCACAAAATTGAGACTGGCCACAGGATTGAGGAATGTCCCGTCTATTCAATCTAGAGGCCTATTTGGGGCCATTGCCGGTTTCATTGAAGGGGGGTGGACAGGGATGGTTGATGGATGGTACGGTTATCACCATCAAAATGAGCAGGGGTCAGGATATGCAGCCGACCTGAAGAGCACACAGAATGCCATTGACGAGATTACTAACAAAGTAAATTCTGTTATTGAAAAGATGAATACACAGTTCACAGCAGTAGGTAAAGAGTTCAACCACCTGGAAAAAAGAATAGAGAATTTAAATGAAAAGGTTGATGATGGTTTCCTGGACATTTGGACTTACAATGCAGAACTGTTGGTTCTATTGGAAAATGAAAGAACTTTGGACTACCACGATTCAAATGTGAAGAACTTATATGAAAAGGTAAGAAGCCAGTTAAAAAACAATGCCAAGGAAATTGGAAACGGCTGCTTTGAATTTTACCACAAATGCGATAACACGTGCATGGAAAGTGTCAAAAATGGGACTTATGACTACCCAAAATACTCAGAGGAAGCAAAATTAAACAGAGAAGAAATAGATGGGGTAAAGCTGGAATCAACAAGGATTTACCAGATTTTGGCGATCTATTCAACTGTCGCCAGTTCATTGGTACTGGTAGTCTCCCTGGGGGCAATCAGTTTCTGGATGTGCTCTAATGGGTCTCTACAGTGTAGAATATGTATTTAA

>LC638240.1Influenza A virus H1N101701

ATGAAGGCAATACTAGTAGTTCTGCTATATACATTTGCAACCGCAAATGCAGACACATTATGTATAGGTTATCATGCGAACAATTCAACAGACACTGTAGACACAGTACTAGAAAAGAATGTAACAGTAACACACTCTGTTAACCTTCTAGAAGACAAACATAACGGGAAACTATGCAAACTAAGAGGGGTAGCCCCATTGCATTTGGGTAAATGTAACATTGCTGGCTGGATCCTGGGAAATCCAGAGTGTGAATCACTCTCCACAGCAAGCTCATGGTCCTACATTGTGGAAACATCTAGTTCAGACAATGGAACGTGTTACCCAGGAGATTTCATCGATTATGAGGAGCTAAGAGAGCAATTGAGCTCAGTGTCATCATTTGAAAGGTTTGAGATATTCCCCAAGACAAGTTCATGGCCCAATCATGACTCGAACAAAGGTGTAACGGCAGCATGTCCTCATGCTGGAGCAAAAAGCTTCTACAAAAATTTAATATGGCTAGTTAAAAAAGGAAATTCATACCCAAAGCTCAGCAAATCCTACATTAATGATAAAGGGAAAGAAGTCCTCGTGCTATGGGGCATTCACCATCCATCTACTAGTGCTGACCAACAAAGTCTCTATCAGAATGCAGATGCATATGTTTTTGTGGGGACATCAAGATACAGCAAGAAGTTCAAGCCGGAAATAGCAATAAGACCCAAAGTGAGGGATCAAGAAGGGAGAATGAACTATTACTGGACACTAGTAGAGCCGGGAGACAAAATAACATTCGAAGCAACTGGAAATCTAGTGGTACCGAGATACGCATTCGCAATGGAAAGAAATGCTGGATCTGGTATTATCATTTCAGATACACCAGTCCATGATTGCAATACAACTTGTCAGACACCCAAGGGTGCTATAAACACCAGCCTCCCATTTCAGAATATACATCCGATCACAATTGGAAAATGTCCAAAATATGTAAAAAGCACAAAATTGAGACTGGCCACAGGATTGAGGAATGTCCCGTCTATTCAATCTAGAGGCCTATTTGGGGCCATTGCCGGCTTCATTGAAGGGGGGTGGACAGGGATGGTAGATGGATGGTACGGTTATCACCATCAAAATGAGCAGGGGTCAGGATATGCAGCCGACCTGAAGAGCACACAGAATGCCATTGACAAGATTACTAACAAAGTAAATTCTGTTATTGAAAAGATGAATACACAGTTCACAGCAGTAGGTAAAGAGTTCAACCACCTGGAAAAAAGAATAGAGAATTTAAATAAAAAAGTTGATGATGGTTTCCTGGACATTTGGACTTACAATGCCGAACTGTTGGTTCTATTGGAAAATGAAAGAACTTTGGACTACCACGATTCAAATGTGAAGAACTTATATGAAAAGGTAAGAAGCCAGTTAAAAAACAATGCCAAGGAAATTGGAAACGGCTGCTTTGAATTTTACCACAAATGCGATAACACGTGCATGGAAAGTGTCAAAAATGGGACTTATGACTACCCAAAATACTCAGAGGAAGCAAAATTAAACAGAGAAGAAATAGATGGGGTAAAGCTGGAATCAACAAGGATTTACCAGATTTTGGCGATCTATTCAACTGTCGCCAGTTCATTGGTACTGGTAGTCTCCCTGGGGGCAATCAGTTTCTGGATGTGCTCTAATGGGTCTCTACAGTGTAAAATATGTATTTAA

>LC638239.1Influenza A virus H1N101701

ATGAAGGCAATACTAGTAGTTCTGCTATATACATTTGCAACCGCAAATGCAGACACATTATGTATAGGTTATCATGCGAACAATTCAACAGACACTGTAGACACAATACTAGAAAAGAATGTAACAGTAACACACTCTGTTAACCTTCTAGAAGACAAACATAACGGGAAACTATGCAAACTAAGAGGGGTAGCCCCATTGCATTTGGGTAAATGTAACATTGCTGGCTGGATCCTGGGAAATCCAGAGTGTGAATCACTCTCCACAGCAAGCTCATGGTCCTACATTGTGGAAACATCTAGTTCAGACAATGGAACGTGTTACCCAGGAGATTTCATCGATTATGAGGAGCTAAGAGAGCAATTGAGCTCAGTGTCATCATTTGAAAGGTTTGAGATATTCCCCAAGACAAGTTCATGGCCCAATCATGACTCGAACAAAGGTGTAACGGCAGCATGTCCTCATGCTGGAGCAAAAAGCTTCTACAAAAATTTAATATGGCTAGTTAAAAAAGGAAATTCATACCCAAAGCTCAGCAAATCCTACATTAATGATAAAGGGAAAGAAGTCCTCGTGCTATGGGGCATTCACCATCCATCTACTAGTGCTGACCAACAAAGTCTCTATCAGAATGCAGATGCATATGTTTTTGTGGGGACATCAAGATACAGCAAGAAGTTCAAGCCGGAAATAGCAATAAGACCCAAAGTGAGGGATCAAGAAGGGAGAATGAACTATTACTGGACACTAGTAGAGCCGGGAGACAAAATAACATTCGAAGCAACTGGAAATCTAGTGGTACCGAGATATGCATTCGCAATGGAAAGAAATGCTGGATCTGGTATTATCATTTCAGATACACCAGTCCACGATTGCAATACAACTTGTCAGACACCCAAGGGTGCTATAAACACCAGCCTCCCATTTCAGAATATACATCCGATCACAATTGGAAAATGTCCAAAATATGTAAAAAGCACAAAATTGAGACTGGCCACAGGATTGAGGAATGTCCCGTCTATTCAATCTAGAGGCCTATTTGGGGCCATTGCCGGTTTCATTGAAGGGGGGTGGACAGGGATGGTAGATGGATGGTACGGTTATCACCATCAAAATGAGCAGGGGTCAGGATATGCAGCCGACCTGAAGAGCACACAGAATGCCATTGACGAGATTACTAACAAGGTAAATTCTGTTATTGAAAAGATGAATACACAGTTCACAGCAGTAGGTAAAGAGTTCAACCACCTGGAAAAAAGAATAGAGAATTTAAATAAAAAGGTTGATGATGGTTTCCTGGACATTTGGACTTACAATGCCGAACTGTTGGTTCTATTGGAAAATGAAAGAACTTTGGACTACCACGATTCAAATGTGAAGAACTTATATGAAAAGGTAAGAAGCCAGTTAAAAAACAATGCCAAGGAAATTGGAAACGGCTGCTTTGAATTTTACCACAAATGCGATAACACGTGCATGGAAAGTGTCAAAAATGGGACTTATGACTACCCAAAATACTCAGAGGAAGCAAAATTAAACAGAGAAGAAATAGATGGGGTAAAGCTGGAATCAACAAGGATTTACCAGATTTTGGCGATCTATTCAACTGTCGCCAGTTCATTGGTACTGATAGTCTCCCTGGGGGCAATCAGTTTCTGGATGTGCTCTAATGGGTCTCTACAGTGTAGAATATGTATTTAA

>LC638238.1Influenza A virus H1N101701

ATGAAGGCAATACTAGTAGTTCTGCTATATACATTTGCAACCGCAAATGCAGACACATTATGTATAGGTTATCATGCGAACAATTCAACAGACACTGTAGACACAGTACTAGAAAAGAATGTAACAGTAACACACTCTGTTAACCTTCTAGAAGACAAGCATAACGGGAAACTATGCAAACTAAGAGGGGTAGCCCCATTGCATTTGGGTAAATGTAACATTGCTGGCTGGATCCTGGGAAATCCAGAGTGTGAATCACTCTCCACAGCAAGCTCATGGTCCTACATTGTGGAAACATCTAGTTCAGACAATGGAACGTGTTACCCAGGAGATTTCATCGATTATGAGGAGCTAAGAGAGCAATTGAGCTCAGTGTCATCATTTGAAAGGTTTGAGATATTCCCCAAGACAAGTTCATGGCCCAATCATGACTCGAACAAAGGTGTAACGGCAGCATGTCCTCATGATGGAGCAAAAAGCTTCTACAAAAATTTAATATGGCTAGTTAAAAAAGGAAATTCATACCCAAAGCTCAGCAAATCCTACATTAATGATAAAGGGAAAGAAGTCCTCGTGCTATGGGGCATTCACCATCCATCTACTAGTGCTGACCAACAAAGTCTCTATCAGAATGCAGATGCATATGTTTTTGTGGGGACATCAAGATACAGCAAGAAGTTCAAGCCGGAAATAGCAATAAGACCCAAAGTGAGGGATCAAGAAGGGAGAATGAACTATTACTGGACACTAGTAGAGCCGGGAGACAAAATAACATTCGAAGCAACTGGAAATCTAGTGGTACCGAGATATGCATTCGCAATGGAAAGAAATGCTGGATCTGGTATTATCATTTCAGATACACCAGTCCACGATTGCAATACAACTTGTCAGACACCCAAGGGTGCTATAAACACCAGCCTCCCATTTCAGAATATACATCCGATCACAATTGGAAAATGTCCAAAATATGTAAAAAGCACAAAATTGAGACTGGCCACAGGATTGAGGAATGTCCCGTCTATTCAATCTAGAGGCCTATTTGGGGCCATTGCCGGTTTCATTGAAGGGGGGTGGACAGGGATGGTAGATGGATGGTACGGTTATCACCATCAAAATGAGCAGGGGTCAGGATATGCAGCCGACCTGAAGAGCACACAGAATGCCATTGACGTGATTACTAACAAAGTAAATTCTGTTATTGAAAAGATGAATACACAGTTCACAGCAGTAGGTAAAGAGTTCAACCACCTGGAAAAAAGAATAGAGAATTTAAATAAAAAGGTTGATGATGGTTTCCTGGACATTTGGACTTACAATGCAGAACTGTTGGTTCTATTGGAAAATGAAAGAACTTTGGACTTCCACGATTCAAATGTGAAGAACTTATATGAAAAGGTAAGAAGCCAGTTAAAAAACAATGCCAAGGAAATTGGAAACGGCTGCTTTGAATTTTACCACAAATGCGATAACACGTGCATGGAAAGTGTCAAAAATGGGACTTATGACTACCCAAAATACTCAGAGGAAGCAAAATTAAACAGAGAAGAAATAGATGGGGTAAAGCTGGAATCAACAAGGATTTACCAGATTTTGGCGATCTATTCAACTGTCGCCAGTTCATTGGTACTGGTAGTCTCCCTGGGGGCAATCAGTTTCTGGATGTGCTCTAATGGGTCTCTACAGTGTAGAATATGTATTTAA

>LC638237.1Influenza A virus H1N101701

ATGAAGGCAATACTAGTAGTTCTGCTATATACATTTGCAACCGCAAATGCAGACACATTATGTATAGGTTATCATGCGAACAATTCAACAGACACTGTAGACACAGTACTAGAAAAGAATGTAACAGTAACACACTCTGTTAACCTTCTAGAAGACAAGCATAACGGGAAACTATGCAAACTAAGAGGGGTAGCCCCATTGCATTTGGGTAAATGTAACATTGCTGGCTGGATCCTGGGAAATCCAGAGTGTGAATCACTCTCCACAGCAAGCTCATGGTCCTACATTGTGGAAACATCTAGTTCAGACAATGGAACGTGTTACCCAGGAGATTTCATCGATTATGAGGAGCTAAGAGAGCAATTGAGCTCAGTGTCATCATTTGAAAGGTTTGAGATATTCCCCAAGACAAGTTCATGGCCCAATCATGACTCAAACAAAGGTGTAACGGCAGCATGTCCTCATGCTGGAGCAAAAAGCTTCTACAAAAATTTAATATGGCTAGTTAAAAAAGGAAATTCATACCCAAAGCTCAGCAAATCCTACATTAATGATAAAGGGAAAGAAGTCCTCGTGCTATGGGGCATTCACCATCCATCTACTAGTGCTGACCAACAAAGCCTCTATCAGAATGCAGATGCATATGTTTTTGTGGGGACATCAAGATACAGCAAGAAGTTCAAGCCGGAAATAGCAATAAGACCCAAAGTGAGGGATCAAGAAGGGAGAATGAACTATTACTGGACACTAGTAGAGCCGGGAGACAAAATAACATTCGAAGCAACTGGAAATCTAATGGTACCGAGATATGCATTCGCAATGGAAAGAAATGCTGGATCTGGTATTATCATTTCAGATACACCAGTCCACGATTGCAATACAACTTGTCAGACACCCAAGGGTGCTATAAACACCAGCCTCCCATTTCAGAATATACATCCGATCACAATTGGAAAATGTCCAAAATATGTAAAAAGCACAAAATTGAGACTGGCCACAGGATTGAGGAATGTCCCTTCTATTCAATCTAGAGGCCTATTTGGGGCCATTGCCGGTTTCATTGAAGGGGGGTGGACAGGGATGGTAGATGGATGGTACGGTTATCACCATCAAAATGAGCAGGGGTCAGGATATGCAGCCGACCTGAAGAGCACACAGAATGCCATTGACGAGATTACTAACAAAGTAAATTCTGTTATTGAAAAGATGAATACACAGTTCACAGCAGTAGGTAAAGAGTTCAACCACCTGGAAAAAAGAATAGAGAATTTAAATAAAAAAGTTGATGATGGTTTCCTGGACATTTGGACTTACAATGCCGAACTGTTGGTTCTATTGGAAAATGAAAGAACTTTGGACTACCACGATTCAAATGTGAAGAACTTATATGAAAAGGTAAGAAGCCAGTTAAAAAACAATGCCAAGGAAATTGGAAACGGCTGCTTTGAATTTTACCACAAATGCGATAACACGTGCATGGAAAGTGTCAAAAATGGGACTTATGACTACCCAAAATACTCAGAGGAAGCAAAATTAAACAGAGAAGAAATAGATGGGGTAAAGCTGGAATCAACAAGGATTTACCAGATCTTGGCGATCTATTCAACTGTCGCCAGTTCATTGGTACTGGTAGTCTCCCTGGGGGCAATCAGTTTCTGGATGTGCTCTAATGGGTCTCTACAGTGTAGAATATGTATTTAA

>LC638236.1Influenza A virus H1N101701

ATGAAGGCAATACTAGTAGTTCTGCTATATACATTTGCAACCGCAAATGCAGACACATTATGTATAGGTTATCATGCGAACAATTCAACAGACACTGTAGACACAATACTAGAAAAGAATGTAACAGTAACACACTCTGTTAACCTTCTAGAAGACAAACATAACGGGAAACTATGCAAACTAAGAGGGGTAGCCCCATTGCATTTGGGTAAATGTAACATTGCTGGCTGGATCCTGGGAAATCCAGAGTGTGAATCACTCTCCACAGCAAGCTCATGGTCCTACATTGTGGAAACATCTAGTTCAGACAATGGAACGTGTTACCCAGGAGATTTCATCGATTATGAGGAGCTAAGAGAGCAATTGAGCTCAGTGTCATCATTTGAAAGGTTTGAGATATTCCCCAAGACAAGTTCATGGCCCAATCATGACTCGAACAAAGGTGTAACGGCAGCATGTCCTCATGCTGGAGCAAAAAGCTTCTACAAAAATTTAATATGGCTAGTTAAAAAAGGAAATTCATACCCAAAGCTCAGCAAATCCTACATTAATGATAAAGGGAAAGAAGTCCTCGTGCTATGGGGCATTCACCATCCATCTACTAGTGCTGACCAACAAAGTCTCTATCAGAATGCAGATGCATATGTTTTTGTGGGGACATCAAGATACAGCAAGAAGTTCAAGCCGGAAATAGCAATAAGACCCAAAGTGAGGGATCAAGAAGGGAGAATGAACTATTACTGGACACTAGTAGAGCCGGGAGACAAAATAACATTCGAAGCAACTGGAAATCTAGTGGTACCGAGATATGCATTCGCAATGGAAAGAAATGCTGGATCTGGTATTATCATTTCAGATACACCAGTCCACGATTGCAATACAACTTGTCAGACACCCAAGGGTGCTATAAACACCAGCCTCCCATTTCAGAATATACATCCGATCACAATTGGAAAATGTCCAAAATATGTAAAAAGCACAAAATTGAGACTGGCCACAGGATTGAGGAATGTCCCGTCTATTCAATCTAGAGGCCTATTTGGGGCCATTGCCGGTTTCATTGAAGGGGGGTGGACAGGGATGGTAGATGGATGGTACGGTTATCACCATCAAAATGAGCAGGGGTCAGGATATGCAGCCGACCTGAAGAGCACACAGAATGCCATTGACGAGATTACTAACAAGGTAAATTCTGTTATTGAAAAGATGAATACACAGTTCACAGCAGTAGGTAAAGAGTTCAACCACCTGGAAAAAAGAATAGAGAATTTAAATAAAAAGGTTGATGATGGTTTCCTGGACATTTGGACTTACAATGCCGAACTGTTGGTTCTATTGGAAAATGAAAGAACTTTGGACTACCACGATTCAAATGTGAAGAACTTATATGAAAAGGTAAGAAGCCAGTTAAAAAACAATGCCAAGGAAATTGGAAACGGCTGCTTTGAATTTTACCACAAATGCGATAACACGTGCATGGAAAGTGTCAAAAATGGGACTTATGACTACCCAAAATACTCAGAGGAAGCAAAATTAAACAGAGAAGAAATAGATGGGGTAAAGCTGGAATCAACAAGGATTTACCAGATTTTGGCGATCTATTCAACTGTCGCCAGTTCATTGGTACTGGTAGTCTCCCTGGGGGCAATCAGTTTCTGGATGTGCTCTAATGGGTCTCTACAGTGTAGAATATGTATTTAA

>LC638235.1Influenza A virus H1N101701

ATGAAGGCAATACTAGTAGTTCTGCTATATACATTTGCAACCGCAAATGCAGACACATTATGTATAGGTTATCATGCGAACAATTCAACAGACACTGTAGACACAGTACTAGAAAAGAATGTAACAGTAACACACTCTGTTAACCTTCTAGAAGACAAGCATAACGGGAAACTATGCAAACTAAGAGGGGTAGCCCCATTGCATTTGGGTAAATGTAACATTGCTGGCTGGATCCTGGGAAATCCAGAGTGTGAATCACTCTCCACAGCAAGCTCATGGTCCTACATTGTGGAAACATCTAGTTCAGACAATGGAACGTGTTACCCAGGAGATTTCATCGATTATGAGGAGCTAAGAGAGCAATTGAGCTCAGTGTCATCATTTGAAAGGTTTGAGATATTCCCCAAGACAAGTTCATGGCCCAATCATGACTCAAACAAAGGTGTAACGGCAGCATGTCCTCATGCTGGAGCAAAAAGCTTCTACAAAAATTTAATATGGCTAGTTAAAAAAGGAAATTCATACCCAAAGCTCAGCAAATCCTACATTAATGATAAAGGGAAAGAAGTCCTCGTGCTATGGGGCATTCACCATCCATCTACTAGTGCTGACCAACAAAGCCTCTATCAGAATGCAGATGCATATGTTTTTGTGGGGACATCAAGATACAGCAAGAAGTTCAAGCCGGAAATAGCAATAAGACCCAAAGTGAGGGATCAAGAAGGGAGAATGAACTATTACTGGACACTAGTAGAGCCGGGAGACAAAATAACATTCGAAGCAACTGGAAATCTAATGGTACCGAGATATGCATTCGCAATGGAAAGAAATGCTGGATCTGGTATTATCATTTCAGATACACCAGTCCACGATTGCAATACAACTTGTCAGACACCCAAGGGTGCTATAAACACCAGCCTCCCATTTCAGAATATACATCCGATCACAATTGGAAAATGTCCAAAATATGTAAAAAGCACAAAATTGAGACTGGCCACAGGATTGAGGAATGTCCCTTCTATTCAATCTAGAGGCCTATTTGGGGCCATTGCCGGTTTCATTGAAGGGGGGTGGACAGGGATGGTAGATGGATGGTACGGTTATCACCATCAAAATGAGCAGGGGTCAGGATATGCAGCCGACCTGAAGAGCACACAGAATGCCATTGACGTGATTACTAACAAAGTAAATTCTGTTATTGAAAAGATGAATACACAGTTCACAGCAGTAGGTAAAGAGTTCAACCACCTGGAAAAAAGAATAGAGAATTTAAATAAAAAAGTTGATGATGGTTTCCTGGACATTTGGACTTACAATGCCGAACTGTTGGTTCTATTGGAAAATGAAAGAACTTTGGACTACCACGATTCAAATGTGAAGAACTTATATGAAAAGGTAAGAAGCCAGTTAAAAAACAATGCCAAGGAAATTGGAAACGGCTGCTTTGAATTTTACCACAAATGCGATAACACGTGCATGGAAAGTGTCAAAAATGGGACTTATGACTACCCAAAATACTCAGAGGAAGCAAAATTAAACAGAGAAGAAATAGATGGGGTAAAGCTGGAATCAACAAGGATTTACCAGATCTTGGCGATCTATTCAACTGTCGCCAGTTCATTGGTACTGGTAGTCTCCCTGGGGGCAATCAGTTTCTGGATGTGCTCTAATGGGTCTCTACAGTGTAGAATATGTATTTAA

>LC638234.1Influenza A virus H1N101701

ATGAAGGCAATACTAGTAGTTCTGCTATATACATTTGCAACCGCAAATGCAGACACATTATGTATAGGTTATCATGCGAACAATTCAACAGACACTGTAGACACAGTACTAGAAAAGAATGTAACAGTAACACACTCTGTTAACCTCCTAGAAGACAAGCATAACGGGAAACTATGCAAACTAAGAGGGGTAGCCCCATTGCATTTGGGTAAATGTAACATTGCTGGCTGGATCCTGGGAAATCCAGAGTGTGAATCACTCTCCACAGCAAGCTCATGGTCCTACATTGTGGAAACATCTAGTTCAGACAATGGAACGTGTTACCCAGGAGATTTCATCGATTATGAGGAGCTAAGAGAGCAATTGAGCTCAGTGTCATCATTTGAAAGGTTTGAGATATTCCCCAAGACAAGTTCATGGCCCAATCATGACTCGAACAAAGGTGTAACGGCAGCATGTCCTCATGCTGGAGCAAAAAGCTTCTACAAAAATTTAATATGGCTAGTTAAAAAAGGAAATTCATACCCAAAGCTCAGCAAATCCTACATTAATGATAAAGGGAAAGAAGTCCTCGTGCTATGGGGCATTCACCATCCATCTACTAGTGCTGACCAACAAAGTCTCTATCAGAATGCAGATACATATGTTTTTGTGGGGACATCAAGATACAGCAAGAAGTTCAAGCCGGAAATAGCAATAAGACCCAAAGTGAGGGATCAAGAAGGGAGAATGAACTATTACTGGACACTAGTAGAGCCGGGAGACAAAATAACATTCGAAGCAACTGGAAATCTAGTGGTACCGAGATATGCATTCGCAATGGAAAGAAATGCTGGATCTGGTATTATCATTTCAGATACACCAGTCCACGATTGCAATACAACTTGTCAGACACCCAAGGGTGCTATAAACACCAGCCTTCCATTTCAGAATATACATCCGATCACAATTGGAAAATGTCCAAAATATGTAAAAAGCACAAAATTGAGACTGGCCACAGGATTGAGGAATATCCCGTCTATTCAATCTAGAGGCCTATTTGGGGCTATTGCCGGTTTCATTGAAGGGGGGTGGACAGGGATGGTAGATGGATGGTACGGTTATCACCATCAAAATGAGCAGGGGTCAGGATATGCAGCCGACCTGAAGAGCACACAGAATGCCATTGACGTGATTACTAACAAAGTAAATTCTGTTATTGAAAAGATGAATACACAGTTCACAGCAGTAGGTAAAGAGTTCAACCACCTGGAAAAAAGAATAGAGAATTTAAATAAAAAAGTTGATGATGGTTTCCTGGACATTTGGACTTACAATGCCGAACTGTTGGTTCTATTGGAAAATGAAAGAACTTTGGACTACCACGATTCAAATGTGAAGAACTTATATGAAAAGGTAAGAAGCCAGTTAAAAAACAATGCCAAGGAAATTGGAAACGGCTGCTTTGAATTTTACCACAAATGCGATAACACGTGCATGGAAAGTGTCAAAAATGGGACTTATGACTACCCAAAATACTCAGAGGAAGCAAAATTAAACAGAGAAGAAATAGATGGGGTAAAGCTGGAATCAACAAGGATTTACCAGATTTTGGCGATCTATTCAACTGTCGCCAGTTCATTGGTACTGGTAGTCTCCCTGGGGGCAATCAGTTTCTGGATGTGCTCTAATGGGTCTCTACAGTGTAGAATATGTATTTAA

>LC638233.1Influenza A virus H1N101701

ATGAAGGCAATACTAGTAGTTCTGCTATATACATTTGCAACCGCAAATGCAGACACATTATGTATAGGTTATCATGCGAACAATTCAACAGACACTGTAGACACAATACTAGAAAAGAATGTAACAGTAACACACTCTGTTAACCTTCTAGAAGACAAGCATAACGGGAAACTATGCAAACTAAGAGGGGTAGCCCCATTGCATTTGGGTAAATGTAACATTGCTGGCTGGATCCTGGGAAATCCAGAGTGTGAATCACTCTCCACAGCAAGCTCATGGTCCTACATTGTGGAAACATCTAGTTCAGACAATGGAACGTGTTACCCAGGAGATTTCATCGATTATGAGGAGCTAAGAGAGCAATTGAGCTCAGTGTCATCATTTGAAAGGTTTGAGATATTCCCCAAGACAAGTTCATGGCCCAATCATGACTCGAACAAAGGTGTAACGGCAGCATGTCCTCATGCTGGAGCAAAAAGCTTCTACAAAAATTTAATATGGCTAGTTAAAAAAGGAAATTCATACCCAAAGCTCAGCAAATCCTACATTAATGATAAAGGGAAAGAAGTCCTCGTGCTATGGGGCATTCACCATCCATCTACTAGTGCTGACCAACAAAGTCTCTATCAGAATGCAGATGCATATGTTTTTGTGGGGACATCAAGATACAGCAAGAAGTTCAAGCCGGAAATAGCAATAAGACCCAAAGTGAGGGATCAAGAAGGGAGAATGAACTATTACTGGACACTAGTAGAGCCGGGAGACAAAATAACATTCGAAGCAACTGGAAATCTAGTGGTACCGAGATATGCATTCGCAATGGAAAGAAATGCTGGATCTGGTATTATCATTTCAGATACACCAGTCCACGATTGCAATACAACTTGTCAGACACCCAAGGGTGCTATAAACACCAGCCTCCCATTTCAGAATATACATCCGATCACAATTGGAAAATGTCCAAAATATGTAAAAAGCACAAAATTGAGACTGGCCACAGGATTGAGGAATGTCCCGTCTATTCAATCTAGAGGCCTATTTGGGGCCATTGCCGGTTTCATTGAAGGGGGGTGGACAGGGATGGTAGATGGATGGTACGGTTATCACCATCAAAATGAGCAGGGGTCAGGATATGCAGCCGACCTGAAGAGCACACAGAATGCCATTGACGAGATTACTAACAAGGTAAATTCTGTTATTGAAAAGATGAATACACAGTTCACAGCAGTAGGTAAAGAGTTCAACCACCTGGAAAAAAGAATAGAGAATTTAAATAAAAAGGTTGATGATGGTTTCCTGGACATTTGGACTTACAATGCCGAACTGTTGGTTCTATTGGAAAATGAAAGAACTTTGGACTACCACGATTCAAATGTGAAGAACTTATATGAAAAGGTAAGAAGCCAGTTAAAAAACAATGCCAAGGAAATTGGAAACGGCTGCTTTGAATTTTACCACAAATGCGATAACACGTGCATGGAAAGTGTCAAAAATGGGACTTATGACTACCCAAAATACTCAGAGGAAGCAAAATTAAACAGAGAAGAAATAGATGGGGTAAAGCTGGAATCAACAAGGATTTACCAGATTTTGGCGATCTATTCAACTGTCGCCAGTTCATTGGTACTGGTAGTCTCCCTGGGGGCAATCAGTTTCTGGATGTGCTCTAATGGGTCTCTACAGTGTAGAATATGTATTTAA

>LC638232.1Influenza A virus H1N101701

ATGAAGGCAATACTAGTAGTTCTGCTATATACATTTGCAACCGCAAATGCAGACACATTATGTATAGGTTATCATGCGAACAATTCAACAGACACTGTAGACACAATACTAGAAAAGAATGTAACAGTAACACACTCTGTTAACCTTCTAGAAGACAAGCATAACGGGAAACTATGCAAACTAAGAGGGGTAGCCCCATTGCATTTGGGTAAATGTAACATTGCTGGCTGGATCCTGGGAAATCCAGAGTGTGAATCACTCTCCACAGCAAGCTCATGGTCCTACATTGTGGAAACATCTAGTTCAGACAATGGAACGTGTTACCCAGGAGATTTCATCGATTATGAGGAGCTAAGAGAGCAATTGAGCTCAGTGTCATCATTTGAAAGGTTTGAGATATTCCCCAAGACAAGTTCATGGCCCAATCATGACTCGAACAAAGGTGTAACGGCAGCATGTCCTCATGCTGGAGCAAAAAGCTTCTACAAAAATTTAATATGGCTAGTTAAAAAAGGAAATTCATACCCAAAGCTCAGCAAATCCTACATTAATGATAAAGGGAAAGAAGTCCTCGTGCTATGGGGCATTCACCATCCATCTACTAGTGCTGACCAACAAAGTCTCTATCAGAATGCAGATGCATATGTTTTTGTGGGGACATCAAGATACAGCAAGAAGTTCAAGCCGGAAATAGCAATAAGACCCAAAGTGAGGGATCAAGAAGGGAGAATGAACTATTACTGGACACTAGTAGAGCCGGGAGACAAAATAACATTCGAAGCAACTGGAAATCTAGTGGTACCGAGATATGCATTCGCAATGGAAAGAAATGCTGGATCTGGTATTATCATTTCAGATACACCAGTCCACGATTGCAATACAACTTGTCAGACACCCAAGGGTGCTATAAACACCAGCCTCCCATTTCAGAATATACATCCGATCACAATTGGAAAATGTCCAAAATATGTAAAAAGCACAAAATTGAGACTGGCCACAGGATTGAGGAATGTCCCGTCTATTCAATCTAGAGGCCTATTTGGGGCCATTGCCGGTTTCATTGAAGGGGGGTGGACAGGGATGGTAGATGGATGGTACGGTTATCACCATCAAAATGAGCAGGGGTCAGGATATGCAGCCGACCTGAAGAGCACACAGAATGCCATTGACGAGATTACTAACAAGGTAAATTCTGTTATTGAAAAGATGAATACACAGTTCACAGCAGTAGGTAAAGAGTTCAACCACCTGGAAAAAAGAATAGAGAATTTAAATAAAAAGGTTGATGATGGTTTCCTGGACATTTGGACTTACAATGCCGAACTGTTGGTTCTATTGGAAAATGAAAGAACTTTGGACTACCACGATTCAAATGTGAAGAACTTATATGAAAAGGTAAGAAGCCAGTTAAAAAACAATGCCAAGGAAATTGGAAACGGCTGCTTTGAATTTTACCACAAATGCGATAACACGTGCATGGAAAGTGTCAAAAATGGGACTTATGACTACCCAAAATACTCAGAGGAAGCAAAATTAAACAGAGAAGAAATAGATGGGGTAAAGCTGGAATCAACAAGGATTTACCAGATTTTGGCGATCTATTCAACTGTCGCCAGTTCATTGGTACTGGTAGTCTCCCTGGGGGCAATCAGTTTCTGGATGTGCTCTAATGGGTCTCTACAGTGTAGAATATGTATTTAA

>LC638231.1Influenza A virus H1N101701

ATGAAGGCAATACTAGTAGTTCTGCTATATACATTTGCAACCGCAAATGCAGACACATTATGTATAGGTTATCATGCGAACAATTCAACAGACACTGTAGACACAGTACTAGAAAAGAATGTAACAGTAACACACTCTGTTAACCTTCTAGAAGACAAGCATAACGGGAAACTATGCAAACTAAGAGGGGTAGCCCCATTGCATTTGGGTAAATGTAACATTGCTGGCTGGATCCTGGGAAATCCAGAGTGTGAATCACTCTCCACAGCAAGCTCATGGTCCTACATTGTGGAAACATCTAGTTCAGACAATGGAACGTGTTACCCAGGAGATTTCATCGATTATGAGGAGCTAAGAGAGCAATTGAGCTCAGTGTCATCATTTGAAAGGTTTGAGATATTCCCCAAGACAATTTCATGGCCCAATCATGACTCGAACAAAGGTGTAACGGCAGCATGTCCTCATGCTGGAGCAAAAAGCTTCTACAAAAATTTAATATGGCTAGTTAAAAAAGGAAATTCATACCCAAAGCTCAGCAAATCCTACATTAATGATAAAGGGAAAGAAGTCCTCGTGCTATGGGGCATTCACCATCCATCTACTAGTGCTGACCAACAAAGTCTCTATCAGAATGCAGATGCATATGTTTTTGTGGGGACATCAAGATACAGCAAGAAGTTCAAGCCGGAAATAGCAATAAGACCCAAAGTGAGGGATCAAGAAGGGAGAATGAACTATTACTGGACACTAGTAGAGCCGGGAGACAAAATAACATTCGAAGCAACTGGAAATCTAGTGGTACCGAGATATGCATTCGCAATGGAAAGAAATGCTGGATCTGGTATTATCATTTCAGATACACCAGTCCACGATTGCAATACAACTTGTCAGACACCCAAGGGTGCTATAAACACCAGCCTCCCATTTCAGAATATACATCCGATCACAATTGGAAAATGTTCAAAATATGTAAAAAGCACAAAATTGAGACTGGCCACAGGATTGAGGAATGTCCCGTCTATTCAATCTAGAGGCCTATTTGGGGCTATTGCCGGTTTCATTGAAGGGGGGTGGACAGGGATGGTAGATGGATGGTACGGTTATCACCATCAAAATGAGCAGGGGTCAGGATATGCAGCCGACCTGAAGAGCACACAAAATGCCATTGACGTGATTACTAACAAAGTAAATTCTGTTATTGAAAAGATGAATACACAGTTCACAGCAGTAGGTAAAGAGTTCAACCACCTGGAAAAAAGAATAGAGAATTTAAATAAAAAGGTTGATGATGGTTTCCTGGACATTTGGACTTACAATGCCGAACTGTTGGTTCTATTGGAAAATGAAAGAACTTTGGACTACCACGATTCAAATGTGAAGAACTTATATGAAAAGGTAAGAAGCCAGTTAAAAAACAATGCCAAGGAAATTGGAAACGGCTGCTTTGAATTTTACCACAAATGCGATAACACGTGCATGGAAAGTATCAAAAATGGGACTTATGACTACCCAAAATACTCAGAGGAAGCAAAATTAAACAGAGAAGAAATAGATGGGGTAAAGCTGGAATCAACAAGGATTTACCAGATTTTGGCGATCTATTCAACTGTCGCCAGTTCATTGGTACTGGTAGTCTCCCTGGGGGCAATCAGTTTCTGGATGTGCTCTAATGGGTCTCTACAGTGTAGAATATGTATTTAA

>LC638230.1Influenza A virus H1N101701

ATGAAGGCAATACTAGTAGTTCTGCTATATACATTTGCAACCGCAAATGCAGACACATTATGTATAGGTTATCATGCGAACAATTCAACAGACACTGTAGACACAATACTAGAAAAGAATGTAACAGTAACACACTCTGTTAACCTTCTAGAAGACAAACATAACGGGAAACTATGCAAACTAAGAGGGGTAGCCCCATTGCATTTGGGTAAATGTAACATTGCTGGCTGGATCCTGGGAAATCCAGAGTGTGAATCACTCTCCACAGCAAGCTCATGGTCCTACATTGTGGAAACATCTAGTTCAGACAATGGAACGTGTTACCCAGGAGATTTCATCGATTATGAGGAGCTAAGAGAGCAATTGAGCTCAGTGTCATCATTTGAAAGGTTTGAGATATTCCCCAAGACAAGTTCATGGCCCAATCATGACTCGAACAAAGGTGTAACGGCAGCATGTCCTCATGCTGGAGCAAAAAGCTTCTACAAAAATTTAATATGGCTAGTTAAAAAAGGAAATTCATACCCAAAGCTCAGCAAATCCTACATTAATGATAAAGGGAAAGAAGTCCTCGTGCTATGGGGCATTCACCATCCATCTACTAGTGCTGACCAACAAAGTCTCTATCAGAATGCAGATGCATATGTTTTTGTGGGGACATCAAGATACAGCAAGAAGTTCAAGCCGGAAATAGCAATAAGACCCAAAGTGAGGGATCAAGAAGGGAGAATGAACTATTACTGGACACTAGTAGAGCCGGGAGACAAAATAACATTCGAAGCAACTGGAAATCTAGTGGTACCGAGATATGCATTCGCAATGGAAAGAAATGCTGGATCTGGTATTATCATTTCAGATACACCAGTCCACGATTGCAATACAACTTGTCAGACACCCAAGGGTGCTATAAACACCAGCCTCCCATTTCAGAATATACATCCGATCACAATTGGAAAATGTCCAAAATATGTAAAAAGCACAAAATTGAGACTGGCCACAGGATTGAGGAATGTCCCGTCTATTCAATCTAGAGGCCTATTTGGGGCCATTGCCGGTTTCATTGAAGGGGGGTGGACAGGGATGGTAGATGGATGGTACGGTTATCACCATCAAAATGAGCAGGGGTCAGGATATGCAGCCGACCTGAAGAGCACACAGAATGCCATTGACGTGATTACTAACAAGGTAAATTCTGTTATTGAAAAGATGAATACACAGTTCACAGCAGTAGGTAAAGAGTTCAACCACCTGGAAAAAAGAATAGAGAATTTAAATAAAAAGGTTGATGATGGTTTCCTGGACATTTGGACTTACAATGCCGAACTGTTGGTTCTATTGGAAAATGAAAGAACTTTGGACTACCACGATTCAAATGTGAAGAACTTATATGAAAAGGTAAGAAGCCAGTTAAAAAACAATGCCAAGGAAATTGGAAACGGCTGCTTTGAATTTTACCACAAATGCGATAACACGTGCATGGAAAGTGTCAAAAATGGGACTTATGACTACCCAAAATACTCAGAGGAAGCAAAATTAAACAGAGAAGAAATAGATGGGGTAAAGCTGGAATCAACAAGGATTTACCAGATTTTGGCGATCTATTCAACTGTCGCCAGTTCATTGGTACTGGTAGTCTCCCTGGGGGCAATCAGTTTCTGGATGTGCTCTAATGGGTCTCTACAGTGTAGAATATGTATTTAA

>LC638229.1Influenza A virus H1N101701

ATGAAGGCAATACTAGTAGTTCTGCTATATACATTTGCAACCGCAAATGCAGACACATTATGTATAGGTTATCATGCGAACAATTCAACAGACACTGTAGACACAGTACTAGAAAAGAATGTAACAGTAACACACTCTGTTAACCTTCTAGAAGACAAGCATAACGGGAAACTATGCAAACTAAGAGGGGTAGCCCCATTGCATTTGGGTAAATGTAACATTGCTGGCTGGATCCTGGGAAATCCAGAGTGTGAATCACTCTCCACAGCAAGCTCATGGTCCTACATTGTGGAAACATCTAGTTCAGACAATGGAACGTGTTACCCAGGAGATTTCATCGATTATGAGGAGCTAAGAGAGCAATTGAGCTCAGTGTCATCATTTGAAAGGTTTGAGATATTCCCCAAGACAATTTCATGGCCCAATCATGACTCGAACAAAGGTGTAACGGCAGCATGTCCTCATGCTGGAGCAAAAAGCTTCTACAAAAATTTAATATGGCTAGTTAAAAAAGGAAATTCATACCCAAAGCTCAGCAAATCCTACATTAATGATAAAGGGAAAGAAGTCCTCGTGCTATGGGGCATTCACCATCCATCTACTAGTGCTGACCAACAAAGTCTCTATCAGAATGCAGATGCATATGTTTTTGTGGGGACATCAAGATACAGCAAGAAGTTCAAGCCGGAAATAGCAATAAGACCCAAAGTGAGGGATCAAGAAGGGAGAATGAACTATTACTGGACACTAGTAGAGCCGGGAGACAAAATAACATTCGAAGCAACTGGAAATCTAGTGGTACCGAGATATGCATTCGCAATGGAAAGAAATGCTGGATCTGGTATTATCATTTCAGATACACCAGTCCACGATTGCAATACAACTTGTCAGACACCCAAGGGTGCTATAAACACCAGCCTCCCATTTCAGAATATACATCCGATCACAATTGGAAAATGTTCAAAATATGTAAAAAGCACAAAATTGAGACTGGCCACAGGATTGAGGAATGTCCCGTCTATTCAATCTAGAGGCCTATTTGGGGCTATTGCCGGTTTCATTGAAGGGGGGTGGACAGGGATGGTAGATGGATGGTACGGTTATCACCATCAAAATGAGCAGGGGTCAGGATATGCAGCCGACCTGAAGAGCACACAAAATGCCATTGACGTGATTACTAACAAAGTAAATTCTGTTATTGAAAAGATGAATACACAGTTCACAGCAGTAGGTAAAGAGTTCAACCACCTGGAAAAAAGAATAGAGAATTTAAATAAAAAGGTTGATGATGGTTTCCTGGACATTTGGACTTACAATGCCGAACTGTTGGTTCTATTGGAAAATGAAAGAACTTTGGACTACCACGATTCAAATGTGAAGAACTTATATGAAAAGGTAAGAAGCCAGTTAAAAAACAATGCCAAGGAAATTGGAAACGGCTGCTTTGAATTTTACCACAAATGCGATAACACGTGCATGGAAAGTATCAAAAATGGGACTTATGACTACCCAAAATACTCAGAGGAAGCAAAATTAAACAGAGAAGAAATAGATGGGGTAAAGCTGGAATCAACAAGGATTTACCAGATTTTGGCGATCTATTCAACTGTCGCCAGTTCATTGGTACTGGTAGTCTCCCTGGGGGCAATCAGTTTCTGGATGTGCTCTAATGGGTCTCTACAGTGTAGAATATGTATTTAA

>LC638228.1Influenza A virus H1N101701

ATGAAGGCAATACTAGTAGTTCTGCTATATACATTTGCAACCGCAAATGCAGACACATTATGTATAGGTTATCATGCGAACAATTCAACAGACACTGTAGACACAGTACTAGAAAAGAATGTAACAGTAACACACTCTGTTAACCTTCTAGAAGACAAGCATAACGGGAAACTATGCAAACTAAGAGGGGTAGCCCCATTGCATTTGGGTAAATGTAACATTGCTGGCTGGATCCTGGGAAATCCAGAGTGTGAATCACTCTCCACAGCAAGCTCATGGTCCTACATTGTGGAAACATCTAGTTCAGACAATGGAACGTGTTACCCAGGAGATTTCATCGATTATGAGGAGCTAAGAGAGCAATTGAGCTCAGTGTCATCATTTGAAAGGTTTGAGATATTCCCCAAGACAAGTTCATGGCCCAATCATGACTCAAACAAAGGTGTAACGGCAGCATGTCCTCATGCTGGAGCAAAAAGCTTCTACAAAAATTTAATATGGCTAGTTAAAAAAGGAAATTCATACCCAAAGCTCAGCAAATCCTACATTAATGATAAAGGGAAAGAAGTCCTCGTGCTATGGGGCATTCACCATCCATCTACTAGTGCTGACCAACAAAGCCTCTATCAGAATGCAGATGCATATGTTTTTGTGGGGACATCAAGATACAGCAAGAAGTTCAAGCCGGAAATAGTAATAAGACCCAAAGTGAGGGATCAAGAAGGGAGAATGAACTATTACTGGACACTAGTAGAGCCGGGAGACAAAATAACATTCGAAGCAACTGGAAATCTAATGGTACCGAGATATGCATTCGCAATGGAAAGAAATGCTGGATCTGGTATTATCATTTCAGATACACCAGTCCACGATTGCAATACAACTTGTCAGACACCCAAGGGTGCTATAAACACCAGCCTCCCATTTCAGAATATACATCCGATCACAATTGGAAAATGTCCAAAATATGTAAAAAGCACAAAATTGAGACTGGCCACAGGATTGAGGAATGTCCCTTCTATTCAATCTAGAGGCCTATTTGGGGCCATTGCCGGTTTCATTGAAGGGGGGTGGACAGGGATGGTAGATGGATGGTACGGTTATCACCATCAAAATGAGCAGGGGTCAGGATATGCAGCCGACCTGAAGAGCACACAGAATGCCATTGACGAGATTACTAACAAAGTAAATTCTGTTATTGAAAAGATGAATACACAGTTCACAGCAGTAGGTAAAGAGTTCAACCACCTGGAAAAAAGAATAGAGAATTTAAATAAAAAAGTTGATGATGGTTTCCTGGACATTTGGACTTACAATGCCGAACTGTTGGTTCTATTGGAAAATGAAAGAACTTTGGACTACCACGATTCAAATGTGAAGAACTTATATGAAAAGGTAAGAAGCCAGTTAAAAAACAATGCCAAGGAAATTGGAAACGGCTGCTTTGAATTTTACCACAAATGCGATAACACGTGCATGGAAAGTGTCAAAAATGGGACTTATGACTACCCAAAATACTCAGAGGAAGCAAAATTAAACAGAGAAGAAATAGATGGGGTAAAGCTGGAATCAACAAGGATTTACCAGATCTTGGCGATCTATTCAACTGTCGCCAGTTCATTGGTACTGGTAGTCTCCCTGGGGGCAATCAGTTTCTGGATGTGCTCTAATGGGTCTCTACAGTGTAGAATATGTATTTAA

>LC638227.1Influenza A virus H1N101701

ATGAAGGCAATACTAGTAGTTCTGCTATATACATTTGCAACCGCAAATGCAGACACATTATGTATAGGTTATCATGCGAACAATTCAACAGACACTGTAGACACAATACTAGAAAAGAATGTAACAGTAACACACTCTGTTAACCTTCTAGAAGACAAACATAACGGGAAACTATGCAAACTAAGAGGGGTAGCCCCATTGCATTTGGGTAAATGTAACATTGCTGGCTGGATCCTGGGAAATCCAGAGTGTGAATCACTCTCCACAGCAAGCTCATGGTCCTACATTGTGGAAACATCTAGTTCAGACAATGGAACGTGTTACCCAGGAGATTTCATCGATTATGAGGAGCTAAGAGAGCAATTGAGCTCAGTGTCATCATTTGAAAGGTTTGAGATATTCCCCAAGACAAGTTCATGGCCCAATCATGACTCGAACAAAGGTGTAACGGCAGCATGTCCTCATGCTGGAGCAAAAAGCTTCTACAAAAATTTAATATGGCTAGTTAAAAAAGGAAATTCATACCCAAAGCTCAGCAAATCCTACATTAATGATAAAGGGAAAGAAGTCCTCGTGCTATGGGGCATTCACCATCCATCTACTAGTGCTGACCAACAAAGTCTCTATCAGAATGCAGATGCATATGTTTTTGTGGGGACATCAAGATACAGCAAGAAGTTCAAGCCGGAAATAGCAATAAGACCCAAAGTGAGGGATCAAGAAGGGAGAATGAACTATTACTGGACACTAGTAGAGCCGGGAGACAAAATAACATTCGAAGCAACTGGAAATCTAGTGGTACCGAGATATGCATTCGCAATGGAAAGAAATGCTGGATCTGGTATTATCATTTCAGATACACCAGTCCACGATTGCAATACAACTTGTCAGACACCCAAGGGTGCTATAAACACCAGCCTCCCATTTCAGAATATACATCCGATCACAATTGGAAAATGTCCAAAATATGTAAAAAGCACAAAATTGAGACTGGCCACAGGATTGAGGAATGTCCCGTCTATTCAATCTAGAGGCCTATTTGGGGCCATTGCCGGTTTCATTGAAGGGGGGTGGACAGGGATGGTAGATGGATGGTACGGTTATCACCATCAAAATGAGCAGGGGTCAGGATATGCAGCCGACCTGAAGAGCACACAGAATGCCATTGACGAGATTACTAACAAGGTAAATTCTGTTATTGAAAAGATGAATACACAGTTCACAGCAGTAGGTAAAGAGTTCAACCACCTGGAAAAAAGAATAGAGAATTTAAATAAAAAGGTTGATGATGGTTTCCTGGACATTTGGACTTACAATGCCGAACTGTTGGTTCTATTGGAAAATGAAAGAACTTTGGACTACCACGATTCAAATGTGAAGAACTTATATGAAAAGGTAAGAAGCCAGTTAAAAAACAATGCCAAGGAAATTGGAAACGGCTGCTTTGAATTTTACCACAAATGCGATAACACGTGCATGGAAAGTGTCAAAAATGGGACTTATGACTACCCAAAATACTCAGAGGAAGCAAAATTAAACAGAGAAGAAATAGATGGGGTAAAGCTGGAATCAACAAGGATTTACCAGATTTTGGCGATCTATTCAACTGTCGCCAGTTCATTGGTACTGGTAGTCTCCCTGGGGGCAATCAGTTTCTGGATGTGCTCTAATGGGTCTCTACAGTGTAGAATATGTATTTAA

>LC638226.1Influenza A virus H1N101701

ATGAAGGCAATACTAGTAGTTCTGCTATATACATTTGCAACCGCAAATGCAGACACATTATGTATAGGTTATCATGCGAACAATTCAACAGACACTGTAGACACAATACTAGAAAAGAATGTAACAGTAACACACTCTGTTAACCTTCTAGAAGACAAACATAACGGGAAACTATGCAAACTAAGAGGGGTAGCCCCATTGCATTTGGGTAAATGTAACATTGCTGGCTGGATCCTGGGAAATCCAGAGTGTGAATCACTCTCCACAGCAAGCTCATGGTCCTACATTGTGGAAACATCTAGTTCAGACAATGGAACGTGTTACCCAGGAGATTTCATCGATTATGAGGAGCTAAGAGAGCAATTGAGCTCAGTGTCATCATTTGAAAGGTTTGAGATATTCCCCAAGACAAGTTCATGGCCCAATCATGACTCGAACAAAGGTGTAACGGCAGCATGTCCTCATGCTGGAGCAAAAAGCTTCTACAAAAATTTAATATGGCTAGTTAAAAAAGGAAATTCATACCCAAAGCTCAGCAAATCCTACATTAATGATAAAGGGAAAGAAGTCCTCGTGCTATGGGGCATTCACCATCCATCTACTAGTGCTGACCAACAAAGTCTCTATCAGAATGCAGATGCATATGTTTTTGTGGGGACATCAAGATACAGCAAGAAGTTCAAGCCGGAAATAGCAATAAGACCCAAAGTGAGGGATCAAGAAGGGAGAATGAACTATTACTGGACACTAGTAGAGCCGGGAGACAAAATAACATTCGAAGCAACTGGAAATCTAGTGGTACCGAGATATGCATTCGCAATGGAAAGAAATGCTGGATCTGGTATTATCATTTCAGATACACCAGTCCACGATTGCAATACAACTTGTCAGACACCCAAGGGTGCTATAAACACCAGCCTCCCATTTCAGAATATACATCCGATCACAATTGGAAAATGTCCAAAATATGTAAAAAGCACAAAATTGAGACTGGCCACAGGATTGAGGAATGTCCCGTCTATTCAATCTAGAGGCCTATTTGGGGCCATTGCCGGTTTCATTGAAGGGGGGTGGACAGGGATGGTAGATGGATGGTACGGTTATCACCATCAAAATGAGCAGGGGTCAGGATATGCAGCCGACCTGAAGAGCACACAGAATGCCATTGACGTGATTACTAACAAGGTAAATTCTGTTATTGAAAAGATGAATACACAGTTCACAGCAGTAGGTAAAGAGTTCAACCACCTGGAAAAAAGAATAGAGAATTTAAATAAAAAGGTTGATGATGGTTTCCTGGACATTTGGACTTACAATGCCGAACTGTTGGTTCTATTGGAAAATGAAAGAACTTTGGACTACCACGATTCAAATGTGAAGAACTTATATGAAAAGGTAAGAAGCCAGTTAAAAAACAATGCCAAGGAAATTGGAAACGGCTGCTTTGAATTTTACCACAAATGCGATAACACGTGCATGGAAAGTGTCAAAAATGGGACTTATGACTACCCAAAATACTCAGAGGAAGCAAAATTAAACAGAGAAGAAATAGATGGAGTAAAGCTGGAATCAACAAGGATTTACCAGATTTTGGCGATCTATTCAACTGTCGCCAGTTCATTGGTACTGGTAGTCTCCCTGGGGGCAATCAGTTTCTGGATGTGCTCTAATGGGTCTCTACAGTGTAGAATATGTATTTAA

>LC638225.1Influenza A virus H1N101701

ATGAAGGCAATACTAGTAGTTCTGCTATATACATTTGCAACCGCAAATGCAGACACATTATGTATAGGTTATCATGCGAACAATTCAACAGACACTGTAGACACAGTACTAGAAAAGAATGTAACAGTAACACACTCTGTTAACCTTCTAGAAGACAAGCATAACGGGAAACTATGCAAACTAAGAGGGGTAGCCCCATTGCATTTGGGTAAATGTAACATTGCTGGCTGGATCCTGGGAAATCCAGAGTGTGAATCACTCTCCACAGCAAGCTCATGGTCCTACATTGTGGAAACATCTAGTTCAGACAATGGAACGTGTTACCCAGGAGATTTCATCGATTATGAGGAGCTAAGAGAGCAATTGAGCTCAGTGTCATCATTTGAAAGGTTTGAGATATTCCCCAAGACAAGTTCATGGCCCAATCATGACTCGAACAAAGGTGTAACGGCAGCATGTCCTCATGCTGGAGCAAAAAGCTTCTACAAAAATTTAATATGGCTAGTTAAAAAAGGAAATTCATACCCAAAGCTCAGCAAATCCTACATTAATGATAAAGGGAAAGAAGTCCTCGTGCTATGGGGCATTCACCATCCATCTACTAGTGCTGACCAACAAAGTCTCTATCAGAATGCAGATGCATATGTTTTTGTGGGGACATCAAGATACAGCAAGAAGTTCAAGCCGGAAATAGCAATAAGACCCAAAGTGAGGGATCAAGAAGGGAGAATGAACTATTACTGGACACTAGTAGAGCCGGGAGACAAAATAACATTCGAAGCAACTGGAAATCTAGTGGTACCGAGATATGCATTCGCAATGGAAAGAAATGCTGGATCTGGTATTATCATTTCAGATACACCAGTCCACGATTGCAATACAACTTGTCAGACACCCAAGGGTGCTATAAACACCAGCCTCCCATTTCAGAATATACATCCGATCACAATTGGAAAATGTCCAAAATATGTAAAAAGCACAAAATTGAGACTGGCCACAGGATTGAGGAATGTCCCGTCTATTCAATCTAGAGGCCTATTTGGAGCCATTGCCGGTTTCATTGAAGGGGGGTGGACAGGGATGGTAGATGGATGGTACGGTTATCACCATCAAAATGAGCAGGGGTCAGGATATGCAGCCGACCTGAAGAGCACACAGAATGCCATTGACGTGATTACTAACAAAGTAAATTCTGTTATTGAAAAGATGAATACACAGTTCACAGCAGTAGGTAAAGAGTTCAACCACCTGGAAAAAAGAATAGAGAATTTAAATAAAAAGGTTGATGATGGTTTCCTGGACATTTGGACTTACAATGCAGAACTGTTGGTTCTATTGGAAAATGAAAGAACTTTGGACTACCACGATTCAAATGTGAAGAACTTATATGAAAAGGTAAGAAGCCAGTTAAAAAACAATGCCAAGGAAATTGGAAACGGCTGCTTTGAATTTTACCACAAATGCGATAACACGTGCATGGGAAGTGTCAAAAATGGGACTTATGACTACCCAAAATACTCAGAGGAAGCAAAATTAAACAGAGAAGAAATAGATGGGGTAAAGCTGGAATCAACAAGGATTTACCAGATTTTGGCGATCTATTCAACTGTCGCCAGTTCATTGGTGCTGGTAGTCTCCCTGGGGGCAATCAGTTTCTGGATGTGCTCTAATGGGTCTCTACAGTGTAGAATATGTATTTAA

>LC638224.1Influenza A virus H1N101701

ATGAAGGCAATACTAGTAGTTCTGCTATATACATTTGCAACCGCAAATGCAGACACATTATGTATAGGTTATCATGCGAACAATTCAACAGACACTGTAGACACAGTACTAGAAAAGAATGTAACAGTAACACACTCTGTTAACCTTCTAGAAGACAAGCATAACGGGAAACTATGCAAACTAAGAGGGGTAGCCCCATTGCATTTGGGTAAATGTAACATTGCTGGCTGGATCCTGGGAAATCCAGAGTGTGAATCACTCTCCACAGCAAGCTCATGGTCCTACATTGTGGAAACATCTAGTTCAGACAATGGAACGTGTTACCCAGGAGATTTCATCGATTATGAGGAGCTAAGAGAGCAATTGAGCTCAGTGTCATCATTTGAAAGGTTTGAGATATTCCCCAAGACAATTTCATGGCCCAATCATGACTCGAACAAAGGTGTAACGGCAGCATGTCCTCATGCTGGAGCAAAAAGCTTCTACAAAAATTTAATATGGCTAGTTAAAAAAGGAAATTCATACCCAAAGCTCAGCAAATCCTACATTAATGATAAAGGGAAAGAAGTCCTCGTGCTATGGGGCATTCACCATCCATCTACTAGTGCTGACCAACAAAGTCTCTATCAGAATGCAGATGCATATGTTTTTGTGGGGACATCAAGATACAGCAAGAAGTTCAAGCCGGAAATAGCAATAAGACCCAAAGTGAGGGATCAAGAAGGGAGAATGAACTATTACTGGACACTAGTAGAGCCGGGAGACAAAATAACATTCGAAGCAACTGGAAATCTAGTGGTACCGAGATATGCATTCGCAATGGAAAGAAATGCTGGATCTGGTATTATCATTTCAGATACACCAGTCCACGATTGCAATACAACTTGTCAGACACCCAAGGGTGCTATAAACACCAGCCTCCCATTTCAGAATATACATCCGATCACAATTGGAAAATGTTCAAAATATGTAAAAAGCACAAAATTGAGACTGGCCACAGGATTGAGGAATGTCCCGTCTATTCAATCTAGAGGCCTATTTGGGGCTATTGCCGGTTTCATTGAAGGGGGGTGGACAGGGATGGTAGATGGATGGTACGGTTATCACCATCAAAATGAGCAGGGGTCAGGATATGCAGCCGACCTGAAGAGCACACAAAATGCCATTGACGAGATTACTAACAAAGTAAATTCTGTTATTGAAAAGATGAATACACAGTTCACAGCAGTAGGTAAAGAGTTCAACCACCTGGAAAAAAGAATAGAGAATTTAAATAAAAAGGTTGATGATGGTTTCCTGGACATTTGGACTTACAATGCCGAACTGTTGGTTCTATTGGAAAATGAAAGAACTTTGGACTACCACGATTCAAATGTGAAGAACTTATATGAAAAGGTAAGAAGCCAGTTAAAAAACAATGCCAAGGAAATTGGAAACGGCTGCTTTGAATTTTACCACAAATGCGATAACACGTGCATGGAAAGTATCAAAAATGGGACTTATGACTACCCAAAATACTCAGAGGAAGCAAAATTAAACAGAGAAGAAATAGATGGGGTAAAGCTGGAATCAACAAGGATTTACCAGATTTTGGCGATCTATTCAACTGTCGCCAGTTCATTGGTACTGGTAGTCTCCCTGGGGGCAATCAGTTTCTGGATGTGCTCTAATGGGTCTCTACAGTGTAGAATATGTATTTAA

>LC638223.1Influenza A virus H1N101701

ATGAAGGCAATACTAGTAGTTCTGCTATATACATTTGCAACCGCAAATGCAGACACATTATGTATAGGTTATCATGCGAACAATTCAACAGACACTGTAGACACAATACTAGAAAAGAATGTAACAGTAACACACTCTGTTAACCTTCTAGAAGACAAACATAACGGGAAACTATGCAAACTAAGAGGGGTAGCCCCATTGCATTTGGGTAAATGTAACATTGCTGGCTGGATCCTGGGAAATCCAGAGTGTGAATCACTCTCCACAGCAAGCTCATGGTCCTACATTGTGGAAACATCTAGTTCAGACAATGGAACGTGTTACCCAGGAGATTTCATCGATTATGAGGAGCTAAGAGAGCAATTGAGCTCAGTGTCATCATTTGAAAGGTTTGAGATATTCCCCAAGACAAGTTCATGGCCCAATCATGACTCGAACAAAGGTGTAACGGCAGCATGTCCTCATGCTGGAGCAAAAAGCTTCTACAAAAATTTAATATGGCTAGTTAAAAAAGGAAATTCATACCCAAAGCTCAGCAAATCCTACATTAATGATAAAGGGAAAGAAGTCCTCGTGCTATGGGGCATTCACCATCCATCTACTAGTGCTGACCAACAAAGTCTCTATCAGAATGCAGATGCATATGTTTTTGTGGGGACATCAAGATACAGCAAGAAGTTCAAGCCGGAAATAGCAATAAGACCCAAAGTGAGGGATCAAGAAGGGAGAATGAACTATTACTGGACACTAGTAGAGCCGGGAGACAAAATAACATTCGAAGCAACTGGAAATCTAGTGGTACCGAGATATGCATTCGCAATGGAAAGAAATGCTGGATCTGGTATTATCATTTCAGATACACCAGTCCACGATTGCAATACAACTTGTCAGACACCCAAGGGTGCTATAAACACCAGCCTCCCATTTCAGAATATACATCCGATCACAATTGGAAAATGTCCAAAATATGTAAAAAGCACAAAATTGAGACTGGCCACAGGATTGAGGAATGTCCCGTCTATTCAATCTAGAGGCCTATTTGGGGCCATTGCCGGTTTCATTGAAGGGGGGTGGACAGGGATGGTAGATGGATGGTACGGTTATCACCATCAAAATGAGCAGGGGTCAGGATATGCAGCCGACCTGAAGAGCACACAGAATGCCATTGACGTGATTACTAACAAGGTAAATTCTGTTATTGAAAAGATGAATACACAGTTCACAGCAGTAGGTAAAGAGTTCAACCACCTGGAAAAAAGAATAGAGAATTTAAATAAAAAGGTTGATGATGGTTTCCTGGACATTTGGACTTACAATGCCGAACTGTTGGTTCTATTGGAAAATGAAAGAACTTTGGACTACCACGATTCAAATGTGAAGAACTTATATGAAAAGGTAAGAAGCCAGTTAAAAAACAATGCCAAGGAAATTGGAAACGGCTGCTTTGAATTTTACCACAAATGCGATAACACGTGCATGGAAAGTGTCAAAAATGGGACTTATGACTACCCAAAATACTCAGAGGAAGCAAAATTAAACAGAGAAGAAATAGATGGGGTAAAGCTGGAATCAACAAGGATTTACCAGATTTTGGCGATCTATTCAACTGTCGCCAGTTCATTGGTACTGATAGTCTCCCTGGGGGCAATCAGTTTCTGGATGTGCTCTAATGGGTCTCTACAGTGTAGAATATGTATTTAA

>LC638222.1Influenza A virus H1N101701

ATGAAGGCAATACTAGTAGTTCTGCTATATACATTTGCAACCGCAAATGCAGACACATTATGTATAGGTTATCATGCGAACAATTCAACAGACACTGTAGACACAGTACTAGAAAAGAATGTAACAGTAACACACTCTGTTAACCTTCTAGAAGACAAGCATAACGGGAAACTATGCAAACTAAGAGGGGTAGCCCCATTGCATTTGGGTAAATGTAACATTGCTGGCTGGATCCTGGGAAATCCAGAGTGTGAATTACTCTCCACAGCAAGCTCATGGTCCTACATTGTGGAAACATCTAGTTCAGACAATGGAACGTGTTACCCAGGAGATTTCATCGATTATGAGGAGCTAAGAGAGCAATTGAGCTCAGTGTCATCATTTGAAAGGTTTGAGATATTCCCCAAGACAAGTTCATGGCCCAATCATGACTCGAACAAAGGTGTAACGGCAGCATGTCCTCATGCTGGAGCAAAAAGCTTCTACAAAAATTTAATATGGCTAGTTAAAAAAGGAAATTCATACCCAAAGCTCAGCAAATCCTACATTAATGATAAAGGGAAAGAAGTCCTCGTGCTATGGGGCATTCACCATCCATCTACTAGTGCTGACCAACAAAGTCTCTATCAGAATGCAGATGCATATGTTTTTGTGGGGACATCAAGATACAGCAAGAAGTTCAAGCCGGAAATAGCAATAAGACCCAAAGTGAGGGATCAAGAAGGGAGAATGAACTATTACTGGACACTAGTAGAGCCGGGAGACAAAATAACATTCGAAGCAACTGGAAATCTAGTGGTACCGAGATATGCATTCGCAATGGAAAGAAATGCTGGATCTGGTATTATCATTTCAGATACACCAGTCCACAATTGCAATACAACTTGTCAGACACCCAAGGGTGCTATAAACACCAGCCTCCCATTTCAGAATATACATCCGATCACAATTGGAAAATGTCCAAAATATGTAAAAAGCACAAAATTGAGACTGGCCACAGGATTGAGGAATGTCCCGTCTATTCAATCTAGAGGCCTATTTGGGGCCATTGCCGGCTTCATTGAAGGGGGGTGGACAGGGATGGTAGATGGATGGTACGGTTATCACCATCAAAATGAGCAGGGGTCAGGATATGCAGCCGACCTGAAGAGCACACAGAATGCCATTGACAAGATTACTAACAAAATAAATTCTGTTATTGAAAAGATGAATACACAGTTCACAGCAGTAGGTAAAGAGTTCAACCACCTGGAAAAAAGAATAGAGAATTTAAATAAAAAGGTTGATGATGGTTTCCTGGACATTTGGACTTACAATGCCGAACTGTTGGTTCTATTGGAAAATGAAAGAACTTTGGACTACCATGATTCAAATGTGAAGAACTTATATGAAAAGGTAAGAAGCCAGTTAAAAAACAATGCCAAGGAAATTGGAAACGGCTGCTTTGAATTTTACCACAAATGCGATAACACGTGCATGGAAAGTGTCAAAAATGGGACTTATGACTACCCAAAATACTCAGAGGAAGCAAAATTAAACAGAGAAGAAATAGATGGGGTAAAGCTGGAATCAACAAGGATTTACCAGATTTTGGCGATCTATTCAACTGTCGCCAGTTCATTGGTACTGGTAGTCTCCCTGGGGGCAATCAGTTTCTGGATGTGCTCTAATGGGTCTCTACAGTGTAGAATATGTATTTAA

>LC638221.1Influenza A virus H1N101701

ATGAAGGCAATACTAGTAGTTCTGCTATATACATTTGCAACCGCAAATGCAGACACATTATGTATAGGTTATCATGCGAACAATTCAACAGACACTGTAGACACAGTACTAGAAAAGAATGTAACAGTAACACACTCTGTTAACCTTCTAGAAGACAAGCATAACGGGAAACTATGCAAACTAAGAGGGGTAGCCCCATTGCATTTGGGTAAATGTAACATTGCTGGCTGGATCCTGGGAAATCCAGAGTGTGAATCACTCTCCACAGCAAGCTCATGGTCCTACATTGTGGAAACATCTAGTTCAGACAATGGAACGTGTTACCCAGGAGATTTCATCGATTATGAGGAGCTAAGAGAGCAATTGAGCTCAGTGTCATCATTTGAAAGGTTTGAGATATTCCCCAAGACAAGTTCATGGCCCAATCATGACTCAAACAAAGGTGTAACGGCAGCATGTCCTCATGCTGGAGCAAAAAGCTTCTACAAAAATTTAATATGGCTAGTTAAAAAAGGAAATTCATACCCAAAGCTCAGCAAATCCTACATTAATGATAAAGGGAAAGAAGTCCTCGTGCTATGGGGCATTCACCATCCATCTACTAGTGCTGACCAACAAAGCATCTATCAGAATGCAGATGCATATGTTTTTGTGGGGACATCAAGATACAGCAAGAAGTTCAAGCCGGAAATAGCAATAAGACCCAAAGTGAGGGATCAAGAAGGGAGAATGAACTATTACTGGACACTAGTAGAGCCGGGAGACAAAATAACATTCGAAGCAACTGGAAATCTAATGGTACCGAGATATGCATTCGCAATGGAAAGAAATGCTGGATCTGGTATTATCATTTCAGATACACCAGTCCACGATTGCAATACAACTTGTCAGACACCCAAGGGTGCTATAAACACCAGCCTCCCATTTCAGAATATACATCCGATCACAATTGGAAAATGTCCAAAATATGTAAAAAGCACAAAATTGAGACTGGCCACAGGATTGAGGAATGTCCCTTCTATTCAATCTAGAGGCCTATTTGGGGCCATTGCCGGTTTCATTGAAGGGGGGTGGACAGGGATGGTAGATGGATGGTACGGTTATCACCATCAAAATGAGCAGGGGTCAGGATATGCAGCCGACCTGAAGAGCACACAGAATGCCATTGACGAGATTACTAACAAAGTAAATTCTGTTATTGAAAAGATGAATACACAGTTCACAGCAGTAGGTAAAGAGTTCAACCACCTGGAAAAAAGAATAGAGAATTTAAATAAAAAAGTTGATGATGGTTTCCTGGACATTTGGACTTACAATGCCGAACTGTTGGTTCTATTGGAAAATGAAAGAACTTTGGACTACCACGATTCAAATGTGAAGAACTTATATGAAAAGGTAAGAAGCCAGTTAAAAAACAATGCCAAGGAAATTGGAAACGGCTGCTTTGAATTTTACCACAAATGCGATAACACGTGCATGGAAAGTGTCAAAAATGGGACTTATGACTACCCAAAATACTCAGAGGAAGCAAAATTAAACAGAGAAGAAATAGATGGGGTAAAGCTGGAATCAACAAGGATTTACCAGATCTTGGCGATCTATTCAACTGTCGCCAGTTCATTGGTACTGGTAGTCTCCCTGGGGGCAATCAGTTTCTGGATGTGCTCTAATGGGTCTCTACAGTGTAAAATATGTATTTAA

>LC638220.1Influenza A virus H1N101701

ATGAAGGCAATACTAGTAGTTCTGCTATATACATTTGCAACCGCAAATGCAGACACATTATGTATAGGTTATCATGCGAACAATTCAACAGACACTGTAGACACAGTACTAGAAAAGAATGTAACAGTAACACACTCTGTTAACCTTCTAGAAGACAAGCATAACGGGAAACTATGCAAACTAAGAGGGGTAGCCCCATTGCATTTGGGTAAATGTAACATTGCTGGCTGGATCCTGGGAAATCCAGAGTGTGAATCACTCTCCACAGCAAGCTCATGGTCCTACATTGTGGAAACATCTAGTTCAGACAATGGAACGTGTTACCCAGGAGATTTCATCGATTATGAGGAGCTAAGAGAGCAATTGAGCTCAGTGTCATCATTTGAAAGGTTTGAGATATTCCCCAAGACAATTTCATGGCCCAATCATGACTCGAACAAAGGTGTAACGGCAGCATGTCCTCATGCTGGAGCAAAAAGCTTCTACAAAAATTTAATATGGCTAGTTAAAAAAGGAAATTCATACCCAAAGCTCAGCAAATCCTACATTAATGATAAAGGGAAAGAAGTCCTCGTGCTATGGGGCATTCACCATCCATCTACTAGTGCTGACCAACAAAGTCTCTATCAGAATGCAGATGCATATGTTTTTGTGGGGACATCAAGATACAGCAAGAAGTTCAAGCCGGAAATAGCAATAAGACCCAAAGTGAGGGATCAAGAAGGGAGAATGAACTATTACTGGACACTAGTAGAGCCGGGAGACAAAATAACATTCGAAGCAACTGGAAATCTAGTGGTACCGAGATATGCATTCGCAATGGAAAGAAATGCTGGATCTGGTATTATCATTTCAGATACACCAGTCCACGATTGCAATACAACTTGTCAGACACCCAAGGGTGCTATAAACACCAGCCTCCCATTTCAGAATATACATCCGATCACAATTGGAAAATGTTCAAAATATGTAAAAAGCACAAAATTGAGACTGGCCACAGGATTGAGGAATGTCCCGTCTATTCAATCTAGAGGCCTATTTGGGGCTATTGCCGGTTTCATTGAAGGGGGGTGGACAGGGATGGTAGATGGATGGTACGGTTATCACCATCAAAATGAGCAGGGGTCAGGATATGCAGCCGACCTGAAGAGCACACAAAATGCCATTGACGTGATTACTAACAAAGTAAATTCTGTTATTGAAAAGATGAATACACAGTTCACAGCAGTAGGTAAAGAGTTCAACCACCTGGAAAAAAGAATAGAGAATTTAAATAAAAAGGTTGATGATGGTTTCCTGGACATTTGGACTTACAATGCCGAACTGTTGGTTCTATTGGAAAATGAAAGAACTTTGGACTACCACGATTCAAATGTGAAGAACTTATATGAAAAGGTAAGAAGCCAGTTAAAAAACAATGCCAAGGAAATTGGAAACGGCTGCTTTGAATTTTACCACAAATGCGATAACACGTGCATGGAAAGTATCAAAAATGGGACTTATGACTACCCAAAATACTCAGAGGAAGCAAAATTAAACAGAGAAGAAATAGATGGGGTAAAGCTGGAATCAACAAGGATTTACCAGATTTTGGCGATCTATTCAACTGTCGCCAGTTCATTGGTACTGGTAGTCTCCCTGGGGGCAATCAGTTTCTGGATGTGCTCTAATGGGTCTCTACAGTGTAGAATATGTATTTAA

>LC638219.1Influenza A virus H1N101701

ATGAAGGCAATACTAGTAGTTCTGCTATATACATTTGCAACCGCAAATGCAGACACATTATGTATAGGTTATCATGCGAACAATTCAACAGACACTGTAGACACAGTACTAGAAAAGAATGTAACAGTAACACACTCTGTTAACCTTCTAGAAGACAAGCATAACGGGAAACTATGCAAACTAAGAGGGGTAGCCCCATTGCATTTGGGTAAATGTAACATTGCTGGCTGGATCCTGGGAAATCCAGAGTGTGAATCACTCTCCACAGCAAGCTCATGGTCCTACATTGTGGAAACATCTAGTTCAGACAATGGAACGTGTTACCCAGGAGATTTCATCGATTATGAGGAGCTAAGAGAGCAATTGAGCTCAGTGTCATCATTTGAAAGGTTTGAGATATTCCCCAAGACAAGTTCATGGCCCAATCATGACTCAAACAAAGGTGTAACGGCAGCATGTCCTCATGCTGGAGCAAAAAGCTTCTACAAAAATTTAATATGGCTAGTTAAAAAAGGAAATTCATACCCAAAGCTCAGCAAATCCTACATTAATGATAAAGGGAAAGAAGTCCTCGTGCTATGGGGCATTCACCATCCATCTACTAGTGCTGACCAACAAAGCCTCTATCAGAATGCAGATGCATATGTTTTTGTGGGGACATCAAGATACAGCAAGAAGTTCAAGCCGGAAATAGAAATAAGACCCAAAGTGAGGGATCAAGAAGGGAGAATGAACTATTACTGGACACTAGTAGAGCCGGGAGACAAAATAACATTCGAAGCAACTGGAAATCTAATGGTACCGAGATATGCATTCGCAATGGAAAGAAATGCTGGATCTGGTATTATCATTTCAGATACACCAGTCCACGATTGCAATACAACTTGTCAGACACCCAAGGGTGCTATAAACACCAGCCTCCCATTTCAGAATATACATCCGATCACAATTGGAAAATGTCCAAAATATGTAAAAAGCACAAAATTGAGACTGGCCACAGGATTGAGGAATGTCCCTTCTATTCAATCTAGAGGCCTATTTGGGGCCATTGCCGGTTTCATTGAAGGGGGGTGGACAGGGATGGTAGATGGATGGTACGGTTATCACCATCAAAATGAGCAGGGGTCAGGATATGCAGCCGACCTGAAGAGCACACAGAATGCCATTGACGAGATTACTAACAAAGTAAATTCTGTTATTGAAAAGATGAATACACAGTTCACAGCAGTAGGTAAAGAGTTCAACCACCTGGAAAAAAGAATAGAGAATTTAAATAAAAAAGTTGATGATGGTTTCCTGGACATTTGGACTTACAATGCCGAACTGTTGGTTCTATTGGAAAATGAAAGAACTTTGGACTACCACGATTCAAATGTGAAGAACTTATATGAAAAGGTAAGAAGCCAGTTAAAAAACAATGCCAAGGAAATTGGAAACGGCTGCTTTGAATTTTACCACAAATGCGATAACACGTGCATGGAAAGTGTCAAAAATGGGACTTATGACTACCCAAAATACTCAGAGGAAGCAAAATTAAACAGAGAAGAAATAGATGGGGTAAAGCTGGAATCAACAAGGATTTACCAGATCTTGGCGATCTATTCAACTGTCGCCAGTTCATTGGTACTGGTAGTCTCCCTGGGGGCAATCAGTTTCTGGATGTGCTCTAATGGGTCTCTACAGTGTAGAATATGTATTTAA

>LC638218.1Influenza A virus H1N101701

ATGAAGGCAATACTAGTAGTTCTGCTATATACATTTGCAACCGCAAATGCAGACACATTATGTATAGGTTATCATGCGAACAATTCAACTGACACTGTAGACACAGTACTAGAAAAGAATGTAACAGTAACACACTCTGTTAACCTTCTAGAAGACAAGCATAACGGGAAACTATGCAAACTAAGAGGGGTAGCCCCATTGCATTTGGGTAAATGTAACATTGCTGGCTGGATCCTGGGAAATCCAGAGTGTGAATCACTCTCCACAGCAAGCTCATGGTCCTACATTGTGGAAACATCTAGTTCAGACAATGGAACGTGTTACCCAGGAGATTTCATCGATTATGAGGAGCTAAGAGAGCAATTGAGCTCAGTGTCATCATTTGAAAGGTTTGAGATATTCCCCAAGACAAGTTCATGGCCCAATCATGACTCGAACAAAGGTGTGACGGCAGCATGTCCTCATGCTGGAGCAAAAAGCTTCTACAAAAATTTAATATGGCTAGTTAAAAAAGGAAATTCATACCCAAAGCTCAGCAAATCCTACATTAATGATAAAGGGAGAGAAGTCCTCGTGCTATGGGGCATTCACCATCCATCTACTAGTGCTGACCAACAAAGTCTCTATCAGAATGCAGATACATATGTTTTTGTGGGGACATCAAGATACAGCAAGAAGTTCAAGCCGGAAATAGCAATAAGACCCAAAGTGAGGGATCAAGAAGGGAGAATGAACTATTACTGGACACTAGTAGAGCCGGGAGACAAAATAACATTCGAAGCAACTGGAAATCTAGTGGTACCGAGATATGCATTCGCAATGGAAAGAAATGCTGGATCTGGTATTATCATTTCAGATACACCAGTCAACGATTGCAATACAACTTGTCAGACACCCAAGGGTGCTATAAACACCAGCCTCCCATTTCAGAATATACATCCGATCACAATTGGAAAATGTCCAAAATATGTAAAAAGCACAAAATTGAGACTGGCCACAGGATTGAGGAATGTCCCGTCTATTCAATCTAGAGGCCTATTTGGGGCCATTGCCGGTTTCATTGAAGGGGGGTGGACAGGGATGGTAGATGGATGGTACGGTTATCACCATCAAAATGAGCAGGGGTCAGGATATGCAGCCGACCTGAAGAGCACACAGAATGCCATTGACGAGATTACTAACAAAGTAAATTCTGTTATTGAAAAGATGAATACACAGTTCACAGCAGTAGGTAAAGAGTTCAACCACCTGGAAAAAAGAATAGAGAATTTAAATAAAAAAGTTGATGATGGTTTCCTGGACATTTGGACTTACAATGCCGAACTGTTGGTTCTATTGGAAAATGAAAGAACTTTGGACTACCACGATTCAAATGTGAAGAACTTATATGAAAAGGTAAGAAGCCAGTTAAAAAACAATGCCAAGGAAATTGGAAACGGCTGCTTTGAATTTTACCACAAATGCGATAACACGTGCATGGAAAGTGTCAAAAATGGGACTTATGACTACCCAAAATACTCAGAGGAAGCAAAATTAAACAGAGAAGAAATAGATGGGGTAAAGCTGGAATCAACAAGGATTTACCAGATTTTGGCGATCTATTCAACTGTCGCCAGTTCATTGGTACTGGTAGTCTCCCTGGGGGCAATCAGTTTCTGGATGTGCTCTAATGGGTCTCTACAGTGTAGAATATGTATTTAA

>LC638217.1Influenza A virus H1N101701

ATGAAGGCAATACTAGTAGTTCTGCTATATACATTTGCAACCGCAAATGCAGACACATTATGTATAGGTTATCATGCGAACAATTCAACAGACACTGTAGACACAGTACTAGAAAAGAATGTAACAGTAACACACTCTGTTAACCTTCTAGAAGACAAGCATAACGGGAAACTATGCAAACTAAGAGGGGTAGCCCCATTGCATTTGGGTAAATGTAACATTGCTGGCTGGATCCTGGGAAATCCAGAGTGTGAATCACTCTCCACAGCAAGCTCATGGTCCTACATTGTGGAAACATCTAGTTCAGACAATGGAACGTGTTACCCAGGAGATTTCATCGATTATGAGGAGCTAAGAGAGCAATTGAGCTCAGTGTCATCATTTGAAAGGTTTGAGATATTCCCCAAGACAATTTCATGGCCCAATCATGACTCGAACAAAGGTGTAACGGCAGCATGTCCTCATGCTGGAGCAAAAAGCTTCTACAAAAATTTAATATGGCTAGTTAAAAAAGGAAATTCATACCCAAAGCTCAGCAAATCCTACATTAATGATAAAGGGAAAGAAGTCCTCGTGCTATGGGGCATTCACCATCCATCTACTAGTGCTGACCAACAAAGTCTCTATCAGAATGCAGATGCATATGTTTTTGTGGGGACATCAAGATACAGCAAGAAGTTCAAGCCGGAAATAGCAATAAGACCCAAAGTGAGGGATCAAGAAGGGAGAATGAACTATTACTGGACACTAGTAGAGCCGGGAGACAAAATAACATTCGAAGCAACTGGAAATCTAGTGGTACCGAGATATGCATTCGCAATGGAAAGAAATGCTGGATCTGGTATTATCATTTCAGATACACCAGTCCACGATTGCAATACAACTTGTCAGACACCCAAGGGTGCTATAAACACCAGCCTCCCATTTCAGAATATACATCCGATCACAATTGGAAAATGTTCAAAATATGTAAAAAGCACAAAATTGAGACTGGCCACAGGATTGAGGAATGTCCCGTCTATTCAATCTAGAGGCCTATTTGGGGCTATTGCCGGTTTCATTGAAGGGGGGTGGACAGGGATGGTAGATGGATGGTACGGTTATCACCATCAAAATGAGCAGGGGTCAGGATATGCAGCCGACCTGAAGAGCACACAAAATGCCATTGACGTGATTACTAACAAAGTAAATTCTGTTATTGAAAAGATGAATACACAGTTCACAGCAGTAGGTAAAGAGTTCAACCACCTGGAAAAAAGAATAGAGAATTTAAATAAAAAGGTTGATGATGGTTTCCTGGACATTTGGACTTACAATGCCGAACTGTTGGTTCTATTGGAAAATGAAAGAACTTTGGACTACCACGATTCAAATGTGAAGAACTTATATGAAAAGGTAAGAAGCCAGTTAAAAAACAATGCCAAGGAAATTGGAAACGGCTGCTTTGAATTTTACCACAAATGCGATAACACGTGCATGGAAAGTATCAAAAATGGGACTTATGACTACCCAAAATACTCAGAGGAAGCAAAATTAAACAGAGAAGAAATAGATGGGGTAAAGCTGGAATCAACAAGGATTTACCAGATTTTGGCGATCTATTCAACTGTCGCCAGTTCATTGGTACTGGTAGTCTCCCTGGGGGCAATCAGTTTCTGGATGTGCTCTAATGGGTCTCTACAGTGTAGAATATGTATTTAA

>LC638216.1Influenza A virus H1N101701

ATGAAGGCAATACTAGTAGTTCTGCTATATACATTTGCAACCGCAAATGCAGACACATTATGTATAGGTTATCATGCGAACAATTCAACAGACACTGTAGACACAGTACTAGAAAAGAATGTAACAGTAACACACTCTGTTAACCTCCTAGAAGACAAGCATAACGGGAAACTATGCAAACTAAGAGGGGTAGCCCCATTGCATTTGGGTAAATGTAACATTGCTGGCTGGATCCTGGGAAATCCAGAGTGTGAATCACTCTCCACAGCAAGCTCATGGTCCTACATTGTGGAAACATCTAGTTCAGACAATGGAACGTGTTACCCAGGAGATTTCATCGATTATGAGGAGCTAAGAGAGCAATTGAGCTCAGTGTCATCATTTGAAAGGTTTGAGATATTCCCCAAGACAAGTTCATGGCCCAATCATGACTCGAACAAAGGTGTAACGGCAGCATGTCCTCATGCTGGAGCAAAAAGCTTCTACAAAAATTTAATATGGCTAGTTAAAAAAGGAAATTCATACCCAAAGCTCAGCAAATCCTACATTAATGATAAAGGGAAAGAAGTCCTCGTGCTATGGGGCATTCACCATCCATCTACTAGTGCTGACCAACAAAGTCTCTATCAGAATGCAGATACATATGTTTTTGTGGGGACATCAAGATACAGCAAGAAGTTCAAGCCGGAAATAGCAATAAGACCCAAAGTGAGGGATCAAGAAGGGAGAATGAACTATTACTGGACACTAGTAGAGCCGGGAGACAAAATAACATTCGAAGCAACTGGAAATCTAGTGGTACCGAGATATGCATTCGCAATGGAAAGAAATGCTGGATCTGGTATTATCATTTCAGATACACCAGTCCACGATTGCAATACAACTTGTCAGACACCCAAGGGTGCTATAAACACCAGCCTCCCATTTCAGAATATACATCCGATCACAATTGGAAAATGTCCAAAATATGTAAAAAGCACAAAATTGAGACTGGCCACAGGATTGAGGAATATCCCGTCTATTCAATCTAGAGGCCTATTTGGGGCTATTGCCGGTTTCATTGAAGGGGGGTGGACAGGGATGGTAGATGGATGGTACGGTTATCACCATCAAAATGAGCAGGGGTCAGGATATGCAGCCGACCTGAAGAGCACACAGAATGCCATTGACGAGATTACTAACAAAGTAAATTCTGTTATTGAAAAGATGAATACACAGTTCACAGCAGTAGGTAAAGAGTTCAACCACCTGGAAAAAAGAATAGAGAATTTAAATAAAAAAGTTGATGATGGTTTCCTGGACATTTGGACTTACAATGCCGAACTGTTGGTTCTATTGGAAAATGAAAGAACTTTGGACTACCACGATTCAAATGTGAAGAACTTATATGAAAAGGTAAGAAGCCAGTTAAAAAACAATGCCAAGGAAATTGGAAACGGCTGCTTTGAATTTTACCACAAATGCGATAACACGTGCATGGAAAGTGTCAAAAATGGGACTTATGACTACCCAAAATACTCAGAGGAAGCAAAATTAAACAGAGAAGAAATAGATGGGGTAAAGCTGGAATCAACAAGGATTTACCAGATTTTGGCGATCTATTCAACTGTCGCCAGTTCATTGGTACTGGTAGTCTCCCTGGGGGCAATCAGTTTCTGGATGTGCTCTAATGGGTCTCTACAGTGTAGAATATGTATTTAA

>LC638215.1Influenza A virus H1N101701

ATGAAGGCAATACTAGTAGTTCTGCTATATACATTTGCAACCGCAAATGCAGACACATTATGTATAGGTTATCATGCGAACAATTCAACAGACACTGTAGACACAATACTAGAAAAGAATGTAACAGTAACACACTCTGTTAACCTTCTAGAAGACAAGCATAACGGGAAACTATGCAAACTAAGAGGGGTAGCCCCATTGCATTTGGGTAAATGTAACATTGCTGGCTGGATCCTGGGAAATCCAGAGTGTGAATCACTCTCCACAGCAAGCTCATGGTCCTACATTGTGGAAACATCTAGTTCAGACAATGGAACGTGTTACCCAGGAGATTTCATCGATTATGAGGAGCTAAGAGAGCAATTGAGCTCAGTGTCATCATTTGAACGGTTTGAGATATTCCCCAAGACAAGTTCATGGCCCAATCATGACTCGAACAAAGGTGTAACGGCAGCATGTCCTCATGCTGGAGCAAAAAGCTTCTACAAAAATTTAATATGGCTAGTTAAAAAAGGAAATTCATACCCAAAGCTCAGCAAATCCTACATTAATGATAAAGGGAAAGAAGTCCTCGTGCTATGGGGCATTCACCATCCATCTACTAGTGCTGACCAACAAAGTCTCTATCAGAATGCAGATGCATATGTTTTTGTGGGGACATCAAGATACAGCAAGAAGTTCAAGCCGGAAATAGCAATAAGACCCAAAGTGAGGGATCAAGAAGGGAGAATGAACTATTACTGGACACTAGTAGAGCCGGGAGACAAAATAACATTCGAAGCAACTGGAAATCTAGTGGTACCGAGATATGCATTCGCAATGGAAAGAAATGCTGGATCTGGTATTATCATTTCAGATACACCAGTCCACGATTGCAATACAACTTGTCAGACACCCAAGGGTGCTATAAACACCAGCCTCCCATTTCAGAATATACATCCGATCACAATTGGAAAATGTCCAAAATATGTAAAAAGCACAAAATTGAGACTGGCCACAGGATTGAGGAATGTCCCGTCTATTCAATCTAGAGGCCTATTTGGGGCCATTGCCGGTTTCATTGAAGGGGGGTGGACAGGGATGGTAGATGGATGGTACGGTTATCACCATCAAAATGAGCAGGGGTCAGGATATGCAGCCGACCTGAAGAGCACACAGAATGCCATTGACGAGATTACTAACAAAGTAAATTCTGTTATTGAAAAGATGAATACACAGTTCACAGCAGTAGGTAAAGAGTTCAACCACCTGGAAAAAAGAATAGAGAATTTAAATAAAAAGGTTGATGATGGTTTCCTGGACATTTGGACTTACAATGCCGAACTGTTGGTTCTATTGGAAAATGAAAGAACTTTGGACTACCACGATTCAAATGTGAAGAACTTATATGAAAAGGTAAGAAGCCAGTTAAAAAACAATGCCAAGGAAATTGGAAACGGCTGCTTTGAATTTTACCACAAATGCGATAACACGTGCATGGAAAGTGTCAAAAATGGGACTTATGACTACCCAAAATACTCAGAGGAAGCAAAATTAAACAGAGAAGAAATAGATGGGGTAAAGCTGGAATCAACAAGGATTTACCAGATTTTGGCGATCTATTCAACTGCCGCCAGTTCATTGGTACTGGTAGTCTCCCTGGGGGCAATCAGTTTCTGGATGTGCTCTAATGGGTCTCTACAGTGTAGAATATGTATTTAA

>LC638214.1Influenza A virus H1N101701

ATGAAGGCAATACTAGTAGTTCTGCTATATACATTTGCAACCGCAAATGCAGACACATTATGTATAGGTTATCATGCGAACAATTCAACAGACACTGTAGACACAGTACTAGAAAAGAATGTAACAGTAACACACTCTGTTAACCTTCTAGAAGACAAGCATAACGGGAAACTATGCAAACTAAGAGGGGTAGCCCCATTGCATTTGGGTAAATGTAACATTGCTGGCTGGATCCTGGGAAATCCAGAGTGTGAATCACTCTCCACAGCAAGCTCATGGTCCTACATTGTGGAAACATCTAGTTCAGACAATGGAACGTGTTACCCAGGAGATTTCATCGATTATGAGGAGCTAAGAGAGCAATTGAGCTCAGTGTCATCATTTGAAAGGTTTGAGATATTCCCCAAGACAAGTTCATGGCCCAATCATGACTCGAACAAAGGTGTAACGGCAGCATGTCCTCATGCTGGAGCAAAAAGCTTCTACAAAAATTTAATATGGCTAGTTAAAAAAGGAAATTCATACCCAAAGCTCAGCAAATCCTACATTAATGATAAAGGGAAAGAAGTCCTCGTGCTATGGGGCATTCACCATCCATCTACTAGTGCTGACCAACAAAGTCTCTATCAGAATGCAGATGCATATGTTTTTGTGGGGACATCAAGATACAGCAAGAAGTTCAAGCCGGAAATAGCAATAAGACCCAAAGTGAGGGATCAAGAAGGGAGAATGAACTATTACTGGACACTAGTAGAGCCGGGAGACAAAATAACATTCGAAGCAACTGGAAATCTAGTGGTACCGAGATACGCATTCGCAATGGAAAGAAATGCTGGATCTGGTATTATCATTTCAGATACACCAGTCCATGATTGCAATACAACTTGTCAGACACCCAAGGGTGCTATAAACACCAGCCTCCCATTTCAGAATATACATCCGATCACAATTGGAAAATGTCCAAAATATGTAAAAAGCACAAAATTGAGACTGGCCACAGGATTGAGGAATGTCCCGTCTATTCAATCTAGAGGCCTATTTGGGGCCATTGCCGGCTTCATTGAAGGGGGGTGGACAGGGATGGTAGATGGATGGTACGGTTATCACCATCAAAATGAGCAGGGGTCAGGATATACAGCCGACCTGAAGAGCACACAGAATGCCATTGACAAGATTACTAACAAAGTAAATTCTGTTATTGAAAAGATGAATACACAGTTCACAGCAGTAGGTAAAGAGTTCAACCACCTGGAAAAAAGAATAGAGAATTTAAATAAAAAAGTTGATGATGGTTTCCTGGACATTTGGACTTACAATGCCGAACTGTTGGTTCTATTGGAAAATGAAAGAACTTTGGACTACCACGATTCAAATGTGAAGAACTTATATGAAAAGGTAAGAAGCCAGTTAAAAAACAATGCCAAGGAAATTGGAAACGGCTGCTTTGAATTTTACCACAAATGCGATAACACGTGCATGGAAAGTGTCAAAAATGGGACTTATGACTACCCAAAATACTCAGAGGAAGCAAAATTAAACAGAGAAGAAATAGATGGGGTAAAGCTGGAATCAACAGGGATTTACCAGATTTTGGCGATCTATTCAACTGTCGCCAGTTCATTGGTACTGGTAGTCTCCCTGGGGGCAATCAGTTTCTGGATGTGCTCTAATGGGTCTCTACAGTGTAGAATATGTATTTAA

>LC638213.1Influenza A virus H1N101701

ATGAAGGCAATACTAGTAGTTCTGCTATATACATTTGCAACCGCAAATGCAGACACATTATGTATAGGTTATCATGCGAACAATTCAACAGACACTGTAGACACAGTACTAGAAAAGAATGTAACAGTAACACACTCTGTTAACCTCCTAGAAGACAAGCATAACGGGAAACTATGCAAACTAAGAGGGGTAGCCCCATTGCATTTGGGTAAATGTAACATTGCTGGCTGGATCCTGGGAAATCCAGAGTGTGAATCACTCTCCACAGCAAGCTCATGGTCCTACATTGTGGAAACATCTAGTTCAGACAATGGAACGTGTTACCCAGGAGATTTCATCGATTATGAGGAGCTAAGAGAGCAATTGAGCTCAGTGTCATCATTTGAAAGGTTTGAGATATTCCCCAAGACAAGTTCATGGCCCAATCATGACTCGAACAAAGGTGTAACGGCAGCATGTCCTCATGCTGGAGCAAAAAGCTTCTACAAAAATTTAATATGGCTAGTTAAAAAAGGAAATTCATACCCAAAGCTCAGCAAATCCTACATTAATGATAAAGGGAAAGAAGTCCTCGTGCTATGGGGCATTCACCATCCATCTACTAGTGCTGACCAACAAAGTCTCTATCAGAATGCAGATACATATGTTTTTGTGGGGACATCAAGATACAGCAAGAAGTTCAAGCCGGAAATAGCAATAAGACCCAAAGTGAGGGATCAAGAAGGGAGAATGAACTATTACTGGACACTAGTAGAGCCGGGAGACAAAATAACATTCGAAGCAACTGGAAATCTAGTGGTACCGAGATATGCATTCGCAATGGAAAGAAATGCTGGATCTGGTATTATCATTTCAGATACACCAGTCCACGATTGCAATACAACTTGTCAGACACCCAAGGGTGCTATAAACACCAGCCTCCCATTTCAGAATATACATCCGATCACAATTGGAAAATGTCCAAAATATGTAAAAAGCACAAAATTGAGACTGGCCACAGGATTGAGGAATATCCCGTCTATTCAATCTAGAGGCCTATTTGGGGCTATTGCCGGTTTCATTGAAGGGGGGTGGACAGGGATGGTAGATGGATGGTACGGTTATCACCATCAAAATGAGCAGGGGTCAGGATATGCAGCCGACCTGAAGAGCACACAGAATGCCATTGACGAGATTACTAACAAAGTAAATTCTGTTATTGAAAAGATGAATACACAGTTCACAGCAGTAGGTAAAGAGTTCAACCACCTGGAAAAAAGAATAGAGAATTTAAATAAAAAAGTTGATGATGGTTTCCTGGACATTTGGACTTACAATGCCGAACTGTTGGTTCTATTGGAAAATGAAAGAACTTTGGACTACCACGATTCAAATGTGAAGAACTTATATGAAAAGGTAAGAAGCCAGTTAAAAAACAATGCCAAGGAAATTGGAAACGGCTGCTTTGAATTTTACCACAAATGCGATAACACGTGCATGGAAAGTGTCAAAAATGGGACTTATGACTACCCAAAATACTCAGAGGAAGCAAAATTAAACAGAGAAGAAATAGATGGGGTAAAGCTGGAATCAACAAGGATTTACCAGATTTTGGCGATCTATTCAACTGTCGCCAGTTCATTGGTACTGGTAGTCTCCCTGGGGGCAATCAGTTTCTGGATGTGCTCTAATGGGTCTCTACAGTGTAGAATATGTATTTAA

>LC638212.1Influenza A virus H1N101701

ATGAAGGCAATACTAGTAGTTCTGCTATATACATTTGCAACCGCAAATGCAGACACATTATGTATAGGTTATCATGCGAACAATTCAACAGACACTGTAGACACAGTACTAGAAAAGAATGTAACAGTAACACACTCTGTTAACCTTCTAGAAGACAAGCATAACGGGAAACTATGCAAACTAAGAGGGGTAGCCCCATTGCATTTGGGTAAATGTAACATTGCTGGCTGGATCCTGGGAAATCCAGAGTGTGAATCACTCTCCACAGCAAGCTCATGGTCCTACATTGTAGAAACATCTAGTTCAGACAATGGAACGTGTTACCCAGGAGATTTCATCGATTATGAGGAGCTAAGAGAGCAATTGAGCTCAGTGTCATCATTTGAAAGGTTTGAGATATTCCCCAAGACAAGTTCATGGCCCAATCATGACTCGAACAAAGGTGTAACGGCAGCATGTCCTCATGCTGGAGCAAAAAGCTTCTACAAAAATTTAATATGGCTAGTTAAAAAAGGAAATTCATACCCAAAGCTCAGCAAATCCTACATTAATGATAAAGGGAAAGAAGTCCTCGTGCTATGGGGCATTCACCATCCATCTACTAGTGCTGACCAACAAAGTCTCTATCAGAATGCAGATGCATATGTTTTTGTGGGGACATCAAGATACAGCAAGAAGTTCAAGCCGGAAATAGCAATAAGACCCAAAGTGAGGGATCAAGAAGGGAGAATGAACTATTACTGGACACTAGTAGAGCCGGGAGACAAAATAACATTCGAAGCAACTGGAAATCTAGTGGTACCGAGATATGCATTCGCAATGGAAAGAAATGCTGGATCTGGTATTATCATTTCAGATACACCAGCCCACGATTGCAATACAACTTGTCAGACACCCAAGGGTGCTATAAACACCAGCCTCCCATTTCAGAATATACATCCGATCACAATTGGAAAATGTCCAAAATATGTAAAAAGCACAAAATTGAGACTGGCCACAGGATTGAGGAATGTCCCGTCTATTCAATCTAGAGGCCTATTTGGGGCCATAGCCGGTTTCATTGAAGGGGGGTGGACAGGGATGGTAGATGGATGGTACGGTTATCACCATCAAAATGAGCAGGGGTCAGGATATGCAGCCGACCTGAAGAGCACACAGAATGCCATTGACAAGATTACTAACAAAGTAAATTCTGTTATTGAAAAGATGAATACACAGTTCACAGCAGTAGGTAAAGAGTTCAACCACCTGGAAAAAAGAATAGAGAATTTAAATAAAAAAGTTGATGATGGTTTCCTGGACATTTGGACTTACAATGCCGAACTGTTGGTTCTATTGGAAAATGAAAGAACTTTGGACTACCACGATTCAAATGTGAAGAACTTATATGAAAAGGTAAGAAGCCAGTTAAAAAACAATGCCAAGGAAATTGGAAACGGCTGCTTTGAATTTTACCACAAATGCGATAACACGTGCATGGAAAGTGTCAAAAATGGGACTTATGACTACCCCAAATACTCAGAGGAAGCAAAATTAAACAGAGAAGAAATAGATGGGGTAAAGCTGGAATCAACAAGGATTTACCAGATTTTGGCGATCTATTCAACTGTCGCCAGTTCATTGGTACTGGTAGTCTCCCTGGGGGCAATCAGTTTCTGGATGTGCTCTAATGGGTCTCTACAGTGTAGAATATGTATTTAA

>LC638211.1Influenza A virus H1N101701

ATGAAGGCAATACTAGTAGTTCTGCTATATACATTTGCAACCGCAAATGCAGACACATTATGTATAGGTTATCATGCGAACAATTCAACAGACACTGTAGACACAGTACTAGAAAAGAATGTAACAGTAACACACTCTGTTAACCTTCTAGAAGACAAGCATAACGGGAAACTATGCAAACTAAGAGGGGTAGCCCCATTGCATTTGGGTAAATGTAACATTGCTGGCTGGATCCTGGGAAATCCAGAGTGTGAATCACTCTCCACAGCAAGCTCATGGTCCTACATTGTGGAAACATCTAGTTCAGACAATGGAACGTGTTACCCAGGAGATTTCATCGATTATGAGGAGCTAAGAGAGCAATTGAGCTCAGTGTCATCATTTGAAAGGTTTGAGATATTCCCCAAGACAAGTTCATGGCCCAATCATGACTCGAACAAAGGTGTAACGGCAGCATGTCCTCATGCTGGAGCAAAAAGCTTCTACAAAAATTTAATATGGCTAGTTAAAAAAGGAAATTCATACCCAAAGCTCAGCAAATCCTACATTAATGATAAAGGGAAAGAAGTCCTCGTGCTATGGGGCATTCACCATCCATCTACTAGTGCTGACCAACAAAGTCTCTATCAGAATGCAGATGCATATGTTTTTGTGGGGACATCAAGATACAGCAAGAAGTTCAAGCCGGAAATAGCAATAAGACCCAAAGTGAGGGATCAAGAAGGGAGAATGAACTATTACTGGACACTAGTAGAGCCGGGAGACAAAATAACATTCGAAGCAACTGGAAATCTAGTGGTACCGAGATACGCATTCGCAATGGAAAGAAATGCTGGATCTGGTATTATCATTTCAGATACACCAGTCCATGATTGCAATACAACTTGTCAGACACCCAAGGGTGCTATAAACACCAGCCTCCCATTTCAGAATATACATCCGATCACAATTGGAAAATGTCCAAAATATGTAAAAAGCACAAAATTGAGACTGGCCACAGGATTGAGGAATGTCCCGTCTATTCAATCTAGAGGCCTATTTGGGGCCATTGCCGGCTTCATTGAAGGGGGGTGGACAGGGATGGTAGATGGATGGTACGGTTATCACCATCAAAATGAGCAGGGGTCAGGATATACAGCCGACCTGAAGAGCACACAGAATGCCATTGACAAGATTACTAACAAAGTAAATTCTGTTATTGAAAAGATGAATACACAGTTCACAGCAGTAGGTAAAGAGTTCAACCACCTGGAAAAAAGAATAGAGAATTTAAATAAAAAAGTTGATGATGGTTTCCTGGACATTTGGACTTACAATGCCGAACTGTTGGTTCTATTGGAAAATGAAAGAACTTTGGACTACCACGATTCAAATGTGAAGAACTTATATGAAAAGGTAAGAAGCCAGTTAAAAAACAATGCCAAGGAAATTGGAAACGGCTGCTTTGAATTTTACCACAAATGCGATAACACGTGCATGGAAAGTGTCAAAAATGGGACTTATGACTACCCAAAATACTCAGAGGAAGCAAAATTAAACAGAGAAGAAATAGATGGGGTAAAGCTGGAATCAACAGGGATTTACCAGATTTTGGCGATCTATTCAACTGTCGCCAGTTCATTGGTACTGGTAGTCTCCCTGGGGGCAATCAGTTTCTGGATGTGCTCTAATGGGTCTCTACAGTGTAGAATATGTATTTAA

>LC638210.1Influenza A virus H1N101701

ATGAAGGCAATACTAGTAGTTCTGCTATATACATTTGCAACCGCAAATGCAGACACATTATGTATAGGTTATCATGCGAACAATTCAACAGACACTGTAGACACAGTACTAGAAAAGAATGTAACAGTAACACACTCTGTTAACCTCCTAGAAGACAAGCATAACGGGAAACTATGCAAACTAAGAGGGGTAGCCCCATTGCATTTGGGTAAATGTAACATTGCTGGCTGGATCCTGGGAAATCCAGAGTGTGAATCACTCTCCACAGCAAGCTCATGGTCCTACATTGTGGAAACATCTAGTTCAGACAATGGAACGTGTTACCCAGGAGATTTCATCGATTATGAGGAGCTAAGAGAGCAATTGAGCTCAGTGTCATCATTTGAAAGGTTTGAGATATTCCCCAAGACAAGTTCATGGCCCAATCATGACTCGAACAAAGGTGTAACGGCAGCATGTCCTCATGCTGGAGCAAAAAGCTTCTACAAAAATTTAATATGGCTAGTTAAAAAAGGAAATTCATACCCAAAGCTCAGCAAATCCTACATTAATGATAAAGGGAAAGAAGTCCTCGTGCTATGGGGCATTCACCATCCATCTACTAGTGCTGACCAACAAAGTCTCTATCAGAATGCAGATACATATGTTTTTGTGGGGACATCAAGATACAGCAAGAAGTTCAAGCCGGAAATAGCAATAAGACCCAAAGTGAGGGATCAAGAAGGGAGAATGAACTATTACTGGACACTAGTAGAGCCGGGAGACAAAATAACATTCGAAGCAACTGGAAATCTAGTGGTACCGAGATATGCATTCGCAATGGAAAGAAATGCTGGATCTGGTATTATCATTTCAGATACACCAGTCCACGATTGCAATACAACTTGTCAGACACCCAAGGGTGCTATAAACACCAGCCTCCCATTTCAGAATATACATCCGATCACAATTGGAAAATGTCCAAAATATGTAAAAAGCACAAAATTGAGACTGGCCACAGGATTGAGGAATATCCCGTCTATTCAATCTAGAGGCCTATTTGGGGCTATTGCCGGTTTCATTGAAGGGGGGTGGACAGGGATGGTAGATGGATGGTACGGTTATCACCATCAAAATGAGCAGGGGTCAGGATATGCAGCCGACCTGAAGAGCACACAGAATGCCATTGACGAGATTACTAACAAAGTAAATTCTGTTATTGAAAAGATGAATACACAGTTCACAGCAGTAGGTAAAGAGTTCAACCACCTGGAAAAAAGAATAGAGAATTTAAATAAAAAAGTTGATGATGGTTTCCTGGACATTTGGACTTACAATGCCGAACTGTTGGTTCTATTGGAAAATGAAAGAACTTTGGACTACCACGATTCAAATGTGAAGAACTTATATGAAAAGGTAAGAAGCCAGTTAAAAAACAATGCCAAGGAAATTGGAAACGGCTGCTTTGAATTTTACCACAAATGCGATAACACGTGCATGGAAAGTGTCAAAAATGGGACTTATGACTACCCAAAATACTCAGAGGAAGCAAAATTAAACAGAGAAGAAATAGATGGGGTAAAGCTGGAATCAACAAGGATTTACCAGATTTTGGCGATCTATTCAACTGTCGCCAGTTCATTGGTACTGGTAGTCTCCCTGGGGGCAATCAGTTTCTGGATGTGCTCTAATGGGTCTCTACAGTGTAGAATATGTATTTAA

>LC638209.1Influenza A virus H1N101701

ATGAAGGCAATACTAGTAGTTCTGCTATATACATTTGCAACCGCAAATGCAGACACATTATGTATAGGTTATCATGCGAACAATTCAACTGACACTGTAGACACAGTACTAGAAAAGAATGTAACAGTAACACACTCTGTTAACCTTCTAGAAGACAAGCATAACGGGAAACTATGCAAACTAAGAGGGGTAGCCCCATTGCATTTGGGTAAATGTAACATTGCTGGCTGGATCCTGGGAAATCCAAAGTGTGAATCACTCTCCACAGCAAGCTCATGGTCCTACATTGTGGAAACATCTAGTTCAGACAATGGAACGTGTTACCCAGGAGATTTCATCGATTATGAGGAGCTAAGAGAGCAATTGAGCTCAGTGTCATCATTTGAAAGGTTTGAGATATTCCCCAAGACAAGTTCATGGCCCAATCATGACTCGAACAAAGGTGTGACGGCAGCATGTCCTCATGCTGGAGCAAAAAGCTTCTACAAAAATTTAATATGGCTAGTTAAAAAAGGAAATTCATACCCAAAGCTCAGCAAATCCTACATTAATGATAAAGGGAGAGAAGTCCTCGTGCTATGGGGCATTCACCATCCATCTACTAGTGCTGACCAACAAAGTCTCTATCAGAATGCAGATACATATGTTTTTGTGGGGACATCAAGATACAGCAAGAAGTTCAAGCCGGAAATAGCAATAAGACCCAAAGTGAGGGATCAAGAAGGGAGAATGAACTATTACTGGACACTAGTAGAGCCGGGAGACAAAATAACATTCGAAGCAACTGGAAATCTAGTGGTACCGAGATATGCATTCGCAATGGAAAGAAATGCTGGATCTGGTATTATCATTTCAGATACACCAGTCCACGATTGCAATACAACTTGTCAGACACCCAAGGGTGCTATAAACACCAGCCTCCCATTTCAGAATATACATCCGATCACAATTGGAAAATGTCCAAAATATGTAAAAAGCACAAAATTGAGACTGGCCACAGGATTGAGGAATGTCCCGTCTATTCAATCTAGAGGCCTATTTGGGGCCATTGCCGGTTTCATTGAAGGGGGGTGGACAGGGATGGTAGATGGATGGTACGGTTATCACCATCAAAATGAGCAGGGGTCAGGATATGCAGCCGACTTGAAGAGCACACAGAATGCCATTGACGAGATTACTAACAAAGTAAATTCTGTTATTGAAAAGATGAATACACAGTTCACAGCAGTAGGTAAAGAGTTCAACCACCTGGAAAAAAGAATAGAGAATTTAAATAAAAAAGTTGATGATGGTTTCCTGGACATTTGGACTTACAATGCCGAACTGTTGGTTCTATTGGAAAATGAAAGAACTTTGGACTACCACGATTCAAATGTGAAGAACTTATATGAAAAGGTAAGAAGCCAGTTAAAAAACAATGCCAAGGAAATTGGAAACGGCTGCTTTGAATTTTACCACAAATGCGATAACACGTGCATGGAAAGTGTCAAAAATGGGACTTATGACTACCCAAAATACTCAGAGGAAGCAAAATTAAACAGAGAAGAAATAGATGGGGTAAAGCTGGAATCAACAAGGATTTACCAGATTTTGGCGATCTATTCAACTGTCGCCAGTTCATTGGTACTGGTAGTCTCCCTGGGGGCAATCAGTTTCTGGATGTGCTCTAATGGGTCTCTACAGTGTAGAATATGTATTTAA

>LC638208.1Influenza A virus H1N101701

ATGAAGGCAATACTAGTAGTTCTGCTATATACATTTGCAACCGCAAATGCAGACACATTATGTATAGGTTATCATGCGAACAATTCAACTGACACTGTAGACACAGTACTAGAAAAGAATGTAACAGTAACACACTCTGTTAACCTTCTAGAAGACAAGCATAACGGGAAACTATGCAAACTAAGAGGGGTAGCCCCATTGCATTTGGGTAAATGTAACATTGCTGGCTGGATCCTGGGAAATCCAGAGTGTGAATCACTCTCCACAGCAAGCTCATGGTCCTACATTGTGGAAACATCTAGTTCAGACAATGGAACGTGTTACCCAGGAGATTTCATCGATTATGAGGAGCTAAGAGAGCAATTGAGCTCAGTGTCATCATTTGAAAGGTTTGAGATATTCCCCAAGACAAGTTCATGGCCCAATCATGACTCGAACAAAGGTGTGACGGCAGCATGTTCTCATGCTGGAGCAAAAAGCTTCTACAAAAATTTAATATGGCTAGTTAAAAAAGGAAATTCATACCCAAAGCTCAGCAAATCCTACATTAATGATAAAGGGAGAGAAGTCCTCGTGCTATGGGGCATTCACCATCCATCTACTAGTGCTGACCAACAAAGTCTCTATCAGAATGCAGATACATATGTTTTTGTGGGGACATCAAGATACAGCAAGAAGTTCAAGCCGGAAATAGCAATAAGACCCAAAGTGAGGGATCAAGAAGGGAGAATGAACTATTACTGGACACTAGTAGAGCCGGGAGACAAAATAACATTCGAAGCAACTGGAAATCTAGTGGTACCGAGATATGCATTCGCAATGGAAAGAAATGCTGGATCTGGTATTATCATTTCAGATACACCAGTCAACGATTGCAATACAACTTGTCAGACACCCAAGGGTGCTATAAACACCAGCCTCCCATTTCAGAATATACATCCGATCACAATTGGAAAATGTCCAAAATATGTAAAAAGCACAAAATTGAGACTGGCCACAGGATTGAGGAATGTCCCGTCTATTCAATCTAGAGGCCTATTTGGGGCCATTGCCGGTTTCATTGAAGGGGGGTGGACAGGGATGGTAGATGGATGGTACGGTTATCACCATCAAAATGAGCAGGGGTCAGGATATGCAGCCGACCTGAAGAGCACACAGAATGCCATTGACGAGATTACTAACAAAGTAAATTCTGTTATTGAAAAGATGAATACACAGTTCACAGCAGTAGGTAAAGAGTTCAACCACCTGGAAAAAAGAATAGAGAATTTAAATAAAAAAGTTGATGATGGTTTCCTGGACATTTGGACTTACAATGCCGAACTGTTGGTTCTATTGGAAAATGAAAGAACTTTGGACTACCACGATTCAAATGTGAAGAACTTATATGAAAAGGTAAGAAGCCAGTTAAAAAACAATGCCAAGGAAATTGGAAACGGCTGCTTTGAATTTTACCACAAATGCGATAACACGTGCATGGAAAGTGTCAAAAATGGGACTTATGACTACCCAAAATACTCAGAGGAAGCAAAATTAAACAGAGAAGAAATAGATGGGGTAAAGCTGGAATCAACAAGGATTTACCAGATTTTGGCGATCTATTCAACTGTCGCCAGTTCATTGGTACTGGTAGTCTCCCTGGGGGCAATCAGTTTCTGGATGTGCTCTAATGGGTCTCTACAGTGTAGAATATGTATTTAA

>LC638207.1Influenza A virus H1N101701

ATGAAGGCAATACTAGTAGTTCTGCTATATACATTTGCAACCGCAAATGCAGACACATTATGTATAGGTTATCATGCGAACAATTCAACAGACACTGTAGACACAGTACTAGAAAAGAATGTAACAGTAACACACTCTGTTAACCTTCTAGAAGACAAGCATAACGGGAAACTATGCAAACTAAGAGGGGTAGCCCCATTGCATTTGGGTAAATGTAACATTGCTGGCTGGATCCTGGGAAATCCAGAGTGTGAATCACTCTCCACAGCAAGCTCATGGTCCTACATTGTGGAAACATCTAGTTCAGACAATGGAACGTGTTACCCAGGAGATTTCATCGATTATGAGGAGCTAAGAGAGCAATTGAGCTCAGTGTCATCATTTGAAAGGTTTGAGATATTCCCCAAGACAAGTTCATGGCCCAATCATGACTCGAACAAAGGTGTAACGGCAGCATGTCCTCATGCTGGAGCAAAAAGCTTCTACAAAAATTTAATATGGCTAGTTAAAAAAGGAAATTCATACCCAAAGCTCAGCAAATCCTACATTAATGATAAAGGGAAAGAAGTCCTCGTGCTATGGGGCATTCACCATCCATCTACTAGTGCTGACCAACAAAGTCTCTATCAGAATGCAGATGCATATGTTTTTGTGGGGACATCAAGATACAGCAAGAAGTTCAAGCCGGAAATAGCAATAAGACCCAAAGTGAGGGATCAAGAAGGGAGAATGAACTATTACTGGACACTAGTAGAGCCGGGAGACAAAATAACATTCGAAGCAACTGGAAATCTAGTGGTACCGAGATACGCATTCGCAATGGAAAGAAATGCTGGATCTGGTATTATCATTTCAGATACACCAGTCCATGATTGCAATACAACTTGTCAGACACCCAAGGGTGCTATAAACACCAGCCTCCCATTTCAGAATATACATCCGATCACAATTGGAAAATGTCCAAAATATGTAAAAAGCACAAAATTGAGACTGGCCACAGGATTGAGGAATGTCCCGTCTATTCAATCTAGAGGCCTATTTGGGGCCATTGCCGGCTTCATTGAAGGGGGGTGGACAGGGATGGTAGATGGATGGTACGGTTATCACCATCAAAATGAGCAGGGGTCAGGATATACAGCCGACCTGAAGAGCACACAGAATGCCATTGACAAGATTACTAACAAAGTAAATTCTGTTATTGAAAAGATGAATACACAGTTCACAGCAGTAGGTAAAGAGTTCAACCACCTGGAAAAAAGAATAGAGAATTTAAATAAAAAAGTTGATGATGGTTTCCTGGACATTTGGACTTACAATGCCGAACTGTTGGTTCTATTGGAAAATGAAAGAACTTTGGACTACCACGATTCAAATGTGAAGAACTTATATGAAAAGGTAAGAAGCCAGTTAAAAAACAATGCCAAGGAAATTGGAAACGGCTGCTTTGAATTTTACCACAAATGCGATAACACGTGCATGGAAAGTGTCAAAAATGGGACTTATGACTACCCAAAATACTCAGAGGAAGCAAAATTAAACAGAGAAGAAATAGATGGGGTAAAGCTGGAATCAACAGGGATTTACCAGATTTTGGCGATCTATTCAACTGTCGCCAGTTCATTGGTACTGGTAGTCTCCCTGGGGGCAATCAGTTTCTGGATGTGCTCTAATGGGTCTCTACAGTGTAGAATATGTATTTAA

>LC638206.1Influenza A virus H1N101701

ATGAAGGCAATACTAGTAGTTCTGCTATATACATTTGCAACCGCAAATGCAGACACATTATGTATAGGTTATCATGCGAACAATTCAACAGACACTGTAGACACAGTACTAGAAAAGAATGTAACAGTAACACACTCTGTTAACCTCCTAGAAGACAAGCATAACGGGAAACTATGCAAACTAAGAGGGGTAGCCCCATTGCATTTGGGTAAATGTAACATTGCTGGCTGGATCCTGGGAAATCCAGAGTGTGAATCACTCTCCACAGCAAGCTCATGGTCCTACATTGTGGAAACATCTAGTTCAGACAATGGAACGTGTTACCCAGGAGATTTCATCGATTATGAGGAGCTAAGAGAGCAATTGAGCTCAGTGTCATCATTTGAAAGGTTTGAGATATTCCCCAAGACAAGTTCATGGCCCAATCATGACTCGAACAAAGGTGTAACGGCAGCATGTCCTCATGCTGGAGCAAAAAGCTTCTACAAAAATTTAATATGGCTAGTTAAAAAAGGAAATTCATACCCAAAGCTCAGCAAATCCTACATTAATGATAAAGGGAAAGAAGTCCTCGTGCTATGGGGCATTCACCATCCATCTACTAGTGCTGACCAACAAAGTCTCTATCAGAATGCAGATACATATGTTTTTGTGGGGACATCAAGATACAGCAAGAAGTTCAAGCCGGAAATAGCAATAAGACCCAAAGTGAGGGATCAAGAAGGGAGAATGAACTATTACTGGACACTAGTAGAGCCGGGAGACAAAATAACATTCGAAGCAACTGGAAATCTAGTGGTACCGAGATATGCATTCGCAATGGAAAGAAATGCTGGATCTGGTATTATCATTTCAGATACACCAGTCCACGATTGCAATACAACTTGTCAGACACCCAAGGGTGCTATAAACACCAGCCTCCCATTTCAGAATATACATCCGATCACAATTGGAAAATGTCCAAAATATGTAAAAAGCACAAAATTGAGACTGGCCACAGGATTGAGGAATATCCCGTCTATTCAATCTAGAGGCCTATTTGGGGCTATTGCCGGTTTCATTGAAGGGGGGTGGACAGGGATGGTAGATGGATGGTACGGTTATCACCATCAAAATGAGCAGGGGTCAGGATATGCAGCCGACCTGAAGAGCACACAGAATGCCATTGACGTGATTACTAACAAAGTAAATTCTGTTATTGAAAAGATGAATACACAGTTCACAGCAGTAGGTAAAGAGTTCAACCACCTGGAAAAAAGAATAGAGAATTTAAATAAAAAAGTTGATGATGGTTTCCTGGACATTTGGACTTACAATGCCGAACTGTTGGTTCTATTGGAAAATGAAAGAACTTTGGACTACCACGATTCAAATGTGAAGAACTTATATGAAAAGGTAAGAAGCCAGTTAAAAAACAATGCCAAGGAAATTGGAAACGGCTGCTTTGAATTTTACCACAAATGCGATAACACGTGCATGGAAAGTGTCAAAAATGGGACTTATGACTACCCAAAATACTCAGAGGAAGCAAAATTAAACAGAGAAGAAATAGATGGGGTAAAGCTGGAATCAACAAGGATTTACCAGATTTTGGCGATCTATTCAACTGTCGCCAGTTCATTGGTACTGGTAGTCTCCCTGGGGGCAATCAGTTTCTGGATGTGCTCTAATGGGTCTCTACAGTGTAGAATATGTATTTAA

>LC638205.1Influenza A virus H1N101701

ATGAAGGCAATACTAGTAGTTCTGCTATATACATTTGCAACCGCAAATGCAGACACATTATGTATAGGTTATCATGCGAACAATTCAACAGACACTGTAGACACAATACTAGAAAAGAATGTAACAGTAACACACTCTGTTAACCTTCTAGAAGACAAGCATAACGGGAAACTATGCAAACTAAGAGGGGTAGCCCCATTGCATTTGGGTAAATGTAACATTGCTGGCTGGATCCTGGGAAATCCAGAGTGTGAATCACTCTCCACAGCAAGCTCATGGTCCTACATTGTGGAAACATCTAGTTCAGACAATGGAACGTGTTACCCAGGAGATTTCATCGATTATGAGGAGCTAAGAGAGCAATTGAGCTCAGTGTCATCATTTGAACGGTTTGAGATATTCCCCAAGACAAGTTCATGGCCCAATCATGACTCGAACAAAGGTGTAACGGCAGCATGTCCTCATGCTGGAGCAAAAAGCTTCTACAAAAATTTAATATGGCTAGTTAAAAAAGGAAATTCATACCCAAAGCTCAGCAAATCCTACATTAATGATAAAGGGAAAGAAGTCCTCGTGCTATGGGGCATTCACCATCCATCTACTAGTGCTGACCAACAAAGTCTCTATCAGAATGCAGATGCATATGTTTTTGTGGGGACATCAAGATACAGCAAGAAGTTCAAGCCGGAAATAGCAATAAGACCCAAAGTGAGGGATCAAGAAGGGAGAATGAACTATTACTGGACACTAGTAGAGCCGGGAGACAAAATAACATTCGAAGCAACTGGAAATCTAGTGGTACCGAGATATGCATTCGCAATGGAAAGAAATGCTGGATCTGGTATTATCATTTCAGATACACCAGTCCACGATTGCAATACAACTTGTCAGACACCCAAGGGTGCTATAAACACCAGCCTCCCATTTCAGAATATACATCCGATCACAATTGGAAAATGTCCAAAATATGTAAAAAGCACAAAATTGAGACTGGCCACAGGATTGAGGAATGTCCCGTCTATTCAATCTAGAGGCCTATTTGGGGCCATTGCCGGTTTCATTGAAGGGGGGTGGACAGGGATGGTAGATGGATGGTACGGTTATCACCATCAAAATGAGCAGGGGTCAGGATATGCAGCCGACCTGAAGAGCACACAGAATGCCATTGACGTGATTACTAACAAAGTAAATTCTGTTATTGAAAAGATGAATACACAGTTCACAGCAGTAGGTAAAGAGTTCAACCACCTGGAAAAAAGAATAGAGAATTTAAATAAAAAGGTTGATGATGGTTTCCTGGACATTTGGACTTACAATGCCGAACTGTTGGTTCTATTGGAAAATGAAAGAACTTTGGACTACCACGATTCAAATGTGAAGAACTTATATGAAAAGGTAAGAAGCCAGTTAAAAAACAATGCCAAGGAAATTGGAAACGGCTGCTTTGAATTTTACCACAAATGCGATAACACGTGCATGGAAAGTGTCAAAAATGGGACTTATGACTACCCAAAATACTCAGAGGAAGCAAAATTAAACAGAGAAGAAATAGATGGGGTAAAGCTGGAATCAACAAGGATTTACCAGATTTTGGCGATCTATTCAACTGCCGCCAGTTCATTGGTACTGGTAGTCTCCCTGGGGGCAATCAGTTTCTGGATGTGCTCTAATGGGTCTCTACAGTGTAGAATATGTATTTAA

>LC638204.1Influenza A virus H1N101701

ATGAAGGCAATACTAGTAGTTCTGCTATATACATTTGCAACCGCAAATGCAGACACATTATGTATAGGTTATCATGCGAACAATTCAACAGACACTGTAGACACAATACTAGAAAAGAATGTAACAGTAACACACTCTGTTAACCTTCTAGAAGACAAGCATAACGGGAAACTATGCAAACTAAGAGGGGTAGCCCCATTGCATTTGGGTAAATGTAACATTGCTGGCTGGATCCTGGGAAATCCAGAGTGTGAATCACTCTCCACAGCAAGCTCATGGTCCTACATTGTGGAAACATCTAGTTCAGACAATGGAACGTGTTACCCAGGAGATTTCATCGATTATGAGGAGCTAAGAGAGCAATTGAGCTCAGTGTCATCATTTGAAAGGTTTGAGATATTCCCCAAGACAAGTTCATGGCCCAATCATGACTCGAACAAAGGTGTAACGGCAGCATGTCCTCATGCTGGAGCAAAAAGCTTCTACAAAAATTTAATATGGCTAGTTAAAAAAGGAAATTCATACCCAAAGCTCAGCAAATCCTACATTAATGATAAAGGGAAAGAAGTCCTCGTGCTATGGGGCATTCACCATCCATCTACTAGTGCTGACCAACAAAGTCTCTATCAGAATGCAGATGCATATGTTTTTGTGGGGACATCAAGATACAGCAAGAAGTTCAAGCCGGAAATAGCAATAAGACCCAAAGTGAGGGATCAAGAAGGGAGAATGAACTATTACTGGACACTAGTAGAGCCGGGAGACAAAATAACATTCGAAGCAACTGGAAATCTAGTGGTACCGAGATATGCATTCGCAATGGAAAGAAATGCTGGATCTGGTATTATCATTTCAGATACACCAGTCCACGATTGCAATACAACTTGTCAGACACCCAAGGGTGCTATAAACACCAGCCTCCCATTTCAGAATATACATCCGATCACAATTGGAAAATGTCCAAAATATGTAAAAAGCACAAAATTGAGACTGGCCACAGGATTGAGGAATGTCCCGTCTATTCAATCTAGAGGCCTATTTGGGGCCATTGCCGGTTTCATTGAAGGGGGGTGGACAGGGATGGTAGATGGATGGTACGGTTATCACCATCAAAATGAGCAGGGGTCAGGATATGCAGCCGACCTGAAGAGCACACAGAATGCCATTGACGAGATTACTAACAAGGTAAATTCTGTTATTGAAAAGATGAATACACAGTTCACAGCAGTAGGTAAAGAGTTCAACCACCTGGAAAAAAGAATAGAGAATTTAAATAAAAAGGTTGATGATGGTTTCCTGGACATTTGGACTTACAATGCCGAACTGTTGGTTCTATTGGAAAATGAAAGAACTTTGGACTACCACGATTCAAATGTGAAGAACTTATATGAAAAGGTAAGAAGCCAGTTAAAAAACAATGCCAAGGAAATTGGAAACGGCTGCTTTGAATTTTACCACAAATGCGATAACACGTGCATGGAAAGTGTCAAAAATGGGACTTATGACTACCCAAAATACTCAGAGGAAGCAAAATTAAACAGAGAAGAAATAGATGGGGTAAAGCTGGAATCAACAAGGATTTACCAGATTTTGGCGATCTATTCAACTGTCGCCAGTTCATTGGTACTGGTAGTCTCCCTGGGGGCAATCAGTTTCTGGATGTGCTCTAATGGGTCTCTACAGTGTAGAATATGTATTTAA

>LC638203.1Influenza A virus H1N101701

ATGAAGGCAATACTAGTAGTTCTGCTATATACATTTGCAACCGCAAATGCAGACACATTATGTATAGGTTATCATGCGAACAATTCAACAGACACTGTAGACACAATACTAGAAAAGAATGTAACAGTAACACACTCTGTTAACCTTCTAGAAGACAAGCATAACGGGAAACTATGCAAACTAAGAGGGGTAGCCCCATTGCATTTGGGTAAATGTAACATTGCTGGCTGGATCCTGGGAAATCCAGAGTGTGAATCACTCTCCACAGCAAGCTCATGGTCCTACATTGTGGAAACATCTAGTTCAGACAATGGAACGTGTTACCCAGGAGATTTCATCGATTATGAGGAGCTAAGAGAGCAATTGAGCTCAGTGTCATCATTTGAACGGTTTGAGATATTCCCCAAGACAAGTTCATGGCCCAATCATGACTCGAACAAAGGTGTAACGGCAGCATGTCCTCATGCTGGAGCAAAAAGCTTCTACAAAAATTTAATATGGCTAGTTAAAAAAGGAAATTCATACCCAAAGCTCAGCAAATCCTACATTAATGATAAAGGGAAAGAAGTCCTCGTGCTATGGGGCATTCACCATCCATCTACTAGTGCTGACCAACAAAGTCTCTATCAGAATGCAGATGCATATGTTTTTGTGGGGACATCAAGATACAGCAAGAAGTTCAAGCCGGAAATAGCAATAAGACCCAAAGTGAGGGATCAAGAAGGGAGAATGAACTATTACTGGACACTAGTAGAGCCGGGAGACAAAATAACATTCGAAGCAACTGGAAATCTAGTGGTACCGAGATATGCATTCGCAATGGAAAGAAATGCTGGATCTGGTATTATCATTTCAGATACACCAGTCCACGATTGCAATACAACTTGTCAGACACCCAAGGGTGCTATAAACACCAGCCTCCCATTTCAGAATATACATCCGATCACAATTGGAAAATGTCCAAAATATGTAAAAAGCACAAAATTGAGACTGGCCACAGGATTGAGGAATGTCCCGTCTATTCAATCTAGAGGCCTATTTGGGGCCATTGCCGGTTTCATTGAAGGGGGGTGGACAGGGATGGTAGATGGATGGTACGGTTATCACCATCAAAATGAGCAGGGGTCAGGATATGCAGCCGACCTGAAGAGCACACAGAATGCCATTGACGTGATTACTAACAAAGTAAATTCTGTTATTGAAAAGATGAATACACAGTTCACAGCAGTAGGTAAAGAGTTCAACCACCTGGAAAAAAGAATAGAGAATTTAAATAAAAAGGTTGATGATGGTTTCCTGGACATTTGGACTTACAATGCCGAACTGTTGGTTCTATTGGAAAATGAAAGAACTTTGGACTACCACGATTCAAATGTGAAGAACTTATATGAAAAGGTAAGAAGCCAGTTAAAAAACAATGCCAAGGAAATTGGAAACGGCTGCTTTGAATTTTACCACAAATGCGATAACACGTGCATGGAAAGTGTCAAAAATGGGACTTATGACTACCCAAAATACTCAGAGGAAGCAAAATTAAACAGAGAAGAAATAGATGGGGTAAAGCTGGAATCAACAAGGATTTACCAGATTTTGGCGATCTATTCAACTGCCGCCAGTTCATTGGTACTGGTAGTCTCCCTGGGGGCAATCAGTTTCTGGATGTGCTCTAATGGGTCTCTACAGTGTAGAATATGTATTTAA

>LC638202.1Influenza A virus H1N101701

ATGAAGGCAATACTAGTAGTTCTGCTATATACATTTGCAACCGCAAATGCAGACACATTATGTATAGGTTATCATGCGAACAATTCAACAGACACTGTAGACACAGTACTAGAAAAGAATGTAACAGTAACACACTCTGTTAACCTTCTAGAAGACAAGCATAACGGGAAACTATGCAAACTAAGAGGGGTAGCCCCATTGCATTTGGGTAAATGTAACATTGCTGGCTGGATCCTGGGAAATCCAGAGTGTGAATCACTCTCCACAGCAAGCTCATGGTCCTACATTGTGGAAACATCTAGTTCAGACAATGGAACGTGTTACCCAGGAGATTTCATCGATTATGAGGAGCTAAGAGAGCAATTGAGCTCAGTGTCATCATTTGAAAGGTTTGAGATATTCCCCAAGACAAGTTCATGGCCCAATCATGACTCAAACAAAGGTGTAACGGCAGCATGTCCTCATGCTGGAGCAAAAAGCTTCTACAAAAATTTAATATGGCTAGTTAAAAAAGGAAATTCATACCCAAAGCTCAGCAAATCCTACATTAATGATAAAGGGAAAGAAGTCCTCGTGCTATGGGGCATTCACCATCCATCTACTAGTGCTGACCAACAAAGCCTCTATCAGAATGCAGATGCATATGTTTTTGTGGGGACATCAAGATACAGCAAGAAGTTCAAGCCGGAAATAGCAATAAGACCCAAAGTGAGGGATCAAGAAGGGAGAATGAACTATTACTGGACACTAGTAGAGCCGGGAGACAAAATAACATTCGAAGCAACTGGAAATCTAATGGTACCGAGATATGCATTCGCAATGGAAAGAAATGCTGGATCTGGTATTATCATTTCAGATACACCAGTCCACGATTGCAATACAACTTGTCAGACACCCAAGGGTGCTATAAACACCAGCCTCCCATTTCAGAATATACATCCGATCACAATTGGAAAATGTCCAAAATATGTAAAAAGCACAAAATTGAGACTGGCCACAGGATTGAGGAATGTCCCTTCTATTCAATCTAGAGGCCTATTTGGGGCCATTGCCGGTTTCATTGAAGGGGGGTGGACAGGGATGGTAGATGGATGGTACGGTTATCACCATCAAAATGAGCAGGGGTCAGGATATGCAGCCGACCTGAAGAGCACACAGAATGCCATTGACGAGATTACTAACAAAGTAAATTCTGTTATTGAAAAGATGAATACACAGTTCACAGCAGTAGGTAAAGAGTTCAACCACCTGGAAAAAAGAATAGAGAATTTAAATAAAAAAGTTGATGATGGTTTCCTGGACATTTGGACTTACAATGCCGAACTGTTGGTTCTATTGGAAAATGAAAGAACTTTGGACTACCACGATTCAAATGTGAAGAACTTATATGAAAAGGTAAGAAGCCAGTTAAAAAACAATGCCAAGGAAATTGGAAACGGCTGCTTTGAATTTTACCACAAATGCGATAACACGTGCATGGAAAGTGTCAAAAATGGGACTTATGACTACCCAAAATACTCAGAGGAAGCAAAATTAAACAGAGAAGAAATAGATGGGGTAAAGCTGGAATCAACAAGGATTTACCAGATCTTGGCGATCTATTCAACTGTCGCCAGTTCATTGGTACTGGTAGTCTCCCTGGGGGCAATCAGTTTCTGGATGTGCTCTAATGGGTCTCTACAGTGTAGAATATGTATTTAA

>LC638201.1Influenza A virus H1N101701

ATGAAGGCAATACTAGTAGTTCTGCTATATACATTTGCAACCGCAAATGCAGACACATTATGTATAGGTTATCATGCGAACAATTCAACAGACACTGTAGACACAGTACTAGAAAAGAATGTAACAGTAACACACTCTGTTAACCTTCTAGAAGACAAGCATAACGGGAAACTATGCAAACTAAGAGGGGTAGCCCCATTGCATTTGGGTAAATGTAACATTGCTGGCTGGATCCTGGGAAATCCAGAGTGTGAATCACTCTCCACAGCAAGCTCATGGTCCTACATTGTGGAAACATCTAGTTCAGACAATGGAACGTGTTACCCAGGAGATTTCATCGATTATGAGGAGCTAAGAGAGCAATTGAGCTCAGTGTCATCATTTGAAAGGTTTGAGATATTCCCCAAGACAAGTTCATGGCCCAATCATGACTCGAACAAAGGTGTAACGGCAGCATGTCCTCATGCTGGAGCAAAAAGCTTCTACAAAAATTTAATATGGCTAGTTAAAAAAGGAAATTCATACCCAAAGCTCAGCAAATCCTACATTAATGATAAAGGGAAAGAAGTCCTCGTGCTATGGGGCATTCACCATCCATCTACTAGTGCTGACCAACAAAGTCTCTATCAGAATGCAGATACATATGTTTTTGTGGGGACATCAAGATACAGCAAGAAGTTCAAGCCGGAAATAGCAATAAGACCCAAAGTGAGGGATCAAGAAGGGAGAATGAACTATTACTGGACACTAGTAGAGCCGGGAGACAAAATAACATTCGAAGCAACTGGAAATCTAGTGGTACCGAGATATGCATTCGCAATGGAAAGAAATGCTGGATCTGGTATTATCATTTCAGATACACCAGTCCACGATTGCAATACAACTTGTCAGACACCCAAGGGTGCTATAAACACCAGCCTCCCATTTCAGAATATACATCCGATCACAATTGGAAAATGTCCAAAATATGTAAAAAGCACAAAATTGAGACTGGCCACAGGATTGAGGAATGTCCCGTCTATTCAATCTAGAGGCCTATTTGGGGCCATTGCCGGTTTCATTGAAGGGGGGTGGACAGGGATGGTAGATGGATGGTACGGTTATCACCATCAAAATGAGCAGGGGTCAGGATATGCAGCCGACCTGAAGAGCACACAGAATGCCATTGACGAGATTACTAACAAAGTAAATTCTGTTATTGAAAAGATGAATACACAGTTCACAGCAGTAGGTAAAGAGTTCAACCACCTGGAAAAAAGAATAGAGAATTTAAATAAAAAAGTTGATGATGGTTTCCTGGACATTTGGACTTACAATGCCGAACTGTTGGTTCTATTGGAAAATGAAAGAACTTTGGACTACCACGATTCAAATGTGAAGAACTTATATGAAAAGGTAAGAAGCCAGTTAAAAAACAATGCCAAGGAAATTGGAAACGGCTGCTTTGAATTTTACCACAAATGCGATAACACGTGCATGGAAAGTGTCAAAAATGGGACTTATGACTACCCAAAATACTCAGAGGAAGCAAAATTAAACAGAGAAGAAATAGATGGGGTAAAGCTGGAATCAACAAGGATTTACCAGATTTTGGCGATCTATTCAACTGTCGCCAGTTCATTGGTACTGGTAGTCTCCCTGGGGGCAATCAGTTTCTGGATGTGCTCTAATGGGTCTCTACAGTGTAGAATATGTATTTAA

>LC638200.1Influenza A virus H1N101701

ATGAAGGCAATACTAGTAGTTCTGCTATATACATTTGCAACCGCAAATGCAGACACATTATGTATAGGTTATCATGCGAACAATTCAACAGACACTGTAGACACAGTACTAGAAAAGAATGTAACAGTAACACACTCTGTTAACCTCCTAGAAGACAAGCATAACGGGAAACTATGCAAACTAAGAGGGGTAGCCCCATTGCATTTGGGTAAATGTAACATTGCTGGCTGGATCCTGGGAAATCCAGAGTGTGAATCACTCTCCACAGCAAGCTCATGGTCCTACATTGTGGAAACATCTAGTTCAGACAATGGAACGTGTTACCCAGGAGATTTCATCGATTATGAGGAGCTAAGAGAGCAATTGAGCTCAGTGTCATCATTTGAAAGGTTTGAGATATTCCCCAAGACAAGTTCATGGCCCAATCATGACTCGAACAAAGGTGTAACGGCAGCATGTCCTCATGCTGGAGCAAAAAGCTTCTACAAAAATTTAATATGGCTAGTTAAAAAAGGAAATTCATACCCAAAGCTCAGCAAATCCTACATTAATGATAAAGGGAAAGAAGTCCTCGTGCTATGGGGCATTCACCATCCATCTACTAGTGCTGACCAACAAAGTCTCTATCAGAATGCAGATACATATGTTTTTGTGGGGACATCAAGATACAGCAAGAAGTTCAAGCCGGAAATAGCAATAAGACCCAAAGTGAGGGATCAAGAAGGGAGAATGAACTATTACTGGACACTAGTAGAGCCGGGAGACAAAATAACATTCGAAGCAACTGGAAATCTAGTGGTACCGAGATATGCATTCGCAATGGAAAGAAATGCTGGATCTGGTATTATCATTTCAGATACACCAGTCCACGATTGCAATACAACTTGTCAGACACCCAAGGGTGCTATAAACACCAGCCTCCCATTTCAGAATATACATCCGATCACAATTGGAAAATGTCCAAAATATGTAAAAAGCACAAAATTGAGACTGGCCACAGGATTGAGGAATATCCCGTCTATTCAATCTAGAGGCCTATTTGGGGCTATTGCCGGTTTCATTGAAGGGGGGTGGACAGGGATGGTAGATGGATGGTACGGTTATCACCATCAAAATGAGCAGGGGTCAGGATATGCAGCCGACCTGAAGAGCACACAGAATGCCATTGACGAGATTACTAACAAAGTAAATTCTGTTATTGAAAAGATGAATACACAGTTCACAGCAGTAGGTAAAGAGTTCAACCACCTGGAAAAAAGAATAGAGAATTTAAATAAAAAAGTTGATGATGGTTTCCTGGACATTTGGACTTACAATGCCGAACTGTTGGTTCTATTGGAAAATGAAAGAACTTTGGACTACCACGATTCAAATGTGAAGAACTTATATGAAAAGGTAAGAAGCCAGTTAAAAAACAATGCCAAGGAAATTGGAAACGGCTGCTTTGAATTTTACCACAAATGCGATAACACGTGCATGGAAAGTGTCAAAAATGGGACTTATGACTACCCAAAATACTCAGAGGAAGCAAAATTAAACAGAGAAGAAATAGATGGGGTAAAGCTGGAATCAACAAGGATTTACCAGATTTTGGCGATCTATTCAACTGTCGCCAGTTCATTGGTACTGGTAGTCTCCCTGGGGGCAATCAGTTTCTGGATGTGCTCTAATGGGTCTCTACAGTGTAGAATATGTATTTAA

>LC638199.1Influenza A virus H1N101701

ATGAAGGCAATACTAGTAGTTCTGCTATATACATTTGCAACCGCAAATGCAGACACATTATGTATAGGTTATCATGCGAACAATTCAACAGACACTGTAGACACAGTACTAGAAAAGAATGTAACAGTAACACACTCTGTTAACCTTCTAGAAGACAAGCATAACGGGAAACTATGCAAACTAAGAGGGGTAGCCCCATTGCATTTGGGTAAATGTAACATTGCTGGCTGGATCCTGGGAAATCCAGAGTGTGAATCACTCTCCACAGCAAGCTCATGGTCCTACATTGTGGAAACATCTAGTTCAGACAATGGAACGTGTTACCCAGGAGATTTCATCGATTATGAGGAGCTAAGAGAGCAATTGAGCTCAGTGTCATCATTTGAAAGGTTTGAGATATTCCCCAAGACAAGTTCATGGCCCAATCATGACTCGAACAAAGGTGTAACGGCAGCATGTCCTCATGCTGGAGCAAAAAGCTTCTACAAAAATTTAATATGGCTAGTTAAAAAAGGAAATTCATACCCAAAGCTCAGCAAATCCTACATTAATGATAAAGGGAAAGAAGTCCTCGTGCTATGGGGCATTCACCATCCATCTACTAGTGCTGACCAACAAAGTCTCTATCAGAATGCAGATGCATATGTTTTTGTGGGGACATCAAGATACAGCAAGAAGTTCAAGCCGGAAATAGCAATAAGACCCAAAGTGAGGGATCAAGAAGGGAGAATGAACTATTACTGGACACTAGTAGAGCCGGGAGACAAAATAACATTCGAAGCAACTGGAAATCTAGTGGTACCGAGATACGCATTCGCAATGGAAAGAAATGCTGGATCTGGTATTATCATTTCAGATACACCAGTCCATGATTGCAATACAACTTGTCAGACACCCAAGGGTGCTATAAACACCAGCCTCCCATTTCAGAATATACATCCGATCACAATTGGAAAATGTCCAAAATATGTAAAAAGCACAAAATTGAGACTGGCCACAGGATTGAGGAATGTCCCGTCTATTCAATCTAGAGGCCTATTTGGGGCCATTGCCGGCTTCATTGAAGGGGGGTGGACAGGGATGGTAGATGGATGGTACGGTTATCACCATCAAAATGAGCAGGGGTCAGGATATGCAGCCGACCTGAAGAGCACACAGAATGCCATTGACAAGATTACTAACAAAGTAAATTCTGTTATTGAAAAGATGAATACACAGTTCACAGCAGTAGGTAAAGAGTTCAACCACCTGGAAAAAAGAATAGAGAATTTAAATAAAAAAGTTGATGATGGTTTCCTGGACATTTGGACTTACAATGCCGAACTGTTGGTTCTATTGGAAAATGAAAGAACTTTGGACTACCACGATTCAAATGTGAAGAACTTATATGAAAAGGTAAGAAGCCAGTTAAAAAACAATGCCAAGGAAATTGGAAACGGCTGCTTTGAATTTTACCACAAATGCGATAACACGTGCATGGAAAGTGTCAAAAATGGGACTTATGACTACCCAAAATACTCAGAGGAAGCAAAATTAAACAGAGAAGAAATAGATGGGGTAAAGCTGGAATCAACAAGGATTTACCAGATTTTGGCGATCTATTCAACTGTCGCCAGTTCATTGGTACTGGTAGTCTCCCTGGGGGCAATCAGTTTCTGGATGTGCTCTAATGGGTCTCTACAGTGTAGAATATGTATTTAA

>LC638198.1Influenza A virus H1N101701

ATGAAGGCAATACTAGTAGTTCTGCTATATACATTTGCAACCGCAAATGCAGACACATTATGTATAGGTTATCATGCGAACAATTCAACAGACACTGTAGACACAATACTAGAAAAGAATGTAACAGTAACACACTCTGTTAACCTTCTAGAAGACAAGCATAACGGGAAACTATGCAAACTAAGAGGGGTAGCCCCATTGCATTTGGGTAAATGTAACATTGCTGGCTGGATCCTGGGAAATCCAGAGTGTGAATCACTCTCCACAGCAAGCTCATGGTCCTACATTGTGGAAACATCTAGTTCAGACAATGGAACGTGTTACCCAGGAGATTTCATCGATTATGAGGAGCTAAGAGAGCAATTGAGCTCAGTGTCATCATTTGAACGGTTTGAGATATTCCCCAAGACAAGTTCATGGCCCAATCATGACTCGAACAAAGGTGTAACGGCAGCATGTCCTCATGCTGGAGCAAAAAGCTTCTACAAAAATTTAATATGGCTAGTTAAAAAAGGAAATTCATACCCAAAGCTCAGCAAATCCTACATTAATGATAAAGGGAAAGAAGTCCTCGTGCTATGGGGCATTCACCATCCATCTACTAGTGCTGACCAACAAAGTCTCTATCAGAATGCAGATGCATATGTTTTTGTGGGGACATCAAGATACAGCAAGAAGTTCAAGCCGGAAATAGCAATAAGACCCAAAGTGAGGGATCAAGAAGGGAGAATGAACTATTACTGGACACTAGTAGAGCCGGGAGACAAAATAACATTCGAAGCAACTGGAAATCTAGTGGTACCGAGATATGCATTCGCAATGGAAAGAAATGCTGGATCTGGTATTATCATTTCAGATACACCAGTCCACGATTGCAATACAACTTGTCAGACACCCAAGGGTGCTATAAACACCAGCCTCCCATTTCAGAATATACATCCGATCACAATTGGAAAATGTCCAAAATATGTAAAAAGCACAAAATTGAGACTGGCCACAGGATTGAGGAATGTCCCGTCTATTCAATCTAGAGGCCTATTTGGGGCCATTGCCGGTTTCATTGAAGGGGGGTGGACAGGGATGGTAGATGGATGGTACGGTTATCACCATCAAAATGAGCAGGGGTCAGGATATGCAGCCGACCTGAAGAGCACACAGAATGCCATTGACGAGATTACTAACAAAGTAAATTCTGTTATTGAAAAGATGAATACACAGTTCACAGCAGTAGGTAAAGAGTTCAACCACCTGGAAAAAAGAATAGAGAATTTAAATAAAAAGGTTGATGATGGTTTCCTGGACATTTGGACTTACAATGCCGAACTGTTGGTTCTATTGGAAAATGAAAGAACTTTGGACTACCACGATTCAAATGTGAAGAACTTATATGAAAAGGTAAGAAGCCAGTTAAAAAACAATGCCAAGGAAATTGGAAACGGCTGCTTTGAATTTTACCACAAATGCGATAACACGTGCATGGAAAGTGTCAAAAATGGGACTTATGACTACCCAAAATACTCAGAGGAAGCAAAATTAAACAGAGAAGAAATAGATGGGGTAAAGCTGGAATCAACAAGGATTTACCAGATTTTGGCGATCTATTCAACTGCCGCCAGTTCATTGGTACTGGTAGTCTCCCTGGGGGCAATCAGTTTCTGGATGTGCTCTAATGGGTCTCTACAGTGTAGAATATGTATTTAA

>LC638197.1Influenza A virus H1N101701

ATGAAGGCAATACTAGTAGTTCTGCTATATACATTTGCAACCGCAAATGCAGACACATTATGTATAGGTTATCATGCGAACAATTCAACTGACACTGTAGACACAGTACTAGAAAAGAATGTAACAGTAACACACTCTGTTAACCTTCTAGAAGACAAGCATAACGGGAAACTATGCAAACTAAGAGGGGTAGCCCCATTGCATTTGGGTAAATGTAACATTGCTGGCTGGATCCTGGGAAATCCAGAGTGTGAATCACTCTCCACAGCAAGCTCATGGTCCTACATTGTGGAAACATCTAGTTCAGACAATGGAACGTGTTACCCAGGAGATTTCATCGATTATGAGGAGCTAAGAGAGCAATTGAGCTCAGTGTCATCATTTGAAAGGTTTGAGATATTCCCCAAGACAAGTTCATGGCCCAATCATGACTCGAACAAAGGTGTGACGGCAGCATGTCCTCATGCTGGAGCAAAAAGCTTCTACAAAAATTTAATATGGCTAGTTAAAAAAGGAAATTCATACCCAAAGCTCAGCAAATCCTACATTAATGATAAAGGGAGAGAAGTCCTCGTGCTATGGGGCATTCACCATCCATCTACTAGTGCTGACCAACAAAGTCTCTATCAGAATGCAGATACATATGTTTTTGTGGGGACATCAAGATACAGCAAGAAGTTCAAGCCGGAAATAGCAATAAGACCCAAAGTGAGGGATCAAGAAGGGAGAATGAACTATTACTGGACACTAGTAGAGCCGGGAGACAAAATAACATTCGAAGCAACTGGAAATCTAGTGGTACCGAGATATGCATTCGCAATGGAAAGAAATGCTGGATCTGGTATTATCATTTCAGATACACCAGTCCACGATTGCAATACAACTTGTCAGACACCCAAGGGTGCTATAAACACCAGCCTCCCATTTCAGAATATACATCCGATCACAATTGGAAAATGTCCAAAATATGTAAAAAGCACAAAATTGAGACTGGCCACAGGATTGAGGAATGTCCCGTCTATTCAATCTAGAGGCCTATTTGGGGCCATTGCCGGTTTCATTGAAGGGGGGTGGACAGGGATGGTAGATGGATGGTACGGTTATCACCATCAAAATGAGCAGGGGTCAGGATATGCAGCCGACCTGAAGAGCACACAGAATGCCATTGACGAGATTACTAACAAAGTAAATTCTGTTATTGAAAAGATGAATACACAGTTCACAGCAGTAGGTAAAGAGTTCAACCACCTGGAAAAAAGAATAGAGAATTTAAATAAAAAAGTTGATGATGGTTTCCTGGACATTTGGACTTACAATGCCGAACTGTTGGTTCTATTGGAAAATGAAAGAACTTTGGACTACCACGATTCAAATGTGAAGAACTTATATGAAAAGGTAAGAAGCCAGTTAAAAAACAATGCCAAGGAAATTGGAAACGGCTGCTTTGAATTTTACCACAAATGCGATAACACGTGCATGGAAAGTGTCAAAAATGGGACTTATGACTACCCAAAATACTCAGAGGAAGCAAAATTAAACAGAGAAGAAATAGATGGGGTAAAGCTGGAATCAACAAGGATTTACCAGATTTTGGCGATCTATTCAACTGTCGCCAGTTCATTGGTACTGGTAGTCTCCCTGGGGGCAATCAGTTTCTGGATGTGCTCTAATGGGTCTCTACAGTGTAGAATATGTATTTAA

>LC638196.1Influenza A virus H1N101701

ATGAAGGCAATACTAGTAGTTCTGCTATATACATTTGCAACCGCAAATGCAGACACATTATGTATAGGTTATCATGCGAACAATTCAACAGACACTGTAGACACAGTACTAGAAAAGAATGTAACAGTAACACACTCTGTTAACCTTCTAGAAGACAAGCATAACGGGAAACTATGCAAACTAAGAGGGGTAGCCCCATTGCATTTGGGTAAATGTAACATTGCTGGCTGGATCCTGGGAAATCCAGAGTGTGAATCACTCTCCACAGCAAGCTCATGGTCCTACATTGTGGAAACATCTAGTTCAGACAATGGAACGTGTTACCCAGGAGATTTCATCGATTATGAGGAGCTAAGAGAGCAATTGAGCTCAGTGTCATCATTTGAAAGGTTTGAGATATTCCCCAAGACAAGTTCATGGCCCAATCATGACTCAAACAAAGGTGTAACGGCAGCATGTCCTCATGCTGGAGCAAAAAGCTTCTACAAAAATTTAATATGGCTAGTTAAAAAAGGAAATTCATACCCAAAGCTCAGCAAATCCTACATTAATGATAAAGGGAAAGAAGTCCTCGTGCTATGGGGCATTCACCATCCATCTACTAGTGCTGACCAACAAAGCCTCTATCAGAATGCAGATGCATATGTTTTTGTGGGGACATCAAGATACAGCAAGAAGTTCAAGCCGGAAATAGCAATAAGACCCAAAGTGAGGGATCAAGAAGGGAGAATGAACTATTACTGGACACTAGTAGAGCCGGGAGACAAAATAACATTCGAAGCAACTGGAAATCTAATGGTACCGAGATATGCATTCGCAATGGAAAGAAATGCTGGATCTGGTATTATCATTTCAGATACACCAGTCCACGATTGCAATACAACTTGTCAGACACCCAAGGGTGCTATAAACACCAGCCTCCCATTTCAGAATATACATCCGATCACAATTGGAAAATGTCCAAAATATGTAAAAAGCACAAAATTGAGACTGGCCACAGGATTGAGGAATGTCCCTTCTATTCAATCTAGAGGCCTATTTGGGGCCATTGCCGGTTTCATTGAAGGGGGGTGGACAGGGATGGTAGATGGATGGTACGGTTATCACCATCAAAATGAGCAGGGGTCAGGATATGCAGCCGACCTGAAGAGCACACAGAATGCCATTGACGAGATTACTAACAAAGTAAATTCTGTTATTGAAAAGATGAATACACAGTTCACAGCAGTAGGTAAAGAGTTCAACCACCTGGAAAAAAGAATAGAGAATTTAAATAAAAAAGTTGATGATGGTTTCCTGGACATTTGGACTTACAATGCCGAACTGTTGGTTCTATTGGAAAATGAAAGAACTTTGGACTACCACGATTCAAATGTGAAGAACTTATATGAAAAGGTAAGAAGCCAGTTAAAAAACAATGCCAAGGAAATTGGAAACGGCTGCTTTGAATTTTACCACAAATGCGATAACACGTGCATGGAAAGTGTCAAAAATGGGACTTATGACTACCCAAAATACTCAGAGGAAGCAAAATTAAACAGAGAAGAAATAGATGGGGTAAAGCTGGAATCAACAAGGATTTACCAGATCTTGGCGATCTATTCAACTGTCGCCAGTTCATTGGTACTGGTAGTCTCCCTGGGGGCAATCAGTTTCTGGATGTGCTCTAATGGGTCTCTACAGTGTAGAATATGTATTTAA

>LC638195.1Influenza A virus H1N101701

ATGAAGGCAATACTAGTAGTTCTGCTATATACATTTGCAACCGCAAATGCAGACACATTATGTATAGGTTATCATGCGAACAATTCAACAGACACTGTAGACACAGTACTAGAAAAGAATGTAACAGTAACACACTCTGTTAACCTTCTAGAAGACAAGCATAACGGGAAACTATGCAAACTAAGAGGGGTAGCCCCATTGCATTTGGGTAAATGTAACATTGCTGGCTGGATCCTGGGAAATCCAGAGTGTGAATCACTCTCCACAGCAAGCTCATGGTCCTACATTGTGGAAACATCTAGTTCAGACAATGGAACGTGTTACCCAGGAGATTTCATCGATTATGAGGAGCTAAGAGAGCAATTGAGCTCAGTGTCATCATTTGAAAGGTTTGAGATATTCCCCAAGACAATTTCATGGCCCAATCATGACTCGAACAAAGGTGTAACGGCAGCATGTCCTCATGCTGGAGCAAAAAGCTTCTACAAAAATTTAATATGGCTAGTTAAAAAAGGAAATTCATACCCAAAGCTCAGCAAATCCTACATTAATGATAAAGGGAAAGAAGTCCTCGTGCTATGGGGCATTCACCATCCATCTACTAGTGCTGACCAACAAAGTCTCTATCAGAATGCAGATGCATATGTTTTTGTGGGGACATCAAGATACAGCAAGAAGTTCAAGCCGGAAATAGCAATAAGACCCAAAGTGAGGGATCAAGAAGGGAGAATGAACTATTACTGGACACTAGTAGAGCCGGGAGACAAAATAACATTCGAAGCAACTGGAAATCTAGTGGTACCGAGATATGCATTCGCAATGGAAAGAAATGCTGGATCTGGTATTATCATTTCAGATACACCAGTCCACGATTGCAATACAACTTGTCAGACACCCAAGGGTGCTATAAACACCAGCCTCCCATTTCAGAATATACATCCGATCACAATTGGAAAATGTTCAAAATATGTAAAAAGCACAAAATTGAGACTGGCCACAGGATTGAGGAATGTCCCGTCTATTCAATCTAGAGGCCTATTTGGGGCTATTGCCGGTTTCATTGAAGGGGGGTGGACAGGGATGGTAGATGGATGGTACGGTTATCACCATCAAAATGAGCAGGGGTCAGGATATGCAGCCGACCTGAAGAGCACACAAAATGCCATTGACGAGATTACTAACAAAGTAAATTCTGTTATTGAAAAGATGAATACACAGTTCACAGCAGTAGGTAAAGAGTTCAACCACCTGGAAAAAAGAATAGAGAATTTAAATAAAAAGGTTGATGATGGTTTCCTGGACATTTGGACTTACAATGCCGAACTGTTGGTTCTATTGGAAAATGAAAGAACTTTGGACTACCACGATTCAAATGTGAAGAACTTATATGAAAAGGTAAGAAGCCAGTTAAAAAACAATGCCAAGGAAATTGGAAACGGCTGCTTTGAATTTTACCACAAATGCGATAACACGTGCATGGAAAGTATCAAAAATGGGACTTATGACTACCCAAAATACTCAGAGGAAGCAAAATTAAACAGAGAAGAAATAGATGGGGTAAAGCTGGAATCAACAAGGATTTACCAGATTTTGGCGATCTATTCAACTGTCGCCAGTTCATTGGTACTGGTAGTCTCCCTGGGGGCAATCAGTTTCTGGATGTGCTCTAATGGGTCTCTACAGTGTAGAATATGTATTTAA

>LC638194.1Influenza A virus H1N101701

ATGAAGGCAATACTAGTAGTTCTGCTATATACATTTGCAACCGCAAATGCAGACACATTATGTATAGGTTATCATGCGAACAATTCAACAGACACTGTAGACACAATACTAGAAAAGAATGTAACAGTAACACACTCTGTTAACCTTCTAGAAGACAAGCATAACGGGAAACTATGCAAACTAAGAGGGGTAGCCCCATTGCATTTGGGTAAATGTAACATTGCTGGCTGGATCCTGGGAAATCCAGAGTGTGAATCACTCTCCACAGCAAGCTCATGGTCCTACATTGTGGAAACATCTAGTTCAGACAATGGAACGTGTTACCCAGGAGATTTCATCGATTATGAGGAGCTAAGAGAGCAATTGAGCTCAGTGTCATCATTTGAAAGGTTTGAGATATTCCCCAAGACAAGTTCATGGCCCAATCATGACTCGAACAAAGGTGTAACGGCAGCATGTCCTCATGCTGGAGCAAAAAGCTTCTACAAAAATTTAATATGGCTAGTTAAAAAAGGAAATTCATACCCAAAGCTCAGCAAATCCTACATTAATGATAAAGGGAAAGAAGTCCTCGTGCTATGGGGCATTCACCATCCATCTACTAGTGCTGACCAACAAAGTCTCTATCAGAATGCAGATGCATATGTTTTTGTGGGGACATCAAGATACAGCAAGAAGTTCAAGCCGGAAATAGCAATAAGACCCAAAGTGAGGGATCAAGAAGGGAGAATGAACTATTACTGGACACTAGTAGAGCCGGGAGACAAAATAACATTCGAAGCAACTGGAAATCTAGTGGTACCGAGATATGCATTCGCAATGGAAAGAAATGCTGGATCTGGTATTATCATTTCAGATACACCAGTCCACGATTGCAATACAACTTGTCAGACACCCAAGGGTGCTATAAACACCAGCCTCCCATTTCAGAATATACATCCGATCACAATTGGAAAATGTCCAAAATATGTAAAAAGCACAAAATTGAGACTGGCCACAGGATTGAGGAATGTCCCGTCTATTCAATCTAGAGGCCTATTTGGGGCCATTGCCGGTTTCATTGAAGGGGGGTGGACAGGGATGGTAGATGGATGGTACGGTTATCACCATCAAAATGAGCAGGGGTCAGGATATGCAGCCGACCTGAAGAGCACACAGAATGCCATTGACGAGATTACTAACAAGGTAAATTCTGTTATTGAAAAGATGAATACACAGTTCACAGCAGTAGGTAAAGAGTTCAACCACCTGGAAAAAAGAATAGAGAATTTAAATAAAAAGGTTGATGATGGTTTCCTGGACATTTGGACTTACAATGCCGAACTGTTGGTTCTATTGGAAAATGAAAGAACTTTGGACTACCACGATTCAAATGTGAAGAACTTATATGAAAAGGTAAGAAGCCAGTTAAAAAACAATGCCAAGGAAATTGGAAACGGCTGCTTTGAATTTTACCACAAATGCGATAACACGTGCATGGAAAGTGTCAAAAATGGGACTTATGACTACCCAAAATACTCAGAGGAAGCAAAATTAAACAGAGAAGAAATAGATGGGGTAAAGCTGGAATCAACAAGGATTTACCAGATTTTGGCGATCTATTCAACTGTCGCCAGTTCATTGGTACTGGTAGTCTCCCTGGGGGCAATCAGTTTCTGGATGTGCTCTAATGGGTCTCTACAGTGTAGAATATGTATTTAA

>LC638193.1Influenza A virus H1N101701

ATGAAGGCAATACTAGTAGTTCTGCTATATACATTTGCAACCGCAAATGCAGACACATTATGTATAGGTTATCATGCGAACAATTCAACAGACACTGTAGACACAATACTAGAAAAGAATGTAACAGTAACACACTCTGTTAACCTTCTAGAAGACAAGCATAACGGGAAACTATGCAAACTAAGAGGGGTAGCCCCATTGCATTTGGGTAAATGTAACATTGCTGGCTGGATCCTGGGAAATCCAGAGTGTGAATCACTCTCCACAGCAAGCTCATGGTCCTACATTGTGGAAACATCTAGTTCAGACAATGGAACGTGTTACCCAGGAGATTTCATCGATTATGAGGAGCTAAGAGAGCAATTGAGCTCAGTGTCATCATTTGAAAGGTTTGAGATATTCCCCAAGACAAGTTCATGGCCCAATCATGACTCGAACAAAGGTGTAACGGCAGCATGTCCTCATGCTGGAGCAAAAAGCTTCTACAAAAATTTAATATGGCTAGTTAAAAAAGGAAATTCATACCCAAAGCTCAGCAAATCCTACATTAATGATAAAGGGAAAGAAGTCCTCGTGCTATGGGGCATTCACCATCCATCTACTAGTGCTGACCAACAAAGTCTCTATCAGAATGCAGATGCATATGTTTTTGTGGGGACATCAAGATACAGCAAGAAGTTCAAGCCGGAAATAGCAATAAGACCCAAAGTGAGGGATCAAGAAGGGAGAATGAACTATTACTGGACACTAGTAGAGCCGGGAGACAAAATAACATTCGAAGCAACTGGAAATCTAGTGGTACCGAGATATGCATTCGCAATGGAAAGAAATGCTGGATCTGGTATTATCATTTCAGATACACCAGTCCACGATTGCGATACAACTTGTCAGACACCCAAGGGTGCTATAAACACCAGCCTCCCATTTCAGAATATACATCCGATCACAATTGGAAAATGTCCAAAATATGTAAAAAGCACAAAATTGAGACTGGCCACAGGATTGAGGAATGTCCCGTCTATTCAATCTAGAGGCCTATTTGGGGCCATTGCCGGTTTCATTGAAGGGGGGTGGACAGGGATGGTAGATGGATGGTACGGTTATCACCATCAAAATGAGCAGGGGTCAGGATATGCAGCCGACCTGAAGAGCACACAGAATGCCATTGACGAGATTACTAACAAGGTAAATTCTGTTATTGAAAAGATGAATACACAGTTCACAGCAGTAGGTAAAGAGTTCAACCACCTGGAAAAAAGAATAGAGAATTTAAATAAAAAGGTTGATGATGGTTTCCTGGACATTTGGACTTACAATGCCGAACTGTTGGTTCTATTGGAAAATGAAAGAACTTTGGACTACCACGATTCAAATGTGAAGAACTTATATGAAAAGGTAAGAAGCCAGTTAAAAAACAATGCCAAGGAAATTGGAAACGGCTGCTTTGAATTTTACCACAAATGCGATAACACGTGCATGGAAAGTGTCAAAAATGGGACTTATGACTACCCAAAATACTCAGAGGAAGCAAAATTAAACAGAGAAGAAATAGATGGGGTAAAGCTGGAATCAACAAGGATTTACCAGATTTTGGCGATCTATTCAACTGTCGCCAGTTCATTGGTACTGGTAGTCTCCCTGGGGGCAATCAGTTTCTGGATGTGCTCTAATGGGTCTCTACAGTGTAGAATATGTATTTAA

>LC638192.1Influenza A virus H1N101701

ATGAAGGCAATACTAGTAGTTCTGCTATATACATTTGCAACCGCAAATGCAGACACATTATGTATAGGTTATCATGCGAACAATTCAACTGACACTGTAGACACAGTACTAGAAAAGAATGTAACAGTAACACACTCTGTTAACCTTCTAGAAGACAAGCATAACGGGAAACTATGCAAACTAAGAGGGGTAGCCCCATTGCATTTGGGTAAATGTAACATTGCTGGCTGGATCCTGGGAAATCCAGAGTGTGAATCACTCTCCACAGCAAGCTCATGGTCCTACATTGTGGAAACATCTAGTTCAGACAATGGAACGTGTTACCCAGGAGATTTCATCGATTATGAGGAGCTAAGAGAGCAATTGAGCTCAGTGTCATCATTTGAAAGGTTTGAGATATTCCCCAAGACAAGTTCATGGCCCAATCATGACTCGAACAAAGGTGTGACGGCAGCATGTCCTCATGCTGGAGCAAAAAGCTTCTACAAAAATTTAATATGGCTAGTTAAAAAAGGAAATTCATACCCAAAGCTCAGCAAATCCTACATTAATGATAAAGGGAGAGAAGTCCTCGTGCTATGGGGCATTCACCATCCATCTACTAGTGCTGACCAACAAAGTCTCTATCAGAATGCAGATACATATGTTTTTGTGGGGACATCAAGATACAGCAAGAAGTTCAAGCCGGAAATAGCAATAAGACCCAAAGTGAGGGATCAAGAAGGGAGAATGAACTATTACTGGACACTAGTAGAGCCGGGAGACAAAATAACATTCGAAGCAACTGGAAATCTAGTGGTACCGAGATATGCATTCGCAATGGAAAGAAATGCTGGATCTGGTATTATCATTTCAGATACACCAGTCCACGATTGCAATACAACTTGTCAGACACCCAAGGGTGCTATAAACACCAGCCTCCCATTTCAGAATATACATCCGATCACAATTGGAAAATGTCCAAAATATGTAAAAAGCACAAAATTGAGACTGGCCACAGGATTGAGGAATGTCCCGTCTATTCAATCTAGAGGCCTATTTGGGGCCATTGCCGGTTTCATTGAAGGGGGGTGGACAGGGATGGTAGATGGATGGTACGGTTATCACCATCAAAATGAGCAGGGGTCAGGATATGCAGCCGACCTGAAGAGCACACAGAATGCCATTGACGTGATTACTAACAAAGTAAATTCTGTTATTGAAAAGATGAATACACAGTTCACAGCAGTAGGTAAAGAGTTCAACCACCTGGAAAAAAGAATAGAGAATTTAAATAAAAAAGTTGATGATGGTTTCCTGGACATTTGGACTTACAATGCCGAACTGTTGGTTCTATTGGAAAATGAAAGAACTTTGGACTACCACGATTCAAATGTGAAGAACTTATATGAAAAGGTAAGAAGCCAGTTAAAAAACAATGCCAAGGAAATTGGAAACGGCTGCTTTGAATTTTACCACAAATGCGATAACACGTGCATGGAAAGTGTCAAAAATGGGACTTATGACTACCCAAAATACTCAGAGGAAGCAAAATTAAACAGAGAAGAAATAGATGGGGTAAAGCTGGAATCAACAAGGATTTACCAGATTTTGGCGATCTATTCAACTGTCGCCAGTTCATTGGTACTGGTAGTCTCCCTGGGGGCAATCAGTTTCTGGATGTGCTCTAATGGGTCTCTACAGTGTAGAATATGTATTTAA

>LC638191.1Influenza A virus H1N101701

ATGAAGGCAATACTAGTAGTTCTGCTATATACATTTGCAACCGCAAATGCAGACACATTATGTATAGGTTATCATGCGAACAATTCAACTGACACTGTAGACACAGTACTAGAAAAGAATGTAACAGTAACACACTCTGTTAACCTTCTAGAAGACAAGCATAACGGGAAACTATGCAAACTAAGAGGGGTAGCCCCATTGCATTTGGGTAAATGTAACATTGCTGGCTGGATCCTGGGAAATCCAGAGTGTGAATCACTCTCCACAGCAAGCTCATGGTCCTACATTGTGGAAACATCTAGTTCAGACAATGGAACGTGTTACCCAGGAGATTTCATCGATTATGAGGAGCTAAGAGAGCAATTGAGCTCAGTGTCATCATTTGAAAGGTTTGAGATATTCCCCAAGACAAGTTCATGGCCCAATCATGACTCGAACAAAGGTGTGACGGCAGCATGTCCTCATGCTGGAGCAAAAAGCTTCTACAAAAATTTAATATGGCTAGTTAAAAAAGGAAATTCATACCCAAAGCTCAGCAAATCCTACATTAATGATAAAGGGAGAGAAGTCCTCGTGCTATGGGGCATTCACCATCCATCTACTAGTGCTGACCAACAAAGTCTCTATCAGAATGCAGATACATATGTTTTTGTGGGGACATCAAGATACAGCAAGAAGTTCAAGCCGGAAATAGCAATAAGACCCAAAGTGAGGGATCAAGAAGGGAGAATGAACTATTACTGGACACTAGTAGAGCCGGGAGACAAAATAACATTCGAAGCAACTGGAAATCTAGTGGTACCGAGATATGCATTCGCAATGGAAAGAAATGCTGGATCTGGTATTATCATTTCAGATACACCAGTCCACGATTGCAATACAACTTGTCAGACACCCAAGGGTGCTATAAACACCAGCCTCCCATTTCAGAATATACATCCGATCACAATTGGAAAATGTCCAAAATATGTAAAAAGCACAAAATTGAGACTGGCCACAGGATTGAGGAATGTCCCGTCTATTCAATCTAGAGGCCTATTTGGGGCCATTGCCGGTTTCATTGAAGGGGGGTGGACAGGGATGGTAGATGGATGGTACGGTTATCACCATCAAAATGAGCAGGGGTCAGGATATGCAGCCGACTTGAAGAGCACACAGAATGCCATTGACGAGATTACTAACAAAGTAAATTCTGTTATTGAAAAGATGAATACACAGTTCACAGCAGTAGGTAAAGAGTTCAACCACCTGGAAAAAAGAATAGAGAATTTAAATAAAAAAGTTGATGATGGTTTCCTGGACATTTGGACTTACAATGCCGAACTGTTGGTTCTATTGGAAAATGAAAGAACTTTGGACTACCACGATTCAAATGTGAAGAACTTATATGAAAAGGTAAGAAGCCAGTTAAAAAACAATGCCAAGGAAATTGGAAACGGCTGCTTTGAATTTTACCACAAATGCGATAACACGTGCATGGAAAGTGTCAAAAATGGGACTTATGACTACCCAAAATACTCAGAGGAAGCAAAATTAAACAGAGAAGAAATAGATGGGGTAAAGCTGGAATCAACAAGGATTTACCAGATTTTGGCGATCTATTCAACTGTCGCCAGTTCATTGGTACTGGTAGTCTCCCTGGGGGCAATCAGTTTCTGGATGTGCTCTAATGGGTCTCTACAGTGTAGAATATGTATTTAA

>LC638190.1Influenza A virus H1N101701

ATGAAGGCAATACTAGTAGTTCTGCTATATACATTTGCAACCGCAAATGCAGACACATTATGTATAGGTTATCATGCGAACAATTCAACAGACACTGTAGACACAATACTAGAAAAGAATGTAACAGTAACACACTCTGTTAACCTTCTAGAAGACAAGCATAACGGGAAACTATGCAAACTAAGAGGGGTAGCCCCATTGCATTTGGGTAAATGTAACATTGCTGGCTGGATCCTGGGAAATCCAGAGTGTGAATCACTCTCCACAGCAAGCTCATGGTCCTACATTGTGGAAACATCTAGTTCAGACAATGGAACGTGTTACCCAGGAGATTTCATCGATTATGAGGAGCTAAGAGAGCAATTGAGCTCAGTGTCATCATTTGAAAGGTTTGAGATATTCCCCAAGACAAGTTCATGGCCCAATCATGACTCGAACAAAGGTGTAACGGCAGCATGTCCTCATGCTGGAGCAAAAAGCTTCTACAAAAATTTAATATGGCTAGTTAAAAAAGGAAATTCATACCCAAAGCTCAGCAAATCCTACATTAATGATAAAGGGAAAGAAGTCCTCGTGCTATGGGGCATTCACCATCCATCTACTAGTGCTGACCAACAAAGTCTCTATCAGAATGCAGATGCATATGTTTTTGTGGGGACATCAAGATACAGCAAGAAGTTCAAGCCGGAAATAGCAATAAGACCCAAAGTGAGGGATCAAGAAGGGAGAATGAACTATTACTGGACACTAGTAGAGCCGGGAGACAAAATAACATTCGAAGCAACTGGAAATCTAGTGGTACCGAGATATGCATTCGCAATGGAAAGAAATGCTGGATCTGGTATTATCATTTCAGATACACCAGTCCACGATTGCGATACAACTTGTCAGACACCCAAGGGTGCTATAAACACCAGCCTCCCATTTCAGAATATACATCCGATCACAATTGGAAAATGTCCAAAATATGTAAAAAGCACAAAATTGAGACTGGCCACAGGATTGAGGAATGTCCCGTCTATTCAATCTAGAGGCCTATTTGGGGCCATTGCCGGTTTCATTGAAGGGGGGTGGACAGGGATGGTAGATGGATGGTACGGTTATCACCATCAAAATGAGCAGGGGTCAGGATATGCAGCCGACCTGAAGAGCACACAGAATGCCATTGACGAGATTACTAACAAGGTAAATTCTGTTATTGAAAAGATGAATACACAGTTCACAGCAGTAGGTAAAGAGTTCAACCACCTGGAAAAAAGAATAGAGAATTTAAATAAAAAGGTTGATGATGGTTTCCTGGACATTTGGACTTACAATGCCGAACTGTTGGTTCTATTGGAAAATGAAAGAACTTTGGACTACCACGATTCAAATGTGAAGAACTTATATGAAAAGGTAAGAAGCCAGTTAAAAAACAATGCCAAGGAAATTGGAAACGGCTGCTTTGAATTTTACCACAAATGCGATAACACGTGCATGGAAAGTGTCAAAAATGGGACTTATGACTACCCAAAATACTCAGAGGAAGCAAAATTAAACAGAGAAGAAATAGATGGGGTAAAGCTGGAATCAACAAGGATTTACCAGATTTTGGCGATCTATTCAACTGTCGCCAGTTCATTGGTACTGGTAGTCTCCCTGGGGGCAATCAGTTTCTGGATGTGCTCTAATGGGTCTCTACAGTGTAGAATATGTATTTAA

>LC638189.1Influenza A virus H1N101701

ATGAAGGCAATACTAGTAGTTCTGCTATATACATTTGCAACCGCAAATGCAGACACATTATGTATAGGTTATCATGCGAACAATTCAACAGACACTGTAGACACAATACTAGAAAAGAATGTAACAGTAACACACTCTGTTAACCTTCTAGAAGACAAGCATAACGGGAAACTATGCAAACTAAGAGGGGTAGCCCCATTGCATTTGGGTAAATGTAACATTGCTGGCTGGATCCTGGGAAATCCAGAGTGTGAATCACTCTCCACAGCAAGCTCATGGTCCTACATTGTGGAAACATCTAGTTCAGACAATGGAACGTGTTACCCAGGAGATTTCATCGATTATGAGGAACTAAGAGAGCAATTGAGCTCAGTGTCATCATTTGAAAGGTTTGAGATATTCCCCAAGACAAGTTCATGGCCCAATCATGACTCGAACAAAGGTGTAACGGCAGCATGTCCTCATGCTGGAGCAAAAAGCTTCTACAAAAATTTAATATGGCTAGTTAAAAAAGGAAATTCATACCCAAAGCTCAGCAAATCCTACATTAATGATAAAGGGAAAGAAGTCCTCGTGCTATGGGGCATTCACCATCCATCTACTAGTGCTGACCAACAAAGTCTCTATCAGAATGCAGATGCATATGTTTTTGTGGGGACATCAAGATACAGCAAGAAGTTCAAGCCGGAAATAGCAATAAGACCCAAAGTGAGGGATCAAGAAGGGAGAATGAACTATTACTGGACACTAGTAGAGCCGGGAGACAAAATAACATTCGAAGCAACTGGAAATCTAGTGGTACCGAGATATGCATTCGCAATGGAAAGAAATGCTGGATCTGGTATTATCATTTCAGATACACCAGTCCACGATTGCAATACAACTTGTCAGACACCCAAGGGTGCTATAAACACCAGCCTCCCATTTCAGAATATACATCCGATCACAATTGGAAAATGTCCAAAATATGTAAAAAGCACAAAATTGAGACTGGCCACAGGATTGAGGAATGTCCCGTCTATTCAATCTAGAGGCCTATTTGGGGCCATTGCCGGTTTCATTGAAGGGGGGTGGACAGGGATGGTAGATGGATGGTACGGTTATCACCATCAAAATGAGCAGGGGTCAGGATATGCAGCCGACCTGAAGAGCACACAGAATGCCATTGACGTGATTACTAACAAGGTAAATTCTGTTATTGAAAAGATGAATACACAGTTCACAGCAGTAGGTAAAGAGTTCAACCACCTGGAAAAAAGAATAGAGAATTTAAATAAAAAGGTTGATGATGGTTTCCTGGACATTTGGACTTACAATGCCGAACTGTTGGTTCTATTGGAAAATGAAAGAACTTTGGACTACCACGATTCAAATGTGAAGAACTTATATGAAAAGGTAAGAAGCCAGTTAAAAAACAATGCCAAGGAAATTGGAAACGGCTGCTTTGAATTTTACCACAAATGCGATAACACGTGCATGGAAAGTGTCAAAAATGGGACTTATGACTACCCAAAATACTCAGAGGAAGCAAAATTAAACAGAGAAAAAATAGATGGGGTAAAGCTGGAATCAACAAGGATTTACCAGATTTTGGCGATCTATTCAACTGTCGCCAGTTCATTGGTACTGGTAGTCTCCCTGGGGGCAATCAGTTTCTGGATGTGCTCTAATGGGTCTCTACAGTGTAGAATATGTATTTAA

>LC638188.1Influenza A virus H1N101701

ATGAAGGCAATACTAGTAGTTCTGCTATATACATTTGCAACCGCAAATGCAGACACATTATGTATAGGTTATCATGCGAACAATTCAACAGACACTGTAGACACAATACTAGAAAAGAATGTAACAGTAACACACTCTGTTAACCTTCTAGAAGACAAGCATAACGGGAAACTATGCAAACTAAGAGGGGTAGCCCCATTGCATTTGGGTAAATGTAACATTGCTGGCTGGATCCTGGGAAATCCAGAGTGTGAATCACTCTCCACAGCAAGCTCATGGTCCTACATTGTGGAAACATCTAGTTCAGACAATGGAACGTGTTACCCAGGAGATTTCATCGATTATGAGGAGCTAAGAGAGCAATTGAGCTCAGTGTCATCATTTGAAAGGTTTGAGATATTCCCCAAGACAAGTTCATGGCCCAATCATGACTCGAACAAAGGTGTAACGGCAGCATGTCCTCATGCTGGAGCAAAAAGCTTCTACAAAAATTTAATATGGCTAGTTAAAAAAGGAAATTCATACCCAAAGCTCAGCAAATCCTACATTAATGATAAAGGGAAAGAAGTCCTCGTGCTATGGGGCATTCACCATCCATCTACTAGTGCTGACCAACAAAGTCTCTATCAGAATGCAGATGCATATGTTTTTGTGGGGACATCAAGATACAGCAAGAAGTTCAAGCCGGAAATAGCAATAAGACCCAAAGTGAGGGATCAAGAAGGGAGAATGAACTATTACTGGACACTAGTAGAGCCGGGAGACAAAATAACATTCGAAGCAACTGGAAATCTAGTGGTACCGAGATATGCATTCGCAATGGAAAGAAATGCTGGATCTGGTATTATCATTTCAGATACACCAGTCCACGATTGCAATACAACTTGTCAGACACCCAAGGGTGCTATAAACACCAGCCTCCCATTTCAGAATATACATCCGATCACAATTGGAAAATGTCCAAAATATGTAAAAAGCACAAAATTGAGACTGGCCACAGGATTGAGGAATGTCCCGTCTATTCAATCTAGAGGCCTATTTGGGGCCATTGCCGGTTTCATTGAAGGGGGGTGGACAGGGATGGTAGATGGATGGTACGGTTATCACCATCAAAATGAGCAGGGGTCAGGATATGCAGCCGACCTGAAGAGCACACAGAATGCCATTGACGAGATTACTAACAAGGTAAATTCTGTTATTGAAAAGATGAATACACAGTTCACAGCAGTAGGTAAAGAGTTCAACCACCTGGAAAAAAGAATAGAGAATTTAAATAAAAAGGTTGATGATGGTTTCCTGGACATTTGGACTTACAATGCCGAACTGTTGGTTCTATTGGAAAATGAAAGAACTTTGGACTACCACGATTCAAATGTGAAGAACTTATATGAAAAGGTAAGAAGCCAGTTAAAAAACAATGCCAAGGAAATTGGAAACGGCTGCTTTGAATTTTACCACAAATGCGATAACACGTGCATGGAAAGTGTCAAAAATGGGACTTATGACTACCCAAAATACTCAGAGGAAGCAAAATTAAACAGAGAAGAAATAGATGGGGTAAAGCTGGAATCAACAAGGATTTACCAGATTTTGGCGATCTATTCAACTGTCGCCAGTTCATTGGTACTGGTAGTCTCCCTGGGGGCAATCAGTTTCTGGATGTGCTCTAATGGGTCTCTACAGTGTAGAATATGTATTTAA

>LC638187.1Influenza A virus H1N101701

ATGAAGGCAATACTAGTAGTTCTGCTATATACATTTGCAACCGCAAATGCAGACACATTATGTATAGGTTATCATGCGAACAATTCAACTGACACTGTAGACACAGTACTAGAAAAGAATGTAACAGTAACACACTCTGTTAACCTTCTAGAAGACAAGCATAACGGGAAACTATGCAAACTAAGAGGGGTAGCCCCATTGCATTTGGGTAAATGTAACATTGCTGGCTGGATCCTGGGAAATCCAGAGTGTGAATCACTCTCCACAGCAAGCTCATGGTCCTACATTGTGGAAACATCTAGTTCAGACAATGGAACGTGTTACCCAGGAGATTTCATCGATTATGAGGAGCTAAGAGAGCAATTGAGCTCAGTGTCATCATTTGAAAGGTTTGAGATATTCCCCAAGACAAGTTCATGGCCCAATCATGACTCGAACAAAGGTGTGACGGCAGCATGTCCTCATGCTGGAGCAAAAAGCTTCTACAAAAATTTAATATGGCTAGTTAAAAAAGGAAATTCATACCCAAAGCTCAGCAAATCCTACATTAATGATAAAGGGAGAGAAGTCCTCGTGCTATGGGGCATTCACCATCCATCTACTAGTGCTGACCAACAAAGTCTCTATCAGAATGCAGATACATATGTTTTTGTGGGGACATCAAGATACAGCAAGAAGTTCAAGCCGGAAATAGCAATAAGACCCAAAGTGAGGGATCAAGAAGGGAGAATGAACTATTACTGGACACTAGTAGAGCCGGGAGACAAAATAACATTCGAAGCAACTGGAAATCTAGTGGTACCGAGATATGCATTCGCAATGGAAAGAAATGCTGGATCTGGTATTATCATTTCAGATACACCAGTCCACGATTGCAATACAACTTGTCAGACACCCAAGGGTGCTATAAACACCAGCCTCCCATTTCAGAATATACATCCGATCACAATTGGAAAATGTCCAAAATATGTAAAAAGCACAAAATTGAGACTGGCCACAGGATTGAGGAATGTCCCGTCTATTCAATCTAGAGGCCTATTTGGGGCCATTGCCGGTTTCATTGAAGGGGGGTGGACAGGGATGGTAGATGGATGGTACGGTTATCACCATCAAAATGAGCAGGGGTCAGGATATGCAGCCGACCTGAAGAGCACACAGAATGCCATTGACGTGATTACTAACAAAGTAAATTCTGTTATTGAAAAGATGAATACACAGTTCACAGCAGTAGGTAAAGAGTTCAACCACCTGGAAAAAAGAATAGAGAATTTAAATAAAAAAGTTGATGATGGTTTCCTGGACATTTGGACTTACAATGCCGAACTGTTGGTTCTATTGGAAAATGAAAGAACTTTGGACTACCACGATTCAAATGTGAAGAACTTATATGAAAAGGTAAGAAGCCAGTTAAAAAACAATGCCAAGGAAATTGGAAACGGCTGCTTTGAATTTTACCACAAATGCGATAACACGTGCATGGAAAGTGTCAAAAATGGGACTTATGACTACCCAAAATACTCAGAGGAAGCAAAATTAAACAGAGAAGAAATAGATGGGGTAAAGCTGGAATCAACAAGGATTTACCAGATTTTGGCGATCTATTCAACTGTCGCCAGTTCATTGGTACTGGTAGTCTCCCTGGGGGCAATCAGTTTCTGGATGTGCTCTAATGGGTCTCTACAGTGTAGAATATGTATTTAA

>LC638186.1Influenza A virus H1N101701

ATGAAGGCAATACTAGTAGTTCTGCTATATACATTTGCAACCGCAAATGCAGACACATTATGTATAGGTTATCATGCGAACAATTCAACAGACACTGTAGACACAATACTAGAAAAGAATGTAACAGTAACACACTCTGTTAACCTTCTAGAAGACAAGCATAACGGGAAACTATGCAAACTAAGAGGGGTAGCCCCATTGCATTTGGGTAAATGTAACATTGCTGGCTGGATCCTGGGAAATCCAGAGTGTGAATCACTCTCCACAGCAAGCTCATGGTCCTACATTGTGGAAACATGTAGTTCAGACAATGGAACGTGTTACCCAGGAGATTTCATCGATTATGAGGAGCTAAGAGAGCAATTGAGCTCAGTGTCATCATTTGAACGGTTTGAGATATTCCCCAAGACAAGTTCATGGCCCAATCATGACTCGAACAAAGGTGTAACGGCAGCATGTCCTCATGCTGGAGCAAAAAGCTTCTACAAAAATTTAATATGGCTAATTAAAAAAGGAAATTCATACCCAAAGCTCAGCAAATCCTACATTAATGATAAAGGGAAAGAAGTCCTCGTGCTATGGGGCATTCACCATCCATCTACTAGTGCTGACCAACAAAGTCTCTATCAGAATGCAGATGCATATGTTTTTGTGGGGACATCAAGATACAGCAAGAAGTTCAAGCCGGAAATAGCAATAAGACCCAAAGTGAGGGATCAAGAAGGGAGAATGAACTATTACTGGACACTAGTAGAGCCGGGAGACAAAATAACATTCGAAGCAACTGGAAATCTAGTGGTACCGAGATATGCATTCGCAATGGAAAGAAATGCTGGATCTGGTATTATCATTTCAGATACACCAGTCCACGATTGCAATACAACTTGTCAGACACCCAAGGGTGCTATAAACACCAGCCTCCCATTTCAGAATATACATCCGATCACAATTGGAAAATGTCCAAAATATGTAAAAAGCACAAAATTGAGACTGGCCACAGGATTGAGGAATGTCCCGTCTATTCAATCTAGAGGCCTATTTGGGGCCATTGCCGGTTTCATTGAAGGGGGGTGGACAGGGATGGTAGATGGATGGTACGGTTATCACCATCAAAATGAGCAGGGGTCAGGATATGCAGCCGACCTGAAGAGCACACAGAATGCCATTGACGAGATTACTAACAAAGTAAATTCTGTTATTGAAAAGATGAATACACAGTTCACAGCAGTAGGTAAAGAGTTCAACCACCTGGAAAAAAGAATAGAGAATTTAAATAAAAAGGTTGATGATGGTTTCCTGGACATTTGGACTTACAATGCCGAACTGTTGGTTCTATTGGAAAATGAAAGAACTTTGGACTACCACGATTCAAATGTGAAGAACTTATATGAAAAGGTAAGAAGCCAGTTAAAAAACAATGCCAAGGAAATTGGAAACGGCTGCTTTGAATTTTACCACAAATGCGATAACACGTGCATGGAAAGTGTCAAAAATGGGACTTATGACTACCCAAAATACTCAGAGGAAGCAAAATTAAACAGAGAAGAAATAGATGGGGTAAAGCTGGAATCAACAAGGATTTACCAGATTTTGGCGATCTATTCAACTGCCGCCAGTTCATTGGTACTGGTAGTCTCCCTGGGGGCAATCAGTTTCTGGATGTGCTCTAATGGGTCTCTACAGTGTAGAATATGTATTTAA

>LC638185.1Influenza A virus H1N101701

ATGAAGGCAATACTAGTAGTTCTGCTATATACATTTGCAACCGCAAATGCAGACACATTATGTATAGGTTATCATGCGAACAATTCAACAGACACTGTAGACACAATACTAGAAAAGAATGTAACAGTAACACACTCTGTTAACCTTCTAGAAGACAAGCATAACGGGAAACTATGCAAACTAAGAGGGGTAGCCCCATTGCATTTGGGTAAATGTAACATTGCTGGCTGGATCCTGGGAAATCCAGAGTGTGAATCACTCTCCACAGCAAGCTCATGGTCCTACATTGTGGAAACATCTAGTTCAGACAATGGAACGTGTTACCCAGGAGATTTCATCGATTATGAGGAGCTAAGAGAGCAATTGAGCTCAGTGTCATCATTTGAAAGGTTTGAGATATTCCCCAAGACAAGTTCATGGCCCAATCATGACTCGAACAAAGGTGTAACGGCAGCATGTCCTCATGCTGGAGCAAAAAGCTTCTACAAAAATTTAATATGGCTAGTTAAAAAAGGAAATTCATACCCAAAGCTCAGCAAATCCTACATTAATGATAAAGGGAAAGAAGTCCTCGTGCTATGGGGCATTCACCATCCATCTACTAGTGCTGACCAACAAAGTCTCTATCAGAATGCAGATGCATATGTTTTTGTGGGGACATCAAGATACAGCAAGAAGTTCAAGCCGGAAATAGCAATAAGACCCAAAGTGAGGGATCAAGAAGGGAGAATGAACTATTACTGGACACTAGTAGAGCCGGGAGACAAAATAACATTCGAAGCAACTGGAAATCTAGTGGTACCGAGATATGCATTCGCAATGGAAAGAAATGCTGGATCTGGTATTATCATTTCAGATACACCAGTCCACGATTGCAATACAACTTGTCAGACACCCAAGGGTGCTATAAACACCAGCCTCCCATTTCAGAATATACATCCGATCACAATTGGAAAATGTCCAAAATATGTAAAAAGCACAAAATTGAGACTGGCCACAGGATTGAGGAATGTCCCGTCTATTCAATCTAGAGGCCTATTTGGGGCCATTGCCGGTTTCATTGAAGGGGGGTGGACAGGGATGGTAGATGGATGGTACGGTTATCACCATCAAAATGAGCAGGGGTCAGGATATGCAGCCGACCTGAAGAGCACACAGAATGCCATTGACGAGATTACTAACAAGGTAAATTCTGTTATTGAAAAGATGAATACACAGTTCACAGCAGTAGGTAAAGAGTTCAACCACCTGGAAAAAAGAATAGAGAATTTAAATAAAAAGGTTGATGATGGTTTCCTGGACATTTGGACTTACAATGCCGAACTGTTGGTTCTATTGGAAAATGAAAGAACTTTGGACTACCACGATTCAAATGTGAAGAACTTATATGAAAAGGTAAGAAGCCAGTTAAAAAACAATGCCAAGGAAATTGGAAACGGCTGCTTTGAATTTTACCACAAATGCGATAACACGTGCATGGAAAGTGTCAAAAATGGGACTTATGACTACCCAAAATACTCAGAGGAAGCAAAATTAAACAGAGAAGAAATAGATGGGGTAAAGCTGGAATCAACAAGGATTTACCAGATTTTGGCGATCTATTCAACTGTCGCCAGTTCATTGGTACTGGTAGTCTCCCTGGGGGCAATCAGTTTCTGGATGTGCTCTAATGGGTCTCTACAGTGTAGAATATGTATTTAA

>LC638184.1Influenza A virus H1N101701

ATGAAGGCAATACTAGTAGTTCTGCTATATACATTTGCAACCGCAAATGCAGACACATTATGTATAGGTTATCATGCGAACAATTCAACAGACACTGTAGACACAGTACTAGAAAAGAATGTAACAGTAACACATTCTGTTAACCTTCTAGAAGACAAGCATAACGGGAAACTATGCAAACTAAGAGGGGTAGCCCCATTGCATTTGGGTAAATGTAACATTGCTGGCTGGATCCTGGGAAATCCAGAGTGTGAATCACTCTCCACAGCAAGCTCATGGTCCTACATTGTGGAAACATCTAGTTCAGACAATGGAACGTGTTACCCAGGAGATTTCATCGATTATGAGGAGCTAAGAGAGCAATTGAGCTCAGTGTCATCATTTGAAAGGTTTGAGATATTCCCCAAGACAAGTTCATGGCCCAATCATGACTCGAACAAAGGTGTAACGGCAGCATGTCCTCATGCTGGAGCAAAAAGCTTCTACAAAAATTTAATATGGCTAGTTAAAAAAGGAAATTCATACCCAAAGCTCAGCAAATCCTACATTAATGATAAAGGGAAAGAAGTCCTCGTGCTATGGGGCATTCATCATCCATCTACTAGTGCTGACCAACAAAGTCTCTATCAGAATGCAGATGCATATGTTTTTGTGGGGACATCAAGATACAGCAAGAAGTTCAAGCCGGAAATAGCAATAAGACCCAAAGTGAGGGATCAAGAAGGGAGAATGAACTATTACTGGACACTAGTAGAGCCGGGAGACAAAATAACATTCGAAGCAACTGGAAATCTAGTGGTACCGAGATATGCATTCGCAATGGAAAGAAATGCTGGATCTGGTATTATCATTTCAGATACACCAGTCCACGATTGCAATACAACTTGTCAGACACCCAAGGGTGCTATAAACACCAGCCTCCCATTTCAGAATATACATCCGATCACAATTGGAAAATGTCCAAAATATGTAAAAAGCACAAAATTGAGACTGGCCACAGGATTGAGGAATGTCCCGTCTATTCAATCTAGAGGCCTATTTGGGGCCATTGCCGGTTTCATTGAAGGGGGGTGGACAGGGATGGTAGATGGATGGTACGGTTATCACCATCAAAATGAGCAGGGGTCAGGATATGCAGCCGACCTGAAGAGCACACAGAATGCCATTGACGTGATTACTAACAAAGTAAATTCTGTTATTGAAAAGATGAATACACAGTTCACAGCAGTAGGTAAAGAGTTCAACCACCTGGAAAAAAGAATAGAGAATTTAAATAAAAAGGTTGATGATGGTTTCCTGGACATTTGGACTTACAATGCAGAACTGTTGGTTCTATTGGAAAATGAAAGAACTTTGGACTACCACGATTCAAATGTGAAGAACTTATATGAAAAGGTAAGAAGCCAGTTAAAAAACAATGCCAAGGAAATTGGAAACGGCTGCTTTGAATTTTACCACAAATGCGATAACACGTGCATGGAAAGTGTCAAAAATGGGACTTATGACTACCCAAAATACTCAGAGGAAGCAAAATTAAACAGAGAAGAAATAGATGGGGTAAAGCTGGAATCAACAAGGATTTACCAGATTTTGGCGATCTATTCAACTGTCGCCAGTTCATTGGTACTGGTAGTCTCCCTGGGGGCAATCAGTTTCTGGATGTGCTCTAATGGGTCTCTACAGTGTAGAATATGTATTTAA

>LC638183.1Influenza A virus H1N101701

ATGAAGGCAATACTAGTAGTTCTGCTATATACATTTGCAACCGCAAATGCAGACACATTATGTATAGGTTATCATGCGAACAATTCAACAGACACTGTAGACACAATACTAGAAAAGAATGTAACAGTAACACACTCTGTTAACCTTCTAGAAGACAAGCATAACGGGAAACTATGCAAACTAAGAGGGGTAGCCCCATTGCATTTGGGTAAATGTAACATTGCTGGCTGGATCCTGGGAAATCCAGAGTGTGAATCACTCTCCACAGCAAGCTCATGGTCCTACATTGTGGAAACATCTAGTTCAGACAATGGAACGTGTTACCCAGGAGATTTCATCGATTATGAGGAGCTAAGAGAGCAATTGAGCTCAGTGTCATCATTTGAAAGGTTTGAGATATTCCCCAAGACAAGTTCATGGCCCAATCATGACTCGAACAAAGGTGTAACGGCAGCATGTCCTCATGCTGGAGCAAAAAGCTTCTACAAAAATTTAATATGGCTAGTTAAAAAAGGAAATTCATACCCAAAGCTCAGCAAATCCTACATTAATGATAAAGGGAAAGAAGTCCTCGTGCTATGGGGCATTCACCATCCATCTACTAGTGCTGACCAACAAAGTCTCTATCAGAATGCAGATGCATATGTTTTTGTGGGGACATCAAGATACAGCAAGAAGTTCAAGCCGGAAATAGCAATAAGACCCAAAGTGAGGGATCAAGAAGGGAGAATGAACTATTACTGGACACTAGTAGAGCCGGGAGACAAAATAACATTCGAAGCAACTGGAAATCTAGTGGTACCGAGATATGCATTCGCAATGGAAAGAAATGCTGGATCTGGTATTATCATTTCAGATACACCAGTCCACGATTGCAATACAACTTGTCAGACACCCAAGGGTGCTATAAACACCAGCCTCCCATTTCAGAATATACATCCGATCACAATTGGAAAATGTCCAAAATATGTAAAAAGCACAAAATTGAGACTGGCCACAGGATTGAGGAATGTCCCGTCTATTCAATCTAGAGGCCTATTTGGGGCCATTGCCGGTTTCATTGAAGGGGGGTGGACAGGGATGGTAGATGGATGGTACGGTTATCACCATCAAAATGAGCAGGGGTCAGGATATGCAGCCGACCTGAAGAGCACACAGAATGCCATTGACGAGATTACTAACAAGGTAAATTCTGTTATTGAAAAGATGAATACACAGTTCACAGCAGTAGGTAAAGAGTTCAACCACCTGGAAAAAAGAATAGAGAATTTAAATAAAAAGGTTGATGATGGTTTCCTGGACATTTGGACTTACAATGCCGAACTGTTGGTTCTATTGGAAAATGAAAGAACTTTGGACTACCACGATTCAAATGTGAAGAACTTATATGAAAAGGTAAGAAGCCAGTTAAAAAACAATGCCAAGGAAATTGGAAACGGCTGCTTTGAATTTTACCACAAATGCGATAACACGTGCATGGAAAGTGTCAAAAATGGGACTTATGACTACCCAAAATACTCAGAGGAAGCAAAATTAAACAGAGAAGAAATAGATGGGGTAAAGCTGGAATCAACAAGGATTTACCAGATTTTGGCGATCTATTCAACTGTCGCCAGTTCATTGGTACTGGTAGTCTCCCTGGGGGCAATCAGTTTCTGGATGTGCTCTAATGGGTCTCTACAGTGTAGAATATGTATTTAA

>LC638182.1Influenza A virus H1N101701

ATGAAGGCAATACTAGTAGTTCTGCTATATACATTTGCAACCGCAAATGCAGACACATTATGTATAGGTTATCATGCGAACAATTCAACAGACACTGTAGACACAATACTAGAAAAGAATGTAACAGTAACACACTCTGTTAACCTTCTAGAAGACAAGCATAACGGGAAACTATGCAAACTAAGAGGGGTAGCCCCATTGCATTTGGGTAAATGTAACATTGCTGGCTGGATCCTGGGAAATCCAGAGTGTGAATCACTCTCCACAGCAAGCTCATGGTCCTACATTGTGGAAACATCTAGTTCAGACAATGGAACGTGTTACCCAGGAGATTTCATCGATTATGAGGAGCTAAGAGAGCAATTGAGCTCAGTGTCATCATTTGAACGGTTTGAGATATTCCCCAAGACAAGTTCATGGCCCAATCATGACTCGAACAAAGGTGTAACGGCAGCATGTCCTCATGCTGGAGCAAAAAGCTTCTACAAAAATTTAATATGGCTAGTTAAAAAAGGAAATTCATACCCAAAGCTCAGCAAATCCTACATTAATGATAAAGGGAAAGAAGTCCTCGTGCTATGGGGCATTCACCATCCATCTACTAGTGCTGACCAACAAAGTCTCTATCAGAATGCAGATGCATATGTTTTTGTGGGGACATCAAGATACAGCAAGAAGTTCAAGCCGGAAATAGCAATAAGACCCAAAGTGAGGGATCAAGAAGGGAGAATGAACTATTACTGGACACTAGTAGAGCCGGGAGACAAAATAACATTCGAAGCAACTGGAAATCTAGTGGTACCGAGATATGCATTCGCAATGGAAAGAAATGCTGGATCTGGTATTATCATTTCAGATACACCAGTCCACGATTGCAATACAACTTGTCAGACACCCAAGGGTGCTATAAACACCAGCCTCCCATTTCAGAATATACATCCGATCACAATTGGAAAATGTCCAAAATATGTAAAAAGCACAAAATTGAGACTGGCCACAGGATTGAGGAATGTCCCGTCTATTCAATCTAGAGGCCTATTTGGGGCCATTGCCGGTTTCATTGAAGGGGGGTGGACAGGGATGGTAGATGGATGGTACGGTTATCACCATCAAAATGAGCAGGGGTCAGGATATGCAGCCGACCTGAAGAGCACACAGAATGCCATTGACGAGATTACTAACAAAGTAAATTCTGTTATTGAAAAGATGAATACACAGTTCACAGCAGTAGGTAAAGAGTTCAACCACCTGGAAAAAAGAATAGAGAATTTAAATAAAAAGGTTGATGATGGTTTCCTGGACATTTGGACTTACAATGCCGAACTGTTGGTTCTATTGGAAAATGAAAGAACTTTGGACTACCACGATTCAAATGTGAAGAACTTATATGAAAAGGTAAGAAGCCAGTTAAAAAACAATGCCAAGGAAATTGGAAACGGCTGCTTTGAATTTTACCACAAATGCGATAACACGTGCATGGAAAGTGTCAAAAATGGGACTTATGACTACCCAAAATACTCAGAGGAAGCAAAATTAAACAGAGAAGAAATAGATGGGGTAAAGCTGGAATCAACAAGGATTTACCAGATTTTGGCGATCTATTCAACTGCCGCCAGTTCATTGGTACTGGTAGTCTCCCTGGGGGCAATCAGTTTCTGGATGTGCTCTAATGGGTCTCTACAGTGTAGAATATGTATTTAA

>LC638181.1Influenza A virus H1N101701

ATGAAGGCAATACTAGTAGTTCTGCTATATACATTTGCAACCGCAAATGCAGACACATTATGTATAGGTTATCATGCGAACAATTCAACAGACACTGTAGACACAATACTAGAAAAGAATGTAACAGTAACACACTCTGTTAACCTTCTAGAAGACAAGCATAACGGGAAACTATGCAAACTAAGAGGGGTAGCCCCATTGCATTTGGGTAAATGTAACATTGCTGGCTGGATCCTGGGAAATCCAGAGTGTGAATCACTCTCCACAGCAAGCTCATGGTCCTACATTGTGGAAACATCTAGTTCAGACAATGGAACGTGTTACCCAGGAGATTTCATCGATTATGAGGAACTAAGAGAGCAATTGAGCTCAGTGTCATCATTTGAAAGGTTTGAGATATTCCCCAAGACAAGTTCATGGCCCAATCATGACTCGAACAAAGGTGTAACGGCAGCATGTCCTCATGCTGGAGCAAAAAGCTTCTACAAAAATTTAATATGGCTAGTTAAAAAAGGAAATTCATACCCAAAGCTCAGCAAATCCTACATTAATGATAAAGGGAAAGAAGTCCTCGTGCTATGGGGCATTCACCATCCATCTACTAGTGCTGACCAACAAAGTCTCTATCAGAATGCAGATGCATATGTTTTTGTGGGGACATCAAGATACAGCAAGAAGTTCAAGCCGGAAATAGCAATAAGACCCAAAGTGAGGGATCAAGAAGGGAGAATGAACTATTACTGGACACTAGTAGAGCCGGGAGACAAAATAACATTCGAAGCAACTGGAAATCTAGTGGTACCGAGATATGCATTCGCAATGGAAAGAAATGCTGGATCTGGTATTATCATTTCAGATACACCAGTCCACGATTGCAATACAACTTGTCAGACACCCAAGGGTGCTATAAACACCAGCCTCCCATTTCAGAATATACATCCGATCACAATTGGAAAATGTCCAAAATATGTAAAAAGCACAAAATTGAGACTGGCCACAGGATTGAGGAATGTCCCGTCTATTCAATCTAGAGGCCTATTTGGGGCCATTGCCGGTTTCATTGAAGGGGGGTGGACAGGGATGGTAGATGGATGGTACGGTTATCACCATCAAAATGAGCAGGGGTCAGGATATGCAGCCGACCTGAAGAGCACACAGAATGCCATTGACGTGATTACTAACAAGGTAAATTCTGTTATTGAAAAGATGAATACACAGTTCACAGCAGTAGGTAAAGAGTTCAACCACCTGGAAAAAAGAATAGAGAATTTAAATAAAAAGGTTGATGATGGTTTCCTGGACATTTGGACTTACAATGCCGAACTGTTGGTTCTATTGGAAAATGAAAGAACTTTGGACTACCACGATTCAAATGTGAAGAACTTATATGAAAAGGTAAGAAGCCAGTTAAAAAACAATGCCAAGGAAATTGGAAACGGCTGCTTTGAATTTTACCACAAATGCGATAACACGTGCATGGAAAGTGTCAAAAATGGGACTTATGACTACCCAAAATACTCAGAGGAAGCAAAATTAAACAGAGAAAAAATAGATGGGGTAAAGCTGGAATCAACAAGGATTTACCAGATTTTGGCGATCTATTCAACTGTCGCCAGTTCATTGGTACTGGTAGTCTCCCTGGGGGCAATCAGTTTCTGGATGTGCTCTAATGGGTCTCTACAGTGTAGAATATGTATTTAA

>LC638180.1Influenza A virus H1N101701

ATGAAGGCAATACTAGTAGTTCTGCTATATACATTTGCAACCGCAAATGCAGACACATTATGTATAGGTTATCATGCGAACAATTCAACAGACACTGTAGACACAGTACTAGAAAAGAATGTAACAGTAACACATTCTGTTAACCTTCTAGAAGACAAGCATAACGGGAAACTATGCAAACTAAGAGGGGTAGCCCCATTGCATTTGGGTAAATGTAACATTGCTGGCTGGATCCTGGGAAATCCAGAGTGTGAATCACTCTCCACAGCAAGCTCATGGTCCTACATTGTGGAAACATCTAGTTCAGACAATGGAACGTGTTACCCAGGAGATTTCATCGATTATGAGGAGCTAAGAGAGCAATTGAGCTCAGTGTCATCATTTGAAAGGTTTGAGATATTCCCCAAGACAAGTTCATGGCCCAATCATGACTCGAACAAAGGTGTAACGGCAGCATGTCCTCATGCTGGAGCAAAAAGCTTCTACAAAAATTTAATATGGCTAGTTAAAAAAGGAAATTCATACCCAAAGCTCAGCAAATCCTACATTAATGATAAAGGGAAAGAAGTCCTCGTGCTATGGGGCATTCATCATCCATCTACTAGTGCTGACCAACAAAGTCTCTATCAGAATGCAGATGCATATGTTTTTGTGGGGACATCAAGATACAGCAAGAAGTTCAAGCCGGAAATAGCAATAAGACCCAAAGTGAGGGATCAAGAAGGGAGAATGAACTATTACTGGACACTAGTAGAGCCGGGAGACAAAATAACATTCGAAGCAACTGGAAATCTAGTGGTACCGAGATATGCATTCGCAATGGAAAGAAATGCTGGATCTGGTATTATCATTTCAGATACACCAGTCCACGATTGCAATACAACTTGTCAGACACCCAAGGGTGCTATAAACACCAGCCTCCCATTTCAGAATATACATCCGATCACAATTGGAAAATGTCCAAAATATGTAAAAAGCACAAAATTGAGACTGGCCACAGGATTGAGGAATGTCCCGTCTATTCAATCTAGAGGCCTATTTGGGGCCATTGCCGGTTTCATTGAAGGGGGGTGGACAGGGATGGTAGATGGATGGTACGGTTATCACCATCAAAATGAGCAGGGGTCAGGATATGCAGCCGACCTGAAGAGCACACAGAATGCCATTGACGAGATTACTAACAAAGTAAATTCTGTTATTGAAAAGATGAATACACAGTTCACAGCAGTAGGTAAAGAGTTCAACCACCTGGAAAAAAGAATAGAGAATTTAAATAAAAAGGTTGATGATGGTTTCCTGGACATTTGGACTTACAATGCAGAACTGTTGGTTCTATTGGAAAATGAAAGAACTTTGGACTACCACGATTCAAATGTGAAGAACTTATATGAAAAGGTAAGAAGCCAGTTAAAAAACAATGCCAAGGAAATTGGAAACGGCTGCTTTGAATTTTACCACAAATGCGATAACACGTGCATGGAAAGTGTCAAAAATGGGACTTATGACTACCCAAAATACTCAGAGGAAGCAAAATTAAACAGAGAAGAAATAGATGGGGTAAAGCTGGAATCAACAAGGATTTACCAGATTTTGGCGATCTATTCAACTGTCGCCAGTTCATTGGTACTGGTAGTCTCCCTGGGGGCAATCAGTTTCTGGATGTGCTCTAATGGGTCTCTACAGTGTAGAATATGTATTTAA

>LC638179.1Influenza A virus H1N101701

ATGAAGGCAATACTAGTAGTTCTGCTATATACATTTGCAACCGCAAATGCAGACACATTATGTATAGGTTATCATGCGAACAATTCAACAGACACTGTAGACACAGTACTAGAAAAGAATGTAACAGTAACACACTCTGTTAACCTTCTAGAAGACAAGCATAACGGGAAACTATGCAAACTAAGAGGGGTAGCCCCATTGCATTTGGGTAAATGTAACATTGCTGGCTGGATCCTGGGAAATCCAGAGTGTGAATCACTCTCCACAGCAAGCTCATGGTCCTACATTGTGGAAACATCTAGTTCAGACAATGGACCGTGTTACCCAGGAGATTTCATCGATTATGAGGAGCTAAGAGAGCAATTGAGCTCAGTGTCATCATTTGAAAGGTTTGAGATATTCCCCAAGACAAGTTCATGGCCCAATCATGACTCGAACAAAGGTGTAACGGCAGCATGTCCTCATGCTGGGGCAAAAAGCTTCTACAAAAATTTAATATGGCTAGTTAAAAAAGGAAATTCATACCCAAAGCTCAGCAAATCCTACATTAATGATAAAGGGAAAGAAGTCCTCGTGCTATGGGGCATTCACCATCCATCTACTAGTGCTGACCAACAAAGTCTCTATCAGAATGCAGATGCATATGTTTTTGTGGGGACATCAAGATACAGCAAGAAGTTCAAGCCGGAAATAGCAATAAGACCCAAAGTGAGGGATCAAGAAGGGAGAATGAACTATTACTGGACACTAGTAGAGCCGGGAGACAAAATAACATTCGAAGCAACTGGAAATCTAGTGGTACCGAGATATGCATTCGCAATGGAAAGAAATGCTGGATCTGGTATTATCATTTCAGATACACCAGTCCACGATTGCAATACAACTTGTCAGACACCCAAGGGTGCTATGAACACCAGCCTCCCATTTCAGAATATACATCCGATCACAATTGGAAAATGTCCAAAATATGTAAAAAGCACAAAATTGAGACTGGCCACAGGATTGAGGAATGTCCCGTCTATTCAATCTAGAGGCCTATTTGGGGCCATTGCCGGTTTCATTGAAGGGGGGTGGACAGGGATGGTAGATGGATGGTACGGTTATCACCATCAAAATGAGCAGGGGTCAGGATATGCAGCCGACTTGAAGAGCACACAGAATGCCATTGACGAGATTACTAACAAAGTAAATTCTGTTATTGAAAAGATGAATACACAGTTCACAGCAGTAGGTAAAGAGTTCAACCACCTGGAAAAAAGAATAGAGAATTTAAATAAAAAGGTTGATGATGGTTTCCTGGACATTTGGACTTACAATGCAGAACTGTTGGTTCTATTGGAAAATGAAAGAACTTTGGACTACCACGATTCAAATGTGAAGAACTTATATGAAAAGGTAAGAAGCCAGTTAAAAAACAATGCCAAGGAAATTGGAAACGGCTGCTTTGAATTTTACCACAAATGCGATAACACGTGCATGGAAAGTGTCAAAAATGGGACTTATGACTACCCAAAATACTCAGAGGAAGCAAAATTAAACAGAGAAGAAATAGATGGGGTAAAGCTGGAATCAACAAGGATTTACCAGATTTTGGCGATCTATTCAACTGTCGCCAGTTCATTGATACTGGTAGTCTCCCTGGGGGCAATCAGTTTCTGGATGTGCTCTAATGGGTCTCTACAGTGTAGAATATGTATTTAA

>LC638178.1Influenza A virus H1N101701

ATGAAGGCAATACTGGTAGTTCTGCTATATACATTTGCAACCGCAAATGCAGACACATTATGTATAGGTTATCATGCGAACAATTCAACAGACACTGTAGACACAGTATTAGAAAAGAATGTAACAGTAACACACTCTGTTAACCTTCTAGAAGACAAGCATAACGGGAAACTATGCAAACTAAGAGGGGTAGCCCCATTGCATTTGGGTAAATGTAACATTGCTGGCTGGATCCTGGGAAATCCAGAGTGTGAATCACTCTCCACAGCAAGCTCATGGTCCTACATTGTGGAAACATCTAGTTCAGACAATGGAACGTGTTACCCAGGAGATTTCATCGATTATGAGGAGCTAAGAGAGCAATTGAGCTCAGTGTCATCATTTGAAAGGTTTGAGATATTCCCCAAGACAAGTTCATGGCCCAATCATGACTCGAACAAAGGTGTAACGGCAGCATGTCCTCATGCTGGAGCAAAAAGCTTCTACAAAAATTTAATATGGCTAGTTAAAAAAAGAAATTCATACCCAAAGCTCAGCAAATCCTACATTAATGATAAAGGGAAAGAAGTCCTCGTGCTATGGGGCATTCACCATCCATCTACTAGTGCTGACCAACAAAGTCTCTATCAGAATGCAGATACATATGTTTTTGTGGGGACATCAAGATACAGCAAGAAGTTCAAGCCGGAAATAGCAATAAGACCCAAAGTGAGGGATCAAGAAGGGAGAATGAACTATTACTGGACACTAGTAGAGCCGGGAGACAAAATAACATTCGAAGCAACTGGAAATCTAGTGGTACCGAGATATGCATTCGCAATGGAAAGAAATGCTGGATCTGGTATTATCATTTCAGATACACCAGTCCACGATTGCAATACAACTTGTCAGACACCCAAGGGTGCTATAAACACCAGCCTCCCATTTCAGAATATACATCCGATCACAATTGGAAAATGTCCAAAATATGTAAAAAGCACAAAATTGAGACTGGCCACAGGATTGAGGAATGTCCCGTCTATTCAATCTAGAGGCCTATTTGGGGCCATTGCCGGTTTCATTGAAGGGGGGTGGACAGGGATGGTAGATGGATGGTACGGTTATCACCATCAAAATGAGCAGGGGTCAGGATATGCAGCCGACCTGAAGAGCACACAGAATGCCATTGACGAGATTACTAACAAAGTAAATTCTGTTATTGAAAAGATGAATACACAGTTCACAGCAGTAGGTAAAGAGTTCAACCACCTGGAAAAAAGAATAGAGAATTTAAATAAAAAAGTTGATGATGGTTTCCTGGACATTTGGACTTACAATGCCGAACTGTTGGTTCTATTGGAAAATGAAAGAACTTTGGACTACCACGATTCAAATGTGAAGAACTTATATGAAAAGGTAAGAAGCCAGTTAAAAAACAATGCCAAGGAAATTGGAAACGGCTGCTTTGAATTTTACCACAAATGCGATAACACGTGCATGGAAAGTGTCAAAAATGGGACTTATGACTACCCAAAATACTCAGAGGAAGCAAAATTAAACAGAGAAGAAATAGATGGAGTAAAGCTGGAATCAACAAGGATTTACCAGATTTTGGCGATCTATTCAACTGTCGCCAGTTCATTGGTACTGGTAGTCTCCCTGGGGGCAATCAGTTTCTGGATGTGCTCTAATGGGTCTCTACAGTGTAGAATATGTATTTAA

>LC638177.1Influenza A virus H1N101701

ATGAAGGCAATACTAGTAGTTCTGCTATATACATTTGCAACCGCAAATGCAGACACATTATGTATAGGTTATCATGCGAACAATTCAACAGACACTGTAGACACAATACTAGAAAAGAATGTAACAGTAACACACTCTGTTAACCTTCTAGAAGACAAGCATAACGGGAAACTATGCAAACTAAGAGGGGTAGCCCCATTGCATTTGGGTAAATGTAACATTGCTGGCTGGATCCTGGGAAATCCAGAGTGTGAATCACTCTCCACAGCAAGCTCATGGTCCTACATTGTGGAAACATCTAGTTCAGACAATGGAACGTGTTACCCAGGAGATTTCATCGATTATGAGGAGCTAAGAGAGCAATTGAGCTCAGTGTCATCATTTGAAAGGTTTGAGATATTCCCCAAGACAAGTTCATGGCCCAATCATGACTCGAACAAAGGTGTAACGGCAGCATGTCCTCATGCTGGAGCAAAAAGCTTCTACAAAAATTTAATATGGCTAGTTAAAAAAGGAAATTCATACCCAAAGCTCAGCAAATCCTACATTAATGATAAAGGGAAAGAAGTCCTCGTGCTATGGGGCATTCACCATCCATCTACTAGTGCTGACCAACAAAGTCTCTATCAGAATGCAGATGCATATGTTTTTGTGGGGACATCAAGATACAGCAAGAAGTTCAAGCCGGAAATAGCAATAAGACCCAAAGTGAGGGATCAAGAAGGGAGAATGAACTATTACTGGACACTAGTAGAGCCGGGAGACAAAATAACATTCGAAGCAACTGGAAATCTAGTGGTACCGAGATATGCATTCGCAATGGAAAGAAATGCTGGATCTGGTATTATCATTTCAGATACACCAGTCCACGATTGCAATACAACTTGTCAGACACCCAAGGGTGCTATAAACACCAGCCTCCCATTTCAGAATATACATCCGATCACAATTGGAAAATGTCCAAAATATGTAAAAAGCACAAAATTGAGACTGGCCACAGGATTGAGGAATGTCCCGTCTATTCAATCTAGAGGCCTATTTGGGGCCATTGCCGGTTTCATTGAAGGGGGGTGGACAGGGATGGTAGATGGATGGTACGGTTATCACCATCAAAATGAGCAGGGGTCAGGATATGCAGCCGACCTGAAGAGCACACAGAATGCCATTGACGAGATTACTAACAAGGTAAATTCTGTTATTGAAAAGATGAATACACAGTTCACAGCAGTAGGTAAAGAGTTCAACCACCTGGAAAAAAGAATAGAGAATTTAAATAAAAAGGTTGATGATGGTTTCCTGGACATTTGGACTTACAATGCCGAACTGTTGGTTCTATTGGAAAATGAAAGAACTTTGGACTACCACGATTCAAATGTGAAGAACTTATATGAAAAGGTAAGAAGCCAGTTAAAAAACAATGCCAAGGAAATTGGAAACGGCTGCTTTGAATTTTACCACAAATGCGATAACACGTGCATGGAAAGTGTCAAAAATGGGACTTATGACTACCCAAAATACTCAGAGGAAGCAAAATTAAACAGAGAAAAAATAGATGGGGTAAAGCTGGAATCAACAAGGATTTACCAGATTTTGGCGATCTATTCAACTGTCGCCAGTTCATTGGTACTGGTAGTCTCCCTGGGGGCAATCAGTTTCTGGATGTGCTCTAATGGGTCTCTACAGTGTAGAATATGTATTTAA

>LC638176.1Influenza A virus H1N101701

ATGAAGACAATACTAGTAGTTCTGCTATATACATTTGCAACCGCAAATGCAGACACATTATGTATAGGTTATCATGCGAACAATTCAACAGACACTGTAGACACAATACTAGAAAAGAATGTAACAGTAACACACTCTGTTAACCTTCTAGAAGACAAGCATAACGGGAAACTATGCAAACTAAGAGGGGTAGCCCCATTGCATTTGGGTAAATGTAACATTGCTGGCTGGATCCTGGGAAATCCAGAGTGTGAATCACTCTCCACAGCAAGCTCATGGTCCTACATTGTGGAAACATCTAGTTCAGACAATGGAACGTGTTACCCAGGAGATTTCATCGATTATGAGGAGCTAAGAGAGCAATTGAGCTCAGTGTCATCATTTGAAAGGTTTGAGATATTCCCCAAGACAAGTTCATGGCCCAATCATGACTCGAACAAAGGTGTAACGGCAGCATGTCCTCATGCTGGAGCAAAAAGCTTCTACAAAAATTTAATATGGCTAGTTAAAAAAGGAAATTCATACCCAAAGCTCAGCAAATCCTACATTAATGATAAAGGGAAAGAAGTCCTCGTGCTATGGGGCATTCACCATCCATCTACTAGTGCTGACCAACAAAGTCTCTATCAGAATGCAGATGCATATGTTTTTGTGGGGACATCAAGATACAGCAAGAAGTTCAAGCCGGAAATAGCAATAAGACCCAAAGTGAGGGATCAAGAAGGGAGAATGAACTATTACTGGACACTAGTAGAGCCGGGAGACAAAATAACATTCGAAGCAACTGGAAATCTAGTGGTACCGAGATATGCATTCGCAATGGAAAGAAATGCTGGATCTGGTATTATCATTTCAGATACACCAGTCCACGATTGCAATACAACTTGTCAGACACCCAAGGGTGCTATAAACACCAGCCTCCCATTTCAGAATATACATCCGATCACAATTGGAAAATGTCCAAAATATGTAAAAAGCACAAAATTGAGACTGGCCACAGGATTGAGGAATGTCCCGTCTATTCAATCTAGAGGCCTATTTGGGGCCATTGCCGGTTTCATTGAAGGGGGGTGGACAGGGATGGTAGATGGATGGTACGGTTATCACCATCAAAATGAGCAGGGGTCAGGATATGCAGCCGACCTGAAGAGCACACAGAATGCCATTGACGAGATTACTAACAAAGTAAATTCTGTTATTGAAAAGATGAATACACAGTTCACAGCAGTAGGTAAAGAGTTCAACCACCTGGAAAAAAGAATAGAGAATTTAAATAAAAAGGTTGATGATGGTTTCCTGGACATTTGGACTTACAATGCCGAACTGTTGGTTCTATTGGAAAATGAAAGAACTTTGGACTACCACGATTCAAATGTGAAGAACTTATATGAAAAGGTAAGAAGCCAGTTAAAAAACAATGCCAAGGAAATTGGAAACGGCTGCTTTGAATTTTACCACAAATGCGATAACACGTGCATGGAAAGTGTCAAAAATGGGACTTATGACTACCCAAAATACTCAGAGGAAGCAAAATTAAACAGAGAAGAAATAGATGGGGTAAAGCTGGAATCAACAAGGATTTACCAGATTTTGGCGATCTATTCAACTGTCGCCAGTTCATTGGTACTGGTAGTCTCCCTGGGGGCAATCAGTTTCTGGATGTGCTCTAATGGGTCTCTACAGTGTAGAATATGTATTTAA

>LC638175.1Influenza A virus H1N101701

ATGAAGGCAATACTAGTAGTTCTGCTATATACATTTGCAACCGCAAATGCAGACACATTATGTATAGGTTATCATGCGAACAATTCAACAGACACTGTAGACACAATACTAGAAAAGAATGTAACAGTAACACACTCTGTTAACCTTCTAGAAGACAAGCATAACGGGAAACTATGCAAACTAAGAGGGGTAGCCCCATTGCATTTGGGTAAATGTAACATTGCTGGCTGGATCCTGGGAAATCCAGAGTGTGAATCACTCTCCACAGCAAGCTCATGGTCCTACATTGTGGAAACATCTAGTTCAGACAATGGAACGTGTTACCCAGGAGATTTCATCGATTATGAGGAGCTAAGAGAGCAATTGAGCTCAGTGTCATCATTTGAAAGGTTTGAGATATTCCCCAAGACAAGTTCATGGCCCAATCATGACTCGAACAAAGGTGTAACGGCAGCATGTCCTCATGCTGGAGCAAAAAGCTTCTACAAAAATTTAATATGGCTAGTTAAAAAAGGAAATTCATACCCAAAGCTCAGCAAATCCTACATTAATGATAAAGGGAAAGAAGTCCTCGTGCTATGGGGCATTCACCATCCATCTACTAGTGCTGACCAACAAAGTCTCTATCAGAATGCAGATGCATATGTTTTTGTGGGGACATCAAGATACAGCAAGAAGTTCAAGCCGGAAATAGCAATAAGACCCAAAGTGAGGGATCAAGAAGGGAGAATGAACTATTACTGGACACTAGTAGAGCCGGGAGACAAAATAACATTCGAAGCAACTGGAAATCTAGTGGTACCGAGATATGCATTCGCAATGGAAAGAAATGCTGGATCTGGTATTATCATTTCAGATACACCAGTCCACGATTGCAATACAACTTGTCAGACACCCAAGGGTGCTATAAACACCAGCCTCCCATTTCAGAATATACATCCGATCACAATTGGAAAATGTCCAAAATATGTAAAAAGCACAAAATTGAGACTGGCCACAGGATTGAGGAATGTCCCGTCTATTCAATCTAGAGGCCTATTTGGGGCCATTGCCGGTTTCATTGAAGGGGGGTGGACAGGGATGGTAGATGGATGGTACGGTTATCACCATCAAAATGAGCAGGGGTCAGGATATGCAGCCGACCTGAAGAGCACACAGAATGCCATTGACGAGATTACTAACAAAGTAAATTCTGTTATTGAAAAGATGAATACACAGTTCACAGCAGTAGGTAAAGAGTTCAACCACCTGGAAAAAAGAATAGAGAATTTAAATAAAAAGGTTGATGATGGTTTCCTGGACATTTGGACTTACAATGCCGAACTGTTGGTTCTATTGGAAAATGAAAGAACTTTGGACTACCACGATTCAAATGTGAAGAACTTATATGAAAAGGTAAGAAGCCAGTTAAAAAACAATGCCAAGGAAATTGGAAACGGCTGCTTTGAATTTTACCACAAATGCGATAACACGTGCATGGAAAGTGTCAAAAATGGGACTTATGACTACCCAAAATACTCAGAGGAAGCAAAATTAAACAGAGAAGAAATAGATGGGGTAAAGCTGGAATCAACAAGGATTTACCAGATTTTGGCGATCTATTCAACTGTCGCCAGTTCATTGGTACTGGTAGTCTCCCTGGGGGCAATCAGTTTCTGGATGTGCTCTAATGGGTCTCTACAGTGTAGAATATGTATTTAA

>LC638174.1Influenza A virus H1N101701

ATGAAGGCAATACTAGTAGTTCTGCTATATACATTTGCAACCGCAAATGCAGACACATTATGTATAGGTTATCATGCGAACAATTCAACAGACACTGTAGACACAGTACTAGAAAAGAATGTAACAGTAACACATTCTGTTAACCTTCTAGAAGACAAGCATAACGGGAAACTATGCAAACTAAGAGGGGTAGCCCCATTGCATTTGGGTAAATGTAACATTGCTGGCTGGATCCTGGGAAATCCAGAGTGTGAATCACTCTCCACAGCAAGCTCATGGTCCTACATTGTGGAAACATCTAGTTCAGACAATGGAACGTGTTACCCAGGAGATTTCATCGATTATGAGGAGCTAAGAGAGCAATTGAGCTCAGTGTCATCATTTGAAAGGTTTGAGATATTCCCCAAGACAAGTTCATGGCCCAATCATGACTCGAACAAAGGTGTAACGGCAGCATGTCCTCATGCTGGAGCAAAAAGCTTCTACAAAAATTTAATATGGCTAGTTAAAAAAGGAAATTCATACCCAAAGCTCAGCAAATCCTACATTAATGATAAAGGGAAAGAAGTCCTCGTGCTATGGGGCATTCATCATCCATCTACTAGTGCTGACCAACAAAGTCTCTATCAGAATGCAGATGCATATGTTTTTGTGGGGACATCAAGATACAGCAAGAAGTTCAAGCCGGAAATAGCAATAAGACCCAAAGTGAGGGATCAAGAAGGGAGAATGAACTATTACTGGACACTAGTAGAGCCGGGAGACAAAATAACATTCGAAGCAACTGGAAATCTAGTGGTACCGAGATATGCATTCGCAATGGAAAGAAATGCTGGATCTGGTATTATCATTTCAGATACACCAGTCCACGATTGCAATACAACTTGTCAGACACCCAAGGGTGCTATAAACACCAGCCTCCCATTTCAGAATATACATCCGATCACAATTGGAAAATGTCCAAAATATGTAAAAAGCACAAAATTGAGACTGGCCACAGGATTGAGGAATGTCCCGTCTATTCAATCTAGAGGCCTATTTGGGGCCATTGCCGGTTTCATTGAAGGGGGGTGGACAGGGATGGTAGATGGATGGTACGGTTATCACCATCAAAATGAGCAGGGGTCAGGATATGCAGCCGACCTGAAGAGCACACAGAATGCCATTGACGAGATTACTAACAAAGTAAATTCTGTTATTGAAAAGATGAATACACAGTTCACAGCAGTAGGTAAAGAGTTCAACCACCTGGAAAAAAGAATAGAGAATTTAAATAAAAAGGTTGATGATGGTTTCCTGGACATTTGGACTTACAATGCAGAACTGTTGGTTCTATTGGAAAATGAAAGAACTTTGGACTACCACGATTCAAATGTGAAGAACTTATATGAAAAGGTAAGAAGCCAGTTAAAAAACAATGCCAAGGAAATTGGAAACGGCTGCTTTGAATTTTACCACAAATGCGATAACACGTGCATGGAAAGTGTCAAAAATGGGACTTATGACTACCCAAAATACTCAGAGGAAGCAAAATTAAACAGAGAAGAAATAGATGGGGTAAAGCTGGAATCAACAAGGATTTACCAGATTTTGGCGATCTATTCAACTGTCGCCAGTTCATTGGTACTGGTAGTCTCCCTGGGGGCAATCAGTTTCTGGATGTGCTCTAATGGGTCTCTACAGTGTAGAATATGTATTTAA

>LC638173.1Influenza A virus H1N101701

ATGAAGGCAATACTAGTAGTTCTGCTATATACATTTGCAACCGCAAATGCAGACACATTATGTATAGGTTATCATGCGAACAATTCAACAGACACTGTAGACACAGTACTAGAAAAGAATGTAACAGTAACACATTCTGTTAACCTTCTAGAAGACAAGCATAACGGGAAACTATGCAAACTAAGAGGGGTAGCCCCATTGCATTTGGGTAAATGTAACATTGCTGGCTGGATCCTGGGAAATCCAGAGTGTGAATCACTCTCCACAGCAAGCTCATGGTCCTACATTGTGGAAACATCTAGTTCAGACAATGGAACGTGTTACCCAGGAGATTTCATCGATTATGAGGAGCTAAGAGAGCAATTGAGCTCAGTGTCATCATTTGAAAGGTTTGAGATATTCCCCAAGACAAGTTCATGGCCCAATCATGACTCGAACAAAGGTGTAACGGCAGCATGTCCTCATGCTGGAGCAAAAAGCTTCTACAAAAATTTAATATGGCTAGTTAAAAAAGGAAATTCATACCCAAAGCTCAGCAAATCCTACATTAATGATAAAGGGAAAGAAGTCCTCGTGCTATGGGGCATTCATCATCCATCTACTAGTGCTGACCAACAAAGTCTCTATCAGAATGCAGATGCATATGTTTTTGTGGGGACATCAAGATACAGCAAGAAGTTCAAGCCGGAAATAGCAATAAGACCCAAAGTGAGGGATCAAGAAGGGAGAATGAACTATTACTGGACACTAGTAGAGCCGGGAGACAAAATAACATTCGAAGCAACTGGAAATCTAGTGGTACCGAGATATGCATTCGCAATGGAAAGAAATGCTGGATCTGGTATTATCATTTCAGATACACCAGTCCACGATTGCAATACAACTTGTCAGACACCCAAGGGTGCTATAAACACCAGCCTCCCATTTCAGAATATACATCCGATCACAATTGGAAAATGTCCAAAATATGTAAAAAGCACAAAATTGAGACTGGCCACAGGATTGAGGAATGTCCCGTCTATTCAATCTAGAGGCCTATTTGGGGCCATTGCCGGTTTCATTGAAGGGGGGTGGACAGGGATGGTAGATGGATGGTACGGTTATCACCATCAAAATGAGCAGGGGTCAGGATATGCAGCCGACCTGAAGAGCACACAGAATGCCATTGACGAGATTACTAACAAAGTAAATTCTGTTATTGAAAAGATGAATACACAGTTCACAGCAGTAGGTAAAGAGTTCAACCACCTGGAAAAAAGAATAGAGAATTTAAATAAAAAGGTTGATGATGGTTTCCTGGACATTTGGACTTACAATGCAGAACTGTTGGTTCTATTGGAAAATGAAAGAACTTTGGACTACCACGATTCAAATGTGAAGAACTTATATGAAAAGGTAAGAAGCCAGTTAAAAAACAATGCCAAGGAAATTGGAAACGGCTGCTTTGAATTTTACCACAAATGCGATAACACGTGCATGGAAAGTGTCAAAAATGGGACTTATGACTACCCAAAATACTCAGAGGAAGCAAAATTAAACAGAGAAGAAATAGATGGGGTAAAGCTGGAATCAACAAGGATTTACCAGATTTTGGCGATCTATTCAACTGTCGCCAGTTCATTGGTACTGGTAGTCTCCCTGGGGGCAATCAGTTTCTGGATGTGCTCTAATGGGTCTCTACAGTGTAGAATATGTATTTAA

>LC638172.1Influenza A virus H1N101701

ATGAAGGCAATACTAGTAGTTCTGCTATATACATTTGCAACCGCAAATGCAGACACATTATGTATAGGTTATCATGCGAACAATTCAACAGACACTGTAGACACAATACTAGAAAAGAATGTAACAGTAACACACTCTGTTAACCTTCTAGAAGACAAGCATAACGGGAAACTATGCAAACTAAGAGGGGTAGCCCCATTGCATTTGGGTAAATGTAACATTGCTGGCTGGATCCTGGGAAATCCAGAGTGTGAATCACTCTCCACAGCAAGCTCATGGTCCTACATTGTGGAAACATCTAGTTCAGACAATGGAACGTGTTACCCAGGAGATTTCATCGTTTATGAGGAGCTAAGAGAGCAATTGAGCTCAGTGTCATCATTTGAAAGGTTTGAGATATTCCCCAAGACAAGTTCATGGCCCAATCATGACTCGAACAAAGGTGTAACGGCAGCATGTCCTCATGCTGGAGCAAAAAGCTTCTACAAAAATTTAATATGGCTAGTTAAAAAAGGAAATTCATACCCAAAGCTCAGCAAATCCTACATTAATGATAAAGGGAAAGAAGTCCTCGTGCTATGGGGCATTCACCATCCATCTACTAGTGCTGACCAACAAAGTCTCTATCAGAATGCAGATGCATATGTTTTTGTGGGGACATCAAGATACAGCAAGAAGTTCAAGCCGGAAATAGCAATAAGACCCAAAGTGAGGGATCAAGAAGGGAGAATGAACTATTACTGGACACTAGTAGAGCCGGGAGACAAAATAACATTCGAAGCAACTGGAAATCTAGTGGTACCGAGATATGCATTCGCAATGGAAAGAAATGCTGGATCTGGTATTATCATTTCAGATACACCAGTCCACGATTGCAATACAACTTGTCAGACACCCAAGGGTGCTATAAACACCAGCCTCCCATTTCAGAATATACATCCGATCACAATTGGAAAATGTCCAAAATATGTAAAAAGCACAAAATTGAGACTGGCCACAGGATTGAGGAATGTCCCGTCTATTCAATCTAGAGGCCTATTTGGGGCCATTGCCGGTTTCATTGAAGGGGGGTGGACAGGGATGGTAGATGGATGGTACGGTTATCACCATCAAAATGAGCAGGGGTCAGGATATGCAGCCGACCTGAAGAGCACACAGAATGCCATTGACGAGATTACTAACAAAGTAAATTCTGTTATTGAAAAGATGAATACACAGTTCACAGCAGTAGGTAAAGAGTTCAACCACCTGGAAAAAAGAATAGAGAATTTAAATAAAAAGGTTGATGATGGTTTCCTGGACATTTGGACTTACAATGCCGAACTGTTGGTTCTATTGGAAAATGAAAGAACTTTGGACTACCACGATTCAAATGTGAAGAACTTATATGAAAAGGTAAGAAGCCAGTTAAAAAACAATGCCAAGGAAATTGGAAACGGCTGCTTTGAATTTTACCACAAATGCGATAACACGTGCATGGAAAGTGTCAAAAATGGGACTTATGACTACCCAAAATACTCAGAGGAAGCAAAATTAAACAGAGAAGAAATAGATGGGGTAAAGCTGGAATCAACAAGGATTTACCAGATTTTGGCGATCTATTCAACTGTCGCCAGTTCATTGGTACTGGTAGTCTCCCTGGGGGCAATCAGTTTCTGGATGTGCTCTAATGGGTCTCTACAGTGTAGAATATGTATTTAA

>LC638169.1Influenza A virus H1N101701

ATGAAGGCAATACTAGTAGTTCTGCTATATACATTTGCAACCGCAAATGCAGACACATTATGTATAGGTTATCATGCGAACAATTCAACAGACACTGTAGACACAGTACTAGAAAAGAATGTAACAGTAACACATTCTGTTAACCTTCTAGAAGACAAGCATAACGGGAAACTATGCAAACTAAGAGGGGTAGCCCCATTGCATTTGGGTAAATGTAACATTGCTGGCTGGATCCTGGGAAATCCAGAGTGTGAATCACTCTCCACAGCAAGCTCATGGTCCTACATTGTGGAAACATCTAGTTCAGACAATGGAACGTGTTACCCAGGAGATTTCATCGATTATGAGGAGCTAAGAGAACAATTGAGCTCAGTGTCATCATTTGAAAGGTTTGAGATATTCCCCAAGACAAGTTCATGGCCCAATCATGACTCGAACAAAGGTGTAACGGCAGCATGTCCTCATGCTGGAGCAAAAAGCTTCTACAAAAATTTAATATGGCTAGTTAAAAAAGGAAATTCATACCCAAAGCTCAGCAAATCCTACATTAATGATAAAGGGAAAGAAGTCCTCGTGCTATGGGGCATTCATCATCCATCTACTAGTGCTGACCAACAAAGTCTCTATCAGAATGCAGATGCATATGTTTTTGTGGGGACATCAAGATACAGCAAGAAGTTCAAGCCGGAAATAGCAATAAGACCCAAAGTGAGGGATCAAGAAGGGAGAATGAACTATTACTGGACACTAGTAGAGCCGGGAGACAAAATAACATTCGAAGCAACTGGAAATCTAGTGGTACCGAGATATGCATTCGCAATGGAAAGAAATGCTGGATCTGGTATTATCATTTCAGATACACCAGTCCACGATTGCAATACAACTTGTCAGACACCCAAGGGTGCTATAAACACCAGCCTCCCATTTCAGAATATACATCCGATCACAATTGGAAAATGTCCAAAATATGTAAAAAGCACAAAATTGAGACTGGCCACAGGATTGAGGAATGTCCCGTCTATTCAATCTAGAGGCCTATTTGGGGCCATTGCCGGTTTCATTGAAGGGGGGTGGACAGGGATGGTAGATGGATGGTACGGTTATCACCATCAAAATGAGCAGGGGTCAGGATATGCAGCCGACCTGAAGAGCACACAGAATGCCATTGACGAGATTACTAACAAAGTAAATTCTGTTATTGAAAAGATGAATACACAGTTCACAGCAGTAGGTAAAGAGTTCAACCACCTGGAAAAAAGAATAGAGAATTTAAATAAAAAGGTTGATGATGGTTTCCTGGACATTTGGACTTACAATGCAGAACTGTTGGTTCTATTGGAAAATGAAAGAACTTTGGACTACCACGATTCAAATGTGAAGAACTTATATGAAAAGGTAAGAAGCCAGTTAAAAAACAATGCCAAGGAAATTGGAAACGGCTGCTTTGAATTTTACCACAAATGCGATAACACGTGCATGGAAAGTGTCAAAAATGGGACTTATGACTACCCAAAATACTCAGAGGAAGCAAAATTAAACAGAGAAGAAATAGATGGGGTAAAGCTGGAATCAACAAGGATTTACCAGATTTTGGCGATCTATTCAACTGTCGCCAGTTCATTGGTACTGGTAGTCTCCCTGGGGGCAATCAGTTTCTGGATGTGCTCTAATGGGTCTCTACAGTGTAGAATATGTATTTAA

>LC638168.1Influenza A virus H1N101701

ATGAAGGCAATACTAGTAGTTCTGCTATATACATTTGCAACCGCAAATGCAGACACATTATGTATAGGTTATCATGCGAACAATTCAACAGACACTGTAGACACAGTACTAGAAAAGAATGTAACAGTAACACATTCTGTTAACCTTCTAGAAGACAAGCATAACGGGAAACTATGCAAACTAAGAGGGGTAGCCCCATTGCATTTGGGTAAATGTAACATTGCTGGCTGGATCCTGGGAAATCCAGAGTGTGAATCACTCTCCACAGCAAGCTCATGGTCCTACATTGTGGAAACATCTAGTTCAGACAATGGAACGTGTTACCCAGGAGATTTCATCGATTATGAGGAGCTAAGAGAGCAATTGAGCTCAGTGTCATCATTTGAAAGGTTTGAGATATTCCCCAAGACAAGTTCATGGCCCAATCATGACTCGAACAAAGGTGTAACGGCAGCATGTCCTCATGCTGGAGCAAAAAGCTTCTACAAAAATTTAATATGGCTAGTTAAAAAAGGAAATTCATACCCAAAGCTCAGCAAATCCTACATTAATGATAAAGGGAAAGAAGTCCTAGTGCTATGGGGCATTCATCATCCATCTACTAGTGCTGACCAACAAAGTCTCTATCAGAATGCAGATGCATATGTTTTTGTGGGGACATCAAGATACAGCAAGAAGTTCAAGCCGGAAATAGCAATAAGACCCAAAGTGAGGGATCAAGAAGGGAGAATGAACTATTACTGGACACTAGTAGAGCCGGGAGACAAAATAACATTCGAAGCAACTGGAAATCTAGTGGTACCGAGATATGCATTCGCAATGGAAAGAAATGCTGGATCTGGTATTATCATTTCAGATACACCAGTCCACGATTGCAATACAACTTGTCAGACACCCAAGGGTGCTATAAACACCAGCCTCCCATTTCAGAATATACATCCGATCACAATTGGAAAATGTCCAAAATATGTAAAAAGCACAAAATTGAGACTGGCCACAGGATTGAGGAATGTCCCGTCTATTCAATCTAGAGGCCTATTTGGGGCCATTGCCGGTTTCATTGAAGGGGGGTGGACAGGGATGGTAGATGGATGGTACGGTTATCACCATCAAAATGAGCAGGGGTCAGGATATGCAGCCGACCTGAAGAGCACACAGAATGCCATTGACGAGATTACTAACAAAGTAAATTCTGTTATTGAAAAGATGAATACACAGTTCACAGCAGTAGGTAAAGAGTTCAACCACCTGGAAAAAAGAATAGAGAATTTAAATAAAAAGGTTGATGATGGTTTCCTGGACATTTGGACTTACAATGCAGAACTGTTGGTTCTATTGGAAAATGAAAGAACTTTGGACTACCACGATTCAAATGTGAAGAACTTATATGAAAAGGTAAGAAGCCAGTTAAAAAACAATGCCAAGGAAATTGGAAACGGCTGCTTTGAATTTTACCACAAATGCGATAACACGTGCATGGAAAGTGTCAAAAATGGGACTTATGACCACCCAAAATACTCAGAGGAAGCAAAATTAAACAGAGAAGAAATAGATGGGGTAAAGCTGGAATCAACAAGGATTTACCAGATTTTGGCGATCTATTCAACTGTCGCCAGTTCATTGGTACTGGTAGTCTCCCTGGGGGCAATCAGTTTCTGGATGTGCTCTAATGGGTCTCTACAGTGTAGAATATGTATTTAA

>LC638165.1Influenza A virus H1N101701

ATGAAGGCAATACTAGTAGTTCTGCTATATACATTTGCAACCGCAAATGCAGACACATTATGTATAGGTTATCATGCGAACAATTCAACAGACACTGTAGACACAGTACTAGAAAAGAATGTAACAGTAACACATTCTGTTAACCTTCTAGAAGACAAGCATAACGGGAAACTATGCAAACTAAGAGGGGTAGCCCCATTGCATTTGGGTAAATGTAACATTGCTGGCTGGATCCTGGGAAATCCAGAGTGTGAATCACTCTCCACAGCAAGCTCATGGTCCTACATTGTGGAAACATCTAGTTCAGACAATGGAACGTGTTACCCAGGAGATTTCATCGATTATGAGGAGCTAAGAGAGCAATTGAGCTCAGTGTCATCATTTGAAAGGTTTGAGATATTCCCCAAGACAAGTTCATGGCCCAATCATGACTCGAACAAAGGTGTAACGGCAGCATGTCCTCATGCTGGAGCAAAAAGCTTCTACAAAAATTTAATATGGCTAGTTAAAAAAGGAAATTCATACCCAAAGCTCAGCAAATCCTACATTAATGATAAAGGGAAAGAAGTCCTCGTGCTATGGGGCATTCATCATCCATCTACTAGTGCTGACCAACAAAGTCTCTATCAGAATGCAGATGCATATGTTTTTGTGGGGACATCAAGATACAGCAAGAAGTTCAAGCCGGAAATAGCAATAAGACCCAAAGTGAGGGATCAAGAAGGGAGAATGAACTATTACTGGACACTAGTAGAGCCGGGAGACAAAATAACATTCGAAGCAACTGGAAATCTAGTGGTACCGAGATATGCATTCGCAATGGAAAGAAATGCTGGATCTGGTATTATCATTTCAGATACACCAGTCCACGATTGCAATACAACTTGTCAGACACCCAAGGGTGCTATAAACACCAGCCTCCCATTTCAGAATATACATCCGATCACAATTGGAAAATGTCCAAAATATGTAAAAAGCACAAAATTGAGACTGGCCACAGGATTGAGGAATGTCCCGTCTATTCAATCTAGAGGCCTATTTGGGGCCATTGCCGGTTTCATTGAAGGGGGGTGGACAGGGATGGTAGATGGATGGTACGGTTATCACCATCAAAATGAGCAGGGGTCAGGATATGCAGCCGACCTGAAGAGCACACAGAATGCCATTGACGAGATTACTAACAAAGTAAATTCTGTTATTGAAAAGATGAATACACAGTTCACAGCAGTAGGTAAAGAGTTCAACCACCTGGAAAAAAGAATAGAGAATTTAAATAAAAAGGTTGATGATGGTTTCCTGGACATTTGGACTTACAATGCAGAACTGTTGGTTCTATTGGAAAATGAAAGAACTTTGGACTACCACGATTCAAATGTGAAGAACTTATATGAAAAGGTAAGAAGCCAGTTAAAAAACAATGCCAAGGAAATTGGAAACGGCTGCTTTGAATTTTACCACAAATGCGATAACACGTGCATGGAAAGTGTCAAAAATGGGACTTATGACTACCCAAAATACTCAGAGGAAGCAAAATTAAACAGAGAAGAAATAGATGGGGTAAAGCTGGAATCAACAAGGATTTACCAGATTTTGGCGATCTATTCAACTGTCGCCAGTTCATTGGTACTGGTAGTCTCCCTGGGGGCAATCAGTTTCTGGATGTGCTCTAATGGGTCTCTACAGTGTAGAATATGTATTTAA

>LC638162.1Influenza A virus H1N101701

ATGAAGGCAATACTAGTAGTTCTGCTATATACATTTGCAACCGCAAATGCAGACACATTATGTATAGGTTATCATGCGAACAATTCAACAGACACTGTAGACACAATACTAGAAAAGAATGTAACAGTAACACACTCTGTTAACCTTCTAGAAGACAAGCATAACGGGAAACTATGCAAACTAAGAGGGGTAGCCCCATTGCATTTGGGTAAATGTAACATTGCTGGCTGGATCCTGGGAAATCCAGAGTGTGAATCACTCTCCACAGCAAGCTCATGGTCCTACATTGTGGAAACATCTAGTTCAGACAATGGAACGTGTTACCCAGGAGATTTCATCGATTATGAGGAGCTAAGAGAGCAATTGAGCTCAGTGTCATCATTTGAAAGGTTTGAGATATTCCCCAAGACAAGTTCATGGCCCAATCATGACTCGAACAAAGGTGTAACGGCAGCATGTCCTCATGCTGGAGCAAAAAGCTTCTACAAAAATTTAATATGGCTAGTTAAAAAAGGAAATTCATACCCAAAGCTCAGCAAATCCTACATTAATGATAAAGGGAAAGAAGTCCTCGTGCTATGGGGCATTCACCATCCATCTACTAGTGCTGACCAACAAAGTCTCTATCAGAATGCAGATGCATATGTTTTTGTGGGGACATCAAGATACAGCAAGAAGTTCAAGCCGGAAATAGCAATAAGACCCAAAGTGAGGGATCAAGAAGGGAGAATGAACTATTACTGGACACTAGTAGAGCCGGGAGACAAAATAACATTCGAAGCAACTGGAAATCTAGTGGTACCGAGATATGCATTCGCAATGGAAAGAAATGCTGGATCTGGTATTATCATTTCAGATACACCAGTCCACGATTGCAATACAACTTGTCAGACACCCAAGGGTGCTATAAACACCAGCCTCCCATTTCAGAATATACATCCGATCACAATTGGAAAATGTCCAAAATATGTAAAAAGCACAAAATTGAGACTGGCCACAGGATTGAGGAATGTCCCGTCTATTCAATCTAGAGGCCTATTTGGGGCCATTGCCGGTTTCATTGAAGGGGGGTGGACAGGGATGGTAGATGGATGGTACGGTTATCACCATCAAAATGAGCAGGGGTCAGGATATGCAGCCGACCTGAAGAGCACACAGAATGCCATTGACGAGATTACTAACAAGGTAAATTCTGTTATTGAAAAGATGAATACACAGTTCACAGCAGTAGGTAAAGAGTTCAACCACCTGGAAAAAAGAATAGAGAATTTAAATAAAAAGGTTGATGATGGTTTCCTGGACATTTGGACTTACAATGCCGAACTGTTGGTTCTATTGGAAAATGAAAGAACTTTGGACTACCACGATTCAAATGTGAAGAACTTATATGAAAAGGTAAGAAGCCAGTTAAAAAACAATGCCAAGGAAATTGGAAACGGCTGCTTTGAATTTTACCACAAATGCGATAACACGTGCATGGAAAGTGTCAAAAATGGGACTTATGACTACCCAAAATACTCAGAGGAAGCAAAATTAAACAGAGAAGAAATAGATGGGGTAAAGCTGGAATCAACAAGGATTTACCAGATTTTGGCGATCTATTCAACTGTCGCCAGTTCATTGGTACTGGTAGTCTCCCTGGGGGCAATCAGTTTCTGGATGTGCTCTAATGGGTCTCTACAGTGTAGAATATGTATTTAA

>LC638161.1Influenza A virus H1N101701

ATGAAGGCAATACTAGTAGTTCTGCTATATACATTTGCAATCGCAAATGCAGACACATTATGTATAGGTTATCATGCGAACAATTCAACAGACACTGTAGACACAATACTAGAAAAGAATGTAACAGTAACACACTCTGTTAACCTTCTAGAAGACAAGCATAACGGGAAACTATGCAAACTAAGAGGGGTAGCCCCATTGCATTTGGGTAAATGTAACATTGCTGGCTGGATCCTGGGAAATCCAGAGTGTGAATCACTCTCCACAGCAAGCTCATGGTCCTACATTGTGGAAACATCTAGTTCAGACAATGGAACGTGTTACCCAGGAGATTTCATCGATTATGAGGAGCTAAGAGAGCAATTGAGCTCAGTGTCATCATTTGAAAGGTTTGAGATATTCCCCAAGACAAGTTCATGGCCCAATCATGACTCGAACAAAGGTGTAACGGCAGCATGTCCTCATGCTGGAGCAAAAAGCTTCTACAAAAATTTAATATGGCTAGTTAAAAAAGGAAATTCATACCCAAAGCTCAGCAAATCCTACATTAATGATAAAGGGAAAGAAGTCCTCGTGCTATGGGGCATTCACCATCCATCTACTAGTGCTGACCAACAAAGTCTCTATCAGAATGCAGATGCATATGTTTTTGTGGGGACATCAAGATACAGCAAGAAGTTCAAGCCGGAAATAGCAATAAGACCCAAAGTGAGGGATCAAGAAGGGAGAATGAACTATTACTGGACACTAGTAGAGCCGGGAGACAAAATAACATTCGAAGCAACTGGAAATCTAGTGGTACCGAGATATGCATTCGCAATGGAAAGAAATGCTGGATCTGGTATTATCATTTCAGATACACCAGTCCACGATTGCAATACAACTTGTCAGACACCCAAGGGTGCTATAAACACCAGCCTCCCATTTCAGAATATACATCCGATCACAATTGGAAAATGTCCAAAATATGTAAAAAGCACAAAATTGAGACTGGCCACAGGATTGAGGAATGTCCCGTCTATTCAATCTAGAGGCCTATTTGGGGCCATTGCCGGTTTCATTGAAGGGGGGTGGACAGGGATGGTAGATGGATGGTACGGTTATCACCATCAAAATGAGCAGGGGTCAGGATATGCAGCCGACCTGAAGAGCACACAGAATGCCATTGACGAGATTACTAACAAGGTAAATTCTGTTATTGAAAAGATGAATACACAGTTCACAGCAGTAGGTAAAGAGTTCAACCACCTGGAAAAAAGAATAGAGAATTTAAATAAAAAGGTTGATGATGGTTTCCTGGACATTTGGACTTACAATGCCGAACTGTTGGTTCTATTGGAAAATGAAAGAACTTTGGACTACCACGATTCAAATGTGAAGAACTTATATGAAAAGGTAAGAAGCCAGTTAAAAAACAATGCCAAGGAAATTGGAAACGGCTGCTTTGAATTTTACCACAAATGCGATAACACGTGCATGGAAAGTGTCAAAAATGGGACTTATGACTACCCAAAATACTCAGAGGAAGCAAAATTAAACAGAGAAGAAATAGATGGGGTAAAGCTGGAATCAACAAGGATTTACCAGATTTTGGCGATCTATTCAACTGTCGCCAGTTCATTGGTACTGGTAGTCTCCCTGGGGGCAATCAGTTTCTGGATGTGCTCTAATGGGTCTCTACAGTGTAGAATATGTATTTAA

>LC638157.1Influenza A virus H1N101701

ATGAAGGCAATACTAGTAGTTCTGCTATATACATTTGCAACCGCAAATGCAGACACATTATGTATAGGTTATCATGCGAACAATTCAACAGACACTGTAGACACAATACTAGAAAAGAATGTAACAGTAACACACTCTGTTAACCTTCTAGAAGACAAGCATAACGGGAAACTATGCAAACTAAGAGGGGTAGCCCCATTGCATTTGGGTAAATGTAACATTGCTGGCTGGATCCTGGGAAATCCAGAGTGTGAATCACTCTCCACAGCAAGCTCATGGTCCTACATTGTGGAAACATCTAGTTCAGACAATGGAACGTGTTACCCAGGAGATTTCATCGATTATGAGGAGCTAAGAGAGCAATTGAGCTCAGTGTCATCATTTGAAAGGTTTGAGATATTCCCCAAGACAAGTTCATGGCCCAATCATGACTCGAACAAAGGTGTAACGGCAGCATGTCCTCATGCTGGAGCAAAAAGCTTCTACAAAAATTTAATATGGCTAGTTAAAAAAGGAAATTCATACCCAAAGCTCAGCAAATCCTACATTAATGATAAAGGGAAAGAAGTCCTCGTGCTATGGGGCATTCACCATCCATCTACTAGTGCTGACCAACAAAGTCTCTATCAGAATGCAGATGCATATGTTTTTGTGGGGACATCAAGATACAGCAAGAAGTTCAAGCCGGAAATAGCAATAAGACCCAAAGTGAGGGATCAAGAAGGGAGAATGAACTATTACTGGACACTAGTAGAGCCGGGAGACAAAATAACATTCGAAGCAACTGGAAATCTAGTGGTACCGAGATATGCATTCGCAATGGAAAGAAATGCTGGATCTGGTATTATCATTTCAGATACACCAGTCCACGATTGCAATACAACTTGTCAGACACCCAAGGGTGCTATAAACACCAGCCTCCCATTTCAGAATATACATCCGATCACAATTGGAAAATGTCCAAAATATGTAAAAAGCACAAAATTGAGACTGGCCACAGGATTGAGGAATGTCCCGTCTATTCAATCTAGAGGCCTATTTGGGGCCATTGCCGGTTTCATTGAAGGGGGGTGGACAGGGATGGTAGATGGATGGTACGGTTATCACCATCAAAATGAGCAGGGGTCAGGATATGCAGCCGACCTGAAGAGCACACAGAATGCCATTGACGAGATTACTAACAAAGTAAATTCTGTTATTGAAAAGATGAATACACAGTTCACAGCAGTAGGTAAAGAGTTCAACCACCTGGAAAAAAGAATAGAGAATTTAAATAAAAAGGTTGATGATGGTTTCCTGGACATTTGGACTTACAATGCCGAACTGTTGGTTCTATTGGAAAATGAAAGAACTTTGGACTACCACGATTCAAATGTGAAGAACTTATATGAAAAGGTAAGAAGCCAGTTAAAAAACAATGCCAAGGAAATTGGAAACGGCTGCTTTGAATTTTACCACAAATGCGATAACACGTGCATGGAAAGTGTCAAAAATGGGACTTATGACTACCCAAAATACTCAGAGGAAGCAAAATTAAACAGAGAAGAAATAGATGGGGTAAAGCTGGAATCAACAAGGATTTACCAGATTTTGGCGATCTATTCAACTGTCGCCAGTTCATTGGTACTGGTAGTCTCCCTGGGGGCAATCAGTTTCTGGATGTGCTCTAATGGGTCTCTACAGTGTAGAATATGTATTTAA

>LC638156.1Influenza A virus H1N101701

ATGAAGGCAATACTAGTAGTTCTGCTATATACATTTGCAACCGCAAATGCAGACACATTATGTATAGGTTATCATGCGAACAATTCAACAGACACTGTAGACACAGTACTAGAAAAGAATGTAACAGTAACACATTCTGTTAACCTTCTAGAAGACAAGCATAACGGGAAACTATGCAAACTAAGAGGGGTAGCCCCATTGCATTTGGGTAAATGTAACATTGCTGGCTGGATCCTGGGAAATCCAGAGTGTGAATCACTCTCCACAGCAAGCTCATGGTCCTACATTGTGGAAACATCTAGTTCAGACAATGGAACGTGTTACCCAGGAGATTTCATCGATTATGAGGAGCTAAGAGAGCAATTGAGCTCAGTGTCATCATTTGAAAGGTTTGAGATATTCCCCAAGACAAGTTCATGGCCCAATCATGACTCGAACAAAGGTGTAACGGCAGCATGTCCTCATGCTGGAGCAAAAAGCTTCTACAAAAATTTAATATGGCTAGTTAAAAAAGGAAATTCATACCCAAAGCTCAGCAAATCCTACATTAATGATAAAGGGAAAGAAGTCCTAGTGCTATGGGGCATTCATCATCCATCTACTAGTGCTGACCAACAAAGTCTCTATCAGAATGCAGATGCATATGTTTTTGTGGGGACATCAAGATACAGCAAGAAGTTCAAGCCGGAAATAGCAATAAGACCCAAAGTGAGGGATCAAGAAGGGAGAATGAACTATTACTGGACACTAGTAGAGCCGGGAGACAAAATAACATTCGAAGCAACTGGAAATCTAGTGGTACCGAGATATGCATTCGCAATGGAAAGAAATGCTGGATCTGGTATTATCATTTCAGATACACCAGTCCACGATTGCAATACAACTTGTCAGACACCCAAGGGTGCTATAAACACCAGCCTCCCATTTCAGAATATACATCCGATCACAATTGGAAAATGTCCAAAATATGTAAAAAGCACAAAATTGAGACTGGCCACAGGATTGAGGAATGTCCCGTCTATTCAATCTAGAGGCCTATTTGGGGCCATTGCCGGTTTCATTGAAGGGGGGTGGACAGGGATGGTAGATGGATGGTACGGTTATCACCATCAAAATGAGCAGGGGTCAGGATATGCAGCCGACCTGAAGAGCACACAGAATGCCATTGACGAGATTACTAACAAAGTAAATTCTGTTATTGAAAAGATGAATACACAGTTCACAGCAGTAGGTAAAGAGTTCAACCACCTGGAAAAAAGAATAGAGAATTTAAATAAAAAGGTTGATGATGGTTTCCTGGACATTTGGACTTACAATGCAGAACTGTTGGTTCTATTGGAAAATGAAAGAACTTTGGACTACCACGATTCAAATGTGAAGAACTTATATGAAAAGGTAAGAAGCCAGTTAAAAAACAATGCCAAGGAAATTGGAAACGGCTGCTTTGAATTTTACCACAAATGCGATAACACGTGCATGGAAAGTGTCAAAAATGGGACTTATGACTACCCAAAATACTCAGAGGAAGCAAAATTAAACAGAGAAGAAATAGATGGGGTAAAGCTGGAATCAACAAGGATTTACCAGATTTTGGCGATCTATTCAACTGTCGCCAGTTCATTGGTACTGGTAGTCTCCCTGGGGGCAATCAGTTTCTGGATGTGCTCTAATGGGTCTCTACAGTGTAGAATATGTATTTAA

>LC638153.1Influenza A virus H1N101701

ATGAAGGCAATACTAGTAGTTCTGCTATATACATTTGCAACCGCAAATGCAGACACATTATGTATAGGTTATCATGCGAACAATTCAACAGACACTGTAGACACAGTACTAGAAAAGAATGTAACAGTAACACACTCTGTTAACCTTCTAGAAGACAAGCATAACGGGAAACTATGCAAACTAAGAGGGGTAGCCCCATTGCATTTGGGTAAATGTAACATTGCTGGCTGGATCCTGGGAAATCCAGAGTGTGAATCACTCTCCACAGCAAGCTCATGGTCCTACATTGTGGAAACATCTAGTTCAGACAATGGAACGTGTTACCCAGGAGATTTCATCGATTATGAGGAGCTAAGAGAGCAATTGAGCTCAGTGTCATCATTTGAAAGGTTTGAGATATTCCCCAAGACAAGTTCATGGCCCAATCATGACTCGAACAAAGGTGTAACGGCAGCATGTCCTCATGCTGGGGCAAAAAGCTTCTACAAAAATTTAATATGGCTAGTTAAAAAAGGAAATTCATACCCAAAGCTCAGCAAATCCTACATTAATGATAAAGGGAAAGAAGTCCTCGTGCTATGGGGCATTCACCATCCATCTACTAGTGCTGACCAACAAAGTCTCTATCAGAATGCAGATGCATATGTTTTTGTGGGGACATCAAGATACAGCAAGAAGTTCAAGCCGGAAATAGCAATAAGACCCAAAGTGAGGGATCAAGAAGGGAGAATGAACTATTACTGGACACTAGTAGAGCCGGGAGACAAAATAACATTCGAAGCAACTGGAAATCTAGTGGTACCGAGATATGCATTCGCAATGGAAAGAAATGCTGGATCTGGTATTATCATTTCAGATACACCAGTCCACGATTGCAATACAACTTGTCAGACACCCAAGGGTGCTATGAACACCAGCCTCCCATTTCAGAATATACATCCGATCACAATTGGAAAATGTCCAAAATATGTAAAAAGCACAAAATTGAGACTGGCCACAGGATTGAGGAATGTCCCGTCTATTCAATCTAGAGGCCTATTTGGGGCCATTGCCGGTTTCATTGAAGGGGGGTGGACAGGGATGGTAGATGGATGGTACGGTTATCACCATCAAAATGAGCAGGGGTCAGGATATGCAGCCGACTTGAAGAGCACACAGAATGCCATTGACGAGATTACTAACAAAGTAAATTCTGTTATTGAAAAGATGAATACACAGTTCACAGCAGTAGGTAAAGAGTTCAACCACCTGGAAAAAAGAATAGAGAATTTAAATAAAAAGGTTGATGATGGTTTCCTGGACATTTGGACTTACAATGCAGAACTGTTGGTTCTATTGGAAAATGAAAGAACTTTGGACTACCACGATTCAAATGTGAAGAACTTATATGAAAAGGTAAGAAGCCAGTTAAAAAACAATGCCAAGGAAATTGGAAACGGCTGCTTTGAATTTTACCACAAATGCGATAACACGTGCATGGAAAGTGTCAAAAATGGGACTTATGACTACCCAAAATACTCAGAGGAAGCAAAATTAAACAGAGAAGAAATAGATGGGGTAAAGCTGGAATCAACAAGGATTTACCAGATTTTGGCGATCTATTCAACTGTCGCCAGTTCATTGGTACTGGTAGTCTCCCTGGGGGCAATCAGTTTCTGGATGTGCTCTAATGGGTCTCTACAGTGTAGAATATGTATTTAA

>LC638149.1Influenza A virus H1N101701

ATGAAGGCAATACTAGTAGTTCTGCTATATACATTTGCAACCGCAAATGCAGACACATTATGTATAGGTTATCATGCGAACAATTCAACAGACACTGTAGACACAATACTAGAAAAGAATGTAACAGTAACACACTCTGTTAACCTTCTAGAAGACAAGCATAACGGGAAACTATGCAAACTAAGAGGGGTAGCCCCATTGCATTTGGGTAAATGTAACATTGCTGGCTGGATCCTGGGAAATCCAGAGTGTGAATCACTCTCCACAGCAAGCTCATGGTCCTACATTGTGGAAACATCTAGTTCAGACAATGGAACGTGTTACCCAGGAGATTTCATCGATTATGAGGAGCTAAGAGAGCAATTGAGCTCAGTGTCATCATTTGAAAGGTTTGAGATATTCCCCAAGACAAGTTCATGGCCCAATCATGACTCGAACAAAGGTGTAACGGCAGCATGTCCTCATGCTGGAGCAAAAAGCTTCTACAAAAATTTAATATGGCTAGTTAAAAAAGGAAATTCATACCCAAAGCTCAGCAAATCCTACATTAATGATAAAGGGAAAGAAGTCCTCGTGCTATGGGGCATTCACCATCCATCTACTAGTGCTGACCAACAAAGTCTCTATCAGAATGCAGATGCATATGTTTTTGTGGGGACATCAAGATACAGCAAGAAGTTCAAGCCGGAAATAGCAATAAGACCCAAAGTGAGGGATCAAGAAGGGAGAATGAACTATTACTGGACACTAGTAGAGCCGGGAGACAAAATAACATTCGAAGCAACTGGAAATCTAGTGGTACCGAGATATGCATTCGCAATGGAAAGAAATGCTGGATCTGGTATTATCATTTCAGATACACCAGTCCACGATTGCAATACAACTTGTCAGACACCCAAGGGTGCTATAAACACCAGCCTCCCATTTCAGAATATACATCCGATCACAATTGGAAAATGTCCAAAATATGTAAAAAGCACAAAATTGAGACTGGCCACAGGATTGAGGAATGTCCCGTCTATTCAATCTAGAGGCCTATTTGGGGCCATTGCCGGTTTCATTGAAGGGGGGTGGACAGGGATGGTAGATGGATGGTACGGTTATCACCATCAAAATGAGCAGGGGTCAGGATATGCAGCCGACCTGAAGAGCACACAGAATGCCATTGACGAGATTACTAACAAAGTAAATTCTGTTATTGAAAAGATGAATACACAGTTCACAGCAGTAGGTAAAGAGTTCAACCACCTGGAAAAAAGAATAGAGAATTTAAATAAAAAGGTTGATGATGGTTTCCTGGACATTTGGACTTACAATGCCGAACTGTTGGTTCTATTGGAAAATGAAAGAACTTTGGACTACCACGATTCAAATGTGAAGAACTTATATGAAAAGGTAAGAAGCCAGTTAAAAAACAATGCCAAGGAAATTGGAAACGGCTGCTTTGAATTTTACCACAAATGCGATAACACGTGCATGGAAAGTGTCAAAAATGGGACTTATGACTACCCAAAATACTCAGAGGAAGCAAAATTAAACAGAGAAGAAATAGATGGGGTAAAGCTGGAATCAACAAGGATTTACCAGATTTTGGCGATCTATTCAACTGTCGCCAGTTCATTGGTACTGGTAGTCTCCCTGGGGGCAATCAGTTTCTGGATGTGCTCTAATGGGTCTCTACAGTGTAGAATATGTATTTAA

>LC638148.1Influenza A virus H1N101701

ATGAAGGCAATACTAGTAGTTCTGCTATATACATTTGCAACCGCAAATGCAGACACATTATGTATAGGTTATCATGCGAACAATTCAACTGACACTGTAGACACAGTACTAGAAAAGAATGTAACAGTAACACACTCTGTTAACCTTCTAGAAGACAAGCATAACGGGAAACTATGCAAACTAAGAGGGGTAGCCCCATTGCATTTGGGTAAATGTAACATTGCTGGCTGGATCCTGGGAAATCCAGAGTGTGAATCACTCTCCACAGCAAGCTCATGGTCCTACATTGTGGAAACATCTAGTTCAGACAATGGAACGTGTTACCCAGGAGATTTCATCGATTATGAGGAGCTAAGAGAGCAATTGAGCTCAGTGTCATCATTTGAAAGGTTTGAGATATTCCCCAAGACAAGTTCATGGCCCAATCATGACTCGAACAAAGGTGTGACGGCAGCATGTCCTCATGCTGGAGCAAAAAGCTTCTACAAAAATTTAATATGGCTAGTTAAAAAAGGAAATTCATACCCAAAGCTCAGCAAATCCTACATTAATGATAAAGGGAGAGAAGTCCTCGTGCTATGGGGCATTCACCATCCATCTACTAGTGCTGACCAACAAAGTCTCTATCAGAATGCAGATACATATGTTTTTGTGGGGACATCAAGATACAGCAAGAAGTTCAAGCCGGAAATAGCAATAAGACCCAAAGTGAGGGATCAAGAAGGGAGAATGAACTATTACTGGACACTAGTAGAGCCGGGAGACAAAATAACATTCGAAGCAACTGGAAATCTAGTGGTACCGAGATATGCATTCGCAATGGAAAGAAATGCTGGATCTGGTATTATCATTTCAGATACACCAGTCCACGATTGCAATACAACTTGTCAGACACCCAAGGGTGCTATAAACACCAGCCTCCCATTTCAGAATATACATCCGATCACAATTGGAAAATGTCCAAAATATGTAAAAAGCACAAAATTGAGACTGGCCACAGGATTGAGGAATGTCCCGTCTATTCAATCTAGAGGCCTATTTGGGGCCATTGCCGGTTTCATTGAAGGGGGGTGGACAGGGATGGTAGATGGATGGTACGGTTATCACCATCAAAATGAGCAGGGGTCAGGATATGCAGCCGACCTGAAGAGCACACAGAATGCCATTGACGAGATTACTAACAAAGTAAATTCTGTTATTGAAAAGATGAATACACAGTTCACAGCAGTAGGTAAAGAGTTCAACCACCTGGAAAAAAGAATAGAGAATTTAAATAAAAAAGTTGATGATGGTTTCCTGGACATTTGGACTTACAATGCCGAACTGTTGGTTCTATTGGAAAATGAAAGAACTTTGGACTACCACGATTCAAATGTGAAGAACTTATATGAAAAGGTAAGAAGCCAGTTAAAAAACAATGCCAAGGAAATTGGAAACGGCTGCTTTGAATTTTACCACAAATGCGATAACACGTGCATGGAAAGTGTCAAAAATGGGACTTATGACTACCCAAAATACTCAGAGGAAGCAAAATTAAACAGAGAAGAAATAGATGGGGTAAAGCTGGAATCAACAAGGATTTACCAGATTTTGGCGATCTATTCAACTGTCGCCAGTTCATTGGTACTGGTAGTCTCCCTGGGGGCAATCAGTTTCTGGATGTGCTCTAATGGGTCTCTACAGTGTAGAATATGTATTTAA

>LC638147.1Influenza A virus H1N101701

ATGAAGGCAATACTAGTAGTTCTGCTATATACATTTGCAACCGCAAATGCAGACACATTATGTATAGGTTATCATGCGAACAATTCAACAGACACTGTAGACACAATACTAGAAAAGAATGTAACAGTAACACACTCTGTTAACCTTCTAGAAGACAAGCATAACGGGAAACTATGCAAACTAAGAGGGGTAGCCCCATTGCATTTGGGTAAATGTAACATTGCTGGCTGGATCCTGGGAAATCCAGAGTGTGAATCACTCTCCACAGCAAGCTCATGGTCCTACATTGTGGAAACATCTAGTTCAGACAATGGAACGTGTTACCCAGGAGATTTCATCGATTATGAGGAGCTAAGAGAGCAATTGAGCTCAGTGTCATCATTTGAAAGGTTTGAGATATTCCCCAAGACAAGTTCATGGCCCAATCATGACTCGAACAAAGGTGTAACGGCAGCATGTCCTCATGCTGGAGCAAAAAGCTTCTACAAAAATTTAATATGGCTAGTTAAAAAAGGAAATTCATACCCAAAGCTCAGCAAATCCTACATTAATGATAAAGGGAAAGAAGTCCTCGTGCTATGGGGCATTCACCATCCATCTACTAGTGCTGACCAACAAAGTCTCTATCAGAATGCAGATGCATATGTTTTTGTGGGGACATCAAGATACAGCAAGAAGTTCAAGCCGGAAATAGCAATAAGACCCAAAGTGAGGGATCAAGAAGGGAGAATGAACTATTACTGGACACTAGTAGAGCCGGGAGACAAAATAACATTCGAAGCAACTGGAAATCTAGTGGTACCGAGATATGCATTCGCAATGGAAAGAAATGCTGGATCTGGTATTATCATTTCAGATACACCAGTCCACGATTGCAATACAACTTGTCAGACACCCAAGGGTGCTATAAACACCAGCCTCCCATTTCAGAATATACATCCGATCACAATTGGAAAATGTCCAAAATATGTAAAAAGCACAAAATTGAGACTGGCCACAGGATTGAGGAATGTCCCGTCTATTCAATCTAGAGGCCTATTTGGGGCCATTGCCGGTTTCATTGAAGGGGGGTGGACAGGGATGGTAGATGGATGGTACGGTTATCACCATCAAAATGAGCAGGGGTCAGGATATGCAGCCGACCTGAAGAGCACACAGAATGCCATTGACGAGATTACTAACAAGGTAAATTCTGTTATTGAAAAGATGAATACACAGTTCACAGCAGTAGGTAAAGAGTTCAACCACCTGGAAAAAAGAATAGAGAATTTAAATAAAAAGGTTGATGATGGTTTCCTGGACATTTGGACTTACAATGCCGAACTGTTGGTTCTATTGGAAAATGAAAGAACTTTGGACTACCACGATTCAAATGTGAAGAACTTATATGAAAAGGTAAGAAGCCAGTTAAAAAACAATGCCAAGGAAATTGGAAACGGCTGCTTTGAATTTTACCACAAATGCGATAACACGTGCATGGAAAGTGTCAAAAATGGGACTTATGACTACCCAAAATACTCAGAGGAAGCAAAATTAAACAGAGAAGAAATAGATGGGGTAAAGCTGGAATCAACAAGGATTTACCAGATTTTGGCGATCTATTCAACTGTCGCCAGTTCATTGGTACTGGTAGTCTCCCTGGGGGCAATCAGTTTCTGGATGTGCTCTAATGGGTCTCTACAGTGTAGAATATGTATTTAA

>LC638146.1Influenza A virus H1N101701

ATGAAGGCAATACTAGTAGTTCTGCTATATACATTTGCAACCGCAAATGCAGACACATTATGTATAGGTTATCATGCGAACAATTCAACAGACACTGTAGACACAATACTAGAAAAGAATGTAACAGTAACACACTCTGTTAACCTTCTAGAAGACAAGCATAACGGGAAACTATGCAAACTAAGAGGGGTAGCCCCATTGCATTTGGGTAAATGTAACATTGCTGGCTGGATCCTGGGAAATCCAGAGTGTGAATCACTCTCCACAGCAAGCTCATGGTCCTACATTGTGGAAACATCTAGTTCAGACAATGGAACGTGTTACCCAGGAGATTTCATCGATTATGAGGAGCTAAGAGAGCAATTGAGCTCAGTGTCATCATTTGAAAGGTTTGAGATATTCCCCAAGACAAGTTCATGGCCCAATCATGACTCGAACAAAGGTGTAACGGCAGCATGTCCTCATGCTGGAGCAAAAAGCTTCTACAAAAATTTAATATGGCTAGTTAAAAAAGGAAATTCATACCCAAAGCTCAGCAAATCCTACATTAATGATAAAGGGAAAGAAGTCCTCGTGCTATGGGGCATTCACCATCCATCTACTAGTGCTGACCAACAAAGTCTCTATCAGAATGCAGATGCATATGTTTTTGTGGGGACATCAAGATACAGCAAGAAGTTCAAGCCGGAAATAGCAATAAGACCCAAAGTGAGGGATCAAGAAGGGAGAATGAACTATTACTGGACACTAGTAGAGCCGGGAGACAAAATAACATTCGAAGCAACTGGAAATCTAGTGGTACCGAGATATGCATTCGCAATGGAAAGAAATGCTGGATCTGGTATTATCATTTCAGATACACCAGTCCACGATTGCAATACAACTTGTCAGACACCCAAGGGTGCTATAAACACCAGCCTCCCATTTCAGAATATACATCCGATCACAATTGGAAAATGTCCAAAATATGTAAAAAGCACAAAATTGAGACTGGCCACAGGATTGAGGAATGTCCCGTCTATTCAATCTAGAGGCCTATTTGGGGCCATTGCCGGTTTCATTGAAGGGGGGTGGACAGGGATGGTAGATGGATGGTACGGTTATCACCATCAAAATGAGCAGGGGTCAGGATATGCAGCCGACCTGAAGAGCACACAGAATGCCATTGACGAGATTACTAACAAAGTAAATTCTGTTATTGAAAAGATGAATACACAGTTCACAGCAGTAGGTAAAGAGTTCAACCACCTGGAAAAAAGAATAGAGAATTTAAATAAAAAGGTTGATGATGGTTTCCTGGACATTTGGACTTACAATGCCGAACTGTTGGTTCTATTGGAAAATGAAAGAACTTTGGACTACCACGATTCAAATGTGAAGAACTTATATGAAAAGGTAAGAAGCCAGTTAAAAAACAATGCCAAGGAAATTGGAAACGGCTGCTTTGAATTTTACCACAAATGCGATAACACGTGCATGGAAAGTGTCAAAAATGGGACTTATGACTACCCAAAATACTCAGAGGAAGCAAAATTAAACAGAGAAGAAATAGATGGGGTAAAGCTGGAATCAACAAGGATTTACCAGATTTTGGCGATCTATTCAACTGTCGCCAGTTCATTGGTACTGGTAGTCTCCCTGGGGGCAATCAGTTTCTGGATGTGCTCTAATGGGTCTCTACAGTGTAGAATATGTATTTAA

>LC638145.1Influenza A virus H1N101701

ATGAAGGCAATACTAGTAGTTCTGCTATATACATTTGCAACCGCAAATGCAGACACATTATGTATAGGTTATCATGCGAACAATTCAACAGACACTGTAGACACAATACTAGAAAAGAATGTAACAGTAACACACTCTGTTAACCTTCTAGAAGACAAGCATAACGGGAAACTATGCAAACTAAGAGGGGTAGCCCCATTGCATTTGGGTAAATGTAACATTGCTGGCTGGATCCTGGGAAATCCAGAGTGTGAATCACTCTCCACAGCAAGCTCATGGTCCTACATTGTGGAAACATCTAGTTCAGACAATGGAACGTGTTACCCAGGAGATTTCATCGATTATGAGGAGCTAAGAGAGCAATTGAGCTCAGTGTCATCATTTGAAAGGTTTGAGATATTCCCCAAGACAAGTTCATGGCCCAATCATGACTCGAACAAAGGTGTAACGGCAGCATGTCCTCATGCTGGAGCAAAAAGCTTCTACAAAAATTTAATATGGCTAGTTAAAAAAGGAAATTCATACCCAAAGCTCAGCAAATCCTACATTAATGATAAAGGGAAAGAAGTCCTCGTGCTATGGGGCATTCACCATCCATCTACTAGTGCTGACCAACAAAGTCTCTATCAGAATGCAGATGCATATGTTTTTGTGGGGACATCAAGATACAGCAAGAAGTTCAAGCCGGAAATAGCAATAAGACCCAAAGTGAGGGATCAAGAAGGGAGAATGAACTATTACTGGACACTAGTAGAGCCGGGAGACAAAATAACATTCGAAGCAACTGGAAATCTAGTGGTACCGAGATATGCATTCGCAATGGAAAGAAATGCTGGATCTGGTATTATCATTTCAGATACACCAGTCCACGATTGCAATACAACTTGTCAGACACCCAAGGGTGCTATAAACACCAGCCTCCCATTTCAGAATATACATCCGATCACAATTGGAAAATGTCCAAAATATGTAAAAAGCACAAAATTGAGACTGGCCACAGGATTGAGGAATGTCCCGTCTATTCAATCTAGAGGCCTATTTGGGGCCATTGCCGGTTTCATTGAAGGGGGGTGGACAGGGATGGTAGATGGATGGTACGGTTATCACCATCAAAATGAGCAGGGGTCAGGATATGCAGCCGACCTGAAGAGCACACAGAATGCCATTGACGAGATTACTAACAAAGTAAATTCTGTTATTGAAAAGATGAATACACAGTTCACAGCAGTAGGTAAAGAGTTCAACCACCTGGAAAAAAGAATAGAGAATTTAAATAAAAAGGTTGATGATGGTTTCCTGGACATTTGGACTTACAATGCCGAACTGTTGGTTCTATTGGAAAATGAAAGAACTTTGGACTACCACGATTCAAATGTGAAGAACTTATATGAAAAGGTAAGAAGCCAGTTAAAAAACAATGCCAAGGAAATTGGAAACGGCTGCTTTGAATTTTACCACAAATGCGATAACACGTGCATGGAAAGTGTCAAAAATGGGACTTATGACTACCCAAAATACTCAGAGGAAGCAAAATTAAACAGAGAAGAAATAGATGGGGTAAAGCTGGAATCAACAAGGATTTACCAGATTTTGGCGATCTATTCAACTGTCGCCAGTTCATTGGTACTGGTAGTCTCCCTGGGGGCAATCAGTTTCTGGATGTGCTCTAATGGGTCTCTACAGTGTAGAATATGTATTTAA

>LC638144.1Influenza A virus H1N101701

ATGAAGGCAATACTAGTAGTTCTGCTATATACATTTGCAACCGCAAATGCAGACACATTATGTATAGGTTATCATGCGAACAATTCAACAGACACTGTAGACACAGTACTAGAAAAGAATGTAACAGTAACACACTCTGTTAACCTCCTAGAAGACAAGCATAACGGGAAACTATGCAAACTAAGAGGGGTAGCCCCATTGCATTTGGGTAAATGTAACATTGCTGGCTGGATCCTGGGAAATCCAGAGTGTGAATCACTCTCCACAGCAAGCTCATGGTCCTACATTGTGGAAACATCTAGTTCAGACAATGGAACGTGTTACCCAGGAGATTTCATCGATTATGAGGAGCTAAGAGAGCAATTGAGCTCAGTGTCATCATTTGAAAGGTTTGAGATATTCCCCAAGACAAGTTCATGGCCCAATCATGACTCGAACAAAGGTGTAACGGCAGCATGTCCTCATGCTGGAGCAAAAAGCTTCTACAAAAATTTAATATGGCTAGTTAAAAAAGGAAATTCATACCCAAAGCTCAGCAAATCCTACATTAATGATAAAGGGAAAGAAGTCCTCGTGCTATGGGGCATTCACCATCCATCTACTAGTGCTGACCAACAAAGTCTCTATCAGAATGCAGATACATATGTTTTTGTGGGGACATCAAGATACAGCAAGAAGTTCAAGCCGGAAATAGCAATAAGACCCAAAGTGAGGGATCAAGAAGGGAGAATGAACTATTACTGGACACTAGTAGAGCCGGGAGACAAAATAACATTCGAAGCAACTGGAAATCTAGTGGTACCGAGATATGCATTCGCAATGGAAAGAAATGCTGGATCTGGTATTATCATTTCAGATACACCAGTCCACGATTGCAATACAACTTGTCAGACACCCAAGGGTGCTATAAACACCAGCCTCCCATTTCAGAATATACATCCGATCACAATTGGAAAATGTCCAAAATATGTAAAAAGCACAAAATTGAGACTGGCCACAGGATTGAGGAATATCCCGTCTATTCAATCTAGAGGCCTATTTGGGGCTATTGCCGGTTTCATTGAAGGGGGGTGGACAGGGATGGTAGATGGATGGTACGGTTATCACCATCAAAATGAGCAGGGGTCAGGATATGCAGCCGACCTGAAGAGCACACAGAATGCCATTGACGAGATTACTAACAAAGTAAATTCTGTTATTGAAAAGATGAATACACAGTTCACAGCAGTAGGTAAAGAGTTCAACCACCTGGAAAAAAGAATAGAGAATTTAAATAAAAAAGTTGATGATGGTTTCCTGGACATTTGGACTTACAATGCCGAACTGTTGGTTCTATTGGAAAATGAAAGAACTTTGGACTACCACGATTCAAATGTGAAGAACTTATATGAAAAGGTAAGAAGCCAGTTAAAAAACAATGCCAAGGAAATTGGAAACGGCTGCTTTGAATTTTACCACAAATGCGATAACACGTGCATGGAAAGTGTCAAAAATGGGACTTATGACTACCCAAAATACTCAGAGGAAGCAAAATTAAACAGAGAAGAAATAGATGGGGTAAAGCTGGAATCAACAAGGATTTACCAGATTTTGGCGATCTATTCAACTGTCGCCAGTTCATTGGTACTGGTAGTCTCCCTGGGGGCAATCAGTTTCTGGATGTGCTCTAATGGGTCTCTACAGTGTAGAATATGTATTTAA

>LC638143.1Influenza A virus H1N101701

ATGAAGGCAATACTAGTAGTTCTGCTATATACATTTGCAACCGCAAATGCAGACACATTATGTATAGGTTATCATGCGAACAATTCAACAGACACTGTAGACACAATACTAGAAAAGAATGTAACAGTAACACACTCTGTTAACCTTCTAGAAGACAAGCATAACGGGAAACTATGCAAACTAAGAGGGGTAGCCCCATTGCATTTGGGTAAATGTAACATTGCTGGCTGGATCCTGGGAAATCCAGAGTGTGAATCACTCTCCACAGCAAGCTCATGGTCCTACATTGTGGAAACATCTAGTTCAGACAATGGAACGTGTTACCCAGGAGATTTCATCGATTATGAGGAGCTAAGAGAGCAATTGAGCTCAGTGTCATCATTTGAAAGGTTTGAGATATTCCCCAAGACAAGTTCATGGCCCAATCATGACTCGAACAAAGGTGTAACGGCAGCATGTCCTCATGCTGGAGCAAAAAGCTTCTACAAAAATTTAATATGGCTAGTTAAAAAAGGAAATTCATACCCAAAGCTCAGCAAATCCTACATTAATGATAAAGGGAAAGAAGTCCTCGTGCTATGGGGCATTCACCATCCATCTACTAGTGCTGACCAACAAAGTCTCTATCAGAATGCAGATGCATATGTTTTTGTGGGGACATCAAGATACAGCAAGAAGTTCAAGCCGGAAATAGCAATAAGACCCAAAGTGAGGGATCAAGAAGGGAGAATGAACTATTACTGGACACTAGTAGAGCCGGGAGACAAAATAACATTCGAAGCAACTGGAAATCTAGTGGTACCGAGATATGCATTCGCAATGGAAAGAAATGCTGGATCTGGTATTATCATTTCAGATACACCAGTCCACGATTGCAATACAACTTGTCAGACACCCAAGGGTGCTATAAACACCAGCCTCCCATTTCAGAATATACATCCGATCACAATTGGAAAATGTCCAAAATATGTAAAAAGCACAAAATTGAGACTGGCCACAGGATTGAGGAATGTCCCGTCTATTCAATCTAGAGGCCTATTTGGGGCCATTGCCGGTTTCATTGAAGGGGGGTGGACAGGGATGGTAGATGGATGGTACGGTTATCACCATCAAAATGAGCAGGGGTCAGGATATGCAGCCGACCTGAAGAGCACACAGAATGCCATTGACGAGATTACTAACAAAGTAAATTCTGTTATTGAAAAGATGAATACACAGTTCACAGCAGTAGGTAAAGAGTTCAACCACCTGGGAAAAAGAATAGAGAATTTAAATAAAAAGGTTGATGATGGTTTCCTGGACATTTGGACTTACAATGCCGAACTGTTGGTTCTATTGGAAAATGAAAGAACTTTGGACTACCACGATTCAAATGTGAAGAACTTATATGAAAAGGTAAGAAGCCAGTTAAAAAACAATGCCAAGGAAATTGGAAACGGCTGCTTTGAATTTTACCACAAATGCGATAACACGTGCATGGAAAGTGTCAAAAATGGGACTTATGACTACCCAAAATACTCAGAGGAAGCAAAATTAAACAGAGAAGAAATAGATGGGGTAAAGCTGGAATCAACAAGGATTTACCAGATTTTGGCGATCTATTCAACTGTCGCCAGTTCATTGGTACTGGTAGTCTCCCTGGGGGCAATCAGTTTCTGGATGTGCTCTAATGGGTCTCTACAGTGTAGAATATGTATTTAA

>LC638142.1Influenza A virus H1N101701

ATGAAGGCAATACTAGTAGTTCTGCTATATACATTTGCAACCGCAAATGCAGACACATTATGTATAGGTTATCATGCGAACAATTCAACAGACACTGTAGACACAGTACTAGAAAAGAATGTAACAGTAACACATTCTGTTAACCTTCTAGAAGACAAGCATAACGGGAAACTATGCAAACTAAGAGGGGTAGCCCCATTGCATTTGGGTAAATGTAACATTGCTGGCTGGATCCTGGGAAATCCAGAGTGTGAATCACTCTCCACAGCAAGCTCATGGTCCTACATTGTGGAAACATCTAGTTCAGACAATGGAACGTGTTACCCAGGAGATTTCATCGATTATGAGGAGCTAAGAGAACAATTGAGCTCAGTGTCATCATTTGAAAGGTTTGAGATATTCCCCAAGACAAGTTCATGGCCCAATCATGACTCGAACAAAGGTGTAACGGCAGCATGTCCTCATGCTGGAGCAAAAAGCTTCTACAAAAATTTAATATGGCTAGTTAAAAAAGGAAATTCATACCCAAAGCTCAGCAAATCCTACATTAATGATAAAGGGAAAGAAGTCCTCGTGCTATGGGGCATTCATCATCCATCTACTAGTGCTGACCAACAAAGTCTCTATCAGAATGCAGATGCATATGTTTTTGTGGGGACATCAAGATACAGCAAGAAGTTCAAGCCGGAAATAGCAATAAGACCCAAAGTGAGGGATCAAGAAGGGAGAATGAACTATTACTGGACACTAGTAGAGCCGGGAGACAAAATAACATTCGAAGCAACTGGAAATCTAGTGGTACCGAGATATGCATTCGCAATGGAAAGAAATGCTGGATCTGGTATTATCATTTCAGATACACCAGTCCACGATTGCAATACAACTTGTCAGACACCCAAGGGTGCTATAAACACCAGCCTCCCATTTCAGAATATACATCCGATCACAATTGGAAAATGTCCAAAATATGTAAAAAGCACAAAATTGAGACTGGCCACAGGATTGAGGAATGTCCCGTCTATTCAATCTAGAGGCCTATTTGGGGCCATTGCCGGTTTCATTGAAGGGGGGTGGACAGGGATGGTAGATGGATGGTACGGTTATCACCATCAAAATGAGCAGGGGTCAGGATATGCAGCCGACCTGAAGAGCACACAGAATGCCATTGACGAGATTACTAACAAAGTAAATTCTGTTATTGAAAAGATGAATACACAGTTCACAGCAGTAGGTAAAGAGTTCAACCACCTGGAAAAAAGAATAGAGAATTTAAATAAAAAGGTTGATGATGGTTTCCTGGACATTTGGACTTACAATGCAGAACTGTTGGTTCTATTGGAAAATGAAAGAACTTTGGACTACCACGATTCAAATGTGAAGAACTTATATGAAAAGGTAAGAAGCCAGTTAAAAAACAATGCCAAGGAAATTGGAAACGGCTGCTTTGAATTTTACCACAAATGCGATAACACGTGCATGGAAAGTGTCAAAAATGGGACTTATGACTACCCAAAATACTCAGAGGAAGCAAAATTAAACAGAGAAGAAATAGATGGGGTAAAGCTGGAATCAACAAGGATTTACCAGATTTTGGCGATCTATTCAACTGTCGCCAGTTCATTGGTACTGGTAGTCTCCCTGGGGGCAATCAGTTTCTGGATGTGCTCTAATGGGTCTCTACAGTGTAGAATATGTATTTAA

>LC638141.1Influenza A virus H1N101701

ATGAAGGCAATACTAGTAGTTCTGCTATATACATTTGCAACCGCAAATGCAGACACATTATGTATAGGTTATCATGCGAACAATTCAACAGACACTGTAGACACAATACTAGAAAAGAATGTAACAGTAACACACTCTGTTAACCTTCTAGAAGACAAGCATAACGGGAAACTATGCAAACTAAGAGGGGTAGCCCCATTGCATTTGGGTAAATGTAACATTGCTGGCTGGATCCTGGGAAATCCAGAGTGTGAATCACTCTCCACAGCAAGCTCATGGTCCTACATTGTGGAAACATCTAGTTCAGACAATGGAACGTGTTACCCAGGAGATTTCATCGATTATGAGGAGCTAAGAGAGCAATTGAGCTCAGTGTCATCATTTGAAAGGTTTGAGATATTCCCCAAGACAAGTTCATGGCCCAATCATGACTCGAACAAAGGTGTAACGGCAGCATGTCCTCATGCTGGAGCAAAAAGCTTCTACAAAAATTTAATATGGCTAGTTAAAAAAGGAAATTCATACCCAAAGCTCAGCAAATCCTACATTAATGATAAAGGGAAAGAAGTCCTCGTGCTATGGGGCATTCACCATCCATCTACTAGTGCTGACCAACAAAGTCTCTATCAGAATGCAGATGCATATGTTTTTGTGGAGACATCAAGATACAGCAAGAAGTTCAAGCCGGAAATAGCAATAAGACCCAAAGTGAGGGATCAAGAAGGGAGAATGAACTATTACTGGACACTAGTAGAGCCGGGAGACAAAATAACATTCGAAGCAACTGGAAATCTAATGGTACCGAGATATGCATTCGCAATGGAAAGAAATGCTGGATCTGGTATTATCATTTCAGATACACCAGTCCACGATTGCAATACAACTTGTCAGACACCCAAGGGTGCTATAAACACCAGCCTCCCATTTCAGAATATACATCCGATCACAATTGGAAAATGTCCAAAATATGTAAAAAGCACAAAATTGAGACTGGCCACAGGATTGAGGAATGTCCCGTCTATTCAATCTAGAGGCCTATTTGGGGCCATTGCCGGTTTCATTGAAGGGGGGTGGACAGGGATGGTAGATGGATGGTACGGTTATCACCATCAAAATGAGCAGGGGTCAGGATATGCAGCCGACCTGAAGAGCACACAGAATGCCATTGACGAGATTACTAACAAAGTAAATTCTGTTATTGAAAAGATGAATACACAGTTCACAGCAGTAGGTAAAGAGTTCAACCACCTGGAAAAAAGAATAGAGAATTTAAATAAAAAGGTTGATGATGGTTTCCTGGACATTTGGACTTACAATGCCGAACTGTTGGTTCTATTGGAAAATGAAAGAACTTTGGACTACCACGATTCAAATGTGAAGAACTTATATGAAAAGGTAAGAAGCCAGTTAAAAAACAATGCCAAGGAAATTGGAAACGGCTGCTTTGAATTTTACCACAAATGCGATAACACGTGCATGGAAAGTGTCAAAAATGGGACTTATGACTACCCAAAATACTCAGAGGAAGCAAAATTAAACAGAGAAGAAATAGATGGGGTAAAGCTGGAATCAACAAGGATTTACCAGATTTTGGCGATCTATTCAACTGTCGCCAGTTCATTGGTACTGGTAGTCTCCCTGGGGGCAATCAGTTTCTGGATGTGCTCTAATGGGTCTCTACAGTGTAGAATATGTATTTAA

>LC638140.1Influenza A virus H1N101701

ATGAAGGCAATACTAGTAGTTCTGCTATATACATTTGCAACCGCAAATGCAGACACATTATGTATAGGTTATCATGCGAACAATTCAACAGACACTGTAGACACAGTACTAGAAAAGAATGTAACAGTAACACATTCTGTTAACCTTCTAGAAGACAAGCATAACGGGAAACTATGCAAACTAAGAGGGGTAGCCCCATTGCATTTGGGTAAATGTAACATTGCTGGCTGGATCCTGGGAAATCCAGAGTGTGAATCACTCTCCACAGCAAGCTCATGGTCCTACATTGTGGAAACATCTAGTTCAGACAATGGAACGTGTTACCCAGGAGATTTCATCGATTATGAGGAGCTAAGAGAGCAATTGAGCTCAGTGTCATCATTTGAAAGGTTTGAGATATTCCCCAAGACAAGTTCATGGCCCAATCATGACTCGAACAAAGGTGTAACGGCAGCATGTCCTCATGCTGGAGCAAAAAGCTTCTACAAAAATTTAATATGGCTAGTTAAAAAAGGAAATTCATACCCAAAGCTCAGCAAATCCTACATTAATGATAAAGGGAAAGAAGTCCTCGTGCTATGGGGCATTCATCATCCATCTACTAGTGCTGACCAACAAAGTCTCTATCAGAATGCAGATGCATATGTTTTTGTGGGGACATCAAGATACAGCAAGAAGTTCAAGCCGGAAATAGCAATAAGACCAAAAGTGAGGGATCAAGAAGGGAGAATGAACTATTACTGGACACTAGTAGAGCCGGGAGACAAAATAACATTCGAAGCAACTGGAAATCTAGTGGTACCGAGATATGCATTCGCAATGGAAAGAAATGCTGGATCTGGTATTATCATTTCAGATACACCAGTCCACGATTGCAATACAACTTGTCAGACACCCAAGGGTGCTATAAACACCAGCCTCCCATTTCAGAATATACATCCGATCACAATTGGAAAATGTCCAAAATATGTAAAAAGCACAAAATTGAGACTGGCCACAGGATTGAGGAATGTCCCGTCTATTCAATCTAGAGGCCTATTTGGGGCCATTGCCGGTTTCATTGAAGGGGGGTGGACAGGGATGGTAGATGGATGGTACGGTTATCACCATCAAAATGAGCAGGGGTCAGGATATGCAGCCGACCTGAAGAGCACACAGAATGCCATTGACGAGATTACTAACAAAGTAAATTCTGTTATTGAAAAGATGAATACACAGTTCACAGCAGTAGGTAAAGAGTTCAACCACCTGGAAAAAAGAATAGAGAATTTAAATAAAAAGGTTGATGATGGTTTCCTGGACATTTGGACTTACAATGCAGAACTGTTGGTTCTATTGGAAAATGAAAGAACTTTGGACTACCACGATTCAAATGTGAAGAACTTATATGAAAAGGTAAGAAGCCAGTTAAAAAACAATGCCAAGGAAATTGGAAACGGCTGCTTTGAATTTTACCACAAATGCGATAACACGTGCATGGAAAGTGTCAAAAATGGGACTTATGACTACCCAAAATACTCAGAGGAAGCAAAATTAAACAGAGAAGAAATAGATGGGGTAAAGCTGGAATCAACAAGGATTTACCAGATTTTGGCGATCTATTCAACTGTCGCCAGTTCATTGGTACTGGTAGTCTCCCTGGGGGCAATCAGTTTCTGGATGTGCTCTAATGGGTCTCTACAGTGTAGAATATGTATTTAA

>LC638139.1Influenza A virus H1N101701

ATGAAGGCAATACTAGTAGTTCTGCTATATACATTTGCAACCGCAAATGCAGACACATTATGTATAGGTTATCATGCGAACAATTCAACAGACACTGTAGACACAGTACTAGAAAAGAATGTAACAGTAACACACTCTGTTAACCTTCTAGAAGACAAGCATAACGGGAAACTATGCAAACTAAGAGGGGTAGCCCCATTGCATTTGGGTAAATGTAACATTGCTGGCTGGATCCTGGGAAATCCAGAGTGTGAATCACTCTCCACAGCAAGCTCATGGTCCTACATTGTGGAAACATCTAGTTCAGACAATGGAACGTGTTACCCAGGAGATTTCATCGATTATGAGGAGCTAAGAGAGCAATTGAGCTCAGTGTCATCATTTGAAAGGTTTGAGATATTCCCCAAGACAAGTTCATGGCCCAATCATGACTCGAACAAAGGTGAAACGGCAGCATGTCCTCATGCTGGAGCAAAAAGCTTCTACAAAAATTTAATATGGCTAGTTAAAAAAGGAAATTCATACCCAAAGCTCAGCAAATCCTACATTAATGATAAAGGGAAAGAAGTCCTCGTGCTATGGGGCATTCACCATCCATCTACTAGTGCTGACCAACAAAGTCTCTATCAGAATGCAGATGCATATGTTTTTGTGGGGACATCAAGATACAGCAAGAAGTTCAAGCCGGAAATAGCAATAAGACCCAAAGTGAGGGATCAAGAAGGGAGAATGAACTATTACTGGACACTAGTAGAGCCGGGAGACAAAATAACATTCGAAGCAACTGGAAATCTAGTGGTACCGAGATATGCATTCGCAATGGAAAGAAATGCTGGATCTGGTATTATCATTTCAGATACACCAGTCCACGATTGCAATACAACTTGTCAGACACCCAAGGGTGCTATAAACACCAGCCTCCCATTTCAGAATATACATCCGATCACAATTGGAAAATGTCCAAAATATGTAAAAAGCACAAAATTGAGACTGGCCACAGGATTGAGGAATGTCCCGTCTATTCAATCTAGAGGCCTATTTGGGGCCATTGCCGGTTTCATTGAAGGGGGGTGGACAGGGATGGTAGATGGATGGTACGGTTATCACCATCAAAATGAGCAGGGGTCAGGATATGCAGCCGACCTGAAGAGCACACAGAATGCCATTGACAAGATTACTAACAAAGTAAATTCTGTTATTGAAAAGATGAATACACAGTTCACAGCAGTAGGTAAAGAGTTCAACCACCTGGAAAAAAGAATAGAGAATTTAAATAAAAAAGTTGATGATGGTTTCCTGGACATTTGGACTTACAATGCCGAACTGTTGGTTCTATTGGAAAATGAAAGAACTTTGGACTACCACGATTCAAATGTGAAGAACTTATATGAAAAGGTAAGAAGCCAGTTAAAAAACAATGCCAAGGAAATTGGAAACGGCTGCTTTGAATTTTACCACAAATGCGATAACACGTGCATGGAAAGTGTCAAAAATGGGACTTATGACTACCCAAAATACTCAGAGGAAGCAAAATTAAACAGAGAAGAAATAGATGGGGTAAAGCTGGAATCAACAAGGATTTACCAGATTTTGGCGATCTATTCAACTGTCGCCAGTTCATTGGTACTGGTAGTCTCCCTGGGGGCAATCAGTTTCTGGATGTGCTCTAATGGGTCTCTACAGTGTAGAATATGTATTTAA

>LC638138.1Influenza A virus H1N101701

ATGAAGGCAATACTAGTAGTTCTGCTATATACATTTGCAACCGCAAATGCAGACACATTATGTATAGGTTATCATGCGAACAATTCAACAGACACTGTAGACACAGTACTAGAAAAGAATGTAACAGTAACACACTCTGTTAACCTTCTAGAAGACAAGCATAACGGGAAACTATGCAAACTAAGAGGGGTAGCCCCATTGCATTTGGGTAAATGTAACATTGCTGGCTGGATCCTGGGAAATCCAGAGTGTGAATCACTCTCCACAGCAAGCTCATGGTCCTACATTGTGGAAACATCTAGTTCAGACAATGGAACGTGTTACCCAGGAGATTTCATCGATTATGAGGAGCTAAGAGAGCAATTGAGCTCAGTGTCATCATTTGAAAGATTTGAGATATTCCCCAAGACAAGTTCATGGCCCAATCATGACTTGAACAAAGGTGTAACGGCAGCATGTCCTCATGCTGGAGCAAAAAGCTTCTACAAAAATTTAATATGGCTAGTTAAAAAAGGAAATTCATACCCAAAGCTCAGCAAATCCTACATTAATGATAAAGGGAAAGAAGTCCTCGTGCTATGGGGCATTCACCATCCATCTACTAGTGCTGACCAACAAAGTCTCTATCAGAATGCAGATGCATATGTTTTTGTGGGGACCTCAAGATACAGCAAGAAGTTCAAGCCGGAAATAGCAATAAGACCCAAAGTGAGGGAACAAGAAGGGAGAATGAACTATTACTGGACACTAGTAGAGCCGGGAGACAAAATAACATTCGAAGCAACTGGAAATCTAGTGGTACCGAGATATGCATTCGCAATGGAAAGAAATGCTGGATCTGGTATTATCATTTCAGATACACCAGTCCACGATTGCAATACAACTTGTCAGACACCCAAGGGTGCTATAAACACCAGCCTCCCATTTCAGAATATACATTCGATCACAATTGGAAAATGTCCAAAATATGTAAAAAGCACAAAATTGAGACTGGCCACAGGATTGAGGAATGTCCCGTCTATTCAATCTAGAGGCCTATTTGGGGCCATTGCCGGTTTCATTGAAGGGGGGTGGACAGGGATGGTAGATGGATGGTACGGTTATCACCATCAAAATGAGCAGGGGTCAGGATATGCAGCCGACCTGAAGAGCACACAGAATGCCATTGACGAGATTACTAACAAAGTAAATTCTGTTATTGAAAAGATGAATACACAGTTCACAGCAGTAGGTAAAGAGTTCAACCACCTGGAAAAAAGAATAGAAAATTTAAATAAAAAAGTTGATGATGGTTTCCTGGACATTTGGACTTACAATGCCGAACTGTTGGTTCTATTGGAAAATGAAAGAACTTTGGACTACCACGATTCAAATGTGAAGAACTTATATGAAAAGGTAAGAAGCCAGTTAAAAAACAATGCCAAGGAAATTGGAAACGGCTGCTTTGAATTTTACCACAAATGCGATAACACGTGCATGGAAAGTGTCAAAAATGGGACTTATGACTACCCAAAATACTCAGAGGAAGCAAAATTAAACAGAGAAGAAATAGATGGGGTAAAGCTGGAATCAACAAGGATTTACCAGATTTTGGCGATCTATTCAACTGTCGCCAGTTCATTGGTACTGGTAGTCTCCCTGGGGGCAATCAGTTTCTGGATGTGCTCTAATGGGTCTCTACAGTGTAGAATATGTATTTAA

>LC638137.1Influenza A virus H1N101701

ATGAAGGCAATACTAGTAGTTCTGCTATATACATTTGCAACCGCAAATGCAGACACATTATGTATAGGTTATCATGCGAACAATTCAACAGACACTGTAGACACAATACTAGAAAAGAATGTAACAGTAACACACTCTGTTAACCTTCTAGAAGACAAGCATAACGGGAAACTATGCAAACTAAGAGGGGTAGCCCCATTGCATTTGGGTAAATGTAACATTGCTGGCTGGATCCTGGGAAATCCAGAGTGTGAATCACTCTCCACAGCAAGCTCATGGTCCTACATTGTGGAAACATCTAGTTCAGACAATGGAACGTGTTACCCAGGAGATTTCATCGATTATGAGGAGCTAAGAGAGCAATTGAGCTCAGTGTCATCATTTGAAAGGTTTGAGATATTCCCCAAGACAAGTTCATGGCCCAATCATGACTCGAACAAAGGTGTAACGGCAGCATGTCCTCATGCTGGAGCAAAAAGCTTCTACAAAAATTTAATATGGCTAGTTAAAAAAGGAAATTCATACCCAAAGCTCAGCAAATCCTACATTAATGATAAAGGGAAAGAAGTCCTCGTGCTATGGGGCATTCACCATCCATCTACTAGTGCTGACCAACAAAGTCTCTATCAGAATGCAGATGCATATGTTTTTGTGGGGACATCAAGATACAGCAAGAAGTTCAAGCCGGAAATAGCAATAAGACCCAAAGTGAGGGATCAAGAAGGGAGAATGAACTATTACTGGACACTAGTAGAGCCGGGAGACAAAATAACATTCGAAGCAACTGGAAATCTAGTGGTACCGAGATATGCATTCGCAATGGAAAGAAATGCTGGATCTGGTATTATCATTTCAGATACACCAGTCCACGATTGCAATACAACTTGTCAGACACCCAAGGGTGCTATAAACACCAGCCTCCCATTTCAGAATATACATCCGATCACAATTGGAAAATGTCCAAAATATGTAAAAAGCACAAAATTGAGACTGGCCACAGGATTGAGGAATGTCCCGTCTATTCAATCTAGAGGCCTATTTGGGGCCATTGCCGGTTTCATTGAAGGGGGGTGGACAGGGATGGTAGATGGATGGTACGGTTATCACCATCAAAATGAGCAGGGGTCAGGATATGCAGCCGACCTGAAGAGCACACAGAATGCCATTGACGAGATTACTAACAAGGTAAATTCTGTTATTGAAAAGATGAATACACAGTTCACAGCAGTAGGTAAAGAGTTCAACCACCTGGAAAAAAGAATAGAGAATTTAAATAAAAAGGTTGATGATGGTTTCCTGGACATTTGGACTTACAATGCCGAACTGTTGGTTCTATTGGAAAATGAAAGAACTTTGGACTACCACGATTCAAATGTGAAGAACTTATATGAAAAGGTAAGAAGCCAGTTAAAAAACAATGCCAAGGAAATTGGAAACGGCTGCTTTGAATTTTACCACAAATGCGATAACACGTGCATGGAAAGTGTCAAAAATGGGACTTATGACTACCCAAAATACTCAGAGGAAGCAAAATTAAACAGAGAAGAAATAGATGGGGTAAAGCTGGAATCAACAAGGATTTACCAGATTTTGGCGATCTATTCAACTGTCGCCAGTTCATTGGTACTGGTAGTCTCCCTGGGGGCAATCAGTTTCTGGATGTGCTCTAATGGGTCTCTACAGTGTAGAATATGTATTTAA

>LC638135.1Influenza A virus H1N101701

ATGAAGGCAATACTAGTAGTTCTGCTATATACATTTGCAACCGCAAATGCAGACACATTATGTATAGGTTATCATGCGAACAATTCAACAGACACTGTAGACACAATACTAGAAAAGAATGTAACAGTAACACACTCTGTTAACCTTCTAGAAGACAAGCATAACGGGAAACTATGCAAACTAAGAGGGGTAGCCCCATTGCATTTGGGTAAATGTAACATTGCTGGCTGGATCCTGGGAAATCCAGAGTGTGAATCACTCTCCACAGCAAGCTCATGGTCCTACATTGTGGAAACATCTAGTTCAGACAATGGAACGTGTTACCCAGGAGATTTCATCGATTATGAGGAGCTAAGAGAGCAATTGAGCTCAGTGTCATCATTTGAAAGGTTTGAGATATTCCCCAAGACAAGTTCATGGCCCAATCATGACTCGAACAAAGGTGTAACGGCAGCATGTCCTCATGCTGGAGCAAAAAGCTTCTACAAAAATTTAATATGGCTAGTTAAAAAAGGAAATTCATACCCAAAGCTCAGCAAATCCTACATTAATGATAAAGGGAAAGAAGTCCTCGTGCTATGGGGCATTCACCATCCATCTACTAGTGCTGACCAACAAAGTCTCTATCAGAATGCAGATGCATATGTTTTTGTGGGGACATCAAGATACAGCAAGAAGTTCAAGCCGGAAATAGCAATAAGACCCAAAGTGAGGGATCAAGAAGGGAGAATGAACTATTACTGGACACTAGTAGAGCCGGGAGACAAAATAACATTCGAAGCAACTGGAAATCTAGTGGTACCGAGATATGCATTCGCAATGGAAAGAAATGCTGGATCTGGTATTATCATTTCAGATACACCAGTCCACGATTGCAATACAACTTGTCAGACACCCAAGGGTGCTATAAACACCAGCCTCCCATTTCAGAATATACATCCGATCACAATTGGAAAATGTCCAAAATATGTAAAAAGCACAAAATTGAGACTGGCCACAGGATTGAGGAATGTCCCGTCTATTCAATCTAGAGGCCTATTTGGGGCCATTGCCGGTTTCATTGAAGGGGGGTGGACAGGGATGGTAGATGGATGGTACGGTTATCACCATCAAAATGAGCAGGGGTCAGGATATGCAGCCGACCTGAAGAGCACACAGAATGCCATTGACGAGATTACTAACAAGGTAAATTCTGTTATTGAAAAGATGAATACACAGTTCACAGCAGTAGGTAAAGAGTTCAACCACCTGGAAAAAAGAATAGAGAATTTAAATAAAAAGGTTGATGATGGTTTCCTGGACATTTGGACTTACAATGCCGAACTGTTGGTTCTATTGGAAAATGAAAGAACTTTGGACTACCACGATTCAAATGTGAAGAACTTATATGAAAAGGTAAGAAGCCAGTTAAAAAACAATGCCAAGGAAATTGGAAACGGCTGCTTTGAATTTTACCACAAATGCGATAACACGTGCATGGAAAGTGTCAAAAATGGGACTTATGACTACCCAAAATACTCAGAGGAAGCAAAATTAAACAGAGAAGAAATAGATGGGGTAAAGCTGGAATCAACAAGGATTTACCAGATTTTGGCGATCTATTCAACTGTCGCCAGTTCATTGGTACTGGTAGTCTCCCTGGGGGCAATCAGTTTCTGGATGTGCTCTAATGGGTCTCTACAGTGTAGAATATGTATTTAA

>LC638134.1Influenza A virus H1N101701

ATGAAGGCAATACTAGTAGTTCTGCTATATACATTTGCAACCGCAAATGCAGACACATTATGTATAGGTTATCATGCGAACAATTCAACAGACACTGTAGACACAATACTAGAAAAGAATGTAACAGTAACACACTCTGTTAACCTTCTAGAAGACAAGCATAACGGGAAACTATGCAAACTAAGAGGGGTAGCCCCATTGCATTTGGGTAAATGTAACATTGCTGGCTGGATCCTGGGAAATCCAGAGTGTGAATCACTCTCCACAGCAAGCTCATGGTCCTACATTGTGGAAACATCTAGTTCAGACAATGGAACGTGTTACCCAGGAGATTTCATCGATTATGAGGAGCTAAGAGAGCAATTGAGCTCAGTGTCATCATTTGAAAGGTTTGAGATATTCCCCAAGACAAGTTCATGGCCCAATCATGACTCGAACAAAGGTGTAACGGCAGCATGTCCTCATGCTGGAGCAAAAAGCTTCTACAAAAATTTAATATGGCTAGTTAAAAAAGGAAATTCATACCCAAAGCTCAGCAAATCCTACATTAATGATAAAGGGAAAGAAGTCCTCGTGCTATGGGGCATTCACCATCCATCTACTAGTGCTGACCAACAAAGTCTCTATCAGAATGCAGATGCATATGTTTTTGTGGGGACATCAAGATACAGCAAGAAGTTCAAGCCGGAAATAGCAATAAGACCCAAAGTGAGGGATCAAGAAGGGAGAATGAACTATTACTGGACACTAGTAGAGCCGGGAGACAAAATAACATTCGAAGCAACTGGAAATCTAGTGGTACCGAGATATGCATTCGCAATGGAAAGAAATGCTGGATCTGGTATTATCATTTCAGATACACCAGTCCACGATTGCAATACAACTTGTCAGACACCCAAGGGTGCTATAAACACCAGCCTCCCATTTCAGAATATACATCCGATCACAATTGGAAAATGTCCAAAATATGTAAAAAGCACAAAATTGAGACTGGCCACAGGATTGAGGAATGTCCCGTCTATTCAATCTAGAGGCCTATTTGGGGCCATTGCCGGTTTCATTGAAGGGGGGTGGACAGGGATGGTAGATGGATGGTACGGTTATCACCATCAAAATGAGCAGGGGTCAGGATATGCAGCCGACCTGAAGAGCACACAGAATGCCATTGACGAGATTACTAACAAGGTAAATTCTGTTATTGAAAAGATGAATACACAGTTCACAGCAGTAGGTAAAGAGTTCAACCACCTGGAAAAAAGAATAGAGAATTTAAATAAAAAGGTTGATGATGGTTTCCTGGACATTTGGACTTACAATGCCGAACTGTTGGTTCTATTGGAAAATGAAAGAACTTTGGACTACCACGATTCAAATGTGAAGAACTTATATGAAAAGGTAAGAAGCCAGTTAAAAAACAATGCCAAGGAAATTGGAAACGGCTGCTTTGAATTTTACCACAAATGCGATAACACGTGCATGGAAAGTGTCAAAAATGGGACTTATGACTACCCAAAATACTCAGAGGAAGCAAAATTAAACAGAGAAGAAATAGATGGGGTAAAGCTGGAATCAACAAGGATTTACCAGATTTTGGCGATCTATTCAACTGTCGCCAGTTCATTGGTACTGGTAGTCTCCCTGGGGGCAATCAGTTTCTGGATGTGCTCTAATGGGTCTCTACAGTGTAGAATATGTATTTAA

>LC638132.1Influenza A virus H1N101701

ATGAAGGCAATACTAGTAGTTCTGCTATATACATTTGCAACCGCAAATGCAGACACATTATGTATAGGTTATCATGCGAACAATTCAACAGACACTGTAGACACAATACTAGAAAAGAATGTAACAGTAACACACTCTGTTAACCTTCTAGAAGACAAGCATAACGGGAAACTATGCAAACTAAGAGGGGTAGCCCCATTGCATTTGGGTAAATGTAACATTGCTGGCTGGATCCTGGGAAATCCAGAGTGTGAATCACTCTCCACAGCAAGCTCATGGTCCTACATTGTGGAAACATCTAGTTCAGACAATGGAACGTGTTACCCAGGAGATTTCATCGATTATGAGGAGCTAAGAGAGCAATTGAGCTCAGTGTCATCATTTGAAAGGTTTGAGATATTCCCCAAGACAAGTTCATGGCCCAATCATGACTCGAACAAAGGTGTAACGGCAGCATGTCCTCATGCTGGAGCAAAAAGCTTCTACAAAAATTTAATATGGCTAGTTAAAAAAGGAAATTCATACCCAAAGCTCAGCAAATCCTACATTAATGATAAAGGGAAAGAAGTCCTCGTGCTATGGGGCATTCACCATCCATCTACTAGTGCTGACCAACAAAGTCTCTATCAGAATGCAGATGCATATGTTTTTGTGGGGACATCAAGATACAGCAAGAAGTTCAAGCCGGAAATAGCAATAAGACCCAAAGTGAGGGATCAAGAAGGGAGAATGAACTATTACTGGACACTAGTAGAGCCGGGAGACAAAATAACATTCGAAGCAACTGGAAATCTAGTGGTACCGAGATATGCATTCGCAATGGAAAGAAATGCTGGATCTGGTATTATCATTTCAGATACACCAGTCCACGATTGCAATACAACTTGTCAGACACCCAAGGGTGCTATAAACACCAGCCTCCCATTTCAGAATATACATCCGATCACAATTGGAAAATGTCCAAAATATGTAAAAAGCACAAAATTGAGACTGGCCACAGGATTGAGGAATGTCCCGTCTATTCAATCTAGAGGCCTATTTGGGGCCATTGCCGGTTTCATTGAAGGGGGGTGGACAGGGATGGTAGATGGATGGTACGGTTATCACCATCAAAATGAGCAGGGGTCAGGATATGCAGCCGACCTGAAGAGCACACAGAATGCCATTGACGAGATTACTAACAAGGTAAATTCTGTTATTGAAAAGATGAATACACAGTTCACAGCAGTAGGTAAAGAGTTCAACCACCTGGAAAAAAGAATAGAGAATTTAAATAAAAAGGTTGATGATGGTTTCCTGGACATTTGGACTTACAATGCCGAACTGTTGGTTCTATTGGAAAATGAAAGAACTTTGGACTACCACGATTCAAATGTGAAGAACTTATATGAAAAGGTAAGAAGCCAGTTAAAAAACAATGCCAAGGAAATTGGAAACGGCTGCTTTGAATTTTACCACAAATGCGATAACACGTGCATGGAAAGTGTCAAAAATGGGACTTATGACTACCCAAAATACTCAGAGGAAGCAAAATTAAACAGAGAAGAAATAGATGGGGTAAAGCTGGAATCAACAAGGATTTACCAGATTTTGGCGATCTATTCAACTGTCGCCAGTTCATTGGTACTGGTAGTCTCCCTGGGGGCAATCAGTTTCTGGATGTGCTCTAATGGGTCTCTACAGTGTAGAATATGTATTTAA

>LC638131.1Influenza A virus H1N101701

ATGAAGGCAATACTAGTAGTTCTGCTATATACATTTGCAACCGCAAATGCAGACACATTATGTATAGGTTATCATGCGAACAATTCAACAGACACTGTAGACACAGTACTAGAAAAGAATGTAACAGTAACACACTCTGTTAACCTTCTAGAAGACAAGCATAACGGGAAACTATGCAAACTAAGAGGGGTAGCCCCATTGCATTTGGGTAAATGTAACATTGCTGGCTGGATCCTGGGAAATCCAGAGTGTGAATCACTCTCCACAGCAAGCTCATGGTCCTACATTGTGGAAACATCTAGTTCAGACAATGGAACGTGTTACCCAGGAGATTTCATCGATTATGAGGAGCTAAGAGAGCAATTGAGCTCAGTGTCATCATTTGAAAGATTTGAGATATTCCCCAAGACAAGTTCATGGCCCAATCATGACTTGAACAAAGGTGTAACGGCAGCATGTCCTCATGCTGGAGCAAAAAGCTTCTACAAAAATTTAATATGGCTAGTTAAAAAAGGAAATTCATACCCAAAGCTCAGCAAATCCTACATTAATGATAAAGGGAAAGAAGTCCTCGTGCTATGGGGCATTCACCATCCATCTACTAGTGCTGACCAACAAAGTCTCTATCAGAATGCAGATGCATATGTTTTTGTGGGGACCTCAAGATACAGCAAGAAGTTCAAGCCGGAAATAGCAATAAGACCCAAAGTGAGGGAACAAGAAGGGAGAATGAACTATTACTGGACACTAGTAGAGCCGGGAGACAAAATAACATTCGAAGCAACTGGAAATCTAGTGGTACCGAGATATGCATTCGCAATGGAAAGAAATGCTGGATCTGGTATTATCATTTCAGATACACCAGTCCACGATTGCAATACAACTTGTCAGACACCCAAGGGTGCTATAAACACCAGCCTCCCATTTCAGAATATACATTCGATCACAATTGGAAAATGTCCAAAATATGTAAAAAGCACAAAATTGAGACTGGCCACAGGATTGAGGAATGTCCCGTCTATTCAATCTAGAGGCCTATTTGGGGCCATTGCCGGTTTCATTGAAGGGGGGTGGACAGGGATGGTAGATGGATGGTACGGTTATCACCATCAAAATGAGCAGGGGTCAGGATATGCAGCCGACCTGAAGAGCACACAGAATGCCATTGACGAGATTACTAACAAAGTAAATTCTGTTATTGAAAAGATGAATACACAGTTCACAGCAGTAGGTAAAGAGTTCAACCACCTGGAAAAAAGAATAGAAAATTTAAATAAAAAAGTTGATGATGGTTTCCTGGACATTTGGACTTACAATGCCGAACTGTTGGTTCTATTGGAAAATGAAAGAACTTTGGACTACCACGATTCAAATGTGAAGAACTTATATGAAAAGGTAAGAAGCCAGTTAAAAAACAATGCCAAGGAAATTGGAAACGGCTGCTTTGAATTTTACCACAAATGCGATAACACGTGCATGGAAAGTGTCAAAAATGGGACTTATGACTACCCAAAATACTCAGAGGAAGCAAAATTAAACAGAGAAGAAATAGATGGGGTAAAGCTGGAATCAACAAGGATTTACCAGATTTTGGCGATCTATTCAACTGTCGCCAGTTCATTGGTACTGGTAGTCTCCCTGGGGGCAATCAGTTTCTGGATGTGCTCTAATGGGTCTCTACAGTGTAGAATATGTATTTAA

>LC638130.1Influenza A virus H1N101701

ATGAAGGCAATACTAGTAGTTCTGCTATATACATTTGCAACCGCAAATGCAGACACATTATGTATAGGTTATCATGCGAACAATTCAACAGACACTGTAGACACAATACTAGAAAAGAATGTAACAGTAACACACTCTGTTAACCTTCTAGAAGACAAGCATAACGGGAAACTATGCAAACTAAGAGGGGTAGCCCCATTGCATTTGGGTAAATGTAACATTGCTGGCTGGATCCTGGGAAATCCAGAGTGTGAATCACTCTCCACAGCAAGCTCATGGTCCTACATTGTGGAAACATCTAGTTCAGACAATGGAACGTGTTACCCAGGAGATTTCATCGATTATGAGGAGCTAAGAGAGCAATTGAGCTCAGTGTCATCATTTGAAAGGTTTGAGATATTCCCCAAGACAAGTTCATGGCCCAATCATGACTCGAACAAAGGTGTAACGGCAGCATGTCCTCATGCTGGAGCAAAAAGCTTCTACAAAAATTTAATATGGCTAGTTAAAAAAGGAAATTCATACCCAAAGCTCAGCAAATCCTACATTAATGATAAAGGGAAAGAAGTCCTCGTGCTATGGGGCATTCACCATCCATCTACTAGTGCTGACCAACAAAGTCTCTATCAGAATGCAGATGCATATGTTTTTGTGGGGACATCAAGATACAGCAAGAAGTTCAAGCCGGAAATAGCAATAAGACCCAAAGTGAGGGATCAAGAAGGGAGAATGAACTATTACTGGACACTAGTAGAGCCGGGAGACAAAATAACATTCGAAGCAACTGGAAATCTAGTGGTACCGAGATATGCATTCGCAATGGAAAGAAATGCTGGATCTGGTATTATCATTTCAGATACACCAGTCCACGATTGCAATACAACTTGTCAGACACCCAAGGGTGCTATAAACACCAGCCTCCCATTTCAGAATATACATCCGATCACAATTGGAAAATGTCCAAAATATGTAAAAAGCACAAAATTGAGACTGGCCACAGGATTGAGGAATGTCCCGTCTATTCAATCTAGAGGCCTATTTGGGGCCATTGCCGGTTTCATTGAAGGGGGGTGGACAGGGATGGTAGATGGATGGTACGGTTATCACCATCAAAATGAGCAGGGGTCAGGATATGCAGCCGACCTGAAGAGCACACAGAATGCCATTGACGAGATTACTAACAAGGTAAATTCTGTTATTGAAAAGATGAATACACAGTTCACAGCAGTAGGTAAAGAGTTCAACCACCTGGAAAAAAGAATAGAGAATTTAAATAAAAAGGTTGATGATGGTTTCCTGGACATTTGGACTTACAATGCCGAACTGTTGGTTCTATTGGAAAATGAAAGAACTTTGGACTACCACGATTCAAATGTGAAGAACTTATATGAAAAGGTAAGAAGCCAGTTAAAAAACAATGCCAAGGAAATTGGAAACGGCTGCTTTGAATTTTACCACAAATGCGATAACACGTGCATGGAAAGTGTCAAAAATGGGACTTATGACTACCCAAAATACTCAGAGGAAGCAAAATTAAACAGAGAAGAAATAGATGGGGTAAAGCTGGAATCAACAAGGATTTACCAGATTTTGGCGATCTATTCAACTGTCGCCAGTTCATTGGTACTGGTAGTCTCCCTGGGGGCAATCAGTTTCTGGATGTGCTCTAATGGGTCTCTACAGTGTAGAATATGTATTTAA

>LC638129.1Influenza A virus H1N101701

ATGAAGGCAATACTAGTAGTTCTGCTATATACATTTGCAACCGCAAATGCAGACACATTATGTATAGGTTATCATGCGAACAATTCAACTGACACTGTAGACACAGTACTAGAAAAGAATGTAACAGTAACACACTCTGTTAACCTTCTAGAAGACAAGCATAACGGGAAACTATGCAAACTAAGAGGGGTAGCCCCATTGCATTTGGGTAAATGTAACATTGCTGGCTGGATCCTGGGAAATCCAGAGTGTGAATCACTCTCCACAGCAAGCTCATGGTCCTACATTGTGGAAACATCTAGTTCAGACAATGGAACGTGTTACCCAGGAGATTTCATCGATTATGAGGAGCTAAGAGAGCAATTGAGCTCAGTGTCATCATTTGAAAGGTTTGAGATATTCCCCAAGACAAGTTCATGGCCCAATCATGACTCGAACAAAGGTGTAACGGCAGCATGTCCTCATGCTGGAGCAAAAAGCTTCTACAAAAATTTAATATGGCTAGTTAAAAAAGGAAATTCATACCCAAAGCTCAGCAAATCCTACATTAATGATAAAGGGAAAGAAGTCCTCGTGCTATGGGGCATTCACCATCCATCTACTAGTGCTGACCAACAAAGTCTCTATCAGAATGCAGATACATATGTTTTTGTGGGGACATCAAGATACAGCAAGAAGTTCAAGCCGGAAATAGCAATAAGACCCAAAGTGAGGGATCAAGAAGGGAGAATGAACTATTACTGGACACTAGTAGAGCCGGGAGACAAAATAACATTCGAAGCAACTGGAAATCTAGTGGTACCGAGATATGCATTCGCAATGGAAAGAAATGCTGGATCTGGTATTATCATTTCAGATACACCAGTCCACGATTGCAATACAACTTGTCAGACACCCAAGGGTGCTATAAACACCAGCCTCCCATTTCAGAATATACATCCGATCACAATTGGAAAATGTCCAAAATATGTAAAAAGCACAAAATTGAGACTGGCCACAGGATTGAGGAATGTCCCGTCTATTCAATCTAGAGGCCTATTTGGGGCCATTGCCGGTTTCATTGAAGGGGGGTGGACAGGGATGGTAGATGGATGGTACGGTTATCACCATCAAAATGAGCAGGGGTCAGGATATGCAGCCGACCTGAAGAGCACACAGAATGCCATTGACGAGATTACTAACAAAGTAAATTCTGTTATTGAAAAGATGAATACACAGTTCACAGCAGTAGGTAAAGAGTTCAACCACCTGGAAAAAAGAATAGAGAATTTAAATAAAAAAGTTGATGATGGTTTCCTGGACATTTGGACTTACAATGCCGAACTGTTGGTTCTATTGGAAAATGAAAGAACTTTGGACTACCACGATTCAAATGTGAAGAACTTATATGAAAAGGTAAGAAGCCAGTTAAAAAACAATGCCAAGGAAATTGGAAACGGCTGCTTTGAATTTTACCACAAATGCGATAACACGTGCATGGAAAGTGTCAAAAATGGGACTTATGACTACCCAAAATACTCAGAGGAAGCAAAATTAAACAGAGAAGAAATAGATGGGGTAAAGCTGGAATCAACAAGGATTTACCAGATTTTGGCGATCTATTCAACTGTCGCCAGTTCATTGGTACTGGTAGTCTCCCTGGGGGCAATCAGTTTCTGGATGTGCTCTAATGGGTCTCTACAGTGTAGAATATGTATTTAA

>LC638128.1Influenza A virus H1N101701

ATGAAGGCAATACTAGTAGTTCTGCTATATACATTTGCAACCGCAAATGCAGACACATTATGTATAGGTTATCATGCGAACAATTCAACAGACACTGTAGACACAATACTAGAAAAGAATGTAACAGTAACACACTCTGTTAACCTTCTAGAAGACAAGCATAACGGGAAACTATGCAAACTAAGAGGGGTAGCCCCATTGCATTTGGGTAAATGTAACATTGCTGGCTGGATCCTGGGAAATCCAGAGTGTGAATCACTCTCCACAGCAAGCTCATGGTCCTACATTGTGGAAACATCTAGTTCAGACAATGGAACGTGTTACCCAGGAGATTTCATCGATTATGAGGAGCTAAGAGAGCAATTGAGCTCAGTGTCATCATTTGAAAGGTTTGAGATATTCCCCAAGACAAGTTCATGGCCCAATCATGACTCGAACAAAGGTGTAACGGCAGCATGTCCTCATGCTGGAGCAAAAAGCTTCTACAAAAATTTAATATGGCTAGTTAAAAAAGGAAATTCATACCCAAAGCTCAGCAAATCCTACATTAATGATAAAGGGAAAGAAGTCCTCGTGCTATGGGGCATTCACCATCCATCTACTAGTGCTGACCAACAAAGTCTCTATCAGAATGCAGATGCATATGTTTTTGTGGGGACATCAAGATACAGCAAGAAGTTCAAGCCGGAAATAGCAATAAGACCCAAAGTGAGGGATCAAGAAGGGAGAATGAACTATTACTGGACACTAGTAGAGCCGGGAGACAAAATAACATTCGAAGCAACTGGAAATCTAGTGGTACCGAGATATGCATTCGCAATGGAAAGAAATGCTGGATCTGGTATTATCATTTCAGATACACCAGTCCACGATTGCAATACAACTTGTCAGACACCCAAGGGTGCTATAAACACCAGCCTCCCATTTCAGAATATACATCCGATCACAATTGGAAAATGTCCAAAATATGTAAAAAGCACAAAATTGAGACTGGCCACAGGATTGAGGAATGTCCCGTCTATTCAATCTAGAGGCCTATTTGGGGCCATTGCCGGTTTCATTGAAGGGGGGTGGACAGGGATGGTAGATGGATGGTACGGTTATCACCATCAAAATGAGCAGGGGTCAGGATATGCAGCCGACCTGAAGAGCACACAGAATGCCATTGACGAGATTACTAACAAGGTAAATTCTGTTATTGAAAAGATGAATACACAGTTCACAGCAGTAGGTAAAGAGTTCAACCACCTGGAAAAAAGAATAGAGAATTTAAATAAAAAGGTTGATGATGGTTTCCTGGACATTTGGACTTACAATGCCGAACTGTTGGTTCTATTGGAAAATGAAAGAACTTTGGACTACCACGATTCAAATGTGAAGAACTTATATGAAAAGGTAAGAAGCCAGTTAAAAAACAATGCCAAGGAAATTGGAAACGGCTGCTTTGAATTTTACCACAAATGCGATAACACGTGCATGGAAAGTGTCAAAAATGGGACTTATGACTACCCAAAATACTCAGAGGAAGCAAAATTAAACAGAGAAGAAATAGATGGGGTAAAGCTGGAATCAACAAGGATTTACCAGATTTTGGCGATCTATTCAACTGTCGCCAGTTCATTGGTACTGGTAGTCTCCCTGGGGGCAATCAGTTTCTGGATGTGCTCTAATGGGTCTCTACAGTGTAGAATATGTATTTAA

>LC638126.1Influenza A virus H1N101701

ATGAAGGCAATACTAGTAGTTCTGCTATATACATTTGCAACCGCAAATGCAGACACATTATGTATAGGTTATCATGCGAACAATTCAACAGACACTGTAGACACAATACTAGAAAAGAATGTAACAGTAACACACTCTGTTAACCTTCTAGAAGACAAGCATAACGGGAAACTATGCAAACTAAGAGGGGTAGCCCCATTGCATTTGGGTAAATGTAACATTGCTGGCTGGATCCTGGGAAATCCAGAGTGTGAATCACTCTCCACAGCAAGCTCATGGTCCTACATTGTGGAAACATCTAGTTCAGACAATGGAACGTGTTACCCAGGAGATTTCATCGATTATGAGGAGCTAAGAGAGCAATTGAGCTCAGTGTCATCATTTGAAAGGTTTGAGATATTCCCCAAGACAAGTTCATGGCCCAATCATGACTCGAACAAAGGTGTAACGGCAGCATGTCCTCATGCTGGAGCAAAAAGCTTCTACAAAAATTTAATATGGCTAGTTAAAAAAGGAAATTCATACCCAAAGCTCAGCAAATCCTACATTAATGATAAAGGGAAAGAAGTCCTCGTGCTATGGGGCATTCACCATCCATCTACTAGTGCTGACCAACAAAGTCTCTATCAGAATGCAGATGCATATGTTTTTGTGGGGACATCAAGATACAGCAAGAAGTTCAAGCCGGAAATAGCAATAAGACCCAAAGTGAGGGATCAAGAAGGGAGAATGAACTATTACTGGACACTAGTAGAGCCGGGAGACAAAATAACATTCGAAGCAACTGGAAATCTAGTGGTACCGAGATATGCATTCGCAATGGAAAGAAATGCTGGATCTGGTATTATCATTTCAGATACACCAGTCCACGATTGCAATACAACTTGTCAGACACCCAAGGGTGCTATAAACACCAGCCTCCCATTTCAGAATATACATCCGATCACAATTGGAAAATGTCCAAAATATGTAAAAAGCACAAAATTGAGACTGGCCACAGGATTGAGGAATGTCCCGTCTATTCAATCTAGAGGCCTATTTGGGGCCATTGCCGGTTTCATTGAAGGGGGGTGGACAGGGATGGTAGATGGATGGTACGGTTATCACCATCAAAATGAGCAGGGGTCAGGATATGCAGCCGACCTGAAGAGCACACAGAATGCCATTGACGAGATTACTAACAAGGTAAATTCTGTTATTGAAAAGATGAATACACAGTTCACAGCAGTAGGTAAAGAGTTCAACCACCTGGAAAAAAGAATAGAGAATTTAAATAAAAAGGTTGATGATGGTTTCCTGGACATTTGGACTTACAATGCCGAACTGTTGGTTCTATTGGAAAATGAAAGAACTTTGGACTACCACGATTCAAATGTGAAGAACTTATATGAAAAGGTAAGAAGCCAGTTAAAAAACAATGCCAAGGAAATTGGAAACGGCTGCTTTGAATTTTACCACAAATGCGATAACACGTGCATGGAAAGTGTCAAAAATGGGACTTATGACTACCCAAAATACTCAGAGGAAGCAAAATTAAACAGAGAAGAAATAGATGGGGTAAAGCTGGAATCAACAAGGATTTACCAGATTTTGGCGATCTATTCAACTGTCGCCAGTTCATTGGTACTGGTAGTCTCCCTGGGGGCAATCAGTTTCTGGATGTGCTCTAATGGGTCTCTACAGTGTAGAATATGTATTTAA

>LC638123.1Influenza A virus H1N101701

ATGAAGGCAATACTAGTAGTTCTGCTATATACATTTGCAACCGCAAATGCAGACACATTATGTATAGGTTATCATGCGAACAATTCAACAGACACTGTAGACACAATACTAGAAAAGAATGTAACAGTAACACACTCTGTTAACCTTCTAGAAGACAAGCATAACGGGAAACTATGCAAACTAAGAGGGGTAGCCCCATTGCATTTGGGTAAATGTAACATTGCTGGCTGGATCCTGGGAAATCCAGAGTGTGAATCACTCTCCACAGCAAGCTCATGGTCCTACATTGTGGAAACATCTAGTTCAGACAATGGAACGTGTTACCCAGGAGATTTCATCGATTATGAGGAGCTAAGAGAGCAATTGAGCTCAGTGTCATCATTTGAAAGGTTTGAGATATTCCCCAAGACAAGTTCATGGCCCAATCATGACTCGAACAAAGGTGTAACGGCAGCATGTCCTCATGCTGGAGCAAAAAGCTTCTACAAAAATTTAATATGGCTAGTTAAAAAAGGAAATTCATACCCAAAGCTCAGCAAATCCTACATTAATGATAAAGGGAAAGAAGTCCTCGTGCTATGGGGCATTCACCATCCATCTACTAGTGCTGACCAACAAAGTCTCTATCAGAATGCAGATGCATATGTTTTTGTGGGGACATCAAGATACAGCAAGAAGTTCAAGCCGGAAATAGCAATAAGACCCAAAGTGAGGGATCAAGAAGGGAGAATGAACTATTACTGGACACTAGTAGAGCCGGGAGACAAAATAACATTCGAAGCAACTGGAAATCTAGTGGTACCGAGATATGCATTCGCAATGGAAAGAAATGCTGGATCTGGTATTATCATTTCAGATACACCAGTCCACGATTGCAATACAACTTGTCAGACACCCAAGGGTGCTATAAACACCAGCCTCCCATTTCAGAATATACATCCGATCACAATTGGAAAATGTCCAAAATATGTAAAAAGCACAAAATTGAGACTGGCCACAGGATTGAGGAATGTCCCGTCTATTCAATCTAGAGGCCTATTTGGGGCCATTGCCGGTTTCATTGAAGGGGGGTGGACAGGGATGGTAGATGGATGGTACGGTTATCACCATCAAAATGAGCAGGGGTCAGGATATGCAGCCGACCTGAAGAGCACACAGAATGCCATTGACGAGATTACTAACAAGGTAAATTCTGTTATTGAAAAGATGAATACACAGTTCACAGCAGTAGGTAAAGAGTTCAACCACCTGGAAAAAAGAATAGAGAATTTAAATAAAAAGGTTGATGATGGTTTCCTGGACATTTGGACTTACAATGCCGAACTGTTGGTTCTATTGGAAAATGAAAGAACTTTGGACTACCACGATTCAAATGTGAAGAACTTATATGAAAAGGTAAGAAGCCAGTTAAAAAACAATGCCAAGGAAATTGGAAACGGCTGCTTTGAATTTTACCACAAATGCGATAACACGTGCATGGAAAGTGTCAAAAATGGGACTTATGACTACCCAAAATACTCAGAGGAAGCAAAATTAAACAGAGAAGAAATAGATGGGGTAAAGCTGGAATCAACAAGGATTTACCAGATTTTGGCGATCTATTCAACTGTCGCCAGTTCATTGGTACTGGTAGTCTCCCTGGGGGCAATCAGTTTCTGGATGTGCTCTAATGGGTCTCTACAGTGTAGAATATGTATTTAA

>LC638121.1Influenza A virus H1N101701

ATGAAGGCAATACTAGTAGTTCTGCTATATACATTTGCAACCGCAAATGCAGACACATTATGTATAGGTTATCATGCGAACAATTCAACAGACACTGTAGACACAATACTAGAAAAGAATGTAACAGTAACACACTCTGTTAACCTTCTAGAAGACAAGCATAACGGGAAACTATGCAAACTAAGAGGGGTAGCCCCATTGCATTTGGGTAAATGTAACATTGCTGGCTGGATCCTGGGAAATCCAGAGTGTGAATCACTCTCCACAGCAAGCTCATGGTCCTACATTGTGGAAACATCTAGTTCAGACAATGGAACGTGTTACCCAGGAGATTTCATCGATTATGAGGAGCTAAGAGAGCAATTGAGCTCAGTGTCATCATTTGAAAGGTTTGAGATATTCCCCAAGACAAGTTCATGGCCCAATCATGACTCGAACAAAGGTGTAACGGCAGCATGTCCTCATGCTGGAGCAAAAAGCTTCTACAAAAATTTAATATGGCTAGTTAAAAAAGGAAATTCATACCCAAAGCTCAGCAAATCCTACATTAATGATAAAGGGAAAGAAGTCCTCGTGCTATGGGGCATTCACCATCCATCTACTAGTGCTGACCAACAAAGTCTCTATCAGAATGCAGATGCATATGTTTTTGTGGAGACATCAAGATACAGCAAGAAGTTCAAGCCGGAAATAGCAATAAGACCCAAAGTGAGGGATCAAGAAGGGAGAATGAACTATTACTGGACACTAGTAGAGCCGGGAGACAAAATAACATTCGAAGCAACTGGAAATCTAGTGGTACCGAGATATGCATTCGCAATGGAAAGAAATGCTGGATCTGGTATTATCATTTCAGATACACCAGTCCACGATTGCAATACAACTTGTCAGACACCCAAGGGTGCTATAAACACCAGCCTCCCATTTCAGAATATACATCCGATCACAATTGGAAAATGTCCAAAATATGTAAAAAGCACAAAATTGAGACTGGCCACAGGATTGAGGAATGTCCCGTCTATTCAATCTAGAGGCCTATTTGGGGCCATTGCCGGTTTCATTGAAGGGGGGTGGACAGGGATGGTAGATGGATGGTACGGTTATCACCATCAAAATGAGCAGGGGTCAGGATATGCAGCCGACCTGAAGAGCACACAGAATGCCATTGACGAGATTACTAACAAGGTAAATTCTGTTATTGAAAAGATGAATACACAGTTCACAGCAGTAGGTAAAGAGTTCAACCACCTGGAAAAAAGAATAGAGAATTTAAATAAAAAGGTTGATGATGGTTTCCTGGACATTTGGACTTACAATGCCGAACTGTTGGTTCTATTGGAAAATGAAAGAACTTTGGACTACCACGATTCAAATGTGAAGAACTTATATGAAAAGGTAAGAAGCCAGTTAAAAAACAATGCCAAGGAAATTGGAAACGGCTGCTTTGAATTTTACCACAAATGCGATAACACGTGCATGGAAAGTGTCAAAAATGGGACTTATGACTACCCAAAATACTCAGAGGAAGCAAAATTAAACAGAGAAGAAATAGATGGGGTAAAGCTGGAATCAACAAGGATTTACCAGATTTTGGCGATCTATTCAACTGTCGCCAGTTCATTGGTACTGGTAGTCTCCCTGGGGGCAATCAGTTTCTGGATGTGCTCTAATGGGTCTCTACAGTGTAGAATATGTATTTAA

>LC638120.1Influenza A virus H1N101701

ATGAAGGCAATACTAGTAGTTCTGCTATATACATTTGCAACCGCAAATGCAGACACATTATGTATAGGTTATCATGCGAACAATTCAACAGACACTGTAGACACAATACTAGAAAAGAATGTAACAGTAACACACTCTGTTAACCTTCTAGAAGACAAGCATAACGGGAAACTATGCAAACTAAGAGGGGTAGCCCCATTGCATTTGGGTAAATGTAACATTGCTGGCTGGATCCTGGGAAATCCAGAGTGTGAATCACTCTCCACAGCAAGCTCATGGTCCTACATTGTGGAAACATCTAGTTCAGACAATGGAACGTGTTACCCAGGAGATTTCATCGATTATGAGGAGCTAAGAGAGCAATTGAGCTCAGTGTCATCATTTGAAAGGTTTGAGATATTCCCCAAGACAAGTTCATGGCCCAATCATGACTCGAACAAAGGTGTAACGGCAGCATGTCCTCATGCTGGAGCAAAAAGCTTCTACAAAAATTTAATATGGCTAGTTAAAAAAGGAAATTCATACCCAAAGCTCAGCAAATCCTACATTAATGATAAAGGGAAAGAAGTCCTCGTGCTATGGGGCATTCACCATCCATCTACTAGTGCTGACCAACAAAGTCTCTATCAGAATGCAGATGCATATGTTTTTGTGGGGACATCAAGATACAGCAAGAAGTTCAAGCCGGAAATAGCAATAAGACCCAAAGTGAGGGATCAAGAAGGGAGAATGAACTATTACTGGACACTAGTAGAGCCGGGAGACAAAATAACATTCGAAGCAACTGGAAATCTAGTGGTACCGAGATATGCATTCGCAATGGAAAGAAATGCTGGATCTGGTATTATCATTTCAGATACACCAGTCCACGATTGCAATACAACTTGTCAGACACCCAAGGGTGCTATAAACACCAGCCTCCCATTTCAGAATATACATCCGATCACAATTGGAAAATGTCCAAAATATGTAAAAAGCACAAAATTGAGACTGGCCACAGGATTGAGGAATGTCCCGTCTATTCAATCTAGAGGCCTATTTGGGGCCATTGCCGGTTTCATTGAAGGGGGGTGGACAGGGATGGTAGATGGATGGTACGGTTATCACCATCAAAATGAGCAGGGGTCAGGATATGCAGCCGACCTGAAGAGCACACAGAATGCCATTGACGAGATTACTAACAAGGTAAATTCTGTTATTGAAAAGATGAATACACAGTTCACAGCAGTAGGTAAAGAGTTCAACCACCTGGGAAAAAGAATAGAGAATTTAAATAAAAAGGTTGATGATGGTTTCCTGGACATTTGGACTTACAATGCCGAACTGTTGGTTCTATTGGAAAATGAAAGAACTTTGGACTACCACGATTCAAATGTGAAGAACTTATATGAAAAGGTAAGAAGCCAGTTAAAAAACAATGCCAAGGAAATTGGAAACGGCTGCTTTGAATTTTACCACAAATGCGATAACACGTGCATGGAAAGTGTCAAAAATGGGACTTATGACTACCCAAAATACTCAGAGGAAGCAAAATTAAACAGAGAAGAAATAGATGGGGTAAAGCTGGAATCAACAAGGATTTACCAGATTTTGGCGATCTATTCAACTGTCGCCAGTTCATTGGTACTGGTAGTCTCCCTGGGGGCAATCAGTTTCTGGATGTGCTCTAATGGGTCTCTACAGTGTAGAATATGTATTTAA

>LC638119.1Influenza A virus H1N101701

ATGAAGGCAATACTAGTAGTTCTGCTATATACATTTGCAACCGCAAATGCAGACACATTATGTATAGGTTATCATGCGAACAATTCAACAGACACTGTAGACACAATACTAGAAAAGAATGTAACAGTAACACACTCTGTTAACCTTCTAGAAGACAAGCATAACGGGAAACTATGCAAACTAAGAGGGGTAGCCCCATTGCATTTGGGTAAATGTAACATTGCTGGCTGGATCCTGGGAAATCCAGAGTGTGAATCACTCTCCACAGCAAGCTCATGGTCCTACATTGTGGAAACATCTAGTTCAGACAATGGAACGTGTTACCCAGGAGATTTCATCGATTATGAGGAGCTAAGAGAGCAATTGAGCTCAGTGTCATCATTTGAAAGGTTTGAGATATTCCCCAAGACAAGTTCATGGCCCAATCATGACTCGAACAAAGGTGTAACGGCAGCATGTCCTCATGCTGGAGCAAAAAGCTTCTACAAAAATTTAATATGGCTAGTTAAAAAAGGAAATTCATACCCAAAGCTCAGCAAATCCTACATTAATGATAAAGGGAAAGAAGTCCTCGTGCTATGGGGCATTCACCATCCATCTACTAGTGCTGACCAACAAAGTCTCTATCAGAATGCAGATGCATATGTTTTTGTGGGGACATCAAGATACAGCAAGAAGTTCAAGCCGGAAATAGCAATAAGACCCAAAGTGAGGGATCAAGAAGGGAGAATGAACTATTACTGGACACTAGTAGAGCCGGGAGACAAAATAACATTCGAAGCAACTGGAAATCTAGTGGTACCGAGATATGCATTCGCAATGGAAAGAAATGCTGGATCTGGTATTATCATTTCAGATACACCAGTCCACGATTGCAATACAACTTGTCAGACACCCAAGGGTGCTATAAACACCAGCCTCCCATTTCAGAATATACATCCGATCACAATTGGAAAATGTCCAAAATATGTAAAAAGCACAAAATTGAGACTGGCCACAGGATTGAGGAATGTCCCGTCTATTCAATCTAGAGGCCTATTTGGGGCCATTGCCGGTTTCATTGAAGGGGGGTGGACAGGGATGGTAGATGGATGGTACGGTTATCACCATCAAAATGAGCAGGGGTCAGGATATGCAGCCGACCTGAAGAGCACACAGAATGCCATTGACGAGATTACTAACAAGGTAAATTCTGTTATTGAAAAGATGAATACACAGTTCACAGCAGTAGGTAAAGAGTTCAACCACCTGGAAAAAAGAATAGAGAATTTAAATAAAAAGGTTGATGATGGTTTCCTGGACATTTGGACTTACAATGCCGAACTGTTGGTTCTATTGGAAAATGAAAGAACTTTGGACTACCACGATTCAAATGTGAAGAACTTATATGAAAAGGTAAGAAGCCAGTTAAAAAACAATGCCAAGGAAATTGGAAACGGCTGCTTTGAATTTTACCACAAATGCGATAACACGTGCATGGAAAGTGTCAAAAATGGGACTTATGACTACCCAAAATACTCAGAGGAAGCAAAATTAAACAGAGAAGAAATAGATGGGGTAAAGCTGGAATCAACAAGGATTTACCAGATTTTGGCGATCTATTCAACTGTCGCCAGTTCATTGGTACTGGTAGTCTCCCTGGGGGCAATCAGTTTCTGGATGTGCTCTAATGGGTCTCTACAGTGTAGAATATGTATTTAA

>LC638118.1Influenza A virus H1N101701

ATGAAGGCAATACTAGTAGTTCTGCTATATACATTTGCAACCGCAAATGCAGACACATTATGTATAGGTTATCATGCGAACAATTCAACAGACACTGTAGACACAATACTAGAAAAGAATGTAACAGTAACACACTCTGTTAACCTTCTAGAAGACAAGCATAACGGGAAACTATGCAAACTAAGAGGGGTAGCCCCATTGCATTTGGGTAAATGTAACATTGCTGGCTGGATCCTGGGAAATCCAGAGTGTGAATCACTCTCCACAGCAAGCTCATGGTCCTACATTGTGGAAACATCTAGTTCAGACAATGGAACGTGTTACCCAGGAGATTTCATCGATTATGAGGAGCTAAGAGAGCAATTGAGCTCAGTGTCATCATTTGAAAGGTTTGAGATATTCCCCAAGACAAGTTCATGGCCCAATCATGACTCGAACAAAGGTGTAACGGCAGCATGTCCTCATGCTGGAGCAAAAAGCTTCTACAAAAATTTAATATGGCTAGTTAAAAAAGGAAATTCATACCCAAAGCTCAGCAAATCCTACATTAATGATAAAGGGAAAGAAGTCCTCGTGCTATGGGGCATTCACCATCCATCTACTAGTGCTGACCAACAAAGTCTCTATCAGAATGCAGATGCATATGTTTTTGTGGGGACATCAAGATACAGCAAGAAGTTCAAGCCGGAAATAGCAATAAGACCCAAAGTGAGGGATCAAGAAGGGAGAATGAACTATTACTGGACACTAGTAGAGCCGGGAGACAAAATAACATTCGAAGCAACTGGAAATCTAGTGGTACCGAGATATGCATTCGCAATGGAAAGAAATGCTGGATCTGGTATTATCATTTCAGATACACCAGTCCACGATTGCAATACAACTTGTCAGACACCCAAGGGTGCTATAAACACCAGCCTCCCATTTCAGAATATACATCCGATCACAATTGGAAAATGTCCAAAATATGTAAAAAGCACAAAATTGAGACTGGCCACAGGATTGAGGAATGTCCCGTCTATTCAATCTAGAGGCCTATTTGGGGCCATTGCCGGTTTCATTGAAGGGGGGTGGACAGGGATGGTAGATGGATGGTACGGTTATCACCATCAAAATGAGCAGGGGTCAGGATATGCAGCCGACCTGAAGAGCACACAGAATGCCATTGACGAGATTACTAACAAGGTAAATTCTGTTATTGAAAAGATGAATACACAGTTCACAGCAGTAGGTAAAGAGTTCAACCACCTGGAAAAAAGAATAGAGAATTTAAATAAAAAGGTTGATGATGGTTTCCTGGACATTTGGACTTACAATGCCGAACTGTTGGTTCTATTGGAAAATGAAAGAACTTTGGACTACCACGATTCAAATGTGAAGAACTTATATGAAAAGGTAAGAAGCCAGTTAAAAAACAATGCCAAGGAAATTGGAAACGGCTGCTTTGAATTTTACCACAAATGCGATAACACGTGCATGGAAAGTGTCAAAAATGGGACTTATGACTACCCAAAATACTCAGAGGAAGCAAAATTAAACAGAGAAGAAATAGATGGGGTAAAGCTGGAATCAACAAGGATTTACCAGATTTTGGCGATCTATTCAACTGTCGCCAGTTCATTGGTACTGGTAGTCTCCCTGGGGGCAATCAGTTTCTGGATGTGCTCTAATGGGTCTCTACAGTGTAGAATATGTATTTAA

>LC638117.1Influenza A virus H1N101701

ATGAAGGCAATACTAGTAGTTCTGCTATATACATTTGCAACCGCAAATGCAGACACATTATGTATAGGTTATCATGCGAACAATTCAACAGACACTGTAGACACAATACTAGAAAAGAATGTAACAGTAACACACTCTGTTAACCTTCTAGAAGACAAGCATAACGGGAAACTATGCAAACTAAGAGGGGTAGCCCCATTGCATTTGGGTAAATGTAACATTGCTGGCTGGATCCTGGGAAATCCAGAGTGTGAATCACTCTCCACAGCAAGCTCATGGTCCTACATTGTGGAAACATCTAGTTCAGACAATGGAACGTGTTACCCAGGAGATTTCATCGATTATGAGGAGCTAAGAGAGCAATTGAGCTCAGTGTCATCATTTGAAAGGTTTGAGATATTCCCCAAGACAAGTTCATGGCCCAATCATGACTCGAACAAAGGTGTAACGGCAGCATGTCCTCATGCTGGAGCAAAAAGCTTCTACAAAAATTTAATATGGCTAGTTAAAAAAGGAAATTCATACTCAAAGCTCAGCAAATCCTACATTAATGATAAAGGGAAAGAAGTCCTCGTGCTATGGGGCATTCACCATCCATCTACTAGTGCTGACCAACAAAGTCTCTATCAGAATGCAGATGCATATGTTTTTGTGGGGACATCAAGATACAGCAAGAAGTTCAAGCCGGAAATAGCAATAAGACCCAAAGTGAGGGATCAAGAAGGGAGAATGAACTATTACTGGACACTAGTAGAGCCGGGAGACAAAATAACATTCGAAGCAACTGGAAATCTAGTGGTACCGAGATATGCATTCGCAATGGAAAGAAATGCTGGATCTGGTATTATCATTTCAGATACACCAGTCCACGATTGCAATACAACTTGTCAGACACCCAAGGGTGCTATAAACACCAGCCTCCCATTTCAGAATATACATCCGATCACAATTGGAAAATGTCCAAAATTTGTAAAAAGCACAAAATTGAGACTGGCCACAGGATTGAGGAATGTCCCGTCTATTCAATCTAGAGGCCTATTTGGGGCCATTGCCGGTTTCATTGAAGGGGGGTGGACAGGGATGGTAGATGGATGGTACGGTTATCACCATCAAAATGAGCAGGGGTCAGGATATGCAGCCGACCTGAAGAGCACACAGAATGCCATTGACGAGATTACTAACAAAGTAAATTCTGTTATTGAAAAGATGAATACACAGTTCACAGCAGTAGGTAAAGAGTTCAACCACCTGGAAAAAAGAATAGAGAATTTAAATAAAAAGGTTGATGATGGTTTCCTGGACATTTGGACTTACAATGCCGAACTGTTGGTTCTATTGGAAAATGAAAGAACTTTGGACTACCACGATTCAAATGTGAAGAACTTATATGAAAAGGTAAGAAGCCAGTTAAAAAACAATGCCAAGGAAATTGGAAACGGCTGCTTTGAATTTTACCACAAATGCGATAACACGTGCATGGAAAGTGTCAAAAATGGGACTTATGACTACCCAAAATACTCAGAGGAAGCAAAATTAAACAGAGAAGAAATAGATGGGGTAAAGCTGGAATCAACAAGGATTTACCAGATTTTGGCGATCTATTCAACTGTCGCCAGTTCATTGGTACTGGTAGTCTCCCTGGGGGCAATCAGTTTCTGGATGTGCTCTAATGGGTCTCTACAGTGTAGAATATGTATTTAA

>LC638116.1Influenza A virus H1N101701

ATGAAGGCAATACTAGTAGTTCTGCTATATACATTTGCAACCGCAAATGCAGACACATTATGTATAGGTTATCATGCGAACAATTCAACAGACACTGTAGACACAATACTAGAAAAGAATGTAACAGTAACACACTCTGTTAACCTTCTAGAAGACAAGCATAACGGGAAACTATGCAAACTAAGAGGGGTAGCCCCATTGCATTTGGGTAAATGTAACATTGCTGGCTGGATCCTGGGAAATCCAGAGTGTGAATCACTCTCCACAGCAAGCTCATGGTCCTACATTGTGGAAACATCTAGTTCAGACAATGGAACGTGTTACCCAGGAGATTTCATCGATTATGAGGAGCTAAGAGAGCAATTGAGCTCAGTGTCATCATTTGAAAGGTTTGAGATATTCCCCAAGACAAGTTCATGGCCCAATCATGACTCGAACAAAGGTGTAACGGCAGCATGTCCTCATGCTGGAGCAAAAAGCTTCTACAAAAATTTAATATGGCTAGTTAAAAAAGGAAATTCATACTCAAAGCTCAGCAAATCCTACATTAATGATAAAGGGAAAGAAGTCCTCGTGCTATGGGGCATTCACCATCCATCTACTAGTGCTGACCAACAAAGTCTCTATCAGAATGCAGATGCATATGTTTTTGTGGGGACATCAAGATACAGCAAGAAGTTCAAGCCGGAAATAGCAATAAGACCCAAAGTGAGGGATCAAGAAGGGAGAATGAACTATTACTGGACACTAGTAGAGCCGGGAGACAAAATAACATTCGAAGCAACTGGAAATCTAGTGGTACCGAGATATGCATTCGCAATGGAAAGAAATGCTGGATCTGGTATTATCATTTCAGATACACCAGTCCACGATTGCAATACAACTTGTCAGACACCCAAGGGTGCTATAAACACCAGCCTCCCATTTCAGAATATACATCCGATCACAATTGGAAAATGTCCAAAATTTGTAAAAAACACAAAATTGAGACTGGCCACAGGATTGAGGAATGTCCCGTCTATTCAATCTAGAGGCCTATTTGGGGCCATTGCCGGTTTCATTGAAGGGGGGTGGACAGGGATGGTAGATGGATGGTACGGTTATCACCATCAAAATGAGCAGGGGTCAGGATATGCAGCCGACCTGAAGAGCACACAGAATGCCATTGACGAGATTACTAACAAAGTAAATTCTGTTATTGAAAAGATGAATACACAGTTCACAGCAGTAGGTAAAGAGTTCAACCACCTGGAAAAAAGAATAGAGAATTTAAATAAAAAGGTTGATGATGGTTTCCTGGACATTTGGACTTACAATGCCGAACTGTTGGTTCTATTGGAAAATGAAAGAACTTTGGACTACCACGATTCAAATGTGAAGAACTTATATGAAAAGGTAAGAAGCCAGTTAAAAAACAATGCCAAGGAAATTGGAAACGGCTGCTTTGAATTTTACCACAAATGCGATAACACGTGCATGGAAAGTGTCAAAAATGGGACTTATGACTACCCAAAATACTCAGAGGAAGCAAAATTAAACAGAGAAGAAATAGATGGGGTAAAGCTGGAATCAACAAGGATTTACCAGATTTTGGCGATCTATTCAACTGTCGCCAGTTCATTGGTACTGGTAGTCTCCCTGGGGGCAATCAGTTTCTGGATGTGCTCTAATGGGTCTCTACAGTGTAGAATATGTATTTAA

>LC638115.1Influenza A virus H1N101701

ATGAAGGCAATACTAGTAGTTCTGCTATATACATTTGCAACCGCAAATGCAGACACATTATGTATAGGTTATCATGCGAACAATTCAACAGACACTGTAGACACAGTACTAGAAAAGAATGTAACAGTAACACACTCTGTTAACCTTCTAGAAGACAAGCATAACGGGAAACTATGCAAACTAAGAGGGGTAGCCCCATTGCATTTGGGTAAATGTAACATTGCTGGCTGGATCCTGGGAAATCCAGAGTGTGAATCACTCTCCACAGCAAGCTCATGGTCCTACATTGTGGAAACATCTAGTTCAGACAATGGAACGTGTTACCCAGGAGATTTCATCGATTATGAGGAGCTAAGAGAGCAATTGAGCTCAGTGTCGTCATTTGAAAGATTTGAGATATTCCCCAAGACAAGTTCATGGCCCAATCATGACTCGAACAAAGGTGTAACGGCAGCATGTCCTCATGCTGGAGCAAAAAGCTTCTACAAAAATTTAATATGGCTAGTTAAAAAAGGAAATTCATACCCAAAGCTCAGCAAATCCTACATTAATGATAAAGGGAAAGAAGTCCTCGTGCTATGGGGCATTCACCATCCATCTACTAGTGCTGACCAACAAAGTCTCTATCAGAATGCAGATGCATATGTTTTTGTGGGGACTTCAAGATACAGCAAGAAGTTCAAGCCGGAAATAGCAATAAGACCCAAAGTGAGGGAACAAGAAGGGAGAATGAACTATTACTGGACACTAGTAGAGCCGGGAGACAAAATAACATTCGAAGCAACTGGAAATCTAGTGGTACCGAGATATGCATTCGCAATGGAAAGAAATGCTGGATCTGGTATTATCATTTCAGATACACCAGTCCACGATTGCAATACAACTTGTCAGACACCCAAGGGTGCTATAAACACCAGCCTCCCATTTCAGAATATACATTCGATCACAATTGGAAAATGTCCAAAATATGTAAAAAGCACAAAATTGAGACTGGCCACAGGATTGAGGAATGTCCCGTCTATTCAATCTAGAGGCCTATTTGGGGCCATTGCCGGTTTCATTGAAGGAGGGTGGACAGGGATGGTAGATGGATGGTACGGTTATCACCATCAAAATGAGCAGGGGTCAGGATATGCAGCCGACCTGAAGAGCACACAGAATGCCATTGACGAGATTACTAACAAAGTAAATTCTGTTATTGAAAAGATGAATACACAGTTCACAGCAGTAGGTAAAGAGTTCAACCACCTGGAAAAAAGAATAGAAAATTTAAATAAAAAAGTTGATGATGGTTTCCTGGACATTTGGACTTACAATGCCGAACTGTTGGTTCTATTGGAAAATGAAAGAACTTTGGACTACCACGATTCAAATGTGAAGAACTTATATGAAAAGGTAAGAAGCCAGTTAAAAAACAATGCCAAGGAAATTGGAAACGGCTGCTTTGAATTTTACCACAAATGCGATAACACGTGCATGGAAAGTGTCAAAAATGGGACTTATGACTACCCAAAATACTCAGAGGAAGCAAAATTAAACAGAGAAGAAATAGATGGGGTAAAGCTGGAATCAACAAGGATTTACCAGATTTTGGCGATCTATTCAACTGTCGCCAGTTCATTGGTACTGGTAGTCTCCCTGGGGGCAATCAGTTTCTGGATGTGCTCTAATGGGTCTCTACAGTGTAGAATATGTATTTAA

>LC638114.1Influenza A virus H1N101701

ATGAAGGCAATACTAGTAGTTCTGCTATATACATTTGCAACCGCAAATGCAGACACATTATGTATAGGTTATCATGCGAACAATTCAACAGACACTGTAGACACAATACTAGAAAAGAATGTAACAGTAACACACTCTGTTAACCTTCTAGAAGACAAGCATAACGGGAAACTATGCAAACTAAGAGGGGTAGCCCCATTGCATTTGGGTAAATGTAACATTGCTGGCTGGATCCTGGGAAATCCAGAGTGTGAATCACTCTCCACAGCAAGCTCATGGTCCTACATTGTGGAAACATCTAGTTCAGACAATGGAACGTGTTACCCAGGAGATTTCATCGATTATGAGGAGCTAAGAGAGCAATTGAGCTCAGTGTCATCATTTGAAAGGTTTGAGATATTCCCCAAGACAAGTTCATGGCCCAATCATGACTCGAACAAAGGTGTAACGGCAGCATGTCCTCATGCTGGAGCAAAAAGCTTCTACAAAAATTTAATATGGCTAGTTAAAAAAGGAAATTCATACCCAAAGCTCAGCAAATCCTACATTAATGATAAAGGGAAAGAAGTCCTCGTGCTATGGGGCATTCACCATCCATCTACTAGTGCTGACCAACAAAGTCTCTATCAGAATGCAGATGCATATGTTTTTGTGGGGACATCAAGATACAGCAAGAAGTTCAAGCCGGAAATAGCAATAAGACCCAAAGTGAGGGATCAAGAAGGGAGAATGAACTATTACTGGACACTAGTAGAGCCGGGAGACAAAATAACATTCGAAGCAACTGGAAATCTAGTGGTACCGAGATATGCATTCGCAATGGAAAGAAATGCTGGATCTGGTATTATCATTTCAGATACACCAGTCCACGATTGCAATACAACTTGTCAGACACCCAAGGGTGCTATAAACACCAGCCTCCCATTTCAGAATATACATCCGATCACAATTGGAAAATGTCCAAAATATGTAAAAAGCACAAAATTGAGACTGGCCACAGGATTGAGGAATGTCCCGTCTATTCAATCTAGAGGCCTATTTGGGGCCATTGCCGGTTTCATTGAAGGGGGGTGGACAGGGATGGTAGATGGATGGTACGGTTATCACCATCAAAATGAGCAGGGGTCAGGATATGCAGCCGACCTGAAGAGCACACAGAATGCCATTGACGAGATTACTAACAAGGTAAATTCTGTTATTGAAAAGATGAATACACAGTTCACAGCAGTAGGTAAAGAGTTCAACCACCTGGAAAAAAGAATAGAGAATTTAAATAAAAAGGTTGATGATGGTTTCCTGGACATTTGGACTTACAATGCCGAACTGTTGGTTCTATTGGAAAATGAAAGAACTTTGGACTACCACGATTCAAATGTGAAGAACTTATATGAAAAGGTAAGAAGCCAGTTAAAAAACAATGCCAAGGAAATTGGAAACGGCTGCTTTGAATTTTACCACAAATGCGATAACACGTGCATGGAAAGTGTCAAAAATGGGACTTATGACTACCCAAAATACTCAGAGGAAGCAAAATTAAACAGAGAAGAAATAGATGGGGTAAAGCTGGAATCAACAAGGATTTACCAGATTTTGGCGATCTATTCAACTGTCGCCAGTTCATTGGTACTGGTAGTCTCCCTGGGGGCAATCAGTTTCTGGATGTGCTCTAATGGGTCTCTACAGTGTAGAATATGTATTTAA

>LC638113.1Influenza A virus H1N101701

ATGAAGGCAATACTAGTAGTTCTGCTATATACATTTGCAACCGCAAATGCAGACACATTATGTATAGGTTATCATGCGAACAATTCAACAGACACTGTAGACACAGTACTAGAAAAGAATGTAACAGTAACACACTCTGTTAACCTTCTAGAAGACAAGCATAACGGGAAACTATGCAAACTAAGAGGGGTAGCCCCATTGCATTTGGGTAAATGTAACATTGCTGGCTGGATCCTGGGAAATCCAGAGTGTGAATCACTCTCTACAGCAAGCTCATGGTCCTACATTGTGGAAACATCTAGTTCAGACAATGGAACGTGTTACCCAGGAGATTTCATCGATTATGAGGAGCTAAGAGAGCAATTGAGCTCAGTGTCATCATTTGAAAGATTTGAGATATTCCCCAAGACAAGTTCATGGCCCAATCATGACTCGAACAAAGGTGTAACGGCAGCATGTCCTCATGCTGGAGCAAAAAGCTTCTACAAAAATTTAATATGGCTAGTTAAAAAAGGAAATTCATACCCAAAGCTCAGCAAATCCTACATTAATGATAAAGGAAAAGAAGTCCTTGTGCTATGGGGCATTCACCATCCATCTACTAGTGCTGACCAACAAAGTCTCTATCAGAATGCAGATGCATATGTTTTTGTGGGGACTTCAAGATACAGCAAGAAGTTCAAGCCGGAAATAGCAATAAGACCCAAAGTGAGGGAACAAGAAGGGAGAATGAACTATTACTGGACACTAGTAGAGCCGGGAGACAAAATAACATTCGAAGCAACTGGAAATCTAGTGGTACCGAGATATGCATTCGCAATGGAAAGAAATGCTGGATCTGGTATTATCATTTCAGATACACCAGTCCACGATTGCAATACAACTTGTCAGACACCCAAGGGTGCTATAAACACCAGCCTCCCATTTCAGAATATACATTCGATCACAATTGGAAAATGTCCAAAATATGTAAAAAGCACGAAATTGAGACTGGCCACAGGATTGAGGAATGTCCCGTCTATTCAATCTAGAGGCCTATTTGGGGCCATTGCCGGTTTCATTGAAGGGGGGTGGACAGGGATGGTAGATGGATGGTACGGTTATCACCATCAAAATGAGCAGGGGTCAGGATATGCAGCCGACCTGAAGAGCACACAGAATGCCATTGACGAGATTACTAACAAAGTAAATTCTGTTATTGAAAAGATGAATACACAGTTCACAGCAGTAGGTAAAGAGTTCAACCACCTGGAAAAAAGAATAGAAAATTTAAATAAAAAAGTTGATGATGGTTTCCTGGACATTTGGACTTACAATGCCGAACTGTTGGTTCTATTGGAAAATGAAAGAACTTTGGACTACCACGATTCAAATGTGAAGAACTTATATGAAAAGGTAAGAAGCCAGTTAAAAAACAATGCCAAGGAAATTGGAAACGGCTGCTTTGAATTTTACCACAAATGCGATAACACGTGCATGGAAAGTGTCAAAAATGGGACTTATGACTACCCAAAATACTCAGAGGAAGCAAAATTAAACAGAGAAGAAATAGATGGGGTAAAGCTGGAATCAACAAGGATTTACCAGATTTTGGCGATCTATTCAACTGTCGCCAGTTCATTGGTACTGGTAGTCTCCCTGGGGGCAATCAGTTTCTGGATGTGCTCTAATGGGTCTCTACAGTGTAGAATATGTATTTAA

>LC638112.1Influenza A virus H1N101701

ATGAAGGCAATACTAGTAGTTCTGCTATATACATTTGCAACCGCAAATGCAGACACATTATGTATAGGTTATCATGCGAACAATTCAACAGACACTGTAGACACAATACTAGAAAAGAATGTAACAGTAACACACTCTGTTAACCTTCTAGAAGACAAGCATAACGGGAAACTATGCAAACTAAGAGGGGTAGCCCCATTGCATTTGGGTAAATGTAACATTGCTGGCTGGATCCTGGGAAATCCAGAGTGTGAATCACTCTCCACAGCAAGCTCATGGTCCTACATTGTGGAAACATCTAGTTCAGACAATGGAACGTGTTACCCAGGAGATTTCATCGATTATGAGGAGCTAAGAGAGCAATTGAGCTCAGTGTCATCATTTGAAAGGTTTGAGATATTCCCCAAGACAAGTTCATGGCCCAATCATGACTCGAACAAAGGTGTAACGGCAGCATGTCCTCATGCTGGAGCAAAAAGCTTCTACAAAAATTTAATATGGCTAGTTAAAAAAGGAAATTCATACCCAAAGCTCAGCAAATCCTACATTAATGATAAAGGGAAAGAAGTCCTCGTGCTATGGGGCATTCACCATCCATCTACTAGTGCTGACCAACAAAGTCTCTATCAGAATGCAGATGCATATGTTTTTGTGGGGACATCAAGATACAGCAAGAAGTTCAAGCCGGAAATAGCAATAAGACCCAAAGTGAGGGATCAAGAAGGGAGAATGAACTATTACTGGACACTAGTAGAGCCGGGAGACAAAATAACATTCGAAGCAACTGGAAATCTAGTGGTACCGAGATATGCATTCGCAATGGAAAGAAATGCTGGATCTGGTATTATCATTTCAGATACACCAGTCCACGATTGCAATACAACTTGTCAGACACCCAAGGGTGCTATAAACACCAGCCTCCCATTTCAGAATATACATCCGATCACAATTGGAAAATGTCCAAAATATGTAAAAAGCACAAAATTGAGACTGGCCACAGGATTGAGGAATGTCCCGTCTATTCAATCTAGAGGCCTATTTGGGGCCATTGCCGGTTTCATTGAAGGGGGGTGGACAGGGATGGTAGATGGATGGTACGGTTATCACCATCAAAATGAGCAGGGGTCAGGATATGCAGCCGACCTGAAGAGCACACAGAATGCCATTGACGAGATTACTAACAAGGTAAATTCTGTTATTGAAAAGATGAATACACAGTTCACAGCAGTAGGTAAAGAGTTCAACCACCTGGAAAAAAGAATAGAGAATTTAAATAAAAAGGTTGATGATGGTTTCCTGGACATTTGGACTTACAATGCCGAACTGTTGGTTCTATTGGAAAATGAAAGAACTTTGGACTACCACGATTCAAATGTGAAGAACTTATATGAAAAGGTAAGAAGCCAGTTAAAAAACAATGCCAAGGAAATTGGAAACGGCTGCTTTGAATTTTACCACAAATGCGATAACACGTGCATGGAAAGTGTCAAAAATGGGACTTATGACTACCCAAAATACTCAGAGGAAGCAAAATTAAACAGAGAAGAAATAGATGGGGTAAAGCTGGAATCAACAAGGATTTACCAGATTTTGGCGATCTATTCAACTGTCGCCAGTTCATTGGTACTGGTAGTCTCCCTGGGGGCAATCAGTTTCTGGATGTGCTCTAATGGGTCTCTACAGTGTAGAATATGTATTTAA

>LC638111.1Influenza A virus H1N101701

ATGAAGGCAATACTAGTAGTTCTGCTATATACATTTGCAACCGCAAATGCAGACACATTATGTATAGGTTATCATGCGAACAATTCAACAGACACTGTAGACACAATACTAGAAAAGAATGTAACAGTAACACACTCTGTTAACCTTCTAGAAGACAAGCATAACGGGAAACTATGCAAACTAAGAGGGGTAGCCCCATTGCATTTGGGTAAATGTAACATTGCTGGCTGGATCCTGGGAAATCCAGAGTGTGAATCACTCTCCACAGCAAGCTCATGGTCCTACATTGTGGAAACATCTAGTTCAGACAATGGAACGTGTTACCCAGGAGATTTCATCGATTATGAGGAGCTAAGAGAGCAATTGAGCTCAGTGTCATCATTTGAAAGGTTTGAGATATTCCCCAAGACAAGTTCATGGCCCAATCATGACTCGAACAAAGGTGTAACGGCAGCATGTCCTCATGCTGGAGCAAAAAGCTTCTACAAAAATTTAATATGGCTAGTTAAAAAAGGAAATTCATACCCAAAGCTCAGCAAATCCTACATTAATGATAAAGGGAAAGAAGTCCTCGTGCTATGGGGCATTCACCATCCATCTACTAGTGCTGACCAACAAAGTCTCTATCAGAATGCAGATGCATATGTTTTTGTGGGGACATCAAGATACAGCAAGAAGTTCAAGCCGGAAATAGCAATAAGACCCAAAGTGAGGGATCAAGAAGGGAGAATGAACTATTACTGGACACTAGTAGAGCCGGGAGACAAAATAACATTCGAAGCAACTGGAAATCTAGTGGTACCGAGATATGCATTCGCAATGGAAAGAAATGCTGGATCTGGTATTATCATTTCAGATACACCAGTCCACGATTGCAATACAACTTGTCAGACACCCAAGGGTGCTATAAACACCAGCCTCCCATTTCAGAATATACATCCGATCACAATTGGAAAATGTCCAAAATATGTAAAAAGCACAAAATTGAGACTGGCCACAGGATTGAGGAATGTCCCGTCTATTCAATCTAGAGGCCTATTTGGGGCCATTGCCGGTTTCATTGAAGGGGGGTGGACAGGGATGGTAGATGGATGGTACGGTTATCACCATCAAAATGAGCAGGGGTCAGGATATGCAGCCGACCTGAAGAGCACACAGAATGCCATTGACGAGATTACTAACAAGGTAAATTCTGTTATTGAAAAGATGAATACACAGTTCACAGCAGTAGGTAAAGAGTTCAACCACCTGGAAAAAAGAATAGAGAATTTAAATAAAAAGGTTGATGATGGTTTCCTGGACATTTGGACTTACAATGCCGAACTGTTGGTTCTATTGGAAAATGAAAGAACTTTGGACTACCACGATTCAAATGTGAAGAACTTATATGAAAAGGTAAGAAGCCAGTTAAAAAACAATGCCAAGGAAATTGGAAACGGCTGCTTTGAATTTTACCACAAATGCGATAACACGTGCATGGAAAGTGTCAAAAATGGGACTTATGACTACCCAAAATACTCAGAGGAAGCAAAATTAAACAGAGAAGAAATAGATGGGGTAAAGCTGGAATCAACAAGGATTTACCAGATTTTGGCGATCTATTCAACTGTCGCCAGTTCATTGGTACTGGTAGTCTCCCTGGGGGCAATCAGTTTCTGGATGTGCTCTAATGGGTCTCTACAGTGTAGAATATGTATTTAA

>LC638109.1Influenza A virus H1N101701

ATGAAGGCAATACTAGTAGTTCTGCTATATACATTTGCAACCGCAAATGCAGACACATTATGTATAGGTTATCATGCGAACAATTCAACAGACACTGTAGACACAGTACTAGAAAAGAATGTAACAGTAACACACTCTGTTAACCTTCTAGAAGACAAGCATAACGGGAAACTATGCAAACTAAGAGGGGTAGCCCCATTGCATTTGGGTAAATGTAACATTGCTGGCTGGATCCTGGGAAATCCAGAGTGTGAATCACTCTCCACAGCAAGCTCATGGTCCTACATTGTGGAAACATCTAGTTCAGACAATGGAACGTGTTACCCAGGAGATTTCATCGATTATGAGGAGCTAAGAGAGCAATTGAGCTCAGTGTCGTCATTTGAAAGATTTGAGATATTCCCCAAGACAAGTTCATGGCCCAATCATGACTCGAACAAAGGTGTAACGGCAGCATGTCCTCATGCTGGAGCAAAAAGCTTCTACAAAAATTTAATATGGCTAGTTAAAAAAGGAAATTCATACCCAAAGCTCAGCAAATCCTACATTAATGATAAAGGGAAAGAAGTCCTCGTGCTATGGGGCATTCACCATCCATCTACTAGTGCTGACCAACAAAGTCTCTATCAGAATGCAGATGCATATGTTTTTGTGGGGACTTCAAGATACAGCAAGAAGTTCAAGCCGGAAATAGCAATAAGACCCAAAGTGAGGGAACAAGAAGGGAGAATGAACTATTACTGGACACTAGTAGAGCCGGGAGACAAAATAACATTCGAAGCAACTGGAAATCTAGTGGTACCGAGATATGCATTCGCAATGGAAAGAAATGCTGGATCTGGTATTATCATTTCAGATACACCAGTCCACGATTGCAATACAACTTGTCAGACACCCAAGGGTGCTATAAACACCAGCCTCCCATTTCAGAATATACATTCGATCACAATTGGAAAATGTCCAAAATATGTAAAAAGCACAAAATTGAGACTGGCCACAGGATTGAGGAATGTCCCGTCTATTCAATCTAGAGGCCTATTTGGGGCCATTGCCGGTTTCATTGAAGGAGGGTGGACAGGGATGGTAGATGGATGGTACGGTTATCACCATCAAAATGAGCAGGGGTCAGGATATGCAGCCGACCTGAAGAGCACACAGAATGCCATTGACGAGATTACTAACAAAGTAAATTCTGTTATTGAAAAGATGAATACACAGTTCACAGCAGTAGGTAAAGAGTTCAACCACCTGGAAAAAAGAATAGAAAATTTAAATAAAAAAGTTGATGATGGTTTCCTGGACATTTGGACTTACAATGCCGAACTGTTGGTTCTATTGGAAAATGAAAGAACTTTGGACTACCACGATTCAAATGTGAAGAACTTATATGAAAAGGTAAGAAGCCAGTTAAAAAACAATGCCAAGGAAATTGGAAACGGCTGCTTTGAATTTTACCACAAATGCGATAACACGTGCATGGAAAGTGTCAAAAATGGGACTTATGACTACCCAAAATACTCAGAGGAAGCAAAATTAAACAGAGAAGAAATAGATGGGGTAAAGCTGGAATCAACAAGGATTTACCAGATTTTGGCGATCTATTCAACTGTCGCCAGTTCATTGGTACTGGTAGTCTCCCTGGGGGCAATCAGTTTCTGGATGTGCTCTAATGGGTCTCTACAGTGTAGAATATGTATTTAA

>LC638108.1Influenza A virus H1N101701

ATGAAGGCAATACTAGTAGTTCTGCTATATACATTTGCAACCGCAAATGCAGACACATTATGTATAGGTTATCATGCGAACAATTCAACAGACACTGTAGACACAGTACTAGAAAAGAATGTAACAGTAACACACTCTGTTAACCTTCTAGAAGACAAGCATAACGGGAAACTATGCAAACTAAGAGGAGTAGCCCCATTGCATTTGGGTAAATGTAACATTGCTGGCTGGATCCTGGGAAATCCAGAATGTGAATCACTCTCCACAGCAAGCTCATGGTCCTACATTGTGGAAACATCTAGTTCAGACAATGGAACGTGTTACCCAGGAGATTTCATCGATTATGAGGAGCTAAGAGAGCAATTGAGCTCAGTGTCATCATTTGAAAGGTTTGAGATATTCCCCAAGACAAGTTCATGGCCCAATCATGACTCGAACAAAGGCGTAACGGCAGCATGTCCTCATGCTGGAGCAAAAAGCTTCTACAAAAATTTAATATGGCTAGTTAAAAAAGGAAATTCATACCCAAAGCTCAGCAAATCCTACATTAATGATAAAGGGAAAGAAGTCCTCGTGCTATGGGGCATTCACCATCCATCTACTAGTGCTGACCAACAAAGTCTCTATCAGAATGCAGATGCATATGTTTTTGTGGGGACATCAAGATACAGCAAGAAGTTCAAGCCGGAAATAGCAATAAGACCCAAAGTGAGGGATCAAGAAGGGAGAATGAACTATTACTGGACACTAGTAGAGCCGGGAGACAAAATAACATTCGAAGCAACTGGAAATCTAGTGGTACCGAGATATGCATTCGCAATGGAAAGAAATGCTGGATCTGGTATTATCATTTCAGATACACCAGTCCACGATTGCAATACAACTTGTCAGACACCCAAGGGTGCTATAAACACCAGCCTCCCATTTCAGAATATACATCCGATCACAATTGGAAAATGTCCAAAATATGTAAAAAGCACAAAATTGAGACTGGCCACAGGATTGAGGAATGTCCCGTCTATTCAATCTAGAGGCCTATTTGGGGCCATTGCCGGTTTCATTGAAGGGGGGTGGACAGGGATGGTAGATGGATGGTACGGTTATCACCATCAAAATGAGCAGGGGTCAGGATATGCAGCCGACCTGAAGAGCACACAGAATGCCATTGACGAGATTACTAACAAAGTAAATTCTGTTATTGAAAAGATGAATACACAGTTCACAGCAGTAGGTAAAGAGTTCAACCACCTGGAAAAAAGAATAGAGAATTTAAATAAAAAGGTTGATGATGGTTTCCTGGACATTTGGACTTACAATGCCGAACTGTTGGTTCTATTGGAAAATGAAAGAACTTTGGACTACCACGATTCAAATGTGAAGAACTTATATGAAAAGGTAAGAAGCCAGTTAAAAAACAATGCCAAGGAAATTGGAAACGGCTGCTTTGAATTTTACCACAAATGCGATAACACGTGCATGGAAAGTGTAAAAAATGGGACTTATGACTACCCAAAATACTCAGAGGAAGCAAAATTAAACAGAGAAGAAATAGATGGGGTAAAGCTGGAATCAACAAGGATTTACCAGATTTTGGCGATCTATTCAACTGTCGCCAGTTCATTGGTACTGGTAGTCTCCCTGGGGGCAATCAGTTTCTGGATGTGCTCTAATGGGTCTCTACAGTGTAGAATATGTATTTAA

>LC638107.1Influenza A virus H1N101701

ATGAAGGCAATACTAGTAGTTCTGCTATATACATTTGCAACCGCAAATGCAGACACATTATGTATAGGTTATCATGCGAACAATTCAACAGACACTGTAGACACAATACTAGAAAAGAATGTAACAGTAACACACTCTGTTAACCTTCTAGAAGACAAGCATAACGGGAAACTATGCAAACTAAGAGGGGTAGCCCCATTGCATTTGGGTAAATGTAACATTGCTGGCTGGATCCTGGGAAATCCAGAGTGTGAATCACTCTCCACAGCAAGCTCATGGTCCTACATTGTGGAAACATCTAGTTCAGACAATGGAACGTGTTACCCAGGAGATTTCATCGATTATGAGGAGCTAAGAGAGCAATTGAGCTCAGTGTCATCATTTGAAAGGTTTGAGATATTCCCCAAGACAAGTTCATGGCCCAATCATGACTCGAACAAAGGTGTAACGGCAGCATGTCCTCATGCTGGAGCAAAAAGCTTCTACAAAAATTTAATATGGCTAGTTAAAAAAGGAAATTCATACCCAAAGCTCAGCAAATCCTACATTAATGATAAAGGGAAAGAAGTCCTCGTGCTATGGGGCATTCACCATCCATCTACTAGTGCTGACCAACAAAGTCTCTATCAGAATGCAGATGCATATGTTTTTGTGGGGACATCAAGATACAGCAAGAAGTTCAAGCCGGAAATAGCAATAAGACCCAAAGTGAGGGATCAAGAAGGGAGAATGAACTATTACTGGACACTAGTAGAGCCGGGAGACAAAATAACATTCGAAGCAACTGGAAATCTAGTGGTACCGAGATATGCATTCGCAATGGAAAGAAATGCTGGATCTGGTATTATCATTTCAGATACACCAGTCCACGATTGCAATACAACTTGTCAGACACCCAAGGGTGCTATAAACACCAGCCTCCCATTTCAGAATATACATCCGATCACAATTGGAAAATGTCCAAAATATGTAAAAAGCACAAAATTGAGACTGGCCACAGGATTGAGGAATGTCCCGTCTATTCAATCTAGAGGCCTATTTGGGGCCATTGCCGGTTTCATTGAAGGGGGGTGGACAGGGATGGTAGATGGATGGTACGGTTATCACCATCAAAATGAGCAGGGGTCAGGATATGCAGCCGACCTGAAGAGCACACAGAATGCCATTGACGAGATTACTAACAAGGTAAATTCTGTTATTGAAAAGATGAATACACAGTTCACAGCAGTAGGTAAAGAGTTCAACCACCTGGAAAAAAGAATAGAGAATTTAAATAAAAAGGTTGATGATGGTTTCCTGGACATTTGGACTTACAATGCCGAACTGTTGGTTCTATTGGAAAATGAAAGAACTTTGGACTACCACGATTCAAATGTGAAGAACTTATATGAAAAGGTAAGAAGCCAGTTAAAAAACAATGCCAAGGAAATTGGAAACGGCTGCTTTGAATTTTACCACAAATGCGATAACACGTGCATGGAAAGTGTCAAAAATGGGACTTATGACTACCCAAAATACTCAGAGGAAGCAAAATTAAACAGAGAAGAAATAGATGGGGTAAAGCTGGAATCAACAAGGATTTACCAGATTTTGGCGATCTATTCAACTGTCGCCAGTTCATTGGTACTGGTAGTCTCCCTGGGGGCAATCAGTTTCTGGATGTGCTCTAATGGGTCTCTACAGTGTAGAATATGTATTTAA

>LC638106.1Influenza A virus H1N101701

ATGAAGGCAATACTAGTAGTTCTGCTATATACATTTGCAACCGCAAATGCAGACACATTATGTATAGGTTATCATGCGAACAATTCAACAGACACTGTAGACACAATACTAGAAAAGAATGTAACAGTAACACACTCTGTTAACCTTCTAGAAGACAAGCATAACGGGAAACTATGCAAACTAAGAGGGGTAGCCCCATTGCATTTGGGTAAATGTAACATTGCTGGCTGGATCCTGGGAAATCCAGAGTGTGAATCACTCTCCACAGCAAGCTCATGGTCCTACATTGTGGAAACATCTAGTTCAGACAATGGAACGTGTTACCCAGGAGATTTCATCGATTATGAGGAGCTAAGAGAGCAATTGAGCTCAGTGTCATCATTTGAAAGGTTTGAGATATTCCCCAAGACAAGTTCATGGCCCAATCATGACTCGAACAAAGGTGTAACGGCAGCATGTCCTCATGCTGGAGCAAAAAGCTTCTACAAAAATTTAATATGGCTAGTTAAAAAAGGAAATTCATACCCAAAGCTCAGCAAATCCTACATTAATGATAAAGGGAAAGAAGTCCTCGTGCTATGGGGCATTCACCATCCATCTACTAGTGCTGACCAACAAAGTCTCTATCAGAATGCAGATGCATATGTTTTTGTGGGGACATCAAGATACAGCAAGAAGTTCAAGCCGGAAATAGCAATAAGACCCAAAGTGAGGGATCAAGAAGGGAGAATGAACTATTACTGGACACTAGTAGAGCCGGGAGACAAAATAACATTCGAAGCAACTGGAAATCTAGTGGTACCGAGATATGCATTCGCAATGGAAAGAAATGCTGGATCTGGTATTATCATTTCAGATACACCAGTCCACGATTGCAATACAACTTGTCAGACACCCAAGGGTGCTATAAACACCAGCCTCCCATTTCAGAATATACATCCGATCACAATTGGAAAATGTCCAAAATATGTAAAAAGCACAAAATTGAGACTGGCCACAGGATTGAGGAATGTCCCGTCTATTCAATCTAGAGGCCTATTTGGGGCCATTGCCGGTTTCATTGAAGGGGGGTGGACAGGGATGGTAGATGGATGGTACGGTTATCACCATCAAAATGAGCAGGGGTCAGGATATGCAGCCGACCTGAAGAGCACACAGAATGCCATTGACGAGATTACTAACAAGGTAAATTCTGTTATTGAAAAGATGAATACACAGTTCACAGCAGTAGGTAAAGAGTTCAACCACCTGGAAAAAAGAATAGAGAATTTAAATAAAAAGGTTGATGATGGTTTCCTGGACATTTGGACTTACAATGCCGAACTGTTGGTTCTATTGGAAAATGAAAGAACTTTGGACTACCACGATTCAAATGTGAAGAACTTATATGAAAAGGTAAGAAGCCAGTTAAAAAACAATGCCAAGGAAATTGGAAACGGCTGCTTTGAATTTTACCACAAATGCGATAACACGTGCATGGAAAGTGTCAAAAATGGGACTTATGACTACCCAAAATACTCAGAGGAAGCAAAATTAAACAGAGAAGAAATAGATGGGGTAAAGCTGGAATCAACAAGGATTTACCAGATTTTGGCGATCTATTCAACTGTCGCCAGTTCATTGGTACTGGTAGTCTCCCTGGGGGCAATCAGTTTCTGGATGTGCTCTAATGGGTCTCTACAGTGTAGAATATGTATTTAA

>LC638105.1Influenza A virus H1N101701

ATGAAGGCAATACTAGTAGTTCTGCTATATACATTTGCAACCGCAAATGCAGACACATTATGTATAGGTTATCATGCGAACAATTCAACAGACACTGTAGACACAATACTAGAAAAGAATGTAACAGTAACACACTCTGTTAACCTTCTAGAAGACAAGCATAACGGGAAACTATGCAAACTAAGAGGGGTAGCCCCATTGCATTTGGGTAAATGTAACATTGCTGGCTGGATCCTGGGAAATCCAGAGTGTGAATCACTCTCCACAGCAAGCTCATGGTCCTACATTGTGGAAACATCTAGTTCAGACAATGGAACGTGTTACCCAGGAGATTTCATCGATTATGAGGAGCTAAGAGAGCAATTGAGCTCAGTGTCATCATTTGAAAGGTTTGAGATATTCCCCAAGACAAGTTCATGGCCCAATCATGACTCGAACAAAGGTGTAACGGCAGCATGTCCTCATGCTGGAGCAAAAAGCTTCTACAAAAATTTAATATGGCTAGTTAAAAAAGGAAATTCATACCCAAAGCTCAGCAAATCCTACATTAATGATAAAGGGAAAGAAGTCCTCGTGCTATGGGGCATTCACCATCCATCTACTAGTGCTGACCAACAAAGTCTCTATCAGAATGCAGATGCATATGTTTTTGTGGGGACTTCAAGATACAGCAAGAAGTTCAAGCCGGAAATAGCAATAAGACCCAAAGTGAGGGATCAAGAAGGGAGAATGAACTATTACTGGACACTAGTAGAGCCGGGAGACAAAATAACATTCGAAGCAACTGGAAATCTAGTGGTACCGAGATATGCATTCGCAATGGAAAGAAATGCTGGATCTGGTATTATCATTTCAGATACACCAGTCCACGATTGCAATACAACTTGTCAGACACCCAAGGGTGCTATAAACACCAGCCTCCCATTTCAGAATATACATTCGATCACAATTGGAAAATGTCCAAAATATGTAAAAAGCACAAAATTGAGACTGGCCACAGGATTGAGGAATGTCCCGTCTATTCAATCTAGAGGCCTATTTGGGGCCATTGCCGGTTTCATTGAAGGGGGGTGGACAGGGATGGTAGATGGATGGTACGGTTATCACCATCAAAATGAGCAGGGGTCAGGATATGCAGCCGACCTGAAGAGCACACAGAATGCCATTGACGAGATTACTAACAAAGTAAATTCTGTTATTGAAAAGATGAATACACAGTTCACAGCAGTAGGTAAAGAGTTCAACCACCTGGAAAAAAGAATAGAAAATTTAAATAAAAAGGTTGATGATGGTTTCCTGGACATTTGGACTTACAATGCCGAACTGTTGGTTCTATTGGAAAATGAAAGAACTTTGGACTACCACGATTCAAATGTGAAGAACTTATATGAAAAGGTAAGAAGCCAGTTAAAAAACAATGCCAAGGAAATTGGAAACGGCTGCTTTGAATTTTACCACAAATGCGATAACACGTGCATGGAAAGTGTCAAAAATGGGACTTATGACTACCCAAAATACTCAGAGGAAGCAAAATTAAACAGAGAAGAAATAGATGGGGTAAAGCTGGAATCAACAAGGATTTACCAGATTTTGGCGATCTATTCAACTGTCGCCAGTTCATTGGTACTGGTAGTCTCCCTGGGGGCAATCAGTTTCTGGATGTGCTCTAATGGGTCTCTACAGTGTAGAATATGTATTTAA

>LC638104.1Influenza A virus H1N101701

ATGAAGGCAATACTAGTAGTTCTGCTATATACATTTGCAACCGCAAATGCAGACACATTATGTATAGGTTATCATGCGAACAATTCAACAGACACTGTAGACACAATACTAGAAAAGAATGTAACAGTAACACACTCTGTTAACCTTCTAGAAGACAAGCATAACGGGAAACTATGCAAACTAAGAGGGGTAGCCCCATTGCATTTGGGTAAATGTAACATTGCTGGCTGGATCCTGGGAAATCCAGAGTGTGAATCACTCTCCACAGCAAGCTCATGGTCCTACATTGTGGAAACATCTAGTTCAGACAATGGAACGTGTTACCCAGGAGATTTCATCGATTATGAGGAGCTAAGAGAGCAATTGAGCTCAGTGTCATCATTTGAAAGGTTTGAGATATTCCCCAAGACAAGTTCATGGCCCAATCATGACTCGAACAAAGGTGTAACGGCAGCATGTCCTCATGCTGGAGCAAAAAGCTTCTACAAAAATTTAATATGGCTAGTTAAAAAAGGAAATTCATACCCAAAGCTCAGCAAATCCTACATTAATGATAAAGGGAAAGAAGTCCTCGTGCTATGGGGCATTCACCATCCATCTACTAGTGCTGACCAACAAAGTCTCTATCAGAATGCAGATGCATATGTTTTTGTGGGGACATCAAGATACAGCAAGAAGTTCAAGCCGGAAATAGCAATAAGACCCAAAGTGAGGGATCAAGAAGGGAGAATGAACTATTACTGGACACTAGTAGAGCCGGGAGACAAAATAACATTCGAAGCAACTGGAAATCTAGTGGTACCGAGATATGCATTCGCAATGGAAAGAAATGCTGGATCTGGTATTATCATTTCAGATACACCAGTCCACGATTGCAATACAACTTGTCAGACACCCAAGGGTGCTATAAACACCAGCCTCCCATTTCAGAATATACATCCGATCACAATTGGAAAATGTCCAAAATATGTAAAAAGCACAAAATTGAGACTGGCCACAGGATTGAGGAATGTCCCGTCTATTCAATCTAGAGGCCTATTTGGGGCCATTGCCGGTTTCATTGAAGGGGGGTGGACAGGGATGGTAGATGGATGGTACGGTTGTCACCATCAAAATGAGCAGGGGTCAGGATATGCAGCCGACCTGAAGAGCACACAGAATGCCATTGACGAGATTACTAACAAGGTAAATTCTGTTATTGAAAAGATGAATACACAGTTCACAGCAGTAGGTAAAGAGTTCAACCACCTGGAAAAAAGAATAGAGAATTTAAATAAAAAGGTTGATGATGGTTTCCTGGACATTTGGACTTACAATGCCGAACTGTTGGTTCTATTGGAAAATGAAAGAACTTTGGACTACCACGATTCAAATGTGAAGAACTTATATGAAAAGGTAAGAAGCCAGTTAAAAAACAATGCCAAGGAAATTGGAAACGGCTGCTTTGAATTTTACCACAAATGCGATAACACGTGCATGGAAAGTGTCAAAAATGGGACTTATGACTACCCAAAATACTCAGAGGAAGCAAAATTAAACAGAGAAGAAATAGATGGGGTAAAGCTGGAATCAACAAGGATTTACCAGATTTTGGCGATCTATTCAACTGTCGCCAGTTCATTGGTACTGGTAGTCTCCCTGGGGGCAATCAGTTTCTGGATGTGCTCTAATGGGTCTCTACAGTGTAGAATATGTATTTAA

>LC638103.1Influenza A virus H1N101701

ATGAAGGCAATACTAGTAGTTCTGCTATATACATTTGCAACCGCAAATGCAGACACATTATGTATAGGTTATCATGCGAACAATTCAACAGACACTGTAGACACAATACTAGAAAAGAATGTAACAGTAACACACTCTGTTAACCTTCTAGAAGACAAGCATAACGGGAAACTATGCAAACTAAGAGGGGTAGCCCCATTGCATTTGGGTAAATGTAACATTGCTGGCTGGATCCTGGGAAATCCAGAGTGTGAATCACTCTCCACAGCAAGCTCATGGTCCTACATTGTGGAAACATCTAGTTCAGACAATGGAACGTGTTACCCAGGAGATTTCATCGATTATGAGGAGCTAAGAGAGCAATTGAGCTCAGTGTCATCATTTGAAAGGTTTGAGATATTCCCCAAGACAAGTTCATGGCCCAATCATGACTCGAACAAAGGTGTAACGGCAGCATGTCCTCATGCTGGAGCAAAAAGCTTCTACAAAAATTTAATATGGCTAGTTAAAAAAGGAAATTCATACCCAAAGCTCAGCAAATCCTACATTAATGATAAAGGGAAAGAAGTCCTCGTGCTATGGGGCATTCACCATCCATCTACTAGTGCTGACCAACAAAGTCTCTATCAGAATGCAGATGCATATGTTTTTGTGGGGACATCAAGATACAGCAAGAAGTTCAAGCCGGAAATAGCAATAAGACTCAAAGTGAGGGATCAAGAAGGGAGAATGAACTATTACTGGACACTAGTAGAGCCGGGAGACAAAATAACATTCGAAGCAACTGGAAATCTAGTGGTACCGAGATATGCATTCGCAATGGAAAGAAATGCTGGATCTGGTATTATCATTTCAGATACACCAGTCCACGATTGCAATACAACTTGTCAGACACCCAAGGGTGCTATAAACACCAGCCTCCCATTTCAGAATATACATCCGATCACAATTGGAAAATGTCCAAAATATGTAAAAAGCACAAAATTGAGACTGGCCACAGGATTGAGGAATGTCCCGTCTATTCAATCTAGAGGCCTATTTGGGGCCATTGCCGGTTTCATTGAAGGGGGGTGGACAGGGATGGTAGATGGATGGTACGGTTATCACCATCAAAATGAGCAGGGGTCAGGATATGCAGCCGACCTGAAGAGCACACAGAATGCCATTGACGAGATTACTAACAAGGTAAATTCTGTTATTGAAAAGATGAATACACAGTTCACAGCAGTAGGTAAAGAGTTCAACCACCTGGAAAAAAGAATAGAGAATTTAAATAAAAAGGTTGATGATGGTTTCCTGGACATTTGGACTTACAATGCCGAACTGTTGGTTCTATTGGAAAATGAAAGAACTTTGGACTACCACGATTCAAATGTGAAGAACTTATATGAAAAGGTAAGAAGCCAGTTAAAAAACAATGCCAAGGAAATTGGAAACGGCTGCTTTGAATTTTACCACAAATGCGATAACACGTGCATGGAAAGTGTCAAAAATGGGACTTATGACTACCCAAAATACTCAGAGGAAGCAAAATTAAACAGAGAAGAAATAGATGGGGTAAAGCTGGAATCAACAAGGATTTACCAGATTTTGGCGATCTATTCAACTGTCGCCAGTTCATTGGTACTGGTAGTCTCCCTGGGGGCAATCAGTTTCTGGATGTGCTCTAATGGGTCTCTACAGTGTAGAATATGTATTTAA

>LC638102.1Influenza A virus H1N101701

ATGAAGGCAATACTAGTAGTTCTGCTATATACATTTGCAACCGCAAATGCAGACACATTATGTATAGGTTATCATGCGAACAATTCAACAGACACTGTAGACACAATACTAGAAAAGAATGTAACAGTAACACACTCTGTTAACCTTCTAGAAGACAAGCATAACGGGAAACTATGCAAACTAAGAGGGGTAGCCCCATTGCATTTGGGTAAATGTAACATTGCTGGCTGGATCCTGGGAAATCCAGAGTGTGAATCACTCTCCACAGCAAGCTCATGGTCCTACATTGTGGAAACATCTAGTTCAGACAATGGAACGTGTTACCCAGGAGATTTCATCGATTATGAGGAGCTAAGAGAGCAATTGAGCTCAGTGTCATCATTTGAAAGGTTTGAGATATTCCCCAAGACAAGTTCATGGCCCAATCATGACTCGAACAAAGGTGTAACGGCAGCATGTCCTCATGCTGGAGCAAAAAGCTTCTACAAAAATTTAATATGGCTAGTTAAAAAAGGAAATTCATACCCAAAGCTCAGCAAATCCTACATTAATGATAAAGGGAAAGAAGTCCTCGTGCTATGGGGCATTCACCATCCATCTACTAGTGCTGACCAACAAAGTCTCTATCAGAATGCAGATGCATATGTTTTTGTGGGGACATCAAGATACAGCAAGAAGTTCAAGCCGGAAATAGCAATAAGACCCAAAGTGAGGGATCAAGAAGGGAGAATGAACTATTACTGGACACTAGTAGAGCCGGGAGACAAAATAACATTCGAAGCAACTGGAAATCTAGTGGTACCGAGATATGCATTCGCAATGGAAAGAAATGCTGGATCTGGTATTATCATTTCAGATACACCAGTCCACGATTGCAATACAACTTGTCAGACACCCAAGGGTGCTATAAACACCAGCCTCCCATTTCAGAATATACATCCGATCACAATTGGAAAATGTCCAAAATATGTAAAAAGCACAAAATTGAGACTGGCCACAGGATTGAGGAATGTCCCGTCTATTCAATCTAGAGGCCTATTTGGGGCCATTGCCGGTTTCATTGAAGGGGGGTGGACAGGGATGGTAGATGGATGGTACGGTTATCACCATCAAAATGAGCAGGGGTCAGGATATGCAGCCGACCTGAAGAGCACACAGAATGCCATTGACGAGATTACTAACAAGGTAAATTCTGTTATTGAAAAGATGAATACACAGTTCACAGCAGTAGGTAAAGAGTTCAACCACCTGGAAAAAAGAATAGAGAATTTAAATAAAAAGGTTGATGATGGTTTCCTGGACATTTGGACTTACAATGCCGAACTGTTGGTTCTATTGGAAAATGAAAGAACTTTGGACTACCACGATTCAAATGTGAAGAACTTATATGAAAAGGTAAGAAGCCAGTTAAAAAACAATGCCAAGGAAATTGGAAACGGCTGCTTTGAATTTTACCACAAATGCGATAACACGTGCATGGAAAGTGTCAAAAATGGGACTTATGACTACCCAAAATACTCAGAGGAAGCAAAATTAAACAGAGAAGAAATAGATGGGGTAAAGCTGGAATCAACAAGGATTTACCAGATTTTGGCGATCTATTCAACTGTCGCCAGTTCATTGGTACTGGTAGTCTCCCTGGGGGCAATCAGTTTCTGGATGTGCTCTAATGGGTCTCTACAGTGTAGAATATGTATTTAA

>LC638101.1Influenza A virus H1N101701

ATGAAGGCAATACTAGTAGTTCTGCTATATACATTTGCAACCGCAAATGCAGACACATTATGTATAGGTTATCATGCGAACAATTCAACAGACACTGTAGACACAATACTAGAAAAGAATGTAACAGTAACACACTCTGTTAACCTTCTAGAAGACAAGCATAACGGGAAACTATGCAAACTAAGAGGGGTAGCCCCATTGCATTTGGGTAAATGTAACATTGCTGGCTGGATCCTGGGAAATCCAGAGTGTGAATCACTCTCCACAGCAAGCTCATGGTCCTACATTGTGGAAACATCTAGTTCAGACAATGGAACGTGTTACCCAGGAGATTTCATCGATTATGAGGAGCTAAGAGAGCAATTGAGCTCAGTGTCATCATTTGAAAGGTTTGAGATATTCCCCAAGACAAGTTCATGGCCCAATCATGACTCGAACAAAGGTGTAACGGCAGCATGTCCTCATGCTGGAGCAAAAAGCTTCTACAAAAATTTAATATGGCTAGTTAAAAAAGGAAATTCATACCCAAAGCTCAGCAAATCCTACATTAATGATAAAGGGAAAGAAGTCCTCGTGCTATGGGGCATTCACCATCCATCTACTAGTGCTGACCAACAAAGTCTCTATCAGAATGCAGATGCATATGTTTTTGTGGGGACATCAAGATACAGCAAGAAGTTCAAGCCGGAAATAGCAATAAGACCCAAAGTGAGGGATCAAGAAGGGAGAATGAACTATTACTGGACACTAGTAGAGCCGGGAGACAAAATAACATTCGAAGCAACTGGAAATCTAGTGGTACCGAGATATGCATTCGCAATGGAAAGAAATGCTGGATCTGGTATTATCATTTCAGATACACCAGTCCACGATTGCAATACAACTTGTCAGACACCCAAGGGTGCTATAAACACCAGCCTCCCATTTCAGAATATACATCCGATCACAATTGGAAAATGTCCAAAATATGTAAAAAGCACAAAATTGAGACTGGCCACAGGATTGAGGAATGTCCCGTCTATTCAATCTAGAGGCCTATTTGGGGCCATTGCCGGTTTCATTGAAGGGGGGTGGACAGGGATGGTAGATGGATGGTACGGTTATCACCATCAAAATGAGCAGGGGTCAGGATATGCAGCCGACCTGAAGAGCACACAGAATGCCATTGACGAGATTACTAACAAGGTAAATTCTGTTATTGAAAAGATGAATACACAGTTCACAGCAGTAGGTAAAGAGTTCAACCACCTGGAAAAAAGAATAGAGAATTTAAATAAAAAGGTTGATGATGGTTTCCTGGACATTTGGACTTACAATGCCGAACTGTTGGTTCTATTGGAAAATGAAAGAACTTTGGACTACCACGATTCAAATGTGAAGAACTTATATGAAAAGGTAAGAAGCCAGTTAAAAAACAATGCCAAGGAAATTGGAAACGGCTGCTTTGAATTTTACCACAAATGCGATAACACGTGCATGGAAAGTGTCAAAAATGGGACTTATGACTACCCAAAATACTCAGAGGAAGCAAAATTAAACAGAGAAGAAATAGATGGGGTAAAGCTGGAATCAACAAGGATTTACCAGATTTTGGCGATCTATTCAACTGTCGCCAGTTCATTGGTACTGGTAGTCTCCCTGGGGGCAATCAGTTTCTGGATGTGCTCTAATGGGTCTCTACAGTGTAGAATATGTATTTAA

>LC638100.1Influenza A virus H1N101701

ATGAAGGCAATACTAGTAGTTCTGCTATATACATTTGCAACCGCAAATGCAGACACATTATGTATAGGTTATCATGCGAACAATTCAACAGACACTGTAGACACAATACTAGAAAAGAATGTAACAGTAACACACTCTGTTAACCTTCTAGAAGACAAGCATAACGGGAAACTATGCAAACTAAGAGGGGTAGCCCCATTGCATTTGGGTAAATGTAACATTGCTGGCTGGATCCTGGGAAATCCAGAGTGTGAATCACTCTCCACAGCAAGCTCATGGTCCTACATTGTGGAAACATCTAGTTCAGACAATGGAACGTGTTACCCAGGAGATTTCATCGATTATGAGGAGCTAAGAGAGCAATTGAGCTCAGTGTCATCATTTGAAAGGTTTGAGATATTCCCCAAGACAAGTTCATGGCCCAATCATGACTCGAACAAAGGTGTAACGGCAGCATGTCCTCATGCTGGAGCAAAAAGCTTCTACAAAAATTTAATATGGCTAGTTAAAAAAGGAAATTCATACCCAAAGCTCAGCAAATCCTACATTAATGATAAAGGGAAAGAAGTCCTCGTGCTATGGGGCATTCACCATCCATCTACTAGTGCTGACCAACAAAGTCTCTATCAGAATGCAGATGCATATGTTTTTGTGGGGACATCAAGATACAGCAAGAAGTTCAAGCCGGAAATAGCAATAAGACCCAAAGTGAGGGATCAAGAAGGGAGAATGAACTATTACTGGACACTAGTAGAGCCGGGAGACAAAATAACATTCGAAGCAACTGGAAATCTAGTGGTACCGAGATATGCATTCGCAATGGAAAGAAATGCTGGATCTGGTATTATCATTTCAGATACACCAGTCCACGATTGCAATACAACTTGTCAGACACCCAAGGGTGCTATAAACACCAGCCTCCCATTTCAGAATATACATCCGATCACAATTGGAAAATGTCCAAAATATGTAAAAAGCACAAAATTGAGACTGGCCACAGGATTGAGGAATGTCCCGTCTATTCAATCTAGAGGCCTATTTGGGGCCATTGCCGGTTTCATTGAAGGGGGGTGGACAGGGATGGTAGATGGATGGTACGGTTATCACCATCAAAATGAGCAGGGGTCAGGATATGCAGCCGACCTGAAGAGCACACAGAATGCCATTGACGAGATTACTAACAAGGTAAATTCTGTTATTGAAAAGATGAATACACAGTTCACAGCAGTAGGTAAAGAGTTCAACCACCTGGAAAAAAGAATAGAGAATTTAAATAAAAAGGTTGATGATGGTTTCCTGGACATTTGGACTTACAATGCCGAACTGTTGGTTCTATTGGAAAATGAAAGAACTTTGGACTACCACAATTCAAATGTGAAGAACTTATATGAAAAGGTAAGAAGCCAGTTAAAAAACAATGCCAAGGAAATTGGAAACGGCTGCTTTGAATTTTACCACAAATGCGATAACACGTGCATGGAAAGTGTCAAAAATGGGACTTATGACTACCCAAAATACTCAGAGGAAGCAAAATTAAACAGAGAAGAAATAGATGGGGTAAAGCTGGAATCAACAAGGATTTACCAGATTTTGGCGATCTATTCAACTGTCGCCAGTTCATTGGTACTGGTAGTCTCCCTGGGGGCAATCAGTTTCTGGATGTGCTCTAATGGGTCTCTACAGTGTAGAATATGTATTTAA

>LC638099.1Influenza A virus H1N101701

ATGAAGGCAATACTAGTAGTTCTGCTATATACATTTGCAACCGCAAATGCAGACACATTATGTATAGGTTATCATGCGAACAATTCAACAGACACTGTAGACACAGTACTAGAAAAGAATGTAACAGTAACACACTCTGTTAACCTTCTAGAAGACAAGCATAACGGGAAACTATGCAAACTAAGAGGAGTAGCCCCATTGCATTTGGGTAAATGTAACATTGCTGGCTGGATCCTGGGAAATCCAGAATGTGAATCACTCTCCACAGCAAGCTCATGGTCCTACATTGTGGAAACATCTAGTTCAGACAATGGAACGTGTTACCCAGGAGATTTCATCGATTATGAGGAGCTAAGAGAGCAATTGAGCTCAGTGTCATCATTTGAAAGGTTTGAGATATTCCCCAAGACAAGTTCATGGCCCAATCATGACTCGAACAAAGGCGTAACGGCAGCATGTCCTCATGCTGGAGCAAAAAGCTTCTACAAAAATTTAATATGGCTAGTTAAAAAAGGAAATTCATACCCAAAGCTCAGCAAATCCTACATTAATGATAAAGGGAAAGAAGTCCTCGTGCTATGGGGCATTCACCATCCATCTACTAGTGCTGACCAACAAAGTCTCTATCAGAATGCAGATGCATATGTTTTTGTGGGGACATCAAGATACAGCAAGAAGTTCAAGCCGGAAATAGCAATAAGACCCAAAGTGAGGGATCAAGAAGGGAGAATGAACTATTACTGGACACTAGTAGAGCCGGGAGACAAAATAACATTCGAAGCAACTGGAAATCTAGTGGTACCGAGATATGCATTCGCAATGGAAAGAAATGCTGGATCTGGTATTATCATTTCAGATACACCAGTCCACGATTGCAATACAACTTGTCAGACACCCAAGGGTGCTATAAACACCAGCCTCCCATTTCAGAATATACATCCGATCACAATTGGAAAATGTCCAAAATATGTAAAAAGCACAAAATTGAGACTGGCCACAGGATTGAGGAATGTCCCGTCTATTCAATCTAGAGGCCTATTTGGGGCCATTGCCGGTTTCATTGAAGGGGGGTGGACAGGGATGGTAGATGGATGGTACGGTTATCACCATCAAAATGAGCAGGGGTCAGGATATGCAGCCGACCTGAAGAGCACACAGAATGCCATTGACGAGATTACTAACAAAGTAAATTCTGTTATTGAAAAGATGAATACACAGTTCACAGCAGTAGGTAAAGAGTTCAACCACCTGGAAAAAAGAATAGAGAATTTAAATAAAAAGGTTGATGATGGTTTCCTGGACATTTGGACTTACAATGCCGAACTGTTGGTTCTATTGGAAAATGAAAGAACTTTGGACTACCACGATTCAAATGTGAAGAACTTATATGAAAAGGTAAGAAGCCAGTTAAAAAACAATGCCAAGGAAATTGGAAACGGCTGCTTTGAATTTTACCACAAATGCGATAACACGTGCATGGAAAGTGTAAAAAATGGGACTTATGACTACCCAAAATACTCAGAGGAAGCAAAATTAAACAGAGAAGAAATAGATGGGGTAAAGCTGGAATCAACAAGGATTTACCAGATTTTGGCGATCTATTCAACTGTCGCCAGTTCATTGGTACTGGTAGTCTCCCTGGGGGCAATCAGTTTCTGGATGTGCTCTAATGGGTCTCTACAGTGTAGAATATGTATTTAA

>LC638098.1Influenza A virus H1N101701

ATGAAGGCAATACTAGTAGTTCTGCTATATACATTTGCAACCGCAAATGCAGACACATTATGTATAGGTTATCATGCGAACAATTCAACAGACACTGTAGACACAATACTAGAAAAGAATGTAACAGTAACACACTCTGTTAACCTTCTAGAAGACAAGCATAACGGGAAACTATGCAAACTAAGAGGGGTAGCCCCATTGCATTTGGGTAAATGTAACATTGCTGGCTGGATCCTGGGAAATCCAGAGTGTGAATCACTCTCCACAGCAAGCTCATGGTCCTACATTGTGGAAACATCTAGTTCAGACAATGGAACGTGTTACCCAGGAGATTTCATCGATTATGAGGAGCTAAGAGAGCAATTGAGCTCAGTGTCATCATTTGAAAGGTTTGAGATATTCCCCAAGACAAGTTCATGGCCCAATCATGACTCGAACAAAGGTGTAACGGCAGCATGTCCTCATGCTGGAGCAAAAAGCTTCTACAAAAATTTAATATGGCTAGTTAAAAAAGGAAATTCATACCCAAAGCTCAGCAAATCCTACATTAATGATAAAGGGAAAGAAGTCCTCGTGCTATGGGGCATTCACCATCCATCTACTAGTGCTGACCAACAAAGTCTCTATCAGAATGCAGATGCATATGTTTTTGTGGGGACATCAAGATACAGCAAGAAGTTCAAGCCGGAAATAGCAATAAGACCCAAAGTGAGGGATCAAGAAGGGAGAATGAACTATTACTGGACACTAGTAGAGCCGGGAGACAAAATAACATTCGAAGCAACTGGAAATCTAGTGGTACCGAGATATGCATTCGCAATGGAAAGAAATGCTGGATCTGGTATTATCATTTCAGATACACCAGTCCACGATTGCAATACAACTTGTCAGACACCCAAGGGTGCTATAAACACCAGCCTCCCATTTCAGAATATACATCCGATCACAATTGGAAAATGTCCAAAATATGTAAAAAGCACAAAATTGAGACTGGCCACAGGATTGAGGAATGTCCCGTCTATTCAATCTAGAGGCCTATTTGGGGCCATTGCCGGTTTCATTGAAGGGGGGTGGACAGGGATGGTAGATGGATGGTACGGTTATCACCATCAAAATGAGCAGGGGTCAGGATATGCAGCCGACCTGAAGAGCACACAGAATGCCATTGACGAGATTACTAACAAGGTAAATTCTGTTATTGAAAAGATGAATACACAGTTCACAGCAGTAGGTAAAGAGTTCAACCACCTGGAAAAAAGAATAGAGAATTTAAATAAAAAGGTTGATGATGGTTTCCTGGACATTTGGACTTACAATGCCGAACTGTTGGTTCTATTGGGAAATGAAAGAACTTTGGACTACCACGATTCAAATGTGAAGAACTTATATGAAAAGGTAAGAAGCCAGTTAAAAAACAATGCCAAGGAAATTGGAAACGGCTGCTTTGAATTTTACCACAAATGCGATAACACGTGCATGGAAAGTGTCAAAAATGGGACTTATGACTACCCAAAATACTCAGAGGAAGCAAAATTAAACAGAGAAGAAATAGATGGGGTAAAGCTGGAATCAACAAGGATTTACCAGATTTTGGCGATCTATTCAACTGTCGCCAGTTCATTGGTACTGGTAGTCTCCCTGGGGGCAATCAGTTTCTGGATGTGCTCTAATGGGTCTCTACAGTGTAGAATATGTATTTAA

>LC638097.1Influenza A virus H1N101701

ATGAAGGCAATACTAGTAGTTCTGCTATATACATTTGCAACCGCAAATGCAGACACATTATGTATAGGTTATCATGCGAACAATTCAACAGACACTGTAGACACAATACTAGAAAAGAATGTAACAGTAACACACTCTGTTAACCTTCTAGAAGACAAGCATAACGGGAAACTATGCAAACTAAGAGGGGTAGCCCCATTGCATTTGGGTAAATGTAACATTGCTGGCTGGATCCTGGGAAATCCAGAGTGTGAATCACTCTCCACAGCAAGCTCATGGTCCTACATTGTGGAAACATCTAGTTCAGACAATGGAACGTGTTACCCAGGAGATTTCATCGATTATGAGGAGCTAAGAGAGCAATTGAGCTCAGTGTCATCATTTGAAAGGTTTGAGATATTCCCCAAGACAAGTTCATGGCCCAATCATGACTCGAACAAAGGTGTAACGGCAGCATGTCCTCATGCTGGAGCAAAAAGCTTCTACAAAAATTTAATATGGCTAGTTAAAAAAGGAAATTCATACCCAAAGCTCAGCAAATCCTACATTAATGATAAAGGGAAAGAAGTCCTCGTGCTATGGGGCATTCACCATCCATCTACTAGTGCTGACCAACAAAGTCTCTATCAGAATGCAGATGCATATGTTTTTGTGGGGACATCAAGATACAGCAAGAAGTTCAAGCCGGAAATAGCAATAAGACCCAAAGTGAGGGATCAAGAAGGGAGAATGAACTATTACTGGACACTAGTAGAGCCGGGAGACAAAATAACATTCGAAGCAACTGGAAATCTAGTGGTACCGAGATATGCATTCGCAATGGAAAGAAATGCTGGATCTGGTATTATCATTTCAGATACACCAGTCCACGATTGCAATACAACTTGTCAGACACCCAAGGGTGCTATAAACACCAGCCTCCCATTTCAGAATATACATCCGATCACAATTGGAAAATGTCCAAAATATGTAAAAAGCACAAAATTGAGACTGGCCACAGGATTGAGGAATGTCCCGTCTATTCAATCTAGAGGCCTATTTGGGGCCATTGCCGGTTTCATTGAAGGGGGGTGGACAGGGATGGTAGATGGATGGTACGGTTATCACCATCAAAATGAGCAGGGGTCAGGATATGCAGCCGACCTGAAGAGCACACAGAATGCCATTGACGAGATTACTAACAAGGTAAATTCTGTTATTGAAAAGATGAATACACAGTTCACAGCAGTAGGTAAAGAGTTCAACCACCTGGAAAAAAGAATAGAGAATTTAAATAAAAAGGTTGATGATGGTTTCCTGGACATTTGGACTTACAATGCCGAACTGTTGGTTCTATTGGAAAATGAAAGAACTTTGGACTACCACGATTCAAATGTGAAGAACTTATATGAAAAGGTAAGAAGCCAGTTAAAAAACAATGCCAAGGAAATTGGAAACGGCTGCTTTGAATTTTACCACAAATGCGATAACACGTGCATGGAAAGTGTCAAAAATGGGACTTATGACTACCCAAAATACTCAGAGGAAGCAAAATTAAACAGAGAAGAAATAGATGGGGTAAAGCTGGAATCAACAAGGATTTACCAGATTTTGGCGATCTATTCAACTGTCGCCAGTTCATTGGTACTGGTAGTCTCCCTGGGGGCAATCAGTTTCTGGATGTGCTCTAATGGGTCTCTACAGTGTAGAATATGTATTTAA

>LC638096.1Influenza A virus H1N101701

ATGAAGGCAATACTAGTAGTTCTGCTATATACATTTGCAACCGCAAATGCAGACACATTATGTATAGGTTATCATGCGAACAATTCAACAGACACTGTAGACACAATACTAGAAAAGAATGTAACAGTAACACACTCTGTTAACCTTCTAGAAGACAAGCATAACGGGAAACTATGCAAACTAAGAGGGGTAGCCCCATTGCATTTGGGTAAATGTAACATTGCTGGCTGGATCCTGGGAAATCCAGAGTGTGAATCACTCTCCACAGCAAGCTCATGGTCCTACATTGTGGAAACATCTAGTTCAGACAATGGAACGTGTTACCCAGGAGATTTCATCGATTATGAGGAGCTAAGAGAGCAATTGAGCTCAGTGTCATCATTTGAAAGGTTTGAGATATTCCCCAAGACAAGTTCATGGCCCAATCATGACTCGAACAAAGGTGTAACGGCAGCATGTCCTCATGCTGGAGCAAAAAGCTTCTACAAAAATTTAATATGGCTAGTTAAAAAAGGAAATTCATACCCAAAGCTCAGCAAATCCTACATTAATGATAAAGGGAAAGAAGTCCTCGTGCTATGGGGCATTCACCATCCATCTACTAGTGCTGACCAACAAAGTCTCTATCAGAATGCAGATGCATATGTTTTTGTGGGGACATCAAGATACAGCAAGAAGTTCAAGCCGGAAATAGCAATAAGACCCAAAGTGAGGGATCAAGAAGGGAGAATGAACTATTACTGGACACTAGTAGAGCCGGGAGACAAAATAACATTCGAAGCAACTGGAAATCTAGTGGTACCGAGATATGCATTCGCAATGGAAAGAAATGCTGGATCTGGTATTATCATTTCAGATACACCAGTCCACGATTGCAATACAACTTGTCAGACACCCAAGGGTGCTATAAACACCAGCCTCCCATTTCAGAATATACATCCGATCACAATTGGAAAATGTCCAAAATATGTAAAAAGCACAAAATTGAGACTGGCCACAGGATTGAGGAATGTCCCGTCTATTCAATCTAGAGGCCTATTTGGGGCCATTGCCGGTTTCATTGAAGGGGGGTGGACAGGGATGGTAGATGGATGGTACGGTTATCACCATCAAAATGAGCAGGGGTCAGGATATGCAGCCGACCTGAAGAGCACACAGAATGCCATTGACGAGATTACTAACAAGGTAAATTCTGTTATTGAAAAGATGAATACACAGTTCACAGCAGTAGGTAAAGAGTTCAACCACCTGGAAAAAAGAATAGAGAATTTAAATAAAAAGGTTGATGATGGTTTCCTGGACATTTGGACTTACAATGCCGAACTGTTGGTTCTATTGGAAAATGAAAGAACTTTGGACTACCACGATTCAAATGTGAAGAACTTATATGAAAAGGTAAGAAGCCAGTTAAAAAACAATGCCAAGGAAATTGGAAACGGCTGCTTTGAATTTTACCACAAATGCGATAACACGTGCATGGAAAGTGTCAAAAATGGGACTTATGACTACCCAAAATACTCAGAGGAAGCAAAATTAAACAGAGAAGAAATAGATGGGGTAAAGCTGGAATCAACAAGGATTTACCAGATTTTGGCGATCTATTCAACTGTCGCCAGTTCATTGGTACTGGTAGTCTCCCTGGGGGCAATCAGTTTCTGGATGTGCTCTAATGGGTCTCTACAGTGTAGAATATGTATTTAA

>LC638093.1Influenza A virus H1N101701

ATGAAGGCAATACTAGTAGTTCTGCTATATACATTTGCAACCGCAAATGCAGACACATTATGTATAGGTTATCATGCGAACAATTCAACAGACACTGTAGACACAATACTAGAAAAGAATGTAACAGTAACACACTCTGTTAACCTTCTAGAAGACAAGCATAACGGGAAACTATGCAAACTAAGAGGGGTAGCCCCATTGCATTTGGGTAAATGTAACATTGCTGGCTGGATCCTGGGAAATCCAGAGTGTGAATCACTCTCCACAGCAAGCTCATGGTCCTACATTGTGGAAACATCTAGTTCAGACAATGGAACGTGTTACCCAGGAGATTTCATCGATTATGAGGAGCTAAGAGAGCAATTGAGCTCAGTGTCATCATTTGAAAGGTTTGAGATATTCCCCAAGACAAGTTCATGGCCCAATCATGACTCGAACAAAGGTGTAACGGCAGCATGTCCTCATGCTGGAGCAAAAAGCTTCTACAAAAATTTAATATGGCTAGTTAAAAAAGGAAATTCATACCCAAAGCTCAGCAAATCCTACATTAATGATAAAGGGAAAGAAGTCCTCGTGCTATGGGGCATTCACCATCCATCTACTAGTGCTGACCAACAAAGTCTCTATCAGAATGCAGATGCATATGTTTTTGTGGGGACATCAAGATACAGCAAGAAGTTCAAGCCGGAAATAGCAATAAGACCCAAAGTGAGGGATCAAGAAGGGAGAATGAACTATTACTGGACACTAGTAGAGCCGGGAGACAAAATAACATTCGAAGCAACTGGAAATCTAGTGGTACCGAGATATGCATTCGCAATGGAAAGAAATGCTGGATCTGGTATTATCATTTCAGATACACCAGTCCACGATTGCAATACAACTTGTCAGACACCCAAGGGTGCTATAAACACCAGCCTCCCATTTCAGAATATACATCCGATCACAATTGGAAAATGTCCAAAATATGTAAAAAGCACAAAATTGAGACTGGCCACAGGATTGAGGAATGTCCCGTCTATTCAATCTAGAGGCCTATTTGGGGCCATTGCCGGTTTCATTGAAGGGGGGTGGACAGGGATGGTAGATGGATGGTACGGTTATCACCATCAAAATGAGCAGGGGTCAGGATATGCAGCCGACCTGAAGAGCACACAGAATGCCATTGACGAGATTACTAACAAGGTAAATTCTGTTATTGAAAAGATGAATACACAGTTCACAGCAGTAGGTAAAGAGTTCAACCACCTGGAAAAAAGAATAGAGAATTTAAATAAAAAGGTTGATGATGGTTTCCTGGACATTTGGACTTACAATGCCGAACTGTTGGTTCTATTGGAAAATGAAAGAACTTTGGACTACCACGATTCAAATGTGAAGAACTTATATGAAAAGGTAAGAAGCCAGTTAAAAAACAATGCCAAGGAAATTGGAAACGGCTGCTTTGAATTTTACCACAAATGCGATAACACGTGCATGGAAAGTGTCAAAAATGGGACTTATGACTACCCAAAATACTCAGAGGAAGCAAAATTAAACAGAGAAGAAATAGATGGGGTAAAGCTGGAATCAACAAGGATTTACCAGATTTTGGCGATCTATTCAACTGTCGCCAGTTCATTGGTACTGGTAGTCTCCCTGGGGGCAATCAGTTTCTGGATGTGCTCTAATGGGTCTCTACAGTGTAGAATATGTATTTAA

>LC638091.1Influenza A virus H1N101701

ATGAAGGCAATACTAGTAGTTCTGCTATATACATTTGCAACCGCAAATGCAGACACATTATGTATAGGTTATCATGCGAACAATTCAACAGACACTGTAGACACAATACTAGAAAAGAATGTAACAGTAACACACTCTGTTAACCTTCTAGAAGACAAGCATAACGGGAAACTATGCAAACTAAGAGGGGTAGCCCCATTGCATTTGGGTAAATGTAACATTGCTGGCTGGATCCTGGGAAATCCAGAGTGTGAATCACTCTCCACAGCAAGCTCATGGTCCTACATTGTGGAAACATCTAGTTCAGACAATGGAACGTGTTACCCAGGAGATTTCATCGATTATGAGGAGCTAAGAGAGCAATTGAGCTCAGTGTCATCATTTGAAAGGTTTGAGATATTCCCCAAGACAAGTTCATGGCCCAATCATGACTCGAACAAAGGTGTAACGGCAGCATGTCCTCATGCTGGAGCAAAAAGCTTCTACAAAAATTTAATATGGCTAGTTAAAAAAGGAAATTCATACCCAAAGCTCAGCAAATCCTACATTAATGATAAAGGGAAAGAAGTCCTCGTGCTATGGGGCATTCACCATCCATCTACTAGTGCTGACCAACAAAGTCTCTATCAGAATGCAGATGCATATGTTTTTGTGGGGACATCAAGATACAGCAAGAAGTTCAAGCCGGAAATAGCAATAAGACCCAAAGTGAGGGATCAAGAAGGGAGAATGAACTATTACTGGACACTAGTAGAGCCGGGAGACAAAATAACATTCGAAGCAACTGGAAATCTAGTGGTACCGAGATATGCATTCGCAATGGAAAGAAATGCTGGATCTGGTATTATCATTTCAGATACACCAGTCCACGATTGCAATACAACTTGTCAGACACCCAAGGGTGCTATAAACACCAGCCTCCCATTTCAGAATATACATCCGATCACAATTGGAAAATGTCCAAAATATGTAAAAAGCACAAAATTGAGACTGGCCACAGGATTGAGGAATGTCCCGTCTATTCAATCTAGAGGCCTATTTGGGGCCATTGCCGGTTTCATTGAAGGGGGGTGGACAGGGATGGTAGATGGATGGTACGGTTATCACCATCAAAATGAGCAGGGGTCAGGATATGCAGCCGACCTGAAGAGCACACAGAATGCCATTGACGAGATTACTAACAAGGTAAATTCTGTTATTGAAAAGATGAATACACAGTTCACAGCAGTAGGTAAAGAGTTCAACCACCTGGAAAAAAGAATAGAGAATTTAAATAAAAAGGTTGATGATGGTTTCCTGGACATTTGGACTTACAATGCCGAACTGTTGGTTCTATTGGAAAATGAAAGAACTTTGGACTACCACGATTCAAATGTGAAGAACTTATATGAAAAGGTAAGAAGCCAGTTAAAAAACAATGCCAAGGAAATTGGAAACGGCTGCTTTGAATTTTACCACAAATGCGATAACACGTGCATGGAAAGTGTCAAAAATGGGACTTATGACTACCCAAAATACTCAGAGGAAGCAAAATTAAACAGAGAAGAAATAGATGGGGTAAAGCTGGAATCAACAAGGATTTACCAGATTTTGGCGATCTATTCAACTGTCGCCAGTTCATTGGTACTGGTAGTCTCCCTGGGGGCAATCAGTTTCTGGATGTGCTCTAATGGGTCTCTACAGTGTAGAATATGTATTTAA

>LC638090.1Influenza A virus H1N101701

ATGAAGGCAATACTAGTAGTTCTGCTATATACATTTGCAACCGCAAATGCAGACACATTATGTATAGGTTATCATGCGAACAATTCAACAGACACTGTAGACACAGTACTAGAAAAGAATGTAACAGTAACACACTCTGTTAACCTTCTAGAAGACAAGCATAACGGGAAACTATGCAAACTAAGAGGGGTAGCCCCATTGCATTTGGGTAAATGTAACATTGCTGGCTGGATCCTGGGAAATCCAGAGTGTGAATCACTCTCCACAGCAAGCTCATGGTCCTACATTGTGGAAACATCTAGTTCAGACAATGGAACGTGTTACCCAGGAGATTTCATCGATTATGAGGAGCTAAGAGAGCAATTGAGCTCAGTGTCGTCATTTGAAAGATTTGAGATATTCCCCAAGACAAGTTCATGGCCCAATCATGACTCGAACAAAGGTGTAACGGCAGCATGTCCTCATGCTGGAGCAAAAAGCTTCTACAAAAATTTAATATGGCTAGTTAAAAAAGGAAATTCATACCCAAAGCTCAGCAAATCCTACATTAATGATAAAGGGAAAGAAGTCCTCGTGCTATGGGGCATTCACCATCCATCTACTAGTGCTGACCAACAAAGTCTCTATCAGAATGCAGATGCATATGTTTTTGTGGGGACTTCAAGATACAGCAAGAAGTTCAAGCCGGAAATAGCAATAAGACCCAAAGTGAGGGAACAAGAAGGGAGAATGAACTATTACTGGACACTAGTAGAGCCGGGAGACAAAATAACATTCGAAGCAACTGGAAATCTAGTGGTACCGAGATATGCATTCGCAATGGAAAGAAATGCTGGATCTGGTATTATCATTTCAGATACACCAGTCCACGATTGCAATACAACTTGTCAGACACCCAAGGGTGCTATAAACACCAGCCTCCCATTTCAGAATATACATTCGATCACAATTGGAAAATGTCCAAAATATGTAAAAAGCACAAAATTGAGACTGGCCACAGGATTGAGGAATGTCCCGTCTATTCAATCTAGAGGCCTATTTGGGGCCATTGCCGGTTTCATTGAAGGAGGGTGGACAGGGATGGTAGATGGATGGTACGGTTATCACCATCAAAATGAGCAGGGGTCAGGATATGCAGCCGACCTGAAGAGCACACAGAATGCCATTGACGAGATTACTAACAAAGTAAATTCTGTTATTGAAAAGATGAATACACAGTTCACAGCAGTAGGTAAAGAGTTCAACCACCTGGAAAAAAGAATAGAAAATTTAAATAAAAAAGTTGATGATGGTTTCCTGGACATTTGGACTTACAATGCCGAACTGTTGGTTCTATTGGAAAATGAAAGAACTTTGGACTACCACGATTCAAATGTGAAGAACTTATATGAAAAGGTAAGAAGCCAGTTAAAAAACAATGCCAAGGAAATTGGAAACGGCTGCTTTGAATTTTACCACAAATGCGATAACACGTGCATGGAAAGTGTCAAAAATGGGACTTATGACTACCCAAAATACTCAGAGGAAGCAAAATTAAACAGAGAAGAAATAGATGGGGTAAAGCTGGAATCAACAAGGATTTACCAGATTTTGGCGATCTATTCAACTGTCGCCAGTTCATTGGTACTGGTAGTCTCCCTGGGGGCAATCAGTTTCTGGATGTGCTCTAATGGGTCTCTACAGTGTAGAATATGTATTTAA

>LC638089.1Influenza A virus H1N101701

ATGAAGGCAATACTAGTAGTTCTGCTATATACATTTGCAACCGCAAATGCAGACACATTATGTATAGGTTATCATGCGAACAATTCAACAGACACTGTAGACACAATACTAGAAAAGAATGTAACAGTAACACACTCTGTTAACCTTCTAGAAGACAAGCATAACGGGAAACTATGCAAACTAAGAGGGGTAGCCCCATTGCATTTGGGTAAATGTAACATTGCTGGCTGGATCCTGGGAAATCCAGAGTGTGAATCACTCTCCACAGCAAGCTCATGGTCCTACATTGTGGAAACATCTAGTTCAGACAATGGAACGTGTTACCCAGGAGATTTCATCGATTATGAGGAGCTAAGAGAGCAATTGAGCTCAGTGTCATCATTTGAAAGGTTTGAGATATTCCCCAAGACAAGTTCATGGCCCAATCATGACTCGAACAAAGGTGTAACGGCAGCATGTCCTCATGCTGGAGCAAAAAGCTTCTACAAAAATTTAATATGGCTAGTTAAAAAAGGAAATTCATACCCAAAGCTCAGCAAATCCTACATTAATGATAAAGGGAAAGAAGTCCTCGTGCTATGGGGCATTCACCATCCATCTACTAGTGCTGACCAACAAAGTCTCTACCAGAATGCAGATGCATATGTTTTTGTGGGGACATCAAGATACAGCAAGAAGTTCAAGCCGGAAATAGCAATAAGACCCAAAGTGAGGGATCAAGAAGGGAGAATGAACTATTACTGGACACTAGTAGAGCCGGGAGACAAAATAACATTCGAAGCAACTGGAAATCTAGTGGTACCGAGATATGCATTCGCAATGGAAAGAAATGCTGGATCTGGTATTATCATTTCAGATACACCAGTCCACGATTGCAATACAACTTGTCAGACACCCAAGGGTGCTATAAACACCAGCCTCCCATTTCAGAATATACATCCGATCACAATTGGAAAATGTCCAAAATATGTAAAAAGCACAAAATTGAGACTGGCCACAGGATTGAGGAATGTCCCGTCTATTCAATCTAGAGGCCTATTTGGGGCCATTGCCGGTTTCATTGAAGGGGGGTGGACAGGGATGGTAGATGGATGGTACGGTTATCACCATCAAAATGAGCAGGGGTCAGGATATGCAGCCGACCTGAAGAGCACACAGAATGCCATTGACGAGATTACTAACAAGGTAAATTCTGTTATTGAAAAGATGAATACACAGTTCACAGCAGTAGGTAAAGAGTTCAACCACCTGGAAAAAAGAATAGAGAATTTAAATAAAAAGGTTGATGATGGTTTCCTGGACATTTGGACTTACAATGCCGAACTGTTGGTTCTATTGGAAAATGAAAGAACTTTGGACTACCACGATTCAAATGTGAAGAACTTATATGAAAAGGTAAGAAGCCAGTTAAAAAACAATGCCAAGGAAATTGGAAACGGCTGCTTTGAATTTTACCACAAATGCGATAACACGTGCATGGAAAGTGTCAAAAATGGGACTTATGACTACCCAAAATACTCAGAGGAAGCAAAATTAAACAGAGAAGAAATAGATGGGGTAAAGCTGGAATCAACAAGGATTTACCAGATTTTGGCGATCTATTCAACTGTCGCCAGTTCATTGGTACTGGTAGTCTCCCTGGGGGCAATCAGTTTCTGGATGTGCTCTAATGGGTCTCTACAGTGTAGAATATGTATTTAA

>LC638088.1Influenza A virus H1N101701

ATGAAGGCAATACTAGTAGTTCTGCTATATACATTTGCAACCGCAAATGCAGACACATTATGTATAGGTTATCATGCGAACAATTCAACAGACACTGTAGACACAATACTAGAAAAGAATGTAACAGTAACACACTCTGTTAACCTTCTAGAAGACAAGCATAACGGGAAACTATGCAAACTAAGAGGGGTAGCCCCATTGCATTTGGGTAAATGTAACATTGCTGGCTGGATCCTGGGAAATCCAGAGTGTGAATCACTCTCCACAGCAAGCTCATGGTCCTACATTGTGGAAACATCTAGTTCAGACAATGGAACGTGTTACCCAGGAGATTTCATCGATTATGAGGAGCTAAGAGAGCAATTGAGCTCAGTGTCATCATTTGAAAGGTTTGAGATATTCCCCAAGACAAGTTCATGGCCCAATCATGACTCGAACAAAGGTGTAACGGCAGCATGTCCTCATGCTGGAGCAAAAAGCTTCTACAAAAATTTAATATGGCTAGTTAAAAAAGGAAATTCATACCCAAAGCTCAGCAAATCCTACATTAATGATAAAGGGAAAGAAGTCCTCGTGCTATGGGGCATTCACCATCCATCTACTAGTGCTGACCAACAAAGTCTCTATCAGAATGCAGATGCATATGTTTTTGTGGGGACATCAAGATACAGCAAGAAGTTCAAGCCGGAAATAGCAATAAGACCCAAAGTGAGGGATCAAGAAGGGAGAATGAACTATTACTGGACACTAGTAGAGCCGGGAGACAAAATAACATTCGAAGCAACTGGAAATCTAGTGGTACCGAGATATGCATTCGCAATGGAAAGAAATGCTGGATCTGGTATTATCATTTCAGATACACCAGTCCACGATTGCAATACAACTTGTCAGACACCCAAGGGTGCTATAAACACCAGCCTCCCATTTCAGAATATACATCCGATCACAATTGGAAAATGTCCAAAATATGTAAAAAGCACAAAATTGAGACTGGCCACAGGATTGAGGAATGTCCCGTCTATTCAATCTAGAGGCCTATTTGGGGCCATTGCCGGTTTCATTGAAGGGGGGTGGACAGGGATGGTAGATGGATGGTACGGTTATCACCATCAAAATGAGCAGGGGTCAGGATATGCAGCCGACCTGAAGAGCACACAGAATGCCATTGACGAGATTACTAACAAGGTAAATTCTGTTGTTGAAAAGATGAATACACAGTTCACAGCAGTAGGTAAAGAGTTCAACCACCTGGAAAAAAGAATAGAGAATTTAAATAAAAAGGTTGATGATGGTTTCCTGGACATTTGGACTTACAATGCCGAACTGTTGGTTCTATTGGAAAATGAAAGAACTTTGGACTACCACGATTCAAATGTGAAGAACTTATATGAAAAGGTAAGAAGCCAGTTAAAAAACAATGCCAAGGAAATTGGAAACGGCTGCTTTGAATTTTACCACAAATGCGATAACACGTGCATGGAAAGTGTCAAAAATGGGACTTATGACTACCCAAAATACTCAGAGGAAGCAAAATTAAACAGAGAAGAAATAGATGGGGTAAAGCTGGAATCAACAAGGATTTACCAGATTTTGGCGATCTATTCAACTGTCGCCAGTTCATTGGTACTGGTAGTCTCCCTGGGGGCAATCAGTTTCTGGATGTGCTCTAATGGGTCTCTACAGTGTAGAATATGTATTTAA

>LC638083.1Influenza A virus H1N101701

ATGAAGGCAATACTAGTAGTTCTGCTATATACATTTGCAACCGCAAATGCAGACACATTATGTATAGGTTATCATGCGAACAATTCAACAGACACTGTAGACACAGTACTAGAAAAGAATGTAACAGTAACACACTCTGTTAACCTTCTAGAAGACAAGCATAACGGGAAACTATGCAAACTAAGAGGAGTAGCCCCATTGCATTTGGGTAAATGTAACATTGCTGGCTGGATCCTGGGAAATCCAGAATGTGAATCACTCTCCACAGCAAGCTCATGGTCCTACATTGTGGAAACATCTAGTTCAGACAATGGAACGTGTTACCCAGGAGATTTCATCGATTATGAGGAGCTAAGAGAGCAATTGAGCTCAGTGTCATCATTTGAAAGGTTTGAGATATTCCCCAAGACAAGTTCATGGCCCAATCATGACTCGAACAAAGGCGTAACGGCAGCATGTCCTCATGCTGGAGCAAAAAGCTTCTACAAAAATTTAATATGGCTAGTTAAAAAAGGAAATTCATACCCAAAGCTCAGCAAATCCTACATTAATGATAAAGGGAAAGAAGTCCTCGTGCTATGGGGCATTCACCATCCATCTACTAGTGCTGACCAACAAAGTCTCTATCAGAATGCAGATGCATATGTTTTTGTGGGGACATCAAGATACAGCAAGAAGTTCAAGCCGGAAATAGCAATAAGACCCAAAGTGAGGGATCAAGAAGGGAGAATGAACTATTACTGGACACTAGTAGAGCCGGGAGACAAAATAACATTCGAAGCAACTGGAAATCTAGTGGTACCGAGATATGCATTCGCAATGGAAAGAAATGCTGGATCTGGTATTATCATTTCAGATACACCAGTCCACGATTGCAATACAACTTGTCAGACACCCAAGGGTGCTATAAACACCAGCCTCCCATTTCAGAATATACATCCGATCACAATTGGAAAATGTCCAAAATATGTAAAAAGCACAAAATTGAGACTGGCCACAGGATTGAGGAATGTCCCGTCTATTCAATCTAGAGGCCTATTTGGGGCCATTGCCGGTTTCATTGAAGGGGGGTGGACAGGGATGGTAGATGGATGGTACGGTTATCACCATCAAAATGAGCAGGGGTCAGGATATGCAGCCGACCTGAAGAGCACACAGAATGCCATTGACGAGATTACTAACAAAGTAAATTCTGTTATTGAAAAGATGAATACACAGTTCACAGCAGTAGGTAAAGAGTTCAACCACCTGGAAAAAAGAATAGAGAATTTAAATAAAAAGGTTGATGATGGTTTCCTGGACATTTGGACTTACAATGCCGAACTGTTGGTTCTATTGGAAAATGAAAGAACTTTGGACTACCACGATTCAAATGTGAAGAACTTATATGAAAAGGTAAGAAGCCAGTTAAAAAACAATGCCAAGGAAATTGGAAACGGCTGCTTTGAATTTTACCACAAATGCGATAACACGTGCATGGAAAGTGTAAAAAATGGGACTTATGACTACCCAAAATACTCAGAGGAAGCAAAATTAAACAGAGAAGAAATAGATGGGGTAAAGCTGGAATCAACAAGGATTTACCAGATTTTGGCGATCTATTCAACTGTCGCCAGTTCATTGGTACTGGTAGTCTCCCTGGGGGCAATCAGTTTCTGGATGTGCTCTAATGGGTCTCTACAGTGTAGAATATGTATTTAA

>LC638082.1Influenza A virus H1N101701

ATGAAGGCAATACTAGTAGTTCTGCTATATACATTTGCAACCGCAAATGCAGACACATTATGTATAGGTTATCATGCGAACAATTCAACAGACACTGTAGACACAATACTAGAAAAGAATGTAACAGTAACACACTCTGTTAACCTTCTAGAAGACAAGCATAACGGGAAACTATGCAAACTAAGAGGGGTAGCCCCATTGCATTTGGGTAAATGTAACATTGCTGGCTGGATCCTGGGAAATCCAGAGTGTGAATCACTCTCCACAGCAAGCTCATGGTCCTACATTGTGGAAACATCTAGTTCAGACAATGGAACGTGTTACCCAGGAGATTTCATCGATTATGAGGAGCTAAGAGAGCAATTGAGCTCAGTGTCATCATTTGAAAGGTTTGAGATATTCCCCAAGACAAGTTCATGGCCCAATCATGACTCGAACAAAGGTGTAACGGCAGCATGTCCTCATGCTGGAGCAAAAAGCTTCTACAAAAATTTAATATGGCTAGTTAAAAAAGGAAATTCATACCCAAAGCTCAGCAAATCCTACATTAATGATAAAGGGAAAGAAGTCCTCGTGCTATGGGGCATTCACCATCCATCTACTAGTGCTGACCAACAAAGTCTCTATCAGAATGCAGATGCATATGTTTTTGTGGGGACATCAAGATACAGCAAGAAGTTCAAGCCGGAAATAGCAATAAGACCCAAAGTGAGGGATCAAGAAGGGAGAATGAACTATTACTGGACACTAGTAGAGCCGGGAGACAAAATAACATTCGAAGCAACTGGAAATCTAGTGGTACCGAGATATGCATTCGCAATGGAAAGAAATGCTGGATCTGGTATTATCATTTCAGATACACCAGTCCACGATTGCAATACAACTTGTCAGACACCCAAGGGTGCTATAAACACCAGCCTCCCATTTCAGAATATACATCCGATCACAATTGGAAAATGTCCAAAATATGTAAAAAGCACAAAATTGAGACTGGCCACAGGATTGAGGAATGTCCCGTCTATTCAATCTAGAGGCCTATTTGGGGCCATTGCCGGTTTCATTGAAGGGGGGTGGACAGGGATGGTAGATGGATGGTACGGTTATCACCATCAAAATGAGCAGGGGTCAGGATATGCAGCCGACCTGAAGAGCACACAGAATGCCATTGACGAGATTACTAACAAGGTAAATTCTGTTATTGAAAAGATGAATACACAGTTCACAGCAGTAGGTAAAGAGTTCAACCACCTGGAAAAAAGAATAGAGAATTTAAATAAAAAGGTTGATGATGGTTTCCTGGACATTTGGACTTACAATGCCGAACTGTTGGTTCTATTGGAAAATGAAAGAACTTTGGACTACCACGATTCAAATGTGAAGAACTTATATGAAAAGGTAAGAAGCCAGTTAAAAAACAATGCCAAGGAAATTGGAAACGGCTGCTTTGAATTTTACCACAAATGCGATAACACGTGCATGGAAAGTGTCAAAAATGGGACTTATGACTACCCAAAATACTCAGAGGAAGCAAAATTAAACAGAGAAGAAATAGATGGGGTAAAGCTGGAATCAACAAGGATTTACCAGATTTTGGCGATCTATTCAACTGTCGCCAGTTCATTGGTACTGGTAGTCTCCCTGGGGGCAATCAGTTTCTGGATGTGCTCTAATGGGTCTCTACAGTGTAGAATATGTATTTAA

>LC638080.1Influenza A virus H1N101701

ATGAAGGCAATACTAGTAGTTCTGCTATATACATTTGCAACCGCAAATGCAGACACATTATGTATAGGTTATCATGCGAACAATTCAACAGACACTGTAGACACAGTACTAGAAAAGAATGTAACAGTAACACACTCTGTTAACCTTCTAGAAGACAAGCATAACGGGAAACTATGCAAACTAAGAGGGGTAGCCCCATTGCATTTGGGTAAATGTAACATTGCTGGCTGGATCCTGGGAAATCCAGAGTGTGAATTACTCTCCACAGCAAGCTCATGGTCCTACATTGTGGAAACATCTAGTTCAGACAATGGAACGTGTTACCCAGGAGATTTCATCGATTATGAGGAGCTAAGAGAGCAATTGAGCTCAGTGTCATCATTTGAAAGGTTTGAGATATTCCCCAAGACAAGTTCATGGCCCAATCATGACTCGAACAAAGGTGTAACGGCAGCATGTCCTCATGCTGGAGCAAAAAGCTTCTACAAAAATTTAATATGGCTAGTTAAAAAAGGAAATTCATACCCAAAGCTCAGCAAATCCTACATTAATGATAAAGGGAAAGAAGTCCTCGTGCTATGGGGCATTCACCATCCATCTACTAGTGCTGACCAACAAAGTCTCTATCAGAATGCAGATGCATATGTTTTTGTGGGGACATCAAGATACAGCAAGAAGTTCAAGCCGGAAATAGCAATAAGACCCAAAGTGAGGGATCAAGAAGGGAGAATGAACTATTACTGGACACTAGTAGAGCCGGGAGACAAAATAACATTCGAAGCAACTGGAAATCTAGTGGTACCGAGATATGCATTCGCAATGGAAAGAAATGCTGGATCTGGTATTATCATTTCAGATACACCAGTCCACAATTGCAATACAACTTGTCAGACACCCAAGGGTGCTATAAACACCAGCCTCCCATTTCAGAATATACATCCGATCACAATTGGAAAATGTCCAAAATATGTAAAAAGCACAAAATTGAGACTGGCCACAGGATTGAGGAATGTCCCGTCTATTCAATCTAGAGGCCTATTTGGGGCCATTGCCGGCTTCATTGAAGGGGGGTGGACAGGGATGGTAGATGGATGGTACGGTTATCACCATCAAAATGAGCAGGGGTCAGGATATGCAGCCGACCTGAAGAGCACACAGAATGCCATTGACAAGATTACTAACAAAGTAAATTCTGTTATTGAAAAGATGAATACACAGTTCACAGCAGTAGGTAAAGAGTTCAACCACCTGGAAAAAAGAATAGAGAATTTAAATAAAAAGGTTGATGATGGTTTCCTGGACATTTGGACTTACAATGCCGAACTGTTGGTTCTATTGGAAAATGAAAGAACTTTGGACTACCATGATTCAAATGTGAAGAACTTATATGAAAAGGTAAGAAGCCAGTTAAAAAACAATGCCAAGGAAATTGGAAACGGCTGCTTTGAATTTTACCACAAATGCGATAACACGTGCATGGAAAGTGTCAAAAATGGGACTTATGACTACCCAAAATACTCAGAGGAAGCAAAATTAAACAGAGAAGAAATAGATGGGGTAAAGCTGGAATCAACAAGGATTTACCAGATTTTGGCGATCTATTCAACTGTCGCCAGTTCATTGGTACTGGTAGTCTCCCTGGGGGCAATCAGTTTCTGGATGTGCTCTAATGGGTCTCTACAGTGTAGAATATGTATTTAA

>LC638079.1Influenza A virus H1N101701

ATGAAGGCAATACTAGTAGTTCTGCTATATACATTTGCAACCGCAAATGCAGACACATTATGTATAGGTTATCATGCGAACAATTCAACAGACACTGTAGACACAGTACTAGAAAAGAATGTAACAGTAACACACTCTGTTAACCTTCTAGAAGACAAGCATAACGGGAAACTATGCAAACTAAGAGGGGTAGCCCCATTGCATTTGGGTAAATGTAACATTGCTGGCTGGATCCTGGGAAATCCAGAGTGTGAATTACTCTCCACAGCAAGCTCATGGTCCTACATTGTGGAAACATCTAGTTCAGACAATGGAACGTGTTACCCAGGAGATTTCATCGATTATGAGGAGCTAAGAGAGCAATTGAGCTCAGTGTCATCATTTGAAAGGTTTGAGATATTCCCCAAGACAAGTTCATGGCCCAATCATGACTCGAACAAAGGTGTAACGGCAGCATGTCCTCATGCTGGAGCAAAAAGCTTCTACAAAAATTTAATATGGCTAGTTAAAAAAGGAAATTCATACCCAAAGCTCAGCAAATCCTACATTAATGATAAAGGGAAAGAAGTCCTCGTGCTATGGGGCATTCACCATCCATCTACTAGTGCTGACCAACAAAGTCTCTATCAGAATGCAGATGCATATGTTTTTGTGGGGACATCAAGATACAGCAAGAAGTTCAAGCCGGAAATAGCAATAAGACCCAAAGTGAGGGATCAAGAAGGGAGAATGAACTATTACTGGACACTAGTAGAGCCGGGAGACAAAATAACATTCGAAGCAACTGGAAATCTAGTGGTACCGAGATATGCATTCGCAATGGAAAGAAATGCTGGATCTGGTATTATCATTTCAGATACACCAGTCCACAATTGCAATACAACTTGTCAGACACCCAAGGGTGCTATAAACACCAGCCTCCCATTTCAGAATATACATCCGATCACAATTGGAAAATGTCCAAAATATGTAAAAAGCACAAAATTGAGACTGGCCACAGGATTGAGGAATGTCCCGTCTATTCAATCTAGAGGCCTATTTGGGGCCATTGCCGGCTTCATTGAAGGGGGGTGGACAGGGATGGTAGATGGATGGTACGGTTATCACCATCAAAATGAGCAGGGGTCAGGATATGCAGCCGACCTGAAGAGCACACAGAATGCCATTGACAAGATTACTAACAAAGTAAATTCTGTTATTGAAAAGATGAATACACAGTTCACAGCAGTAGGTAAAGAGTTCAACCACCTGGAAAAAAGAATAGAGAATTTAAATAAAAAGGTTGATGATGGTTTCCTGGACATTTGGACTTACAATGCCGAACTGTTGGTTCTATTGGAAAATGAAAGAACTTTGGACTACCATGATTCAAATGTGAAGAACTTATATGAAAAGGTAAGAAGCCAGTTAAAAAACAATGCCAAGGAAATTGGAAACGGCTGCTTTGAATTTTACCACAAATGCGATAACACGTGCATGGAAAGTGTCAAAAATGGGACTTATGACTACCCAAAATACTCAGAGGAAGCAAAATTAAACAGAGAAGAAATAGATGGGGTAAAGCTGGAATCAACAAGGATTTACCAGATTTTGGCGATCTATTCAACTGTCGCCAGTTCATTGGTACTGGTAGTCTCCCTGGGGGCAATCAGTTTCTGGATGTGCTCTAATGGGTCTCTACAGTGTAGAATATGTATTTAA

>LC638078.1Influenza A virus H1N101701

ATGAAGGCAATACTAGTAGTTCTGCTATATACATTTGCAACCGCAAATGCAGACACATTATGTATAGGTTATCATGCGAACAATTCAACAGACACTGTAGACACAGTACTAGAAAAGAATGTAACAGTAACACACTCTGTTAACCTTCTAGAAGACAAGCATAACGGGAAACTATGCAAACTAAGAGGGGTAGCCCCATTGCATTTGGGTAAATGTAACATTGCTGGCTGGATCCTGGGAAATCCAGAGTGTGAATTACTCTCCACAGCAAGCTCATGGTCCTACATTGTGGAAACATCTAGTTCAGACAATGGAACGTGTTACCCAGGAGATTTCATCGATTATGAGGAGCTAAGAGAGCAATTGAGCTCAGTGTCATCATTTGAAAGGTTTGAGATATTCCCCAAGACAAGTTCATGGCCCAATCATGACTCGAACAAAGGTGTAACGGCAGCATGTCCTCATGCTGGAGCAAAAAGCTTCTACAAAAATTTAATATGGCTAGTTAAAAAAGGAAATTCATACCCAAAGCTCAGCAAATCCTACATTAATGATAAAGGGAAAGAAGTCCTCGTGCTATGGGGCATTCACCATCCATCTACTAGTGCTGACCAACAAAGTCTCTATCAGAATGCAGATGCATATGTTTTTGTGGGGACATCAAGATACAGCAAGAAGTTCAAGCCGGAAATAGCAATAAGACCCAAAGTGAGGGATCAAGAAGGGAGAATGAACTATTACTGGACACTAGTAGAGCCGGGAGACAAAATAACATTCGAAGCAACTGGAAATCTAGTGGTACCGAGATATGCATTCGCAATGGAAAGAAATGCTGGATCTGGTATTATCATTTCAGATACACCAGTCCACAATTGCAATACAACTTGTCAGACACCCAAGGGTGCTATAAACACCAGCCTCCCATTTCAGAATATACATCCGATCACAATTGGAAAATGTCCAAAATATGTAAAAAGCACAAAATTGAGACTGGCCACAGGATTGAGGAATGTCCCGTCTATTCAATCTAGAGGCCTATTTGGGGCCATTGCCGGCTTCATTGAAGGGGGGTGGACAGGGATGGTAGATGGATGGTACGGTTATCACCATCAAAATGAGCAGGGGTCAGGATATGCAGCCGACCTGAAGAGCACACAGAATGCCATTGACAAGATTACTAACAAAGTAAATTCTGTTATTGAAAAGATGAATACACAGTTCACAGCAGTAGGTAAAGAGTTCAACCACCTGGAAAAAAGAATAGAGAATTTAAATAAAAAGGTTGATGATGGTTTCCTGGACATTTGGACTTACAATGCCGAACTGTTGGTTCTATTGGAAAATGAAAGAACTTTGGACTACCATGATTCAAATGTGAAGAACTTATATGAAAAGGTAAGAAGCCAGTTAAAAAACAATGCCAAGGAAATTGGAAACGGCTGCTTTGAATTTTACCACAAATGCGATAACACGTGCATGGAAAGTGTCAAAAATGGGACTTATGACTACCCAAAATACTCAGAGGAAGCAAAATTAAACAGAGAAGAAATAGATGGGGTAAAGCTGGAATCAACAAGGATTTACCAGATTTTGGCGATCTATTCAACTGTCGCCAGTTCATTGGTACTGGTAGTCTCCCTGGGGGCAATCAGTTTCTGGATGTGCTCTAATGGGTCTCTACAGTGTAGAATATGTATTTAA

>LC638077.1Influenza A virus H1N101701

ATGAAGGCAATACTAGTAGTTCTGCTATATACATTTGCAACCGCAAATGCAGACACATTATGTATAGGTTATCATGCGAACAATTCAACAGACACTGTAGACACAATACTAGAAAAGAATGTAACAGTAACACACTCTGTTAACCTTCTAGAAGACAAGCATAACGGGAAACTATGCAAACTAAGAGGGGTAGCCCCATTGCATTTGGGTAAATGTAACATTGCTGGCTGGATCCTGGGAAATCCAGAGTGTGAATCACTCTCCACAGCAAGCTCATGGTCCTACATTGTGGAAACATCTAGTTCAGACAATGGAACGTGTTACCCAGGAGATTTCATCGATTATGAGGAGCTAAGAGAGCAATTGAGCTCAGTGTCATCATTTGAAAGGTTTGAGATATTCCCCAAGACAAGTTCATGGCCCAATCATGACTCGAACAAAGGTGTAACGGCAGCATGTCCTCATGCTGGAGCAAAAAGCTTCTACAAAAATTTAATATGGCTAGTTAAAAAAGGAAATTCATACCCAAAGCTCAGCAAATCCTACATTAATGATAAAGGGAAAGAAGTCCTCGTGCTATGGGGCATTCACCATCCATCTACTAGTGCTGACCAACAAAGTCTCTATCAGAATGCAGATGCATATGTTTTTGTGGGGACATCAAGATACAGCAAGAAGTTCAAGCCGGAAATAGCAATAAGACCCAAAGTGAGGGATCAAGAAGGGAGAATGAACTATTACTGGACACTAGTAGAGCCGGGAGACAAAATAACATTCGAAGCAACTGGAAATCTAGTGGTACCGAGATATGCATTCGCAATGGAAAGAAATGCTGGATCTGGTATTATCATTTCAGATACACCAGTCCACGATTGCAATACAACTTGTCAGACACCCAAGGGTGCTATAAACACCAGCCTCCCATTTCAGAATATACATCCGATCACAATTGGAAAATGTCCAAAATATGTAAAAAGCACAAAATTGAGACTGGCCACAGGATTGAGGAATGTCCCGTCTATTCAATCTAGAGGCCTATTTGGGGCCATTGCCGGTTTCATTGAAGGGGGGTGGACAGGGATGGTAGATGGATGGTACGGTTATCACCATCAAAATGAGCAGGGGTCAGGATATGCAGCCGACCTGAAGAGCACACAGAATGCCATTGACGAGATTACTAACAAGGTAAATTCTGTTATTGAAAAGATGAATACACAGTTCACAGCAGTAGGTAAAGAGTTCAACCACCTGGAAAAAAGAATAGAGAATTTAAATAAAAAGGTTGATGATGGTTTCCTGGACATTTGGACTTACAATGCCGAACTGTTGGTTCTATTGGAAAATGAAAGAACTTTGGACTACCACGATTCAAATGTGAAGAACTTATATGAAAAGGTAAGAAGCCAGTTAAAAAACAATGCCAAGGAAATTGGAAACGGCTGCTTTGAATTTTACCACAAATGCGATAACACGTGCATGGAAAGTGTCAAAAATGGGACTTATGACTACCCAAAATACTCAGAGGAAGCAAAATTAAACAGAGAAGAAATAGATGGGGTAAAGCTGGAATCAACAAGGATTTACCAGATTTTGGCGATCTATTCAACTGTCGCCAGTTCATTGGTACTGGTAGTCTCCCTGGGGGCAATCAGTTTCTGGATGTGCTCTAATGGGTCTCTACAGTGTAGAATATGTATTTAA

>LC638076.1Influenza A virus H1N101701

ATGAAGGCAATACTAGTAGTTCTGCTATATACATTTGCAACCGCAAATGCAGACACATTATGTATAGGTTATCATGCGAACAATTCAACAGACACTGTAGACACAGTACTAGAAAAGAATGTAACAGTAACACACTCTGTTAACCTTCTAGAAAACAAGCATAACGGGAAACTATGCAAACTAAGAGGGGTAGCCCCATTGCATTTGGGTAAATGTAACATTGCTGGCTGGATCCTGGGAAATCCAGAGTGTGAATTACTCTCCACAGCAAGCTCATGGTCCTACATTGTGGAAACATCTAGTTCAGACAATGGAACGTGTTACCCAGGAGATTTCATCGATTATGAGGAGCTAAGAGAGCAATTGAGCTCAGTGTCATCATTTGAAAGGTTTGAGATATTCCCCAAGACAAGTTCATGGCCCAATCATGACTCGAACAAAGGTGTAACGGCAGCATGTCCTCATGCTGGAGCAAAAAGCTTCTACAAAAATTTAATATGGCTAGTTAAAAAAGGAAATTCATACCCAAAGCTCAGCAAATCCTACATTAATGATAAAGGGAAAGAAGTCCTCGTGCTATGGGGCATTCACCATCCATCTACTAGTGCTGACCAACAAAGTCTCTATCAGAATGCAGATGCATATGTTTTTGTGGGGACATCAAGATACAGCAAGAAGTTCAAGCCGGAAATAGCAATAAGACCCAAAGTGAGGGATCAAGAAGGGAGAATGAACTATTACTGGACACTAGTAGAGCCGGGAGACAAAATAACATTCGAAGCAACTGGAAATCTAGTGGTACCGAGATATGCATTCGCAATGGAAAGAAATGCTGGATCTGGTATTATCATTTCAGATACACCAGTCCACAATTGCAATACAACTTGTCAGACACCCAAGGGTGCTATAAACACCAGCCTCCCATTTCAGAATATACATCCGATCACAATTGGAAAATGTCCAAAATATGTAAAAAGCACAAAATTGAGACTGGCCACAGGATTGAGGAATGTCCCGTCTATTCAATCTAGAGGCCTATTTGGGGCCATTGCCGGCTTCATTGAAGGGGGGTGGACAGGGATGGTAGATGGATGGTACGGTTATCACCATCAAAATGAGCAGGGGTCAGGATATGCAGCCGACCTGAAGAGCACACAGAATGCCATTGACAAGATTACTAACAAAGTAAATTCTGTTATTGAAAAGATGAATACACAGTTCACAGCAGTAGGTAAAGAGTTCAACCACCTGGAAAAAAGAATAGAGAATTTAAATAAAAAGGTTGATGATGGTTTCCTGGACATTTGGACTTACAATGCCGAACTGTTGGTTCTATTGGAAAATGAAAGAACTTTGGACTACCATGATTCAAATGTGAAGAACTTATATGAAAAGGTAAGAAGCCAGTTAAAAAACAATGCCAAGGAAATTGGAAACGGCTGCTTTGAATTTTACCACAAATGCGATAACACGTGCATGGAAAGTGTCAAAAATGGGACTTATGACTACCCAAAATACTCAGAGGAAGCAAAATTAAACAGAGAAGAAATAGATGGGGTAAAGCTGGAATCAACAAGGATTTACCAGATTTTGGCGATCTATTCAACTGTCGCCAGTTCATTGGTACTGGTAGTCTCCCTGGGGGCAATCAGTTTCTGGATGTGCTCTAATGGGTCTCTACAGTGTAGAATATGTATTTAA

>LC638075.1Influenza A virus H1N101701

ATGAAGGCAATACTAGTAGTTCTGCTATATACATTTGCAACCGCAAATGCAGACACATTATGTATAGGTTATCATGCGAACAATTCAACAGACACTGTAGACACAGTACTAGAAAAGAATGTAACAGTAACACACTCTGTTAACCTTCTAGAAGACAAGCATAACGGGAAACTATGCAAACTAAGAGGAGTAGCCCCATTGCATTTGGGTAAATGTAACATTGCTGGCTGGATCCTGGGAAATCCAGAATGTGAATCACTCTCCACAGCAAGCTCATGGTCCTACATTGTGGAAACATCTAGTTCAGACAATGGAACGTGTTACCCAGGAGATTTCATCGATTATGAGGAGCTAAGAGAGCAATTGAGCTCAGTGTCATCATTTGAAAGGTTTGAGATATTCCCCAAGACAAGTTCATGGCCCAATCATGACTCGAACAAAGGCGTAACGGCAGCATGTCCTCATGCTGGAGCAAAAAGCTTCTACAAAAATTTAATATGGCTAGTTAAAAAAGGAAATTCATACCCAAAGCTCAGCAAATCCTACATTAATGATAAAGGGAAAGAAGTCCTCGTGCTATGGGGCATTCACCATCCATCTACTAGTGCTGACCAACAAAGTCTCTATCAGAATGCAGATGCATATGTTTTTGTGGGGACATCAAGATACAGCAAGAAGTTCAAGCCGGAAATAGCAATAAGACCCAAAGTGAGGGATCAAGAAGGGAGAATGAACTATTACTGGACACTAGTAGAGCCGGGAGACAAAATAACATTCGAAGCAACTGGAAATCTAGTGGTACCGAGATATGCATTCGCAATGGAAAGAAATGCTGGATCTGGTATTATCATTTCAGATACACCAGTCCACGATTGCAATACAACTTGTCAGACACCCAAGGGTGCTATAAACACCAGCCTCCCATTTCAGAATATACATCCGATCACAATTGGAAAATGTCCAAAATATGTAAAAAGCACAAAATTGAGACTGGCCACAGGATTGAGGAATGTCCCGTCTATTCAATCTAGAGGCCTATTTGGGGCCATTGCCGGTTTCATTGAAGGGGGGTGGACAGGGATGGTAGATGGATGGTACGGTTATCACCATCAAAATGAGCAGGGGTCAGGATATGCAGCCGACCTGAAGAGCACACAGAATGCCATTGACGAGATTACTAACAAAGTAAATTCTGTTATTGAAAAGATGAATACACAGTTCACAGCAGTAGGTAAAGAGTTCAACCACCTGGAAAAAAGAATAGAGAATTTAAATAAAAAGGTTGATGATGGTTTCCTGGACATTTGGACTTACAATGCCGAACTGTTGGTTCTATTGGAAAATGAAAGAACTTTGGACTACCACGATTCAAATGTGAAGAACTTATATGAAAAGGTAAGAAGCCAGTTAAAAAACAATGCCAAGGAAATTGGAAACGGCTGCTTTGAATTTTACCACAAATGCGATAACACGTGCATGGAAAGTGTAAAAAATGGGACTTATGACTACCCAAAATACTCAGAGGAAGCAAAATTAAACAGAGAAGAAATAGATGGGGTAAAGCTGGAATCAACAAGGATTTACCAGATTTTGGCGATCTATTCAACTGTCGCCAGTTCATTGGTACTGGTAGTCTCCCTGGGGGCAATCAGTTTCTGGATGTGCTCTAATGGGTCTCTACAGTGTAGAATATGTATTTAA

>LC638074.1Influenza A virus H1N101701

ATGAAGGCAATACTAGTAGTTCTGCTATATACATTTGCAACCGCAAATGCAGACACATTATGTATAGGTTATCATGCGAACAATTCAACAGACACTGTAGACACAGTACTAGAAAAGAATGTAACAGTAACACACTCTGTTAACCTTCTAGAAGACAAGCATAACGGGAAACTATGCAAACTAAGAGGGGTAGCCCCATTGCATTTGGGTAAATGTAACATTGCTGGCTGGATCCTGGGAAATCCAGAGTGTGAATTACTCTCCACAGCAAGCTCATGGTCCTACATTGTGGAAACATCTAGTTCAGACAATGGAACGTGTTACCCAGGAGATTTCATCGATTATGAGGAGCTAAGAGAGCAATTGAGCTCAGTGTCATCATTTGAAAGGTTTGAGATATTCCCCAAGACAAGTTCATGGCCCAATCATGACTCGAACAAAGGTGTAACGGCAGCATGTCCTCATGCTGGAGCAAAAAGCTTCTACAAAAATTTAATATGGCTAGTTAAAAAAGGAAATTCATACCCAAAGCTCAGCAAATCCTACATTAATGATAAAGGGAAAGAAGTCCTCGTGCTATGGGGCATTCACCATCCATCTACTAGTGCTGACCAACAAAGTCTCTATCAGAATGCAGATGCATATGTTTTTGTGGGGACATCAAGATACAGCAAGAAGTTCAAGCCGGAAATAGCAATAAGACCCAAAGTGAGGGATCAAGAAGGGAGAATGAACTATTACTGGACACTAGTAGAGCCGGGAGACAAAATAACATTCGAAGCAACTGGAAATCTAGTGGTACCGAGATATGCATTCGCAATGGAAAGAAATGCTGGATCTGGTATTATCATTTCAGATACACCAGTCCACAATTGCAATACAACTTGTCAGACACCCAAGGGTGCTATAAACACCAGCCTCCCATTTCAGAATATACATCCGATCACAATTGGAAAATGTCCAAAATATGTAAAAAGCACAAAATTGAGACTGGCCACAGGATTGAGGAATGTCCCGTCTATTCAATCTAGAGGCCTATTTGGGGCCATTGCCGGCTTCATTGAAGGGGGGTGGACAGGGATGGTAGATGGATGGTACGGTTATCACCATCAAAATGAGCAGGGGTCAGGATATGCAGCCGACCTGAAGAGCACACAGAATGCCATTGACAAGATTACTAACAAAGTAAATTCTGTTATTGAAAAGATGAATACACAGTTCACAGCAGTAGGTAAAGAGTTCAACCACCTGGAAAAAAGAATAGAGAATTTAAATAAAAAGGTTGATGATGGTTTCCTGGACATTTGGACTTACAATGCCGAACTGTTGGTTCTATTGGAAAATGAAAGAAATTTGGACTACCATGATTCAAATGTGAAGAACTTATATGAAAAGGTAAGAAGCCAGTTAAAAAACAATGCCAAGGAAATTGGAAACGGCTGCTTTGAATTTTACCACAAATGCGATAACACGTGCATGGAAAGTGTCAAAAATGGGACTTATGACTACCCAAAATACTCAGAGGAAGCAAAATTAAACAGAGAAGAAATAGATGGGGTAAAGCTGGAATCAACAAGGATTTACCAGATTTTGGCGATCTATTCAACTGTCGCCAGTTCATTGGTACTGGTAGTCTCCCTGGGGGCAATCAGTTTCTGGATGTGCTCTAATGGGTCTCTACAGTGTAGAATATGTATTTAA

>LC638073.1Influenza A virus H1N101701

ATGAAGGCAATACTAGTAGTTCTGCTATATACATTTGCAACCGCAAATGCAGACACATTATGTATAGGTTATCATGCGAACAATTCAACAGACACTGTAGACACAGTACTAGAAAAGAATGTAACAGTAACACACTCTGTTAACCTTCTAGAAGACAAGCATAACGGGAAACTATGCAAACTAAGAGGGGTAGCCCCATTGCATTTGGGTAAATGTAACATTGCTGGCTGGATCCTGGGAAATCCAGAGTGTGAATTACTCTCCACAGCAAGCTCATGGTCCTACATTGTGGAAACATCTAGTTCAGACAATGGAACGTGTTACCCAGGAGATTTCATCGATTATGAGGAGCTAAGAGAGCAATTGAGCTCAGTGTCATCATTTGAAAGGTTTGAGATATTCCCCAAGACAAGTTCATGGCCCAATCATGACTCGAACAAAGGTGTAACGGCAGCATGTCCTCATGCTGGAGCAAAAAGCTTCTACAAAAATTTAATATGGCTAGTTAAAAAAGGAAATTCATACCCAAAGCTCAGCAAATCCTACATTAATGATAAAGGGAAAGAAGTCCTCGTGCTATGGGGCATTCACCATCCATCTACTAGTGCTGACCAACAAAGTCTCTATCAGAATGCAGATGCATATGTTTTTGTGGGGACATCAAGATACAGCAAGAAGTTCAAGCCGGAAATAGCAATAAGACCCAAAGTGAGGGATCAAGAAGGGAGAATGAACTATTACTGGACACTAGTAGAGCCGGGAGACAAAATAACATTCGAAGCAACTGGAAATCTAGTGGTACCGAGATATGCATTCGCAATGGAAAGAAATGCTGGATCTGGTATTATCATTTCAGATACACCAGTCCACAATTGCAATACAACTTGTCAGACACCCAAGGGTGCTATAAACACCAGCCTCCCATTTCAGAATATACATCCGATCACAATTGGAAAATGTCCAAAATATGTAAAAAGCACAAAATTGAGACTGGCCACAGGATTGAGGAATGTCCCGTCTATTCAATCTAGAGGCCTATTTGGGGCCATTGCCGGCTTCATTGAAGGGGGGTGGACAGGGATGGTAGATGGATGGTACGGTTATCACCATCAAAATGAGCAGGGGTCAGGATATGCAGCCGACCTGAAGAGCACACAGAATGCCATTGACAAGATTACTAACAAAGTAAATTCTGTTATTGAAAAGATGAATACACAGTTCACAGCAGTAGGTAAAGAGTTCAACCACCTGGAAAAAAGAATAGAGAATTTAAATAAAAAGGTTGATGATGGTTTCCTGGACATTTGGACTTACAATGCCGAACTGTTGGTTCTATTGGAAAATGAAAGAACTTTGGACTACCATGATTCAAATGTGAAGAACTTATATGAAAAGGTAAGAAGCCAGTTAAAAAACAATGCCAAGGAAATTGGAAACGGCTGCTTTGAATTTTACCACAAATGCGATAACACGTGCATGGAAAGTGTCAAAAATGGGACTTATGACTACCCAAAATACTCAGAGGAAGCAAAATTAAACAGAGAAGAAATAGATGGGGTAAAGCTGGAATCAACAAGGATTTACCAGATTTTGGCGATCTATTCAACTGTCGCCAGTTCATTGGTACTGGTAGTCTCCCTGGGGGCAATCAGTTTCTGGATGTGCTCTAATGGGTCTCTACAGTGTAGAATATGTATTTAA

>LC638072.1Influenza A virus H1N101701

ATGAAGGCAATACTAGTAGTTCTGCTATATACATTTGCAACCGCAAATGCAGACACATTATGTATAGGTTATCATGCGAACAATTCAACAGACACTGTAGACACAGTACTAGAAGAGAATGTAACAGTAACACACTCTGTTAACCTTCTAGAAGACAAGCATAACGGGAAACTATGCAAACTAAGAGGGGTAGCCCCATTGCATTTGGGTAAATGTAACATTGCTGGCTGGATCCTGGGAAATCCAGAGTGTGAATTACTCTCCACAGCAAGCTCATGGTCCTACATTGTGGAAACATCTAGTTCAGACAATGGAACGTGTTACCCAGGAGATTTCATCGATTATGAGGAGCTAAGAGAGCAATTGAGCTCAGTGTCATCATTTGAAAGGTTTGAGATATTCCCCAAGACAAGTTCATGGCCCAATCATGACTCGAACAAAGGTGTAACGGCAGCATGTCCTCATGCTGGAGCAAAAAGCTTCTACAAAAATTTAATATGGCTAGTTAAAAAAGGAAATTCATACCCAAAGCTCAGCAAATCCTACATTAATGATAAAGGGAAAGAAGTCCTCGTGCTATGGGGCATTCACCATCCATCTACTAGTGCTGACCAACAAAGTCTCTATCAGAATGCAGATGCATATGTTTTTGTGGGGACATCAAGATACAGCAAGAAGTTCAAGCCGGAAATAGCAATAAGACCCAAAGTGAGGGATCAAGAAGGGAGAATGAACTATTACTGGACACTAGTAGAGCCGGGAGACAAAATAACATTCGAAGCAACTGGAAATCTAGTGGTACCGAGATATGCATTCGCAATGGAAAGAAATGCTGGATCTGGTATTATCATTTCAGATACACCAGTCCACAATTGCAATACAACTTGTCAGACACCCAAGGGTGCTATAAACACCAGCCTCCCATTTCAGAATATACATCCGATCACAATTGGAAAATGTCCAAAATATGTAAAAAGCACAAAATTGAGACTGGCCACAGGATTGAGGAATGTCCCGTCTATTCAATCTAGAGGCCTATTTGGGGCCATTGCCGGCTTCATTGAAGGGGGGTGGACAGGGATGGTAGATGGATGGTACGGTTATCACCATCAAAATGAGCAGGGGTCAGGATATGCAGCCGACCTGAAGAGCACACAGAATGCCATTGACAAGATTACTAACAAAGTAAATTCTGTTATTGAAAAGATGAATACACAGTTCACAGCAGTAGGTAAAGAGTTCAACCACCTGGAAAAAAGAATAGAGAATTTAAATAAAAAGGTTGATGATGGTTTCCTGGACATTTGGACTTACAATGCCGAACTGTTGGTTCTATTGGAAAATGAAAGAACTTTGGACTACCATGATTCAAATGTGAAGAACTTATATGAAAAGGTAAGAAGCCAGTTAAAAAACAATGCCAAGGAAATTGGAAACGGCTGCTTTGAATTTTACCACAAATGCGATAACACGTGCATGGAAAGTGTCAAAAATGGGACTTATGACTACCCAAAATACTCAGAGGAAGCAAAATTAAACAGAGAAGAAATAGATGGGGTAAAGCTGGAATCAACAAGGATTTACCAGATTTTGGCGATCTATTCAACTGTCGCCAGTTCATTGGTACTGGTAGTCTCCCTGGGGGCAATCAGTTTCTGGATGTGCTCTAATGGGTCTCTACAGTGTAAAATATGTATTTAA

>LC638071.1Influenza A virus H1N101701

ATGAAGGCAATACTAGTAGTTCTGCTATATACATTTGCAACCGCAAATGCAGACACATTATGTATAGGTTATCATGCGAACAATTCAACAGACACTGTAGACACAATACTAGAAAAGAATGTAACAGTAACACACTCTGTTAACCTTCTAGAAGACAAGCATAACGGGAAACTATGCAAACTAAGAGGGGTAGCCCCATTGCATTTGGGTAAATGTAACATTGCTGGCTGGATCCTGGGAAATCCAGAGTGTGAATCACTCTCCACAGCAAGCTCATGGTCCTACATTGTGGAAACATCTAGTTCAGACAATGGAACGTGTTACCCAGGAGATTTCATCGATTATGAGGAGCTAAGAGAGCAATTGAGCTCAGTGTCATCATTTGAAAGGTTTGAGATATTCCCCAAGACAAGTTCATGGCCCAATCATGACTCGAACAAAGGTGTAACGGCAGCATGTCCTCATGCTGGAGCAAAAAGCTTCTACAAAAATTTAATATGGCTAGTTAAAAAAGGAAATTCATACCCAAAGCTCAGCAAATCCTACATTAATGATAAAGGGAAAGAAGTCCTCGTGCTATGGGGCATTCACCATCCATCTACTAGTGCTGACCAACAAAGTCTCTATCAGAATGCAGATGCATATGTTTTTGTGGGGACATCAAGATACAGCAAGAAGTTCAAGCCGGAAATAGCAATAAGACCCAAAGTGAGGGATCAAGAAGGGAGAATGAACTATTACTGGACACTAGTAGAGCCGGGAGACAAAATAACATTCGAAGCAACTGGAAATCTAGTGGTACCGAGATATGCATTCGCAATGGAAAGAAATGCTGGATCTGGTATTATCATTTCAGATACACCAGTCCACGATTGCAATACAACTTGTCAGACACCCAAGGGTGCTATAAACACCAGCCTCCCATTTCAGAATATACATCCGATCACAATTGGAAAATGTCCAAAATATGTAAAAAGCACAAAATTGAGACTGGCCACAGGATTGAGGAATGTCCCGTCTATTCAATCTAGAGGCCTATTTGGGGCCATTGCCGGTTTCATTGAAGGGGGGTGGACAGGGATGGTAGATGGATGGTACGGTTATCACCATCAAAATGAGCAGGGGTCAGGATATGCAGCCGACCTGAAGAGCACACAGAATGCCATTGACGAGATTACTAACAAGGTAAATTCTGTTATTGAAAAGATGAATACACAGTTCACAGCAGTAGATAAAGAGTTCAACCACCTGGAAAAAAGAATAGAGAATTTAAATAAAAAGGTTGATGATGGTTTCCTGGACATTTGGACTTACAATGCCGAACTGTTGGTTCTATTGGAAAATGAAAGAACTTTGGACTACCACGATTCAAATGTGAAGAACTTATATGAAAAGGTAAGAAGCCAGTTAAAAAACAATGCCAAGGAAATTGGAAACGGCTGCTTTGAATTTTACCACAAATGCGATAACACGTGCATGGAAAGTGTCAAAAATGGGACTTATGACTACCCAAAATACTCAGAGGAAGCAAAATTAAACAGAGAAGAAATAGATGGGGTAAAGCTGGAATCAACAAGGATTTACCAGATTTTGGCGATCTATTCAACTGTCGCCAGTTCATTGGTACTGGTAGTCTCCCTGGGGGCAATCAGTTTCTGGATGTGCTCTAATGGGTCTCTACAGTGTAGAATATGTATTTAA

>LC638070.1Influenza A virus H1N101701

ATGAAGGCAATACTAGTAGTTCTGCTATATACATTTGCAACCGCAAATGCAGACACATTATGTATAGGTTATCATGCGAACAATTCAACAGACACTGTAGACACAGTACTAGAAAAGAATGTAACAGTAACACACTCTGTTAACCTTCTAGAAGACAAGCATAACGGGAAACTATGCAAACTAAGAGGGGTAGCCCCATTGCATTTGGGTAAATGTAACATTGCTGGCTGGATCCTGGGAAATCCAGAGTGTGAATTACTCTCCACAGCAAGCTCATGGTCCTACATTGTGGAAACATCTAGTTCAGACAATGGAACGTGTTACCCAGGAGATTTCATCGATTATGAGGAGCTAAGAGAGCAATTGAGCTCAGTGTCATCATTTGAAAGGTTTGAGATATTCCCCAAGACAAGTTCATGGCCCAATCATGACTCGAACAAAGGTGTAACGGCAGCATGTCCTCATGCTGGAGCAAAAAGCTTCTACAAAAATTTAATATGGCTAGTTAAAAAAGGAAATTCATACCCAAAGCTCAGCAAATCCTACATTAATGATAAAGGGAAAGAAGTCCTCGTGCTATGGGGCATTCACCATCCATCTACTAGTGCTGACCAACAAAGTCTCTATCAGAATGCAGATGCATATGTTTTTGTGGGGACATCAAGATACAGCAAGAAGTTCAAGCCGGAAATAGCAATAAGACCCAAAGTGAGGGATCAAGAAGGGAGAATGAACTATTACTGGACACTAGTAGAGCCGGGAGACAAAATAACATTCGAAGCAACTGGAAATCTAGTGGTACCGAGATATGCATTCGCAATGGAAAGAAATGCTGGATCTGGTATTATCATTTCAGATACACCAGTCCACAATTGCAATACAACTTGTCAGACACCCAAGGGTGCTATAAACACCAGCCTCCCATTTCAGAATATACATCCGATCACAATTGGAAAATGTCCAAAATATGTAAAAAGCACAAAATTGAGACTGGCCACAGGATTGAGGAATGTCCCGTCTATTCAATCTAGAGGCCTATTTGGGGCCATTGCCGGCTTCATTGAAGGGGGGTGGACAGGGATGGTAGATGGATGGTACGGTTATCACCATCAAAATGAGCAGGGGTCAGGATATGCAGCCGACCTGAAGAGCACACAGAATGCCATTGACAAGATTACTAACAAAGTAAATTCTGTTATTGAAAAGATGAATACACAGTTCACAGCAGTAGGTAAAGAGTTCAACCACCTGGAAAAAAGAATAGAGAATTTAAATAAAAAGGTTGATGATGGTTTCCTGGACATTTGGACTTACAATGCCGAACTGTTGGTTCTATTGGAAAATGAAAGAACTTTGGACTACCATGATTCAAATGTGAAGAACTTATATGAAAAGGTAAGAAGCCAGTTAAAAAACAATGCCAAGGAAATTGGAAACGGCTGCTTTGAATTTTACCACAAATGCGATAACACGTGCATGGAAAGTGTCAAAAATGGGACTTATGACTACCCAAAATACTCAGAGGAAGCAAAATTAAACAGAGAAGAAATAGATGGGGTAAAGCTGGAATCAACAAGGATTTACCAGATTTTGGCGATCTATTCAACTGTCGCCAGTTCATTGGTACTGGTAGTCTCCCTGGGGGCAATCAGTTTCTGGATGTGCTCTAATGGGTCTCTACAGTGTAGAATATGTATTTAA

>LC638069.1Influenza A virus H1N101701

ATGAAGGCAATACTAGTAGTTCTGCTATATACATTTGCAACCGCAAATGCAGACACATTATGTATAGGTTATCATGCGAACAATTCAACAGACACTGTAGACACAGTACTAGAAAAGAATGTAACAGTAACACACTCTGTTAACCTTCTAGAAAACAAGCATAACGGGAAACTATGCAAACTAAGAGGGGTAGCCCCATTGCATTTGGGTAAATGTAACATTGCTGGCTGGATCCTGGGAAATCCAGAGTGTGAATTACTCTCCACAGCAAGCTCATGGTCCTACATTGTGGAAACATCTAGTTCAGACAATGGAACGTGTTACCCAGGAGATTTCATCGATTATGAGGAGCTAAGAGAGCAATTGAGCTCAGTGTCATCATTTGAAAGGTTTGAGATATTCCCCAAGACAAGTTCATGGCCCAATCATGACTCGAACAAAGGTGTAACGGCAGCATGTCCTCATGCTGGAGCAAAAAGCTTCTACAAAAATTTAATATGGCTAGTTAAAAAAGGAAATTCATACCCAAAGCTCAGCAAATCCTACATTAATGATAAAGGGAAAGAAGTCCTCGTGCTATGGGGCATTCACCATCCATCTACTAGTGCTGACCAACAAAGTCTCTATCAGAATGCAGATGCATATGTTTTTGTGGGGACATCAAGATACAGCAAGAAGTTCAAGCCGGAAATAGCAATAAGACCCAAAGTGAGGGATCAAGAAGGGAGAATGAACTATTACTGGACACTAGTAGAGCCGGGAGACAAAATAACATTCGAAGCAACTGGAAATCTAGTGGTACCGAGATATGCATTCGCAATGGAAAGAAATGCTGGATCTGGTATTATCATTTCAGATACACCAGTCCACAATTGCAATACAACTTGTCAGACACCCAAGGGTGCTATAAACACCAGCCTCCCATTTCAGAATATACATCCGATCACAATTGGAAAATGTCCAAAATATGTAAAAAGCACAAAATTGAGACTGGCCACAGGATTGAGGAATGTCCCGTCTATTCAATCTAGAGGCCTATTTGGGGCCATTGCCGGCTTCATTGAAGGGGGGTGGACAGGGATGGTAGATGGATGGTACGGTTATCACCATCAAAATGAGCAGGGGTCAGGATATGCAGCCGACCTGAAGAGCACACAGAATGCCATTGACAAGATTACTAACAAAGTAAATTCTGTTATTGAAAAGATGAATACACAGTTCACAGCAGTAGGTAAAGAGTTCAACCACCTGGAAAAAAGAATAGAGAATTTAAATAAAAAGGTTGATGATGGTTTCCTGGACATTTGGACTTACAATGCCGAACTGTTGGTTCTATTGGAAAATGAAAGAACTTTGGACTACCATGATTCAAATGTGAAGAACTTATATGAAAAGGTAAGAAGCCAGTTAAAAAACAATGCCAAGGAAATTGGAAACGGCTGCTTTGAATTTTACCACAAATGCGATAACACGTGCATGGAAAGTGTCAAAAATGGGACTTATGACTACCCAAAATACTCAGAGGAAGCAAAATTAAACAGAGAAGAAATAGATGGGGTAAAGCTGGAATCAACAAGGATTTACCAGATTTTGGCGATCTATTCAACTGTCGCCAGTTCATTGGTACTGGTAGTCTCCCTGGGGGCAATCAGTTTCTGGATGTGCTCTAATGGGTCTCTACAGTGTAGAATATGTATTTAA

>LC638068.1Influenza A virus H1N101701

ATGAAGGCAATACTAGTAGTTCTGCTATATACATTTGCAACCGCAAATGCAGACACATTATGTATAGGTTATCATGCGAACAATTCAACAGACACTGTAGACACAGTACTAGAAAAGAATGTAACAGTAACACACTCTGTTAACCTTCTAGAAGACAAGCATAACGGGAAACTATGCAAACTAAGAGGGGTAGCCCCATTGCATTTGGGTAAATGTAACATTGCTGGCTGGATCCTGGGAAATCCAGAGTGTGAATTACTCTCCACAGCAAGCTCATGGTCCTACATTGTGGAAACATCTAGTTCAGACAATGGAACGTGTTACCCAGGAGATTTCATCGATTATGAGGAGCTAAGAGAGCAATTGAGCTCAGTGTCATCATTTGAAAGGTTTGAGATATTCCCCAAGACAAGTTCATGGCCCAATCATGACTCGAACAAAGGTGTAACGGCAGCATGTCCTCATGCTGGAGCAAAAAGCTTCTACAAAAATTTAATATGGCTAGTTAAAAAAGGAAATTCATACCCAAAGCTCAGCAAATCCTACATTAATGATAAAGGGAAAGAAGTCCTCGTGCTATGGGGCATTCACCATCCATCTACTAGTGCTGACCAACAAAGTCTCTATCAGAATGCAGATGCATATGTTTTTGTGGGGACATCAAGATACAGCAAGAAGTTCAAGCCGGAAATAGCAATAAGACCCAAAGTGAGGGATCAAGAAGGGAGAATGAACTATTACTGGACACTAGTAGAGCCGGGAGACAAAATAACATTCGAAGCAACTGGAAATCTAGTGGTACCGAGATATGCATTCGCAATGGAAAGAAATGCTGGATCTGGTATTATCATTTCAGATACACCAGTCCACAATTGCAATACAACTTGTCAGACACCCAAGGGTGCTATAAACACCAGCCTCCCATTTCAGAATATACATCCGATCACAATTGGAAAATGTCCAAAATATGTAAAAAGCACAAAATTGAGACTGGCCACAGGATTGAGGAATGTCCCGTCTATTCAATCTAGAGGCCTATTTGGGGCCATTGCCGGCTTCATTGAAGGGGGGTGGACAGGGATGGTAGATGGATGGTACGGTTATCACCATCAAAATGAGCAGGGGTCAGGATATGCAGCCGACCTGAAGAGCACACAGAATGCCATTGACAAGATTACTAACAAAGTAAATTCTGTTATTGAAAAGATGAATACACAGTTCACAGCAGTAGGTAAAGAGTTCAACCACCTGGAAAAAAGAATAGAGAATTTAAATAAAAAGGTTGATGATGGTTTCCTGGACATTTGGACTTACAATGCCGAACTGTTGGTTCTATTGGAAAATGAAAGAACTTTGGACTACCATGATTCAAATGTGAAGAACTTATATGAAAAGGTAAGAAGCCAGTTAAAAAACAATGCCAAGGAAATTGGAAACGGCTGCTTTGAATTTTACCACAAATGCGATAACACGTGCATGGAAAGTGTCAAAAATGGGACTTATGACTACCCAAAATACTCAGAGGAAGCAAAATTAAACAGAGAAGAAATAGATGGGGTAAAGCTGGAATCAACAAGGATTTACCAGATTTTGGCGATCTATTCAACTGTCGCCAGTTCATTGGTACTGGTAGTCTCCCTGGGGGCAATCAGTTTCTGGATGTGCTCTAATGGGTCTCTACAGTGTAGAATATGTATTTAA

>LC638066.1Influenza A virus H1N101701

ATGAAGGCAATACTAGTAGTTCTGCTATATACATTTGCAACCGCAAATGCAGACACATTATGTATAGGTTATCATGCGAACAATTCAACAGACACTGTAGACACAGTACTAGAAAAGAATGTAACAGTAACACACTCTGTTAACCTTCTAGAAGACAAGCATAACGGGAAACTATGCAAACTAAGAGGGGTAGCCCCATTGCATTTGGGTAAATGTAACATTGCTGGCTGGATCCTGGGAAATCCAGAGTGTGAATCACTCTCCACAGCAAGCTCATGGTCCTACATTGTGGAAACATCTAGTTCAGACAATGGAACGTGTTACCCAGGAGATTTCATCGATTATGAGGAGCTAAGAGAGCAATTGAGCTCAGTGTCATCATTTGAAAGGTTTGAGATATTCCCCAAGACAAGTTCATGGCCCAATCATGACTCGAACAAAGGTGTAACGGCAGCATGTCCTCATGCTGGAGCAAAAAGCTTCTACAAAAATTTAATATGGCTAGTTAAAAAAGGAAATTCATACCCAAAGCTCAGCAAATCCTACATTAATGATAAAGGGAAAGAAGTCCTCGTGCTATGGGGCATTCACCATCCATCTACTAGTGCTGACCAACAAAATCTCTATCAGAATGCAGATGCATATGTTTTTGTGGGGACATCAAGATACAGCAAGAAGTTCAAGCCGGAAATAGCAATAAGACCCAAAGTGAGGGATCAAGAAGGGAGAATGAACTATTACTGGACACTAGTAGAGCCGGGAGACAAAATAACATTCGAAGCAACTGGAAATCTAGTGGTACCGAGATATGCATTCGCAATGGAAAGAAATGCTGGATCTGGTATTATCATTTCAGATACACCAGTCCACGATTGCAATACAACTTGTCAAACACCCAAGGGTGCTATAAACACCAGCCTCCCATTTCAGAATATACATCCGATCACAATTGGAAAATGTCCAAAATATGTAAAAAGCACAAAATTGAGACTGGCCACAGGATTGAGGAATGTCCCGTCTATTCAATCTAGAGGCCTATTTGGGGCCATTGCCGGTTTCATTGAAGGGGGGTGGACAGGGATGGTAGATGGATGGTACGGTTATCACCATCAAAATGAGCAGGGGTCAGGATATGCAGCCGACCTGAAGAGCACACAGAATGCCATTGACGAGATTACTAACAAAGTAAATTCTGTTATTGAAAAGATGAATACACAGTTCACAGCAGTAGGTAAAGAGTTCAACCACCTGGAAAAAAGAATAGAGAATTTAAATAAAAAGGTTGATGATGGTTTCCTGGACATTTGGACTTACAATGCAGAACTGTTGGTTCTATTGGAAAATGAAAGAACTTTGGACTACCACGATTCAAATGTGAAGAACTTATATGAAAAGGTAAGAAGCCAGTTAAAAAACAATGCCAAGGAAATTGGAAACGGCTGCTTTGAATTTTACCACAAATGCGATAACACGTGCATGGAAAGTGTCAAAAATGGGACTTATGACTACCCAAAATACTCAGAGGAAGCAAAATTAAACAGAGAAGAAATAGATGGGGTAAAGCTGGAATCAACAAGGATTTACCAGATTTTGGCGATCTATTCAACTGTCGCCAGTTCATTGGTACTGGTAGTCTCCCTGGGGGCAATCAGTTTCTGGATGTGCTCTAATGGGTCTCTACAGTGTAGAATATGTATTTAA

>LC638065.1Influenza A virus H1N101701

ATGAAGGCAATACTAGTAGTTCTGCTATATACATTTGCAACCGCAAATGCAGACACATTATGTATAGGTTATCATGCGAACAATTCAACAGACACTGTAGACACAGTACTAGAAAAGAATGTAACAGTAACACACTCTGTTAACCTTCTAGAAGACAAGCATAACGGGAAACTATGCAAACTAAGAGGGGTAGCCCCATTGCATTTGGGTAAATGTAACATTGCTGGCTGGATCCTGGGAAATCCAGAGTGTGAATCACTCTCCACAGCAAGCTCATGGTCCTACATTGTGGAAACATCTAGTTCAGACAATGGAACGTGTTACCCAGGAGATTTCATCGATTATGAGGAGCTAAGAGAGCAATTGAGCTCAGTGTCATCATTTGAAAGGTTTGAGATATTCCCCAAGACAAGTTCATGGCCCAATCATGACTCGAACAAAGGTGTAACGGCAGCATGTCCTCATGCTGGAGCAAAAAGCTTCTACAAAAATTTAATATGGCTAGTTAAAAAAGGAAATTCATACCCAAAGCTCAGCAAATCCTACATTAATGATAAAGGGAAAGAAGTCCTCGTGCTATGGGGCATTCACCATCCATCTACTAGTGCTGACCAACAAAATCTCTATCAGAATGCAGATGCATATGTTTTTGTGGGGACATCAAGATACAGCAAGAAGTTCAAGCCGGAAATAGCAATAAGACCCAAAGTGAGGGATCAAGAAGGGAGAATGAACTATTACTGGACACTAGTAGAGCCGGGAGACAAAATAACATTCGAAGCAACTGGAAATCTAGTGGTACCGAGATATGCATTCGCAATGGAAAGAAATGCTGGATCTGGTATTATCATTTCAGATACACCAGTCCACGATTGCAATACAACTTGTCAAACACCCAAGGGTGCTATAAACACCAGCCTCCCATTTCAGAATATACATCCGATCACAATTGGAAAATGTCCAAAATATGTAAAAAGCACAAAATTGAGACTGGCCACAGGATTGAGGAATGTCCCGTCTATTCAATCTAGAGGCCTATTTGGGGCCATTGCCGGTTTCATTGAAGGGGGGTGGACAGGGATGGTAGATGGATGGTACGGTTATCACCATCAAAATGAGCAGGGGTCAGGATATGCAGCCGACCTGAAGAGCACACAGAATGCCATTGACGAGATTACTAACAAAGTAAATTCTGTTATTGAAAAGATGAATACACAGTTCACAGCAGTAGGTAAAGAGTTCAACCACCTGGAAAAAAGAATAGAGAATTTAAATAAAAAGGTTGATGATGGTTTCCTGGACATTTGGACTTACAATGCAGAACTGTTGGTTCTATTGGAAAATGAAAGAACTTTGGACTACCACGATTCAAATGTGAAGAACTTATATGAAAAGGTAAGAAGCCAGTTAAAAAACAATGCCAAGGAAATTGGAAACGGCTGCTTTGAATTTTACCACAAATGCGATAACACGTGCATGGAAAGTGTCAAAAATGGGACTTATGACTACCCAAAATACTCAGAGGAAGCAAAATTAAACAGAGAAGAAATAGATGGGGTAAAGCTGGAATCAACAAGGATTTACCAGATTTTGGCGATCTATTCAACTGTCGCCAGTTCATTGGTACTGGTAGTCTCCCTGGGGGCAATCAGTTTCTGGATGTGCTCTAATGGGTCTCTACAGTGTAGAATATGTATTTAA

>LC638063.1Influenza A virus H1N101701

ATGAAGGCAATACTAGTAGTTCTGCTATATACATTTGCAACCGCAAATGCAGACACATTATGTATAGGTTATCATGCGAACAATTCAACAGACACTGTAGACACAGTACTAGAAAAGAATGTAACAGTAACACACTCTGTTAACCTTCTAGAAGACAAGCATAACGGGAAACTATGCAAACTAAGAGGGGTAGCCCCATTGCATTTGGGTAAATGTAACATTGCTGGCTGGATCCTGGGAAATCCAGAGTGTGAATCACTCTCCACAGCAAGCTCATGGTCCTACATTGTGGAAACATCTAGTTCAGACAATGGAACGTGTTACCCAGGAGATTTCATCGATTATGAGGAGCTAAGAGAGCAATTGAGCTCAGTGTCATCATTTGAAAGGTTTGAGATATTCCCCAAGACAAGTTCATGGCCCAATCATGACTCGAACAAAGGTGTAACGGCAGCATGTCCTCATGCTGGAGCAAAAAGCTTCTACAAAAATTTAATATGGCTAGTTAAAAAAGGAAATTCATACCCAAAGCTCAGCAAATCCTACATTAATGATAAAGGGAAAGAAGTCCTCGTGCTATGGGGCATTCACCATCCATCTACTAGTGCTGACCAACAAAGTCTCTATCAGAATGCAGATGCATATGTTTTTGTGGGGACATCAAGATACAGCAAGAAGTTCAAGCCGGAAATAGCAATAAGACCCAAAGTGAGGGATCAAGAAGGGAGAATGAACTATTACTGGACACTAGTAGAGCCGGGAGACAAAATAACATTCGAAGCAACTGGAAATCTAGTGGTACCGAGATATGCATTCGCAATGGAAAGAAATGCTGGATCTGGTATTATCATTTCAGATACACCAGTCCACGATTGCAATACAACTTGTCAGACACCCAAGGGTGCTATAAACACCAGCCTCCCATTTCAGAATATACATCCGATCACAATTGGAAAATGTCCAAAATATGTAAAAAGCACAAAATTGAGACTGGCCACAGGATTGAGGAATGTCCCGTCTATTCAATCTAGAGGCCTATTTGGGGCCATTGCCGGTTTCATTGAAGGGGGGTGGACAGGGATGGTAGATGGATGGTACGGTTATCACCATCAAAATGAGCAGGGGTCAGGATATGCAGCCGACCTGAAGAGCACACAGAATGCCATTGACGAGATTACTAACAAAGTAAATTCTGTTATTGAAAAGATGAATACACAGTTCACAGCAGTAGGTAAAGAGTTCAACCACCTGGAAAAAAGAATAGAGAATTTAAATAAAAAGGTTGATGATGGTTTCCTGGACATTTGGACTTACAATGCAGAACTGTTGGTTCTATTGGAAAATGAAAGAACTTTGGACTACCACGATTCAAATGTGAAGAACTTATATGAAAAGGTAAGAAGCCAGTTAAAAAACAATGCCAAGGAAATTGGAAACGGCTGCTTTGAATTTTACCACAAATGCGATAACACGTGCATGGAAAGTGTCAAAAATGGGACTTATGACTACCCAAAATACTCAGAGGAAGCAAAATTAAACAGAGAAGAAATAGATGGGGTAAAGCTGGAATCAACAAGGATTTACCAGATTTTGGCGATCTATTCAACTGTCGCCAGTTCATTGGTACTGGTAGTCTCCCTGGGGGCAATCAGTTTCTGGATGTGCTCTAATGGGTCTCTACAGTGTAGAATATGTATTTAA

>LC638062.1Influenza A virus H1N101701

ATGAAGGCAATACTAGTAGTTCTGCTATATACATTTGCAACCGCAAATGCAGACACATTATGTATAGGTTATCATGCGAACAATTCAACAGACACTGTAGACACAGTACTAGAAAAGAATGTAACAGTAACACACTCTGTTAACCTTCTAGAAGACAAGCATAACGGGAAACTATGCAAACTAAGAGGGGTAGCCCCATTGCATTTGGGTAAATGTAACATTGCTGGCTGGATCCTGGGAAATCCAGAGTGTGAATCACTCTCCACAGCAAGCTCATGGTCCTACATTGTGGAAACATCTAGTTCAGACAATGGAACGTGTTACCCAGGAGATTTCATCGATTATGAGGAGCTAAGAGAGCAATTGAGCTCAGTGTCATCATTTGAAAGGTTTGAGATATTCCCCAAGACAAGTTCATGGCCCAATCATGACTCGAACAAAGGTGTAACGGCAGCATGTCCTCATGCTGGAGCAAAAAGCTTCTACAAAAATTTAATATGGCTAGTTAAAAAAGGAAATTCATACCCAAAGCTCAGCAAATCCTACATTAATGATAAAGGGAAAGAAGTCCTCGTGCTATGGGGCATTCACCATCCATCTACTAGTGCTGACCAACAAAGTCTCTATCAGAATGCAGATGCATATGTTTTTGTGGGGACATCAAGATACAGCAAGAAGTTCAAGCCGGAAATAGCAATAAGACCCAAAGTGAGGGATCAAGAAGGGAGAATGAACTATTACTGGACACTAGTAGAGCCGGGAGACAAAATAACATTCGAAGCAACTGGAAATCTAGTGGTACCGAGATATGCATTCGCAATGGAAAGAAATGCTGGATCTGGTATTATCATTTCAGATACACCAGTCCACGATTGCAATACAACTTGTCAGACACCCAAGGGTGCGATAAACACCAGCCTACCATTTCAGAATATACATCCGATCACAATTGGAAAATGTCCAAAATATGTAAAAAGCACAAAATTGAGACTGGCCACAGGATTGAGGAATGTCCCGTCTATTCAATCTAGAGGCCTATTTGGGGCCATTGCCGGTTTCATTGAAGGGGGGTGGACAGGGATGGTAGATGGATGGTACGGTTATCACCATCAAAATGAGCAGGGGTCAGGATATGCAGCCGACCTGAAGAGCACACAGAATGCCATTGACGAGATTACTAACAAAGTAAATTCTGTTATTGAAAAGATGAATACACAGTTCACAGCAGTAGGTAAAGAGTTCAACCACCTGGAAAAAAGAATAGAGAATTTAAATAAAAAAGTTGATGATGGTTTCCTGGACATTTGGACTTACAATGCCGAACTGTTGGTTCTATTGGAAAATGAAAGAACTTTGGACTACCACGATTCAAATGTGAAGAACTTATATGAAAAGGTAAGAAGCCAGTTAAAAAACAATGCCAAGGAAATTGGAAACGGCTGCTTTGAATTTTACCACAAATGTGATAACACGTGCATGGAAAGTGTCAAAAATGGGACTTATGACTACCCAAAATACTCAGAGGAAGCAAAATTAAACAGAGAAGAAATAGATGGGGTAAAGCTGGAATCAACAAGGATTTACCAGATTTTGGCGATCTATTCAACTGTCGCCAGTTCATTGGTACTGGTAGTCTCCCTGGGGGCAATCAGTTTCTGGATGTGCTCTAATGGGTCTCTACAGTGTAGAATATGTATTTAA

>LC778491.1Influenza A virus H1N101701

ATGAAGGCAATACTAGTAGTCCTGCTATACACATATGCAACCGCAAATGCAGACACATTATGTATAGGTTATCATGCGAACAATTCAACAGACACAGTAGACACAATACTAGAAAAAAATGTAACAGTAACACACTCTGTTAACCTTCTAGAAGACAAGCATAACGGGAAACTATGCAAACTAAGAGGGGTAGCCCCATTGCATTTGGGCAAATGCAACATTGCTGGCTGGATCCTGGGAAATCCAGAGTGTGAATCACTCTACACGGCAAGCTCATGGTCCTACATTGTGGAAAAGTCTAGTCCAGACAATGGAACGTGTTACCCAGGAGAGTTCATCGATTATGAGGAACTAAGAGAGCAATTAAGCTCAGTGTCATCATTTGAAAGGTTTGAGATATTTCCCAATACAAGTTCATGGCCCAATCATGACTCGAACAAAGGTGTAACGGCAGCATGTCCTCATGATGGAGCAAAAGGCTTCTACAAAAATTTAATATGGCTGGTTAAAAAAGAAAATTCATACCCAAAGCTCAACATCTCTTACATTAATAATAAAGGAAAAGAAGTCCTTGTGCTATGGGGCATTCACCATCCAACTACTACTGCTAGCCAAGAAACTCTCTATCAGAATGCAGATGCATATGTTTTTGTGGGGACATCAAAATATAGCAAGAAGTTCAAGCCGGAGATAGCAATAAGACCCAAAGTGAGAGATCAAGAAGGGAGAATGAACTATTACTGGACATTAGTAGAGCCGGGAGACAAAATAACATTTGAAGCAACTGGAAATCTAGTGGTACCGAGATATGCATTCGCAATGGAGAGAAATGCTGGTTCTGGTATTATCATTTCAGATACACCAGTCCACAATTGTTATACAACTTGTCAGACACCCAAGGGTGCTGTAAACACCAGCCTCCCATTTCAGAACATACATCCGATCACAATAGGAAAATGTCCAAAATATGTAAAAAGCACAAAACTGAGACTGGCCACAGGATTGAGGAATACACCGTCTATCCAATCTAGAGGCCTATTTGGGGCCATTGCCGGTTTTATTGAAGGGGGGTGGACAGGGATGGTAGATGGATGGTACGGTTATCATCATCAAAATGAACAGGGGTCAGGATATGCAGCCGACCTGAAGAGCACACAGAATGCCATTGACGAGATTACTAACAAAGTAAATTCTGTTATTGAAAAGATGAATACACAATTCACAGCAATAGGTAAAGAGTTCAACCATTTGGAAAAAAGAATAGAGAATTTAAATAAAAAGGTTGATGATGGTTTCCTGGACATTTGGACTTACAATGCCGAACTGTTGGTTCTATTGGAAAATGAAAGAACTTTGGACTACCACGATTCAAATGTAAAGAACTTATATGAAAAGGTAAGAAGCCAGTTAAAAAACAATGCCAAGGAAATTGGAAACGGTTGCTTTGAATTTTATCACAAATGTGATAACACGTGCATTGAAAGTGTCAAAAATGGGACTTACGACTACCCCAAATACTCAGAGGAAGCAAAATTAAACAGAGAAGAAATAGATGGGGTAAAGCTGGAACCAACAAGGATTCACCAGATTTTGGCGATCTACTCAACTGTTGCCAGTTCATTAGTACTGGTAGTCTCCCTGGCGGCAATCAGTTTCTGGATGTGCTCTAATGGGTCTCTACAGTGTAGAATATGTATTTAA

>LC778483.1Influenza A virus H1N101701

ATGAAGGCAATACTAGTAGTCCTGCTATACACATATGCAACCGCAAATGCAGACACATTATGTATAGGTTATCATGCGAACAATTCAACAGACACAGTAGACACAATACTAGAAAAAAATGTAACAGTAACACACTCTGTTAACCTTCTAGAAGACAAGCATAACGGGAAACTATGCAAACTAAGAGGGGTAGCCCCATTGCATTTGGGCAAATGCAACATTGCTGGCTGGATCCTGGGAAATCCAGAGTGTGAATCACTCTACACGGCAAGCTCATGGTCCTACATTGTGGAAAAGTCTAGTCCAGACAATGGAACGTGTTACCCAGGAGAGTTCATCGATTATGAGGAACTAAGAGAGCAATTAAGCTCAGTGTCATCATTTGAAAGGTTTGAGATATTTCCCAATACAAGTTCATGGCCCAATCATGACTCGAACAAAGGTGTAACGGCAGCATGTCCTCATGATGGAGCAAAAGGCTTCTACAAAAATTTAATATGGCTGGTTAAAAAAGAAAATTCATACCCAAAGCTCAACATCTCTTACATTAATAATAAAGGAAAAGAAGTCCTTGTGCTATGGGGCATTCACCATCCAACTACTACTGCTAGCCAAGAAACTCTCTATCAGAATGCAGATGCATATGTTTTTGTGGGGACATCAAAATATAGCAAGAAGTTCAAGCCGGAGATAGCAATAAGACCCAAAGTGAGAGATCAAGAAGGGAGAATGAACTATTACTGGACATTAGTAGAGCCGGGAGACAAAATAACATTTGAAGCAACTGGAAATCTAGTGGTACCGAGATATGCATTCGCAATGGAAAGAAATGCTGGTTCTGGTATTATCATTTCAGATACACCAGTCCACAATTGTTATACAACTTGTCAGACACCCAAGGGTGCTGTAAACACCAGCCTCCCATTTCAGAACATACATCCGATCACAATAGGAAAATGTCCAAAATATGTAAAAAGCACAAAACTGAGACTGGCCACAGGATTGAGGAATACACCGTCTATCCAATCTAGAGGCCTATTTGGGGCCATTGCCGGTTTTATTGAAGGGGGGTGGACAGGGATGGTAGATGGATGGTACGGTTATCATCATCAAAATGAACAGGGGTCAGGATATGCAGCCGACCTGAAGAGCACACAGAATGCCATTGACGAGATTACTAACAAAGTAAATTCTGTTATTGAAAAGATGAATACACAATTCACAGCAATAGGTAAAGAGTTCAACCATTTGGAAAAAAGAATAGAGAATTTAAATAAAAAGGTTGATGATGGTTTCCTGGACATTTGGACTTACAATGCCGAACTGTTGGTTCTATTGGAAAATGAAAGAACTTTGGACTACCACGATTCAAATGTAAAGAACTTATATGAAAAGGTAAGAAGCCAGTTAAAAAACAATGCCAAGGAAATTGGAAACGGTTGCTTTGAATTTTATCACAAATGTGATAACACGTGCATTGAAAGTGTCAAAAATGGGACTTACGACTACCCCAAATACTCAGAGGAAGCAAAATTAAACAGAGAAGAAATAGATGGGGTAAAGCTGGAACCAACAAGGATTCACCAGATTTTGGCGATCTACTCAACTGTTGCCAGTTCATTAGTACTGGTAGTCTCCCTGGCGGCAATCAGTTTCTGGATGTGCTCTAATGGGTCTCTACAGTGTAGAATATGTATTTAA

>LC778475.1Influenza A virus H1N101701

ATGAAGGCAATACTAGTAGTCCTGCTATACACATATGCAACCGCAAATGCAGACACATTATGTATAGGTTATCATGCGAACAATTCAACAGACACAGTAGACACAATACTAGAAAAAAATGTAACAGTAACACACTCTGTTAACCTTCTAGAAGACAAGCATAACGGGAAACTATGCAAACTAAGAGGGGTAGCCCCATTGCATTTGGGCAAATGCAACATTGCTGGCTGGATCCTGGGAAATCCAGAGTGTGAATCACTCTACACGGCAAGCTCATGGTCCTACATTGTGGAAACGTCTAGTCCAGACAATGGAACGTGTTACCCAGGAGAGTTCATCGATTATGAGGAACTAAGAGAGCAATTAAGCTCAGTGTCATCATTTGAAAGGTTTGAGATATTTCCCAATACAAGTTCATGGCCCAATCATGACTCGAACAAAGGTGTAACGGCAGCATGTCCTCATGATGGAGCAAAAGGCTTCTACAAAAATTTAATATGGCTGGTTAAAAAAGAAAATTCATACCCAAAGCTCAACATCTCTTACATTAATAATAAAGGAAAAGAAGTCCTTGTGCTATGGGGCATTCACCATCCAACTACTACTGCTAGCCAAGAAACTCTCTATCAGAATGCAGATGCATATGTTTTTGTGGGGACATCAAAATATAGCAAGAAGTTCAAGCCGGAGATAGCAATAAGACCCAAAGTGAGAGATCAAGAAGGGAGAATGAACTATTACTGGACATTAGTAGAGCCGGGAGACAAAATAACATTTGAAGCAACTGGAAATCTAGTGGTACCGAGATATGCATTCGCAATGGAAAGAAATGCTGGTTCTGGTATTATCATTTCAGATACACCAGTCCACAATTGTTATACAACTTGTCAGACACCCAAGGGTGCTGTAAACACCAGCCTCCCATTTCAGAACATACATCCGATCACAATAGGAAAATGTCCAAAATATGTAAAAAGCACAAAACTGAGACTGGCCACAGGATTGAGGAATACACCGTCTATTCAATCTAGAGGCCTATTTGGGGCCATTGCCGGTTTTATTGAAGGGGGGTGGACAGGGATGGTAGATGGATGGTACGGTTATCATCATCAAAATGAACAGGGGTCAGGATATGCAGCCGACCTGAAGAGCACACAGAATGCCATTGACGAGATTACTAACAAAGTAAATTCTGTTATTGAAAAGATGAATACACAATTCACAGCAATAGGTAAAGAGTTCAACCATTTGGAAAAAAGAATAGAGAATTTAAATAAAAAGGTTGATGATGGTTTCCTGGACATTTGGACTTACAATGCCGAACTGTTGGTTCTATTGGAAAATGAAAGAACTTTGGACTACCACGATTCAAATGTAAAGAACTTATATGAAAAGGTAAGAAGCCAGTTAAAAAACAATGCCAAGGAAATTGGAAACGGTTGCTTTGAATTTTATCACAAATGTGATAACACGTGCATTGAAAGTGTCAAAAATGGGACTTACGACTACCCCAAATACTCAGAGGAAGCAAAATTAAACAGAGAAGAAATAGATGGGGTAAAGCTGGAACCAACAAGGATTCACCAGATTTTGGCGATCTACTCAACTGTTGCCAGTTCATTAGTACTGGTAGTCTCCCTGGCGGCAATCAGTTTCTGGATGTGCTCTAATGGGTCTCTACAGTGTAGAATATGTATTTAA

>LC778467.1Influenza A virus H1N101701

ATGAAGGCTATACTAGTAGTCCTGCTATACAGATTTGCAACCGCAAATGCAGACAAATTATGCATAGGTTATCATGCGAACAATTCAACAGACACTGTAGACACAGTACTAGAAAAGAATGTAACAGTAACACATTCTGTCAACCTTCTAGAAGACAAGCATAACGAAAAACTATGCAAACTAAAAGGGGTAGCCCCATTGCATTTGGGCAAATGTAACATTGCTGGCTGGATACTGGGAAATCCAGAGTGTGAATCACTCTCCACAGCAAGCTCATGGTCCTACATTGTGGAGACATCTAGTTCAGACAATGGAACGTGTTACCCAGGAGATTTCATCGATTATGAGGAGCTAAGAGAGCAGCTGAGTTCAGTGTCATCATTTGAAAAGTTTGAGATATTCCCCAAGACAAGCTCATGGCCTGATCATGACTCGAACAATGGTGTAACGGCAGCATGTTCTCATGCTGGAGCGAGAAGCTTCTACGAAAATTTAATATGGCTAGTTAAAAAAGGAAATTCTTACCCAAAACTCAGCAAATCCTACATTAATAATAAAGGGAAAGAAGTTCTCGTGCTATGGGGCATTCACCATCCACCGACTAGTAATGACCAACGGAGTCTCTATCAGAATGAAGATGCATATGTTTTTGTAGGGTCAACAAAATACAGCAAGAAGTTCAAGCCGGAGATAGCAAGAAGACCCAAAGTGAGGGATCAAGAAGGGAGAATGAACTATTACTGGACACTAGTAGAACCGGGAGGCAAAATAACATTCGAAGCAACTGGAAATCTATTGGTACCGAGATATGCATTCGCAATGGAAAAAAATGCTGGATCTGGCATTATCGTTTCAGATACACCAGTCCACGATTGCAATACAACTTGTCAAACACCCAAGGGTGCTATAAACACCAGCCTCCCATTTCAGAATATACATCCGATCACAATTGGGAAATGTCCAAAATATGTAAAAAGCACAAAATTGAGATTGGCCACAGGACTGAGGAATGTCCCGTCTATTCAATCTAGAGGCCTATTTGGGGCCATTGCCGGTTTCATTGAAGGGGGATGGACAGGAATGGTAGATGGGTGGTACGGTTATCACCATCAAAATGAGCAGGGATCAGGATATGCAGCCGATCTGAAGAGCACACAGAATGCCATTGACGAAATTACTAACAAAGTAAATTCTGTTATTGAAAAGATGAATACACAGTTCACAGCAGTAGGTAAAGAGTTCAACCACCTGGAAAAAAGAATAGAGAATTTAAATAAAAAAGTTGATGATGGTTTTCTGGACATTTGGACTTACAATGCTGAACTGTTGGTTCTATTGGAAAATGAAAGAACTTTGGATTACCACGATTCAAATGTGAAGAACTTATATGAAAAAATAAGAAGCCAGTTAAAAAACAATGCCAAGGAAATTGGAAACGGCTGCTTTGAATTTTACCACAAGTGCGATAACACGTGCATGGAAAGTGTCAAAAATGGGACTTATGACTACCCAAAATACTCAGAGGAAGCAAAATTAAACAGAGAGAAAATAGATGGGGTAAAGCTGGAATCAACAAGGATTTACCAGATTTTGGCGATCTATTCAACTGTCGCCAGTTCATTGGTACTGGTAGTCTCCCTGGGGGCAATCAGTTTCTGGATGTGCTCTAATGGGTCTCTACAGTGTAGAATATGTATTTAA

>LC778459.1Influenza A virus H1N101701

ATGAAGGTAATACTAGTAGTTCTGCTGTATACATTTACAACCGCAAATGCAGACACATTATGTATAGGTTATCATGCGAACAATTCAACAGACACTGTAGACACAGTACTAGAAAAGAATGTAACAGTAACACACTCTGTTAATCTTCTGGAAGACAAGCATAACGGAAAACTATGCAAACTAAGAGGGGTAGCCCCATTGCATTTGGGTAAATGTAACATTGCTGGCTGGATCCTGGGAAATCCAGAGTGTGAATCACTCTCCACAGCAAGATCATGGTCCTACATTGTGGAAACATCTAATTCAGACAATGGAACGTGTTACCCAGGAGATTTCATCAATTATGAGGAGCTAAGAGAGCAATTGAGCTCAGTGTCATCATTTGAAAGGTTTGAAATATTCCCCAAGACAAGTTCATGGCCTAATCATGAATCGAACAAGGGTGTAACGGCAGCATGTCCTCACGCTGGAGCAAAAAGCTTCTACAAAAACTTGATATGGCTGGTTAAAAAAGGAAATTCATACCCAAAGCTCAGCCAAACCTACATTAATGATAAAGGGAAAGAAGTCCTCGTGCTGTGGGGCATTCACCATCCATCTACTACTGCTGACCAACAAAGTCTCTATCAGAATGCAGATGCATATGTTTTTGTGGGGACATCAAGATACAGCAAGAAGTTCAAGCCGGAAATAGCAACAAGACCCAAAGTGAGGGATCAAGAAGGGAGAATGAACTATTACTGGACACTAGTAGAGCCGGGAGACAAAATAACATTCGAAGCAACTGGAAATCTAGTGGTACCGAGATACGCATTCACAATGGAAAGAAATGCTGGATCTGGTATTATCATTTCAGATACACCAGTCCACGATTGCAATACAACTTGTCAGACACCCGAGGGTGCTATAAACACCAGCCTCCCATTTCAGAATGTACATCCGATCACAATTGGGAAATGTCCAAAGTATGTAAAAAGCACAAAATTGAGACTGGCCACAGGATTGAGGAATATCCCGTCTATTCAATCTAGAGGCCTATTCGGGGCCATTGCCGGCTTCATTGAAGGGGGGTGGACAGGGATGGTAGATGGATGGTACGGTTATCACCATCAAAATGAGCAGGGGTCAGGATATGCAGCCGATCTGAAGAGCACACAAAATGCCATTGATAAGATTACTAACAAAGTAAATTCTGTTATTGAAAAGATGAATACACAGTTCACAGCAGTGGGTAAAGAGTTCAACCACCTTGAAAAAAGAATAGAGAATCTAAATAAAAAAGTTGATGATGGTTTCCTGGACATTTGGACTTACAATGCCGAATTGTTGGTTCTACTGGAAAATGAAAGAACTTTGGACTATCACGATTCAAATGTGAAGAACTTGTATGAAAAAGTAAGAAACCAGTTAAAAAACAATGCCAAGGAAATTGGAAACGGCTGCTTTGAATTTTACCACAAATGCGATAACACATGCATGGAAAGTGTCAAGAATGAGACTTATGACTACCCAAAATACTCAGAGGAAGCAAAATTAAACAGAGAAAAAATAGATGGAGTAAAGCTGGAATCAACAAGGATCTACCAGATTTTGGCGATCTATTCAACTGTCGCCAGTTCATTGGTACTGGTAGTCTCCCTGGGGGCAATCAGCTTCTGGATGTGCTCTAATGGGTCTCTACAGTGTAGAATATGTATTTAA

>LC638404.1Influenza A virus H1N101701

ATGAAGGCAATACTAGTAGTTCTGCTATATACATTTGCAACCGCAAATGCAGACACATTATGCATAGGTTATCATGCGAACAATTCAACAGACACTGTAGACACAGTACTAGAAAAGAATGTAACAGTAACACACTCTGTTAATCTTCTAGAAGACAAGCATAACGGGAAACTATGCAAACTAAGAGGGGTAGCCCCATTGCATTTGGGTAAATGTAACATTGCTGGCTGGATCCTGGGAAATCCAGAGTGTGAATCACTCTCCACAGCAAGCTCATGGTCCTACATTGTGGAAACATCTAGTTCAGACAATGGAACGTGTTACCCAGGAGATTTCATCGATTATGAGGAGCTAAGAGAGCAATTGAGCTCAGTGTCGTCATTTGAAAGGTTTGAGATATTCCCCAAGACAAGTTCATGGCCCAATCATGACTCGAACAAAGGTGTAACGGCAGCATGTCCTCATGCTGGAGCAAAAAGCTTCTACAAAAATTTAATATGGCTAGTTAAAAAAGGAAGTTCATACCCAAAGCTCAGCAAATCCTACATTAATGATAAAGGGAAAGAAGTCCTCGTGCTATGGGGCATTCACCATCCATCTACTACTGCTGACCAACAAAGTCTCTATCAGAATGCAGATACATATGTTTTTGTGGGGACATCAAGATACAGCAAGAAGTTCAAGCCGGAAATAGCAATAAGACCCAAAGTGAGGGATCAAGAAGGGAGAATGAACTATTACTGGACACTAGTAGAGCCGGGAGACAAAATAACATTCGAAGCAACTGGAAATCTAGTGGTACCGAGATATGCATTCGCAATGGAAAGAAATGCTGGATCTGGTATTATCATTTCAGATACACCAGTCCACGATTGCAATACAACTTGTCAGACACCCAAGGGTGCTATAAACACCAGCCTCCCATTTCAAAATATACATCCGATCACAATTGGAAAATGTCCAAAATATGTAAAAAGCACAAAATTGAGACTGGCCACAGGATTGAGGAATGTCCCGTCTATTCAATCTAGAGGCCTATTTGGGGCCATTGCCGGCTTCATTGAAGGGGGGTGGACAGGGATGGTAGATGGATGGTACGGTTATCACCATCAAAATGAGCAGGGGTCAGGATATGCAGCCGACCTGAAGAGCACACAGAATGCCATTGACAAGATTACTAACAAAGTAAATTCTGTTATTGAAAAGATGAATACACAGTTCACAGCAGTAGGTAAAGAGTTCAACCACCTGGAAAAAAGAATAGAGAATTTAAATAAAAAAGTTGATGATGGTTTCCTGGACATTTGGACTTACAATGCCGAACTGTTGGTTCTATTGGAAAATGAAAGAACTTTGGACTACCACGATTCAAATGTGAAGAACTTATATGAAAAAGTAAGAAACCAGTTAAAAAACAATGCCAAGGAAATTGGAAATGGCTGCTTTGAATTTTACCACAAATGCGATAACACGTGCATGGAAAGTGTCAAAAATGGGACTTATGACTACCCAAAATACTCAGAGGAAGCAAAATTAAACAGAGAAGAAATAGATGGGGTAAAGCTGGAATCAACAAGGATTTACCAGATTTTGGCGATCTATTCAACTGTCGCCAGTTCATTGGTACTGGTAGTCTCCCTGGGGGCAATCAGTTTTTGGATGTGCTCTAATGGGTCTCTACAGTGTAGAATATGTATTTAA

>LC638403.1Influenza A virus H1N101701

ATGAAGGCAATACTAGTAGTTCTGCTATATACATTTGCAACCGCAAATGCAGACACATTATGTATAGGTTACCATGCGAACAATTCAACAGACACTGTAGACACAGTACTAGAAAAGAATGTAACAGTAACACACTCTGTTAATCTTCTAGAAGACAAGCATAACGGGAAACTATGCAAACTAAGAGGGGTAGCCCCATTGCATTTGGGTAAATGTAACATTGCTGGCTGGATCCTGGGAAATCCAGAGTGTGAATCACTCTCCACAGCAAGCTCATGGTCCTACATTGTGGAAACATCTAGTTCAGACAATGGAACGTGTTACCCAGGAGATTTCATCGATTATGAGGAGCTAAGAGAGCAATTGAGCTCAGTGTCATCATTTGAAAGGTTTGAGATATTCCCCAAGACAAGTTCATGGCCCAATCATGACTCGAACAAAGGTGTAACGGCAGCATGTCCTCATGCTGGAGCAAAAGGCTTCTACAAAAATTTAATATGGCTAGTTAAAAAAGGAAATTCATACCCAAAGCTCAGCAAATCCTACATTAATGATAAAGGGAAAGAAGTCCTCGTGCTATGGGGCATTCACCATCCATCTACTACTGCTGACCAACAAAGTCTCTATCAGAATGCAGATACATATGTTTTTGTGGGGACATCAAGATACAGCAAGAAGTTCAAGCCGGAAATAGCAATAAGACCCAAAGTGAGGGATCAAGAAGGGAGAATGAACTATTACTGGACACTAGTAGAGCCGGGAGACAAAATAACATTCGAAGCAACTGGAAATCTAGTGGTACCGAGATATGCATTCGCAATGGAAAGAAATGCTGGATCTGGTATTATCATTTCAGATACACCAGTCCACGATTGCAATACAACTTGTCAGACACCCAAGGGTGCTATAAACACCAGCCTCCCATTTCAGAATATACATCCGATCACAATTGGAAAATGTCCAAAATATGTAAAAAGCACAAAATTGAGACTGGCCACAGGATTGAGGAATGTCCCGTCTATTCAATCTAGAGGCCTATTTGGGGCCATTGCCGGCTTCATTGAAGGGGGGTGGACAGGGATGGTAGATGGATGGTACGGTTATCACCATCAAAATGAGCAGGGGTCAGGATATGCAGCCGACCTGAAGAGCACACAGAATGCCATTGACAAGATTACTAACAAAGTAAATTCTGTTATTGAAAAGATGAATACACAGTTCACAGCAGTAGGTAAAGAGTTCAACCACCTGGAAAAAAGAATAGAGAATTTAAATAAAAAAGTTGATGATGGTTTCCTGGACATTTGGACTTACAATGCCGAACTGTTGGTTCTATTGGAAAATGAAAGAACTTTGGACTACCACGATTCAAATGTGAAGAACTTATATGAAAAGGTAAGAAACCAGTTAAAAAACAATGCCAAGGAAATTGGAAATGGCTGCTTTGAATTTTACCACAAATGCGATAACACGTGCATGGAAAGTGTCAAAAATGGGACTTATGACTACCCAAAATACTCAGAGGAAGCAAAATTAAACAGAGAAGAAATAGATGGGGTAAAACTGGAATCAACAAGGATTTACCAGATTTTGGCGATCTATTCAACTGTCGCCAGTTCATTGGTATTGGTAGTCTCCCTGGGGGCAATCAGTTTTTGGATGTGCTCTAATGGGTCTCTACAGTGTAGAATATGTATTTAA

>LC638402.1Influenza A virus H1N101701

ATGAAGGCAATACTAGTAGTTATGCTATATACATTTGCAACCGCAAATGCAGACACATTATGTATAGGTTATCATGCGAACAATTCTACAGACACTGTAGACACAGTACTAGAAAAGAATGTAACAGTAACACACTCTGTTAATCTTCTAGAAGACAAGCATAACGGGAAACTATGCAAACTAAGAGGGGTAGCCCCATTGCATTTGGGTAAATGTAACATTGCTGGCTGGATCCTGGGAAATCCAGAGTGTGAATCACTCTCCACAGCAAGCTCATGGTCCTACATTGTGGAAACATCTAGTTCAGACAATGGAACGTGTTACCCAGGAGATTTCATCGATTATGAGGAGCTAAGAGAGCAATTGAGCTCAGTGTCATCATTTGAAAGGTTTGAGATATTCCCCAAAACAAGTTCATGGCCCAATCATGACTCGAACAAAGGTGTAACGGCAGCATGTCCTCATGCTGGAGCAAAAGGCTTCTACAAAAATTTAATATGGCTAGTTAAAAAAGGAAATTCATACCCAAAGCTCAGCAAATCCTACATTAATGATAAAGGGAAAGAAGTCCTCGTGCTATGGGGCATTCACCATCCATCTACTACTGCTGACCAACAAAGTCTCTATCAGAATGCAGATACATATGTTTTTGTGGGGACATCAAGATACAGCAAGAAGTTCAAGCCGGAAATAGCAATAAGACCCAAAGTGAGGGATCAAGAAGGGAGAATGAACTATTACTGGACACTAGTAGAGCCGGGAGACAAAATAACATTCGAAGCAACTGGAAATCTAGTGGTACCGAGATATGCATTTGCAATGGAAAGAAATGCTGGATCTGGTATTATCATTTCAGATACACCAGTCCACGATTGCAATACAACTTGTCAGACACCCAAGGGTGCTATAAACACCAGCCTCCCATTTCAGAATATACATCCGATCACAATTGGAAAATGTCCAAAATATGTAAAAAGCACAAAATTGAGACTGGCCACAGGATTGAGGAATGTCCCGTCTATTCAATCTAGAGGCCTATTTGGGGCCATTGCCGGCTTCATTGAAGGGGGGTGGACAGGGATGGTAGATGGATGGTACGGTTATCACCATCAAAATGAGCAGGGGTCAGGATATGCAGCCGACCTGAAGAGCACACAGAATGCCATTGACAAGATTACTAACAAAGTAAATTCTGTTATTGAAAAGATGAATACACAGTTCACAGCAGTAGGTAAAGAGTTCAACCACCTGGAAAAAAGAATAGAGAATTTAAATAAAAAAGTTGATGATGGTTTCCTGGACATTTGGACTTACAATGCCGAACTGTTGGTTCTATTGGAAAATGAAAGAACTTTGGACTACCACGATTCAAATGTGAAGAACTTATATGAAAAGGTAAGAAACCAGTTAAAAAACAATGCCAAGGAAATTGGAAATGGCTGCTTTGAATTTTACCACAAATGCGATAACACGTGCATGGAAAGTGTCAAAAATGGGACTTATGACTACCCAAAATACTCAGAGGAAGCAAAATTAAACAGAGAAGAAATAGATGGGGTAAAGCTGGAATCAACAAGGATTTACCAGATTTTGGCGATCTATTCAACTGTCGCCAGTTCATTGGTACTGGTAGTCTCCCTGGGGGCAATCAGTTTTTGGATGTGCTCTAATGGGTCTCTACAGTGTAGAATATGTATTTAA

>LC638401.1Influenza A virus H1N101701

ATGAAGGCAATACTAGTAGTTCTGCTATATACATTTGCAACCGCAAATGCAGACACATTATGTATAGGTTACCATGCGAACAATTCAACAGACACTGTAGACACAGTACTAGAAAAGAATGTAACAGTAACACACTCTGTTAATCTTCTAGAAGACAAGCATAACGGGAAACTATGCAAACTAAGAGGGGTAGCCCCATTGCATTTGGGTAAATGTAACATTGCTGGCTGGATCCTGGGAAATCCAGAGTGTGAATCACTCTCCACAGCAAGCTCATGGTCCTACATTGTGGAAACATCTAGTTCAGACAATGGAACGTGTTACCCAGGAGATTTCATCGATTATGAGGAGCTAAGAGAGCAATTGAGCTCAGTGTCATCATTTGAAAGGTTTGAGATATTCCCCAAGACAAGTTCATGGCCCAATCATGACTCGAACAAAGGTGTAACGGCAGCATGTCCTCATGCTGGAGCAAAAGGCTTCTACAAAAATTTAATATGGCTAGTTAAAAAAGGAAATTCATACCCAAAGCTCAGCAAATCCTACATTAATGATAAAGGGAAAGAAGTCCTCGTGCTATGGGGCATTCACCATCCATCTACTACTGCTGACCAACAAAGTCTCTATCAGAATGCAGATACATATGTTTTTGTGGGGACATCAAGATACAGCAAGAAGTTCAAGCCGGAAATAGCAATAAGACCCAAAGTGAGGGATCAAGAAGGGAGAATGAACTATTACTGGACACTAGTAGAGCCGGGAGACAAAATAACATTCGAAGCAACTGGAAATCTAGTGGTACCGAGATATGCATTCGCAATGGAAAGAAATGCTGGATCTGGTATTATCATTTCAGATACACCAGTCCACGATTGCAATACAACTTGTCAGACACCCAAGGGTGCTATAAACACCAGCCTTCCATTTCAGAATATACATCCGATCACAATTGGAAAATGTCCAAAATATGTAAAAAGCACAAAATTGAGACTGGCCACAGGATTGAGGAATGTCCCGTCTATTCAATCTAGAGGCCTATTTGGGGCCATTGCCGGCTTCATTGAAGGGGGGTGGACAGGGATGGTAGATGGATGGTACGGTTATCACCATCAAAATGAGCAGGGGTCAGGATATGCAGCCGACCTGAAGAGCACACAGAATGCCATTGACAAGATTACTAACAAAGTAAATTCTGTTATTGAAAAGATGAATACACAGTTCACAGCAGTAGGTAAAGAGTTCAACCACCTGGAAAAAAGAATAGAGAATTTAAATAAAAAAGTTGATGATGGTTTCCTGGACATTTGGACTTACAATGCCGAACTGTTGGTTCTATTGGAAAATGAAAGAACTTTGGACTACCACGATTCAAATGTGAAGAACTTATATGAAAAGGTAAGAAACCAGTTAAAAAACAATGCCAAGGAAATTGGAAATGGCTGCTTTGAATTTTACCACAAATGCGATAACACGTGCATGGAAAGTGTCAAAAATGGGACTTATGACTACCCAAAATACTCAGAGGAAGCAAAATTAAACAGAGAAGAAATAGATGGGGTAAAACTGGAATCAACAAGGATTTACCAGATTTTGGCGATCTATTCAACTGTCGCCAGTTCATTGGTATTGGTAGTCTCCCTGGGGGCAATCAGTTTTTGGATGTGCTCTAATGGGTCTCTACAGTGTAGAATATGTATTTAA

>LC638400.1Influenza A virus H1N101701

ATGAAGGCAATACTAGTAGTTCTGCTATATACATTTGCAACCGCAAATGCAGACACATTATGTATAGGTTACCATGCGAACAATTCAACAGACACTGTAGACACAGTACTAGAAAAGAATGTAACAGTAACACACTCTGTTAATCTTCTAGAAGACAAGCATAACGGGAAACTATGCAAACTAAGAGGGGTAGCCCCATTGCATTTGGGTAAATGTAACATTGCTGGCTGGATCCTGGGAAATCCAGAGTGTGAATCACTCTCCACAGCAAGCTCATGGTCCTACATTGTGGAAACATCTAGTTCAGACAATGGAACGTGTTACCCAGGAGATTTCATCGATTATGAGGAGCTAAGAGAGCAATTGAGCTCAGTGTCATCATTTGAAAGGTTTGAGATATTCCCCAAGACAAGTTCATGGCCCAATCATGACTCGAACAAAGGTGTAACGGCAGCATGTCCTCATGCTGGAGCAAAAGGCTTCTACAAAAATTTAATATGGCTAGTTAAAAAAGGAAATTCATACCCAAAGCTCAGCAAATCCTACATTAATGATAAAGGGAAAGAAGTCCTCGTGCTATGGGGCATTCACCATCCATCTACTACTGCTGACCAACAAAGTCTCTATCAGAATGCAGATACATATGTTTTTGTGGGGACATCAAGATACAGCAAGAAGTTCAAGCCGGAAATAGCAATAAGACCCAAAGTGAGGGATCAAGGAGGGAGAATGAACTATTACTGGACACTAGTAGAGCCGGGAGACAAAATAACATTCGAAGCAACTGGAAATCTAGTGGTACCGAGATATGCATTCGCAATGGAAAGAAATGCTGGATCTGGTATTATCATTTCAGATACACCAGTCCACGATTGCAATACAACTTGTCAGACACCCAAGGGTGCTATAAACACCAGCCTCCCATTTCAGAATATACATCCGATCACAATTGGAAAATGTCCAAAATATGTAAAAAGCACAAAATTGAGACTGGCCACAGGATTGAGGAATGTCCCGTCTATTCAATCTAGAGGCCTATTTGGGGCCATTGCCGGCTTCATTGAAGGGGGGTGGACAGGGATGGTAGATGGATGGTACGGTTATCACCATCAAAATGAGCAGGGGTCAGGATATGCAGCCGACCTGAAGAGCACACAGAATGCCATTGATAAGATTACTAACAAAGTAAATTCTGTTATTGAAAAGATGAATACACAGTTCACAGCAGTAGGTAAAGAGTTCAACCACCTGGAAAAAAGAATAGAGAATTTAAATAAAAAAGTTGATGATGGTTTCCTGGACATTTGGACTTACAATGCCGAACTGTTGGTTCTATTGGAAAATGAAAGAACTTTGGACTACCACGATTCAAATGTGAAGAACTTATATGAAAAGGTAAGAAACCAGTTAAAAAACAATGCCAAGGAAATTGGAAATGGCTGCTTTGAATTTTACCACAAATGCGATAACACGTGCATGGAAAGTGTCAAAAATGGGACTTATGACTACCCAAAATACTCAGAGGAAGCAAAATTAAACAGAGAAGAAATAGATGGGGTAAAACTGGAATCAACAAGGATTTACCAGATTTTGGCGGTCTATTCAACTGTCGCCAGTTCATTGGTATTGGTAGTCTCCCTGGGGGCAATCAGTTTTTGGATGTGCTCTAATGGGTCTCTACAGTGTAGAATATGTATTTAA

>LC638399.1Influenza A virus H1N101701

ATGAAGGCAATACTAGTAGTTCTGCTATATACATTTGCAACCGCAAATGCAGACACATTATGTATAGGTTATCATGCGAACAATTCAACAGACACTGTAGACACAGTACTAGAAAAGAATGTAACAGTAACACACTCTGTTAATCTTCTAGAAGACAAGCATAACGGAAAACTATGCAAACTAAGAGGGGTAGCCCCATTGCATTTGGGTAAATGTAACATTGCTGGCTGGATCCTGGGAAATCCAGAGTGTGAATCACTCTCCACAGCAAGCTCATGGTCCTACATTGTGGAAACATCTAGTTCAGACAATGGGACGTGTTACCCAGGAGATTTCATCGATTATGAGGAGCTAAGAGAGCAATTGAGCTCAGTGTCATCATTTGAAAGGTTTGAGATATTCCCCAAGACAAGTTCATGGCCCAATCATGACTCGAACAAAGGTGTAACGGCAGCATGTCCTCATGCTGGAGCAAAAAGCTTCTACAAAAATTTAATATGGCTAGTTAAAAAAGGAAATTCATACCCAAAGCTCAGCAAATCCTACATTAATGATAAAGGGAAAGAAGTCCTCGTGCTATGGGGCATTCACCATCCATCTACTACTGCTGACCAACAAAGTCTCTATCAGAATGCAGATACATATGTTTTTGTGGGGACATCAAGATACAGCAAGAAGTTCAAGCCGGAAATAGCAATAAGACCCAAAGTGAGGGATCAAGAAGGGAGAATGAACTATTACTGGACACTAGTAGAGCCGGGAGACAAAATAACATTCGAAGCAACTGGAAATCTAGTGGTACCGAGATATGCATTCGCAATGGAAAGAAATGCTGGATCTGGTATTATCATTTCAGATACACCAGTCCACGATTGCAATACAACTTGTCAGACACCCAAGGGTGCTATAAACACCAGCCTCCCATTTCAAAATATACATCCGATCACAATTGGAAAATGTCCAAAATATGTAAAAAGCACAAAATTGAGACTGGCCACAGGATTGAGGAATGTCCCGTCTATTCAATCTAGAGGCCTATTTGGGGCCATTGCCGGCTTCATTGAAGGGGGGTGGACAGGGATGGTAGATGGATGGTACGGTTATCACCATCAAAATGAGCAGGGGTCAGGATATGCAGCCGACCTGAAGAGCACACAGAATGCCATTGACAAGATTACTAACAAAGTAAATTCTGTTATTGAAAAGATGAATACACAGTTCACAGCAGTAGGTAAAGAGTTCAACCACCTGGAAAAAAGAATAGAGAATTTAAATAAAAAAGTTGATGATGGTTTCCTGGACATTTGGACTTACAATGCCGAACTGTTGGTTCTATTGGAAAATGAAAGAACTTTGGACTACCACGATTCAAATGTGAAGAACTTATATGAAAAAGTAAGAAACCAGTTAAAAAACAATGCCAAGGAAATTGGAAATGGCTGCTTTGAATTTTACCACAAATGCGATAACACGTGCATGGAAAGTGTCAAAAATGGGACTTATGACTACCCAAAATACTCAGAGGAAGCAAAATTAAACAGAGAAGAAATAGATGGGGTAAAGCTGGAATCAACAAGGATTTACCAGATTTTGGCGATCTATTCAACTGTCGCCAGTTCATTGGTACTGGTAGTCTCCCTGGGGGCAATCAGTTTTTGGATGTGCTCTAATGGGTCTCTACAGTGTAGAATATGTATTTAA

>LC638398.1Influenza A virus H1N101701

ATGAAGGCAATACTAGTAGTTCTGCTATATACATTTGCAACCGCAAATGCAGACACATTATGTATAGGTTATCATGCGAACAATTCAACAGACACTGTAGACACAGTACTAGAAAAGAATGTAACAGTAACACACTCTGTTAATCTTCTAGAAGACAAGCATAACGGAAAACTATGCAAACTAAGAGGGGTAGCCCCATTGCATTTGGGTAAATGTAACATTGCTGGCTGGATCCTGGGAAATCCAGAGTGTGAATCACTCTCCACAGCAAGCTCATGGTCCTACATTGTGGAAACATCTAGTTCAGACAATGGAACGTGTTACCCAGGAGATTTCATCGATTATGAGGAGCTAAGAGAGCAATTGAGCTCAGTGTCATCATTTGAAAGGTTTGAGATATTCCCCAAGACAAGTTCATGGCCCAATCATGACTCGAACAAAGGTGTAACGGCAGCATGTCCTCATGCTGGAGCAAAAAGCTTCTACAAAAATTTAATATGGCTAGTTAAAAAAGGAAATTCATACCCAAAGCTCAGCAAATCCTACATTAATGATAAAGGGAAAGAAGTCCTCGTGCTATGGGGCATTCACCATCCATCTACTACTGCTGACCAACAAAGTCTCTATCAGAATGCAGATACATATGTTTTTGTGGGGACATCAAGATACAGCAAGAAGTTCAAGCCGGAAATAGCAATAAGACCCAAAGTGAGGGATCAAGAAGGGAGAATGAACTATTACTGGACACTAGTAGAGCCGGGAGACAAAATAACATTCGAAGCAACTGGAAATCTAGTGGTGCCGAGATATGCATTCGCAATGGAAAGAAATGCTGGATCTGGTATTATCATTTCAGATACACCAGTCCACGATTGCAATACAACTTGTCAGACACCCAAGGGTGCTATAAACACCAGCCTCCCATTTCAAGATATACATCCGATCACAATTGGAAAATGTCCAAAATATGTAAAAAGCACAAAATTGAGACTGGCCACAGGATTGAGGAATGTCCCGTCTATTCAATCTAGAGGCCTATTTGGGGCCATTGCCGGCTTCATTGAAGGGGGGTGGACAGGGATGGTAGATGGATGGTACGGTTATCACCATCAAAATGAGCAGGGGTCAGGATATGCAGCCGACCTGAAGAGCACACAGAATGCCATTGACAAGATTACTAACAAAGTAAATTCTGTTATTGAAAAGATGAATACACAGTTCACAGCAGTAGGTAAAGAGTTCAACCACCTGGAAAAAAGAATAGAGAATTTAAATAAAAAAGTTGATGATGGTTTCCTGGACATTTGGACTTACAATGCCGAACTGTTGGTTCTATTGGAAAATGAAAGAACTTTGGACTACCACGATTCAAATGTGAAGAACTTATATGAAAAAGTAAGAAACCAGTTAAAAAACAATGCCAAGGAAATTGGAAATGGCTGCTTTGAATTTTACCACAAATGCGATAACACGTGCATGGAAAGTGTCAAAAATGGGACTTATGACTACCCAAAATACTCAGAGGAAGCAAAATTAAACAGAGAAAAAATAGATGGGGTAAAGCTGGAATCAACAAGGATTTACCAGATTTTGGCGATCTATTCAACTGTCGCCAGTTCATTGGTACTGGTAGTCTCCCTGGGGGCAATCAGTTTTTGGATGTGCTCTAATGGGTCTCTACAGTGTAGAATATGTATTTAA

>LC638397.1Influenza A virus H1N101701

ATGAAGGCAATACTAGTAGTTCTGCTATATACATTTGCAACCGCAAATGCAGACACATTATGTATAGGTTATCATGCGAACAATTCAACAGACACTGTAGACACAGTACTAGAAAAGAATGTAACAGTAACACACTCTGTTAATCTTCTAGAAGACAAGCATAACGGAAAACTATGCAAACTAAGAGGGGTAGCCCCATTGCATTTGGGTAAATGTAACATTGCTGGCTGGATCCTGGGAAATCCAGAGTGTGAATCACTCTCCACAGCAAGCTCATGGTCCTACATTGTGGAAACATCTAGTTCAGACAATGGAACGTGTTACCCAGGAGATTTCATCGATTATGAGGAGCTAAGAGAGCAATTGAGCTCAGTGTCATCATTTGAAAGGTTTGAGATATTCCCCAAGACAAGTTCATGGCCCAATCATGACTCGAACAAAGGTGTAACGGCAGCATGTCCTCATGCTGGAGCAAAAAGCTTCTACAAAAATTTAATATGGCTAGTTAAAAAAGGAAATTCATACCCAAAGCTCAGCAAATCCTACATTAATGATAAAGGGAAAGAAGTCCTCGTGCCATGGGGCATTCACCATCCATCTACTACTGCTGACCAACAAAGTCTCTATCAGAATGCAGATACATATGTTTTTGTGGGGACATCAAGATACAGCAAGAAGTTCAAGCCGGAAATAGCAATAAGACCCAAAGTGAGGGATCAAGAAGGGAGAATGAACTATTACTGGACACTAGTAGAGCCGGGAGACAAAATAACATTCGAAGCAACTGGAAATCTAGTGGTGCCGAGATATGCATTCGCAATGGAAAGAAATGCTGGATCTGGTATTATCATTTCAGATACACCAGTCCACGATTGCAATACAACTTGTCAGACACCCAAGGGTGCTATAAACACCAGCCTCCCATTTCAAGATATACATCCGATCACAATTGGAAAATGTCCAAAATATGTAAAAAGCACAAAATTGAGACTGGCCACAGGATTGAGGAATGTCCCGTCTATTCAATCTAGAGGCCTATTTGGGGCCATTGCCGGCTTCATTGAAGGGGGGTGGACAGGGATGGTAGATGGATGGTACGGTTATCACCATCAAAATGAGCAGGGGTCAGGATATGCAGCCGACCTGAAGAGCACACAGAATGCCATTGACAAGATTACTAACAAAGTAAATTCTGTTATTGAAAAGATGAATACACAGTTCACAGCAGTAGGTAAAGAGTTCAACCACCTGGAAAAAAGAATAGAGAATTTAAATAAAAAAGTTGATGATGGTTTCCTGGACATTTGGACTTACAATGCCGAACTGTTGGTTCTATTGGAAAATGAAAGAACTTTGGACTACCACGATTCAAATGTGAAGAACTTATATGAAAAAGTAAGAAACCAGTTAAAAAACAATGCCAAGGAAATTGGAAATGGCTGCTTTGAATTTTACCACAAATGCGATAACACGTGCATGGAAAGTGTCAAAAATGGGACTTATGACTACCCAAAATACTCAGAGGAAGCAAAATTAAACAGAGAAAAAATAGATGGGGTAAAGCTGGAATCAACAAGGATTTACCAGATTTTGGCGATCTATTCAACTGTCGCCAGTTCATTGGTACTGGTAGTCTCCCTGGGGGCAATCAGTTTTTGGATGTGCTCTAATGGGTCTCTACAGTGTAGAATATGTATTTAA

>LC638396.1Influenza A virus H1N101701

ATGAAGGCAATACTAGTAGTTCTGCTATATACATTTGCAACCGCAAATGCAGACACATTATGTATAGGTTATCATGCGAACAATTCAACAGACACTGTAGACACAGTACTAGAAAAGAATGTAACAGTAACACACTCTGTTAATCTTCTAGAAGACAAGCATAACGGAAAACTATGCAAACTAAGAGGGGTAGCCCCATTGCATTTGGGTAAATGTAACATTGCTGGCTGGATCCTGGGAAATCCAGAGTGTGAATCACTCTCCACAGCAAGCTCATGGTCCTACATTGTGGAAACATCTAGTTCAGACAATGGAACGTGTTACCCAGGAGATTTCATCGATTATGAGGAGCTAAGAGAGCAATTGAGCTCAGTGTCATCATTTGAAAGGTTTGAGATATTCCCCAAGACAAGTTCATGGCCCAATCATGACTCGAACAAAGGTGTAACGGCAGCATGTCCTCATGCTGGAGCAAAAAGCTTCTACAAAAATTTAATATGGCTAGTTAAAAAAGGAAATTCATACCCAAAGCTCAGCAAATCCTACATTAATGATAAAGGGAAAGAAGTCCTCGTGCTATGGGGCATTCACCATCCATCTACTACTGCTGACCAACAAAGTCTCTATCAGAATGCAGATACATATGTTTTTGTGGGGACATCAAGATACAGCAAGAAGTTCAAGCCGGAAATAGCAATAAGACCCAAAGTGAGGGATCAAGAAGGGAGAATGAACTATTACTGGACACTAGTAGAGCCGGGAGACAAAATAACATTCGAAGCAACTGGAAATCTAGTGGTACCGAGATATGCATTCGCAATGGAAAGAAATGCTGGATCTGGTATTATCATTTCAGATACACCAGTCCACGATTGCAATACAACTTGTCAGACACCCAAGGGTGCTATAAACACCAGCCTCCCATTTCAAAATATACATCCGATCACAATTGGAAAATGTCCAAAATATGTAAAAAGCACAAAATTGAGACTGGCCACAGGATTGAGGAATGTCCCGTCTATTCAATCTAGAGGCCTATTTGGGGCCATTGCCGGCTTCATTGAAGGGGGGTGGACAGGGATGGTAGATGGATGGTACGGTTATCACCATCAAAATGAGCAGGGGTCAGGATATGCAGCCGACCTGAAGAGCACACAGAATGCCATTGACAAGATTACTAACAAAGTAAATTCTGTTATTGAAAAGATGAATACACAGTTCACAGCAGTAGGTAAAGAGTTCAACCACCTGGAAAAAAGAATAGAGAATTTAAATAAAAAAGTTGATGATGGTTTCCTGGACATTTGGACTTACAATGCCGAACTGTTGGTTCTATTGGAAAATGAAAGAACTTTGGACTACCACGATTCAAATGTGAAGAACTTATATGAAAAAGTAAGAAACCAGTTAAAAAACAATGCCAAGGAAATTGGAAATGGCTGCTTTGAATTTTACCACAAATGCGATAACACGTGCATGGAAAGTGTCAAAAATGGGACTTATGACTACCCAAAATACTCAGAGGAAGCAAAATTAAACAGAGAAGAAATAGATGGGGTAAAGCTGGAATCAACAAGGATTTACCAGATTTTGGCGATCTATTCAACTGTCGCCAGTTCATTGGTACTGGTAGTCTCCCTGGGGGCAATCAGTTTTTGGATGTGCTCTAATGGGTCTCTACAGTGTAGAATATGTATTTAA

>LC638395.1Influenza A virus H1N101701

ATGAAGGCAATACTAGTAGTTCTGCTATATACATTTGCAACCGCAAATGCAGACACATTATGTATAGGTTATCATGCGAACAATTCAACAGACACTGTAGACACAGTACTAGAAAAGAATGTAACAGTAACACACTCTGTTAATCTTCTAGAAGACAAGCATAACGGAAAACTATGCAAACTAAGAGGGGTAGCCCCATTGCATTTGGGTAAATGTAACATTGCTGGCTGGATCCTGGGAAATCCAGAGTGTGAATCACTCTCCACAGCAGGCTCATGGTCCTACATTGTGGAAACATCTAGTTCAGACAATGGAACGTGTTACCCAGGAGATTTCATCGATTATGAGGAGCTAAGAGAGCAATTGAGCTCAGTGTCATCATTTGAAAGGTTTGAGATATTCCCCAAGACAAGTTCATGGCCCAATCATGACTCGAACAAAGGTGTAACGGCAGCATGTCCTCATGCTGGAGCAAAAAGCTTCTACAAAAATTTAATATGGCTAGTTAAAAAAGGAAATTCATACCCAAAGCTCAGCAAATCCTACATTAATGATAAAGGGAAAGAAGTCCTCGTGCTATGGGGCATTCACCATCCATCTACTACTGCTGACCAACAAAGTCTCTATCAGAATGCAGATACATATGTTTTTGTGGGGACATCAAGATACAGCAAGAAGTTCAAGCCGGAAATAGCAATAAGACCCAAAGTGAGGGATCAAGAAGGGAGAATGAACTATTACTGGACACTAGTAGAGCCGGGAGACAAAATAACATTCGAAGCAACTGGAAATCTAGTGGTACCGAGATATGCATTCGCAATGGAAAGAAATGCTGGATCTGGTATTATCATTTCAGATACACCAGTCCACGATTGCAATACAACTTGTCAGACACCCAAGGGTGCTATAAACACCAGCCTCCCATTTCAAGATGTACATCCGATCACAATTGGAAAATGTCCAAAATATGTAAAAAGCACAAAATTGAGACTGGCCACAGGATTGAGGAATGTCCCGTCTATTCAATCTAGAGGCCTATTTGGGGCCATTGCCGGCTTCATTGAAGGGGGGTGGACAGGGATGGTAGATGGATGGTACGGTTATCACCATCAAAATGAGCAGGGGTCAGGATATGCAGCCGACCTGAAGAGCACACAGAATGCCATTGACAAGATTACTAACAAAGTAAATTCTGTTATTGAAAAGATGAATACACAGTTCACAGCAGTAGGTAAAGAGTTCAACCACCTGGAAAAAAGAATAGAGAATTTAAATAAAAAAGTTGATGATGGTTTCCTGGACATTTGGACTTACAATGCCGAACTGTTGGTTCTATTGGAAAATGAAAGAACTTTGGACTACCACGATTCAAATGTGAAGAACTTATATGAAAAAGTAAGAAACCAGTTAAAAAACAATGCCAAGGAAATTGGAAATGGCTGCTTTGAATTTTACCACAAATGCGATAACACGTGCATGGAAAGTGTCAAAAATGGGACTTATGACTACCCAAAATACTCAGAGGAAGCAAAATTAAACAGAGAAAAAATAGATGGGGTAAAGCTGGAATCAACAAGGATTTACCAGATTTTGGCGATCTATTCAACTGTCGCCAGTTCATTGGTACTGGTAGTCTCCCTGGGGGCAATCAGTTTTTGGATGTGCTCTAATGGGTCTCTACAGTGTAGAATATGTATTTAA

>LC638394.1Influenza A virus H1N101701

ATGAAGGCAATACTAGTAGTTCTGCTATATACATTTGCAACCGCAAATGCAGACACATTATGTATAGGTTATCATGCGAACAATTCAACAGACACTGTAGACACAGTACTAGAAAAGAATGTAACAGTAACACACTCTGTTAATCTTCTAGAAGACAAGCATAACGGGAAACTATGCAAACTAAGAGGGGTAGCCCCATTGCATTTGGGTAAATGTAACATTGCTGGCTGGATCCTGGGAAATCCAGAGTGTGAATCACTCTCCACAGCAAGCTCATGGTCCTACATTGTGGAAACATCTAGTTCAGACAATGGAACGTGTTACCCAGGAGATTTCATCGATTATGAGGAGCTAAGAGAGCAATTGAGCTCAGTGTCATCATTTGAAAGGTTTGAGATATTCCCCAAGACAAGTTCATGGCCCAATCATGACTCGAACAAAGGTGTAACGGCAGCATGTCCTCATGCTGGAGCAAAAAGCTTCTACAAAAATTTAATATGGCTAGTTAAAAAAGGAAATTCATACCCAAAGCTCAGCAAATCCTACATTAATGATAAAGGGAAAGAAGTCCTCGTGCTATGGGGCATTCACCATCCATCTACTACTGCTGACCAACAAAGTCTCTATCAGAATGCAGATACATATGTTTTTGTGGGGACATCAAGATACAGCAAGAAGTTCAAGCCGGAAATAGCAATAAGACCCAAAGTGAGGGATCAAGAAGGGAGAATGAACTATTACTGGACACTAGTAGAGCCGGGAGACAAAATAACATTCGAAGCAACTGGAAATCTAGTGGTACCGAGATATGCATTCGCAATGGAAAGAAATGCTGGATCTGGTATTATCATTTCAGATACACCAGTCCACGATTGCAATACAACTTGTCAGACACCCAAGGGTTCTATAAACACCAGCCTCCCATTTCAAAATATACATCCGATCACAATTGGAAAATGTCCAAAATATGTAAAAAGCACAAAATTGAGACTGGCCACAGGATTGAGGAATGTCCCGTCTATTCAATCTAGAGGCCTATTTGGGGCCATTGCCGGCTTCATTGAAGGGGGGTGGACAGGGATGGTAGATGGATGGTACGGTTATCACCATCAAAATGAGCAGGGGTCAGGATATGCAGCCGACCTGAAGAGCACACAGAATGCCATTGACAAGATTACTAACAAAGTAAATTCTGTTATTGAAAAGATGAATACACAGTTCACAGCAGTAGGTAAAGAGTTCAACCACCTGGAAAAAAGAATAGAGAATTTAAATAAAAAAGTTGATGATGGTTTCCTGGACATTTGGACTTACAATGCCGAACTGTTGGTTCTATTGGAAAATGAAAGAACTTTGGACTACCACGATTCAAATGTGAAGAACTTATATGAAAAAGTAAGAAACCAGTTAAAAAACAATGCCAAGGAAATTGGAAATGGCTGCTTTGAATTTTACCACAAATGCGATAACACGTGCATGGAAAGTGTCAAAAATGGGACTTATGACTACCCAAAATACTCAGAGGAAGCAAAATTAAACAGAGAAGAAATAGATGGGGTAAAGCTGGAATCAACAAGGATTTACCAGATTTTGGCGATCTATTCAACTGTCGCCAGTTCATTGGTACTGATAGTCTCCCTGGGGGCAATCAGTTTTTGGATGTGCTCTAATGGGTCTCTACAGTGTAGAATATGTATTTAA

>LC638393.1Influenza A virus H1N101701

ATGAAGGCAATACTAGTAGTTCTGCTATATACATTTGCAACCGCAAATGCAGACACATTATGTATAGGTTACCATGCGAACAATTCAACAGACACTGTAGACACAGTACTAGAAAAGAATGTAACAGTAACACACTCTGTTAATCTTCTAGAAGACAAGCATAACGGGAAACTATGCAAACTAAGAGGGGTAGCCCCATTGCATTTGGGTAAATGTAACATTGCTGGCTGGATCCTGGGAAATCCAGAGTGTGAATCACTCTCCACAGCAAGCTCATGGTCCTACATTGTGGAAACATCTAGTTCAGACAATGGAACGTGTTACCCAGGAGATTTCATCGATTATGAGGAGCTAAGAGAGCAATTGAGCTCAGTGTCATCATTTGAAAGGTTTGAGATATTCCCCAAGACAAGTTCATGGCCCAATCATGACTCGAACAAAGGTGTAACGGCAGCATGTCCTCATGCTGGAGCAAAAGGCTTCTACAAAAATTTAATATGGCTAGTTAAAAAAGGAAATTCATACCCAAAGCTCAGCAAATCCTACATTAATGATAAAGGGAAAGAAGTCCTCGTGCTATGGGGCATTCACCATCCATCTACTACTGCTGACCAACAAAGTCTCTATCAGAATGCAGATACATATGTTTTTGTGGGGACATCAAGATACAGCAAGAAGTTCAAGCCGGAAATAGCAATAAGACCCAAAGTGAGGGATCAAGAAGGGAGAATGAACTATTACTGGACACTAGTAGAGCCGGGAGACAAAATAACATTCGAAGCAACTGGAAATCTAGTGGTACCGAGATATGCATTCGCAATGGAAAGAAATGCTGGATCTGGTATTATCATTTCAGATACACCAGTCCACGATTGCAATACAACTTGTCAGACACCCAAGGGTGCTATAAACACCAGCCTCCCATTTCAGAATATACATCCGATCACAATTGGAAAATGTCCAAAATATGTAAAAAGCACAAAATTGAGACTGGCCACAGGATTGAGGAATGTCCCGTCTATTCAATCTAGAGGCCTATTTGGGGCCATTGCCGGCTTCATTGAAGGGGGGTGGACAGGGATGGTAGATGGATGGTACGGTTATCACCATCAAAATGAGCAGGGGTCAGGATATGCAGCCGACCTGAAGAGCACACAGAATGCCATTGACAAGATTACTAACAAAGTAAATTCTGTTATTGAAAAGATGAATACACAGTTCACAGCAGTAGGTAAAGAGTTCAACCACCTGGAAAAAAGAATAGAGAATTTAAATAAAAAAGTTGATGATGGTTTCCTGGACATTTGGACTTACAATGCCGAACTGTTGGTTCTATTGGAAAATGAAAGAACTTTGGACTACCACGATTCAAATGTGAAGAACTTATATGAAAAGGTAAGAAACCAGTTAAAAAACAATGCCAAGGAAATTGGAAATGGCTGCTTTGAATTTTACCACAAATGCGATAACACGTGCATGGAAAGTGTCAAAAATGGGACTTATGACTACCCAAAATACTCAGAGGAAGCAAAATTAAACAGAGAAGAAATAGATGGGGTAAAACTGGAACCAACAAGGATTTACCAGATTTTGGCGATCTATTCAACTGTCGCCAGTTCATTGGTATTGGTAGTCTCCCTGGGGGCAATCAGTTTTTGGATGTGCTCTAATGGGTCTCTACAGTGTAGAATATGTATTTAA

>LC638392.1Influenza A virus H1N101701

ATGAAGGCAATACTAGTAGTTCTGCTATATACATTTGCAACCGCAAATGCAGACACATTATGTATAGGTTATCATGCGAACAATTCAACAGACACTGTAGACACAGTACTAGAAAAGAATGTAACAGTAACACACTCTGTTAATCTTCTAGAAGACAAGCATAACGGAAAACTATGCAAACTAAGAGGGGTAGCCCCATTGCATTTGGGTAAATGTAACATTGCTGGCTGGATCCTGGGAAATCCAGAGTGTGAATCACTCTCCACAGCAAGCTCATGGTCCTACATTGTGGAAACATCTAGTTCAGACAATGGAACGTGTTACCCAGGAGATTTCATCGATTATGAGGAGCTAAGAGAGCAATTGAGCTCAGTGTCATCATTTGAAAGGTTTGAGATATTCCCCAAGACAAGTTCATGGCCCAATCATGACTCGAACAAAGGTGTAACGGCAGCATGTCCTCATGCTGGAGCAAAAAGCTTCTACAAAAATTTAATATGGCTAGTTAAAAAAGGAAATTCATACCCAAAGCTCAGCAAATCCTACATTAATGATAAAGGGAAAGAAGTCCTCGTGCTATGGGGCATTCACCATCCATCTACTACTGCTGACCAACAAAGTCTCTATCAGAATGCAGATACATATGTTTTTGTGGGGACATCAAGATACAGCAAGAAGTTCAAGCCGGAAATAGCAATAAGACCCAAAGTGAGGGATCAAGAAGGGAGAATGAACTATTACTGGACACTAGTAGAGCCGGGAGACAAAATAACATTCGAAGCAACTGGAAATCTAGTGGTACCGAGATATGCATTCGCAATGGAAAGAAATGCTGGATCTGGTATTATCATTTCAGATACACCAGTCCACGATTGCAATACAACTTGTCAGACACCCAAGGGTGCTATAAACACCAGCCTCCCATTTCAAGATATACATCCGATCACAATTGGAAAATGTCCAAAATATGTAAAAAGCACAAAATTGAGACTGGCCACAGGATTGAGGAATGTCCCGTCTATTCAATCTAGAGGCCTATTTGGGGCCATTGCCGGCTTCATTGAAGGGGGGTGGACAGGGATGGTAGATGGATGGTACGGTTATCACCATCAAAATGAGCAGGGGTCAGGATATGCAGCCGACCTGAAGAGCACACAGAATGCCATTGACAAGATTACTAACAAAGTAAATTCTGTTATTGAAAAGATGAATACACAGTTCACAGCAGTAGGTAAAGAGTTCAACCACCTGGAAAAAAGAATAGAGAATTTAAATAAAAAAGTTGATGATGGTTTCCTGGACATTTGGACTTACAATGCCGAACTGTTGGTTCTATTGGAAAATGAAAGAACTTTGGACTACCACGATTCAAATGTGAAGAATTTATATGAAAAAGTAAGAAACCAGTTAAAAAACAATGCCAAGGAAATTGGAAATGGCTGCTTTGAATTTTACCACAAATGCGATAACACGTGCATGGAAAGTGTCAAAAATGGGACTTATGACTACCCAAAATACTCAGAGGAAGCAAAATTAAACAGAGAAAAAATAGATGGGGTAAAGCTGGAATCAACAAGGATTTACCAGATTTTGGCGATCTATTCAACTGTCGCCAGTTCATTGGTACTGGTAGTCTCCCTGGGGGCAATCAGTTTTTGGATGTGCTCTAATGGGTCTCTACAGTGTAGAATATGTATTTAA

>LC638391.1Influenza A virus H1N101701

ATGAAGGCAATACTAGTAGTTCTGCTATATACATTTGCAACCGCAAATGCAGACACATTATGTATAGGTTACCATGCGAACAATTCAACAGACACTGTAGACACAGTACTAGAAAAGAATGTAACAGTAACACACTCTGTTAATCTTCTAGAAGACAAGCATAACGGGAAACTATGCAAACTAAGAGGGGTAGCCCCATTGCATTTGGGTAAATGTAACATTGCTGGCTGGATCCTGGGAAATCCAGAGTGTGAATCACTCTCCACAGCAAGCTCATGGTCCTACATTGTGGAAACATCTAGTTCAGACAATGGAACGTGTTACCCAGGAGATTTCATCGATTATGAGGAGCTAAGAGAGCAATTGAGCTCAGTGTCATCATTTGAAAGGTTTGAGATATTCCCCAAGACAAGTTCATGGCCCAATCATGACTCGAACAAAGGTGTAACGGCAGCATGTCCTCATGCTGGAGCAAAAGGCTTCTACAAAAATTTAATATGGCTAGTTAAAAAAGGAAATTCATACCCAAAGCTCAGCAAATCCTACATTAATGATAAAGGGAAAGAAGTCCTCGTGCTATGGGGCATTCACCATCCATCTACTACTGCTGACCAACAAAGTCTCTATCAGAATGCAGATACATATGTTTTTGTGGGGACATCAAGATACAGCAAGAAGTTCAAGCCGGAAATAGCAATAAGACCCAAAGTGAGGGATCAAGAAGGGAGAATGAACTATTACTGGACACTAGTAGAGCCGGGAGACAAAATAACATTCGAAGCAACTGGAAATCTAGTGGTACCGAGATATGCATTCGCAATGGAAAGAAATGCTGGATCTGGTATTATCATTTCAGATACACCAGTCCACGATTGCAATACAACTTGTCAGACACCCAAGGGTGCTATAAACACCAGCCTCCCATTTCAGAATATACATCCGATCACAATTGGAAAATGTCCAAAATATGTAAAAAGCACAAAATTGAGACTGGCCACAGGATTGAGGAATGTCCCGTCTATTCAATCTAGAGGCCTATTTGGGGCCATTGCCGGCTTCATTGAAGGGGGGTGGACAGGGATGGTAGATGGATGGTACGGTTATCACCATCAAAATGAGCAGGGGTCAGGATATGCAGCCGACCTGAAGAGCACACAGAATGCCATTGACAAGATTACTAACAAAGTAAATTCTGTTATTGAAAAGATGAATACACAGTTCACAGCAGTAGGTAAAGAGTTCAACCACCTGGAAAAAAGAATAGAGAATTTAAATAAAAAAGTTGATGATGGTTTCCTGGACATTTGGACTTACAATGCCGAACTGTTGGTTCTATTGGAAAATGAAAGAACTTTGGACTACCACGATTCAAATGTGAAGAACTTATATGAAAAGGTAAGAAACCAGTTAAAAAACAATGCCAAGGAAATTGGAAATGGCTGCTTTGAATTTTACCACAAATGCGATAACACGTGCATGGAAAGTGTCAAAAATGGGACTTATGACTACCCAAAATACTCAGAGGAAGCAAAATTAAACAGAGAAGAAATAGATGGGGTAAAACTGGAACCAACAAGGATTTACCAGATTTTGGCGATCTATTCAACTGTCGCCAGTTCATTGGTATTGGTAGTCTCCCTGGGGGCAATCAGTTTTTGGATGTGCTCTAATGGGTCTCTACAGTGTAGAATATGTATTTAA

>LC638390.1Influenza A virus H1N101701

ATGAAGGCAATACTAGTAGTTCTGCTATATACATTTGCAACCGCAAATGCAGACACATTATGTATAGGTTACCATGCGAACAATTCAACAGACACTGTAGACACAGTACTAGAAAAGAATGTAACAGTAACACACTCTGTTAATCTTCTAGAAGACAAGCATAACGGGAAACTATGCAAACTAAGAGGGGTAGCCCCATTGCATTTGGGTAAATGTAACATTGCTGGCTGGATCCTGGGAAATCCAGAGTGTGAATCACTCTCCACAGCAAGCTCATGGTCCTACATTGTGGAAACATCTAGTTCAGACAATGGAACGTGTTACCCAGGAGATTTCATCGATTATGAGGAGCTAAGAGAGCAATTGAGCTCAGTGTCATCATTTGAAAGGTTTGAGATATTCCCCAAGACAAGTTCATGGCCCAATCATGACTCGAACAAAGGTGTAACGGCAGCATGTCCTCATGCTGGGGCAAAAGGCTTCTACAAAAATTTAATATGGCTAGTTAAAAAAGGAAATTCATACCCAAAGCTCAGCAAATCCTACATTAATGATAAAGGGAAAGAAGTCCTCGTGCTATGGGGCATTCACCATCCATCTACTACTGCTGACCAACAAAGTCTCTATCAGAATGCAGATACATATGTTTTTGTGGGGACATCAAGATACAGCAAGAAGTTCAAGCCGGAAATAGCAATAAGACCCAAAGTGAGGGATCAAGAAGGGAGAATGAACTATTACTGGACACTAGTAGAGCCGGGAGACAAAATAACATTCGAAGCAACTGGAAATCTAGTGGTACCGAGATATGCATTCGCAATGGAAAGAAATGCTGGATCTGGTATTATCATTTCAGATACACCAGTCCACGATTGCAATACAACTTGTCAGACACCCAAGGGTGCTATAAACACCAGCCTCCCATTTCAGAATATACATCCGATCACAATTGGAAAATGTCCAAAATATGTAAAAAGCACAAAATTGAGACTGGCCACAGGATTGAGGAATGTCCCGTCTATTCAATCTAGAGGCCTATTTGGGGCCATTGCCGGCTTCATTGAAGGGGGGTGGACAGGGATGGTAGATGGATGGTACGGTTATCACCATCAAAATGAGCAGGGGTCAGGATATGCAGCCGACCTGAAGAGCACACAGAATGCCATTGACAAGATTACTAACAAAGTAAATTCTGTTATTGAAAAGATGAATACACAGTTCACAGCAATAGGTAAAGAGTTCAACCACCTGGAAAAAAGAATAGAGAATTTAAATAAAAAAGTTGATGATGGTTTCCTGGACATTTGGACTTACAATGCCGAACTGTTGGTTCTATTGGAAAATGAAAGAACTTTGGACTACCACGATTCAAATGTGAAGAACTTATATGAAAAGGTAAGAAACCAGTTAAAAAACAATGCCAAGGAAATTGGAAATGGCTGCTTTGAATTTTACCACAAATGCGATAACACGTGCATGGAAAGTGTCAAAAATGGGACTTATGACTACCCAAAATACTCAGAGGAAGCAAAATTAAACAGAGAAGAAATAGATGGGGTAAAACTGGAATCAACAAGGATTTACCAGATTTTGGCGATCTATTCAACTGTCGCCAGTTCATTGGTATTGGTAGTCTCCCTGGGGGCAATCAGTTTTTGGATGTGCTCTAATGGGTCTCTACAGTGTAGAATATGTATTTAA

>LC638389.1Influenza A virus H1N101701

ATGAAGGCAATACTAGTAGTTCTGCTATATACATTTGCAACCGCAAATGCAGACACATTATGTATAGGTTATCATGCGAACAATTCAACAGACACTGTAGACACAGTACTAGAAAAGAATGTAACAGTAACACACTCTGTTAATCTTCTAGAAGACAAGCATAACGGAAAACTATGCAAACTAAGAGGGGTAGCCCCATTGCATTTGGGTAAATGTAACATTGCTGGCTGGATCCTGGGAAATCCAGAGTGTGAATCACTCTCCACAGCAAGCTCATGGTCCTACATTGTGGAAACATCTAGTTCAGACAATGGAACGTGTTACCCAGGAGATTTCATCGATTATGAGGAGCTAAGAGAGCAATTGAGCTCAGTGTCATCATTTGAAAGGTTTGAGATATTCCCCAAGACAAGTTCATGGCCCAATCATGACTCGAACAAAGGTGTAACGGCAGCATGTCCTCATGCTGGAGCAAAAAGCTTCTACAAAAATTTAATATGGCTAGTTAAAAAAGGAAATTCATACCCAAAGCTCAGCAAATCCTACATTAATGATAAAGGGAAAGAAGTCCTCGTGCTATGGGGCATTCACCATCCATCTACTACTGCTGACCAACAAAGTCTCTATCAGAATGCAGATACATATGTTTTTGTGGGGACATCAAGATACAGCAAGAAGTTCAAGCCGGAAATAGCAATAAGACCCAAAGTGAGGGATCAAGAAGGGAGAATGAACTATTACTGGACACTAGTAGAGCCGGGAGACAAAATAACATTCGAAGCAACTGGAAATCTAGTGGTGCCGAGATATGCATTCGCAATGGAAAGAAATGCTGGATCTGGTATTATCATTTCAGATACACCAGTCCACGATTGCAATACAACTTGTCAGACACCCAAGGGTGCTATAAACACCAGCCTCCCATTTCAAGATATACATCCGATCACAATTGGAAAATGTCCAAAATATGTAAAAAGCACAAAATTGAGACTGGCCACAGGATTGAGGAATGTCCCGTCTATTCAATCTAGAGGCCTATTTGGGGCCATTGCCGGCTTCATTGAAGGGGGGTGGACAGGGATGGTAGATGGATGGTACGGTTATCACCATCAAAATGAGCAGGGGTCAGGATATGCAGCCGACCTGAAGAGCACACAGAATGCCATTGACAAGATTACTAACAAAGTAAATTCTGTTATTGAAAAGATGAATACACAGTTCACAGCAGTAGGTAAAGAGTTCAACCACCTGGAAAAAAGAATAGAGAATTTAAATAAAAAAGTTGATGATGGTTTCCTGGACATTTGGACTTACAATGCCGAACTGTTGGTTCTATTGGAAAATGAAAGAACTTTGGACTACCACGATTCAAATGTGAAGAACTTATATGAAAAAGTAAGAAACCAGTTAAAAAACAATGCCAAGGAAATTGGAAATGGCTGCTTTGAATTTTACCACAAATGCGATAACACGTGCATGGAAAGTGTCAAAAATGGGACTTATGACTACCCAAAATACTCAGAGGAAGCAAAATTAAACAGAGAAAAAATAGATGGGGTAAAGCTGGAATCAACAAGGATTTACCAGATTTTGGCGATCTATTCAACTGTCGCCAGTTCATTGGTACTGGTAGTCTCCCTGGGGGCAATCAGTTTTTGGATGTGCTCTAATGGGTCTCTACAGTGTAGAATATGTATTTAA

>LC638388.1Influenza A virus H1N101701

ATGAAGGCAATACTAGTAGTTCTGCTATATACATTTGCAACCGCAAATGCAGACACATTATGTATAGGTTACCATGCGAACAATTCAACAGACACTGTAGACACAGTACTAGAAAAGAATGTAACAGTAACACACTCTGTTAATCTTCTAGAAGACAAGCATAACGGGAAACTATGCAAACTAAGAGGGGTAGCCCCATTGCATTTGGGTAAATGTAACATTGCTGGCTGGATCCTGGGAAATCCAGAGTGTGAATCACTCTCCACAGCAAGCTCATGGTCCTACATTGTGGAAACATCTAGTTCAGACAATGGAACGTGTTACCCAGGAGATTTCATCGATTATGAGGAGCTAAGAGAGCAATTGAGCTCAGTGTCATCATTTGAAAGGTTTGAGATATTCCCCAAGACAAGTTCATGGCCCAATCATGACTCGAACAAAGGTGTAACGGCAGCATGTCCTCATGCTGGAGCAAAAGGCTTCTACAAAAATTTAATATGGCTAGTTAAAAAAGGAAATTCATACCCAAAGCTCAGCAAATCCTACATTAATGATAAAGGGAAAGAAGTCCTCGTGCTATGGGGCATTCACCATCCATCTACTACTGCTGACCAACAAAGTCTCTATCAGAATGCAGATACATATGTTTTTGTGGGGACATCAAGATACAGCAAGAAGTTCAAGCCGGAAATAGCAATAAGACCCAAAGTGAGGGATCAAGAAGGGAGAATGAACTATTACTGGACACTAGTAGAGCCGGGAGACAAAATAACATTCGAAGCAACTGGAAATCTAGTGGTACCGAGATATGCATTCGCAATGGAAAGAAATGCTGGATCTGGTATTATCATTTCAGATACACCAGTCCACGATTGCAATACAACTTGTCAGACACCCAAGGGTGCTATAAACACCAGCCTCCCATTTCAGAATATACATCCGATCACAATTGGAAAATGTCCAAAATATGTAAAAAGCACAAAATTGAGACTGGCCACAGGATTGAGGAATGTCCCGTCTATTCAATCTAGAGGCCTATTTGGGGCCATTGCCGGCTTCATTGAAGGGGGGTGGACAGGGATGGTAGATGGATGGTACGGTTATCACCATCAAAATGAGCAGGGGTCAGGATATGCAGCCGACCTGAAGAGCACACAGAATGCCATTGACAAGATTACTAACAAAGTAAATTCTGTTATTGAAAAGATGAATACACAGTTCACAGCAGTAGGTAAAGAGTTCAACCACCTGGAAAAAAGAATAGAGAATTTAAATAAAAAAGTTGATGATGGTTTCCTGGACATTTGGACTTACAATGCCGAACTGTTGGTTCTATTGGAAAATGAAAGAACTTTGGACTACCACGATTCAAATGTGAAGAACTTATATGAAAAGGTAAGAAACCAGTTAAAAAACAATGCCAAGGAAATTGGAAATGGCTGCTTTGAATTTTACCACAAATGCGATAACACGTGCATGGAAAGTGTCAAAAATGGGACTTATGACTACCCAAAATACTCAGAGGAAGCAAAATTAAACAGAGAAGAAATAGATGGGGTAAAACTGGAACCAACAAGGATTTACCAGATTTTGGCGATCTATTCAACTGTCGCCAGTTCATTGGTATTGGTAGTCTCCCTGGGGGCAATCAGTTTTTGGATGTGCTCTAATGGGTCTCTACAGTGTAGAATATGTATTTAA

>LC638387.1Influenza A virus H1N101701

ATGAAGGCAATACTAGTAGTTCTGCTATATACATTTGCAACCGCAAATGCAGACACATTATGTATAGGTTACCATGCGAACAATTCAACAGACACTGTAGACACAGTACTAGAAAAGAATGTAACAGTAACACACTCTGTTAATCTTCTAGAAGACAAGCATAACGGGAAACTATGCAAACTAAGAGGGGTAGCCCCATTGCATTTGGGTAAATGTAACATTGCTGGCTGGATCCTGGGAAATCCAGAGTGTGAATCACTCTCCACAGCAAGCTCATGGTCCTACATTGTGGAAACATCTAGTTCAGACAATGGAACGTGTTACCCAGGAGATTTCATCGATTATGAGGAGCTAAGAGAGCAATTGAGCTCAGTGTCATCATTTGAAAGGTTTGAGATATTCCCCAAGACAAGTTCATGGCCCAATCATGACTCGAACAAAGGTGTAACGGCAGCATGTCCTCATGCTGGAGCAAAAGGCTTCTACAAAAATTTAATATGGCTAGTTAAAAAAGGAAATTCATACCCAAAGCTCAGCAAATCCTACATTAATGATAAAGGGAAAGAAGTCCTCGTGCTATGGGGCATTCACCATCCATCTACTACTGCTGACCAACAAAGTCTCTATCAGAATGCAGATACATATGTTTTTGTGGGGACATCAAGATACAGCAAGAAGTTCAAGCCGGAAATAGCAATAAGACCCAAAGTGAGGGATCAAGAAGGGAGAATGAACTATTACTGGACACTAGTAGAGCCGGGAGACAAAATAACATTCGAAGCAACTGGAAATCTAGTGGTACCGAGATATGCATTCGCAATGGAAAGAAATGCTGGATCTGGTATTATCATTTCAGATACACCAGTCCACGATTGCAATACAACTTGTCAGACACCCAAGGGTGCTATAAACACCAGCCTCCCATTTCAGAATATACATCCGATCACAATTGGAAAATGTCCAAAATATGTAAAAAGCACAAAATTGAGACTGGCCACAGGATTGAGGAATGTCCCGTCTATTCAATCTAGAGGCCTATTTGGGGCCATTGCCGGCTTCATTGAAGGGGGGTGGACAGGGATGGTAGATGGATGGTACGGTTATCACCATCAAAATGAGCAGGGGTCAGGATATGCAGCCGACCTGAAGAGCACACAGAATGCCATTGACAAGATTACTAACAAAGTAAATTCTGTTATTGAAAAGATGAATACACAGTTCACAGCAGTAGGTAAAGAGTTCAACCACCTGGAAAAAAGAATAGAGAATTTAAATAAAAAAGTTGATGATGGTTTCCTGGACATTTGGACTTACAATGCCGAACTGTTGGTTCTATTGGAAAATGAAAGAACTTTGGACTACCACGATTCAAATGTGAAGAACTTATATGAAAAGGTAAGAAACCAGTTAAAAAACAATGCCAAGGAAATTGGAAATGGCTGCTTTGAATTTTACCACAAATGCGATAACACGTGCATGGAAAGTGTCAAAAATGGGACTTATGACTACCCAAAATACTCAGAGGAAGCAAAATTAAACAGAGAAGAAATAGATGGGGTAAAACTGGAACCAACAAGGATTTACCAGATTTTGGCGATCTATTCAACTGTCGCCAGTTCATTGGTATTGGTAGTCTCCCTGGGGGCAATCAGTTTTTGGATGTGCTCTAATGGGTCTCTACAGTGTAGAATATGTATTTAA

>LC638386.1Influenza A virus H1N101701

ATGAAGGCAATACTAGTAGTTCTGCTATATACATTTGCAACCGCAAATGCAGACACATTATGTATAGGTTACCATGCGAACAATTCAACAGACACTGTAGACACAGTACTAGAAAAGAATGTAACAGTAACACACTCTGTTAATCTTCTAGAAGACAAGCATAACGGGAAACTATGCAAACTAAGAGGGGTAGCCCCATTGCATTTGGGTAAATGTAACATTGCTGGCTGGATCCTGGGAAATCCAGAGTGTGAATCACTCTCCACAGCAAGCTCATGGTCCTACATTGTGGAAACATCTAGTTCAGACAATGGAACGTGTTACCCAGGAGATTTCATCGATTATGAGGAGCTAAGAGAGCAATTGAGCTCAGTGTCATCATTTGAAAGGTTTGAGATATTCCCCAAGACAAGTTCATGGCCCAATCATGACTCGAACAAAGGTGTAACGGCAGCATGTCCTCATGCTGGAGCAAAAGGCTTCTACAAAAATTTAATATGGCTAGTTAAAAAAGGAAATTCATACCCAAAGCTCAGCAAATCCTACATTAATGATAAAGGGAAAGAAGTCCTCGTGCTATGGGGCATTCACCATCCATCTACTACTGCTGACCAACAAAGTCTCTATCAGAATGCAGATACATATGTTTTTGTGGGGACATCAAGATACAGCAAGAAGTTCAAGCCGGAAATAGCAATAAGACCCAAAGTGAGGGATCAAGAAGGGAGAATGAACTATTACTGGACACTAGTAGAGCCGGGAGACAAAATAACATTCGAAGCAACTGGAAATCTAGTGGTACCGAGATATGCATTCGCAATGGAAAGAAATGCTGGATCTGGTATTATCATTTCAGATACACCAGTCCACGATTGCAATACAACTTGTCAGACACCCAAGGGTGCTATAAACACCAGCCTCCCATTTCAGAATATACATCCGATCACAATTGGAAAATGTCCAAAATATGTAAAAAGCACAAAATTGAGACTGGCCACAGGATTGAGGAATGTCCCGTCTATTCAATCTAGAGGCCTATTTGGGGCCATTGCCGGCTTCATTGAAGGGGGGTGGACAGGGATGGTAGATGGATGGTACGGTTATCACCATCAAAATGAGCAGGGGTCAGGATATGCAGCCGACCTGAAGAGCACACAGAATGCCATTGACAAGATTACTAACAAAGTAAATTCTGTTATTGAAAAGATGAATACACAGTTCACAGCAGTAGGTAAAGAGTTCAACCACCTGGAAAAAAGAATAGAGAATTTAAATAAAAAAGTTGATGATGGTTTCCTGGACATTTGGACTTACAATGCCGAACTGTTGGTTCTATTGGAAAATGAAAGAACTTTGGACTACCACGATTCAAATGTGAAGAACTTATATGAAAAGGTAAGAAACCAGTTAAAAAACAATGCCAAGGAAATTGGAAATGGCTGCTTTGAATTTTACCACAAATGCGATAACACGTGCATGGAAAGTGTCAAAAATGGGACTTATGACTACCCAAAATACTCAGAGGAAGCAAAATTAAACAGAGAAGAAATAGATGGGGTAAAACTGGAACCAACAAGGATTTACCAGATTTTGGCGATCTATTCAACTGTCGCCAGTTCATTGGTATTGGTAGTCTCCCTGGGGGCAATCAGTTTTTGGATGTGCTCTAATGGGTCTCTACAGTGTAGAATATGTATTTAA

>LC638385.1Influenza A virus H1N101701

ATGAAGGCAATACTAGTAGTTCTGCTATATACATTTGCAACCGCAAATGCAGACACATTATGCATAGGTTATCATGCGAACAATTCAACAGACACTGTAGACACAGTACTAGAAAAGAATGTAACAGTAACACACTCTGTTAACCTTCTAGAAGACAAGCATAACGGGAAACTATGCAAACTAAGAGGGGTAGCCCCATTGCATTTGGGTAAATGTAACATTGCTGGCTGGATCCTGGGAAATCCAGAGTGTGAATCACTCTCCACAGCAAGCTCATGGTCCTACATTGTGGAAACATCTAGTTCAGACAATGGAACGTGTTACCCAGGAGATTTCATCAATTATGAGGAGCTAAGAGAGCAATTGAGCTCAGTGTCATCATTTGAAAGGTTTGAGATATTCCCCAAGACAAGTTCATGGCCCAATCATGACTCGAACAAAGGTGTAACGGCAGCATGTCCTCATGCTGGAGCAAAAAGCTTCTACAAAAATTTAATATGGCTAGTTAAAAAAGGAAATTCATACCCAAAGCTCAGCAAATCCTACATTAATGATAAAGGGAAAGAAGTCCTCGTGCTGTGGGGCATTCACCATCCATCTACTACTGCTGACCAACAAAGTCTCTATCAGAATGCAGATGCATATGTTTTTGTGGGGACATCAAGATACAGCAAGAAGTTCAAGCCGGAAATAGCAATAAGACCCAAAGTGAGGGATCAAGAAGGGAGAATGAACTATTACTGGACACTAGTAGAGCCGGGAGACAAAATAACATTCGAAGCAACTGGAAATCTAGTGGTACCGAGATATGCATTCGCAATGGAAAGAAATGCTGGATCTGGTATTATCATTTCAGATACACCAGTCCACGATTGCAATACAACTTGTCAGACACCCAAGGGTGCTATAAACACCAGCCTCCCATTTCAGAATATACATCCGATCACAATTGGAAAATGTCCAAAATATGTAAAAAGCACAAAATTGAGACTGGCCACAGGATTGAGGAATGTCCCGTCTATTCAATCTAGAGGCCTATTTGGGGCCATTGCCGGCTTCATTGAAGGGGGGTGGACAGGGATGGTAGATGGATGGTACGGTTATCACCATCAAAATGAGCAGGGGTCAGGATATGCAGCCGACCTGAAGAGCACACAGAATGCCATTGACAAGATTACTAACAAAGTAAATTCTGTTATTGAAAAGATGAATACACAGTTCACAGCAGTAGGTAAAGAGTTCAACCACCTGGAAAAAAGAATAGAGAATTTAAATAAAAAAGTTGATGATGGTTTCCTGGACATTTGGACTTACAATGCCGAACTGTTGGTTCTATTGGAAAATGAAAGAACTTTGGACTACCACGATTCAAATGTGAAGAACTTGTATGAAAAGGTAAGAAACCAGTTAAAAAACAATGCCAAGGAAATTGGAAACGGCTGCTTTGAATTTTACCACAAATGCGATAACACGTGCATGGAAAGTGTCAAAAATGGGACTTATGACTACCCAAAATACTCAGAGGAAGCAAAATTAAACAGAGAAGAAATAGATGGGGTAAAGCTGGAATCAACAAGGATTTACCAGATTTTGGCGATCTATTCAACTGTCGCCAGTTCATTGGTACTGGTAATCTCCCTGGGGGCAATCAGTTTCTGGATGTGCTCTAATGGGTCTCTACAGTGTAGAATATGTATTTAA

>LC638384.1Influenza A virus H1N101701

ATGAAGGCAATACTAGTAGTTCTGCTATATACATTTGCAACCGCAAATGCAGACACATTATGTATAGGTTATCATGCGAACAATTCAACAGACACTGTAGACACAGTACTAGAAAAGAATGTAACAGTAACACACTCTGTTAATCTTCTAGAAGACAAGCATAACGGGAAACTATGCAAACTAAGAGGGGTAGCCCCATTGCATTTGGGTAAATGTAACATTGCTGGCTGGATCCTGGGAAATCCAGAGTGTGAATCACTCTCCACAGCAAGCTCATGGTCCTACATTGTGGAAACATCTAGTTCAGACAATGGAACGTGTTACCCAGGAGATTTCATCGATTATGAGGAGCTAAGAGAGCAATTGAGCTCAGTGTCATCGTTTGAAAGGTTTGAGATATTCCCCAAGACAAGTTCATGGCCCAATCATGACTCGAACAAAGGTGTAACGGCAGCATGTCCTCATGCTGGAGCAAAAAGCTTCTACAAAAACTTAATATGGCTAGTTAAAAAAGGAAATTCATACCCAAAGCTCAGCAAATCCTACATTAATGATAAAGGGAAAGAAGTCCTCGTGCTATGGGGCATTCACCATCCATCTACTACTGCTGACCAACAAAGTCTCTATCAGAATGCAGATACATATGTTTTTGTGGGGACATCAAGATACAGCAAGAAGTTCAAGCCGGAAATAGCAATAAGACCCAAAGTGAGGGATCAAGAAGGGAGAATGAACTATTACTGGACACTAGTAGAGCCGGGAGACAAAATAACATTCGAAGCAACTGGAAATCTAGTGGTACCGAGATATGCATTCGCAATGGAAAGAAATGCTGGATCTGGTATTATCATTTCAGATACACCAGTCCACGATTGCAATACAACTTGTCAGACACCCAAGGGTGCTATAAACACCAGCCTCCCATTTCAAAATATACATCCGATCACAATTGGAAAATGTCCAAAATATGTAAAAAGCACAAAATTGAGACTGGCCACAGGATTGAGGAATGTCCCGTCTATTCAATCTAGAGGCCTATTTGGGGCCATTGCCGGCTTCATTGAAGGGGGGTGGACAGGGATGGTAGATGGATGGTACGGTTATCACCATCAAAATGAGCAGGGGTCAGGATATGCAGCCGACCTGAAGAGCACACAGAATGCCATTGACAAGATTACTAACAAAGTAAATTCTGTTATTGAAAAGATGAATACACAGTTCACAGCAGTAGGTAAAGAGTTCAACCACCTGGAAAAAAGAATAGAGAATTTAAATAAAAAAGTTGATGATGGTTTCCTGGACATTTGGACTTACAATGCCGAACTGTTGGTTCTATTGGAAAATGAAAGAACTTTGGACTACCACGATTCAAATGTGAAGAACTTATATGAAAAAGTAAGAAACCAGTTAAAAAACAATGCCAAGGAAATTGGAAATGGCTGCTTTGAATTTTACCACAAATGCGATAACACGTGCATGGAAAGTGTCAAAAATGGGACTTATGACTACCCAAAATACTCAGAGGAAGCAAAATTAAACAGAGAAGAAATAGATGGAGTAAAGCTGGAATCAACAAGGATTTACCAGATTTTGGCGATCTATTCAACTGTCGCCAGTTCATTGGTACTGGTAGTCTCCCTGGGGGCAATCAGTTTTTGGATGTGCTCTAATGGGTCTCTACAGTGTAGAATATGTATTTAA

>LC638383.1Influenza A virus H1N101701

ATGAAGGCAATACTAGTAGTTCTGCTATATACATTTGCAACCGCAAATGCAGACACATTATGTATAGGTTACCATGCGAACAATTCAACAGACACTGTAGACACAGTACTAGAAAAGAATGTAACAGTAACACACTCTGTTAATCTTCTAGAAGACAAGCATAACGGGAAACTATGCAAACTAAGAGGGGTAGCCCCATTGCATTTGGGTAAATGTAACATTGCTGGCTGGATCCTGGGAAATCCAGAGTGTGAATCACTCTCCACAGCAAGCTCATGGTCCTACATTGTGGAAACATCTAGTTCAGACAATGGAACGTGTTACCCAGGAGATTTCATCGATTATGAGGAGCTAAGAGAGCAATTGAGCTCAGTGTCATCATTTGAAAGGTTTGAGATATTCCCCAAGACAAGTTCATGGCCCAATCATGACTCGAACAAAGGTGTAACGGCAGCATGTCCTCATGCTGGAGCAAAAGGCTTCTACAAAAATTTAATATGGCTAGTTAAAAAAGGAAATTCATACCCAAAGCTCAGCAAATCCTACATTAATGATAAAGGGAAAGAAGTCCTCGTGCTATGGGGCATTCACCATCCATCTACTACTGCTGACCAACAAAGTCTCTATCAGAATGCAGATACATATGTTTTTGTGGGGACATCAAGATGCAGCAAGAAGTTCAAGCCGGAAATAGCAATAAGACCCAAAGTGAGGGATCAAGAAGGGAGAATGAACTATTACTGGACACTAGTAGAGCCGGGAGACAAAATAACATTCGAAGCAACTGGAAATCTAGTGGTACCGAGATATGCATTCGCAATGGAAAGAAATGCTGGATCTGGTATTATCATTTCAGATACACCAGTCCACGATTGCAATACAACTTGTCAGACACCCAAGGGTGCTATAAACACCAGCCTCCCATTTCAGAATATACATCCGATCACAATTGGAAAATGTCCAAAATATGTAAAAAGCACAAAATTGAGACTGGCCACAGGATTGAGGAATGTCCCGTCTATTCAATCTAGAGGCCTATTTGGGGCCATTGCCGGCTTCATTGAAGGGGGGTGGACAGGGATGGTAGATGGATGGTACGGTTATCACCATCAAAATGAGCAGGGGTCAGGATATGCAGCCGACCTGAAGAGCACACAGAATGCCATTGACAAGATTACTAACAAAGTAAATTCTGTTATTGAAAAGATGAATACACAGTTCACAGCAGTAGGTAAAGAGTTCAACCACCTGGAAAAAAGAATAGAGAATTTAAATAAAAAAGTTGATGATGGTTTCCTGGACATTTGGACTTACAATGCCGAACTGTTGGTTCTATTGGAAAATGAAAGAACTTTGGACTACCACGATTCAAATGTGAAGAACTTATATGAAAAGGTAAGAAACCAGTTAAAAAACAATGCCAAGGAAATTGGAAATGGCTGCTTTGAATTTTACCACAAATGCGATAACACGTGCATGGAAAGTGTCAAAAATGGGACTTATGACTACCCAAAATACTCAGAGGAAGCAAAATTAAACAGAGAAGAAATAGATGGGGTAAAACTGGAACCAACAAGGATTTACCAGATTTTGGCGATCTATTCAACTGTCGCCAGTTCATTGGTATTGGTAGTCTCCCTGGGGGCAATCAGTTTTTGGATGTGCTCTAATGGGTCTCTACAGTGTAGAATATGTATTTAA

>LC638382.1Influenza A virus H1N101701

ATGAAGGCAATACTAGTAGTTCTGCTATATACATTTGCAACCGCAAATGCAGACACATTATGTATAGGTTATCATGCGAACAATTCAACAGACACTGTAGACACAGTACTAGAAAAGAATGTAACAGTAACACACTCTGTTAATCTTCTAGAAGACAAGCATAACGGAAAACTATGCAAACTAAGAGGGGTAGCCCCATTGCATTTGGGTAAATGTAACATTGCTGGCTGGATCCTGGGAAATCCAGAGTGTGAATCACTCTCCACAGCAAGCTCATGGTCCTACATTGTGGAAACATCTAGTTCAGACAATGGAACGTGTTACCCAGGAGATTTCATCGATTATGAGGAGCTAAGAGAGCAATTGAGCTCAGTGTCATCATTTGAAAGGTTTGAGATATTCCCCAAGACAAGTTCATGGCCCAATCATGACTCGAACAAAGGTGTAACGGCAGCATGTCCTCATGCTGGAGCAAAAAGCTTCTACAAAAATTTAATATGGCTAGTTAAAAAAGGAAATTCATACCCAAAGCTCAGCAAATCCTACATTAATGATAAAGGGAAAGAAGTCCTCGTGCTATGGGGCATTCACCATCCATCTACTACTGCTGACCAACAAAGTCTCTATCAGAATGCAGATACATATGTTTTTGTGGAGACATCAAGATACAGCAAGAAGTTCAAGCCGGAAATAGCAATAAGACCCAAAGTGAGGGATCAAGAAGGGAGAATGAACTATTACTGGACACTAGTAGAACCGGGAGACAAAATAACATTCGAAGCAACTGGAAATCTAGTGGTACCGAGATATGCATTCGCAATGGAAAGAAATGCTGGATCTGGTATTATCATTTCAGATACACCAGTCCACGATTGCAATACAACTTGTCAGACACCCAAGGGTGCTATAAACACCAGCCTCCCATTTCAAAATATACATCCGATCACAATTGGAAAATGTCCAAAATATGTAAAAAGCACAAAATTGAGACTGGCCACAGGATTGAGGAATGTCCCGTCTATTCAATCTAGAGGCCTATTTGGGGCCATTGCCGGCTTCATTGAAGGGGGGTGGACAGGGATGGTAGATGGATGGTACGGTTATCACCATCAAAATGAGCAGGGGTCAGGATATGCAGCCGACCTGAAGAGCACACAGAATGCCATTGACAAGATTACTAACAAAGTAAATTCTGTTATTGAAAAGATGAATACACAGTTCACAGCAGTAGGTAAAGAGTTCAACCACCTGGAAAAAAGAATAGAGAATTTAAATAAAAAAGTTGATGATGGTTTCCTGGACATTTGGACTTACAATGCCGAACTGTTGGTTCTATTGGAAAATGAAAGAACTTTGGACTACCACGATTCAAATGTGAAGAACTTATATGAAAAAGTAAGAAACCAGTTAAAAAACAATGCCAAGGAAATTGGAAATGGCTGCTTTGAATTTTACCACAAATGCGATAACACGTGCATGGAAAGTGTCAAAAATGGGACTTATGACTACCCAAAATACTCAGAGGAAGCAAAATTAAACAGAGAAGAAATAGATGGGGTAAAGCTGGAATCAACAAGGATTTACCAGATTTTGGCGATCTATTCAACTGTCGCCAGTTCATTGGTACTGGTAGTCTCCCTGGGGGCAATCAGTTTTTGGATGTGCTCTAATGGGTCTCTACAGTGTAGAATATGTATTTAA

>LC638381.1Influenza A virus H1N101701

ATGAAGGCAATACTAGTAGTTCTGCTATATACATTTGCAACCGCAAATGCAGACACATTATGTATAGGTTATCATGCGAACAATTCAACAGACACTGTAGACACAGTACTAGAAAAGAATGTAACAGTAACACACTCTGTTAATCTTCTAGAAGACAAGCATAACGGGAAACTATGCAAACTAAGAGGGGTAGCCCCATTGCATTTGGGTAAATGTAACATTGCTGGCTGGATCCTGGGAAATCCAGAGTGTGAATCACTCTCCACAGCAAGCTCATGGTCCTACATTGTGGAAACATCTAGTTCAGACAATGGAACGTGTTACCCAGGAGATTTCATCGATTATGAGGAGCTAAGAGAGCAATTGAGCTCAGTGTCATCATTTGAAAGGTTTGAGATATTCCCCAAGACAAGTTCATGGCCCAATCATGACTCGAACAAAGGTGTAACGGCAGCATGTCCTCATGCTGGAGCAAAAAGCTTCTACAAAAATTTAATATGGCTAGTTAAAAAAGGAAATTCATACCCAAAGCTCAGCAAATCCTACATTAATGATAAAGGGAAAGAAGTCCTCGTGCTATGGGGCATTCACCATCCATCTACTACTGCTGACCAACAAAGTCTCTATCAGAATGCAGATACATATGTTTTTGTGGAGACATCAAGATACAGCAAGAAGTTCAAGCCGGAAATAGCAATAAGACCCAAAGTGAGGGATCAAGAAGGGAGAATGAACTATTACTGGACACTAGTAGAGCCGGGAGACAAAATAACATTCGAAGCAACTGGAAATCTAGTGGTACCGAGATATGCATTCGCAATGGAAAGAAATGCTGGATCTGGTATTATCATTTCAGATACACCAGTCCACGATTGCAATACAACTTGTCAGACACCCAAGGGTGCTATAAACACCAGCCTCCCATTTCAGAATATACATCCGATCACAATTGGAAAATGTCCAAAATATGTAAAAAGCACAAAATTGAGACTGGCCACAGGATTGAGGAATGTCCCGTCTATTCAATCTAGAGGCCTATTTGGGGCCATTGCCGGCTTCATTGAAGGGGGGTGGACAGGGATGGTAGATGGATGGTACGGTTATCACCATCAAAATGAGCAGGGGTCAGGATATGCAGCCGACCTGAAGAGCACACAGAATGCCATTGACAAGATTACTAACAAAGTAAATTCTGTTATTGAAAAGATGAATACACAGTTCACAGCAGTAGGTAAAGAGTTCAACCACCTGGAAAAAAGAATAGAGAATTTAAATAAAAAAGTTGATGATGGTTTCCTGGACATTTGGACTTACAATGCCGAACTGTTGGTTCTATTGGAAAATGAAAGAACTTTGGACTACCACGATTCAAATGTGAAGAACTTATATGAAAAAGTAAGAAACCAGTTAAAAAACAATGCCAAGGAAATTGGAAATGGCTGCTTTGAATTTTACCACAAATGCGATAACACGTGCATGGAAAGTGTCAAAAATGGGACTTATGACTACCCAAAATACTCAGAGGAAGCAAAATTAAACAGAGAAGAAATAGATGGGGTAAAGCTGGAATCAACAAGGATTTACCAGATTTTGGCGATCTATTCAACTGTCGCCAGTTCATTGGTACTGGTAGTCTCCCTGGGGGCAATCAGTTTTTGGATGTGCTCTAATGGGTCTCTACAGTGTAGAATATGTATTTAA

>LC638380.1Influenza A virus H1N101701

ATGAAGGCAATACTAGTAGTTCTGCTATATACATTTGCAACCGCAAATGCAGACACATTATGCATAGGTTATCATGCGAACAATTCAACAGACACTGTAGACACAGTACTAGAAAAGAATGTAACAGTAACACACTCTGTTAACCTTCTAGAAGACAAGCATAACGGGAAACTATGCAAACTAAGAGGGGTAGCCCCATTGCATTTGGGTAAATGTAACATTGCTGGCTGGATCCTGGGAAATCCAGAGTGTGAATCACTCTCCACAGCAAGCTCATGGTCCTACATTGTGGAAACATCTAGTTCAGACAATGGAACGTGTTACCCAGGAGATTTCATCAATTATGAGGAGCTAAGAGAGCAATTGAGCTCAGTGTCATCATTTGAAAGGTTTGAGATATTCCCCAAGACAAGTTCATGGCCCAATCATGACTCGAACAAAGGTGTAACGGCAGCATGTCCTCATGCTGGAGCAAAAAGCTTCTACAAAAATTTAATATGGCTAGTTAAAAAAGGAAATTCATACCCAAAGCTCAGCAAATCCTACATTAATGATAAAGGGAAAGAAGTCCTCGTGCTGTGGGGCATTCACCATCCATCTACTACTGCTGACCAACAAAGTCTCTATCAGAATGCAGATGCATATGTTTTTGTGGGGACATCAAGATACAGCAAGAAGTTCAAGCCGGAAATAGCAATAAGACCCAAAGTGAGGGATCAAGAAGGGAGAATGAACTATTACTGGACACTAGTAGAGCCGGGAGACAAAATAACATTCGAAGCAACTGGAAATCTAGTGGTACCGAGATATGCATTCGCAATGGAAAGAAATGCTGGATCTGGTATTATCATTTCAGATACACCAGTCCACGATTGCAATACAACTTGTCAGACACCCAAGGGTGCTATAAACACCAGCCTCCCATTTCAGAATATACATCCGATCACAATTGGAAAATGTCCAAAATATGTAAAAAGCACAAAATTGAGACTGGCCACAGGATTGAGGAATGTCCCGTCTATTCAATCTAGAGGCCTATTTGGGGCCATTGCCGGCTTCATTGAAGGGGGGTGGACAGGGATGGTAGATGGATGGTACGGTTATCACCATCAAAATGAGCAGGGGTCAGGATATGCAGCCGACCTGAAGAGCACACAGAATGCCATTGACAAGATTACTAACAAAGTAAATTCTGTTATTGAAAAGATGAATACACAGTTCACAGCAGTAGGTAAAGAGTTCAACCACCTGGAAAAAAGAATAGAGAATTTAAATAAAAAAGTTGATGATGGTTTCCTGGACATTTGGACTTACAATGCCGAACTGTTGGTTCTATTGGAAAATGAAAGAACTTTGGACTACCACGATTCAAATGTGAAGAACTTGTATGAAAAGGTAAGAAACCAGTTAAAAAACAATGCCAAGGAAATTGGAAACGGCTGCTTTGAATTTTACCACAAATGCGATAACACGTGCATGGAAAGTGTCAAAAATGGGACTTATGACTACCCAAAATACTCAGAGGAAGCAAAATTAAACAGAGAAGAAATAGATGGGGTAAAGCTGGAATCAACAAGGATTTACCAGATTTTGGCGATCTATTCAACTGTCGCCAGTTCATTGGTACTGGTAATCTCCCTGGGGGCAATCAGTTTCTGGATGTGCTCTAATGGGTCTCTACAGTGTAGAATATGTATTTAA

>LC638379.1Influenza A virus H1N101701

ATGAAGGCAATACTAGTAGTTCTGCTATATACATTTGCAACCGCAAATGCAGACACATTATGTATAGGTTACCATGCGAACAATTCAACAGACACTGTAGACACAGTACTAGAAAAGAATGTAACAGTAACACACTCTGTTAATCTTCTAGAAGACAAGCATAACGGGAAACTATGCAAACTAAGAGGGGTAGCCCCATTGCATTTGGGTAAATGTAACATTGCTGGCTGGATCCTGGGAAATCCAGAGTGTGAATCACTCTCCACAGCAAGCTCATGGTCCTACATTGTGGAAACATCTAGTTCAGACAATGGAACGTGTTACCCAGGAGATTTCATCGATTATGAGGAGCTAAGAGAGCAATTGAGCTCAGTGTCATCATTTGAAAGGTTTGAGATATTCCCCAAGACAAGTTCATGGCCCAATCATGACTCGAACAAAGGTGTAACGGCAGCATGTCCTCATGCTGGAGCAAAAGGCTTCTACAAAAATTTAATATGGCTAGTTAAAAAAGGAAATTCATACCCAAAGCTCAGCAAATCCTACATTAATGATAAAGGGAAAGAAGTCCTCGTGCTATGGGGCATTCACCATCCATCTACTACTGCTGACCAACAAAGTCTCTATCAGAATGCAGATACATATGTTTTTGTGGGGACATCAAGATACAGCAAGAAGTTCAAGCCGGAAATAGCAATAAGACCCAAAGTGAGGGATCAAGAAGGGAGAATGAACTATTACTGGACACTAGTAGAGCCGGGAGACAAAATAACATTCGAAGCAACTGGAAATCTAGTGGTACCGAGATATGCATTCGCAATGGAAAGAAATGCTGGATCTGGTATTATCATTTCAGATACACCAGTCCACGATTGCAATACAACTTGTCAGACACCCAAGGGTGCTATAAACACCAGCCTCCCATTTCAGAATATACATCCGATCACAATTGGAAAATGTCCAAAATATGTAAAAAGCACAAAATTGAGACTGGCCACAGGATTGAGGAATGTCCCGTCTATTCAATCTAGAGGCCTATTTGGGGCCATTGCCGGCTTCATTGAAGGGGGGTGGACAGGGATGGTAGATGGATGGTACGGTTATCACCATCAAAATGAGCAGGGGTCAGGATATGCAGCCGACCTGAAGAGCACACAGAATGCCATTGACAAGATTACTAACAAAGTAAATTCTGTTATTGAAAAGATGAATACACAGTTCACAGCAGTAGGTAAAGAGTTCAACCACCTGGAAAAAAGAATAGAGAATTTAAATAAAAAAGTTGATGATGGTTTCCTGGACATTTGGACTTACAATGCCGAACTGTTGGTTCTATTGGAAAATGAAAGAACTTTGGACTACCACGATTCAAATGTGAAGAACTTATATGAAAAGGTAAGAAACCAGTTAAAAAACAATGCCAAGGAAATTGGAAATGGCTGCTTTGAATTTTACCACAAATGCGATAACACGTGCATGGAAAGTGTCAAAAATGGGACTTATGACTACCCAAAATACTCAGAGGAAGCAAAATTAAACAGAGAAGAAATAGATGGGGTAAAACTGGAACCAACAAGGATTTACCAGATTTTGGCGATCTATTCAACTGTCGCCAGTTCATTGGTATTGGTAGTCTCCCTGGGGGCAATCAGTTTTTGGATGTGCTCTAATGGGTCTCTACAGTGTAGAATATGTATTTAA

>LC638378.1Influenza A virus H1N101701

ATGAAGGCAATACTAGTAGTTCTGCTATATACATTTGCAACCGCAAATGCAGACACATTATGTATAGGTTATCATGCGAACAATTCAACAGACACTGTAGACACAGTACTAGAAAAGAATGTAACAGTAACACACTCTGTTAATCTTCTAGAAGACAAGCATAACGGAAAACTATGCAAACTAAGAGGGGTAGCCCCATTGCATTTGGGTAAATGTAACATTGCTGGCTGGATCCTGGGAAATCCAGAGTGTGAATCACTCTCCACAGCAAGCTCATGGTCCTACATTGTGGAAACATCTAGTTCAGACAATGGAACGTGTTACCCAGGAGATTTCATCGATTATGAGGAGCTAAGAGAGCAATTGAGCTCAGTGTCATCATTTGAAAGGTTTGAGATATTCCCCAAGACAAGTTCATGGCCCAATCATGACTCGAACAAAGGTGTAACGGCAGCATGTCCTCATGCTGGAGCAAAAAGCTTCTACAAAAATTTAATATGGCTAGTTAAAAAAGGAAATTCATACCCAAAGCTCAGCAAATCCTACATTAATGATAAAGGGAAAGAAGTCCTCGTGCTATGGGGCATTCACCATCCATCTACTACTGCTGACCAACAAAGTCTCTATCAGAATGCAGATACATATGTTTTTGTGGGGACATCAAGATACAGCAAGAAGTTCAAGCCGGAAATAGCAATAAGACCCAAAGTGAGGGATCAAGAAGGGAGAATGAACTATTACTGGACACTAGTAGAGCCGGGAGACAAAATAACATTCGAAGCAACTGGAAATCTAGTGGTACCGAGATATGCATTCGCAATGGAAAGAAATGCTGGATCTGGTATTATCATTTCAGATACACCAGTCCACGATTGCAATACAACTTGTCAGACACCCAAGGGTGCTATAAACACCAGCCTCCCATTTCAAAATATACATCCGATCACAATTGGAAAATGTCCAAAATATGTAAAAAGCACAAAATTGAGACTGGCCACAGGATTGAGGAATGTCCCGTCTATTCAATCTAGAGGCCTATTTGGGGCCATTGCCGGCTTCATTGAAGGGGGGTGGACAGGGATGGTAGATGGATGGTACGGTTATCACCATCAAAATGAGCAGGGGTCAGGATATGCAGCCGACCTGAAGAGCACACAGAATGCCATTGACAAGATTACTAACAAAGTAAATTCTGTTATTGAAAAGATGAATACACAGTTCACAGCAGTAGGTAAAGAGTTCAACCACCTGGAAAAAAGAATAGAGAATTTAAATAAAAAAGTTGATGATGGTTTCCTGGACATTTGGACTTACAATGCCGAACTGTTGGTTCTATTGGAAAATGAAAGAACTTTGGACTACCACGATTCAAATGTGAAGAACTTATATGAAAAAGTAAGAAACCAGTTAAAAAACAATGCCAAGGAAATTGGAAATGGCTGCTTTGAATTTTACCACAAATGCGATAACACGTGCATGGAAAGTGTCAAAAATGGGACTTATGACTACCCAAAATACTCAGAGGAAGCAAAATTAAACAGAGAAGAAATAGATGGGGTAAAGCTGGAATCAACAAGGATTTACCAGATTTTGGCGATCTATTCAACTGTCGCCAGTTCATTGGTACTGGTAGTCTCCCTGGGGGCAATCAGTTTTTGGATGTGCTCTAATGGGTCTCTACAGTGTAGAATATGTATTTAA

>LC638377.1Influenza A virus H1N101701

ATGAAGGCAATACTAGTAGTTCTGCTATATACATTTGCAACCGCAAATGCAGACACATTATGTATAGGTTATCATGCGAACAATTCAACAGACACTGTAGACACAGTACTAGAAAAGAATGTAACAGTAACACACTCTGTTAATCTTCTAGAAGACAAGCATAACGGAAAACTATGCAAACTAAGAGGGGTAGCCCCATTGCATTTGGGTAAATGTAACATTGCTGGCTGGATCCTGGGAAATCCAGAGTGTGAATCACTCTCCACAGCAAGCTCATGGTCCTACATTGTGGAAACATCTAGTTCAGACAATGGAACGTGTTACCCAGGAGATTTCATCGATTATGAGGAGCTAAGAGAGCAATTGAGCTCAGTGTCATCATTTGAAAGGTTTGAGATATTCCCCAAGACAAGTTCATGGCCCAATCATGACTCGAACAAAGGTGTAACGGCAGCATGTCCTCATGCTGGAGCAAAAAGCTTCTACAAAAATTTAATATGGCTAGTTAAAAAAGGAAATTCATACCCAAAGCTCAGCAAATCCTACATTAATGATAAAGGGAAAGAAGTCCTCGTGCTATGGGGCATTCACCATCCATCTACTACTGCTGACCAACAAAGTCTCTATCAGAATGCAGATACATATGTTTTTGTGGGGACATCAAGATACAGCAAGAAGTTCAAGCCGGAAATAGCAATAAGACCCAAAGTGAGGGATCAAGAAGGGAGAATGAACTATTACTGGACACTAGTAGAGCCGGGAGACAAAATAACATTCGAAGCAACTGGAAATCTAGTGGTACCGAGATATGCATTCGCAATGGAAAGAAATGCTGGATCTGGTATTATCATTTCAGATACACCAGTCCACGATTGCAATACAACTTGTCAGACACCCAAGGGTGCTATAAACACCAGCCTCCCATTTCAAGATATACATCCGATCACAATTGGAAAATGTCCAAAATATGTAAAAAGCACAAAATTGAGACTGGCCACAGGATTGAGGAATGTCCCGTCTATTCAATCTAGAGGCCTATTTGGGGCCATTGCCGGCTTCATTGAAGGGGGGTGGACAGGGATGGTAGATGGATGGTACGGTTATCACCATCAAAATGAGCAGGGGTCAGGATATGCAGCCGACCTGAAGAGCACACAGAATGCCATTGACAAGATTACTAACAAAGTAAATTCTGTTATTGAAAAGATGAATACACAGTTCACAGCAGTAGGTAAAGAGTTCAACCACCTGGAAAAAAGAATAGAGAATTTAAATAAAAAAGTTGATGATGGTTTCCTGGACATTTGGACTTACAATGCCGAACTGTTGGTTCTATTGGAAAATGAAAGAACTTTGGACTACCACGATTCAAATGTGAAGAATTTATATGAAAAAGTAAGAAACCAGTTAAAAAACAATGCCAAGGAAATTGGAAATGGCTGCTTTGAATTTTACCACAAATGCGATAACACGTGCATGGAAAGTGTCAAAAATGGGACTTATGACTACCCAAAATACTCAGAGGAAGCAAAATTAAACAGAGAAAAAATAGATGGGGTAAAGCTGGAATCAACAAGGATTTACCAGATTTTGGCGATCTATTCAACTGTCGCCAGTTCATTGGTACTGGTAGTCTCCCTGGGGGCAATCAGTTTTTGGATGTGCTCTAATGGGTCTCTACAGTGTAGAATATGTATTTAA

>LC638376.1Influenza A virus H1N101701

ATGAAGGCAATACTAGTAGTTCTGCTATATACATTTGCAACCGCAAATGCAGACACATTATGTATAGGTTATCATGCGAACAATTCAACAGACACTGTAGACACAGTACTAGAAAAGAATGTAACAGTAACACACTCTGTTAATCTTCTAGAAGACAAGCATAACGGAAAACTATGCAAACTAAGAGGGGTAGCCCCATTGCATTTGGGTAAATGTAACATTGCTGGCTGGATCCTGGGAAATCCAGAGTGTGAATCACTCTCCACAGCAAGCTCATGGTCCTACATTGTGGAAACATCTAGTTCAGACAATGGAACGTGTTACCCAGGAGATTTCATCGATTATGAGGAGCTAAGAGAGCAATTGAGCTCAGTGTCATCATTTGAAAGGTTTGAGATATTCCCCAAGACAAGTTCATGGCCCAATCATGACTCGAACAAAGGTGTAACGGCAGCATGTCCTCATGCTGGAGCAAAAAGCTTCTACAAAAATTTAATATGGCTAGTTAAAAAAGGAAATTCATACCCAAAGCTCAGCAAATCCTACATTAATGATAAAGGGAAAGAAGTCCTCGTGCTATGGGGCATTCACCATCCATCTACTACTGCTGACCAACAAAGTCTCTATCAGAATGCAGATACATATGTTTTTGTGGGGACATCAAGATACAGCAAGAAGTTCAAGCCGGAAATAGCAATAAGACCCAAAGTGAGGGATCAAGAAGGGAGAATGAACTATTACTGGACACTAGTAGAGCCGGGAGACAAAATAACATTCGAAGCAACTGGAAATCTAGTGGTACCGAGATATGCATTCGCAATGGAAAGAAATGCTGGATCTGGTATTATCATTTCAGATACACCAGTCCACGATTGCAATACAACTTGTCAGACACCCAAGGGTGCTATAAACACCAGCCTCCCATTTCAAGATATACATCCGATCACAATTGGAAAATGTCCAAAATATGTAAAAAGCACAAAATTGAGACTGGCCACAGGATTGAGGAATGTCCCGTCTATTCAATCTAGAGGCCTATTTGGGGCCATTGCCGGCTTCATTGAAGGGGGGTGGACAGGGATGGTAGATGGATGGTACGGTTATCACCATCAAAATGAGCAGGGGTCAGGATATGCAGCCGACCTGAAGAGCACACAGAATGCCATTGACAAGATTACTAACAAAGTAAATTCTGTTATTGAAAAGATGAATACACAGTTCACAGCAGTAGGTAAAGAGTTCAACCACCTGGAAAAAAGAATAGAGAATTTAAATAAAAAAGTTGATGATGGTTTCCTGGACATTTGGACTTACAATGCCGAACTGTTGGTTCTATTGGAAAATGAAAGAACTTTGGACTACCACGATTCAAATGTGAAGAATTTATATGAAAAAGTAAGAAACCAGTTAAAAAACAATGCCAAGGAAATTGGAAATGGCTGCTTTGAATTTTACCACAAATGCGATAACACGTGCATGGAAAGTGTCAAAAATGGGACTTATGACTACCCAAAATACTCAGAGGAAGCAAAATTAAACAGAGAAAAAATAGATGGGGTAAAGCTGGAATCAACAAGGATTTACCAGATTTTGGCGATCTATTCAACTGTCGCCAGTTCATTGGTACTGGTAGTCTCCCTGGGGGCAATCAGTTTTTGGATGTGCTCTAATGGGTCTCTACAGTGTAGAATATGTATTTAA

>LC638373.1Influenza A virus H1N101701

ATGAAGGCAATACTAGTAGTTCTGCTGTATACATTTACAACCGCAAATGCAGACACATTATGTATAGGTTATCATGCGAACAATTCAACAGACACTGTAGACACAGTACTAGAAAAGAATGTAACAGTAACACACTCTGTCAATCTTCTGGAAGACAAGCATAACGGAAAACTATGCAAACTAAGAGGGGTATCCCCATTGCATTTGGGTAAATGTAACATTGCTGGCTGGATCCTGGGAAATCCAGAGTGTGAGTCACTCTCCACAGCAAGATCATGGTCCTACATTGTGGAAACATCTAATTCAGACAATGGGACGTGTTACCCAGGAGATTTCATCAATTATGAGGAGCTAAGAGAGCAATTGAGCTCAGTGTCATCATTTGAAAGGTTTGAAATATTCCCCAAGACAAGTTCATGGCCTAATCATGACTCGGACAAAGGTGTAACGGCAGCATGTCCTCACGCTGGAGCAAAAAGCTTCTACAAAAACTTGATATGGCTGGTTAAAAAAGGAAATTCATACCCAAAGCTCAACCAAACCTACATTAATGATAAAGGGAAAGAAGTCCTCGTGCTGTGGGGCATTCATCATCCACCTACTATTGCTGCCCAAGAAAGTCTCTATCAGAATGCAGATGCATATGTTTTTGTGGGGACATCAAGATACAGCAAGAAGTTCAAGCCGGAAATAGCAACAAGACCCAAAGTGAGGGATCAAGAAGGGAGAATGAACTATTACTGGACACTAGTAGAACCGGGAGACAAGATAACATTCGAAGCAACTGGAAATCTAGTGGTACCGAGATATGCATTCACAATGGAAAGAGATGCTGGATCTGGTATTGTCATTTCAGATACACCAGTCCACGATTGCAATACAACTTGTCAGACACCCGAGGGTGCTATAAACACCAGCCTTCCATTTCAGAATGTACATCCGATTACAATTGGGAAATGTCCAAAGTATGTAAAAAGCACAAAATTGAGACTGGCCACAGGATTGAGGAATGTCCCGTCTATTCAATCTAGAGGCCTATTCGGGGCCATTGCTGGCTTCATTGAAGGGGGGTGGACAGGGATGGTAGATGGATGGTACGGTTATCACCATCAAAATGAGCAGGGGTCAGGATATGCAGCCGATCTGAAGAGCACACAAAATGCCATTGATAAGATTACTAACAAAGTAAATTCTGTTATTGAAAAGATGAATACACAGTTCACAGCAGTGGGTAAAGAGTTCAACCACCTTGAAAAAAGAATAGAGAATCTAAATAAAAAAGTTGATGATGGTTTCCTGGACATTTGGACTTACAATGCCGAACTGTTGGTTCTACTGGAAAACGAAAGAACTTTGGACTATCACGATTCAAATGTGAAGAACTTGTATGAAAAAGTAAGAAACCAGTTAAAAAACAATGCCAAGGAAATTGGAAACGGCTGCTTTGAATTTTACCACAAATGCGACAACACATGCATGGAAAGTGTCAAGAATGGGACTTATGACTACCCAAAATACTCAGAGGAAGCAAAATTAAACAGAGAAAAAATAGATGGAGTAAAGCTGGAATCAACAAGGATCTACCAGATTTTGGCGATCTATTCAACTGTTGCCAGTTCATTGGTACTGGTAGTCTCCCTGGGGGCAATCAGCTTCTGGATGTGCTCTAATGGGTCTCTACAGTGTAGAATATGTATTTAA

>LC638372.1Influenza A virus H1N101701

ATGAAGGCAATACTAGTAGTTCTGCTGTATACATTTACAACCGCAAATGCAGACACATTATGTATAGGTTATCATGCGAACAATTCAACAGACACTGTAGACACAGTACTAGAAAAGAATGTAACAGTAACACACTCTGTCAATCTTCTGGAAGACAAGCATAACGGAAAACTATGCAAACTAAGAGGGGTATCCCCATTGCATTTGGGTAAATGTAACATTGCTGGCTGGATCCTGGGAAATCCAGAGTGTGAGTCACTCTCCACAGCAAGATCATGGTCCTACATTGTGGAAACATCTAATTCAGACAATGGAACGTGTTACCCAGGAGATTTCATCAATTATGAGGAGCTAAGAGAGCAATTGAGCTCAGTGTCATCATTTGAAAGGTTTGAAATATTCCCCAAGACAAGTTCATGGCCTAATCATGACTCGGACAAAGGTGTAACGGCAGCATGTCCTCACGCTGGAGCAAAAAGCTTCTACAAAAACTTGATATGGCTGGTTAAAAAAGGAAATTCATACCCAAAGCTCAACCAAACCTACATTAATGATAAAGGGAAAGAAGTCCTCGTGCTGTGGGGCATTCATCATCCACCTACTATTGCTGCCCAAGAAAGTCTCTATCAGAATGCAGATGCATATGTTTTTGTGGGGACATCAAGATACAGCAAGAAGTTCAAGCCGGAAATAGCAACAAGACCCAAAGTGAGGGATCAAGAAGGGAGAATGAACTATTACTGGACACTAGTAGAACCGGGAGACAAGATAACATTCGAAGCAACTGGAAATCTAGTGGTACCGAGATATGCATTCACAATGGAAAGAGATGCTGGATCTGGTATTGTCATTTCAGATACACCAGTCCACGATTGCAATACAACTTGTCAGACACCCGAGGGTGCTATAAACACCAGCCTTCCATTTCAGAATGTACATCCGATTACAATTGGGAAATGTCCAAAGTATGTAAAAAGCACAAAATTGAGACTGGCCACAGGATTGAGGAATGTCCCGTCTATTCAATCTAGAGGCCTATTCGGGGCCATTGCTGGCTTCATTGAAGGGGGGTGGACAGGGATGGTAGATGGATGGTACGGTTATCACCATCAAAATGAGCAGGGGTCAGGATATGCAGCCGATCTGAAGAGCACACAAAATGCCATTGATAAGATTACTAACAAAGTAAATTCTGTTATTGAAAAGATGAATACACAGTTCACAGCAGTGGGTAAAGAGTTCAACCACCTTGAAAAAAGAATAGAGAATCTAAATAAAAAAGTTGATGATGGTTTCCTGGACATTTGGACTTACAATGCCGAACTGTTGGTTCTACTGGAAAACGAAAGAACTTTGGACTATCACGATTCAAATGTGAAGAACTTGTATGAAAAAGTAAGAAACCAGTTAAAAAACAATGCCAAGGAAATTGGAAACGGCTGCTTTGAATTTTACCACAAATGCGACAACACATGCATGGAAAGTGTCAAGAATGGGACTTATGACTACCCAAAATACTCAGAGGAAGCAAAATTAAACAGAGAAAAAATAGATGGAGTAAAGCTGGAATCAACAAGGATCTACCAGATTTTGGCGATCTATTCAACTGTTGCCAGTTCATTGGTACTGGTAGTCTCCCTGGGGGCAATCAGCTTCTGGATGTGCTCTAATGGGTCTCTACAGTGTAGAATATGTATTTAA

>LC638371.1Influenza A virus H1N101701

ATGAAGGCAATACTAGTAGTTCTGCTGTATACATTTACAACCGCAAATGCAGACACATTATGTATAGGTTATCATGCGAACAATTCAACAGACACTGTAGACACAGTACTAGAAAAGAATGTAACAGTAACACACTCTGTCAATCTTCTGGAAGACAAGCACAACGGAAAACTATGCAAACTAAGAGGGGTAGCCCCATTGCATTTGGGTAAATGTAACATTGCTGGCTGGATCCTGGGAAATCCAGAGTGTGAATCACTCTCCACAGCAAGATCATGGTCCTACATTGTGGAAACATCTAATTCAGACAATGGAACGTGTTACCCAGGAGATTTCATCAATTATGAGGAGCTAAGAGAGCAATTGAGCTCAGTGTCATCATTTGAAAGGTTTGAAATATTCCCCAAGACAAGTTCATGGCCTAATCATGACTCGGACAAAGGTGTAACGGCAGCATGTCCTCACGCTGGAGCAAAAAGCTTCTACAAAAACTTGATATGGCTGGTTAAAAAAGGAAATTCATACCCAAAGCTCAACCAAACCTACATTAATGATAAAGGGAAAGAAGTCCTCGTGCTGTGGGGCATTCACCATCCACCTACTATTGCTGCCCAAGAAAGTCTCTATCAGAATGCAGATGCATATGTTTTTGTGGGGACATCAAGATACAGCAAGAAGTTCAAGCCGGAAATAGCAACAAGACCCAAAGTGAGGGATCAAGAAGGGAGAATGAACTATTACTGGACACTAGTAGAACCGGGAGACAAAATAACATTCGAAGCAACTGGAAATCTAGTGGTACCGAGATATGCATTCACAATGGAAAGAGATGCTGGATCTGGTATTATCATTTCAGATACACCAGTCCACGATTGCAATACAACTTGTCAGACACCCGAGGGTGCTATAAACACCAGCCTTCCATTTCAGAATGTACATCCGATTACAATTGGGAAATGTCCAAAGTATGTAAAAAGCACAAAATTGAGACTGGCCACAGGATTGAGGAATGTCCCGTCTATTCAATCTAGAGGCCTATTCGGGGCCATTGCTGGCTTCATTGAAGGGGGGTGGACAGGGATGGTAGATGGATGGTACGGTTATCACCATCAAAATGAGCAGGGGTCAGGATATGCAGCCGATCTGAAGAGCACACAAAATGCCATTGATAAGATTACTAACAAAGTAAATTCTGTTATTGAAAAGATGAATACACAGTTCACAGCAGTGGGTAAAGAGTTCAACCACCTTGAAAAAAGAATAGAGAATCTAAATAAAAAAGTTGATGATGGTTTCCTGGACATTTGGACTTACAATGCCGAACTGTTGGTTCTACTGGAAAACGAAAGAACTTTGGACTATCACGATTCAAATGTGAAGAACTTGTATGAAAAAGTAAGAAACCAGTTAAAAAACAATGCCAAGGAAATTGGAAACGGCTGCTTTGAATTTTACCACAAATGCGACAACACATGCATGGAAAGTGTCAAGAATGGGACTTATGACTACCCAAAATACTCAGAGGAAGCAAAATTAAACAGAGAAAAAATAGATGGAGTAAAGCTGGAATCAACAAGGATCTACCAGATTTTGGCGATCTATTCAACTGTTGCCAGTTCATTGGTACTGGTAGTCTCCCTGGGGGCAATCAGCTTCTGGATGTGCTCTAATGGGTCTCTACAGTGTAGAATATGCATTTAA

>LC638370.1Influenza A virus H1N101701

ATGAAGGCAATACTAGTAGTTCTGCTGTATACATTTACAACCGCAAATGCAGACACATTATGTATAGGTTATCATGCGAACAATTCAACAGACACTGTAGACACAGTACTAGAAAAGAATGTAACAGTAACACACTCTGTCAATCTTCTGGAAGACAAGCATAACGGAAAACTATGCAAACTAAGAGGAGTAGCCCCATTGCATTTGGGTAAATGTAACATTGCTGGCTGGATCCTGGGAAATCCAGAGTGTGAATCACTCTCCACAGCAAGATCATGGTCCTACATTGTGGAAACATCCAATTCAGACAATGGAACGTGTTACCCAGGAGATTTCATCAATTATGAGGAGCTAAGAGAGCAATTGAGCTCAGTGTCATCATTTGAAAGGTTTGAAATATTCCCCAAGACAAGTTCATGGCCTAATCATGACTCGGACAAAGGTGTAACGGCAGCATGTCCTCACGCTGGAGCAAAAAGCTTCTACAAAAACTTGATATGGCTGGTTAAAAAAGGAAATTCATACCCAAAGCTCAACCAAACCTACATTAATGATAAAGGGAAAGAAGTCCTCGTGCTGTGGGGCATTCACCATCCACCTACTATTGCTGCCCAAGAAAGTCTCTATCAGAATGCAGATGCATATGTTTTTGTGGGGACATCAAGATACAGCAAGAAGTTCAAGCCGGAAATAGCAACAAGACCCAAAGTGAGGGATCAAGAAGGGAGAATGAACTATTACTGGACACTAGTAGAACCGGGAGACAAAATAACATTCGAAGCAACTGGAAATCTAGTGGTACCGAGATATGCATTCACAATGGAAAGAGATGCTGGATCTGGTATTATCATTTCAGATACACCAGTCCACGATTGCAATACAACTTGTCAGACACCCGAGGGTGCTATAAACACCAGCCTTCCATTTCAGAATGTACATCCGATTACAATTGGGAAATGTCCAAAGTATGTAAAAAGCACAAAATTGAGACTGGCCACAGGATTGAGGAATGTCCCGTCTATTCAATCTAGAGGCCTATTCGGGGCCATTGCTGGCTTCATTGAAGGGGGGTGGACAGGGATGGTAGATGGATGGTACGGTTATCACCATCAAAATGAGCAGGGGTCAGGATATGCAGCCGATCTGAAGAGCACACAAAATGCCATTGATAAGATTACTAACAAAGTAAATTCTGTTATTGAAAAGATGAATACACAGTTCACAGCAGTGGGTAAAGAGTTCAACCACCTTGAAAAAAGAATAGAGAATCTAAATAAAAAAGTTGATGATGGTTTCCTGGACATTTGGACTTACAATGCCGAACTGTTGGTTCTACTGGAAAACGAAAGAACTTTGGACTATCACGATTCAAATGTGAAGAACTTGTATGAAAAAGTAAGAAACCAGTTAAAAAACAATGCCAAGGAAATTGGAAACGGCTGCTTTGAATTTTACCACAAATGCGACAACACATGCATGGAAAGTGTCAAGAATGGGACTTATGACTACCCAAAATACTCAGAGGAAGCAAAATTAAACAGAGAAAAAATAGATGGAGTAAAGCTGGAATCAACAAGGATCTACCAGATTTTGGCGATCTATTCAACTGTTGCCAGTTCATTGGTACTGGTAGTCTCCCTGGGGGCAATCAGCTTCTGGATGTGCTCTAATGGGTCTCTACAGTGTAGAATATGCATTTAA

>LC638369.1Influenza A virus H1N101701

ATGAAGGCAATACTAGTAGTTCTGCTGTATACATTTACAACCGCAAATGCAGACACATTATGTATAGGTTATCATGCAAACAATTCAACAGACACTGTAGACACAGTACTAGAAAAGAATGTAACAGTAACACACTCTGTCAATCTTCTGGAAGACAAGCATAACGGAAAACTATGCAAACTAAGAGGGGTAGCCCCATTGCATTTGGGTAAATGTAACATTGCTGGCTGGATCCTGGGAAATCCAGAGTGTGAATCACTCTCCACAGCAAGATCATGGTCCTACATTGTGGAAACATCTAATTCAGACAATGGAACGTGTTACCCAGGAGATTTCATCAATTATGAGGAGCTAAGAGAGCAATTGAGCTCAGTGTCATCATTTGAAAGGTTTGAAATATTCCCCAAGACAAGTTCATGGCCTAATCATGACTCGGACAAAGGTGTAACGGCAGCATGTCCTCACGCTGGAGCAAAAAGCTTCTACAAAAACTTGATATGGCTGGTTAAAAAAGGAAATTCATACCCAAAGCTCAACCAAACCTACATTAATGATAAAGGGAAAGAAGTCCTCGTGCTGTGGGGCATTCACCATCCACCTACTATTGCTGACCAACAAAGTCTCTATCAGAATGCAGATGCATATGTTTTTGTGGGGACATCAAGATACAGCAAGAAGTTCAAGCCGGAAATAGCAACAAGACCCAAAGTGAGGGATCAAGAAGGGAGAATGAACTATTACTGGACACTAGTAGAACCGGGAGACAAAATAACATTCGAAGCAACTGGAAATCTAGTGGTACCGAGATATGCATTCACAATGGAAAGAGATGCTGGATCTGGTATTATCATTTCAGATACACCAGTCCACGATTGCAATACAACTTGTCAGACACCCGAGGGTGCTATAAACACCAGCCTCCCATTTCAGAATGTACATCCGATCACAATTGGGAAATGTCCAAAGTATGTAAAAAGCACAAAATTGAGACTGGCCACAGGATTGAGGAATGTCCCGTCTATTCAATCTAGAGGCCTATTCGGGGCCATTGCTGGCTTCATTGAAGGGGGGTGGACAGGGATGGTAGATGGATGGTACGGTTATCACCATCAAAATGAGCAGGGGTCAGGATATGCAGCCGATCTGAAGAGCACACAAAATGCCATTGATAAGATTACTAACAAAGTAAATTCTGTTATTGAAAAGATGAATACACAGTTCACAGCAGTGGGTAAAGAGTTCAACCACCTTGAAAAAAGAATAGAGAATCTAAATAAAAAAGTTGATGATGGTTTCCTGGACATTTGGACTTACAATGCCGAACTGTTGGTTCTACTGGAAAACGAAAGAACTTTGGACTATCACGATTCAAATGTGAAGAACTTGTATGAAAAAGTAAGAAACCAGTTAAAAAACAATGCCAAGGAAATTGGAAACGGCTGCTTTGAATTTTACCACAAATGCGACAACAAATGCATGGAAAGTGTCAAGAATGGGACTTATGACTACCCAAAATACTCAGAGGAAGCAAAATTAAACAGAGAAAAAATAGATGGAGTAAGGCTGGAATCAACAAGGATCTACCAGATTTTGGCGATCTATTCAACTGTTGCCAGTTCATTGGTACTGGTAGTCTCCCTGGGGGCAATCAGCTTCTGGATGTGCTCTAATGGGTCTCTACAGTGTAGAATATGTATTTAA

>LC638368.1Influenza A virus H1N101701

ATGAAGGTAATACTAGTAGTTCTGCTGTATACATTTACAACCGCAAATGCAGACACATTATGTATAGGTTATCATGCGAACAATTCAACAGACACTGTAGACACAGTACTAGAGAAGAATGTAACAGTAACACACTCTGTTAATCTTCTGGAAGACAAGCATAACGGAAAACTATGCAAACTAAGAGGGGTAGCCCCATTGCATTTGGGTAAATGTAACATTGCTGGCTGGATCCTGGGAAATCCAGAGTGTGAATCACTCTCCACAGCAAGATCATGGTCCTACATTGTGGAAACATCTAATTCAGACAATGGAACGTGTTACCCAGGAGATTTCATCAATTATGAGGAGCTAAGAGAGCAATTGAGCTCAGTGTCATCATTTGAAAGGTTTGAAATATTCCCCAAGACAAGTTCATGGCCTAATCATGACTCGAACAAAGGTGTAACGGCAGCATGTCCTCACGCTGGAGCAAAAAGCTTCTACAAAAACTTGATATGGCTGGTTAAAAAAGGAAATTCATACCCAAAGATCAATCAAACCTACATTAATGATAAAGGGAAAGAAGTCCTCGTGCTGTGGGGCATTCACCATCCATCTACTACTGCTGACCAACAAAGTCTCTATCAGAATGCAGATGCATATGTTTTTGTGGGGACATCAAGATACAGCAAGAAGTTCAAGCCGGAAATAGCAACAAGACCCAAAGTGAGGGATCAAGAAGGGAGAATGAACTATTACTGGACACTAGTAGAGCCGGGAGACAAAATAACATTCGAAGCAACTGGAAATCTAGTGGCACCGAGATATGCATTCACAATGGAAAGAAATGCTGGATCTGGTATTATCATTTCAGATACACCAGTCCACGATTGCAATACAACTTGTCAGACACCCGAGGGTGCTATAAACACCAGCCTCCCATTTCAGAATGTACATCCGATCACAATTGGGAAATGTCCAAAGTATGTAAAAAGCACAAAATTGAGACTGGCCACAGGATTGAGGAATGTCCCGTCTATTCAATCTAGAGGCCTATTCGGGGCCATTGCCGGCTTCATTGAAGGGGGGTGGACAGGGATGGTAGATGGATGGTACGGTTATCACCATCAAAATGAGCAGGGGTCAGGATATGCAGCCGATCTGAAGAGCACACAAAATGCCATTGATAAGATTACTAACAAAGTAAATTCTGTTATTGAAAAGATGAATACACAGTTCACAGCAGTGGGTAAAGAGTTCAACCACCTTGAAAAAAGAATGGAGAATCTAAATAAAAAAGTTGATGATGGTTTCCTGGACATTTGGACTTACAATGCCGAACTGTTGGTTCTACTGGAAAATGAAAGAACTTTGGACTATCACGATTCAAATGTGAAGAACTTGTATGAAAAAGTAAGAAACCAGTTAAAAAACAATGCCAAGGAAATTGGAAACGGCTGCTTTGAATTTTACCACAAATGCGACAACACATGCATGGAAAGTGTCAAGAATGGGACTTATGACTACCCAAAATACTCAGAAGAAGCAAAATTAAACAGAGAAAAAATAGATGGAGTAAAGCTGGAATCAACAAGGATCTACCAGATTTTGGCGATCTATTCAACTGTCGCCAGTTCATTGGTACTGGTAGTCTCCCTGGGGGCAATCAGCTTCTGGATGTGCTCTAATGGGTCTCTACAGTGTAGAATATGTATTTAA

>LC638367.1Influenza A virus H1N101701

ATGAAGGCAATACTAGTAGTTCTGCTATATACATTTACAACCGCAAATGCAGACACATTATGTATAGGTTATCATGCGAACAATTCAACAGACACTGTAGACACAGTACTAGAAAAGAATGTAACAGTAACACACTCTGTTAATCTTCTGGAAGACAAGCATAACGGAAAACTATGCAAACTAAGAGGGGTAGCCCCATTGCATTTGGGTAAATGTAACATTGCTGGCTGGATCCTGGGAAATCCAGAGTGTGAATCACTCTCCACAGCAAGATCATGGTCCTACATTGTGGAAACATCTAATTCAGACAATGGAACGTGTTACCCAGGAGATTTCATCAATTATGAGGAGCTAAGAGAGCAATTGAGCTCAGTGTCATCATTTGAAAGGTTTGAAATATTCCCCAAGACAAGTTCATGGCCTAATCATGACTCGAACAAAGGTGTAACGGCAGCATGTTCTCACGCTGGAGCAAAAAGCTTCTACAAAAACTTGATATGGCTAGTTAAAAAAGGAAATTCATACCCAAAGCTCAACCAAACCTACATTAATGATAAAGGGAAAGAAGTCCTCGTGCTGTGGGGCATTCACCATCCATCTACTACTGCTGACCAACAAAGTCTCTATCAGAATGCAGATGCATATGTTTTTGTGGGGACATCAAGATACAGCAAGAAGTTCAAGCCGGAAATAGCAACAAGACCCAAAGTGAGGGATCAAGAAGGGAGAATGAACTATTACTGGACACTAGTAGAGCCGGGTGACAAAATAACATTCGAAGCAACTGGAAATCTAGTGGTACCGAGATATGCATTCACAATGGAAAGAAATGCTGGATCTGGTATTATCACTTCAGATACACCAGTTCACGATTGCAATACAACTTGTCAGACACCCGAGGGTGCTATAAACACTAGCCTCCCATTTCAGAATGTACATCCGATCACAATTGGGAAATGTCCAAAGTATGTAAAAAGCACAAAATTGAGACTGGCCACAGGATTGAGGAATGTCCCGTCTATTCAATCTAGAGGCCTATTCGGGGCCATTGCCGGCTTCATTGAAGGGGGGTGGACAGGGATGGTAGATGGATGGTACGGTTATCACCATCAAAATGAGCAGGGGTCAGGATATGCAGCCGATCTGAAGAGCACACAAAATGCCCTTGATAAGATTACTAACAAAGTAAATTCTGTTATTGAAAAGATGAATACACAGTTCACAGCAGTGGGTAAAGAGTTCAACCACCTTGAAAAAAGAATAGAGAATCTAAATAAAAAAGTTGATGATGGTTTCCTGGACATTTGGACTTACAATGCCGAACTGTTGGTTCTACTGGAAAATGAAAGAACTTTGGACTATCACGATTCAAATGTGAAGAACTTGTATGAAAAAGTAAGAAACCAGTTAAAAAACAATGCCAAGGAAATTGGAAACGGCTGCTTTGAATTTTACCACAAATGCGATAACACATGCATGGAAAGTGTCAAGAATGGGACTTATGACTACCCGAAATACTCAGAGGAAGCAAAATTAAACAGAGAAAAAATAGATGGAGTAAAGCTGGAATCAACAAGGATCTACCAGATTTTGGCGATCTATTCAACTGTCGCCAGTTCATTGGTACTGGTAGTCTCCCTGGGGGCAATCAGCTTCTGGATGTGCTCTAATGGGTCTCTACAGTGTAGAATATGTATTTAA

>LC638366.1Influenza A virus H1N101701

ATGAAGGCAATACTAGTAGTTCTGCTATATACATTTACAACCGCAAATGCAGACACATTATGTATAGGTTATCATGCGAACAATTCAACAGACACTGTAGACACAGTACTAGAAAAGAATGTAACAGTAACACACTCTGTTAATCTTCTGGAAGACAAGCATAACGGAAAATTATGCAAACTAAGAGGGGTAGCCCCATTGCATTTGGGTAAATGTAACATTGCTGGCTGGATCCTGGGAAATCCAGAGTGTGAATCACTCTCCACAGCAAGTTCATGGTCCTACATTGTGGAAACATCTAATTCAGACAATGGAACGTGTTACCCAGGAGATTTCATCAATTATGAGGAGCTAAGAGAACAATTGAGCTCAGTGTCATCATTTGAAAGGTTTGAAATATTCCCCAAGACAAGTTCATGGCCCAATCATGACTCGAACAAAGGTGTAACGGCAGCATGTCCTCACGCTGGAGCAAAAAGCTTCTACAAAAACTTGATATGGCTAGTTAAAAAAGGAAATTCATACCCAAAGCTCAACCAATCCTACATTAATGATAAAGGGAAAGAAGTCCTCGTGCTGTGGGGCATTCACCATCCATCTACTACTGCTGACCAACAAAGTCTCTATCAGAATGCAGATGCATATGTTTTTGTGGGGACATCAAGATACAGCAAGAAGTTCAAGCCGGAAATAGCAACAAGACCCAAAGTGAGGGATCAAGAAGGGAGAATGAACTATTACTGGACACTAGTAGAGCCGGGAGACAAAATAACATTCGAAGCAACTGGAAATCTAGTGGTACCGAGGTATGCATTCACAATGGAAAGAAATGCTGGATCTGGTATTATCATTTCAGATACACCAGTCCACGATTGCAATACAACTTGTCAGACACCCGAGGGTGCTATAAACACCAGCCTCCCATTTCAGAATATACATCCGATCACAATTGGGAAATGTCCAAAGTATGTAAAAAGCACAAAATTGAGACTGGCCACAGGATTGAGGAATGTCCCGTCTATTCAATCTAGAGGCCTATTCGGGGCCATTGCCGGCTTCATTGAAGGGGGGTGGACAGGGATGGTAGATGGATGGTACGGTTATCACCATCAAAATGAGCAGGGGTCAGGATATGCAGCCGACCTGAAGAGCACACAAAATGCCATTGACAAGATTACTAACAAAGTAAATTCTGTTATTGAAAAGATGAATACACAGTTCACAGCAGTGGGTAAAGAGTTCAACCACCTGGAAAAAAGAATAGAGAATCTAAATAAAAAAGTTGATGATGGATTCCTGGACATTTGGACTTACAATGCCGAACTGTTGGTTCTATTGGAAAATGAAAGAACTTTGGACTATCACGATTCAAATGTGAAGAACTTGTATGAAAAAGTAAGAAACCAGTTAAAAAACAATGCCAAGGAAATTGGAAACGGCTGCTTTGAATTTTACCACAAATGCGATAACACGTGCATGGAAAGTGTCAAAAATGGGACTTATGACTACCCAAAATACTCAGAGGAAGCAAAATTAAACAGAGAAAAAATAGATGGGGTAAAGCTGGAATCAACAAGGATCTACCAGATTTTGGCGATCTACTCAACTGTCGCCAGTTCATTGGTACTGGTAGTCTCCCTGGGGGCAATCAGCTTCTGGATGTGCTCTAATGGGTCTCTACAGTGTAGAATATGTATTTAA

>LC638365.1Influenza A virus H1N101701

ATGAAGGCAATACTAGTAGTTCTGCTATATACATTTACAACCGCAAATGCAGACACATTATGTATAGGTTATCATGCGAACAATTCAACAGACACTGTAGACACAGTACTAGAAAAGAATGTAACAGTAACACACTCTGTTAATCTTCTGGAAGACAAGCATAACGGAAAACTATGCAAACTAAGAGGGGTAGCCCCATTGCATTTGGGTAAATGTAACATTGCTGGCTGGATCCTGGGAAATCCAGAGTGTGAATCACTCTCCACAGCAAGTTCATGGTCCTACATTGTGGAAACATCTAATTCAGACAATGGAACGTGTTACCCAGGAGATTTCATCAATTATGAGGAGCTAAGAGAACAATTGAGCTCAGTGTCATCATTTGAAAGGTTTGAAATATTCCCCAAGACAAGTTCATGGCCCAATCATGACTCGAACAAAGGTGTAACGGCAGCATGTCCTCACGCTGGAGCAAAAAGCTTCTACAAAAACTTGATATGGCTAGTTAAAAAAGGAAATTCATACCCAAAGCTCAACCAATCCTACATTAATGATAAAGGGAAAGAAGTCCTCGTGCTGTGGGGCATTCACCATCCATCTACTACTGCTGACCAACAAAGTCTCTATCAGAATGCAGATGCATATGTTTTTGTGGGGACATCAAGATACAGCAAGAAGTTCAAGCCGGAAATAGCAACAAGACCCAAAGTGAGGGATCAAGAAGGGAGAATGAACTATTACTGGACACTAGTAGAGCCGGGAGACAAAATAACATTCGAAGCAACTGGAAATCTAGTGGTACCGAGGTATGCATTCACAATGGAAAGAAATGCTGGATCTGGTATTATCATTTCAGATACACCAGTCCACGATTGCAATACAACTTGTCAGACACCCGAGGGTGCTATAAACACCAGCCTCCCATTTCAGAATATACATCCGATCACAATTGGGAAATGTCCAAAGTATGTAAAAAGCACAAAATTGAGACTGGCCACAGGATTGAGGAATGTCCCGTCTATTCAATCTAGAGGCCTATTCGGGGCCATTGCCGGCTTCATTGAAGGGGGGTGGACAGGGATGGTAGATGGATGGTACGGTTATCACCATCAAAATGAGCAGGGGTCAGGATATGCAGCCGACCTGAAGAGCACACAAAATGCCATTGACAAGATTACTAACAAAGTAAATTCTGTTATTGAAAAGATGAATACACAGTTCACAGCAGTGGGTAAAGAGTTCAACCACCTGGAAAAAAGAATAGAGAATCTAAATAAAAAAGTTGATGATGGATTCCTGGACATTTGGACTTACAATGCCGAACTGTTGGTTCTATTGGAAAATGAAAGAACTTTGGACTATCACGATTCAAATGTGAAGAACTTGTATGAAAAAGTAAGAAACCAGTTAAAAAACAATGCCAAGGAAATTGGAAACGGCTGCTTTGAATTTTACCACAAATGCGATAACACGTGCATGGAAAGTGTCAAAAATGGGACTTATGACTACCCAAAATACTCAGAGGAAGCAAAATTAAACAGAGAAAAAATAGATGGGGTAAAGCTGGAATCAACAAGGATCTACCAGATTTTGGCGATCTACTCAACTGTCGCCAGTTCATTGGTACTGGTAGTCTCCCTGGGGGCAATCAGCTTCTGGATGTGCTCTAATGGGTCTCTACAGTGTAGAATATGTATTTAA

>LC638364.1Influenza A virus H1N101701

ATGAAGGCAATACTAGTAGTTCTGCTATATACATTTACAACCGCAAATGCAGACACATTATGTATAGGTTATCATGCGAACAATTCAACAGACACTGTAGACACAGTACTAGAAAAGAATGTAACAGTAACACACTCTGTTAATCTTCTGGAAGACAAGCATAACGGAAAACTATGCAAACTAAGAGGGGTAGCCCCATTGCATTTGGGTAAATGTAACATTGCTGGCTGGATCCTGGGAAATCCAGAGTGTGAATCACTCTCCACAGCAAGTTCATGGTCCTACATTGTGGAAACATCTAATTCAGACAATGGAACGTGTTACCCAGGAGATTTCATCAATTATGAGGAGCTAAGAGAACAATTGAGCTCAGTGTCATCATTTGAAAGGTTTGAAATATTCCCCAAGACAAGTTCATGGCCCAATCATGACTCGAACAAAGGTGTAACGGCAGCATGTCCTCACGCTGGAGCAAAAAGCTTCTACAAAAACTTGATATGGCTAGTTAAAAAAGGAAATTCATACCCAAAGCTCAACCAATCCTACATTAATGATAAAGGGAAAGAAGTCCTCGTGCTGTGGGGCATTCACCATCCATCTACTACTGCTGACCAACAAAGTCTCTATCAGAATGCAGATGCATATGTTTTTGTGGGGACATCAAGATACAGCAAGAAGTTCAAGCCGGAAATAGCAACAAGACCCAAAGTGAGGGATCAAGAAGGGAGAATGAACTATTACTGGACACTAGTAGAGCCAGGAGACAAAATAACATTCGAAGCAACTGGAAATCTAGTGGTACCGAGGTATGCATTCACAATGGAAAGAAATGCTGGATCTGGTATTATCATTTCAGATACACCAGTCCACGATTGCAATACAACTTGTCAGACACCCGAGGGTGCTATAAACACCAGCCTCCCATTTCAGAATATACATCCGATCACAATTGGGAAATGTCCAAAGTATGTAAAAAGCACAAAATTGAGACTGGCCACAGGATTGAGGAATGTCCCGTCTATTCAATCTAGAGGCCTATTCGGGGCCATTGCCGGCTTCATTGAAGGGGGGTGGACAGGGATGGTAGATGGATGGTACGGTTATCACCATCAAAATGAGCAGGGGTCAGGATATGCAGCCGACCTGAAGAGCACACAAAATGCCATTGACAAGATTACTAACAAAGTAAATTCTGTTATTGAAAAGATGAATACACAGTTCACAGCAGTGGGTAAAGAGTTCAACCACCTGGAAAAAAGAATAGAGAATCTAAATAAAAAAGTTGATGATGGATTCCTGGACATTTGGACTTACAATGCCGAACTGTTGGTTCTATTGGAAAATGAAAGAACTTTGGACTATCACGATTCAAATGTGAAGAACTTGTATGAAAAAGTAAGAAACCAGTTAAAAAACAATGCCAAGGAAATTGGAAACGGCTGCTTTGAATTTTACCACAAATGCGATAACACGTGCATGGAAAGTGTCAAAAATGGGACTTATGACTACCCAAAATACTCAGAGGAAGCAAAATTAAACAGAGAAAAAATAGATGGGGTAAAGCTGGAATCAACAAGGATCTACCAGATTTTGGCGATCTACTCAACTGTCGCCAGTTCATTGGTACTGGTAGTCTCCCTGGGGGCAATCAGCTTCTGGATGTGCTCTAATGGGTCTCTACAGTGTAGAATATGTATTTAA

>LC638363.1Influenza A virus H1N101701

ATGAAGGCAATACTAGTAGTTCTGCTATATACATTTGCAACCGCAAATGCAGACACATTATGTATAGGTTATCATGCGAACAATTCAACAGACACTGTAGACACAGTACTAGAAAAGAATGTAACAGTAACACACTCTGTTAACCTTCTAGAAGACAAGCATAACGGGAAACTATGCAAACTAAGAGGGGTAGCCCCATTGCATTTGGGTAAATGTAACATTGCTGGCTGGATCCTGGGAAATCCAGAGTGTGAATCACTCTCCACAGCAAGTTCATGGTCCTACATTGTGGAAACATCTAATTCAGACAATGGAACGTGTTACCCAGGAGATTTCATCAATTATGAGGAGCTAAGAGAGCAATTGAGCTCAGTGTCATCATTTGAAAGGTTTGAGATATTCCCCAAGACAAGTTCATGGCCCAATCATGACTCGAACAAAGGTGTAACGGCAGCATGTCCTCACGCTGGAGCAAAAAGCTTCTACAAAAATTTAATATGGCTAGTTAAAAAAGGAAATTCATACCCAAAGCTCAGCCAATCCTACATTAATGATAAAGGGAAAGAAGTCCTCGTGCTGTGGGGCATTCACCACCCATCTACTACTGCTGACCAACAAAGTCTCTATCAGAATGCAGATGCATATGTTTTTGTGGGGACATCAAGATACAGCAAGAAGTTCAAGCCGGAAATAGCAATAAGACCCAAAGTGAGGGATCAAGAAGGGAGAATGAACTATTACTGGACACTAGTAGAGCCGGGAGACAAAATAACATTCGAAGCAACTGGAAATCTAGTGGTACCGAGATATGCATTCACAATGGAAAGAAATGCTGGATCTGGTATTATCATTTCAGATACACCAGTCCACGATTGCAATACAACTTGTCAGACACCCGAGGGTGCTATAAACACCAGCCTCCCATTTCAGAATATACATCCGATCACAATTGGAAAATGTCCAAAGTATGTAAAAAGCACAAAATTGAGACTGGCCACAGGATTGAGGAATGTCCCGTCTATTCAATCTAGAGGCCTATTCGGGGCCATTGCCGGCTTCATTGAAGGAGGGTGGACAGGGATGGTAGATGGATGGTACGGTTATCACCATCAAAATGAGCAGGGGTCAGGATATGCAGCCGACCTGAAGAGCACACAAAATGCCATTGACAAGATTACTAACAAAGTAAATTCTGTTATTGAAAAGATGAATACACAGTTCACAGCAGTGGGTAAAGAGTTCAACCACCTGGAAAAAAGAATAGAGAATTTAAATAAAAAAGTTGATGATGGTTTCCTGGACATTTGGACTTACAATGCCGAACTGTTGGTTCTATTGGAAAATGAAAGAACTTTGGACTACCACGATTCAAATGTGAAGAACTTGTATGAAAAGGTAAGAAACCAGTTAAAAAACAATGCCAAGGAAATTGGAAACGGCTGCTTTGAATTTTACCACAAATGCGATAACACGTGCATGGAAAGTGTCAAAAATGGGACTTATGACTACCCAAAATATTCAGAGGAAGCAAAATTAAACAGAGAAAAAATAGATGGGGTAAAGCTGGAATCAACAAGGATTTACCAGATTTTGGCGATCTATTCAACTGTCGCCAGTTCATTGGTACTGGTAGTCTCCCTGGGGGCAATCAGCTTCTGGATGTGCTCTAATGGGTCTCTACAGTGTAGAATATGTATTTAA

>LC638362.1Influenza A virus H1N101701

ATGAAGGCAATACTAGTAGTTCTGCTATATACATTTGCAACCGCAAATGCAGACACATTATGTATAGGTTATCATGCGAACAATTCAACAGACACTGTAGACACAGTACTAGAAAAGAATGTAACAGTAACACACTCTGTCAATCTTCTAGAAGACAAGCATAACGGAAAACTATGCAAACTAAGAGGGGTAGCCCCATTGCATTTGGGTAAATGTAACATTGCTGGCTGGATCCTGGGAAATCCAGAGTGTGAATCACTCTCCACAGCAAGCTCATGGTCCTACATTGTGGAAACATCTAGTTCAGACAATGGAACGTGTTACCCAGGAGATTTCATCGATTATGAGGAGCTAAGAGAGCAATTGAGCTCAGTGTCATCATTTGAAAGGTTTGAGATATTCCCCAAGACAAGTTCATGGCCCAATCATGAATCGAACAAAGGTGTAACGGCAGCATGTCCTCATGCTGGAGCAAAAAGCTTCTACAAAAATTTAATATGGCTAGTTAAAAAAGGAAATTCATACCCAAAGCTCAGCAAATCCTACATTAATGATAAAGGGAAAGAAGTCCTCGTGCTATGGGGCATTCACCATCCATCTACTACTGCTGACCAACAAAGTCTCTATCAGAATGCAGATACATATGTTTTTGTGGGAACATCAAGATACAGCAAGAAGTTCAAACCGGAAATAGCAATAAGACCCAAAGTGAGGGATCAAGAAGGGAGAATGAACTATTACTGGACACTAGTAGAGCCGGGAGACAAAATAACATTCGAAGCAACTGGAAATCTAGTGGTACCGAGATATGCATTCGCAATGGAAAGAAATGCTGGATCTGGTATTATCATTTCAGATACACCAGTCCACGATTGCAATACAACTTGTCAGACACCCAAGGGTGCTATAAACACCAGCCTCCCATTTCAAGATATACATCCGATCACAATTGGAAAATGTCCAAAATATGTAAAAAGCACAAAATTGAGACTGGCCACAGGATTGAGGAATGTCCCGTCTATTCAATCTAGAGGCCTATTTGGGGCCATTGCCGGCTTCATTGAAGGGGGGTGGACAGGGATGGTAGATGGATGGTACGGTTATCACCATCAAAATGAGCAGGGGTCAGGATATGCAGCCGACCTGAAGAGCACACAGAATGCCATTGACAAGATTACTAACAAAGTAAATTCTGTTATTGAAAAGATGAATACACAGTTCACAGCAGTAGGTAAAGAGTTCAACCACCTGGAAAAAAGAATAGAGAATTTAAATAAAAAAGTTGATGATGGTTTCCTGGACATTTGGACTTACAATGCCGAACTGTTGGTTCTATTGGAAAATGAAAGAACTTTGGACTACCACGATTCAAATGTGAAGAACTTATATGAAAAAGTAAGAAACCAGTTAAAAAACAATGCCAAGGAAATTGGAAATGGCTGCTTTGAATTTTACCACAAATGCGATAACACGTGCATGGAAAGTGTCAAAAATGGGACTTATGACTACCCAAAATACTCAGAGGAAGCAAAATTAAACAGAGAAAAAATAGATGGGGTAAAGCTGGAATCAACAAGGATTTACCAGATTTTGGCGATCTATTCAACTGTCGCCAGTTCATTGGTACTGGTAGTCTCCCTGGGGGCAATCAGTTTTTGGATGTGCTCTAATGGGTCTCTACAGTGTAGAATATGTATTTAA

>LC638361.1Influenza A virus H1N101701

ATGAAGGCAATACTAGTAGTTCTGCTATATACATTTGCAACCGCAAATGCAGACACATTATGTATAGGTTATCATGCGAACAATTCAACAGACACTGTAGACACAGTACTAGAAAAGAATGTAACAGTAACACACTCTGTTAATCTTCTAGAAGACAAGCATAACGGGAAACTATGCAAACTAAGAGGGGTAGCCCCATTGCATTTGGGTAAATGTAACATTGCTGGCTGGATCCTGGGAAATCCAGAGTGTGAATCATTCTCCACAGCAAGCTCATGGTCCTACATTGTGGAAACATCTAGTTCAGACAATGGAACGTGTTACCCAGGAGATTTCATCGATTATGAGGAGCTAAGAGAGCAATTGAGCTCAGTGTCATCATTTGAAAGGTTTGAGATATTCCCCAAGACAAGTTCATGGCCCAATCATGACTCGAACAAAGGTGTAACGGCAGCATGTCCTCATGCTGGAGCAAAAAGCTTCTACAAAAATTTAATATGGCTAGTTAAAAAAGGAAATTCATACCCAAAGCTCAGCAAATCCTACATTAATGATAAAGGGAAAGAAGTCCTCGTGCTATGGGGCATTCACCATCCATCTACTACTGCTGACCAACAAAGTCTCTATCAGAATGCAGATACATATGTTTTTGTGGGGACATCAAGATACAGCAAGAAGTTCAAGCCGGAAATAGCAATAAGACCCAAAGTGAGGGATCAAGAAGGGAGAATGAACTATTACTGGACACTAGTAGAGCCGGGAGACAAAATAACATTCGAAGCAACTGGAAATCTAGTGGTACCGAGATATGCATTCGCAATGGAAAGAAATGCTGGATCTGGTATTATCATTTCAGATACACCAGTCCACGATTGCAATACAACTTGTCAGACACCCAAGGGTGCTATAAACACCAGCCTCCCATTTCAAAATATACATCCGATCACAATTGGAAAATGTCCAAAATATGTAAAAAGCACAAAATTGAGACTGGCCACAGGATTGAGGAATGTCCCGTCTATTCAATCTAGAGGCCTATTTGGGGCCATTGCCGGCTTCATTGAAGGGGGGTGGACAGGGATGGTAGATGGATGGTACGGTTATCACCATCAAAATGAGCAGGGGTCAGGATATGCAGCCGACCTGAAGAGCACACAGAATGCCATTGACAAGATTACTAACAAAGTAAATTCTGTTATTGAAAAGATGAATACACAGTTCACAGCAGTAGGTAAAGAGTTCAACCACCTGGAAAAAAGAATAGAGAATTTAAATAAAAAAGTTGATGATGGTTTCCTGGACATTTGGACTTACAATGCCGAACTGTTGGTTCTATTGGAAAATGAAAGAACTTTGGACTACCACGATTCAAATGTGAAGAACTTATATGAAAAAGTAAGAAACCAGTTAAAAAACAATGCCAAGGAAATTGGAAATGGCTGCTTTGAATTTTACCACAAATGCGATAACACGTGCATGGAAAGTGTCAAAAATGGGACTTATGACTACCCAAAATACTCAGAGGAAGCAAAATTAAACAGAGAAGAAATAGATGGGGTAAAGCTGGAATCAACAAGGATTTACCAGATTTTGGCGATCTATTCAACTGTCGCCAGTTCATTGGTACTGGTAGTCTCCCTGGGGGCAATCAGTTTTTGGATGTGCTCTAATGGGTCTCTACAGTGTAGAATATGTATTTAA

>LC638360.1Influenza A virus H1N101701

ATGAAGGCAATACTAGTAGTTCTGCTATATACATTTGCAACCGCAAATGCAGACACATTATGTATAGGTTATCATGCGAACAATTCAACAGACACTGTAGACACAGTACTAGAAAAGAATGTAACAGTAACACACTCTGTCAATCTTCTAGAAGACAAGCATAACGGAAAACTATGCAAACTAAGAGGGGTAGCCCCATTGCATTTGGGTAAATGTAACATTGCTGGCTGGATCCTGGGAAATCCAGAGTGTGAATCACTCTCCACAGCAAGCTCATGGTCCTACATTGTGGAAACATCTAGTTCAGACAATGGAACGTGTTACCCAGGAGATTTCATCGATTATGAGGAGCTAAGAGAGCAATTGAGCTCAGTGTCATCATTTGAAAGGTTTGAGATATTCCCCAAGACAAGTTCATGGCCCAATCATGACTCGAACAAAGGTGTAACGGCAGCATGTCCTCATGCTGGAGCAAAAAGCTTCTACAAAAATTTAATATGGCTAGTTAAAAAAGGAAATTCATACCCAAAGCTCAGCAAATCCTACATTAATGATAAAGGGAAAGAAGTCCTCGTGCTATGGGGCATTCACCATCCATCTACTACTGCTGACCAACAAAGTCTCTATCAGAATGCAGATACATATGTTTTTGTGGGAACATCAAGATACAGCAAGAAGTTCAAACCGGAAATAGCAATAAGACCCAAAGTGAGGGATCAAGAAGGGAGAATGAACTATTACTGGACACTAGTAGAGCCGGGAGACAAAATAACATTCGAAGCAACTGGAAATCTAGTGGTACCGAGATATGCATTCGCAATGGAAAGAAATGCTGGATCTGGTATTATCATTTCAGATACACCAGTCCACGATTGCAATACAACTTGTCAGACACCCAAGGGTGCTATAAACACCAGCCTCCCATTTCAAGATATACATCCGATCACAATTGGAAAATGTCCAAAATATGTAAAAAGCACAAAATTGAGACTGGCCACAGGATTGAGGAATGTCCCGTCTATTCAATCTAGAGGCCTATTTGGGGCCATTGCCGGCTTCATTGAAGGGGGGTGGACAGGGATGGTAGATGGATGGTACGGTTATCACCATCAAAATGAGCAGGGGTCAGGATATGCAGCCGACCTGAAGAGCACACAGAATGCCATTGACAAGATTACTAACAAAGTAAATTCTGTTATTGAAAAGATGAATACACAGTTCACAGCAGTAGGTAAAGAGTTCAACCACCTGGAAAAAAGAATAGAGAATTTAAATAAAAAAGTTGATGATGGTTTCCTGGACATTTGGACTTACAATGCCGAACTGTTGGTTCTATTGGAAAATGAAAGAACTTTGGACTACCACGATTCAAATGTGAAGAACTTATATGAAAAAGTAAGAAACCAGTTAAAAAACAATGCCAAGGAAATTGGAAATGGCTGCTTTGAATTTTACCACAAATGCGATAACACGTGCATGGAAAGTGTCAAAAATGGGACTTATGACTACCCAAAATACTCAGAGGAAGCAAAATTAAACAGAGAAAAAATAGATGGGGTAAAGCTGGAATCAACAAGGATTTACCAGATTTTGGCGATCTATTCAACTGTCGCCAGTTCATTGGTACTGGTAGTCTCCCTGGGGGCAATCAGTTTTTGGATGTGCTCTAATGGGTCTCTACAGTGTAGAATATGTATTTAA

>LC638359.1Influenza A virus H1N101701

ATGAAGGCAATACTAGTAGTTCTGCTATATACATTTGCAACCGCAAATGCAGACACATTATGTATAGGTTATCATGCGAACAATTCAACAGACACTGTAGACACAGTACTAGAAAAGAATGTAACAGTAACACACTCTGTCAATCTTCTAGAAGACAAGCATAACGGAAAACTATGCAAACTAAGAGGGGTAGCCCCATTGCATTTGGGTAAATGTAACATTGCTGGCTGGATCCTGGGAAATCCAGAGTGTGAATCACTCTCCACAGCAAGCTCATGGTCCTACATTGTGGAAACATCTAGTTCAGACAATGGAACGTGTTACCCAGGAGATTTCATCGATTATGAGGAGCTAAGAGAGCAATTGAGCTCAGTGTCATCATTTGAAAGGTTTGAGATATTCCCCAAGACAAGTTCATGGCCCAATCATGACTCGAACAAAGGTGTAACGGCAGCATGTCCTCATGCTGGAGCAAAAAGCTTCTACAAAAATTTAATATGGCTAGTTAAAAAAGGAAATTCATACCCAAAGCTCAGCAAATCCTACATTAATGATAAAGGGAAAGAAGTCCTCGTGCTATGGGGCATTCACCATCCATCTACTACTGCTGACCAACAAAGTCTCTATCAGAATGCAGATACATATGTTTTTGTGGGAACATCAAGATACAGCAAGAAGTTCAAACCGGAAATAGCAATAAGACCCAAAGTGAGGGATCAAGAAGGGAGAATGAACTATTACTGGACACTAGTAGAGCCGGGAGACAAAATAACATTCGAAGCAACTGGAAATCTAGTGGTACCGAGATATGCATTCGCAATGGAAAGAAATGCTGGATCTGGTATTATCATTTCAGATACACCAGTCCACGATTGCAATACAACTTGTCAGACACCCAAGGGTGCTATAAACACCAGCCTCCCATTTCAAGATATACATCCGATCACAATTGGAAAATGTCCAAAATATGTAAAAAGCACAAAATTGAGACTGGCCACAGGATTGAGGAATGTCCCGTCTATTCAATCTAGAGGCCTATTTGGGGCCATTGCCGGCTTCATTGAAGGGGGGTGGACAGGGATGGTAGATGGATGGTACGGTTATCACCATCAAAATGAGCAGGGGTCAGGATATGCAGCCGACCTGAAGAGCACACAGAATGCCATTGACAAGATTACTAACAAAGTAAATTCTGTTATTGAAAAGATGAATACACAGTTCACAGCAGTAGGTAAAGAGTTCAACCACCTGGAAAAAAGAATAGAGAATTTAAATAAAAAAGTTGATGATGGTTTCCTGGACATTTGGACTTACAATGCCGAACTGTTGGTTCTATTGGAAAATGAAAGAACTTTGGACTACCACGATTCAAATGTGAAGAACTTATATGAAAAAGTAAGAAACCAGTTAAAAAACAATGCCAAGGAAATTGGAAATGGCTGCTTTGAATTTTACCACAAATGCGATAACACGTGCATGGAAAGTGTCAAAAATGGGACTTATGACTACCCAAAATACTCAGAGGAAGCAAAATTAAACAGAGAAAAAATAGATGGGGTAAAGCTGGAATCAACAAGGATTTACCAGATTTTGGCGATCTATTCAACTGTCGCCAGTTCATTGGTACTGGTAGTCTCCCTGGGGGCAATCAGTTTTTGGATGTGCTCTAATGGGTCTCTACAGTGTAGAATATGTATTTAA

>LC638358.1Influenza A virus H1N101701

ATGAAGGCAATACTAGTAGTTCTGCTATATACATTTGCAACCGCAAATGCAGACACATTATGTATAGGTTATCATGCGAACAATTCAACAGACACTGTAGACACAGTACTAGAAAAGAATGTAACAGTAACACACTCTGTCAATCTTCTAGAAGACAAGCATAACGGAAAACTATGCAAACTAAGAGGGGTAGCCCCATTGCATTTGGGTAAATGTAACATTGCTGGCTGGATCCTGGGAAATCCAGAGTGTGAATCACTCTCCACAGCAAGCTCATGGTCCTACATTGTGGAAACATCTAGTTCAGACAATGGAACGTGTTACCCAGGAGATTTCATCGATTATGAGGAGCTAAGAGAGCAATTGAGCTCAGTGTCATCATTTGAAAGGTTTGAGATATTCCCCAAGACAAGTTCATGGCCCAATCATGACTCGAACAAAGGTGTAACGGCAGCATGTCCTCATGCTGGAGCAAAAAGCTTCTACAAAAATTTAATATGGCTAGTTAAAAAAGGAAATTCATACCCAAAGCTCAGCAAATCCTACATTAATGATAAAGGGAAAGAAGTCCTCGTGCTATGGGGCATTCACCATCCATCTACTACTGCTGACCAACAAAGTCTCTATCAGAATGCAGATACATATGTTTTTGTGGGAACATCAAGATACAGCAAGAAGTTCAAACCGGAAATAGCAATAAGACCCAAAGTGAGGGATCAAGAAGGGAGAATGAACTATTACTGGACACTAGTAGAGCCGGGAGACAAAATAACATTCGAAGCAACTGGAAATCTAGTGGTACCGAGATATGCATTCGCAATGGAAAGAAATGCTGGATCTGGTATTATCATTTCAGATACACCAGTCCACGATTGCAATACAACTTGTCAGACACCCAAGGGTGCTATAAACACCAGCCTCCCATTTCAAGATATACATCCGATCACAATTGGAAAATGTCCAAAATATGTAAAAAGCACAAAATTGAGACTGGCCACAGGATTGAGGAATGTCCCGTCTATTCAATCTAGAGGCCTATTTGGGGCCATTGCCGGCTTCATTGAAGGGGGGTGGACAGGGATGGTAGATGGATGGTACGGTTATCACCATCAAAATGAGCAGGGGTCAGGATATGCAGCCGACCTGAAGAGCACACAGAATGCCATTGACAAGATTACTAACAAAGTAAATTCTGTTATTGAAAAGATGAATACACAGTTCACAGCAGTAGGTAAAGAGTTCAACCACCTGGAAAAAAGAATAGAGAATTTAAATAAAAAAGTTGATGATGGTTTCCTGGACATTTGGACTTACAATGCCGAACTGTTGGTTCTATTGGAAAATGAAAGAACTTTGGACTACCACGATTCAAATGTGAAGAACTTATATGAAAAAGTAAGAAACCAGTTAAAAAACAATGCCAAGGAAATTGGAAATGGCTGCTTTGAATTTTACCACAAATGCGATAACACGTGCATGGAAAGTGTCAAAAATGGGACTTATGACTACCCAAAATACTCAGAGGAAGCAAAATTAAACAGAGAAAAAATAGATGGGGTAAAGCTGGAATCAACAAGGATTTACCAGATTTTGGCGATCTATTCAACTGTCGCCAGTTCATTGGTACTGGTAGTCTCCCTGGGGGCAATCAGTTTTTGGATGTGCTCTAATGGGTCTCTACAGTGTAAAATATGTATTTAA

>LC638357.1Influenza A virus H1N101701

ATGAAGGCAATACTAGTAGTTCTGCTATATACATTTGCAACCGCAAATGCAGACACATTATGTATAGGTTATCATGCGAACAATTCAACAGACACTGTAGACACAGTACTAGAAAAGAATGTAACAGTAACACACTCTGTCAATCTTCTAGAAGACAAGCATAACGGAAAACTATGCAAACTAAGAGGGGTAGCCCCATTGCATTTGGGTAAATGTAACATTGCTGGCTGGATCCTGGGAAATCCAGAGTGTGAATCACTCTCCACAGCAAGCTCATGGTCCTACATTGTGGAAACATCTAGTTCAGACAATGGAACGTGTTACCCAGGAGATTTCATCGATTATGAGGAGCTAAGAGAGCAATTGAGCTCAGTGTCATCATTTGAAAGGTTTGAGATATTCCCCAAGACAAGTTCATGGCCCAATCATGACTCGAACAAAGGTGTAACGGCAGCATGTCCTCATGCTGGAGCAAAAAGCTTCTACAAAAATTTAATATGGCTAGTTAAAAAAGGAAATTCATACCCAAAGCTCAGCAAATCCTACATTAATGATAAAGGGAAAGAAGTCCTCGTGCTATGGGGCATTCACCATCCATCTACTACTGCTGACCAACAAAGTCTCTATCAGAATGCAGATGCATATGTTTTTGTGGGAACATCAAGATACAGCAAGAAGTTCAAACCGGAAATAGCAATAAGACCCAAAGTGAGGGATCAAGAAGGGAGAATGAACTATTACTGGACACTAGTAGAGCCGGGAGACAAAATAACATTCGAAGCAACTGGAAATCTAGTGGTACCGAGATATGCATTCGCAATGGAAAGAAATGCTGGATCTGGTATTATCATTTCAGATACACCAGTCCACGATTGCAATACAACTTGTCAGACACCCAAGGGTGCTATAAACACCAGCCTCCCATTCCAAGATATACATCCGATCACAATTGGAAAATGTCCAAAATATGTAAAAAGCACAAAATTGAGACTGGCCACAGGATTGAGGAATGTCCCGTCTATTCAATCTAGAGGCCTATTTGGGGCCATTGCCGGCTTCATTGAAGGGGGGTGGACAGGGATGGTAGATGGATGGTACGGTTATCACCATCAAAATGAGCAGGGGTCAGGATATGCAGCCGACCTGAAGAGCACACAGAATGCCATTGACAAGATTACTAACAAAGTAAATTCTGTTATTGAAAAGATGAATACACAGTTCACAGCAGTAGGTAAAGAGTTCAACCACCTGGAAAAAAGAATAGAGAATTTAAATAAAAAAGTTGATGATGGTTTCCTGGACATTTGGACTTACAATGCCGAACTGTTGGTTCTATTGGAAAATGAAAGAACTTTGGACTACCACGATTCAAATGTGAAGAACTTATATGAAAAAGTAAGAAACCAGTTAAAAAACAATGCCAAGGAAATTGGAAATGGCTGCTTTGAATTTTACCACAAATGCGATAACACGTGCATGGAAAGTGTCAAAAATGGGACTTATGACTACCCAAAATACTCAGAGGAAGCAAAATTAAACAGAGAAAAAATAGATGGGGTAAAGCTGGAATCAACAAGGATTTACCAGATTTTGGCGATCTATTCAACTGTCGCCAGTTCATTGGTACTGGTAGTCTCCCTGGGGGCAATCAGTTTTTGGATGTGCTCTAATGGGTCTCTACAGTGTAGAATATGTATTTAA

>LC638356.1Influenza A virus H1N101701

ATGAAGGCAATACTAGTAGTTCTGCTATATACATTTGCAACCGCAAATGCAGACACATTATGTATAGGTTATCATGCGAACAATTCAACAGACACTGTAGACACAGTACTAGAAAAGAATGTAACAGTAACACACTCTGTCAATCTTCTAGAAGACAAGCATAACGGAAAACTATGCAAACTAAGAGGGGTAGCCCCATTGCATTTGGGTAAATGTAACATTGCTGGCTGGATCCTGGGAAATCCAGAGTGTGAATCACTCTCCACAGCAAGCTCATGGTCCTACATTGTGGAAACATCTAGTTCAGACAATGGAACGTGTTACCCAGGAGATTTCATCGATTATGATGAGCTAAGAGAGCAATTGAGCTCAGTGTCATCATTTGAAAGGTTTGAGATATTCCCCAAGACAAGTTCATGGCCCAATCATGACTCGAACAAAGGTGTAACGGCAGCATGTCCTCATGCTGGAGCAAAAAGCTTCTACAAAAATTTAATATGGCTAGTTAAAAAAGGAAATTCATACCCAAAGCTCAGCAAATCCTACATTAATGATAAAGGGAAAGAAGTCCTCGTGCTATGGGGCATTCACCATCCATCTACTACTGCTGACCAACAAAGTCTCTATCAGAATGCAGATACATATGTTTTTGTGGGAACATCAAGATACAGCAAGAAGTTCAAACCGGAAATAGCAATAAGACCCAAAGTGAGGGATCAAGAAGGGAGAATGAACTATTACTGGACACTAGTAGAGCCGGGAGACAAAATAACATTCGAAGCAACTGGAAATCTAGTGGTACCGAGATATGCATTCGCAATGGAAAGAAATGCTGGATCTGGTATTATCATTTCAGATACACCAGTCCACGATTGCAATACAACTTGTCAGACACCCAAGGGTGCTATAAACACCAGCCTCCCATTTCAAGATATACATCCGATCACAATTGGAAAATGTCCAAAATATGTAAAAAGCACAAAATTGAGACTGGCCACAGGATTGAGGAATGTCCCGTCTATTCAATCTAGAGGCCTATTTGGGGCCATTGCCGGCTTCATTGAAGGGGGGTGGACAGGGATGGTAGATGGATGGTACGGTTATCACCATCAAAATGAGCAGGGGTCAGGATATGCAGCCGACCTGAAGAGCACACAGAATGCCATTGACAAGATTACTAACAAAGTAAATTCTGTTATTGAAAAGATGAATACACAGTTCACAGCAGTAGGTAAAGAGTTCAACCACCTGGAAAAAAGAATAGAGAATTTAAATAAAAAAGTTGATGATGGTTTCCTGGACATTTGGACTTACAATGCCGAACTGTTGGTTCTATTGGAAAATGAAAGAACTTTGGACTACCACGATTCAAATGTGAAGAACTTATATGAAAAAGTAAGAAACCAGTTAAAAAACAATGCCAAGGAAATTGGAAATGGCTGCTTTGAATTTTACCACAAATGCGATAACACGTGCATGGAAAGTGTCAAAAATGGGACTTATGACTACCCAAAATACTCAGAGGAAGCAAAATTAAACAGAGAAAAAATAGATGGGGTAAAGCTGGAATCAACAAGGATTTACCAGATTTTGGCGATCTATTCAACTGTCGCCAGTTCATTGGTACTGGTAGTCTCCCTGGGGGCAATCAGTTTTTGGATGTGCTCTAATGGGTCTCTACAGTGTAGAATATGTATTTAA

>LC638355.1Influenza A virus H1N101701

ATGAAGGCAATACTAGTAGTTCTGCTATATACATTTGCAACCGCAAATGCAGACACATTATGTATAGGTTATCATGCGAACAATTCAACAGACACTGTAGACACAGTACTAGAAAAGAATGTAACAGTAACACACTCTGTTAATCTTCTAGAAGACAAGCATAACGGAAAACTATGCAAACTAAGAGGGGTAGCCCCATTGCATTTGGGTAAATGTAACATTGCTGGCTGGATCCTGGGAAATCCAGAGTGTGAATCACTCTCCACAGCAAGTTCATGGTCCTACATTGTGGAAACATCTAGTTCAGACAATGGAACGTGTTACCCAGGAGATTTCATCGATTATGAGGAGCTAAGAGAGCAATTGAGCTCAGTGTCATCATTTGAAAGGTTTGAGATATTCCCCAAGACAAGTTCATGGCCCAATCATGACTCGAACAAAGGTGTAACGGCAGCATGTCCTCATGCTGGAGCAAAAAGCTTCTACAAAAATTTAATATGGCTAGTTAAAAAAGGAAATTCATACCCAAAGCTCAGCAAATCCTACATTAATGATAAAGGGAAAGAAGTCCTCGTGCTATGGGGCATTCACCATCCATCTACTACTGCTGACCAACAAAGTCTCTATCAGAATGCAGATACATATGTTTTTGTGGGGACATCAAGATACAGCAAGAAGTTCAAGCCGGAAATAGCAATAAGACCCAAAGTGAGGGATCAAGAAGGGAGAATGAACTATTACTGGACACTAGTAGAGCCGGGAGACAAAATAACATTCGAAGCAACTGGAAATCTAGTGGTACCGAGATATGCATTCGCAATGGAAAGAAATGCTGGATCTGGTATTATCATTTCAGATACACCAGTCCACGATTGCAATACAACTTGTCAGACACCCAAGGGTGCTATAAACACCAGCCTCCCATTTCAAGATATACATCCGATCACAATTGGAAAATGTCCAAAATATGTAAAAAGCACAAAATTGAGACTGGCCACAGGATTGAGGAATGTCCCGTCTATTCAATCTAGAGGCCTATTTGGGGCCATTGCCGGCTTCATTGAAGGGGGGTGGACAGGGATGGTAGATGGATGGTACGGTTATCACCATCAAAATGAGCAGGGGTCAGGATATGCAGCCGACCTGAAGAGCACACAGAATGCCATTGACAAGATTACTAACAAAGTAAATTCTGTTATTGAAAAGATGAATACACAGTTCACAGCAGTAGGTAAAGAGTTCAACCACCTGGAAAAAAGAATAGAGAATTTAAATAAAAAAGTTGATGATGGTTTCCTGGACATTTGGACTTACAATGCCGAACTGTTGGTTCTATTGGAAAATGAAAGAACTTTGGACTACCACGATTCAAATGTGAAGAATTTATATGAAAAAGTAAGAAACCAGTTAAAAAACAATGCCAAGGAAATTGGAAATGGCTGCTTTGAATTTTACCACAAATGCGATAACACGTGCATGGAAAGTGTCAAAAATGGGACTTATGATTACCCAAAATACTCAGAGGAAGCAAAATTAAACAGAGAAAAAATAGATGGGGTAAAGCTGGAATCAACAAGGATTTACCAGATTTTGGCGATCTATTCAACTGTCGCCAGTTCATTGGTACTGGTAGTCTCCCTGGGGGCAATCAGTTTTTGGATGTGCTCTAATGGGTCTCTACAGTGTAGAATATGTATTTAA

>LC638354.1Influenza A virus H1N101701

ATGAAGGCAATACTAGTAGTTCTGCTATATACATTTGCAACCGCAAATGCAGACACATTATGTATAGGTTATCATGCGAACAATTCAACAGACACTGTAGACACAGTACTAGAAAAGAATGTAACAGTAACACACTCTGTCAATCTTCTAGAAGACAAGCATAACGGAAAACTATGCAAACTAAGAGGGGTAGCCCCATTGCATTTGGGTAAATGTAACATTGCTGGCTGGATCCTGGGAAATCCAGAGTGTGAATCACTCTCCACAGCAAGCTCATGGTCCTACATTGTGGAAACATCTAGTTCAGACAATGGAACGTGTTACCCAGGAGATTTCATCGATTATGAGGAGCTAAGAGAGCAATTGAGCTCAGTGTCATCATTTGAAAGGTTTGAGATATTCCCCAAGACAAGTTCATGGCCCAATCATGAATCGAACAAAGGTGTAACGGCAGCATGTCCTCATGCTGGAGCAAAAAGCTTCTACAAAAATTTAATATGGCTAGTTAAAAAAGGAAATTCATACCCAAAGCTCAGCAAATCCTACATTAATGATAAAGGGAAAGAAGTCCTCGTGCTATGGGGCATTCACCATCCATCTACTACTGCTGACCAACAAAGTCTCTATCAGAATGCAGATACATATGTTTTTGTGGGAACATCAAGATACAGCAAGAAGTTCAAACCGGAAATAGCAATAAGACCCAAAGTGAGGGATCAAGAAGGGAGAATGAACTATTACTGGACACTAGTAGAGCCGGGAGACAAAATAACATTCGAAGCAACTGGAAATCTAGTGGTACCGAGATATGCATTCGCAATGGAAAGAAATGCTGGATCTGGTATTATCATTTCAGATACACCAGTCCACGATTGCAATACAACTTGTCAGACACCCAAGGGTGCTATAAACACCAGCCTCCCATTTCAAGATATACATCCGATCACAATTGGAAAATGTCCAAAATATGTAAAAAGCACAAAATTGAGACTGGCCACAGGATTGAGGAATGTCCCGTCTATTCAATCTAGAGGCCTATTTGGGGCCATTGCCGGCTTCATTGAAGGGGGGTGGACAGGGATGGTAGATGGATGGTACGGTTATCACCATCAAAATGAGCAGGGGTCAGGATATGCAGCCGACCTGAAGAGCACACAGAATGCCATTGACAAGATTACTAACAAAGTAAATTCTGTTATTGAAAAGATGAATACACAGTTCACAGCAGTAGGTAAAGAGTTCAACCACCTGGAAAAAAGAATAGAGAATTTAAATAAAAAAGTTGATGATGGTTTCCTGGACATTTGGACTTACAATGCCGAACTGTTGGTTCTATTGGAAAATGAAAGAACTTTGGACTACCACGATTCAAATGTGAAGAACTTATATGAAAAAGTAAGAAACCAGTTAAAAAACAATGCCAAGGAAATTGGAAATGGCTGCTTTGAATTTTACCACAAATGCGATAACACGTGCATGGAAAGTGTCAAAAATGGGACTTATGACTACCCAAAATACTCAGAGGAAGCAAAATTAAACAGAAAAAAAATAGATGGGGTAAAGCTGGAATCAACAAGGATTTACCAGATTTTGGCGATCTATTCAACTGTCGCCAGTTCATTGGTACTGGTAGTCTCCCTGGGGGCAATCAGTTTTTGGATGTGCTCTAATGGGTCTCTACAGTGTAGAATATGTATTTAA

>LC638353.1Influenza A virus H1N101701

ATGAAGGCAATACTAGTAGTTCTGCTATATACATTTGCAACCGCAAATGCAGACACATTATGTATAGGTTATCATGCGAACAATTCAACAGACACTGTAGACACAGTACTAGAAAAGAATGTAACAGTAACACACTCTGTCAATCTTCTAGAAGACAAGCATAACGGAAAACTATGCAAACTAAGAGGGGTAGCCCCATTGCATTTGGGTAAATGTAACATTGCTGGCTGGATCCTGGGAAATCCAGAGTGTGAATCACTCTCCACAGCAAGCTCATGGTCCTACATTGTGGAAACATCTAGTTCAGACAATGGAACGTGTTACCCAGGAGATTTCATCGATTATGAGGAGCTAAGAGAGCAATTGAGCTCAGTGTCATCATTTGAAAGGTTTGAGATATTCCCCAAGACAAGTTCATGGCCCAATCATGAATCGAACAAAGGTGTAACGGCAGCATGTCCTCATGCTGGAGCAAAAAGCTTCTACAAAAATTTAATATGGCTAGTTAAAAAAGGAAATTCATACCCAAAGCTCAGCAAATCCTACATTAATGATAAAGGGAAAGAAGTCCTCGTGCTATGGGGCATTCACCATCCATCTACTACTGCTGACCAACAAAGTCTCTATCAGAATGCAGATACATATGTTTTTGTGGGAACATCAAGATACAGCAAGAAGTTCAAACCGGAAATAGCAATAAGACCCAAAGTGAGGGATCAAGAAGGGAGAATGAACTATTACTGGACACTAGTAGAGCCGGGAGACAAAATAACATTCGAAGCAACTGGAAATCTAGTGGTACCGAGATATGCATTCGCAATGGAAAGAAATGCTGGATCTGGTATTATCATTTCAGATACACCAGTCCACGATTGCAATACAACTTGTCAGACACCCAAGGGTGCTATAAACACCAGCCTCCCATTTCAAGATATACATCCGATCACAATTGGAAAATGTCCAAAATATGTAAAAAGCACAAAATTGAGACTGGCCACAGGATTGAGGAATGTCCCGTCTATTCAATCTAGAGGCCTATTTGGGGCCATTGCCGGCTTCATTGAAGGGGGGTGGACAGGGATGGTAGATGGATGGTACGGTTATCACCATCAAAATGAGCAGGGGTCAGGATATGCAGCCGACCTGAAGAGCACACAGAATGCCATTGACAAGATTACTAACAAAGTAAATTCTGTTATTGAAAAGATGAATACACAGTTCACAGCAGTAGGTAAAGAGTTCAACCACCTGGAAAAAAGAATAGAGAATTTAAATAAAAAAGTTGATGATGGTTTCCTGGACATTTGGACTTACAATGCCGAACTGTTGGTTCTATTGGAAAATGAAAGAACTTTGGACTACCACGATTCAAATGTGAAGAACTTATATGAAAAAGTAAGAAACCAGTTAAAAAACAATGCCAAGGAAATTGGAAATGGCTGCTTTGAATTTTACCACAAATGCGATAACACGTGCATGGAAAGTGTCAAAAATGGGACTTATGACTACCCAAAATACTCAGAGGAAGCAAAATTAAACAGAGAAAAAATAGATGGGGTAAAGCTGGAATCAACAAGGATTTACCAGATTTTGGCGATCTATTCAACTGTCGCCAGTTCATTGGTACTGGTAGTCTCCCTGGGGGCAATCAGTTTTTGGATGTGCTCTAATGGGTCTCTACAGTGTAAAATATGTATTTAA

>LC638352.1Influenza A virus H1N101701

ATGAAGGCAATACTAGTAGTTCTGCTATATACATTTGCAACCGCAAATGCAGACACATTATGTATAGGTTATCATGCGAACAATTCAACAGACACTGTAGACACAGTACTAGAAAAGAATGTAACAGTAACACACTCTGTCAATCTTCTAGAAGACAAGCATAACGGAAAACTATGCAAACTAAGAGGGGTAGCCCCATTGCATTTGGGTAAATGTAACATTGCTGGCTGGATCCTGGGAAATCCAGAGTGTGAATCACTCTCCACAGCAAGCTCATGGTCCTACATTGTGGAAACATCTAGTTCAGACAATGGAACGTGTTACCCAGGAGATTTCATCGATTATGAGGAGCTAAGAGAGCAATTGAGCTCAGTGTCATCATTTGAAAGGTTTGAGATATTCCCCAAGACAAGTTCATGGCCCAATCATGACTCGAACAAAGGTGTAACGGCAGCATGTCCTCATGCTGGAGCAAAAAGCTTCTACAAAAATTTAATATGGCTAGTTAAAAAAGGAAATTCATACCCAAAGCTCAGCAAATCCTACATTAATGATAAAGGGAAAGAAGTCCTCGTGCTATGGGGCATTCACCATCCATCTACTACTGCTGACCAACAAAGTCTCTATCAGAATGCAGATACATATGTTTTTGTGGGAACATCAAGATACAGCAAGAAGTTCAAACCGGAAATAGCAATAAGACCCAAAGTGAGGGATCAAGAAGGGAGAATGAACTATTACTGGACACTAGTAGAGCCGGGAGACAAAATAACATTCGAAGCAACTGGAAATCTAGTGGTACCGAGATATGCATTCGCAATGGAAAGAAATGCTGGATCTGGTATTATCATTTCAGATACACCAGTCCACGATTGCAATACAACTTGTCAGACACCCAAGGGTGCTATAAACACCAGCCTCCCATTTCAAGATATACATCCGATCACAATTGGAAAATGTCCAAAATATGTAAAAAGCACAAAATTGAGACTGGCCACAGGATTGAGGAATGTCCCGTCTATTCAATCTAGAGGCCTATTTGGGGCCATTGCCGGCTTCATTGAAGGGGGGTGGACAGGGATGGTAGATGGATGGTACGGTTATCACCATCAAAATGAGCAGGGGTCAGGATATGCAGCCGACCTGAAGAGCACACAGAATGCCATTGACAAGATTACTAACAAAGTAAATTCTGTTATTGAAAAGATGAATACACAGTTCACAGCAGTAGGTAAAGAGTTCAACCACCTGGAAAAAAGAATAGAGAATTTAAATAAAAAAGTTGATGATGGTTTCCTGGACATTTGGACTTACAATGCCGAACTGTTGGTTCGATTGGAAAATGAAAGAACTTTGGACTACCACGATTCAAATGTGAAGAACTTATATGAAAAAGTAAGAAACCAGTTAAAAAACAATGCCAAGGAAATTGGAAATGGCTGCTTTGAATTTTACCACAAATGCGATAACACGTGCATGGAAAGTGTCAAAAATGGGACTTATGACTACCCAAAATACTCAGAGGAAGCAAAATTAAACAGAGAAAAAATAGATGGGGTAAAGCTGGAATCAACAAGGATTTACCAAATTTTGGCGATCTATTCAACTGTCGCCAGTTCATTGGTACTGGTAGTCTCCCTGGGGGCAATCAGTTTTTGGATGTGCTCTAATGGGTCTCTACAGTGTAAAATATGTATTTAA

>LC638351.1Influenza A virus H1N101701

ATGAAGGCAATACTAGTAGTTCTGCTATATACATTTGCAACCGCAAATGCAGACACATTATGTATAGGTTATCATGCGAACAATTCAACAGACACTGTAGACACAGTACTAGAAAAGAATGTAACAGTAACACACTCTGTCAATCTTCTAGAAGACAAGCATAACGGAAAACTATGCAAACTAAGAGGGGTAGCCCCATTGCATTTGGGTAAATGTAACATTGCTGGCTGGATCCTGGGAAATCCAGAGTGTGAATCACTCTCCACAGCAAGCTCATGGTCCTACATTGTGGAAACATCTAGTTCAGACAATGGAACGTGTTACCCAGGAGATTTCATCGATTATGAGGAGCTAAGAGAGCAATTGAGCTCAGTGTCATCATTTGAAAGGTTTGAGATATTCCCCAAGACAAGTTCGTGGCCCAATCATGACTCGAACAAAGGTGTAACGGCAGCATGTCCTCATGCTGGAGCAAAAAGCTTCTACAAAAATTTAATATGGCTAGTTAAAAAAGGAAATTCATACCCAAAGCTCAGCAAATCCTACATTAATGATAAAGGGAAAGAAGTCCTCGTGCTATGGGGCATTCACCATCCATCTACTACTGCTGACCAACAAAGTCTCTATCAGAATGCAGATGCATATGTTTTTGTGGGAACATCAAGATACAGCAAGAAGTTCAAACCGGAAATAGCAATAAGACCCAAAGTGAGGGATCAAGAAGGGAGAATGAACTATTACTGGACACTAGTAGAGCCGGGAGACAAAATAACATTCGAAGCAACTGGAAATCTAGTGGTACCGAGATATGCATTCGCAATGGAAAGAAATGCTGGATCTGGTATTATCATTTCAGATACACCAGTCCACGATTGCAATACAACTTGTCAGACACCCAAGGGTGCTATAAACACCAGCCTCCCATTTCAAGATATACATCCGATCACAATTGGAAAATGTCCAAAATATGTAAAAAGCACAAAATTGAGACTGGCCACAGGATTGAGGAATGTCCCGTCTATTCAATCTAGAGGCCTATTTGGGGCCATTGCCGGCTTCATTGAAGGGGGGTGGACAGGGATGGTAGATGGATGGTACGGTTATCACCATCAAAATGAGCAGGGGTCAGGATATGCAGCCGACCTGAAGAGCACACAGAATGCCATTGACAAGATTACTAACAAAGTAAATTCTGTTATTGAAAAGATGAATACACAGTTCACAGCAGTAGGTAAAGAGTTCAACCACCTGGAAAAAAGAATAGAGAATTTAAATAAAAAAGTTGATGATGGTTTCCTGGACATTTGGACTTACAATGCCGAACTGTTGGTTCGATTGGAAAATGAAAGAACTTTGGACTACCACGATTCAAATGTGAAGAACTTATATGAAAAAGTAAGAAACCAGTTAAAAAACAATGCCAAGGAAATTGGAAATGGCTGCTTTGAATTTTACCACAAATGCGATAACACGTGCATGGAAAGTGTCAAAAATGGGACTTATGACTACCCAAAATACTCAGAGGAAGCAAAATTAAACAGAGAAAAAATAGATGGGGTAAAGCTGGAATCAACAAGGATTTACCAGATTTTGGCGATCTATTCAACTGTCGCCAGTTCATTGGTACTGGTAGTCTCCCTGGGGGCAATCAGTTTTTGGATGTGCTCTAATGGGTCTCTACAGTGTAGAATATGTATTTAA

>LC638350.1Influenza A virus H1N101701

ATGAAGGCAATACTAGTAGTTTTGCTATATACATTTGCAACCGCAAATGCAGACACATTATGTATAGGTTATCATGCGAACAATTCAACAGACACTGTAGACACAGTACTAGAAAAGAATGTAACAGTAACACACTCTGTCAATCTTCTAGAAGACAAGCATAACGGAAAACTATGCAAACTAAGAGGGGTAGCCCCATTGCATTTGGGTAAATGTAACATTGCTGGCTGGATCCTGGGAAATCCAGAGTGTGAATCACTCTCCACAGCAAGCTCATGGTCCTACATTGTGGAAACATCTAGTTCAGACAATGGAACGTGTTACCCAGGAGATTTCATCGATTATGAGGAGCTAAGAGAGCAATTGAGCTCAGTGTCATCATTTGAAAGGTTTGAGATATTCCCCAAGACAAGTTCATGGCCCAATCATGACTCGAACAAAGGTGTAACGGCAGCATGTCCTCATGCTGGAGCAAAAAGCTTCTACAAAAATTTAATATGGCTAGTTAAAAAAGGAAATTCATACCCAAAGCTCAGCAAATCCTACATTAATGATAAAGGGAAAGAAGTCCTCGTGCTATGGGGCATTCACCATCCATCTACTACTGCTGACCAACAAAGTCTCTATCAGAATGCAGATACATATGTTTTTGTGGGAACATCAAGATACAGCAAGAAGTTCAAACCGGAAATAGCAATAAGACCCAAAGTGAGGGATCAAGAAGGGAGAATGAACTATTACTGGACACTAGTAGAGCCGGGAGACAAAATAACATTCGAAGCAACTGGAAATCTAGTGGTACCGAGATATGCATTCGCAATGGAAAGAAATGCTGGATCTGGTATTATCATTTCAGATACACCAGTCCACGATTGCAATACAACTTGTCAGACACCCAAGGGTGCTATAAACACCAGCCTCCCATTTCAAGATATACATCCGATCACAATTGGAAAATGTCCAAAATATGTAAAAAGCACAAAATTGAGACTGGCCACAGGATTGAGGAATGTCCCGTCTATTCAATCTAGAGGCCTATTTGGGGCCATTGCCGGCTTCATTGAAGGGGGGTGGACAGGGATGGTAGATGGATGGTACGGTTATCACCATCAAAATGAGCAGGGGTCAGGATATGCAGCCGACCTGAAGAGCACACAGAATGCCATTGACAAGATTACTAACAAAGTAAATTCTGTTATTGAAAAGATGAATACACAGTTCACAGCAGTAGGTAAAGAGTTCAACCACCTGGAAAAAAGAATAGAGAATTTAAATAAAAAAGTTGATGATGGTTTCCTGGACATTTGGACTTACAATGCCGAACTGTTGGTTCTATTGGAAAATGAAAGAACTTTGGACTACCACGATTCAAATGTGAAGAACTTATATGAAAAAGTAAGAAACCAGTTAAAAAACAATGCCAAGGAAATTGGAAATGGCTGCTTTGAATTTTACCACAAATGCGATAACACGTGCATGGAAAGTGTCAAAAATGGGACTTATGACTACCCAAAATACTCAGAGGAAGCAAAATTAAACAGAGAAAAAATAGATGGGGTAAAGCTGGAATCAACAAGGATTTACCAGATTTTGGCGATCTATTCAACTGTCGCCAGTTCATTGGTACTGGTAGTCTCCCTGGGGGCAATCAGTTTTTGGATGTGCTCTAATGGGTCTCTACAGTGTAGAATATGTATTTAA

>LC638349.1Influenza A virus H1N101701

ATGAAGGCAATACTAGTAGTTCTGCTATATACATTTGCAACCGCAAATGCAGACACATTATGTATAGGTTATCATGCGAACAATTCAACAGACACTGTAGACACAGTACTAGAAAAGAATGTAACAGTAACACACTCTGTCAATCTTCTAGAAGACAAGCATAACGGAAAACTATGCAAACTAAGAGGGGTAGCCCCATTGCATTTGGGTAAATGTAACATTGCTGGCTGGATCCTGGGAAATCCAGAGTGTGAATCACTCTCCACAGCAAGCTCATGGTCCTACATTGTGGAAACATCTAGTTCAGACAATGGAACGTGTTACCCAGGAGATTTCATCGATTATGAGGAGCTAAGAGAGCAATTGAGCTCAGTGTCATCATTTGAAAGGTTTGAGATATTCCCCAAGACAAGTTCATGGCCCAATCATGGCTCGAACAAAGGTGTAACGGCAGCATGTCCTCATGCTGGAGCAAAAAGCTTCTACAAAAATTTAATATGGCTAGTTAAAAAAGGAAATTCATACCCAAAGCTCAGCAAATCCTACATTAATGATAAAGGGAAAGAAGTCCTCGTGCTATGGGGCATTCACCATCCATCTACTACTGCTGACCAACAAAGTCTCTATCAGAATGCAGATACATATGTTTTTGTGGGAACATCAAGATACAGCAAGAAGTTCAAACCGGAAATAGCAATAAGACCCAAAGTGAGGGATCAAGAAGGGAGAATGAACTATTACTGGACACTAGTAGAGCCGGGAGACAAAATAACATTCGAAGCAACTGGAAATCTAGTGGTACCGAGATATGCATTCGCAATGGAAAGAAATGCTGGATCTGGTATTATCATTTCAGATACACCAGTCCACGATTGCAATACAACTTGTCAGACACCCAAGGGTGCTATAAACACCAGCCTCCCATTTCAAGATATACATCCGATCACAATTGGAAAATGTCCAAAATATGTAAAAAGCACAAAATTGAGACTGGCCACAGGATTGAGGAATGTCCCGTCTATTCAATCTAGAGGCCTATTTGGGGCCATTGCCGGCTTCATTGAAGGGGGGTGGACAGGGATGGTAGATGGATGGTACGGTTATCACCATCAAAATGAGCAGGGGTCAGGATATGCAGCCGACCTGAAGAGCACACAGAATGCCATTGACAAGATTACTAACAAAGTAAATTCTGTTATTGAAAAGATGAATACACAGTTCACAGCAGTAGGTAAAGAGTTCAACCACCTGGAAAAAAGAATAGAGAATTTAAATAAAAAAGTTGATGATGGTTTCCTGGACATTTGGACTTACAATGCCGAACTGTTGGTTCTATTGGAAAATGAAAGAACTTTGGACTACCACGATTCAAATGTGAAGAACTTATATGAAAAAGTAAGAAACCAGTTAAAAAACAATGCCAAGGAAATTGGAAATGGCTGCTTTGAATTTTACCACAAATGCGATAACACGTGCATGGAAAGTGTCAAAAATGGGACTTATGACTACCCAAAATACTCAGAGGAAGCAAAATTAAACAGAGAAAAAATAGATGGGGTAAAGCTGGAATCAACAAGGATTTACCAGATTTTGGCGATCTATTCAACTGTCGCCAGTTCATTGGTACTGGTAGTCTCCCTGGGGGCAATCAGTTTTTGGATGTGCTCTAATGGGTCTCTACAGTGTAGAATATGTATTTAA

>LC638348.1Influenza A virus H1N101701

ATGAAGGCAATACTAGTAGTTCTGCTATATACATTTGCAACCGCAAATGCAGACACATTATGTATAGGTTATCATGCGAACAATTCAACAGACACTGTAGACACAGTACTAGAAAAGAATGTAACAGTAACACACTCTGTTAATCTTCTAGAAGACAAGCATAACGGAAAACTATGCAAACTAAGAGGGGTAGCCCCATTGCATTTGGGTAAATGTAACATTGCTGGCTGGATCCTGGGAAATCCAGAGTGTGAATCACTCTCCACAGCAAGCTCATGGTCCTACATTGTGGAAACATCTAGTTCAGACAATGGAACGTGTTACCCAGGAGATTTCATCGATTATGAGGAGCTAAGAGAGCAATTGAGCTCAGTGTCATCATTTGAAAGGTTTGAGATATTCCCCAAGACAAGTTCATGGCCCAATCATGACTCGAACAAAGGTGTAACGGCAGCATGTCCTCATGCTGGAGCAAAAAGCTTCTACAAAAATTTAATATGGCTAGTTAAAAAAGGAAATTCATACCCAAAGCTCAGCAAATCCTACATTAATGATAAAGGGAAAGAAGTCCTCGTGCTATGGGGCATTCACCATCCATCTACTACTGCTGACCAACAAAGTCTCTATCAGAATGCAGATACATATGTTTTTGTGGGGACATCAAGATACAGCAAGAAGTTCAAACCGGAAATAGCAATAAGACCCAAAGTGAGGGATCAAGAAGGGAGAATGAACTATTACTGGACACTAGTAGAGCCGGGAGACAAAATAACATTCGAAGCAACTGGAAATCTAGTGGTACCGAGATATGCATTCGCAATGGAAAGAAATGCTGGATCTGGTATTATCATTTCAGATACACCAGTCCACGATTGCAATACAACTTGTCAGACACCCAAGGGTGCTATAAACACCAGCCTCCCATTTCAAGATATACATCCGATCACAATTGGAAAATGTCCAAAATATGTAAAAAGCACAAAATTGAGACTGGCCACAGGATTGAGGAATGTCCCGTCTATTCAATCTAGAGGCCTATTTGGGGCCATTGCCGGCTTCATTGAAGGGGGGTGGACAGGGATGGTAGATGGATGGTACGGTTATCACCATCAAAATGAGCAGGGGTCAGGATATGCAGCCGACCTGAAGAGCACACAGAATGCCATTGACAAGATTACTAACAAAGTAAATTCTGTTATTGAAAAGATGAATACACAGTTCACAGCAGTAGGTAAAGAGTTCAACCACCTGGAAAAAAGAATAGAGAATTTAAATAAAAAAGTTGATGATGGTTTCCTGGACATTTGGACTTACAATGCCGAACTGTTGGTTCTATTGGAAAATGAAAGAACTTTGGACTACCACGATTCAAATGTGAAGAACTTATATGAAAAAGTAAGAAACCAGTTAAAAAACAATGCCAAGGAAATTGGAAATGGCTGCTTTGAATTTTACCACAAATGCGATAACACGTGCATGGAAAGTGTCAAAAATGGGACTTATGACTACCCAAAATACTCAGAGGAAGCAAAATTAAACAGAGAAAAAATAGATGGGGTAAAGCTGGAATCAACAAGGATTTACCAGATTTTGGCGATCTATTCAACTGTCGCCAGTTCATTGGTACTGGTAGTCTCCCTGGGGGCAATCAGTTTTTGGATGTGCTCTAATGGGTCTCTACAGTGTAGAATATGTATTTAA

>LC638347.1Influenza A virus H1N101701

ATGAAGGCAATACTAGTAGTTCTGCTATATACATTTGCAACCGCAAATGCAGACACATTATGTATAGGTTATCATGCGAACAATTCAACAGACACTGTAGACACAGTACTAGAAAAGAATGTAACAGTAACACACTCTGTCAATCTTCTAGAAGACAAGCATAACGGAAAACTATGCAAACTAAGAGGGGTAGCCCCATTGCATTTGGGTAAATGTAACATTGCTGGTTGGATCCTGGGAAATCCAGAGTGTGAATCACTCTCCACAGCAAGCTCATGGTCCTACATTGTGGAAACATCTAGTTCAGACAATGGAACGTGTTACCCAGGAGATTTCATCGATTATGAGGAGCTAAGAGAGCAATTGAGCTCAGTGTCATCATTTGAAAGGTTTGAGATATTCCCCAAGACAAGTTCATGGCCCAATCATGACTCGAACAAAGGTGTAACGGCAGCATGTCCTCATGCTGGAGCAAAAAGCTTCTACAAAAATTTAATATGGCTAGTTAAAAAAGGAAATTCATACCCAAAGCTCAGCAAATCCTACATTAATGATAAAGGGAAAGAAGTCCTCGTGCTATGGGGCATTCACCATCCATCTACTACTGCTGACCAACAAAGTCTCTATCAGAATGCAGATACATATGTTTTTGTGGGAACATCAAGATACAGCAAGAAGTTCAAACCGGAAATAGCAATAAGACCCAAAGTGAGGGATCAAGAAGGGAGAATGAACTATTACTGGACACTAGTAGAGCCGGGAGACAAAATAACATTCGAAGCAACTGGAAATCTAGTGGTACCGAGATATGCATTCGCAATGGAAAGAAATGCTGGATCTGGTATTATCATTTCAGATACACCAGTCCACGATTGCAATACAACTTGTCAGACACCCAAGGGTGCTATAAACACCAGCCTCCCATTTCAAGATATACATCCGATCACAATTGGAAAATGTCCAAAATATGTAAAAAGCACAAAATTGAGACTGGCCACAGGATTGAGGAATGTCCCGTCTATTCAATCTAGAGGCCTATTTGGGGCCATTGCCGGCTTCATTGAAGGGGGGTGGACAGGGATGGTAGATGGATGGTACGGTTATCACCATCAAAATGAGCAGGGGTCAGGATATGCAGCCGACCTGAAGAGCACACAGAATGCCATTGACAAGATTACTAACAAAGTAAATTCTGTTATTGAAAAGATGAATACACAGTTCACAGCAGTAGGTAAAGAGTTCAACCACCTGGAAAAAAGAATAGAGAATTTAAATAAAAAAGTTGATGATGGTTTCCTGGACATTTGGACTTACAATGCCGAACTGTTGGTTCTATTGGAAAATGAAAGAACTTTGGACTACCACGATTCAAATGTGAAGAACTTATATGAAAAAGTAAGAAACCAGTTAAAAAACAATGCCAAGGAAATTGGAAATGGCTGCTTTGAATTTTACCACAAATGCGATAACACGTGCATGGAAAGTGTCAAAAATGGGACTTATGACTACCCAAAATACTCAGAGGAAGCAAAATTAAACAGAGAAAAAATAGATGGGGTAAAGCTGGAATCAACAAGGATTTACCAGATTTTGGCGATCTATTCAACTGTCGCCAGTTCATTGGTACTGGTAGTCTCCCTGGGGGCAATCAGTTTTTGGATGTGCTCTAATGGGTCTCTACAGTGTAGAATATGTATTTAA

>LC638346.1Influenza A virus H1N101701

ATGAAGGCAATACTAGTAGTTCTGCTATATACATTTGCAACCGCAAATGCAGACACATTATGTATAGGTTATCATGCGAACAATTCAACAGACACTGTAGACACAGTACTAGAAAAGAATGTAACAGTAACACACTCTGTTAATCTTCTAGAAGACAAGCATAACGGGAAACTATGCAAACTAAGAGGGGTAGCCCCATTGCATTTGGGTAAATGTAACATTGCTGGCTGGATCCTGGGAAATCCAGAGTGTGAATCATTCTCCACAGCAAGCTCATGGTCCTACATTGTGGAAACATCTAGTTCAGACAATGGAACGTGTTACCCAGGAGATTTCATCGATTATGAGGAGCTAAGAGAGCAATTGAGCTCAGTGTCATCATTTGAAAGGTTTGAGATATTCCCCAAGACAAGTTCATGGCCCAATCATGACTCGAACAAAGGTGTAACGGCAGCATGTCCTCATGCTGGAGCAAAAAGCTTCTACAAAAATTTAATATGGCTAGTTAAAAAAGGAAATTCATACCCAAAGCTCAGCAAATCCTACATTAATGATAAAGGGAAAGAAGTCCTCGTGCTATGGGGCATTCACCATCCATCTACTACTGCTGACCAACAAAGTCTCTATCAGAATGCAGATACATATGTTTTTGTGGGGACATCAAGATACAGCAAGAAGTTCAAGCCGGAAATAGCAATAAGACCCAAAGTGAGGGATCAAGAAGGGAGAATGAACTATTACTGGACACTAGTAGAGCCGGGAGACAAAATAACATTCGAAGCAACTGGAAATCTAGTGGTACCGAGATATGCATTCGCAATGGAAAGAAATGCTGGATCTGGTATTATCATTTCAGATACACCAGTCCACGATTGCAATACAACTTGTCAGACACCCAAGGGTGCTATAAACACCAGCCTCCCATTTCAAAATATACATCCGATCACAATTGGAAAATGTCCAAAATATGTAAAAAGCACAAAATTGAGACTGGCCACAGGATTGAGGAATGTCCCGTCTATTCAATCTAGAGGCCTATTTGGGGCCATTGCCGGCTTCATTGAAGGGGGGTGGACAGGGATGGTAGATGGATGGTACGGTTATCACCATCAAAATGAGCAGGGGTCAGGATATGCAGCCGACCTGAAGAGCACACAGAATGCCATTGACAAGATTACTAACAAAGTAAATTCTGTTATTGAAAAGATGAATACACAGTTCACAGCAGTAGGTAAAGAGTTCAACCACCTGGAAAAAAGTATAGAGAATTTAAATAAAAAAGTTGATGATGGTTTCCTGGACATTTGGACTTACAATGCCGAACTGTTGGTTCTATTGGAAAATGAAAGAACTTTGGACTACCACGATTCAAATGTGAAGAACTTATATGAAAAAGTAAGAAACCAGTTAAAAAACAATGCCAAGGAAATTGGAAATGGCTGCTTTGAATTTTACCACAAATGCGATAACACGTGCATGGAAAGTGTCAAAAATGGGACTTATGACTACCCAAAATACTCAGAGGAAGCAAAATTAAACAGAGAAGAAATAGATGGGGTAAAGCTGGAATCAACAAGGATTTACCAGATTTTGGCGATCTATTCAACTGTCGCCAGTTCATTGGTACTGGTAGTCTCCCTGGGGGCAATCAGTTTTTGGATGTGCTCTAATGGGTCTCTACAGTGTAGAATATGTATTTAA

>LC638345.1Influenza A virus H1N101701

ATGAAGGCAATACTAGTAGTTCTGCTATATACATTTGCAACCGCAAATGCAGACACATTATGTATAGGTTATCATGCGAACAATTCAACAGACACTGTAGACACAGTACTAGAAAAGAATGTAACAGTGACACACTCTGTCAATCTTCTAGAAGACAAGCATAACGGAAAACTATGCAAACTAAGAGGGGTAGCCCCATTGCATTTGGGTAAATGTAACATTGCTGGCTGGATCCTGGGAAATCCAGAGTGTGAATCACTCTCCACAGCAAGCTCATGGTCCTACATTGTGGAAACATCTAGTTCAGACAATGGAACGTGTTACCCAGGAGATTTCATCGATTATGAGGAGCTAAGAGAGCAATTGAGCTCAGTGTCATCATTTGAAAGGTTTGAGATATTCCCCAAGACAAGTTCATGGCCCAATCATGACTCGAACAAAGGTGTAACGGCAGCATGTCCTCATGCTGGAGCAAAAAGCTTCTACAAAAATTTAATATGGCTAGTTAAAAAAGGAAATTCATACCCAAAGCTCAGCAAATCCTACATTAATGATAAAGGGAAAGAAGTCCTCGTGCTATGGGGCATTCACCATCCATCTACTACTGCTGACCAACAAAGTCTCTATCAGAATGCAGATACATATGTTTTTGTGGGAACATCAAGATACAGCAAGAAGTTCAAACCGGAAATAGCAATAAGACCCAAAGTGAGGGATCAAGAAGGGAGAATGGACTATTACTGGACACTAGTAGAGCCGGGAGACAAAATAACATTCGAAGCAACTGGAAATCTAGTGGTACCGAGATATGCATTCGCAATGGAAAGAAATGCTGGATCTGGTATTATCATTTCAGATACACCAGTCCACGATTGCAATACAACTTGTCAGACACCCAAGGGTGCTATAAACACCAGCCTCCCATTTCAAGATATACATCCGATCACAATTGGAAAATGTCCAAAATATGTAAAAAGCACAAAATTGAGACTGGCCACAGGATTGAGGAATGTCCCGTCTATTCAATCTAGAGGCCTATTTGGGGCCATTGCCGGCTTCATTGAAGGGGGGTGGACAGGGATGGTAGATGGATGGTACGGTTATCACCATCAAAATGAGCAGGGGTCAGGATATGCAGCCGACCTGAAGAGCACACAGAATGCCATTGACAAGATTACTAACAAAGTAAATTCTGTTATTGAAAAGATGAATACACAGTTCACAGCAGTAGGTAAAGAGTTCAACCACCTGGAAAAAAGAATAGAGAATTTAAATAAAAAAGTTGATGATGGTTTCCTGGACATTTGGACTTACAATGCCGAACTGTTGGTTCTATTGGAAAATGAAAGAACTTTGGACTACCACGATTCAAATGTGAAGAACTTATATGAAAAAGTAAGAAACCAGTTAAAAAACAATGCCAAGGAAATTGGAAATGGCTGCTTTGAATTTTACCACAAATGCGATAACACGTGCATGGAAAGTGTCAAAAATGGGACTTATGACTACCCAAAATACTCAGAGGAAGCAAAATTAAACAGAGAAAAAATAGATGGGGTAAAGCTGGAATCAACAAGGATTTACCAGATTTTGGCGATCTATTCAACTGTCGCCAGTTCATTGGTACTGGTAGTCTCCCTGGGGGCAATCAGTTTTTGGATGTGCTCTAATGGGTCTCTACAGTGTAGAATATGTATTTAA

>LC638344.1Influenza A virus H1N101701

ATGAAGGCAATACTAGTAGTTCTGCTATATACATTTGCAACCGCAAATGCAGACACATTATGTATAGGTTATCATGCGAACAATTCAACAGACACTGTAGACACAGTACTAGAAAAGAATGTAACAGTAACACACTCTGTCAATCTTCTAGAAGACAAGCATAACGGAAAACTATGCAAACTAAGAGGGGTAGCCCCATTGCATTTGGGTAAATGTAACATTGCTGGCTGGATCCTGGGAAATCCAGAGTGTGAATCACTCTCCACAGCAAGCTCATGGTCCTACATTGTGGAAACATCTAGTTCAGACAATGGAACGTGTTACCCAGGAGATTTCATCGATTATGAGGAGCTAAGAGAGCAATTGAGCTCAGTGTCATCATTTGAAAGGTTTGAGATATTCCCCAAGACAAGTTCATGGCCCAATCATGACTCGAACAAAGGTGTAACGGCAGCATGTCCTCATGCTGGAGCAAAAAGCTTCTACAAAAATTTAATATGGCTAGTTAAAAAAGGAAATTCATACCCAAAGCTCAGCAAATCCTACATTAATGATAAAGGGAAAGAAGTCCTCGTGCTATGGGGCATTCACCATCCATCTACTACTGCTGACCAACAAAGTCTCTATCAGAATGCAGATACATATGTTTTTGTGGGAACATCAAGATACAGCAAGAAGTTCAAACCGGAAATAGCAATAAGACCCAAAGTGAGGGATCAAGAAGGGAGAATGAACTATTACTGGACACTAGTAGAGCCGGGAGACAAAATAACATTCGAAGCAACTGGAAATCTAGTGGTACCGAGATATGCATTCGCAATGGAAAGAAATGCTGGATCTGGTATTATCATTTCAGATACACCAGTCCACGATTGCAATACAACTTGTCAGACACCCAAGGGTGCTATAAACACCAGCCTCCCATTTCAAGATATACATCCGATCACAATTGGAAAATGTCCAAAATATGTAAAAAGCACAAAATTGAGACTGGCCACAGGATTGAGGAATGTCCCGTCTATTCAATCTAGAGGCCTATTTGGGGCCATTGCCGGCTTCATTGAAGGGGGGTGGACAGGGATGGTAGATGGATGGTACGGTTATCACCATCAAAATGAGCAGGGGTCAGGATATGCAGCCGACCTGAAGAGCACACAGAATGCCATTGACAAGATTACTAACAAAGTAAATTCTGTTATTGAAAAGATGAATACACAGTTCACAGCAGTAGGTAAAGAGTTCAACCACCTGGAAAAAAGAATAGAGAATTTAAATAAAAAAGTTGATGATGGTTTCCTGGACATTTGGACTTACAATGCCGAACTGTTGGTTCTATTGGAAAATGAAAGAACTTTGGACTACCACGATTCAAATGTGAAGAACTTATATGAAAAAGTAAGAAACCAGTTAAAAAACAATGCCAAGGAAATTGGAAATGGCTGCTTTGAATTTTACCACAAATGCGATAACACGTGCATGGAAAGTGTCAAAAATGGGACTTATGACTACCCAAAATACTCAGAGGAAGCAAAATTAAACAGAGAAAAAATAGATGGGGTAAAGCTGGAATCAACAAGGATTTACCAGATTTTGGCGATCTATTCAACTGTCGCCAGTTCATTGGTACTGGTAGTCTCCCTGGGGGCAATCAGTTTTTGGATGTGCTCTAATGGGTCTCTACAGTGTAGAATATGTATTTAA

>LC638343.1Influenza A virus H1N101701

ATGAAGGCAATACTAGTAGTTCTGCTATATACATTTGCAACCGCAAATGCAGACACATTATGTATAGGTTATCATGCGAACAATTCAACAGACACTGTAGACACAGTACTAGAAAAGAATGTAACAGTGACACACTCTGTCAATCTTCTAGAAGACAAGCATAACGGAAAACTATGCAAACTAAGAGGGGTAGCCCCATTGCATTTGGGTAAATGTAACATTGCTGGCTGGATCCTGGGAAATCCAGAGTGTGAATCACTCTCCACAGCAAGCTCATGGTCCTACATTGTGGAAACATCTAGTTCAGACAATGGAACGTGTTACCCAGGAGATTTCATCGATTATGAGGAGCTAAGAGAGCAATTGAGCTCAGTGTCATCATTTGAAAGGTTTGAGATATTCCCCAAGACAAGTTCATGGCCCAATCATGACTCGAACAAAGGTGTAACGGCAGCATGTCCTCATGCTGGAGCAAAAAGCTTCTACAAAAATTTAATATGGCTAGTTAAAAAAGGAAATTCATACCCAAAGCTCAGCAAATCCTACATTAATGATAAAGGGAAAGAAGTCCTCGTGCTATGGGGCATTCACCATCCATCTACTACTGCTGACCAACAAAGTCTCTATCAGAATGCAGATACATATGTTTTTGTGGGAACATCAAGATACAGCAAGAAGTTCAAACCGGAAATAGCAATAAGACCCAAAGTGAGGGATCAAGAAGGGAGAATGGACTATTACTGGACACTAGTAGAGCCGGGAGACAAAATAACATTCGAAGCAACTGGAAATCTAGTGGTACCGAGATATGCATTCGCAATGGAAAGAAATGCTGGATCTGGTATTATCATTTCAGATACACCAGTCCACGATTGCAATACAACTTGTCAGACACCCAAGGGTGCTATAAACACCAGCCTCCCATTTCAAGATATACATCCGATCACAATTGGAAAATGTCCAAAATATGTAAAAAGCACAAAATTGAGACTGGCCACAGGATTGAGGAATGTCCCGTCTATTCAATCTAGAGGCCTATTTGGGGCCATTGCCGGCTTCATTGAAGGGGGGTGGACAGGGATGGTAGATGGATGGTACGGTTATCACCATCAAAATGAGCAGGGGTCAGGATATGCAGCCGACCTGAAGAGCACACAGAATGCCATTGACAAGATTACTAACAAAGTAAATTCTGTTATTGAAAAGATGAATACACAGTTCACAGCAGTAGGTAAAGAGTTCAACCACCTGGAAAAAAGAATAGAGAATTTAAATAAAAAAGTTGATGATGGTTTCCTGGACATTTGGACTTACAATGCCGAACTGTTGGTTCTATTGGAAAATGAAAGAACTTTGGACTACCACGATTCAAATGTGAAGAACTTATATGAAAAAGTAAGAAACCAGTTAAAAAACAATGCCAAGGAAATTGGAAATGGCTGCTTTGAATTTTACCACAAATGCGATAACACGTGCATGGAAAGTGTCAAAAATGGGACTTATGACTACCCAAAATACTCAGAGGAAGCAAAATTAAACAGAGAAAAAATAGATGGGGTAAAGCTGGAATCAACAAGGATTTACCAGATTTTGGCGATCTATTCAACTGTCGCCAGTTCATTGGTACTGGTAGTCTCCCTGGGGGCAATCAGTTTTTGGATGTGCTCTAATGGGTCTCTACAGTGTAGAATATGTATTTAA

>LC638342.1Influenza A virus H1N101701

ATGAAGGCAATACTAGTAGTTCTGCTATATACATTTGCAACCGCAAATGCAGACACATTATGTATAGGTTATCATGCGAACAATTCAACAGACACTGTAGACACAGTACTAGAAAAGAATGTAACAGTAACACACTCTGTCAATCTTCTAGAAGACAAGCATAACGGAAAACTATGCAAACTAAAAGGGGTAGCCCCATTGCATTTGGGTAAATGTAACATTGCTGGCTGGATCCTGGGAAATCCAGAGTGTGAATCACTCTCCACAGCAAGCTCATGGTCCTACATTGTGGAAACATCTAGTTCAGACAATGGAACGTGTTACCCAGGAGATTTCATCGATTATGAGGAGCTAAGAGAGCAATTGAGCTCAGTGTCATCATTTGAAAGGTTTGAGATATTCCCCAAGACAAGTTCATGGCCCAATCATGACTCGAACAAAGGTGTAACGGCAGCATGTCCTCATGCTGGAGCAAAAAGCTTCTACAAAAATTTAATATGGCTAGTTAAAAAAGGAAATTCATACCCAAAGCTCAGCAAATCCTACATTAATGATAAAGGGAAAGAAGTCCTCGTGCTATGGGGCATTCACCATCCATCTACTACTGCTGACCAACAAAGTCTCTATCAGAATGCAGATACATATGTTTTTGTGGGAACATCAAGATACAGCAAGAAGTTCAAACCGGAAATAGCAATAAGACCCAAAGTGAGGGATCAAGAAGGGAGAATGAACTATTACTGGACACTAGTAGAGCCGGGAGACAAAATAACATTCGAAGCAACTGGAAATCTAGTGGTACCGAGATATGCATTCGCAATGGAAAGAAATGCTGGATCTGGTATTATCATTTCAGATACACCAGTCCACGATTGCAATACAACTTGTCAGACACCCAAGGGTGCTATAAACACCAGCCTCCCATTTCAAGATATACATCCGATCACAATTGGAAAATGTCCAAAATATGTAAAAAGCACAAAATTGAGACTGGCCACAGGATTGAGGAATGTCCCGTCTATTCAATCTAGAGGCCTATTTGGGGCCATTGCCGGCTTCATTGAAGGGGGGTGGACAGGGATGGTAGATGGATGGTACGGTTATCACCATCAAAATGAGCAGGGGTCAGGATATGCAGCCGACCTGAAGAGCACACAGAATGCCATTGACAAGATTACTAACAAAGTAAATTCTGTTATTGAAAAGATGAATACACAGTTCACAGCAGTAGGTAAAGAGTTCAACCACCTGGAAAAAAGAATAGAGAATTTAAATAAAAAAGTTGATGATGGTTTCCTGGACATTTGGACTTACAATGCCGAACTGTTGGTTCTATTGGAAAATGAAAGAACTTTGGACTACCACGATTCAAATGTGAAGAACTTATATGAAAAAGTAAGAAACCAGTTAAAAAACAATGCCAAGGAAATTGGAAATGGCTGCTTTGAATTTTACCACAAATGCGATAACACGTGCATGGAAAGTGTCAAAAATGGGACTTATGACTACCCAAAATACTCAGAGGAAGCAAAATTAAACAGAGAAAAAATAGATGGGGTAAAGCTGGAATCAACAAGGATTTACCAGATTTTGGCGATCTATTCAACTGTCGCCAGTTCATTGGTACTGGTAGTCTCCCTGGGGGCAATCAGTTTTTGGATGTGCTCTAATGGGTCTCTACAGTGTAGAATATGTATTTAA

>LC638341.1Influenza A virus H1N101701

ATGAAGGCAATACTAGTAGTTCTGCTATATACATTTGCAACCGCAAATGCAGACACATTATGTATAGGTTATCATGCGAACAATTCAACAGACACTGTAGACACAGTACTAGAAAAGAATGTAACAGTAACACACTCTGTTAATCTTCTAGAAGACAAGCATAACGGGAAACTATGCAAACTAAGAGGGGTAGCCCCATTGCATTTGGGTAAATGTAACATTGCTGGCTGGATCCTGGGAAATCCAGAGTGTGAATCACTCTCCACAGCAAGCTCCTGGTCCTACATTGTGGAAACATCTAGTTCAGACAATGGAACGTGTTACCCAGGAGATTTCATCGATTATGAGGAGCTAAGAGAGCAATTGAGCTCAGTGTCATCATTTGAAAGGTTTGAGATATTCCCCAAGACAAGTTCATGGCCCAATCATGACTCGAACAAAGGTGTAACGGCAGCATGTCCTCATGCTGGAGCAAAAAGCTTCTACAAAAATTTAATATGGCTAGTTAAAAAAGGAAATTCATACCCAAAGCTCAGCAAATCCTACATTAATGATAAAGGGAAAGAAGTCCTCGTGCTATGGGGCATTCACCATCCATCTACTACTGCTGACCAACAAAGTCTCTATCAGAATGCAGATACATATGTTTTTGTGGAGACATCAAGATACAGCAAGAAGTTCAAGCCGGAAATAGCAATAAGACCCAAAGTGAGGGATCAAGAAGGGAGAATGAACTATTACTGGACACTAGTAGAGCCGGGAGACAAAATAACATTCGAAGCAACTGGAAATCTAGTGGTACCGAGATATGCATTCGCAATGGAAAGAAATGCTGGATCTGGTATTATCATTTCAGATACACCAGTCCACGATTGCAATACAACTTGTCAGACACCCAAGGGTGCTATAAACACCAGCCTCCCATTTCAAAATATACATCCGATCACAATTGGAAAATGTCCAAAATATGTAAAAAGCACAAAATTGAGACTGGCCACAGGATTGAGGAATGTCCCGTCTATTCAATCTAGAGGCCTATTTGGGGCCATTGCCGGCTTCATTGAAGGGGGGTGGACAGGGATGGTAGATGGATGGTACGGTTATCACCATCAAAATGAGCAGGGGTCAGGATATGCAGCCGACCTGAAGAGCACACAGAATGCCATTGACAAGATTACTAACAAAGTAAATTCTGTTATTGAAAAGATGAATACACAGTTCACAGCAGTAGGTAAAGAGTTCAACCACCTGGAAAAAAGAATAGAGAATTTAAATAAAAAAGTTGATGATGGTTTCCTGGACATTTGGACTTACAATGCCGAACTGTTGGTTCTATTGGAAAATGAAAGAACTTTGGACTACCACGATTCAAATGTGAAGAACTTATATGAAAAAGTAAGAAACCAGTTAAAAAACAATGCCAAGGAAATTGGAAATGGCTGCTTTGAATTTTACCACAAATGCGATAACACGTGCATGGAAAGTGTCAAAAATGGGACTTATGACTACCCAAAATACTCAGAGGAAGCAAAATTAAACAGAGAAGAAATAGATGGGGTAAAGCTGGAATCAACAAGGATTTACCAGATTTTGGCGATCTATTCAACTGTCGCCAGTTCATTGGTACTGGTAGTCTCCCTGGGGGCAATCAGTTTTTGGATGTGCTCTAATGGGTCTCTACAGTGTAGAATATGTATTTAA

>LC638340.1Influenza A virus H1N101701

ATGAAGGCAATACTAGTAGTTCTGCTATATACATTTGCAACCGCAAATGCAGACACATTATGTATAGGTTACCATGCGAACAATTCAACAGACACTGTAGACACAGTACTAGAAAAGAATGTAACAGTAACACACTCTGTTAATCTTCTAGAAGACAAGCATAACGGGAAACTATGCAAACTAAGAGGGGTAGCCCCATTGCATTTGGGTAAATGTAACATTGCTGGCTGGATCCTGGGAAATCCAGAGTGTGAATCACTCTCCACAGCAAGCTCATGGTCCTACATTGTGGAAACATCTAGTTCAGACAATGGAACGTGTTACCCAGGAGATTTCATCGATTATGAGGAGCTAAGAGAGCAATTGAGCTCAGTGTCATCATTTGAAAGGTTTGAGATATTCCCCAAGACAAGTTCATGGCCCAATCATGACTCGAACAAAGGTGTAACGGCAGCATGTCCTCATGCTGGAGCAAAAGGCTTCTACAAAAATTTAATATGGCTAGTTAAAAAAGGAAATTCATACCCAAAGCTCAGCAAATCCTACATTAATGATAAAGGGAAAGAAGTCCTCGTGCTATGGGGCATTCACCATCCATCTACTACTGCTGACCAACAAAGTCTCTATCAGAATGCAGATACATATGTTTTTGTGGGGACATCAAGATACAGCAAGAAGTTCAAGCCGGAAATAGCAATAAGACCCAAAGTGAGGGATCAAGAAGGGAGAATGAACTATTACTGGACACTAGTAGAGCCGGGAGACAAAATAACATTCGAAGCAACTGGAAATCTAGTGGTACCGAGATATGCATTCGCAATGGAAAGAAATGCTGGATCTGGTATTATCATTTCAGATACACCAGTCCACGATTGCAATACAACTTGTCAGACACCCAAGGGTGCTATAAACACCAGCCTTCCATTTCAGAATATACATCCGATCACAATTGGAAAATGTCCAAAATATGTAAAAAGCACAAAATTGAGACTGGCCACAGGATTGAGGAATGTCCCGTCTATTCAATCTAGAGGCCTATTTGGGGCCATTGCCGGCTTCATTGAAGGGGGGTGGACAGGGATGGTAGATGGATGGTACGGTTATCACCATCAAAATGAGCAGGGGTCAGGATATGCAGCCGACCTGAAGAGCACACAGAATGCCATTGACAAGATTACTAACAAAGTAAATTCTGTTATTGAAAAGATGAATACACAGTTCACAGCAGTAGGTAAAGAGTTCAACCACCTGGAAAAAAGAATAGAGAATTTAAATAAAAAAGTTGATGATGGTTTCCTGGACATTTGGACTTACAATGCCGAACTGTTGGTTCTATTGGAAAATGAAAGAACTTTGGACTACCACGATTCAAATGTGAAGAACTTATATGAAAAGGTAAGAAACCAGTTAAAAAACAATGCCAAGGAAATTGGAAATGGCTGCTTTGAATTTTACCACAAATGCGATAACACGTGCATGGAAAGTGTCAAAAATGGGACTTATGACTACCCAAAATACTCAGAGGAAGCAAAATTAAACAGAGAAGAAATAGATGGGGTAAAACTGGAATCAACAAGGATTTACCAGATTTTGGCGATCTATTCAACTGTCGCCAGTTCATTGGTATTGGTAGTCTCCCTGGGGGCAATCAGTTTTTGGATGTGCTCTAATGGGTCTCTACAGTGTAGAATATGTATTTAA

>LC638339.1Influenza A virus H1N101701

ATGAAGGCAATACTAGTAGTTCTGCTATATACATTTGCAACCGCAAATGCAGACACATTATGTATAGGTTATCATGCGAACAATTCAACAGACACTGTAGACACAGTACTAGAAAAGAATGTAACAGTAACACACTCTGTCAATCTTCTAGAAGACAAGCATAACGGAAAACTATGCAAACTAAGAGGGGTAGCCCCATTGCATTTGGGTAAATGTAACATTGCTGGCTGGATCCTGGGAAATCCAGAGTGTGAATCACTCTCCACAGCAAGCTCATGGTCCTACATTGTGGAAACATCTAGTTCAGACAATGGAACGTGTTACCCAGGAGATTTCATCGATTATGAGGAGCTAAGAGAGCAATTGAGCTCAGTGTCATCATTTGAAAGGTTTGAGATATTCCCCAAGACAAGTTCATGGCCCAATCATGACTCGAACAAAGGTGTAACGGCAGCATGTCCTCATGCTGGAGCAAAAAGCTTCTACAAAAATTTAATATGGCTAGTTAAAAAAGGAAATTCATACCCAAAGCTCAGCAAATCCTACATTAATGATAAAGGGAAAGAAGTCCTCGTGCTATGGGGCATTCACCATCCATCTACTACTGCTGACCAACAAAGTCTCTATCAGAATGCAGATACATATGTTTTTGTGGGAACATCAAGATACAGCAAGAAGTTCAAACCGGAAATAGCAATAAGACCCAAAGTGAGGGATCAAGAAGGGAGAATGAACTATTACTGGACACTAGTAGAGCCGGGAGACAAAATAACATTCGAAGCAACTGGAAATCTAGTGGTACCGAGATATGCATTCGCAATGGAAAGAAATGCTGGATCTGGTATTATCATTTCAGATACACCAGTCCACGATTGCAATACAACTTGTCAGACACCCAAGGGTGCTATAAACACCAGCCTCCCATTTCAAGATATACATCCGATCACAATTGGAAAATGTCCAAAATATGTAAAAAGCACAAAATTGAGACTGGCCACAGGATTGAGGAATGTCCCGTCTATTCAATCTAGAGGCCTATTTGGGGCCATTGCCGGCTTCATTGAAGGGGGGTGGACAGGGATGGTAGATGGATGGTACGGTTATCACCATCAAAATGAGCAGGGGTCAGGATATGCAGCCGACCTGAAGAGCACACAGAATGCCATTGACAAGATTACTAACAAAGTAAATTCTGTTATTGAAAAGATGAATACACAGTTCACAGCAGTAGGTAAAGAGTTCAACCACCTGGAAAAAAGAATAGAGAATTTAAATAAAAAAGTTGATGATGGTTTCCTGGACATTTGGACTTACAATGCCGAACTGTTGGTTCTATTGGAAAATGAAAGAACTTTGGACTACCACGATTCAAATGTGAAGAACTTATATGAAAAAGTAAGAAACCAGTTAAAAAACAATGCCAAGGAAATTGGAAATGGCTGCTTTGAATTTTACCACAAATGCGATAACACGTGCATGGAAAGTGTCAAAAATGGGACTTATGACTACCCAAAATACTCAGAGGAAGCAAAATTAAACAGAGAAAAAATAGATGGGGTAAAGCTGGAATCAACAAGGATTTACCAGATTTTGGCGATCTATTCAACTGTCGCCAGTTCATTGGTACTGGTAGTCTCCCTGGGGGCAATCAGTTTTTGGATGTGCTCTAATGGGTCTCTACAGTGTAGAATATGTATTTAA

>LC638338.1Influenza A virus H1N101701

ATGAAGGCAATACTAGTAGTTCTGCTATATACATTTGCAACCGCAAATGCAGACACATTATGTATAGGTTATCATGCGAACAATTCAACAGACACTGTAGACACAGTACTAGAAAAGAATGTAACAGTAACACACTCTGTCAATCTTCTAGAAGACAAGCATAACGGAAAACTATGCAAACTAAGAGGGGTAGCCCCATTGCATTTGGGTAAATGTAACATTGCTGGCTGGATCCTGGGAAATCCAGAGTGTGAATCACTCTCCACAGCAAGCTCATGGTCCTACATTGTGGAAACATCTAGTTCAGACAATGGAACGTGTTACCCAGGAGATTTCATCGATTATGAGGAGCTAAGAGAGCAATTGAGCTCAGTGTCATCATTTGAAAGGTTTGAGATATTCCCCAAGACAAGTTCATGGCCCAATCATGACTCGAACAAAGGTGTAACGGCAGCATGTCCTCATGCTGGAGCAAAAAGCTTCTACAAAAATTTAATATGGCTAGTTAAAAAAGGAAATTCATACCCAAAGCTCAGCAAATCCTACATTAATGATAAAGGGAAAGAAGTCCTCGTGCTATGGGGCATTCACCATCCATCTACTACTGCTGACCAACAAAGTCTCTATCAGAATGCAGATACATATGTTTTTGTGGGAACATCAAGATACAGCAAGAAGTTCAAACCGGAAATAGCAATAAGACCCAAAGTGAGGGATCAAGAAGGGAGAATGAACTATTACTGGACACTAGTAGAGCCGGGAGACAAAATAACATTCGAAGCAACTGGAAATCTAGTGGTACCGAGATATGCATTCGCAATGGAAAGAAATGCTGGATCTGGTATTATCATTTCAGATACACCAGTCCACGATTGCAATACAACTTGTCAGACACCCAAGGGTGCTATAAACACCAGCCTCCCATTTCAAGATATACATCCGATCACAATTGGAAAATGTCCAAAATATGTAAAAAGCACAAAATTGAGACTGGCCACAGGATTGAGGAATGTCCCGTCTATTCAATCTAGAGGCCTATTTGGGGCCATTGCCGGCTTCATTGAAGGGGGGTGGACAGGGATGGTAGATGGATGGTACGGTTATCACCATCAAAATGAGCAGGGGTCAGGATATGCAGCCGACCTGAAGAGCACACAGAATGCCATTGACAAGATTACTAACAAAGTAAATTCTGTTATTGAAAAGATGAATACACAGTTCACAGCAGTAGGTAAAGAGTTCAACCACCTGGAAAAAAGAATAGAGAATTTAAATAAAAAAGTTGATGATGGTTTCCTGGACATTTGGACTTACAATGCCGAACTGTTGGTTCTATTGGAAAATGAAAGAACTTTGGACTACCACGATTCAAATGTGAAGAACTTATATGAAAAAGTAAGAAACCAGTTAAAAAACAATGCCAAGGAAATTGGAAATGGCTGCTTTGAATTTTACCACAAATGCGATAACACGTGCATGGAAAGTGTCAAAAATGGGACTTATGACTACCCAAAATACTCAGAGGAAGCAAAATTAAACAGAGAAAAAATAGATGGGGTAAAGCTGGAATCAACAAGGATTTACCAGATTTTGGCGATCTATTCAACTGTCGCCAGTTCATTGGTACTGGTAGTCTCCCTGGGGGCAATCAGTTTTTGGATGTGCTCTAATGGGTCTCTACAGTGTAGAATATGTATTTAA

>LC638337.1Influenza A virus H1N101701

ATGAAGGCAATACTAGTAGTTCTGCTATATACATTTGCAACCGCAAATGCAGACACATTATGTATAGGTTATCATGCGAACAATTCAACAGACACTGTAGACACAGTACTAGAAAAGAATGTAACAGTAACACACTCTGTCAATCTTCTAGAAGACAAGCATAACGGAAAACTATGCAAACTAAGAGGGGTAGCCCCATTGCATTTGGGTAAATGTAACATTGCTGGCTGGATCCTGGGAAATCCAGAGTGTGAATCACTCTCCACAGCAAGCTCATGGTCCTACATTGTGGAAACATCTAGTTCAGACAATGGAACGTGTTACCCAGGAGATTTCATCGATTATGAGGAGCTAAGAGAGCAATTGAGCTCAGTGTCATCATTTGAAAGGTTTGAGATATTCCCCAAGACAAGTTCATGGCCCAATCATGAATCGAACAAAGGTGTAACGGCAGCATGTCCTCATGCTGGAGCAAAAAGCTTCTACAAAAATTTAATATGGCTAGTTAAAAAAGGAAATTCATACCCAAAGCTCAGCAAATCCTACATTAATGATAAAGGGAAAGAAGTCCTCGTGCTATGGGGCATTCACCATCCATCTACTACTGCTGACCAACAAAGTCTCTATCAGAATGCAGATACATATGTTTTTGTGGGAACATCAAGATACAGCAAGAAGTTCAAACCGGAAATAGCAATAAGACCCAAAGTGAGGGATCAAGAAGGGAGAATGAACTATTACTGGACACTAGTAGAGCCGGGAGACAAAATAACATTCGAAGCAACTGGAAATCTAGTGGTACCGAGATATGCATTCGCAATGGAAAGAAATGCTGGATCTGGTATTATCATTTCAGATACACCAGTCCACGATTGCAATACAACTTGTCAGACACCCAAGGGTGCTATAAACACCAGCCTCCCATTTCAAGATATACATCCGATCACAATTGGAAAATGTCCAAAATATGTAAAAAGCACAAAATTGAGACTGGCCACAGGATTGAGGAATGTCCCGTCTATTCAATCTAGAGGCCTATTTGGGGCCATTGCCGGCTTCATTGAAGGGGGGTGGACAGGGATGGTAGATGGATGGTACGGTTATCACCATCAAAATGAGCAGGGGTCAGGATATGCAGCCGACCTGAAGAGCACACAGAATGCCATTGACAAGATTACTAACAAAGTAAATTCTGTTATTGAAAAGATGAATACACAGTTCACAGCAGTAGGTAAAGAGTTCAACCACCTGGAAAAAAGAATAGAGAATTTAAATAAAAAAGTTGATGATGGTTTCCTGGACATTTGGACTTACAATGCCGAACTGTTGGTTCTATTGGAAAATGAAAGAACTTTGGACTACCACGATTCAAATGTGAAGAACTTATATGAAAAAGTAAGAAACCAGTTAAAAAACAATGCCAAGGAAATTGGAAATGGCTGCTTTGAATTTTACCACAAATGCGATAACACGTGCATGGAAAGTGTCAAAAATGGGACTTATGACTACCCAAAATACTCAGAGGAAGCAAAATTAAACAGAGAAAAAATAGATGGGGTAAAGCTGGAATCAACAAGGATTTACCAGATTTTGGCGATCTATTCAACTGTCGCCAGTTCATTGGTACTGGTAGTCTCCCTGGGGGCAATCAGTTTTTGGATGTGCTCTAATGGGTCTCTACAGTGTAGAATATGTATTTAA

>LC638336.1Influenza A virus H1N101701

ATGAAGGCAATACTAGTAGTTCTGCTATATACATTTGCAACCGCAAATGCAGACACATTATGTATAGGTTACCATGCGAACAATTCAACAGACACTGTAGACACAGTACTAGAAAAGAATGTAACAGTAACACACTCTGTTAATCTTCTAGAAGACAAGCATAACGGGAAACTATGCAAACTAAGAGGGGTAGCCCCATTGCATTTGGGTAAATGTAACATTGCTGGCTGGATCCTGGGAAATCCAGAGTGTGAATCACTCTCCACAGCAAGCTCATGGTCCTACATTGTGGAAACATCTAGTTCAGACAATGGAACGTGTTACCCAGGAGATTTCATCGATTATGAGGAGCTAAGAGAGCAATTGAGCTCAGTGTCATCATTTGAAAGGTTTGAGATATTCCCCAAGACAAGTTCATGGCCCAATCATGACTCGAACAAAGGTGTAACGGCAGCATGTCCTCATGCTGGAGCAAAAGGCTTCTACAAAAATTTAATATGGCTAGTTAAAAAAGGAAATTCATACCCAAAGCTCAGCAAATCCTACATTAATGATAAAGGGAAAGAAGTCCTCGTGCTATGGGGCATTCACCATCCATCTACTACTGCTGACCAACAAAGTCTCTATCAGAATGCAGATACATATGTTTTTGTGGGGACATCAAGATACAGCAAGAAGTTCAAGCCGGAAATAGCAATAAGACCCAAAGTGAGGGATCAAGAAGGGAGAATGAACTATTACTGGACACTAGTAGAGCCGGGAGACAAAATAACATTCGAAGCAACTGGAAATCTAGTGGTACCGAGATATGCATTCGCAATGGAAAGAAATGCTGGATCTGGTATTATCATTTCAGATACACCAGTCCACGATTGCAATACAACTTGTCAGACACCCAAGGGTGCTATAAACACCAGCCTCCCATTTCAGAATATACATCCGATCACAATTGGAAAATGTCCAAAATATGTAAAAAGCACAAAATTGAGACTGGCCACAGGATTGAGGAATGTCCCGTCTATTCAATCTAGAGGCCTATTTGGGGCCATTGCCGGCTTCATTGAAGGGGGGTGGACAGGGATGGTAGATGGATGGTACGGTTATCACCATCAAAATGAGCAGGGGTCAGGATATGCAGCCGACCTGAAGAGCACACAGAATGCCATTGACAAGATTACTAACAAAGTAAATTCTGTTATTGAAAAGATGAATACACAGTTCACAGCAGTAGGTAAAGAGTTCAACCACCTGGAAAAAAGAATAGAGAATTTAAATAAAAAAGTTGATGATGGTTTCCTGGACATTTGGACTTACAATGCCGAACTGTTGGTTCTATTGGAAAATGAAAGAACTTTGGACTACCACGATTCAAATGTGAAGAACTTATATGAAAAGGTAAGAAACCAGTTAAAAAACAATGCCAAGGAAATTGGAAATGGCTGCTTTGAATTTTACCACAAATGCGATAACACGTGCATGGAAAGTGTCAAAAATGGGACTTATGACTACCCAAAATACTCAGAGGAAGCAAAATTAAACAGAGAAGAAATAGATGGGGTAAAACTGGAATCAACAAGGATTTACCAGATTTTGGCGATCTATTCAACTGTCGCCAGTTCATTGGTATTGGTAGTCTCCCTGGGGGCAATCAGTTTTTGGATGTGCTCTAATGGGTCTCTACAGTGTAGAATATGTATTTAA

>LC638335.1Influenza A virus H1N101701

ATGAAGGCAATACTAGTAGTTCTGCTATATACATTTGCAACCGCAAATGCAGACACATTATGTATAGGTTACCATGCGAACAATTCAACAGACACTGTAGACACAGTACTAGAAAAGAATGTAACAGTAACACACTCTGTTAATCTTCTAGAAGACAAGCATAACGGGAAACTATGCAAACTAAGAGGGGTAGCCCCATTGCATTTGGGTAAATGTAACATTGCTGGCTGGATCCTGGGAAATCCAGAGTGTGAATCACTCTCCACAGCAAGCTCATGGTCCTACATTGTGGAAACATCTAGTTCAGACAATGGAACGTGTTACCCAGGAGATTTCATCGATTATGAGGAGCTAAGAGAGCAATTGAGCTCAGTGTCATCATTTGAAAGGTTTGAGATATTCCCCAAGACAAGTTCATGGCCCAATCATGACTCGAACAAAGGTGTAACGGCAGCATGTCCTCATGCTGGAGCAAAAGGCTTCTACAAAAATTTAATATGGCTAGTTAAAAAAGGAAATTCATACCCAAAGCTCAGCAAATCCTACATTAATGATAAAGGGAAAGAAGTCCTCGTGCTATGGGGCATTCACCATCCATCTACTACTGCTGACCAACAAATTCTCTATCAGAATGCAGATACATATGTTTTTGTGGGGACATCAAGATACAGCAAGAAGTTCAAGCCGGAAATAGCAATAAGACCCAAAGTGAGGGATCAAGAAGGGAGAATGAACTATTACTGGACACTAGTAGAGCCGGGAGACAAAATAACATTCGAAGCAACTGGAAATCTAGTGGTACCGAGATATGCATTCGCAATGGAAAGAAATGCTGGATCTGGTATTATCATTTCAGATACACCAGTCCACGATTGCAATACAACTTGTCAGACACCCAAGGGTGCTATAAACACCAGCCTCCCATTTCAGAATATACATCCGATCACAATTGGAAAATGTCCAAAATATGTAAAAAGCACAAAATTGAGACTGGCCACAGGATTGAGGAATGTCCCGTCTATTCAATCTAGAGGCCTATTTGGGGCCATTGCCGGCTTCATTGAAGGGGGGTGGACAGGGATGGTAGATGGATGGTACGGTTATCACCATCAAAATGAGCAGGGGTCAGGATATGCAGCCGACCTGAAGAGCACACAGAATGCCATTGACAAGATTACTAACAAAGTAAATTCTGTTATTGAAAAGATGAATACACAGTTCACAGCAGTAGGTAAAGAGTTCAACCACCTGGAAAAAAGAATAGAGAATTTAAATAAAAAAGTTGATGATGGTTTCCTGGACATTTGGACTTACAATGCCGAACTGTTGGTTCTATTGGAAAATGAAAGAACTTTGGACTACCACGATTCAAATGTGAAGAACTTATATGAAAAGGTAAGAAACCAGTTAAAAAACAATGCCAAGGAAATTGGAAATGGCTGCTTTGAATTTTACCACAAATGCGATAACACGTGCATGGAAAGTGTCAAAAATGGGACTTATGACTACCCAAAATACTCAGAGGAAGCAAAATTAAACAGAGAAGAAATAGATGGGGTAAAACTGGAATCAACAAGGATTTACCAGATTTTGGCGATCTATTCAACTGTCGCCAGTTCATTGGTATTGGTAGTCTCCCTGGGGGCAATCAGTTTTTGGATGTGCTCTAATGGGTCTCTACAGTGTAGAATATGTATTTAA

>LC638334.1Influenza A virus H1N101701

ATGAAGGCAATACTAGTAGTTCTGCTATATACATTTGCAACCGCAAATGCAGACACATTATGTATAGGTTACCATGCGAACAATTCAACAGACACTGTAGACACAGTACTAGAAAAGAATGTAACAGTAACACACTCTGTTAATCTTCTAGAAGACAAGCATAACGGGAAACTATGCAAACTAAGAGGGGTAGCCCCATTGCATTTGGGTAAATGTAACATTGCTGGCTGGATCCTGGGAAATCCAGAGTGTGAATCACTCTCCACAGCAAGCTCATGGTCCTACATTGTGGAAACATCTAGTTCAGACAATGGAACGTGTTACCCAGGAGATTTCATCGATTATGAGGAGCTAAGAGAGCAATTGAGCTCAGTGTCATCATTTGAAAGGTTTGAGATATTCCCCAAGACAAGTTCATGGCCCAATCATGACTCGAACAAAGGTGTAACGGCAGCATGTCCTCATGCTGGAGCAAAAGGCTTCTACAAAAATTTAATATGGCTAGTTAAAAAAGGAAATTCATACCCAAAGCTCAGCAAATCCTACATTAATGATAAAGGGAAAGAAGTCCTCGTGCTATGGGGCATTCACCATCCATCTACTACTGCTGACCAACAAAGTCTCTATCAGAATGCAGATACATATGTTTTTGTGGGGACATCAAGATACAGCAAGAAGTTCAAGCCGGAAATAGCAATAAGACCCAAAGTGAGGGATCAAGAAGGGAGAATGAACTATTACTGGACACTAGTAGAGCCGGGAGACAAAATAACATTCGAAGCAACTGGAAATCTAGTGGTACCGAGATATGCATTCGCAATGGAAAGAAATGCTGGATCTGGTATTATCATTTCAGATACACCAGTCCACGATTGCAATACAACTTGTCAGACACCCAAGGGTGCTATAAACACCAGCCTCCCATTTCAGAATATACATCCGATCACAATTGGAAAATGTCCAAAATATGTAAAAAGCACAAAATTGAGACTGGCCACAGGATTGAGGAATGTCCCGTCTATTCAATCTAGAGGCCTATTTGGGGCCATTGCCGGCTTCATTGAAGGGGGGTGGACAGGGATGGTAGATGGATGGTACGGTTATCACCATCAAAATGAGCAGGGGTCAGGATATGCAGCCGACCTGAAGAGCACACAGAATGCCATTGACAAGATTACTAACAAAGTAAATTCTGTTATTGAAAAGATGAATACACAGTTCACAGCAGTAGGTAAAGAGTTCAACCACCTGGAAAAAAGAATAGAGAATTTAAATAAAAAAGTTGATGATGGTTTCCTGGACATTTGGACTTACAATGCCGAACTGTTGGTTCTATTGGAAAATGAAAGAACTTTGGACTACCACGATTCAAATGTGAAGAACTTATATGAAAAGGTAAGAAACCAGTTAAAAAACAATGCCAAGGAAATTGGAAATGGCTGCTTTGAATTTTACCACAAATGCGATAACACGTGCATGGAAAGTGTCAAAAATGGGACTTATGACTACCCAAAATACTCAGAGGAAGCAAAATTAAACAGAGAAGAAATAGATGGGGTAAAACTGGAATCAACAAGGATTTACCAGATTTTGGCGATCTATTCAACTGTCGCCAGTTCATTGGTATTGGTAGTCTCCCTGGGGGCAATCAGTTTTTGGATGTGCTCTAATGGGTCTCTACAGTGTAGAATATGTATTTAA

>LC638333.1Influenza A virus H1N101701

ATGAAGGCAATACTAGTAGTTCTGCTATATACATTTGCAACCGCAAATGCAGACACATTATGTATAGGTTATCATGCGAACAATTCAACAGACACTGTAGACACAGTACTAGAAAAGAATGTAACAGTAACACACTCTGTTAATCTTCTAGAAGACAAGCATAACGGAAAACTATGCAAACTAAGAGGGGTAGCCCCATTGCATTTGGGTAAATGTAACATTGCTGGCTGGATCCTGGGAAATCCAGAGTGTGAATCACTCTCCACAGCAAGCTCATGGTCCTACATTGTGGAAACATCTAGTTCAGACAATGGAACGTGTTACCCAGGAGATTTCATCGATTATGAGGAGCTAAGAGAGCAATTGAGCTCAGTGTCATCATTTGAAAGGTTTGAGATATTCCCCAAGACAAGTTCATGGCCCAATCATGACTCGAACAAAGGTGTAACGGCAGCATGTCCTCATGCTGGAGCAAAAAGCTTCTACAAAAATTTAATATGGCTAGTTAAAAAAGGAAATTCATACCCAAAGCTCAGCAAATCCTACATTAATGATAAAGGGAAAGAAGTCCTCGTGCTATGGGGCATTCACCATCCATCTACTACTGCTGACCAACAAAGTCTCTATCAGAATGCAGATACATATGTTTTTGTGGGGACATCAAGATACAGCAAGAAGTTCAAGCCGGAAATAGCAATAAGACCCAAAGTGAGGGATCAAGAAGGGAGAATGAACTATTACTGGACACTAGTAGAGCCGGGAGACAAAATAACATTCGAAGCAACTGGAAATCTAGTGGTACCGAGATATGCATTCGCAATGGAAAGAAATGCTGGATCTGGTATTATCATTTCAGATACACCAGTCCACGATTGCAATACAACTTGTCAGACACCCAAGGGTGCTATAAACACCAGCCTCCCATTTCAAAATATACATCCGATCACAATTGGAAAATGTCCAAAATATGTAAAAAGCACAAAATTGAGACTGGCCACAGGATTGAGGAATGTCCCGTCTATTCAATCTAGAGGCCTATTTGGGGCCATTGCCGGCTTCATTGAAGGGGGGTGGACAGGGATGGTAGATGGATGGTACGGTTATCACCATCAAAATGAGCAGGGGTCAGGATATGCAGCCGACCTGAAGAGCACACAGAATGCCATTGACAAGATTACTAACAAAGTAAATTCTGTTATTGAAAAGATGAATACACAGTTCACAGCAGTAGGTAAAGAGTTCAACCACCTGGAAAAAAGAATAGAGAATTTAAATAAAAAAGTTGATGATGGTTTCCTGGACATTTGGACTTACAATGCCGAACTGTTGGTTCTATTGGAAAATGAAAGAACTTTGGACTACCACGATTCAAATGTGAAGAACTTATATGAAAAAGTAAGAAACCAGTTAAAAAACAATGCCAAGGAAATTGGAAATGGCTGCTTTGAATTTTACCACAAATGCGATAACACGTGCATGGAAAGTGTCAAAAATGGGACTTATGACTACCCAAAATACTCAGAGGAAGCAAAATTAAACAGAGAAGAAATAGATGGGGTAAAGCTGGAATCAACAAGGATTTACCAGATTTTGGCGATCTATTCAACTGTCGCCAGTTCATTGGTACTGGTAGTCTCCCTGGGGGCAATCAGTTTTTGGATGTGCTCTAATGGGTCTCTACAGTGTAGAATATGTATTTAA

>LC638332.1Influenza A virus H1N101701

ATGAAGGCAATACTAGTAGTTCTGCTATATACATTTGCAACCGCAAATGCAGACACATTATGTATAGGTTATCATGCGAACAATTCAACAGACACTGTAGACACAGTACTAGAAAAGAATGTAACAGTAACACACTCTGTTAATCTTCTAGAAGACAAGCATAACGGGAAACTATGCAAACTAAGAGGGGTAGCCCCATTGCATTTGGGTAAATGTAACATTGCTGGCTGGATCCTGGGAAATCCAGAGTGTGAATCACTCTCCACAGCAAGCTCATGGTCCTACATTGTGGAAACATCTAGTTCAGACAATGGAACGTGTTACCCAGGAGATTTCATCGATTATGAGGAGCTAAGAGAGCAATTGAGCTCAGTGTCATCATTTGAAAGGTTTGAGATATTCCCCAAGACAAGTTCATGGCCCAATCATGACTCGAACAAAGGTGTAACGGCAGCATGTCCTCATGCTGGAGCAAAAAGCTTCTACAAAAATTTAATATGGCTAGTTAAAAAAGGAAATTCATACCCAAAGCTCAGCAAATCCTACATTAATGATAAAGGGAAAGAAGTCCTCGTGCTATGGGGCATTCACCATCCATCTACTACTGCTGACCAACAAAGTCTCTATCAGAATGCAGATACATATGTTTTTGTGGGGACATCAAGATACAGCAAGAAGTTCAAGCCGGAAATAGCAATAAGACCCAAAGTGAGGGATCAAGAAGGGAGAATGAACTATTACTGGACACTAGTAGAGCCGGGAGACAAAATAACATTCGAAGCAACTGGAAATCTAGTGGTACCGAGATATGCATTCGCAATGGAAAGAAATGCTGGATCTGGTATTATCATTTCAGATACACCAGTCCACGATTGCAATACAACTTGTCAGACACCCAAGGGTGCTATAAACACCAGCCTCCCATTTCAAAATATACATCCGATCACAATTGGAAAATGTCCAAAATATGTAAAAAGCACAAAATTGAGACTGGCCACAGGATTGAGGAATGTCCCGTCTATTCAATTTAGAGGCCTATTTGGGGCCATTGCCGGCTTCATTGAAGGGGGGTGGACAGGGATGGTAGATGGATGGTACGGTTATCACCATCAAAATGAGCAGGGGTCAGGATATGCAGCCGACCTGAAGAGCACACAGAATGCCATTGACAAGATTACTAACAAAGTAAATTCTGTTATTGAAAAGATGAATACACAGTTCACAGCAGTAGGTAAAGAGTTCAACCACCTGGAAAAAAGAATAGAGAATTTAAATAAAAAAGTTGATGATGGTTTCCTGGACATTTGGACTTACAATGCCGAACTGTTGGTTCTATTGGAAAATGAAAGAACTTTGGACTACCACGATTCAAATGTGAAGAACTTATATGAAAAAGTAAGAAACCAGTTAAAAAACAATGCCAAGGAAATTGGAAATGGCTGCTTTGAATTTTACCACAAATGCGATAACACGTGCATGGAAAGTGTCAAAAATGGGACTTATGACTACCCAAAATACTCAGAGGAAGCAAAATTAAACAGAGAAGAAATAGATGGGGTAAAGCTGGAATCAACAAGGATTTACCAGATTTTGGCGATCTATTCAACTGTCGCCAGTTCATTGGTACTGGTAGTCTCCCTGGGGGCAATCAGTTTTTGGATGTGCTCTAATGGGTCTCTACAGTGTAGAATATGTATTTAA

>LC638331.1Influenza A virus H1N101701

ATGAAGGCAATACTAGTAGTTCTGCTATATACATTTGCAACCGCAAATGCAGACACATTATGTATAGGTTACCATGCGAACAATTCAACAGACACTGTAGACACAGTACTAGAAAAGAATGTAACAGTAACACACTCTGTTAATCTTCTAGAAGACAAGCATAACGGGAAACTATGCAAACTAAGAGGGGTAGCCCCATTGCATTTGGGTAAATGTAACATTGCTGGCTGGATCCTGGGAAATCCAGAGTGTGAATCACTCTCCACAGCAAGCTCATGGTCCTACATTGTGGAAACATCTAGTTCAGACAATGGAACGTGTTACCCAGGAGATTTCATCGATTATGAGGAGCTAAGAGAGCAATTGAGCTCAGTGTCATCATTTGAAAGGTTTGAGATATTCCCCAAGACAAGTTCATGGCCCAATCATGACTCGAACAAAGGTGTAACGGCAGCATGTCCTCATGCTGGAGCAAAAGGCTTCTACAAAAATTTAATATGGCTAGTTAAAAAAGGAAATTCATACCCAAAGCTCAGCAAATCCTACATTAATGATAAAGGGAAAGAAGTCCTCGTGCTATGGGGCATTCACCATCCATCTACTACTGCTGACCAACAAAGTCTCTATCAGAATGCAGATACATATGTTTTTGTGGGGACATCAAGATACAGCAAGAAGTTCAAGCCGGAAATAGCAATAAGACCCAAAGTGAGGGATCAAGAAGGGAGAATGAACTATTACTGGACACTAGTAGAGCCGGGAGACAAAATAACATTCGAAGCAACTGGAAATCTAGTGGTACCGAGATATGCATTCGCAATGGAAAGAAATGCTGGATCTGGTATTATCATTTCAGATACACCAGTCCACGATTGCAATACAACTTGTCAGACACCCAAGGGTGCTATAAACACCAGCCTCCCATTTCAGAATATACATCCGATCACAATTGGAAAATGTCCAAAATATGTAAAAAGCACAAAATTGAGACTGGCCACAGGATTGAGGAATGTCCCGTCTATTCAATCTAGAGGCCTATTTGGGGCCATTGCCGGCTTCATTGAAGGGGGGTGGACAGGGATGGTAGATGGATGGTACGGTTATCACCATCAAAATGAGCAGGGGTCAGGATATGCAGCCGACCTGAAGAGCACACAGAATGCCATTGACAAGATTACTAACAAAGTAAATTCTGTTATTGAAAAGATGAATACACAGTTCACAGCAGTAGGTAAAGAGTTCAACCACCTGGAAAAAAGAATAGAGAATTTAAATAAAAAAGTTGATGATGGTTTCCTGGACATTTGGACTTACAATGCCGAACTGTTGGTTCTATTGGAAAATGAAAGAACTTTGGACTACCACGATTCAAATGTGAAGAACTTATATGAAAAGGTAAGAAACCAGTTAAAAAACAATGCCAAGGAAATTGGAAATGGCTGCTTTGAATTTTACCACAAATGCGATAACACGTGCATGGAAAGTGTCAAAAATGGGACTTATGACTACCCAAAATACTCAGAGGAAGCAAAATTAAACAGAGAAGAAATAGATGGGGTAAAACTGGAATCAACAAGGATTTACCAGATTTTGGCGATCTATTCAACTGTCGCCAGTTCATTGGTATTGGTAGTCTCCCTGGGGGCAATCAGTTTTTGGATGTGCTCTAATGGGTCTCTACAGTGTAGAATATGTATTTAA

>LC638330.1Influenza A virus H1N101701

ATGAAGGCAATACTAGTAGTTCTGCTATATACATTTGCAACCGCAAATGCAGACACATTATGTATAGGTTATCATGCGAACAATTCAACAGACACTGTAGACACAGTACTAGAAAAGAATGTAACAGTAACACACTCTGTCAATCTTCTAGAAGACAAACATAACGGAAAACTATGCAAACTAAGAGGGGTAGCCCCATTGCATTTGGGTAAATGTAACATTGCTGGCTGGATCCTGGGAAATCCAGAGTGTGAATCACTCTCCACAGCAAGCTCATGGTCCTACATTGTGGAAACATCTAGTTCAGACAATGGAACGTGTTACCCAGGAGATTTCATCGATTATGAGGAGCTAAGAGAGCAATTGAGCTCAGTGTCATCATTTGAAAGGTTTGAGATATTCCCCAAGACAAGTTCATGGCCCAATCATGACTCGAACAAAGGTGTAACGGCAGCATGTCCTCATGCTGGAGCAAAAAGCTTCTACAAAAATTTAATATGGCTAGTTAAAAAAGGAAATTCATACCCAAAGCTCAGCAAATCCTACATTAATGATAAAGGGAAAGAAGTCCTCGTGCTATGGGGCATTCACCATCCATCTACTACTGCTGACCAACAAAGTCTCTATCAGAATGCAGATACATATGTTTTTGTGGGAACATCAAGATACAGCAAGAAGTTCAAACCGGAAATAGCAATAAGACCCAAAGTGAGGGATCAAGAAGGGAGAATGAACTATTACTGGACACTAGTAGAGCCGGGAGACAAAATAACATTCGAAGCAACTGGAAATCTAGTGGTACCGAGATATGCATTCGCAATGGAAAGAAATGCTGGATCTGGTATTATCATTTCAGATACACCAGTCCACGATTGCAATACAACTTGTCAGACACCCAAGGGTGCTATAAACACCAGCCTCCCATTTCAAGATATACATCCGATCACAATTGGAAAATGTCCAAAATATGTAAAAAGCACAAAATTGAGACTGGCCACAGGATTGAGGAATGTCCCGTCTATTCAATCTAGAGGCCTATTTGGGGCCATTGCCGGCTTCATTGAAGGGGGGTGGACAGGGATGGTAGATGGATGGTACGGTTATCACCATCAAAATGAGCAGGGGTCAGGATATGCAGCCGACCTGAAGAGCACACAGAATGCCATTGACAAGATTACTAACAAAGTAAATTCTGTTATTGAAAAGATGAATACACAGTTCACAGCAGTAGGTAAAGAGTTCAACCACCTGGAAAAAAGAATAGAGAATTTAAATAAAAAAGTTGATGATGGTTTCCTGGACATTTGGACTTACAATGCCGAACTGTTGGTTCTATTGGAAAATGAAAGAACTTTGGACTACCACGATTCAAATGTGAAGAACTTATATGAAAAAGTAAGAAACCAGTTAAAAAACAATGCCAAGGAAATTGGAAATGGCTGCTTTGAATTTTACCACAAATGCGATAACACGTGCATGGAAAGTGTCAAAAATGGGACTTATGACTACCCAAAATACTCAGAGGAAGCAAAATTAAACAGAGAAAAAATAGATGGGGTAAAGCTGGAATCAACAAGGATTTACCAGATTTTGGCGATCTATTCAACTGTCGCCAGTTCATTGGTACTGGTAGTCTCCCTGGGGGCAATCAGTTTTTGGATGTGCTCTAATGGGTCTCTACAGTGTAGAATATGTATTTAA

>LC638329.1Influenza A virus H1N101701

ATGAAGGCAATACTAGTAGTTCTGCTATATACATTTGCAACCGCAAATGCAGACACATTATGTATAGGTTACCATGCGAACAATTCAACAGACACTGTAGACACAGTACTAGAAAAGAATGTAACAGTAACACACTCTGTTAATCTTCTAGAAGACAAGCATAACGGGAAACTATGCAAACTAAGAGGGGTAGCCCCATTGCATTTGGGTAAATGTAACATTGCTGGCTGGATCCTGGGAAATCCAGAGTGTGAATCACTCTCCACAGCAAGCTCATGGTCCTACATTGTGGAAACATCTAGTTCAGACAATGGAACGTGTTACCCAGGAGATTTCATCGATTATGAGGAGCTAAGAGAGCAATTGAGCTCAGTGTCATCATTTGAAAGGTTTGAGATATTCCCCAAGACAAGTTCATGGCCCAATCATGACTCGAACAAAGGTGTAACGGCAGCATGTCCTCATGCTGGAGCAAAAGGCTTCTACAAAAATTTAATATGGCTAGTTAAAAAAGGAAATTCATACCCAAAGCTCAGCAAATCCTACATTAATGATAAAGGGAAAGAAGTCCTCGTGCTATGGGGCATTCACCATCCATCTACTACTGCTGACCAACAAAGTCTCTATCAGAATGCAGATACATATGTTTTTGTGGGGACATCAAGATACAGCAAGAAGTTCAAGCCGGAAATAGCAATAAGACCCAAAGTGAGGGATCAAGAAGGGAGAATGAACTATTACTGGACACTAGTAGAGCCGGGAGACAAAATAACATTCGAAGCAACTGGAAATCTAGTGATACCGAGATATGCATTCGCAATGGAAAGAAATGCTGGATCTGGTATTATCATTTCAGATACACCAGTCCACGATTGCAATACAACTTGTCAGACACCCAAGGGTGCTATAAACACCAGCCTCCCATTTCAGAATATACATCCGATCACAATTGGAAAATGTCCAAAATATGTAAAAAGCACAAAATTGAGACTGGCCACAGGATTGAGGAATGTCCCGTCTATTCAATCTAGAGGCCTATTTGGGGCCATTGCCGGCTTCATTGAAGGGGGGTGGACAGGGATGGTAGATGGATGGTACGGTTATCACCATCAAAATGAGCAGGGGTCAGGATATGCAGCCGACCTGAAGAGCACACAGAATGCCATTGACAAGATTACTAACAAAGTAAATTCTGTTATTGAAAAGATGAATACACAGTTCACAGCAGTAGGTAAAGAGTTCAACCACCTGGAAAAAAGAATAGAGAATTTAAATAAAAAAGTTGATGATGGTTTCCTGGACATTTGGACTTACAATGCCGAACTGTTGGTTCTATTGGAAAATGAAAGAACTTTGGACTACCACGATTCAAATGTGAAGAACTTATATGAAAAGGTAAGAAACCAGTTAAAAAACAATGCCAAGGAAATTGGAAATGGCTGCTTTGAATTTTACCACAAATGCGATAACACGTGCATGGAAAGTGTCAAAAATGGGACTTATGACTACCCAAAATACTCAGAGGAAGCAAAATTAAACAGAGAAGAAATAGATGGGGTAAAACTGGAATCAACAAGGATTTACCAGATTTTGGCGATCTATTCAACTGTCGCCAGTTCATTGGTATTGGTAGTCTCCCTGGGGGCAATCAGTTTTTGGATGTGCTCTAATGGGTCTCTACAGTGTAGAATATGTATTTAA

>LC638328.1Influenza A virus H1N101701

ATGAAGGCAATACTAGTAGTTCTGCTATATACATTTGCAACCGCAAATGCAGACACATTATGTATAGGTTATCATGCGAACAATTCAACAGACACTGTAGACACAGTACTAGAAAAGAATGTAACAGTAACACACTCTGTCAATCTTCTAGAAGACAAGCATAACGGAAAACTATGCAAACTAAGAGGGGTAGCCCCATTGCATTTGGGTAAATGTAACATTGCTGGCTGGATCCTGGGAAATCCAGAGTGTGAATCACTCTCCACAGCAAGCTCATGGTCCTACATTGTGGAAACATCTAGTTCAGACAATGGAACGTGTTACCCAGGAGATTTCATCGATTATGAGGAGCTAAGAGAGCAATTGAGCTCAGTGTCATCATTTGAAAGGTTTGAGATATTCCCCAAGACAAGTTCATGGCCCAATCATGACTCGAACAAAGGTGTAACGGCAGCATGTCCTCATGCTGGAGCAAAAAGCTTCTACAAAAATTTAATATGGCTAGTTAAAAAAGGAAATTCATACCCAAAGCTCAGCAAATCCTACATTAATGATAAAGGGAAAGAAGTCCTCGTGCTATGGGGCATTCACCATCCATCTACTACTGCTGACCAACAAAGTCTCTATCAGAATGCAGATACATATGTTTTTGTGGGAACATCAAGATACAGCAAGAAGTTCAAACCGGAAATAGCAATAAGACCCAAAGTGAGGGATCAAGAAGGGAGAATGAACTATTACTGGACACTAGTAGAGCCGGGAGACAAAATAACATTCGAAGCAACTGGAAATCTAGTGGTACCGAGATATGCATTCGCAATGGAAAGAAATGCTGGATCTGGTATTATCATTTCAGATACACCAGTCCACGATTGCAATACAACTTGTCAGACACCCAAGGGTGCTATAAACACCAGCCTCCCATTTCAAGATATACATCCGATCACAATTGGAAAATGTCCAAAATATGTAAAAAGCACAAAATTGAGACTGGCCACAGGATTGAGGAATGTCCCGTCTATTCAATCTAGAGGCCTATTTGGGGCCATTGCCGGCTTCATTGAAGGGGGGTGGACAGGGATGGTAGATGGATGGTACGGTTATCACCATCAAAATGAGCAGGGGTCAGGATATGCAGCCGACCTGAAGAGCACACAGAATGCCATTGACAAGATTACTAACAAAGTAAATTCTGTTATTGAAAAGATGAATACACAGTTCACAGCAGTAGGTAAAGAGTTCAACCACCTGGAAAAAAGAATAGAGAATTTAAATAAAAAAGTTGATGATGGTTTCCTGGACATTTGGACTTACAATGCCGAACTGTTGGTTCTATTGGAAAATGAAAGAACTTTGGACTACCACGATTCAAATGTGAAGAACTTATATGAAAAAGTAAGAAACCAGTTAAAAAACAATGCCAAGGAAATTGGAAATGGCTGCTTTGAATTTTACCACAAATGCGATAACACGTGCATGGAAAGTGTCAAAAATGGGACTTATGACTACCCAAAATACTCAGAGGAAGCAAAATTAAACAGAGAAAAAATAGATGGGGTAAAGCTGGAATCAACAAGGATTTACCAGATTTTGGCGATCTATTCAACTGTCGCCAGTTCATTGGTACTGGTAGTCTCCCTGGGGGCAATCAGTTTTTGGATGTGCTCTAATGGGTCTCTACAGTGTAGAATATGTATTTAA

>LC638327.1Influenza A virus H1N101701
[truncated: 325,901 more chars]
