# Supplementary material for: Evolutionary trajectory and co-infection dynamics of human influenza A(H1N1) virus (2000–2025): an integrated framework informed by expert-informed bibliometrics
Source: Front Microbiol. 2026 Mar 26;17:1793244. doi: 10.3389/fmicb.2026.1793244 (PMC13064542; doi:10.3389/fmicb.2026.1793244)
Supplement: Supplementary file 8 — Table 8= Supplementary File S8 [file Table_8.doc]

2000-H1N1-strain：

**（GenBank accession no.** LC660661.1 ，LC660659.1 ，LC638240.1 ，LC638239.1 ，LC638238.1 ，LC638237.1 ，LC638236.1 ，LC638235.1 ，LC638234.1 ，LC638233.1 ，LC638232.1 ，LC638231.1 ，LC638230.1 ，LC638229.1 ，LC638228.1 ，LC638227.1 ，LC638226.1 ，LC638225.1 ，LC638224.1 ，LC638223.1 ，LC638222.1 ，LC638221.1 ，LC638220.1 ，LC638219.1 ，LC638218.1 ，LC638217.1 ，LC638216.1 ，LC638215.1 ，LC638214.1 ，LC638213.1 ，LC638212.1 ，LC638211.1 ，LC638210.1 ，LC638209.1 ，LC638208.1 ，LC638207.1 ，LC638206.1 ，LC638205.1 ，LC638204.1 ，LC638203.1 ，LC638202.1 ，LC638201.1 ，LC638200.1 ，LC638199.1 ，LC638198.1 ，LC638197.1 ，LC638196.1 ，LC638195.1 ，LC638194.1 ，LC638193.1 ，LC638192.1 ，LC638191.1 ，LC638190.1 ，LC638189.1 ，LC638188.1 ，LC638187.1 ，LC638186.1 ，LC638185.1 ，LC638184.1 ，LC638183.1 ，LC638182.1 ，LC638181.1 ，LC638180.1 ，LC638179.1 ，LC638178.1 ，LC638177.1 ，LC638176.1 ，LC638175.1 ，LC638174.1 ，LC638173.1 ，LC638172.1 ，LC638169.1 ，LC638168.1 ，LC638165.1 ，LC638162.1 ，LC638161.1 ，LC638157.1 ，LC638156.1 ，LC638153.1 ，LC638149.1 ，LC638148.1 ，LC638147.1 ，LC638146.1 ，LC638145.1 ，LC638144.1 ，LC638143.1 ，LC638142.1 ，LC638141.1 ，LC638140.1 ，LC638139.1 ，LC638138.1 ，LC638137.1 ，LC638135.1 ，LC638134.1 ，LC638132.1 ，LC638131.1 ，LC638130.1 ，LC638129.1 ，LC638128.1 ，LC638126.1 ，LC638123.1 ，LC638121.1 ，LC638120.1 ，LC638119.1 ，LC638118.1 ，LC638117.1 ，LC638116.1 ，LC638115.1 ，LC638114.1 ，LC638113.1 ，LC638112.1 ，LC638111.1 ，LC638109.1 ，LC638108.1 ，LC638107.1 ，LC638106.1 ，LC638105.1 ，LC638104.1 ，LC638103.1 ，LC638102.1 ，LC638101.1 ，LC638100.1 ，LC638099.1 ，LC638098.1 ，LC638097.1 ，LC638096.1 ，LC638093.1 ，LC638091.1 ，LC638090.1 ，LC638089.1 ，LC638088.1 ，LC638083.1 ，LC638082.1 ，LC638080.1 ，LC638079.1 ，LC638078.1 ，LC638077.1 ，LC638076.1 ，LC638075.1 ，LC638074.1 ，LC638073.1 ，LC638072.1 ，LC638071.1 ，LC638070.1 ，LC638069.1 ，LC638068.1 ，LC638066.1 ，LC638065.1 ，LC638063.1 ，LC638062.1 **）**

MKAILVVLLYTFATANADTLCIGYHANNSTDTVDTVLEKNVTVTHSVNLLEDKHNGKLCKLRGVAPLHLGKCNIAGWILGNPECESLSTASSWSYIVETSSSDNGTCYPGDFIDYEELREQLSSVSSFERFEIFPKTSSWPNHDSNKGVTAACPHAGAKSFYKNLIWLVKKGNSYPKLSKSYINDKGKEVLVLWGIHHPSTSADQQSLYQNADAYVFVGTSRYSKKFKPEIAIRPKVRDQEGRMNYYWTLVEPGDKITFEATGNLVVPRYAFAMERNAGSGIIISDTPVHDCNTTCQTPKGAINTSLPFQNIHPITIGKCPKYVKSTKLRLATGLRNVPSIQSRGLFGAIAGFIEGGWTGMVDGWYGYHHQNEQGSGYAADLKSTQNAIDEITNKVNSVIEKMNTQFTAVGKEFNHLEKRIENLNKKVDDGFLDIWTYNAELLVLLENERTLDYHDSNVKNLYEKVRSQLKNNAKEIGNGCFEFYHKCDNTCMESVKNGTYDYPKYSEEAKLNREEIDGVKLESTRIYQILAIYSTVASSLVLVVSLGAISFWMCSNGSLQCRICI

>sequence 2010-H1N1-strain

( **GenBank accession no.** LC778493.1 ， LC778491.1 ， LC778485.1 ， LC778483.1 ， LC778477.1 LC778475.1 ， LC778469.1 ， LC778467.1 ， LC778461.1 ， LC778459.1 ， LC638404.1 ， LC638403.1 ， LC638402.1 ， LC638401.1 ， LC638400.1 ， LC638399.1 ， LC638398.1 ， LC638397.1 ， LC638396.1 ， LC638395.1 ， LC638394.1 ， LC638393.1 ， LC638392.1 ， LC638391.1 ， LC638390.1 ， LC638389.1 ， LC638388.1 ， LC638387.1 ， LC638386.1 ， LC638385.1 ， LC638384.1 ， LC638383.1 ， LC638382.1 ， LC638381.1 ， LC638380.1 ， LC638379.1 ， LC638378.1 ， LC638377.1 ， LC638376.1 ， LC638373.1 ， LC638372.1 ， LC638371.1 ， LC638370.1 ， LC638369.1 ， LC638368.1 ， LC638367.1 ， LC638366.1 ， LC638365.1 ， LC638364.1 ， LC638363.1 ， LC638362.1 ， LC638361.1 ， LC638360.1 ， LC638359.1 ， LC638358.1 ， LC638357.1 ， LC638356.1 ， LC638355.1 ， LC638354.1 ， LC638353.1 ， LC638352.1 ， LC638351.1 ， LC638350.1 ， LC638349.1 ， LC638348.1 ， LC638347.1 ， LC638346.1 ， LC638345.1 ， LC638344.1 ， LC638343.1 ， LC638342.1 ， LC638341.1 ， LC638340.1 ， LC638339.1 ， LC638338.1 ， LC638337.1 ， LC638336.1 ， LC638335.1 ， LC638334.1 ， LC638333.1 ， LC638332.1 ， LC638331.1 ， LC638330.1 ， LC638329.1 ， LC638328.1 ， LC638327.1 ， LC638326.1 ， LC638325.1 ， LC638324.1 ， LC638323.1 ， LC638322.1 ， LC638321.1 ， LC638320.1 ， LC638319.1 ， LC638318.1 ， LC638317.1 ， LC638316.1 ， LC638315.1 ， LC638314.1 ， LC638313.1 ， LC638312.1 ， LC638311.1 ， LC638310.1 ， LC638309.1 ， LC638308.1 ， LC638307.1 ， LC638306.1 ， LC638305.1 ， LC638304.1 ， LC638303.1 ， LC638302.1 ， LC638301.1 ， LC638300.1 ， LC638299.1 ， LC638298.1 ， LC638297.1 ， LC638296.1 ， LC638295.1 ， LC638294.1 ， LC638293.1 ， LC638292.1 ， LC638291.1 ， LC638290.1 ， LC638289.1 ， LC638288.1 ， LC638287.1 ， LC638286.1 ， LC638285.1 ， LC638284.1 ， LC638283.1 ， LC638282.1 ， LC638281.1 ， LC638280.1 ， LC638279.1 ， LC638278.1 ， LC638277.1 ， LC638276.1 ， LC638275.1 ， LC638274.1 ， LC638273.1 ， LC638272.1 ， LC638271.1 ， LC638270.1 ， LC638269.1 ， LC638268.1 ， LC638267.1 ， LC638266.1 ， LC638265.1 ， LC638264.1 ， LC638263.1 ， LC638262.1 ， LC638261.1 ， LC638260.1 ， LC638259.1 ， LC638258.1 ， LC638257.1 ， LC638256.1 ， LC638255.1 ， LC638254.1 ， LC638253.1 ， LC638252.1 ， LC638251.1 ， LC638250.1 ， LC638249.1 ， LC638248.1 ， LC638247.1 ， LC638246.1 ， LC638245.1 ， LC638244.1 ， LC638243.1 ， LC638242.1 ， LC638241.1 ， LC638164.1 ， LC638163.1 ， LC638160.1 ， LC638159.1 ， LC638158.1 ， LC638155.1 ， LC638154.1 ， LC638152.1 ， LC638151.1 ， LC638150.1 ， LC638136.1 ， LC638133.1 ， LC638127.1 ， LC638125.1 ， LC638124.1 ， LC638122.1 ， LC638110.1 ， LC638095.1 ， LC638094.1 ， LC638092.1 ， LC638087.1 ， LC638086.1 ， LC638085.1 ， LC638084.1 ， LC638081.1 ， LC638067.1 ， LC638064.1 ， LC638061.1 ， LC638060.1 ， LC638059.1 ， LC638058.1 ， LC638057.1 ， LC638056.1 ， LC638055.1 ， LC638054.1 ， LC638053.1 ， LC637996.1 ， LC637997.1 ， LC637998.1 ， LC637999.1 ， LC638000.1 ， LC638001.1 ， LC638002.1 ， LC638003.1 ， LC638004.1 ， LC638005.1 ， LC638006.1 ， LC638007.1 ， LC638008.1 ， LC638009.1 ， LC638010.1 ， LC638011.1 ， LC638012.1 ， LC638013.1 ， LC638014.1 ， LC638015.1 ， LC638016.1 ， LC638017.1 ， LC638018.1 ， LC638019.1 ， LC638052.1 ， LC638051.1 ， LC638050.1 ， LC638049.1 ， LC638048.1 ， LC638047.1 ， LC638046.1 ， LC638045.1 ， LC638044.1 ， LC638043.1 ， LC638042.1N ， LC638041.1 ， LC638040.1 ， LC638039.1 ， LC638038.1 ， LC638037.1 ， LC638036.1 ， LC638035.1 ， LC638034.1 ， LC638026.1 ， LC638025.1 ， LC638024.1 ， LC638023.1 ， LC638022.1 ， LC638021.1 ， LC638020.1 )

MKAILVVLLYTFATANADTLCIGYHANNSTDTVDTVLEKNVTVTHSVNLLEDKHNGKLCKLRGVAPLHLGKCNIAGWILGNPECESLSTASSWSYIVETSSSDNGTCYPGDFIDYEELREQLSSVSSFERFEIFPKTSSWPNHDSNKGVTAACPHAGAKSFYKNLIWLVKKGNSYPKLSKSYINDKGKEVLVLWGIHHPSTTADQQSLYQNADTYVFVGTSRYSKKFKPEIAIRPKVRDQEGRMNYYWTLVEPGDKITFEATGNLVVPRYAFAMERNAGSGIIISDTPVHDCNTTCQTPK

GAINTSLPFQNIHPITIGKCPKYVKSTKLRLATGLRNVPSIQSRGLFGAIAGFIEGGWTGMVDGWYGYHHQNEQGSGYAADLKSTQNAIDKITNKVNSVIEKMNTQFTAVGKEFNHLEKRIENLNKKVDDGFLDIWTYNAELLVLLENERTLDYHDSNVKNLYEKVRNQLKNNAKEIGNGCFEFYHKCDNTCMESVKNGTYDYPKYSEEAKLNREKIDGVKLESTRIYQILAIYSTVASSLVLVVSLGAISFWMCSNGSLQCRICI

>sequence 2020-H1N1-strain

( **GenBank accession no.** ,LC 662542.1 , LC 662540.1 , LC 638375.1 , LC 638374.1 , LC 638171.1 , LC 638170.1 , LC 638167.1 , LC 638166.1 , LC 638033.1 , LC 638032.1 , LC 638031.1 , LC 638030.1 , LC 638029.1 , LC 638028.1 , LC 638027.1）

MKAILVVLLYTFTTANADTLCIGYHANNSTDTVDTVLEKNVTVTHSVNLLEDKHNGKLCKLRGVAPLHLGKCNIAGWILGNPECESLSTARSWSYIVETSNSDNGTCYPGDFINYEELREQLSSVSSFERFEIFPKTSSWPNHDSDKGVTAACPHAGAKSFYKNLIWLVKKGNSYPKLNQTYINDKGKEVLVLWGIHHPPTIAAQESLYQNADAYVFVGTSRYSKKFKPEIATRPKVRDQEGRMNYYWTLVEPGDKITFEATGNLVVPRYAFTMERDAGSGIIISDTPVHDCNTTCQTPE

GAINTSLPFQNVHPITIGKCPKYVKSTKLRLATGLRNVPSIQSRGLFGAIAGFIEGGWTGMVDGWYGYHHQNEQGSGYAADLKSTQNAIDKITNKVNSVIEKMNTQFTAVGKEFNHLEKRIENLNKKVDDGFLDIWTYNAELLVLLENERTLDYHDSNVKNLYEKVRNQLKNNAKEIGNGCFEFYHKCDNTCMESVKNGTYDYPKYSEEAKLNREKIDGVKLESTRIYQILAIYSTVASSLVLVVSLGAISFWMCSNGSLQCRICI
