# Supplementary material for: Evolutionary trajectory and co-infection dynamics of human influenza A(H1N1) virus (2000–2025): an integrated framework informed by expert-informed bibliometrics
Source: Front Microbiol. 2026 Mar 26;17:1793244. doi: 10.3389/fmicb.2026.1793244 (PMC13064542; doi:10.3389/fmicb.2026.1793244)
Supplement: Supplementary file 11 — Table 11= Supplementary File S11 (Supplementary Figures S5–S8) [file Table_11.doc]

To identify potential mutation-prone sites in the H1N1 influenza virus HA protein, we performed sequence alignment of all local H1N1 HA protein sequences collected from 2000 to 2025. The analysis revealed that several HA residues—including S13, S36, S91, S101, S114, S179, S180, S181, S200, S202, S214, S233, S273, S300, S311, S312, S391, S468, and S516—exhibited less than 85% conservation and displayed multiple forms of amino acid substitutions (Figure S5). Among these, S202 and S214 are located within the HA1 antigenic region, where mutations may alter the antigenicity of H1N1. Furthermore, mutations at S202, S214, and S233 occur within the receptor-binding site (RBS), suggesting that changes at these positions could potentially influence viral infectivity and host cell binding affinity.
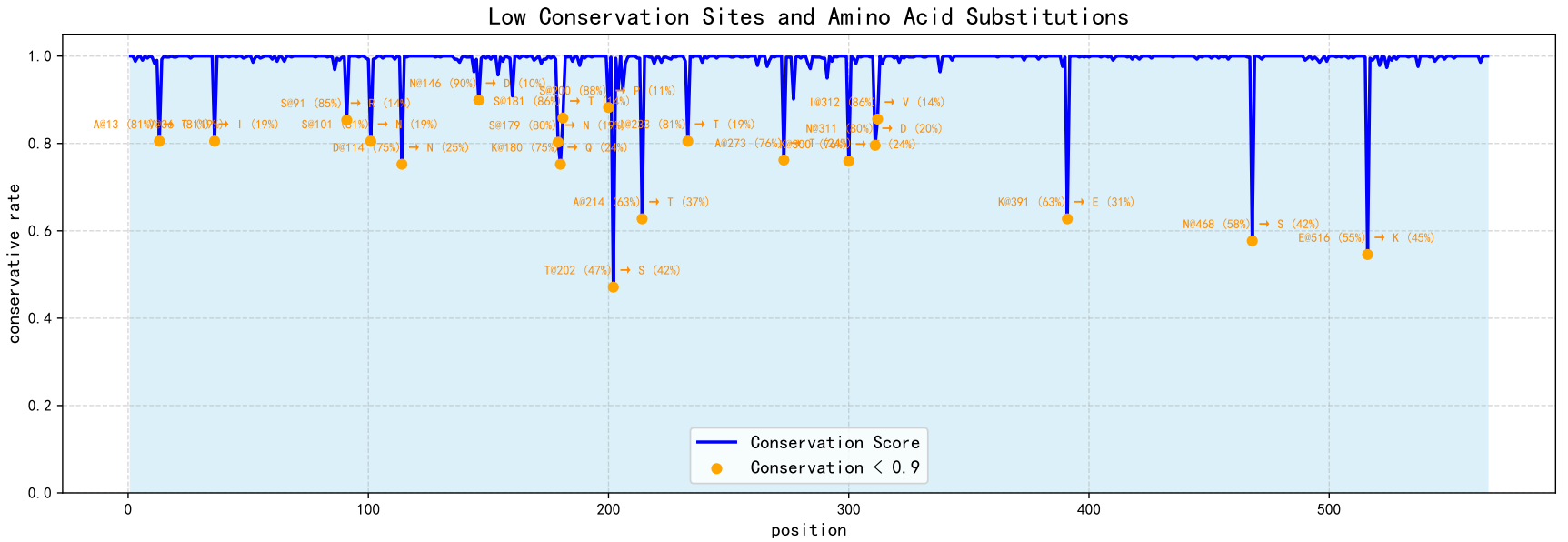


**Figure S5.** Conservation analysis of HA protein amino acid residues in circulating H1N1 influenza virus strains from 2000 to 2025.

To assess the temporal dynamics of amino acid conservation in the H1N1 influenza virus HA protein, we analyzed the year-by-year conservation of HA residues. Between 2000 and 2009, potential mutation-prone sites were observed at S36, S214, and S391. Notably, variation at the S214 site may affect both viral antigenicity and infectivity. However, during this period, the overall changes in the HA protein were relatively minor, indicating a slower evolutionary rate (Figure S6).


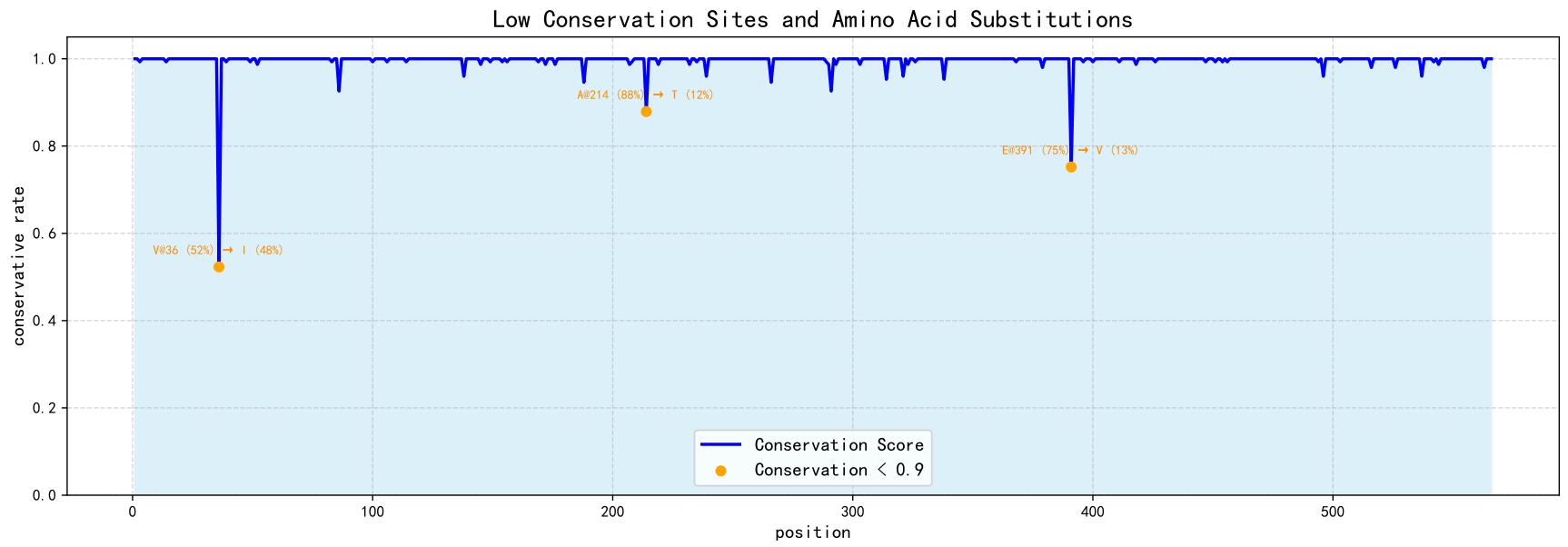


**Figure S6.** Analysis of amino acid conservation in the HA protein of circulating H1N1 influenza virus strains from 2000 to 2009.

Compared with 2000–2009, circulating H1N1 influenza virus HA proteins from 2010 to 2019 exhibited dynamic changes at multiple amino acid positions. Specifically, the N-terminal residues S13, S91, S101, S114, S179, S180, S181, S182, S200, S214, S233, S273, S277, S300, and S311 showed less than 85% conservation. Notably, substantial changes were observed within both antigenic sites and the receptor-binding site (RBS) regions (Figure S7). These alterations may represent a major factor underlying the rapid and widespread circulation of H1N1 variants following 2010.


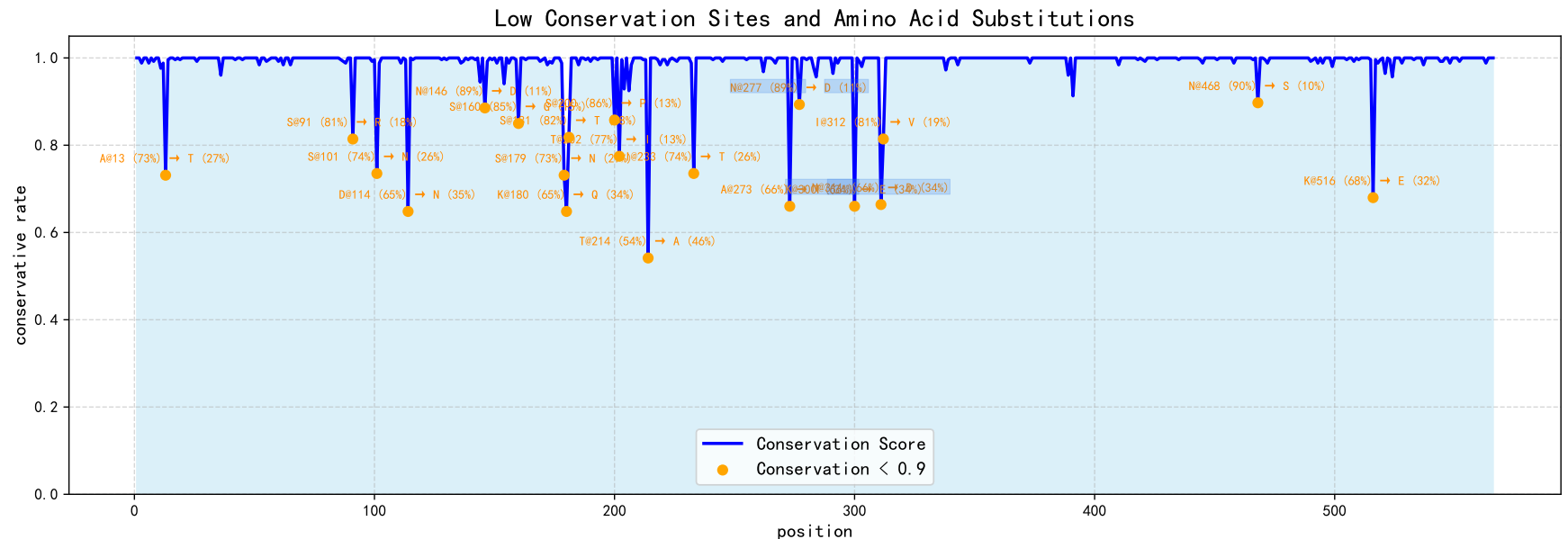


**Figure S7.** Analysis of amino acid conservation in the HA protein of circulating H1N1 influenza virus strains from 2010 to 2019.

After 2020, circulating H1N1 influenza virus HA proteins exhibited low conservation only at residue S497, indicating that the virus had largely stabilized. Notably, residues S48, S103, S154, S222, S319, S391, and S490 still show potential for substantial variation, suggesting that these positions may serve as hidden sites for future viral evolution (Figure S8).


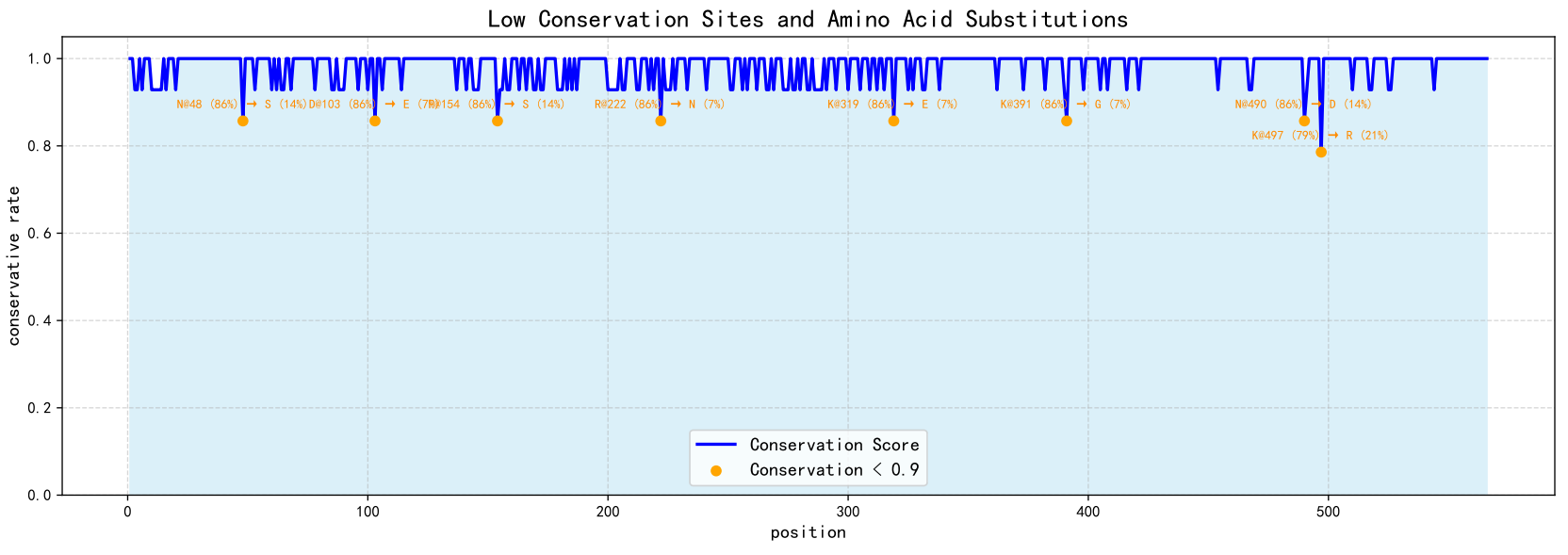


**Figure S8.** Analysis of amino acid conservation in the HA protein of circulating H1N1 influenza virus strains from 2020 to 2025.
